# Dance versus other exercise modalities in mild cognitive impairment and dementia: comparative efficacy from a systematic review and bayesian network meta-analysis

**DOI:** 10.3389/fphys.2026.1782774

**Published:** 2026-03-25

**Authors:** Ye Zhao, Dan Tao, Bian Zhao, Wei Li, Xuehai Lv, Shuai Shao, Yan Sun, Xuefeng Zhao, Alistair Cole, Yang Gao

**Affiliations:** 1 Department of Physical Education, China Medical University, Shenyang, China; 2 School of Chinese Medicine, Hong Kong Baptist University, Hong Kong SAR, China; 3 The Institute of Sports Medicine of China, Beijing, China; 4 Department of Rehabilitation, Handan Central Hospital, Handan, China; 5 Section of Pulmonary, Critical Care, and Sleep Medicine, Yale School of Medicine, Yale University, New Haven, CT, United States; 6 Department of Social Work, The Chinese University of Hong Kong, Hong Kong SAR, China; 7 Department of Martial Arts and Dance, Shenyang Sport University, Shenyang, China; 8 Department of Political Science, Sciences Po Lyon, University of Lyon, Lyon, France; 9 Department of Sports and Health Sciences, Academy of Wellness and Human Development, Hong Kong Baptist University, Hong Kong SAR, China

**Keywords:** aging, dance, dementia, exercise, mild cognitive impairment, network meta-analysis

## Abstract

**Background:**

Dance is a non-pharmacological option for people with cognitive impairment, yet its comparative advantage over other exercise modalities is still uncertain. This study compared and ranked dance versus other exercise in this population.

**Methods:**

A systematic review and network meta-analysis of randomized controlld trials was conducted using five databases. Primary outcomes were cognitive function, psychological wellbeing, and physical performance. Standardized mean differences and *p*-values were calculated using pairwise and network meta-analysis within a random-effects model. PROSPERO (CRD42024549996).

**Results:**

The systematic review included 209 randomized controlled trials (3,773 participants). Pairwise meta-analyses identified several statistically significant effects for dance interventions. In the network meta-analysis, dance showed no statistically significant advantages over other modalities for any outcome. Nevertheless, for global cognition, dance ranked highest on MoCA by the Surface Under the Cumulative Ranking Curve. For working memory and attention outcomes, dance ranked first or second. For physical performance and mood, it generally ranked second or mid-range. For executive function, speed/attention control, and mobility, dance ranked lower. Several favorable Surface Under the Cumulative Ranking Curve results were driven by single dance studies, warranting caution in interpretation.

**Conclusion:**

Exercise yields broad benefits for individuals with cognitive impairment. Although dance was not superior across all outcomes, it ranked favorably on selected measures and performed competitively, sometimes on sparse evidence. Given its integrative combination of movement, rhythm, cognitive engagement, and social interaction, dance remains a promising, underutilized option for cognitive rehabilitation, warranting wider implementation and more robust head-to-head trials.

## 1 Introduction

In 2021, neurological conditions affected over three billion people worldwide, constituting a leading cause of disease burden and disability. Within this spectrum, neurodegenerative diseases, which are strongly associated with aging represent a substantial contributor, with an estimated 500 million people affected by 2023 (Hao et al., 2023; The World Health Organization, 2024). Among common neurological disorders, dementia ranks fourth in terms of prevalence and impact (The World Health Organization, 2024). As the populations age worldwide, cognitive impairment, encompassing mild cognitive impairment (MCI) and dementia, has emerged as a pressing public health challenge, attracting growing attention from clinicians, researchers, and policymakers alike (Huang et al., 2022).

Dementia comprises a spectrum of disorders characterized by gradual deterioration in cognitive abilities—memory, language, problem-solving, and other executive abilities—severe enough to disrupt everyday functioning (Alzheimer’s and Association, 2025). It ranks among the foremost causes of disability and dependence in later life, placing heavy emotional, social, and financial burdens on families and society (Voinescu et al., 2021). In 2019, an estimated 57 million people worldwide were affected, and projections suggest this number will reach about 153 million by 2050 (Livingston et al., 2024). Risk factors span lower educational attainment, hearing loss, hypertension, smoking, depression, diabetes, sedentary behavior, traumatic brain injury, harmful alcohol use, social isolation, and air pollution; more recently, uncorrected vision impairment and elevated low-density lipoprotein (LDL) cholesterol have also been implicated (Livingston et al., 2024). Among these, physical inactivity is highly modifiable, making it a prime target for dementia prevention efforts.

MCI occupies a key position along the neurodegenerative continuum, representing an intermediate stage between normative age-related cognitive decline and dementia (Tao et al., 2023b). Individuals with MCI exhibit deficits in memory, language, attention, and judgment, yet these are typically insufficient to cause marked dependence in everyday activities (Mayo Clinic, 2024). In adults aged 65 years and above, the prevalence of MCI is estimated at 10%–20%, with approximately 32% progressing to dementia over a mean follow-up of 4.57 years (Voinescu et al., 2021). Despite its clinical significance, MCI is often under-recognized, posing challenges for early diagnosis and timely intervention. This is particularly concerning given its potential to advance into more severe forms of cognitive impairment (Mian et al., 2024). Currently, treatment options for MCI are limited, and strategies to delay its progression to dementia remain inadequate (Ornish et al., 2024; Voinescu et al., 2021). As global life expectancy rise, the population living with cognitive impairment is projected to grow, despite a decline in age-specific incidence rates in high-income regions (Livingston et al., 2024). This trend underscores the urgent necessity to identify and deploy effective preventive strategies to mitigate the growing burden of cognitive impairment.

Exercise is pivotal for slowing cognitive decline and supporting brain health. It enhances neuroplasticity, reduces inflammatory processes, improves vascular function, and modulates neurotransmitter systems (Guilherme et al., 2022; Mian et al., 2024). Evidence indicates that exercise activity at any age can benefit cognition, potentially through mechanisms such as increased cerebral perfusion, reductions in hypertension, and greater nitric oxide production, and attenuation of neuroinflammation—mechanisms that collectively foster brain plasticity. Individuals who engage more frequently in moderate-to-vigorous physical activity (PA) typically show larger brain volumes than their sedentary peers (Livingston et al., 2024). As a non-pharmacological strategy, exercise is increasingly recognized for helping prevent cognitive deterioration and for improving quality of life (QoL) in people with cognitive impairment (Huang et al., 2022; Tao et al., 2023a; Tao et al., 2023c). The choice of exercise modality is nevertheless crucial when prescribing programs for cognitive health (Huang et al., 2022). Prior research indicates that different exercise modalities act through distinct physiological and molecular pathways, yielding variable cognitive and functional gains (Akalp et al., 2024; Li et al., 2024).

As a form of exercise activity, dance has emerged as an innovative, multidimensional intervention with demonstrated cognitive and psychosocial benefits (Tao et al., 2022; Tao et al., 2024; Tao et al., 2021a; Tao et al., 2021b). It typically consists of choreographed movements performed to music, delivering a rich, multisensory experience that combines auditory, visual, and kinesthetic stimulations (Marquez et al., 2017; Guzman et al., 2021; Jaldin et al., 2024; Lopez et al., 2024). Early epidemiological evidence (2003) reported dance as the only leisure activity linked to a lower risk of dementia (Verghese et al., 2003). Subsequent randomized controlled trials (RCTs) and meta-analyses indicate that dance interventions can enhance cognitive performance, physical and mental health, and activity of daily living (ADLs) in individuals with cognitive impairment. Neuroimaging studies further suggest that dance-based exercise may promote neural plasticity, providing biological plausibility for its therapeutic effects (Chan et al., 2020; Huang et al., 2023; Wu et al., 2021). By integrating physical, cognitive, emotional, and social elements, dance appears particularly suitable for Alzheimer’s disease (AD)—the most common cause of dementia—and other dementias; its relatively modest space and equipment requirements further facilitate implementation (Ruiz-Muelle and López-Rodríguez, 2019; Tao et al., 2022).

Network meta-analysis (NMA) enables the simultaneous comparison of multiple interventions within a single analytic framework by integrating both direct and indirect evidence and provides rankings of interventions by estimated effectiveness (Seide et al., 2020). Most prior NMAs have compared several exercise modalities but typically evaluated only one or two cognition- or physiology-related outcomes (Huang et al., 2022; Li et al., 2025; Liu et al., 2023; Lv et al., 2023; Wang et al., 2019; Zeng et al., 2025; Zhao et al., 2022). Although several NMA have included dance and compared its efficacy with other exercise modalities, evidence for a comparative advantage of dance in preventing or slowing cognitive decline and improving overall wellbeing remains limited. Much of the evidence still comes from trials that compare dance with usual care or inactive controls rather than active exercise comparators. Nevertheless, clinicians, patients, and policymakers need rigorous comparative evidence to guide exercise selection for cognitive health (Ahn and Kang, 2021). To address this gap, and given the growing promise of dance, the aim of this study was to conduct a comprehensive systematic review and NMA of RCTs to compare the efficacy of dance with other exercise interventions on cognitive function, physical performance, and psychological wellbeing in individuals with MCI and dementia, and to generate clinically relevant rankings and recommendations to inform evidence-based exercise prescription.

## 2 Methodology

### 2.1 Protocol and registration

The systematic review and NMA followed the Preferred Reporting Items for Systematic Reviews and Meta-Analysis incorporating Network Meta-analyses (PRISMA NMA) guidelines (PRISMA, 2020). The PRISMA Extension Statement for Reporting Systematic Reviews Incorporating Network Meta-analyses of Healthcare Interventions was also adhered to, which provides comprehensive guidance for conducting and reporting NMA in healthcare research (Hutton et al., 2015). The protocol was prospectively registered with PROSPERO (CRD 42024549996) on 5 May 2024.

### 2.2 Eligibility criteria

#### 2.2.1 Inclusion criteria

Eligibility was determined using the PICOS framework (population, intervention, comparison, outcome, and study design) (Methley et al., 2014). Studies were included if they met the following criteria: (1) Population: adults diagnosed with MCI or dementia (any stage or etiology) by a specialist or via validated instruments, including the Mini-Mental State Examination (MMSE) (Jia et al., 2021), Montreal Cognitive Assessment (MoCA) (Nasreddine et al., 2005), Clinical Dementia Rating (CDR) (Morris, 1993), National Institute on Aging and Alzheimer’s Association (NIA-AA) guidelines (Hyman et al., 2012), Diagnostic and Statistical Manual of Mental (DSM) (Wakefield, 2016) or the Neurological and Communicative Disorders and Stroke-Alzheimer’s Disease and Related Disorders Association (NINCDS-ADRDA) (McKhann et al., 1984). Studies were also eligible if participants were identified as cognitively impaired by the study’s authors or were residents of neuropsychiatric care facilities. Participants needed to be physically able to exercise (e.g., not wheelchair-bound) and free of major comorbidities (e.g., significant cardiovascular disease, cancer, or chronic respiratory illness). (2) Intervention: dance-based or other structured exercise programs with a minimum duration of 3 weeks, with sufficient procedural detail to permit replication. (3) Comparison: usual care or an alternative exercise intervention. (4) Outcomes: quantitative measures of neurological, physiological, or psychological health. (5) Study design: RCTs only. When multiple reports described on the same trial, data were extracted from all relevant publications and consolidated as a single study to prevent duplication.

#### 2.2.2 Exclusion criteria

Studies were excluded if any of the following applied: (1) the primarily focus was physiotherapy, cognitive training, or other non-exercise interventions; (2) only acute interventions were evaluated; (3) the article was not published in English or lack peer review; (4) the report type was a review, dissertation, commentary, or conference abstract; or (5) the full text was unavailable and authors could not be contacted for clarification.

### 2.3 Information sources and search

An initial search in PubMed validated the research question and confirmed the novelty of this review; no prior NMA comparing the dance-based with other exercise interventions for cognitive impairment was identified. A comprehensive search was then independently performed by two reviewers (DT and YZ) across five databases (PubMed, Web of Science, MEDLINE, Embase, and PsycINFO) on 1^st^ May 2025, capturing all available records. Database-specific search strategies incorporated MeSH terms and keywords variants (Supplementary Table S1). The reference lists of eligible publications and pertinent reviews published within the previous 2 years were screened manually. Study authors were contacted to obtain missing data; when no response was received, the study was retained in the systematic review but excluded from the pairwise meta-analysis and NMA to preserve data integrity.

### 2.4 Study selection

Search results were managed in EndNote (version 21.5). After removing duplicates, two reviewers (DT and YZ) independently screened titles and abstracts, then conducted full-text assessments for records deemed potentially eligible. Disagreements were settled through discussion, when necessary, by consulting a third author (BZ).

### 2.5 Data collection process and data items

A standardized data extraction form was developed and pilot-tested in accordance with the Cochrane Handbook (Julian et al., 2024). Two reviewers (DT and YZ) refined the procedures during piloting. After finalizing, two reviewers (DT and YZ) independently extracted data and iteratively updated the forms as needed. Extracted variables included: (1) study identification (author, year, and study design); (2) sample characteristics (cognitive status, sample size, age, sex, and education); (3) intervention details (description, intensity, frequency, and adherence; see Supplementary Table S2, S42) outcomes with mean ± standard deviation (SD) values used to compute standardized mean differences (SMDs) for global cognition and specific cognitive domains (Mini-Mental State Examination [MMSE]; Montreal Cognitive Assessment [MoCA], Trail Making Test-A [TMT-A], Trail Making Test-B [TMT-B], Digit Span Forward [DSF], Digit Span Backward [DSB]); psychological status (Geriatric Depression Scale [GDS]), physical performance (Timed Up and Go [TUG], Berg Balance Scale [BBS], Short Physical Performance Battery [SPPB], gait speed), and QoL (Quality of Life in Alzheimer’s Disease [QoL-AD]; see Supplementary Table S3, S5), intervention protocols (Supplementary Table S4, S6), measurement instruments (Supplementary Table S5, S7) study limitations and future research recommendations (Supplementary Table S6).

Exercise interventions were categorized as follows: (1) dance (e.g., dance movement therapy [DMT], ballroom, aerobic, traditional, and improvisational dance); (2) exergaming; (3) yoga; (4) Chinese traditional exercise (CTE); (5) aerobic exercise (AE); (6) resistance exercise (RE); (7) multicomponent exercise (ME; combining ≥2 modalities). Cognitive impairment was classified as: (1) normal: MMSE 27–30 or MoCA 26–30; (2) MCI: MMSE 21–26 or MoCA 18–25; (3) moderate: MMSE 10–20 or MoCA 10–17; (4) severe: MMSE 0–9 or MoCA 0–9 (McCarthy et al., 2024; Nasreddine et al., 2005).

### 2.6 Risk of bias within individual studies

The methodological rigor of the included RCTs was appraised with the Cochrane Risk of Bias Tool for Randomized Trials (RoB 2) (Higgins and Altman, 2008) in RevMan (version 5.4.1). Overall judgments were categorized as low, high, and unclear risk of bias. Given the nature of the interventions, participant blinding was not feasible; therefore, the assessment focused on six domains: random sequence generation, allocation concealment, blinding of outcome assessment, incomplete outcome data, selective reporting, and other bias (specifically gender imbalance). Attrition rate—calculated as the proportion of participants completing post-test assessments—was used to appraise “incomplete outcome data”. Attrition bias was rated high if (1) attrition exceeding 20% in any group or (2) between-group differences in attrition exceeded 10% without appropriate handling (e.g., intention-to-treat analysis). For the “other bias” domain, studies in which one gender constituted ≥60% of the sample were classified as high risk. Two authors (DT and YZ) conducted the assessments independently; disagreements were settled through discussion, when required, by consulting a third author (BZ).

### 2.7 Statistical analysis

Pairwise meta-analyses were performed in R (version 4.4.3) using random-effects models. Effect sizes were expressed as SMDs with 95% confidence intervals (Cis) and interpreted as small (<0.4), moderate (0.4–0.7), or large (>0.7) (Julian et al., 2024). Between-study heterogeneity was evaluated with the Chi-square test and the *I* ^*2*^ statistic, using the following benchmarks: 0%–40% (may be unimportant); 30%–60% (may indicate moderate heterogeneity); 50%–90% (may indicate substantial heterogeneity); and 75%–100% (considerable heterogeneity) (Higgins et al., 2023). When heterogeneity was low, fixed-effect models were examined in sensitive analyses. Pairwise syntheses were conducted only for outcomes reported by at least two studies. Results are reported as pooled SMDs with 95% Cis, *p*-values, and heterogeneity estimates.

The NMA was conducted with brms and related packages within a Bayesian random-effects framework in R (version 4.4.3). Network plots displayed interventions as nodes, with edge thickness proportional to the number of direct comparisons and node size proportional to the number of contributing studies (Shim and Lee, 2019). The NMA combined direct evidence from head-to-head trials with indirect evidence through a shared comparator (Ahn and Kang, 2021). For multi-arms studies, arms involving non-exercise interventions were excluded from the network. Relative rankings were summarized from the posterior distributions using the Surface Under the Cumulative Ranking Curve (SUCRA), where 100% denotes the highest rank (most effective) and 0% the lowest (Huang et al., 2022). *P*-scores pertain to frequentist NMAs; they were not calculated. A league table was produced to display all pairwise contrasts among interventions, reporting SMDs with 95% Cis; statistically significant differences were highlighted to indicate clinical relevance.

To evaluate the robustness of the Bayesian NMA, both local and global inconsistency were examined. Local inconsistency was assessed using a node-splitting approach, comparing direct with indirect evidence for specific intervention comparisons informed by both sources. If the 95% credible interval (CrI) for the inconsistency factor included the null value (0) and the corresponding *p*-value was ≥0.05, the comparison was considered consistent. Global inconsistency was explored using a design-by-treatment interaction approach within a complementary frequentist framework, given methodological constraints on implementing a full global inconsistency test within the Bayesian model. When inconsistency was detected, a Bayesian random-effects inconsistency model was fitted as a sensitivity analysis. Subgroup and meta-regression analyses were not undertaken due to limited data availability.

## 3 Results

### 3.1 Study selection

**FIGURE 1:**
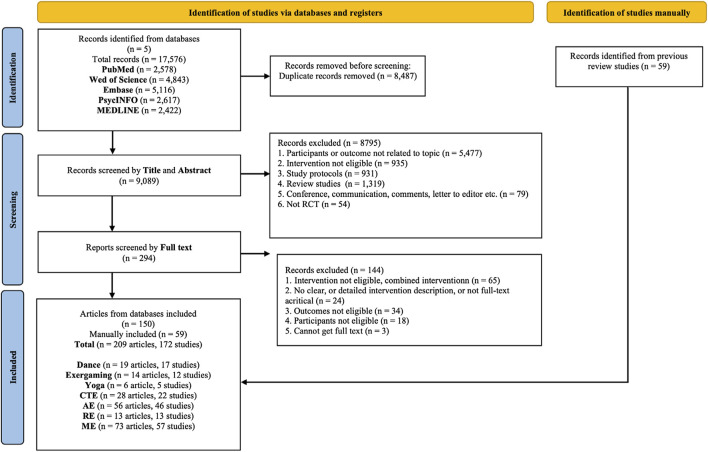
PRISMA flow diagram of screening and selection process.

The initial database search yielded 17,576 records. After removing 8,487 duplicates, we screened 9,089 titles and abstracts. Of these, 294 articles underwent full-text review, and an additional 59 were identified through manual reference screening. Ultimately, 209 articles (corresponding to 172 unique studies) published up to 1^st^ May 2025 were included. These studies compared seven interventions with control groups or with one another. Reasons for exclusion are presented in the PRISMA flow diagram (Figure 1). To obtain missing data, authors of 47 studies were contacted and received usable data from ten. Across all included studies, there were 13,773 participants: 8,322 females, 5,027 males, and 424 with gender not reported. In total, 106 studies were eligible for pairwise meta-analysis and 112 for the NMA.

### 3.2 Study characteristics

Detailed characteristics of the 209 RCTs are summarized in Supplementary Table S2. Studies were categorized into seven intervention groups: dance (n = 19), exergaming (n = 14), yoga (n = 6), CTE (n = 28), AE (n = 56), RE (n = 13), and ME (n = 73).

#### 3.2.1 Study design and publication trends

**FIGURE 2:**
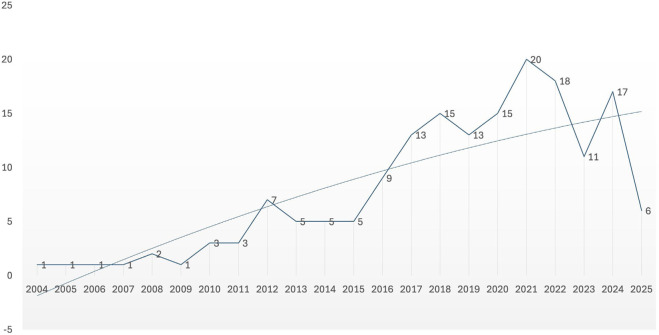
Publication yearly trend.

Publication years ranged from 2004 to 2025. Between 2004 and 2011, publication rates were low, with only one to three studies per year. A gradual increase began in 2012, peaking at 20 studies in 2021. A modest decline followed in 2022 (n = 18) and 2023 (n = 11), with a rebounded in 2024 (n = 17). Because the search was conducted early 2025, the apparent decline that year (n = 6) was not interpreted. This trend suggests growing scientific interest in exercise-based cognitive interventions (Figure 2).

#### 3.2.2 Diagnostic instruments utilized

The MMSE was the most used cognitive diagnostic tool across several interventions, including dance (47%), exergaming (33%), CTE (41%), AE (52%), RE (62%), and ME (47%). Yoga studies employed a broader range of tools, with the MMSE, MoCA, CDR, and NIA-AA each used in 20% of studies (Supplementary Table S7). A comprehensive overview of all outcome measurement instruments used across the included studies is available in Supplementary Table S5.

#### 3.2.3 Cognitive status

Baseline cognitive statis varied across intervention types according to MMSE and MoCA classification (Supplementary Table S7). In dance studies, 47% of participants were MCI by MoCA; 35% cognitively normal, 12% MCI, and 12% had moderate to severe impairment by MMSE. Exergaming studies reported 17% cognitively normal, 33% MCI, and 42% moderate impairment by MMSE; plus 25% MCI and 8% severe impairment by MoCA. Yoga studies showed 40% MCI by MoCA; 20% MCI and 20% cognitively normal by MMSE. For CTE studies, 41% were MCI by MoCA and 36% by MMSE, with moderate impairment in 9% by MoCA and 18% by MMSE, and 9% cognitively normal by MoCA. AE studies reported 30% MCI by MoCA; 17% cognitively normal, 22% MCI, 24% moderate, and 2% severe impairment by MMSE. In RE studies, 54% were MCI by MMSE and 31% by MoCA, with 8% moderate impairment by each tool, and 15% cognitively normal by MMSE. ME studies showed 1.7% cognitively normal, 44% MCI, 38.5% moderate, and 1.7% severe impairment by MMSE; and 14% MCI and 5% moderate impairment by MoCA.

#### 3.2.4 Age distribution

**FIGURE 3:**
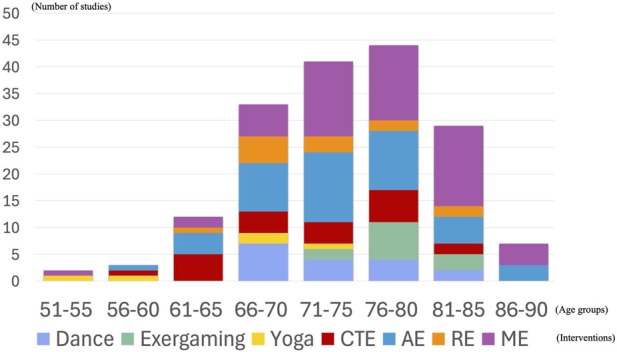
Participant age distribution.

Participants in age distributions varied across intervention types. In dance studies, the most common age range was 66–70 years (n = 7). Exergaming studies primarily included participants aged 76–80 years (n = 7). Yoga interventions tended to include young participants, with a skew toward the 51–75 years range. CTE studies exhibited a broader age distribution, primarily between 61 and 80 years. In AE studies, the most common age range was 71–75 years (n = 13). RE studies primarily involved participants aged 66–70 years (n = 5). ME studies included the highest number of participants in the 71–85 years (n = 43) (Figure 3).

#### 3.2.5 Distribution of sample sizes

Sample sizes distributions varied across intervention groups, reflecting differences in study design and recruitment strategies (Supplementary Table S7). For dance interventions, the most common ranges were 21–25 and 201–205 (each 12%), followed by 31–35 (18%). In exergaming studies, the range of 21–25 was most frequent (25%), followed by 46–50 and 111–115 (17% each). Yoga studies predominantly fell within the 76–80 range (60%). For CTE studies, the most common sample size range was 66–70 (14%). Sample sizes in AE studies were widely distributed, with 21–25 being the most frequent (11%). In RE studies, the 26–30 range was most common (31%), followed by 41–45 (23%). ME studies tended to have larger sample sizes (491–495), with the most common ranges (26–30, 31–35, and 46–50) each accounting for 8.8%.

#### 3.2.6 Intervention characteristics

Twelve-week interventions were most commonly implemented across several interventions: dance (59%), exergaming (33%), yoga (80%), CTE (36%), AE (24%), RE (61.5%), and ME (40%). Session duration was consistently reported as 60-min across all intervention types. The most common session frequency was three times per week for most interventions. The exceptions were dance, for which twice-weekly sessions were most common (47%), and yoga, which most frequently used a once-weekly schedule (40%).

Monitoring practices varied across intervention types. Among dance, 23% of studies employed heart rate (HR) monitor, 12% used Rating of Perceived Exertion (RPE), and 65% reported no monitoring. In exergaming, 25% used HR monitor, 17% used RPE, and 67% used no monitoring. No monitoring was reported in yoga studies. For CTE, one study used RPE, while 95% reported no monitoring. Among AE, 46% used HR monitor, 33% used RPE, and 35% used no monitoring. In RE, 15% used HR monitor, 31% used RPE, and 54% used no monitoring. For ME, 26% used HR monitor, 14% used RPE, and 65% reported no monitoring.

Reporting of exercise intensity was inconsistent across intervention types. Among dance, 41% of studies ranged from light to vigorous, using metrics such as 60%–80% HRmax, VO_2_max, or RPE (between 2/10 and 14/20). In exergaming, 42% of studies reported intensity ranging from 50% to 75% HRmax, RPE 5/10–14/20, or 65%–75% of Heart Rate Reserve (HRR). Intensity was largely unreported in yoga and CTE, with one CTE study reporting an RPE of 13/20. Among AE, 74% of studies used 60%–90% HRmax, 50%–85% HRR, 4/10–17/20 RPE, VO_2_max, or 1 Repetition maximum (RM). In RE, 69% of studies reported intensities ranged from 53% to 85% HRmax or 1RM, with RPE values from 12 to 16/20. For ME, 40% of studies used 40%–85% HRmax, 35%–85% HRR, 4/10–16/20 RPE, or 40%–85% 1RM (Supplementary Table S7).

#### 3.2.7 Adherence rates

Adherence was assessed at the end of each intervention period (Supplementary Table S7). In dance trials, the most commonly reported adherence was 91%–95% in both groups, observed in 23.5% of studies. Exergaming most often showed 96%–100% adherence in both intervention and control groups (25%). In yoga studies, adherence was most commonly 76%–80% in both groups (40%). For CTE studies, the most frequent adherence in intervention groups was 86%–90% (41%). In AE studies, the most common adherence was 86%–90% and 96%–100% (22%) observed in control groups. RE studies most often reported 86%–90% or 96%–100% in intervention groups (23%) and 96%–100% in control groups (33%). In ME studies, the most common adherence was 96%–100% (30%) in control groups.

### 3.3 Risk of bias within studies

The distribution of studies across risk of bias domains is summarized in Supplementary Table S8. For “random sequence generation”, 56.4% of studies were rated as low risk, 43.6% as unclear risk, and none as high risk. “Allocation concealment” was rated as low risk in 18.6% of studies, high risk in 1.2% of studies, and unclear in 80.2% of studies. Regarding the “blinding of outcome assessors”, 60.5% of studies were rated as low risk, 4% as high risk, and 35.5% as unclear. All studies were rated as low risk for “selective reporting” and demonstrated high-quality reporting, with all protocol-specific outcomes fully detailed in the results. For “other bias” (particularly gender imbalance), 25.6% of studies were considered low risk, 69.7% high risk, and 4.7% unclear. For “incomplete outcome data”, 57.6% of studies were assessed as low risk, 30.2% as high risk, and 12.2% as unclear.

Participant dropout was attributed to various factors: limited physical capacity, fatigue, or generalized weakness (Chan et al., 2016; Nakatsuka et al., 2015); confirmed COVID-19 cases or isolation requirements (Huang et al., 2025; Kušleikienė et al., 2025; Levinger et al., 2023; Shokri et al., 2023; Vints et al., 2024); medical issues such as malaise, uncontrolled blood pressure, depressive episode, ankle sprain, or general health issues (Bisbe et al., 2020; Brett et al., 2021; Cancela et al., 2016; Cezar et al., 2021; Chan et al., 2016; David et al., 2025; Esmail et al., 2020; Fischbacher et al., 2020; Hsu et al., 2022; Kušleikienė et al., 2025; Lam et al., 2012; Singh et al., 2014; Song and Yu, 2019; Thiel et al., 2024; Venturelli et al., 2010; Verdelho et al., 2024; Yan et al., 2024); hospitalization (Brett et al., 2021; Chan et al., 2016; Li et al., 2021; Venturelli et al., 2010; Yan et al., 2024); participants mortality (Baker et al., 2025; Brett et al., 2021; Cancela et al., 2016; Lam et al., 2012; Mak et al., 2022; Venturelli et al., 2010); personal reasons such as lacked time, family or personal commitments or events (Bisbe et al., 2020; Cezar et al., 2021; Esmail et al., 2020; Fischbacher et al., 2020; Hong et al., 2018; Huang et al., 2025; Singh et al., 2014; Song and Yu, 2019; Verdelho et al., 2024; Yan et al., 2024; Yu et al., 2022; Zhang et al., 2023; Zheng et al., 2022); relocation (Bisbe et al., 2020; Cancela et al., 2016; Hsu et al., 2022; Li et al., 2021; Mak et al., 2022; Verdelho et al., 2024); extreme weather conditions or travelling issues (Huang et al., 2025; Levinger et al., 2023); insufficient caregiver support (Levinger et al., 2023); unmet expectations regarding the intervention (Esmail et al., 2020; Singh et al., 2014); lacked motivation or interest (Baker et al., 2025; Cancela et al., 2016; David et al., 2025; Fischbacher et al., 2020; Hsu et al., 2022; Huang et al., 2025; Kashyap et al., 2022; Kim et al., 2016; Kušleikienė et al., 2025; Thiel et al., 2024); loss of contact (Chan et al., 2016; Hsu et al., 2022; Verdelho et al., 2024); forgetfulness (Chan et al., 2016; Fischbacher et al., 2020); discontinued intervention (Verdelho et al., 2024; Baker et al., 2025); refusal to undergo assessment (Mak et al., 2022; Shokri et al., 2023; Kušleikienė et al., 2025); or unspecified reasons (Zheng et al., 2022). Despite these dropouts, attrition rates remain within acceptable limits and are often unavoidable in this population due to health fluctuations and caregiving demands.

### 3.4 Results of pairwise meta-analysis

A total of 106 studies involving 8,214 participants were included in the pairwise meta-analysis. Direct comparisons were made between the seven interventions and control groups across 12 outcome measures with only statistically significant results are reported in Table 1. From the perspective of dance, the meta-analysis revealed that dance significantly improved global cognition (MMSE and MoCA), reduced depressive symptoms (GDS), and improving balance (BBS). Executive function (TMT-B) improved most with RE and ME, whereas short-term verbal memory/attention (DSF) was most effectively enhanced by CTE. For physical performance and QoL, improvements such as mobility and balance gains (TUG, SPPB, and gait speed) were observed with AE, RE, ME, and exergaming. Despite these promising effects, substantial heterogeneity was observed in some outcomes (e.g., MoCA with CTE, *I* ^*2*^ = 88.5%), likely reflecting variability in study designs, intervention protocols, participants and characteristics.

**TABLE 1:** The results of the pairwise meta-analysis.

Outcome measure	Interventions (no. of studies)	Effect size (SMD) 95% CI, *p*-value, *I* ^*2*^	Interpretation
MMSE	Dance n = 4	SMD = 0.6766; 95% CI: [0.3999, 0.9533]; *p* = 0.0001; *I* ^*2*^ = 42.0%	Moderate improvement in global cognition
	ME n = 18	SMD = 0.6043; 95% CI: [0.1604, 1.0482]; *p* = 0.0106; *I* ^*2*^ = 85.6%	Moderate cognitive benefit
MoCA	Dance n = 7	SMD = 0.6853; 95% CI: 0.2956, 1.0751]; *p* = 0.0051; *I* ^*2*^ = 69.3%	Moderate improvement in executive/visuospatial function
	Yoga n = 2	SMD = 0.6873; 95% CI: [0.3580, 1.0167]; *p* = 0.0001; *I* ^*2*^ = 0.0%	Moderate benefit with low heterogeneity
	CTE n = 8	SMD = 1.4506; 95% CI: [0.2916, 2.6097]; *p* = 0.0211; *I* ^*2*^ = 88.5%	Large cognitive effect, but high variability
	AE n = 12	SMD = 0.7885; 95% CI: [0.3879, 1.1891]; *p* = 0.0012; *I* ^*2*^ = 75.2%	Moderate-to-large cognitive gains
	RE n = 5	SMD = 0.7799; 95% CI: [0.0049, 1.5549]; *p* = 0.0491; *I* ^*2*^ = 50.2%	Potential benefit; wide CI and high heterogeneity
	ME n = 9	SMD = 1.0246; 95% CI: [0.2435, 1.8057]; *p* = 0.0164; *I* ^*2*^ = 88.2%	Large cognitive effect; high heterogeneity
TMT-B	RE n = 4	SMD = −0.5608; 95% CI: [-0.8829, −0.2387]; *p* = 0.0006; *I* ^*2*^ = 40.4%	Moderate effect in executive function
	ME n = 6	SMD = −0.2343; 95% CI: [-0.4441, −0.0245]; *p* = 0.0286; *I* ^*2*^ = 54.8%	Small improvement in executive function
DSF	CTE n = 3	SMD = 0.2979; 95% CI: [0.1105, 0.4852]; *p* = 0.0018; *I* ^*2*^ = 46.6%	Small-to-moderate benefit in attention span/short-term verbal memory
GDS	Dance n = 2	SMD = −0.3691; 95% CI: [-0.6758, −0.0624]; *p* = 0.0183; *I* ^*2*^ = 24.8%	Reduced depressive symptoms
TUG	AE n = 10	SMD = −0.5752; 95% CI: [-0.9547, −0.1956]; *p* = 0.0075; *I* ^*2*^ = 53.2%	Moderate improvement in mobility
	RE n = 6	SMD = −0.6134; 95% CI: [-1.0769, −0.1498]; *p* = 0.0192; *I* ^*2*^ = 21.8%	Moderate improvement in mobility
	ME n = 14	SMD = −0.3970; 95% CI: [-0.6104, −0.1835]; *p* = 0.0015; *I* ^*2*^ = 49.4%	Small-to-moderate mobility improvement
BBS	Dance n = 3	SMD = 0.4090; 95% CI: [0.0983, 0.7198]; *p* = 0.0298; *I* ^*2*^ = 0.0%	Improved balance with low heterogeneity
SPPB	ME n = 4	SMD = 0.7343; 95% CI: [0.2471, 1.2216]; *p* = 0.0172; *I* ^*2*^ = 56.5%	Moderate-to-large improvement in physical performance
Gait speed	ME n = 2	SMD = 0.4431; 95% CI: [0.3163, 0.5699]; *p* = 0.0143; *I* ^*2*^ = 0.0%	Moderate increase in gait speed
QoL-AD	Exergaming N = 2	SMD = 0.5868; 95% CI: [0.0624, 1.1112]; *p =* 0.0283; *I* ^*2*^ = 59.4%	Moderate improvement in quality of life

### 3.5 Results of network meta-analysis

**FIGURE 4:**
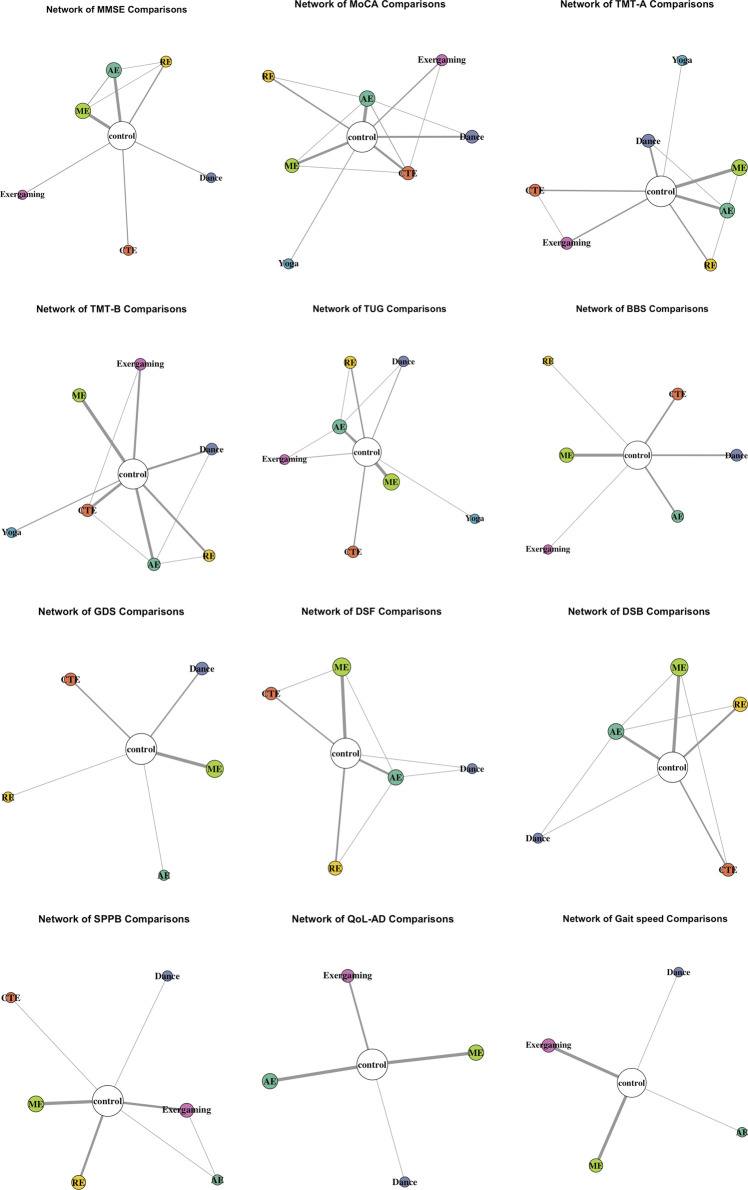
Network comparison plots.

**FIGURE 5:**
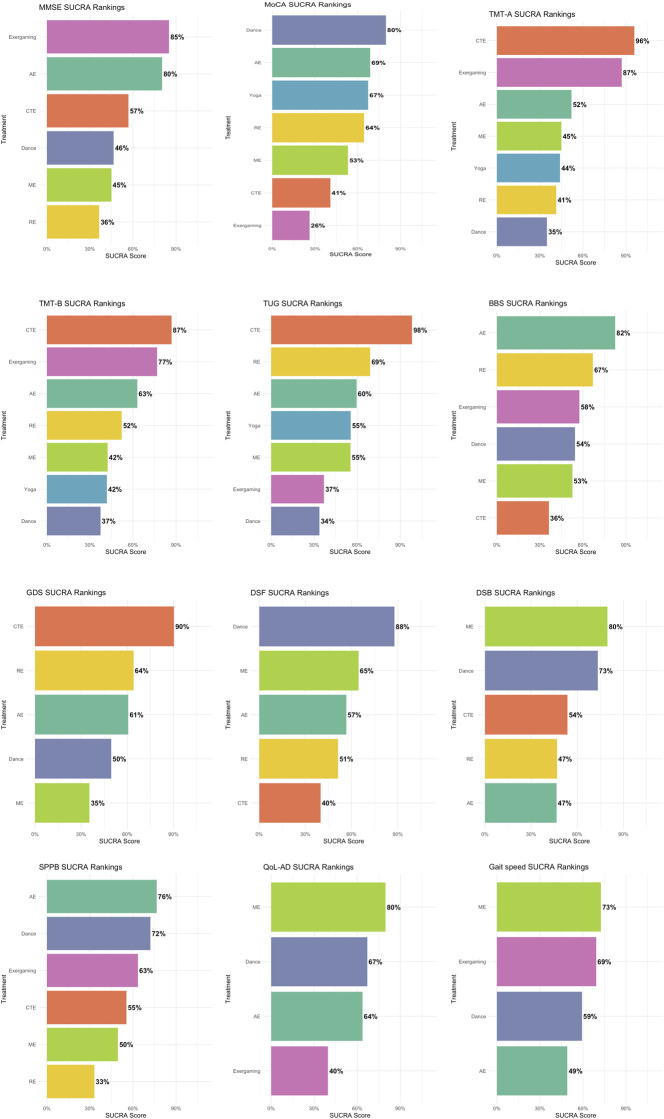
SUCRA ranking of interventions across outcomes.

A total of 112 studies comprising 8,250 participants were included in the NMA. Given the substantial heterogeneity identified in the pairwise meta-analysis, a Bayesian framework with a random-effects model was employed (see Figures 4, 5; Table 2; inconsistency test results are presented in Supplementary Table S9).

**TABLE 2:** NMA league tables for all interventions across outcomes.

**MMSE**						
	AE	CTE	Dance	Exergaming	ME	RE
AE	—	−0.20 [−0.96, 0.57]	−0.39 [−1.25, 0.49]	0.28 [−0.69, 1.28]	−0.37 [−0.88, 0.15]	−0.55 [−1.27, 0.15]
CTE	0.20 [−0.57, 0.96]	—	−0.19 [−1.24, 0.84]	0.48 [−0.68, 1.65]	−0.17 [−0.94, 0.60]	−0.36 [−1.29, 0.56]
Dance	0.39 [−0.49, 1.25]	0.19 [−0.84, 1.24]	—	0.68 [−0.56, 1.88]	0.02 [−0.88, 0.90]	−0.17 [−1.19, 0.84]
Exergaming	−0.28 [−1.28, 0.69]	−0.48 [−1.65, 0.68]	−0.68 [−1.88, 0.56]	—	−0.66 [−1.66, 0.35]	−0.84 [−1.96, 0.23]
ME	0.37 [−0.15, 0.88]	0.17 [−0.60, 0.94]	−0.02 [−0.90, 0.88]	0.66 [−0.35, 1.66]	—	−0.18 [−0.91, 0.52]
RE	0.55 [−0.15, 1.27]	0.36 [−0.56, 1.29]	0.17 [−0.84, 1.19]	0.84 [−0.23, 1.96]	0.18 [−0.52, 0.91]	—

**MoCA**							
	AE	CTE	Dance	Exergaming	ME	RE	Yoga
AE	—	−0.38 [−1.14, 0.37]	0.18 [−0.64, 0.99]	−0.72 [−1.75, 0.26]	−0.21 [−0.99, 0.57]	−0.03 [−1.07, 0.97]	0.04 [−1.33, 1.45]
CTE	0.38 [−0.37, 1.14]	—	0.56 [−0.28, 1.42]	−0.34 [−1.37, 0.66]	0.17 [−0.64, 0.99]	0.34 [−0.69, 1.42]	0.42 [−0.94, 1.84]
Dance	−0.18 [−0.99, 0.64]	−0.56 [−1.42, 0.28]	—	−0.90 [−2.01, 0.16]	−0.39 [−1.28, 0.49]	−0.22 [−1.32, 0.84]	−0.13 [−1.54, 1.31]
Exergaming	0.72 [−0.26, 1.75]	0.34 [−0.66, 1.37]	0.90 [−0.16, 2.01]	—	0.52 [−0.53, 1.57]	0.69 [−0.54, 1.93]	0.77 [−0.76, 2.36]
ME	0.21 [−0.57, 0.99]	−0.17 [−0.99, 0.64]	0.39 [−0.49, 1.28]	−0.52 [−1.57, 0.53]	—	0.17 [−0.91, 1.24]	0.25 [−1.11, 1.67]
RE	0.03 [−0.97, 1.07]	−0.34 [−1.42, 0.69]	0.22 [−0.84, 1.32]	−0.69 [−1.93, 0.54]	−0.17 [−1.24, 0.91]	—	0.08 [−1.46, 1.69]
Yoga	−0.04 [−1.45, 1.33]	−0.42 [−1.84, 0.94]	0.13 [−1.31, 1.54]	−0.77 [−2.36, 0.76]	−0.25 [−1.68, 1.11]	−0.08 [−1.69, 1.46]	—

**TMT**−**A**							
	AE	CTE	Dance	Exergaming	ME	RE	Yoga
AE	—	**2.23 [0.51, 3.99]	−0.48 [−1.95, 0.95]	1.56 [−0.01, 3.26]	−0.19 [−1.46, 1.07]	−0.31 [−2.01, 1.38]	−0.29 [−2.91, 2.26]
CTE	**−**2.23 [**−**3.99,** −**0.51]**	—	**−**2.71 [**−**4.61,** −**0.90]**	−0.66 [−2.58, 1.36]	**−**2.42 [**−**4.17,** −**0.74]**	**−**2.55 [**−**4.64,** −**0.55]**	−2.51 [−5.44, 0.21]
Dance	0.48 [−0.95, 1.95]	**2.71 [0.90, 4.61]	—	**2.04 [0.36, 3.88]	0.30 [−1.14, 1.72]	0.18 [−1.65, 2.03]	0.22 [−2.49, 2.84]
Exergaming	−1.56 [−3.26, 0.01]	0.66 [−1.36, 2.58]	**−**2.04 [**−**3.89,** −**0.36]**	—	**−**1.76 [**−**3.44,** −**0.20]**	−1.87 [−3.88, 0.01]	−1.83 [−4.68, 0.83]
ME	0.19 [−1.07, 1.46]	**2.42 [0.74, 4.17]	−0.30 [−1.72, 1.14]	**1.76 [0.20, 3.44]	—	−0.11 [−1.78, 1.58]	−0.08 [−2.73, 2.44]
RE	0.31 [−1.38, 2.01]	**2.55 [0.55, 4.64]	−0.18 [−2.03, 1.65]	1.87 [−0.01, 3.88]	0.11 [−1.58, 1.78]	—	0.03 [−2.77, 2.83]
Yoga	0.29 [−2.26, 2.91]	2.51 [−0.21, 5.44]	−0.22 [−2.84, 2.49]	1.83 [−0.83, 4.68]	0.08 [−2.44, 2.73]	−0.03 [−2.83, 2.77]	—

**TMT**−**B**							
	AE	CTE	Dance	Exergaming	ME	RE	Yoga
AE	—	0.67 [−0.84, 2.23]	−0.65 [−2.31, 0.95]	0.38 [−1.22, 2.09]	−0.50 [−2.03, 1.07]	−0.26 [−2.01, 1.45]	−0.61 [−2.76, 1.55]
CTE	−0.67 [−2.23, 0.84]	—	−1.32 [−2.98, 0.28]	−0.29 [−1.90, 1.38]	−1.16 [−2.76, 0.38]	−0.93 [−2.71, 0.72]	−1.28 [−3.50, 0.87]
Dance	0.65 [−0.95, 2.31]	1.32 [−0.28, 2.98]	—	1.02 [−0.60, 2.79]	0.14 [−1.48, 1.76]	0.39 [−1.40, 2.13]	0.03 [−2.19, 2.28]
Exergaming	−0.38 [−2.09, 1.22]	0.29 [−1.38, 1.90]	−1.02 [−2.79, 0.60]	—	−0.89 [−2.57, 0.74]	−0.64 [−2.50, 1.09]	−1.00 [−3.26, 1.20]
ME	0.50 [−1.07, 2.03]	1.16 [−0.38, 2.76]	−0.14 [−1.76, 1.48]	0.89 [−0.74, 2.57]	—	0.24 [−1.48, 1.95]	−0.11 [−2.30, 2.05]
RE	0.26 [−1.45, 2.01]	0.93 [−0.72, 2.71]	−0.39 [−2.13, 1.40]	0.64 [−1.09, 2.50]	−0.24 [−1.95, 1.48]	—	−0.34 [−2.64, 1.95]
Yoga	0.61 [−1.55, 2.76]	1.28 [−0.87, 3.50]	−0.03 [−2.28, 2.19]	1.00 [−1.20, 3.26]	0.11 [−2.05, 2.30]	0.34 [−1.96, 2.64]	—

**TUG**							
	AE	CTE	Dance	Exergaming	ME	RE	Yoga
AE	—	**0.78 [0.19, 1.43]	−0.32 [−1.02, 0.39]	−0.27 [−0.99, 0.42]	−0.12 [−0.56, 0.33]	0.13 [−0.48, 0.74]	−0.02 [−1.11, 1.04]
CTE	**−**0.78 [**−**1.43,** −**0.19]**	—	**−**1.10 [**−**1.93,** −**0.31]**	**−**1.05 [**−**1.91,** −**0.27]**	**−**0.89 [**−**1.53,** −**0.33]**	−0.65 [−1.40, 0.05]	−0.80 [−1.97, 0.32]
Dance	0.32 [−0.39, 1.02]	**1.10 [0.31, 1.93]	—	0.04 [−0.84, 0.91]	0.20 [−0.46, 0.86]	0.44 [−0.32, 1.22]	0.30 [−0.90, 1.48]
Exergaming	0.27 [−0.42, 0.97]	**1.05 [0.27, 1.91]	−0.04 [−0.91, 0.84]	—	0.16 [−0.53, 0.84]	0.40 [−0.39, 1.21]	0.25 [−0.93, 1.48]
ME	0.12 [−0.33, 0.56]	**0.89 [0.33, 1.53]	−0.20 [−0.86, 0.46]	−0.16 [−0.84, 0.53]	—	0.25 [−0.30, 0.82]	0.09 [−0.97, 1.15]
RE	−0.13 [−0.74, 0.48]	0.65 [−0.05, 1.40]	−0.44 [−1.22, 0.32]	−0.40 [−1.21, 0.39]	−0.25 [−0.82, 0.30]	—	−0.15 [−1.31, 0.97]
Yoga	0.02 [−1.04, 1.11]	0.80 [−0.32, 1.97]	−0.30 [−1.45, 0.90]	−0.25 [−1.48, 0.93]	−0.09 [−1.15, 0.97]	0.15 [−0.97, 1.31]	—

**BBS**						
	AE	CTE	Dance	Exergaming	ME	RE
AE	—	−0.78 [−1.91, 0.25]	−0.49 [−1.59, 0.63]	−0.44 [−2.11, 1.25]	−0.51 [−1.50, 0.44]	−0.22 [−1.91, 1.45]
CTE	0.78 [−0.25, 1.91]	—	0.30 [−0.77, 1.43]	0.35 [−1.30, 2.04]	0.27 [−0.70, 1.30]	0.56 [−1.11, 2.27]
Dance	0.49 [−0.63, 1.59]	−0.30 [−1.43, 0.77]	—	0.06 [−1.63, 1.74]	−0.02 [−1.08, 0.98]	0.27 [−1.40, 1.97]
Exergaming	0.44 [−1.25, 2.11]	−0.35 [−2.04, 1.30]	−0.06 [−1.74, 1.63]	—	−0.08 [−1.69, 1.50]	0.21 [−1.85, 2.34]
ME	0.51 [−0.44, 1.50]	−0.27 [−1.30, 0.70]	0.02 [−0.98, 1.08]	0.08 [−1.50, 1.69]	—	0.28 [−1.31, 1.93]
RE	0.22 [−1.45, 1.91]	−0.56 [−2.27, 1.11]	−0.27 [−1.97, 1.40]	−0.21 [−2.34, 1.85]	−0.28 [−1.93, 1.31]	—

**GDS**					
	AE	CTE	Dance	ME	RE
AE	—	0.64 [−1.02, 2.34]	−0.21 [−1.89, 1.40]	−0.44 [−2.01, 1.08]	0.07 [−1.84, 1.99]
CTE	−0.64 [−2.34, 1.02]	—	−0.84 [−2.28, 0.54]	−1.08 [−2.34, 0.12]	−0.57 [−2.31, 1.11]
Dance	0.21 [−1.40, 1.89]	0.84 [−0.54, 2.28]	—	−0.24 [−1.51, 1.04]	0.27 [−1.43, 1.98]
ME	0.44 [−1.08, 2.01]	1.08 [−0.12, 2.34]	0.24 [−1.04, 1.51]	—	0.51 [−1.04, 2.08]
RE	−0.07 [−1.99, 1.84]	0.57 [−1.11, 2.31]	−0.27 [−1.98, 1.43]	−0.51 [−2.08, 1.04]	—

**DSF**					
	AE	CTE	Dance	ME	RE
AE	—	−0.16 [−0.83, 0.50]	0.36 [−0.40, 1.14]	0.06 [−0.45, 0.60]	−0.06 [−0.69, 0.64]
CTE	0.16 [−0.50, 0.83]	—	0.52 [−0.31, 1.37]	0.23 [−0.38, 0.86]	0.11 [−0.61, 0.89]
Dance	−0.36 [−1.14, 0.40]	−0.52 [−1.37, 0.31]	—	−0.29 [−1.03, 0.42]	−0.41 [−1.22, 0.42]
ME	−0.06 [−0.60, 0.45]	−0.23 [−0.86, 0.38]	0.29 [−0.42, 1.03]	—	−0.12 [−0.74, 0.53]
RE	0.06 [−0.64, 0.69]	−0.11 [−0.89, 0.61]	0.41 [−0.42, 1.22]	0.12 [−0.53, 0.74]	—

**DSB**					
	AE	CTE	Dance	ME	RE
AE	—	0.05 [−0.59, 0.69]	0.24 [−0.48, 1.01]	0.27 [−0.25, 0.83]	−0.02 [−0.69, 0.66]
CTE	−0.05 [−0.69, 0.59]	—	0.18 [−0.60, 1.00]	0.22 [−0.39, 0.89]	−0.07 [−0.81, 0.68]
Dance	−0.24 [−1.01, 0.48]	−0.18 [−1.00, 0.60]	—	0.03 [−0.69, 0.77]	−0.25 [−1.09, 0.56]
ME	−0.27 [−0.83, 0.25]	−0.22 [−0.89, 0.39]	−0.03 [−0.77, 0.69]	—	−0.28 [−0.95, 0.36]
RE	0.02 [−0.66, 0.69]	0.07 [−0.68, 0.81]	0.25 [−0.56, 1.09]	0.28 [−0.36, 0.95]	—

**SPPB**						
	AE	CTE	Dance	Exergaming	ME	RE
AE	—	−0.63 [−3.39, 2.12]	−0.12 [−2.98, 2.72]	−0.45 [−2.70, 1.69]	−0.74 [−2.89, 1.40]	−1.20 [−3.60, 1.00]
CTE	0.63 [−2.12, 3.39]	—	0.49 [−2.30, 3.26]	0.18 [−2.06, 2.27]	−0.13 [−2.25, 1.96]	−0.56 [−2.94, 1.59]
Dance	0.12 [−2.72, 2.98]	−0.49 [−3.26, 2.30]	—	−0.33 [−2.56, 1.84]	−0.62 [−2.85, 1.54]	−1.08 [−3.56, 1.17]
Exergaming	0.45 [−1.69, 2.70]	−0.18 [−2.27, 2.06]	0.33 [−1.84, 2.56]	—	−0.30 [−1.64, 1.06]	−0.76 [−2.39, 0.77]
ME	0.74 [−1.40, 2.89]	0.13 [−1.96, 2.25]	0.62 [−1.54, 2.85]	0.30 [−1.06, 1.64]	—	−0.46 [−2.14, 1.06]
RE	1.20 [−1.00, 3.60]	0.56 [−1.59, 2.94]	1.08 [−1.17, 3.56]	0.76 [−0.77, 2.39]	0.46 [−1.06, 2.14]	—

**QoL**−**AD**				
	AE	Dance	Exergaming	ME
AE	—	0.05 [−1.31, 1.27]	−0.37 [−1.39, 0.63]	0.21 [−0.76, 0.93]
Dance	−0.05 [−1.27, 1.31]	—	−0.41 [−1.74, 1.02]	0.15 [−1.16, 1.40]
Exergaming	0.37 [−0.63, 1.39]	0.41 [−1.02, 1.74]	—	0.57 [−0.53, 1.47]
ME	−0.21 [−0.93, 0.76]	−0.15 [−1.40, 1.16]	−0.57 [−1.47, 0.53]	—

**Gait speed**				
	AE	Dance	Exergaming	ME
AE	—	0.35 [−3.56, 4.31]	0.62 [−2.71, 3.97]	0.70 [−2.90, 4.09]
Dance	−0.35 [−4.31, 3.56]	—	0.26 [−2.95, 3.59]	0.35 [−3.13, 3.73]
Exergaming	−0.62 [−3.97, 2.71]	−0.26 [−3.59, 2.95]	—	0.08 [−2.86, 2.82]
ME	−0.70 [−4.09, 2.90]	−0.35 [−3.73, 3.13]	−0.08 [−2.82, 2.86]	—

Values represent standardized mean differences (SMDs) with 95% credible intervals. Bolded values with double asterisks ** indicate statistically significant differences at the 95% level, where the credible interval does not include zero.

#### 3.5.1 Mini-mental state examination (MMSE)

A total of 53 studies were included: dance (n = 4), exergaming (n = 3), CTE (n = 5), AE (n = 15), RE (n = 5), and ME (n = 17), comprising 3,107 participants. All interventions were connected through direct or indirect comparisons, with the control groups as the central node. Dance was only directly compared to control. Dance showed no statistically significant differences compared to other interventions, with SMDs ranging from −0.68 (95% CI: −1.88 to 0.56) versus exergaming to 0.39 (−0.49–1.25) versus AE; all 95% Cis crossing the null. Based on SUCRA ranking, dance ranked fourth of seven (46%), behind exergaming (85%), AE (80%), and CTE (57%). Global inconsistency testing revealed significant inconsistency across the network (Q = 19.20, df = 6, *p* = 0.0038), primarily due to comparisons involving control versus ME, RE, and CTE. Node-splitting analysis also indicated inconsistency, particularly for control versus ME comparison. A Bayesian inconsistency model was conducted as a sensitivity analysis. Under this model, SUCRA rankings shifted, with exergaming (100%) and AE (77%) ranking highest, followed by ME (71%) and dance (30%). Several comparisons became significant, including ME and RE versus control, and AE versus RE. In accordance with our prespecified protocol and standard NMA guidance, we prioritized the consistency model for the main analysis because it is parsimonious and readily interpretable.

#### 3.5.2 Montreal cognitive assessment (MoCA)

A total of 40 studies were included: dance (n = 7), exergaming (n = 4), yoga (n = 2), CTE (n = 7), AE (n = 8), RE (n = 4), and ME (n = 8), comprising 2,583 participants. All interventions were connected through direct or indirect comparisons, with the control groups as the central node. Dance was connected to the network through direct comparisons with both the control and AE groups. Dance showed no statistically significant differences compared to other interventions, with SMDs ranging from −0.90 (95% CI: −2.01 to 0.16) versus exergaming to 0.56 (−0.28–1.42) versus CTE. All 95% CIs crossed the null. Dance achieved the highest SUCRA ranking (80%), indicating a high probability of being among the most effective interventions for improving MoCA scores. Global inconsistency was significant (Q = 357.10, df = 40, *p* < 0.0001), largely driven by comparisons involving control versus ME, dance, and exergaming. Node-splitting revealed local inconsistencies, particularly for control versus ME, RE, and exergaming. A Bayesian inconsistency model was conducted as a sensitivity analysis. SUCRA rankings shifted substantially, with exergaming (99%), ME (82%), and RE (72%) rankings highest. Several comparisons became statistically significant, including control versus AE, CTE, ME, and RE. Nevertheless, the consistency model was retained as primary analytical approach, in line with the prespecified protocol and prevailing NMA guidance, given its parsimony and interpretability.

#### 3.5.3 Trail making test-A (TMT-A)

A total of 40 studies were included: dance (n = 7), exergaming (n = 4), yoga (n = 2), CTE (n = 7), AE (n = 8), RE (n = 4), and ME (n = 8), comprising 2,583 participants. All interventions were connected through direct or indirect comparisons, with the control groups serving as the central node. The network plot showed that dance was primarily linked through control and AE groups. Dance showed no statistically significant differences compared to other interventions, with SMDs ranging from −2.04 (95% CI: −3.89 to −0.36) versus exergaming to −0.18 (−2.03 to 1.65) versus RE. All 95% CIs crossed the null, except for comparisons with exergaming and CTE. Dance ranked lowest among the interventions, with a SUCRA score of 35%. Yoga was represented by a single study in the network. The consistency assumption was supported, with no significant inconsistency detected in the network.

#### 3.5.4 Trail making test-B (TMT-B)

A total of 26 studies were included: dance (n = 4), exergaming (n = 4), yoga (n = 2), CTE (n = 4), AE (n = 3), RE (n = 3), and ME (n = 6), comprising 1,860 participants. All interventions were connected through direct or indirect comparisons, with the control groups as the central node. Dance was directly compared to control and AE groups. Dance showed no statistically significant differences compared to other interventions, with SMDs ranging from −1.32 (95% CI: −2.98 to 0.28) versus CTE to 1.32 (−0.28–2.98) versus CTE. All 95% CIs crossed the null. Dance ranked lowest among the interventions, with a SUCRA score of 37%. The consistency assumption was supported, with no significant inconsistency detected in the network.

#### 3.5.5 Timed up and go (TUG)

A total of 37 studies were included: dance (n = 3), exergaming (n = 2), yoga (n = 1), CTE (n = 5), AE (n = 7), RE (n = 4), and ME (n = 14), comprising 1,997 participants. All interventions were connected through direct or indirect comparisons, with the control groups as the central node. Dance was directly compared to control and AE groups. Dance showed no statistically significant differences compared to other interventions, with SMDs ranging from −1.10 (95% CI: −1.93 to −0.31) versus CTE to 0.44 (−0.32–1.22) versus RE. All 95% CIs crossed the null. Dance had the lowest probability (34%) of being the most effective intervention. Yoga was represented by a single study in the network. The consistency assumption was supported, with no significant inconsistency detected in the network.

#### 3.5.6 Berg balance scale (BBS)

A total of 16 studies were included: dance (n = 3), exergaming (n = 1), CTE (n = 3), AE (n = 3), RE (n = 1), and ME (n = 5), comprising 1,401 participants. In the network, all interventions were connected solely through comparisons with the control groups, forming a star-shaped structure. Dance did not show statistically significant differences compared to other interventions, with SMDs ranging from −0.49 (95% CI: −1.59 to 0.63) versus AE to 0.30 (−0.77–1.43) versus CTE. All 95% CIs crossed the null. Dance achieved a SUCRA value of 54%, placing it in the mid-range among all interventions. AE had the highest SUCRA score (82%), followed by RE (67%) and exergaming (58%), while CTE ranked lowest (36%). Both the exergaming and RE interventions were each represented by only one study in the network. No inconsistency analysis was conducted, as the network lacked closed loops.

#### 3.5.7 Geriatric depression scale (GDS)

A total of 10 studies were included: dance (n = 2), CTE (n = 2), AE (n = 1), RE (n = 1), and ME (n = 4), comprising 730 participants. In the network, all interventions were connected solely through comparisons with the control groups, forming a star-shaped structure. Dance did not demonstrate statistically significant differences compared to other interventions, with SMDs ranging from −0.84 (95% CI: −2.28 to 0.54) versus CTE to 0.27 (−1.43–1.98) versus RE. All 95% CIs crossed the null. Dance achieved a SUCRA value of 50%, placing it in the middle range among the evaluated interventions. CTE ranked highest with a SUCRA score of 90%, followed by RE (64%) and AE (61%), while ME ranked lowest (35%). It is important to note that both AE and RE were each represented by only one study in the network. No inconsistency analysis was conducted, as the network lacked closed loops.

#### 3.5.8 Digit span forward (DSF)

A total of 16 studies were included: dance (n = 1), CTE (n = 3), AE (n = 3), RE (n = 3), and ME (n = 6), comprising 1,641 participants. All interventions were connected through direct or indirect comparisons, with the control groups as the central node. In the network, dance was directly compared to the control and AE groups. Dance did not demonstrate statistically significant differences compared to other interventions, with SMDs ranging from −0.52 (95% CI: −1.37 to 0.31) versus CTE to 0.41 (−0.42–1.22) versus RE. All 95% CIs crossed the null. Dance achieved the highest surface under the SUCRA value of 88%, suggesting the greatest probability of being the most effective intervention among those assessed. Dance was followed by ME (65%), AE (57%), RE (51%), and CTE (40%). Dance intervention was informed by only one study in the network. Although the network comprised multiple indirect comparisons, inconsistency assessment was not prioritized due to the limited number of closed loops involving dance.

#### 3.5.9 Digit span backward (DSB)

A total of 15 studies were included: dance (n = 1), CTE (n = 3), AE (n = 3), RE (n = 3), and ME (n = 5), comprising 1,552 participants. All interventions were connected through direct or indirect comparisons, with the control groups as the central node. In the network, dance was connected to the network via direct comparisons with AE and control groups. Dance did not show statistically significant differences compared to other interventions, with SMDs ranging from −0.25 (95% CI: −1.09 to 0.56) versus ME to 0.25 (−0.56–1.09) versus RE. All 95% CIs crossed the null. Dance achieved a SUCRA score of 73%, ranking second among all interventions. The highest SUCRA score was observed for ME (80%), followed by dance (73%), CTE (54%), and both RE and AE (47%). Dance intervention was informed by only one study in the network. The consistency assumption was supported, with no significant inconsistency detected in the network.

#### 3.5.10 Short physical performance battery (SPPB)

A total of 12 studies were included: dance (n = 1), exergaming (n = 3), CTE (n = 1), RE (n = 3), and ME (n = 4), comprising 836 participants. In the network, all interventions were connected through direct or indirect comparisons, with the control groups as the central node. Dance was only directly compared to the control groups. Dance did not show statistically significant differences compared to other interventions, with SMDs ranging from −0.49 (95% CI: −3.26 to 2.30) versus CTE to 1.08 (−1.17–3.56) versus RE. All 95% CIs crossed the null. Dance achieved a SUCRA score of 72%, suggesting a relatively high probability of being among the more effective interventions. AE had the highest SUCRA score (76%), followed by dance (72%), exergaming (63%), CTE (55%), ME (50%), and RE (33%). Dance and CTE interventions were each informed by only one study in the network. No inconsistency analysis was conducted, as the network lacked closed loops.

#### 3.5.11 Quality of life in alzheimer’s disease (QoL-AD)

A total of 9 studies were included: dance (n = 1), exergaming (n = 2), AE (n = 3), and ME (n = 3), comprising 827 participants. In the network, all interventions were connected solely through comparisons with the control groups, forming a star-shaped structure. Dance did not show statistically significant differences compared to other interventions, with SMDs ranging from −0.41 (95% CI: −1.74 to 1.02) versus exergaming to 0.15 (−1.16–1.40) versus ME. Dance had a SUCRA score of 67%, second only to ME (80%), and higher than AE (64%) and exergaming (40%). Dance was informed by only one study in the network. No inconsistency analysis was conducted, as the network lacked closed loops.

#### 3.5.12 Gait speed

A total of 6 studies were included: dance (n = 1), exergaming (n = 2), AE (n = 1), and ME (n = 2), comprising 193 participants. In the network, all interventions were connected solely through comparisons with the control groups, forming a star-shaped structure. Dance did not show statistically significant differences compared to other interventions, with SMDs ranging from −0.35 (95% CI: −4.31 to 3.56) versus AE to 0.35 (−3.13–3.73) versus ME. Based on SUCRA ranking, ME ranked highest (73%), followed by exergaming (69%), dance (59%), and AE (49%). Only one study contributed data for both dance and AE in the network. No inconsistency analysis was conducted, as the network lacked closed loops.

## 4 Discussion

This systematic review and NMA evaluated the relative efficacy of dance, exergaming, yoga, CTE, AE, RE, and ME on cognitive function, psychological wellbeing, physical performance, and QoL in individuals with MCI and dementia. To our knowledge, this is the first comprehensive NMA to synthesize evidence from a large sample size while simultaneously examining a broad spectrum of outcome domains. By incorporating diverse cognitive, psychological, and physical measures, the study provides a holistic comparison of non-pharmacological, exercise-based interventions, highlighting the distinctive contribution of dance in cognitive rehabilitation and patient-centered outcomes.

In NMA, findings provided nuanced estimates of relative efficacy across exercise modalities. Although direct head-to-head evidence for dance was limited, indirect comparisons yielded informative rankings; nevertheless, several confidence intervals remained wide, reflecting the limited number and variable quality of dance-specific trials. Notably, under the consistency model, dance had the highest probability of improving global cognition on MoCA, outperforming AE, yoga, RE, ME, CTE, and exergaming, but these rank advantages did not translate into statistically significant pairwise effects. Results from the inconsistency model did not always align with the consistency model, likely due to clinical and methodological heterogeneity, imbalanced direct comparisons, and unaccounted effect modifiers across studies. This pattern differs slightly with a prior report indicating RE as the most effective global cognition (Wang et al., 2019; Huang et al., 2022; Lv et al., 2023). For DSF outcome (short-term verbal memory/attention span), dance also ranked first, followed by ME, AE, RE, and CTE. For DSB (working memory, attentional control, and executive functioning), SPPB (physical performance) and QoL-AD outcomes, dance consistently ranked second, again without statistically significant superiority. Considered together, and consistent with prior meta-analytic findings (Quan et al., 2024), these results suggest that dance may confer a broad spectrum of benefits across cognitive, physical, and QoL in individuals with MCI and dementia. Although not the top-ranked intervention for every outcome, dance consistently performed well across key domains, underscoring its multidimensional value. Nevertheless, effect estimates remain imprecise, and rank-based advantages should be interpreted cautiously pending more high-quality, head-to-head trials.

The unique advantages of dance likely stem from its integration of cognitive stimulation, aerobic and motor coordination, rhythmic entrainment, and social interaction, a combination that may be particularly beneficial for neuroplasticity and emotional wellbeing. These multifaceted components may explain why dance shows favorable effects across variable outcomes and among diverse populations (Tao et al., 2023b; Zhang et al., 2025). Zhang et al. examined five dance styles in older adults and concluded that ballroom and square dance appeared most effective for enhancing cognitive function and mental health, whereas tango showed potential benefits for balance and mobility (Zhang et al., 2025). In contrast, the present study treated dance as a single intervention category, irrespective of style. Here, “dance” denotes structured, rhythmic sequences performed to music, with coordinated whole-body movements, repetition and variation of choreographed patterns, and an explicit intention to engage aesthetic, social, and cognitive processes beyond general exercise. The definition targets mechanisms relevant to MCI and dementia, including dual-tasking, motor-cognitive integration, rhythm-based entertainment, and social engagement. This approach facilitates network synthesis across heterogeneous trials but may dilute style-specific effects observed in dance-specific analyses.

Dance involves complex motor coordination, balance, and mobility, capacities that may diminish with age due to reduced joint flexibility, muscle strength, and postural control (Tao et al., 2021b; Tao et al., 2023b). Our analysis reflected this pattern, showing fewer participants older than 70 years. Nevertheless, the inclusion of individuals aged 76–85 years in several studies suggests that dance remains feasible for this age group with cognitive impairment. Adapted formats, such as low-impacted or seated dance routines, can enhance feasibility and engagement among frail or less mobile participants. In addition, integrating strength training and fall-prevention components may help sustain participation. As outlined in the recommendations section following this discussion, dance programs should be tailored to participants’ cognitive status, physical capabilities, and preferences. Although execution quality (e.g., completeness of movements and posture accuracy) is relevant to effectiveness, the cognitive gains arising from the learning process itself should not be overlooked. In view of the characteristics of this population, it is essential to structure sessions to fostered cognitive engagement without provoking undue stress or frustration. Accordingly, balancing movement standardization with participants’ motivation and adherence is key to maximizing both cognitive and physical benefits.

Dance movement therapy (DMT), employed in some included studies (Ho et al., 2018; Esmail et al., 2020), represents a psychotherapeutic application of movement, as defined by the American Dance Therapy Association (ADTA) (American Dance Therapy Association, 2025). Its goal is to promote integration across emotional, cognitive, and physical integration. DMT is especially suitable for individuals with moderate to severe cognitive impairment and those without prior dance experience. However, its broader implementation is challenged by limited availability of certified dance therapists and associated costs. Expanding access to DMT through certification programs could enhance its scalability and accessibility.

Other exercise modalities likewise performed well on selected outcomes and merit consideration. CTEs such as Tai Chi and Baduanjin, which emphasize mind-body integration, performed well in the NMA on processing speed, attention (TMT-A), exercise function, cognitive flexibility (TMT-B), mobility, dynamic balance (TUG), and depressive symptoms/mood (GDS), aligning with prior findings (Liu et al., 2023). Such culturally aligned activity may be particularly beneficial for older populations (Sungkarat et al., 2018). Exergaming represents an innovative modality that integrates physical and cognitive training (Wu et al., 2023), and is well suited for home-based implementation, reducing caregiver burden and logistical constraints (Aguiñaga et al., 2021; Tao et al., 2024). Our NMA indicates that exergaming improves global cognition (MMSE), and good performance on physical performance (BBS, SPPB and gait speed), although previous reviews suggest limited effects on PA itself (Voinescu et al., 2021). Importantly, during public health emergencies such as the COVID-19 pandemic, exergaming and online classes offer viable alternatives to maintain activity levels among vulnerable populations (Tao et al., 2024). Multicomponent interventions also demonstrated positive outcomes on working memory, executive control (DSB), QoL (quality of life in AD), and gait speed; a holistic design may confer synergistic benefits cross multiple domains (Lv et al., 2023; Zeng et al., 2025). However, the composite design makes it challenging to disentangle the specific contribution of each element. Among the commonly used exercise modalities, AE demonstrated notable strengths, particularly in enhancing balance and lower-limb function, as reflected by its top rankings in both the BBS and the SPPB. RE also showed considerable benefits, ranking second in TUG and BBS physical performance measures, as well as in reducing negative mood (GDS).

The World Health Organization (WHO) advised that adults aged 65 years and older accumulate 150–300 min of moderate-intensity aerobic activity per week, 75–150 min of vigorous-intensity activity, or an equivalent mix of the two, to achieve substantial health benefits (Green and Smith, 2016). Our synthesis suggests that a 60-min dance session performed three times weekly aligns with these guidelines and is well tolerated by individuals with MCI and dementia. Several included studies also involved caregivers alongside participants, enabling caregivers to provide physical and emotional support during the interventions (Liu et al., 2018; Lowery et al., 2014; Prick et al., 2017; Venturelli et al., 2011; Vreugdenhil et al., 2012). This dual benefit highlights the value of caregiver inclusion, particularly given the central yet under-supported role of informal caregivers (often spouses) in dementia care (Zhu et al., 2024). Although subgroup analyses comparing trials with versus without caregiver involvement were not feasible due to limited numbers within each modality, it is plausible that caregiver participation enhances intervention adherence and outcomes. Moreover, caregivers themselves may derive physical and psychological benefits, including reduced stress associated with caregiving responsibilities (Cheng et al., 2022).

Despite its strengths, this study has several limitations. First, although only RCTs were included to increase methodological rigor, many comparisons relied on indirect evidence and the assumption of transitivity, which may introduce bias. Second, participants with MCI and dementia were merged for analysis. We recognized these populations differ substantially; however, the limited number of studies per outcome in the NMA necessitated combining them to preserve network connectivity and statistical power. Moreover, most included studies enrolled participants with MCI, which limits the generalizability of findings to individuals with moderate or severe dementia. Third, due to the data limitations, we were unable to include more comparisons involving dance programs; in some cases, only a single dance-related study was available in the network. This limits the robustness and generalizability of the effect estimates and SUCRA rankings for the interventions. Last, several unmeasured variables may have influenced the observed outcomes, including the integration of music, the presence of caregivers, education level, and gender bias.

Future research should examine these moderators to clarify the effects of dance and other exercise modalities. Moreover, identifying individual characteristics that predict responsiveness to interventions could improve personalized treatment strategies. Control-group selection similarly warrants attention; given the blinding challenges in behavioral interventions, employing both active and passive controls may help mitigate expectancy effects. Moreover, future studies should investigate multicomponent intervention programs that incorporate cognitive training, health education, social engagement, and peer support to yield a more comprehensive and accurate appraisal effectiveness in real-world settings.

Drawing on this systematic review, we propose practical, evidence-informed recommendations for exercise-based interventions, complemented by a dedicated set for dance-based programs tailored to individuals with MCI and dementia. These are summarized in two tables (Supplementary Table S10). Leveraging the large sample based synthesized here, the guidance offers concise, actionable advice for time-constrained health and social care professionals, as well as for people living with cognitive impairment across disease stage and their caregivers.

## 5 Conclusion

This study provides robust evidence that exercise-based interventions—particularly dance—improve cognitive, psychological, and physical outcomes in individuals with MCI or dementia. Dance appears especially promising modality given its multimodal nature, integrating movement, rhythm, cognition, and social interaction. Incorporating dance-based and other structured exercise programs as complementary treatments may enhance the effectiveness of conventional cognitive care. Our findings support the broader adoption of non-pharmacological strategies across clinical, community, and rehabilitation settings and highlight the necessity for policies that promote active aging and caregiver inclusion. These results can inform evidence-based decision-making and motivate further research into cost-effective, scalable approaches for the preventing and managing cognitive impairment.

## Data Availability

The original contributions presented in the study are included in the article/Supplementary Material, further inquiries can be directed to the corresponding author.
