# Supplementary material for: Dance versus other exercise modalities in mild cognitive impairment and dementia: comparative efficacy from a systematic review and bayesian network meta-analysis
Source: Front Physiol. 2026 Mar 25;17:1782774. doi: 10.3389/fphys.2026.1782774 (PMC13056856; doi:10.3389/fphys.2026.1782774)
Supplement: Supplementary file 1 [file Table5.pdf]

Supplementary Table 5. Outcome Measurement Instruments

Intervention categories	Outcomes	Measurement Instruments
**Dance**		
	Global Cognitive Function–Cognitive Assessment, Orientation	1. Mini-Mental State Examination (MMSE)[1–8,9]<br>2. Dual-task Paradigm[10]<br>3. Functional and Cognitive Assessment Test (FUCAS)[3]
	Global Cognitive Function–Executive Function	4. Montreal Cognitive Assessment (MoCA)[2–4,6,8,10–14,9]<br>5. Trail Making Test-B (TMT-B)[1–3,5,8,12,15,4,9]<br>6. Five-point Test[11]
	Global Cognitive Function–Memory	7. Wechsler Memory Scale (WMS)[1,2,8,11,12]<br>8. Digit Span Forward (DSF)[2,8,12,15]<br>9. Digit Span Backward (DSB)[2,8,12,15]<br>10. The Behavioral Memory Test (RBMT)[3]<br>11. The Fuld Objective Memory Evaluation[15]<br>12. The N-back test[10]<br>13. Logical Memory Delayed Recall after 30 min[11]<br>14. Visual Memory subtest of the Repeatable Battery for the Assessment of Neuropsychological Status (RBANS)[1]<br>15. Digit Span Subtest from WAIS III[11]<br>16. Category Verbal Fluency (CVF)[1]<br>17. The Boston Naming Test (BNT)[1]<br>18. Neuropsychological Status (RBANS)[1]<br>19. The Amsterdam Dementia Screening Test 6 (ADS 6)[6]
	Global Cognitive Function–Processing Speed	20. Trail Making Test-A (TMT-A)[1,2,4,5,8,12,15,9]<br>21. The Symbol Digit Modalities Test (SDMT)[2,8,12]<br>22. The Digit symbol substitution test (DSST)[16]<br>23. Symbol Search Subtest from WAIS III[11]
	Global Cognitive Function–Attention	24. Test of Everyday Attention (TEA)[3]<br>25. The Digit Stroop Color and Word Test (SCWT)[10]<br>26. Flanker Interference Task[16]<br>27. Walking While Taking (WWT)[16]
	Global Cognitive Function–Problem-solving Skills	28. Tower of Hanoi (3 and 4 disks)[11]
	Global Cognitive Function -Language	29. The Verbal Fluency F-A-S Test (FAS)[3]<br>30. Letter Verbal Fluency (LVF)[1]

		31. The Isaac Test[9]
	Global Cognitive Function–Visuospatial Constructional Ability	32. The Judgment of Line Orientation test[1,11]<br>33. Taylor Figure Test copy[11]<br>34. Rey Osterrieth Complex Figure Test Copy and Delay Recall (ROCFT-copy and delayed recall)[3]<br>35. Taylor Figure Test Recall 3 min after copy[11]<br>36. Taylor Figure Test Recall 30 min after copy[11]
	Psychological Wellbeing	37. Geriatric Depression Scale (GDS)[3,6,8,10,12,14,15]<br>38. Neuropsychiatric Inventory (NPI)[3,7]<br>39. Beck Depression Inventory (BDI)[3,11]<br>40. State-Trait Anxiety Inventory (STAI)[10]<br>41. Hospital Anxiety and Depression Scale (HADS)[1]<br>42. Hamilton Scale for Depression [3]<br>43. Perceived Stress Scale (PSS)[3]<br>44. Beck Anxiety Inventory[3]<br>45. The de Jong Fierveld Longlines Scale[15]<br>46. Visual Analogue Mood Scale[15]<br>47. Behavioural and Psychological Symptoms of Dementia (BPSD)[15]<br>48. Mental Health Continuum-Short Form (MHC)[10]<br>49. The Yesavage Geriatric Depression Scale[9]
	Neurological Conditions	50. Clinical Dementia Rating Scale (CDR)[15]
	Functional Ability and Mobility	51. Timed Up and Go (TUG)[7,10]<br>52. Berg Balance Scale (BBS)[2]<br>53. The 10-m Walk Test[10]<br>54. Falls Efficacy Scale[4]<br>55. The 4-m Gait Speed Test[7]<br>56. Short Physical Performance Battery (SPPB)[7]<br>57. The Single-leg Stance with Eyes Closed Test[4]<br>58. National Center for Geriatrics and Gerontology Functional Assessment Tool (NCGG-FAT)[5]<br>59. The Sit-to-Stand Test[17]
	Strength	60. Handgrip Strength[10]
	Health Related and QoL	61. 36-Short Form Health Survey (36-SF)[1,8,12]<br>62. Quality of Life (QoL) self-administered, SF-12[10,14]<br>63. Pittsburgh Sleep Quality Index (PSQI)[10,13]<br>64. Quality of Life in Alzheimer's Disease (QoL-AD)[7]<br>65. Short-Form 12 Health Survey (SF-12)[14]<br>66. FRAIL screening questionnaire[9]
	Self-dependency	67. Bristol Activities of Daily Living Scale[11]

		68. Functional Activities Questionnaire (FAQ)[12]<br>69. Katz Index of Independence in Activities of Daily Living[7]
	Cardiovascular Fitness	70. VO_2peak test[10]<br>71. Cardiopulmonary Exercise Testing (CPET)[17]
	Brain Imaging	72. Magnetic Resonance Imaging (MRI)[2,11,8]<br>73. Resting-state Functional Magnetic Resonance Imaging (RS-fMRI)[8,16]
	Biomarker	74. Saliva Sample (cortisol measures)[15]
	Lifestyle	75. Health Promoting Lifestyle Profile-2 (HPLP2)[10]
	Physiological	76. Heart Rate Variability (HRV)[17]
**Exergaming**		
	Global Cognitive Function–Cognitive Assessment, Orientation	1. Mini-Mental State Examination (MMSE)[18–20,21]
	Global Cognitive Function–Executive Function	2. Trail Making Test-B (TMT-B)[19,22–26]<br>3. Montreal Cognitive Assessment (MoCA)[23–25,27,19]<br>4. The Executive Interview 25 (EXIT-25)[25]<br>5. Rule Shift Cards Test[26]
	Global Cognitive Function–Memory	6. Digit Span Forward (DSF)[22,26]<br>7. The California Verbal Learning Test (CVLT)[24,25]<br>8. Digital Span Backward (DSB)[22]<br>9. Logical Memory subtest (Story Recall) of Wechsler Adult Intelligence Scale-Revised (WAIS-R)[22]<br>10. Wechsler Memory Scale-Revised (WMS-R)[22]<br>11. The N-back Test[25]<br>12. The Spatial N-back Task Test[24]<br>13. Location Learning Test-Revised[26]<br>14. Dual-task Cost (DTC)[24]
	Global Cognitive Function–Processing Speed	15. Trail Making Test-A (TMT-A)[22,23,24,25,26,19]<br>16. Digit Symbol Substitution Test (DSST)[22]
	Global Cognitive Function–Attention	17. The Eriksen Flanker Test[28]<br>18. The Stroop Color and Word Test (SCWT)[24–26]
	Global Cognitive Function–Language	19. The Letter Verbal Fluency Test[26]
	Decision-making	20. The Step Reaction Time Test (SRTT)[27]
	Psychological Wellbeing	21. Neuropsychiatric Inventory (NPI)[27,19]<br>22. The Cornell Scale for Depression in dementia (CSDD)[20,27]<br>23. Physical Activity Enjoyment Scale (PACES)[22]<br>24. Center for Epidemiology Studies Depression Scale (CES-D)[23]
	Neurological Conditions	25. The Computerized Assessment of Mild Cognitive Impairment (CAMCI)[29]

		26. The Cognitive Self-Report Questionare-25[29]
	Functional ability and Mobility	27. Short Physical Performance Battery (SPPB)[19,27]<br>28. Gait Speed and Variability (validated algorithms)[23,24]<br>29. 6-m Walk Test (6MWT)[29]<br>30. Wearable Sensors (balance)[23]<br>31. Berg Balance Scale (BBS)[18]<br>32. Tinetti Assessment Tool (TAT)[18]<br>33. Timed Up and Go (TUG)[18,21]
	Health Related and QoL	34. Quality of Life in Alzheimer's Disease (QoL-AD)[18,20]<br>35. Dementia Quality of Life (DQoL)[27]<br>36. The Senior Fitness Test[28]<br>37. The EuroQoL-5 Dimension (EQ-5D)[19]<br>38. Visual Analogue Scale[23]<br>39. Tinetti Falls Efficacy Scale[21]
	Self-dependency	40. Activities of Daily Living Inventory (ADL)[18]<br>41. Katz Index of Independence in Activities of Daily Living[27]<br>42. The Time Instrumental Activities of Daily Living[29]<br>43. The Barthel Index of Activities of Daily Living (ADL)[23]
	Brain Imaging	44. Electroencephalographic (EEG) Measurement[28]<br>45. Functional Near-infrared Spectroscopy (fNIRS)[25]
	Gene Identification	46. Saliva Sample (the apolipoprotein E (APOE) genotype)[26]
**Yoga**		
	Global Cognitive Function–Cognitive assessment, Orientation	1. Mini-Mental State Examination (MMSE)[30,31]<br>2. Neuropsychological Test Battery[32]<br>3. Everyday Cognition Scale[32]<br>4. Event-related Potential (P300)[31]
	Global Cognitive Function–Executive Function	5. Trail Making Test-B (TMT-B)[30,32–34]<br>6. Montreal Cognitive Assessment (MoCA)[30,31]<br>7. Frontal Assessment Battery (FAB)[31]
	Global Cognitive Function–Memory	8. Hopkins Verbal Learning Test-Revised (delayed recall)[33,34,32]<br>9. Rey-Osterreith Complex Figure Delayed Recall Trial[33,34]<br>10. Wechsler Memory Scale-Revised (WMS-R)[33,34]<br>11. The Memory Function Questionnaire (MFQ)[33]<br>12. Wechsler Adult Intelligence Scale-IV Letter-Number Sequencing Subtest[32]
	Global Cognitive Function–Processing Speed	13. Trail Making Test-A (TMT-A)[30,32]<br>14. Wechsler Adult Intelligence Scale-IV Digit Symbol[32]
	Global Cognitive Function–Attention	15. The Stroop Word-Color Test[34]

		16. Stroop Color and Word Interference[33]
	Global Cognitive Function–Language	17. Hopkins Verbal Learning Test-Revised[32,33]<br>18. Controlled Oral Ward Association[32]<br>19. The Verbal Fluency Test (VF)[30]
	Global Cognitive Function–Visuospatial constructional ability	20. Rey-Osterrieth Complex Figure-Presence/Accuracy and Organization Scores[32]<br>21. Brief Visuospatial Memory Test-Revised[32]
	Psychological Wellbeing	22. Perceived Stress Scale (PSS)[32,33]<br>23. Geriatric Depression Scale (GDS)[34]<br>24. Beck Depression Inventory (BDI)[33]<br>25. Centers for Epidemiological Studies Depression Scale[32]<br>26. State-Trait Anxiety Inventory[32]<br>27. Hamilton Anxiety Rating Scale (HAM-A)[33]<br>28. Positive and Negative Affect Schedule[32]<br>29. Apathy Evaluation Scale (AES)[34]<br>30. Connor-Davidson Resilience scale[34,33]<br>31. The Five-Factor Mindfulness Questionnaire[32]<br>32. Clinician-Rated Dimensions of Psychosis Symptom Severity (CDPSS)[31]
	Functional ability and Mobility	33. Timed Up and Go (TUG)[30,32]<br>34. 10-m normal Walking speed (NWS)[30]<br>35. 5-m High Walking speed (HWS)[30,31]
	Strength	36. Handgrip Strength[32]
	Health Related and QoL	37. 36-Short Form Health Survey (36-SF)[33,32]
**Chinese Traditional Exercise (CTE)**		
	Global Cognitive Function–Cognitive Assessment, Orientation	1. Mini-Mental State Examination (MMSE)[35–40,41]<br>2. Alzheimer's Disease Assessment Scale-Cognitive Subscale (ADAS-Cog)[35]
	Global Cognitive Function–Executive Function	3. Montreal Cognitive Assessment (MoCA)[37,40,42–48]<br>4. Trail Making Test-B (TMT-B)[35,37,42,43,45,46,48,49]
	Global Cognitive Function–Memory	5. Digit Span Backward (DSB)[35,38,42,45,49]<br>6. Digit Span Forward (DSF)[35,38,42,45,49]<br>7. Wechsler Memory Scale (WMS)[44,47,49]<br>8. The N-back Test[50]<br>9. The 30-min Delay Recall Test[38,45]<br>10. The Auditory Verbal Learning test (AVLT)[46,48]<br>11. Visual Span Forward[35]<br>12. Visual Span Backward[35]<br>13. Wechsler Memory Quotients (MQ)[43]

		14. The Boston Naming Test (BNT)[43]<br>15. Memory Inventory for Chinese (MIC) Questionnaire[39]<br>16. Subjective Memory Complaint Questionnaire (SMCQ)[46]<br>17. Dual-task Cost (DTC)[42]
	Global Cognitive Function–Processing speed	18. Trail Making Test-A (TMT-A)[35,37,43,45,46,48,49]<br>19. Digit Symbol Substitution Test (DSST)[43]<br>20. Digit Symbol Coding (DSC)[48]
	Global Cognitive Function–Attention	21. Test of Attention Performance (TAP)[48]<br>22. The Victoria Stroop Test[45]
	Global Cognitive Function–Language	23. The Categorical Verbal Fluency Test (VFT)[38]
	Global Cognitive Function–Learning	24. The WHO-University of California Los Angeles-Auditory Verbal Learning test (WHO-UCLA-AVLT)[37]
	Global Cognitive Function–Visuospatial constructional ability	25. The Rey-Osterrieth Complex Figure Test (ROCF)[43]<br>26. Clock Drawing Task (CDT)[48]
	Psychological Wellbeing	27. Geriatric Depression Scale (GDS)[37,38,46]<br>28. The Cornell Scale for Depression in dementia (CSDD)[35,48]<br>29. Neuropsychiatric Inventory (NPI)[35,37]
	Neurological Conditions	30. Clinical Dementia Rating (CDR)[35,36]
	Functional ability and Mobility	31. Timed Up and Go (TUG)[36,40,42,51–53]<br>32. Berg Balance Scale (BBS)[35,40,53]<br>33. The 30-second Chair Stands Test[36,51,41]<br>34. The Incidence of Falls[51]<br>35. The 4-Stage Balance Test[51]<br>36. Short Physical Performance Battery (SPPB)[36,54]<br>37. The Five Times Chair Stand Test[45]<br>38. The Timed-Chair- Stand Test[52]<br>39. The Single Led Stand Test[45]<br>40. The Functional Reach Test[52]<br>41. The Step Test[52]<br>42. Tinetti Assessment Tool (TAT)[55]<br>43. The Tampa Scale of Kinesiophobia (TSK)[55]<br>44. The Falls Behavioral (FaB) Acale[55]<br>45. The 6-minute Walk Test (6MWT)[41]
	Physical Activity Level	46. The Physical Activity Scale for the Elderly (PASE)[55]
	Health Related and QoL	47. Short-Form 12 Health Survey (SF-12)[45,39]<br>48. The Pittsburgh Sleep Quality Index (PSQI)[39,45,54]<br>49. The SF-12 quality-of-life questionnaire[40,41]

		50. Fried Frailty Criteria[36]<br>51. Quality of Life in Alzheimer's Disease (QoL-AD)[54]
	Self-dependency	52. The Barthel Index of Activities of Daily Living (ADL)[37]<br>53. The General Self-efficacy Scale (GSES)[46]
	Brain Imaging	54. Magnetic Resonance Imaging (MRI)[44,47]<br>55. EEG[56]
	Biomarkers	56. Blood sample (BDNF)[49]<br>57. Blood sample (Serum levels of fasting glucose, insulin, glycated hemoglobin (HbA1c), advanced glycation end products (AGE), and soluble receptor of AGE (sRAGE)[43]<br>58. Blood Samples (serum exosome isolation, identification of exosomes, proteomics of exosomes and validation)[44]
	Gene Identification	59. APOE Genotyping[38]
	Strength	60. Handgrip strength[41]
**Aerobic exercise (AE)**		
	Global Cognitive Function–Cognitive assessment, Orientation	1. Mini-Mental State Exam (MMSE)[57–77]<br>2. The Alzheimer's Disease Assessment Scale-cognitive (ADAS-Cog)[66,69,78,79,80,81,82]<br>3. The Computer-based CogState Battery[83]<br>4. The Cambridge Cognitive Examination (CAMCOG)[84]<br>5. Raven's Coloured Progression Matrices (CPM)[70]<br>6. Cognitive Change Index[80]
	Global Cognitive Function–Executive Function	7. Montreal Cognitive Assessment (MoCA)[60,65,67,75,85–91,74]<br>8. Trail Making Test-B (TMT-B)[59,61,67,75,88,92–94,74]<br>9. Delis-Kaplan Executive Function System (D-KEFS)[57,95]<br>10. Frontal Assessment Battery (FAB)[70]<br>11. Frontal Executive Functioning Tests (namely, the category naming)[93]<br>12. National Institutes of Health (NIH) Toolbox-Cognition Battery[90]<br>13. Walking Stroop Carpet Test (WSC)[96]<br>14. Task-switching Task[97]<br>15. Behavior Rating Inventory of Executive Function for Adults (BRIEF-A)[80]
	Global Cognitive Function–Memory	16. Digit Span Forward (DSF)[71,84,88,95,96,98,99,74]<br>17. Digit Span Backward (DSB)[84,88,95,96,99,71,74]<br>18. The Wechsler Memory Scale-Revised (WMS-R)[61,73,93,74]<br>19. The Ray Auditory Verbal Learning Test (RAVLT)[70,71,100]<br>20. The Riverhead Behavioral Memory Test[93,99]<br>21. The California Verbal Learning Test (CCVLT)[57,93,93]<br>22. The Story Recall Test[92]

		23. The Delayed-Match-To-Sample Test[92]<br>24. 1-back Task[97]<br>25. Logical Memory (immediate and delayed)[95]<br>26. Free and Cued Selective Reminding Test (sum of free recall)[95]<br>27. The Boston Naming Test[95]<br>28. The Prose Memory test[70]<br>29. The Fuld Object Memory Evaluation (FOME)[77]<br>30. Category Verbal Fluency (CVF)[95,99]<br>31. Letter Number Sequencing[95]<br>32. Spatial Working Memory[97]<br>33. Cognitive Self-Report Questionnaire (CSRQ)[90]<br>34. Working Memory Dual-task (WMDT)[91]<br>35. Semantic Memory Dual-task (SMDT)[91]
	Global Cognitive Function–Processing speed	36. Trail Making Test-A (TMT-A)[59,61,63,75,84,88,94,67,74,93]<br>37. The Digit Symbol subtest of the Wechsler Adult Intelligence Scale-III (WAIS-III)[61,98]<br>38. Symbol Digit Modalities Test (SDMT) [92,74]
	Global Cognitive Function–Attention	39. The Stroop Color and Word Test (SCWT)[61,95,98,96,92,88,84]<br>40. Stroop Color and Word Interference[95,70]<br>41. The Stroop Test Color[61,98,70,82]<br>42. Visual Attention Assessment[70]<br>43. Neurobehavioral Cognitive Status Examination (NCSE)[94]<br>44. Visual Attention Assessment[70]
	Global Cognitive Function–Language	45. Category Verbal Fluency Test animal (VFT)[94,61]<br>46. The Verbal Fluency test (animal category)[84,94]<br>47. Letter Verbal Fluency (LVF)[61,99]<br>48. Word Fluency (WF)[63]
	Global Cognitive Function–Visuospatial constructional ability	49. Spatial Working Memory[100,97]<br>50. The Clock Drawing Test (CDT)[84]<br>51. Mental Rotation Test (MRT)[73]<br>52. Stick Design Test (SDT)[88]<br>53. Copying Geometric Drawings (CGD)[70]<br>54. Spatial Orientation Test (SOT)[73]
	Decision-making	55. The Simple Reaction Times (SRT) test[72]<br>56. The Choice Reaction Time (CRT) test[72,100]<br>57. Reaction Time Test (RTT)[73,98]
	Psychological Wellbeing	58. Geriatric Depression Scale (GDS)[58,65,85,100,74,80]<br>59. The Cornell Scale for Depression in dementia (CSDD)[69,77,95]

	60. Hospital Anxiety and Depression Scale (HADS)[59,90]
	61. Beck Depression Inventory (BDI)[101]
	62. Rosenberg Self-Esteem Scale (RSES)[101]
	63. Profile of Mood States (POMS)[101]
	64. Neuropsychiatric Inventory (NPI)[66,77,102,80]
Neurological Conditions	65. Clinical Dementia Rating Scale (CDR)[86,80]
Functional ability and Mobility	66. Timed Up and Go (TUG)[68,76–78,84,88,89,96,61,]
	67. 6-minute Walk Test (6MWT)[63,71,74,79,95,60,69]
	68. Berg Balance Scale (BBS)[69,76,84,89,94]
	69. Gait Speed (electronic gait analysis system)[61,91,96,86]
	70. The Functional Reach Test[69,84,89]
	71. The Timed Chair Stand Test (CST)[94,61]
	72. The Sit-to-Stand Test (SST)[73,84]
	73. Single Task Walking[96]
	74. Short Physical Performance Battery[100]
	75. Disability Assessment of Dementia (DAD)[95]
	76. National Centre for Geriatrics and Gerontology-Functional Assessment Tool (NCGG-FAT)[58]
	77. Stopwatch (walking speed)[58]
	78. The Incremental Arm Exercise Test[72]
	79. John Hopkins Fall Risk Assessment Form (JHFRAF)[73]
	80. Tinetti Assessment Tool (TAT)[74]
	81. The Suttle Walk Test (SWT)[79]
	82. Short Physical Performance Battery (SPPB)[100,82]
	83. Functional Comorbidity Index[100]
	84. The Physical Performance Test (PPT)[74]
	85. Electromyography (EMG)[103]
	86. Intraindividual Variability (IIV)[97]
Physical Activity Level	87. The Physical Activities Scale for the Elderly (PASE)[100,82]
Strength	88. Grip Strength (handheld dynamometer)[58,89,61]
	89. The Arm Curl Test[89]
	90. HUMAC NORM Isokinetic Dynamometer[103]
	91. Muscle strength of knee extensors and elbow extensors[82]
Health Related and QoL	92. Carotid-stiffness Index[57]
	93. Carotid Intima-media Thickness (CIMT)[57]
	94. The Common Carotid Artery (CCA) Images[57]
	95. Cerebrovascular Hemodynamics in both steady-state and pulsatile components (duplex ultrasonography)[57]

		96. The Quality of Life-Alzheimer's Disease (QoL-AD)[66,70,71,85]
		97. 36-Item Short-Form Health Survey (SF-36)[78,101,74,94]
		98. The SF-12 Quality-of-life Questionnaire[69,94,80]
		99. The Quality of Life (QoL)[63]
		100. The Pittsburgh Sleep Quality Index[85,90]
		101. The Health-related Quality of Life for People with Dementia (DemQoL)[83,102]
		102. The Charlson Comorbidity Index (CCI)[65]
		103. The General Health Questionnaire (GHQ)[102]
		104. EuroQoL-5 Dimension (EQ-5D)[80]
	Self-dependency	105. Instrumental Activity of Daily Living Scale (IADL)[62,70]
		106. Activity of Daily Living Scale (ADL)[70,94,80]
		107. Lawton and Brody Instrumental Activities of Daily Living Scale[94,100]
		108. The Barthel Index of Activities of Daily Living (ADL)[65,74]
		109. Problems in Everyday Living Test (PELT)[94]
		110. The Physical Self-maintenance Scale (PSMS)[62]
		111. Edmonton Frail Scale (EFS)[73]
		112. Katz Index of Independence in Activities of Daily Living [77]
		113. Functional Activities Questionnaire (FAQ)[74]
	Cardiovascular Fitness	114. VO_2peak[57,83,92,95]
		115. VO_2max[79,90,96,84]
		116. The Modified Balke Maximal-graded Exercise Treadmill Test[92]
		117. Ergometric Test and Rest Electrocardiogram (ECG)[84]
	Biomarkers	118. Blood sample (serum insulin, leptin, neuroprotective growth factors (*e.g.,* BDNF, IGF-1, VEGF, and FGF-2), and cytokines (*e.g.,* TNF-α, IL-1β, IL-6, IL-8, and IL-15) levels)[104]
		119. Blood sample (plasma glucose and insulin sensitivity)[92]
		120. Blood sample (serum level of BDNF)[64,65,71,80]
		121. Blood sample (plasma MCP-1, and plasma IGF-1)[65]
		122. Blood sample (plasma lipids and cholesterol)[66]
		123. Blood samples (total cholesterol (TC), triglycerides (TG), high-density lipoprotein cholesterol (HDL-C), LDL-C, Plasma 8-iso-PGF2α Concentration and FBG and Serum Lipid Profiles)[72]
		124. Blood sample (TNF-αlevel)[101]
	Brain Imaging	125. Magnetic Resonance Imaging (MRI)[57,95,74,81]
		126. Electroencephalographic (EEG) Measurement[104,57,75]
		127. Resting-state Functional Magnetic Resonance Imaging (RS-fMRI)[60]
**Resistance Exercise**		

**(RE)**		
	Global Cognitive Function–Cognitive Assessment, Orientation	1. Mini-Mental State Exam (MMSE)[105–108]<br>2. The Alzheimer’s Disease Assessment Scale-cognitive (ADAS-Cog)[109,110]<br>3. Automated Neuropsychological Assessment Metrics (ANAM)[111,112]<br>4. Wechsler Adult Intelligence Scale 3rd Edition (WAIS-III)[109]<br>5. The Consortium to Establish a Registry for Alzheimer’s Disease (CERAD)[105,107]
	Global Cognitive Function–Executive Function	6. Trail Making Test-B (TMT-B)[105,110,113]<br>7. Frontal Lobe Battery (FAB-K)[103]<br>8. Frontal Assessment Battery (FAB)[105]<br>9. Montreal Cognitive Assessment (MoCA)[111,112]
	Global Cognitive Function–Memory	10. Digit Span Forward (DSF)[105,110,113,114]<br>11. The Ray 15-item Memory Test[105,114]<br>12. The Memory Search Test[111]<br>13. Spatial Span Forward[113]<br>14. Wechsler Memory Scale 3rd Edition (WMS-III)-auditory logical memory I (immediate) and II (delayed)[109]<br>15. Digit Span Backward (DSB)[105,110,113,114]<br>16. The Modified Boston Naming Test (BNT)[105]<br>17. Benton Visual Retention Test-Revised 5th Edition (BVRT-R)[109]
	Global Cognitive Function–Processing Speed	18. Trail Making Test-A (TMT-A)[105,113]<br>19. Symbol Digit Modalities Test (SDMT)[109]
	Global Cognitive Function–Attention	20. The Sustained Attention Index of the Conners Continuous Performance Test-II (CPT-II)[113]<br>21. The Stroop Color and Word Test (SCWT)[113,114]
	Global Cognitive Function–Language	22. The Ray Auditory Verbal Learning Test[113]<br>23. The Verbal Fluency Test (VF)[113]<br>24. Semantic Fluency (animals)[113]<br>25. The Controlled Oral Word Association Test (COWAT) and Animal Naming[114]<br>26. Verbal Fluency F-A-S Test (FAS)[107,113]
	Global Cognitive Function–Visuospatial Constructional Ability	27. The Visual Construction Test[105]
	Global Cognitive Function–Learning	28. The Word-List-Learning Test[105]
	Numerical Reasoning	29. Mathematical Processing Test[111]
	Decision-making	30. 2-Choice Reaction Time Test[111]<br>31. Reaction time Ruler or FETZ-test[107]
	Psychological Wellbeing	32. Geriatric Depression Scale (GDS)[115,108]<br>33. Center for Epidemiologic Studies Depression Scale (CES-D)[105]<br>34. Neuropsychiatric Inventory (NPI)[107]

		35. Profile of Mood States (POMS)[108]
	Neurological Conditions	36. Clinical Dementia Rating (CDR)[105]
	Functional Ability and Mobility	37. Timed Up and Go (TUG)[103,105,110,113,116]<br>38. The Short Physical Performance Battery (SPPB)[105,116]<br>39. The Functional Reach Test[110]<br>40. Berg Balance Scale (BBS)[113]<br>41. Talking-While-Walking Test[113]<br>42. The Activities-Specific Balance Confidence scale[110]<br>43. 4-Meter Fast Gait Speed test[105]<br>44. 4.44-m Gait Speed Test[103]<br>45. The back-scratch Test[106]<br>46. The Purdue Pegboard Test[115]
	Physical Activity Level	47. International Physical Activity Questionnaire (IPAQ)[105]
	Strength	48. Grip Strength (hand-to-hand dynamometer)[105,116]<br>49. Lower Limb Concentric Dynamic Strength (HUMAC NORM isokinetic dynamometer)[103,116]<br>50. Upper Body Strength and Flexibility[106]
	Health Related and QoL	51. The Senior Fitness Test (SFT)[108,114]<br>52. 36-Item Short-Form Health Survey (SF-36)[113]<br>53. Pittsburgh Sleep Quality Index (PSQI)[115]
	Self-dependency	54. Bayer Activities of Daily Living (B-ADL)[109]<br>55. Activities Daily of Living (ADL)[106,107]
	Response Inhibition	56. The Go/No-go Test[111]
	Brain Imaging	57. Magnetic Resonance Imaging (MRI)[113,112]<br>58. Electroencephalograms (EEG) Measurement[114]
	Biomarker	59. Blood sample (Serum IL-6, KYN, and IGF-1 concentrations)[111]
**Multicomponent Exercise (ME)**		
	Global Cognitive Function–Cognitive Assessment, Orientation	1. Mini-Mental State Exam (MMSE)[117–140,141,142,143,144]<br>2. Alzheimer's Disease Assessment Scale-Cognitive Subscale (ADAS-Cog)[130,135,138,145,146,147,146,148]<br>3. The Consortium to Establish a Registry for Alzheimer's Disease (CERAD)[125]<br>4. The Standardized Mini-Mental State Exam (SMMSE)[149]<br>5. Modified Mini-Mental State Examination (3MS)[150]<br>6. The Severe Impairment Battery-Short Form (SIB-S)[141]<br>7. Rapid Evaluation of Cognitive Function (ERFC)[151]<br>8. The 8 Words Test of the Amsterdam Dementia Screening Test (ADS)[152]<br>9. Raven's Matrices 1947 (RM47)[120]
	Global Cognitive Function–Executive	10. Montreal Cognitive Assessment (MoCA)[118,120,124,131,153–156,157,148,158,143]

Function	11. Trail Making Test-B (TMT-B)[120,125,130,154,159–161,148,143]<br>12. Frontal Assessment Battery (FAB)[120,126,130,141]<br>13. The Colour Trails Test (CTT)[145]<br>14. The Key Search Test of the Behavioral Assessment of the Dysexecutive Syndrome (BADS)[152]
Global Cognitive Function–Memory	15. Digit Span Forward (DSF)[127,141,145,152,159,161,158]<br>16. Digit Span Backward (DSB)[127,141,145,152,159,148,158,143]<br>17. The Face and Picture Recognition of the Rivermead Behavioral Memory Test (RBMT)[130,152]<br>18. Wechsler Memory Scale (WMS)[119,136]<br>19. Spatial Corsi's Block-tapping Test (CB)[120]<br>20. The Visual Memory Span Forward and Backward (VMSF and VMSB)[127]<br>21. The California Verbal Learning Test Second Edition (CVLT-II)[129,148,143]<br>22. The Rivermead Behavioral Memory Test[136]<br>23. The Boston Naming Test (BNT)[158]
Global Cognitive Function–Processing Speed	24. Trail Making Test-A (TMT-A)[117,120,125,127,130,136,154,159,148,143]<br>25. Symbol Digit Modalities Test (SDMT)[124,129,162]<br>26. Symbol Digit Substitution Test (SDST)[117]<br>27. The Digit Symbol-coding (SDC)[119,148]<br>28. Wechsler Adult Intelligence Scale (WAIS)[148]
Global Cognitive Function–Attention	29. The Stroop Color and Word Test (SCWT)[119,129,161,136,127,128]<br>30. Addenbrooke's Cognitive Examination (ACE)[121,159,163]<br>31. Digit Cancellation Test (DCT)[130]<br>32. The Attentive Matrices[120]<br>33. Stroop interference task[143]
Global Cognitive Function–Language	34. The Letter Verbal Fluency Test (LVFT)[119,128]<br>35. The Verbal fluency Test (animals and professions)[136,138,158]<br>36. Phonological Fluency (FAS)[120]<br>37. The Groninger Intelligence Test (GIT)[152,141,136]<br>38. The Phonemic Fluency Test[127,143]<br>39. The Assessment of Communication and Interaction Skills (ACIS)[164]<br>40. Phonemic and Semantic Verbal Fluency[148]<br>41. The Auditory Verbal Learning Test[158]
Global Cognitive Function–Learning	42. The Hauser Index[120]
Global Cognitive Function–Visuospatial constructional ability	43. The Clock Drawing Test (CDT)[128,135,165,158]<br>44. The Mental Rotation Test (MRT)[161]<br>45. The Brief Visuospatial Memory Test-Revised (BVMT-R)[129]
Psychological Wellbeing	46. Geriatric Depression Scale (GDS)[125,131,147,156,166,167,155,138,158]<br>47. Neuropsychiatric Inventory (NPI)[130,137,162,168]

		48. The Cornell Scale for Depression in dementia (CSDD)[133,137,141,157]
		49. The Apathy Evaluation Scale-Clinical version (AES-C)[166]
		50. 17-item Hamilton Depression Scale (HAMD-17)[124]
		51. UCLA 3-item Loneliness Scale[155]
		52. The Cohen-Mansfield Agitation Inventory (CMAI)[141]
		53. The Montgomery-As-berg Depression Rating Scale (MADRS)[168]
		54. Neuropsychiatric Inventory (NPI)[146,142]
		55. 16-item Self-report Assessment Tool[160]
		56. Hamilton Anxiety Scale[158]
		57. Hospital Anxiety and Depression Scale (HADS)[142]
	Neurological Conditions	58. The Clinical Dementia Rating (CDR)[128,133,137]
	Functional ability and Mobility	59. The Timed up-and-go (TUG)[117,125–127,129,133,141,147,154,156,162,164,166,167,169,170,136,163,168]
		60. 6-min Walking Test (6MWT)[118,127,129,130,136,140,168,170,171,141,148,158,142]
		61. The Short Physical Performance Battery (SPPB)[121,125,127,131,139,164,166]
		62. Gait Speed Test (The 400/10-m Walk Test) (tri-axial accelerometer)[117,162,166,167,172,169,129]
		63. (30-seocnd) The Chair Stand Test (CTS)[126,133,141,160,162,137,163,144]
		64. Berg Balance Scale (BBS)[135,137,150,156,160]
		65. 5-Times-Sit-to-Stand (FTSTS)[170,155,129,133,134]
		66. The Functional Test (FRT)[123,126,147,170]
		67. (30-second) Sit-to-Stand Test[147,156,136,137]
		68. Falls Efficacy Scale International (FES-I)[150,155,125]
		69. The Frailty and Injuries: Cooperative Studies of Intervention Techniques-4 scale (FICSIT-4)[127,141,136]
		70. Tinetti Assessment Tool (TAT)[173,174,158]
		71. 10-Meter Walk Test[129,154]
		72. Performance Oriented Motor Assessment (POMA)[125,169]
		73. Step Test[126,155,144]
		74. The Short Falls Efficacy Scale International (Short FES-I)[166]
		75. Functional Comorbidity Index-18 (FCI-18)[127]
		76. 2-minute Walk Test[155]
		77. 4-Meter Walk Test[155]
		78. The Hill Step Test[121]
		79. Activities-specific Balance Confidence (ABC) Scale[150]
		80. 8-ft Walk Test[160]
		81. The figure-of-8 test[136,141]
		82. The Mini-Physical Performance Test (PPT)[118]
		83. Upper Extremity Range of Motion (goniometer)[167]

		84. Six Spot Step Test (SSST)[129]<br>85. Gait performance (velocity, cadence, stride time, and stride length)[164]<br>86. The Frailty and Injuries Cooperative Studies of Intervention Techniques-Subtest[136]<br>87. The Groningen Meander Walking Test[136]<br>88. The One-leg Balance Test[168]<br>89. The Tandem Stance Group Test (static and dynamic balance)[129]<br>90. Peak and Mean Isometric Torque of the Knee Flexors and Extensors (Biodex isokinetic dynamometer)[133]<br>91. Stationary Balance (the pressure platform E.P.S.-R1)[134]<br>92. The Revised Elderly Disability Scale (REPDS)[165]<br>93. The Timed Chair Stand Test[141]<br>94. The University of Alabama Life Space Assessment (UAB LSA)[121]<br>95. The Fear of Falling Avoidance Behavior Questionnaire[166]<br>96. The Falls Risk Assessment Tool (FRAT)[155]<br>97. The Acute Care Index of Function (ACIF)[140]<br>98. Timed Static Pedaling (TSP)[170]<br>99. The NeuroCom Balance Master (long plate)[126]<br>100. Bessou locometer and SATEL software (Walking speed, Stride length, and Double limb support time)[151]<br>101. The Brief-balance Evaluation Systems Test[163]<br>102. Attitudes to Falls Related Interventional Scale (AFRIS)[125]<br>103. Test of Dual-task Performance[162]<br>104. Test of Exercise Self-efficacy (five-item questionnaire)[162]<br>105. The Sit and reach test[144]<br>106. Modified push-up test[144]<br>107. The Romberg test[144]
	Physical Activity Level	108. The Pool Activity level (PAL)[155]<br>109. Physical Activity Questionnaire for the Elderly (PAQE)[125]<br>110. The Adjusted Activity Scare (AAS)[126]<br>111. The Brief Physical Activity Assessment Tool[163]
	Strength	112. Hand Grip Strength (HGS) (hand dynamometer, Jamar hydraulic hand dynamometer)[117,129,133,135,141,154,125]<br>113. The Arm Curl Test (ACT)[156]<br>114. Maximal Knee Extension Strength (dynamometer)[136]<br>115. The Muscle Research Council and The digital hand dynamometer (Muscle strength: abductors of the humerus, flexors of the forearm, extensors of the wrist, hip flexors, knee flexors, and dorsiflexors of the ankle; upper extremity muscle strength)[167]

	116. Muscle strength (leg extension, leg flexion, and plantar flexion)[125]<br>117. The upper and lower limb and respiratory muscle strength (Baseline Hydraulic Hand Dynamometer and a respiratory pressure gauge)[163]
Health Related and QoL	118. Quality of Life in Alzheimer's Disease (QoL-AD)[132,146,150,155,148,142]<br>119. Short-Form 12 Health Survey (SF-12)[125,134]<br>120. EuroQoL-5 Dimension (EQ-5D)[131,155,148]<br>121. Short From 36 Health Survey (SF-36)[138,145]<br>122. Senior Fitness Battery (SFB)[173,174]<br>123. The Pittsburgh Sleep Quality Index[149,158,142]<br>124. Cumulative Illness Rating Scale (CIRS)[125]<br>125. The Assessment of Quality of Life[126]<br>126. The Visual Analog Scale (VAS)[155]<br>127. Quality of Life Instrument for Dementia[164]<br>128. Alzheimer's Disease Related Quality of Life (ADRQL)[156]<br>129. The Quality of Life in Late-stage Dementia Scale (QUALID)[137]
Self-dependency	130. Katz Index of Independence in Activities of Daily Living[125,131,150,156,168,173,174,124,148]<br>131. The Barthel Index of Activities of Daily Living (ADL)[131,137,147,173]<br>132. Lawton and Brody Instrumental Activities of Daily Living Scale[128,163,174]<br>133. The Activities of Daily Living Questionnaire (ADL-Q)[133,125]<br>134. The Instrumental Activities of Daily Living (IADL)[130,159]<br>135. The Siu and Reuben Physical Scale for Advanced Activities of Daily Living (AAVD)[174]<br>136. The Bayer Activities of Daily Living Scale (B-ADL)[164]<br>137. The Direct Assessment of Functional Status (DAFS-R)[133]<br>138. The Grocery Shelving Task[163]<br>139. The Bristol Activity of Daily Living Index[146]<br>140. The Alzheimer's Disease Cooperative Study Activities of Daily Living (ADCS-ADL)[132,139]
Cardiovascular Fitness	141. The 6-min Astrand Cycle Ergometer Test[162]<br>142. VO_2max[128,142]
Response Inhibition	143. The Go/No-go Test[141]
Brain Imaging	144. Electroencephalographic (EEG) Measurement[117]<br>145. MRI[148,143]
Biomarkers	146. Blood sample (Total Cholesterol (TC), high-density lipoprotein cholesterol (HDL-C), triglyceride (TG), and glycosylated hemoglobin (HbA1c))[171]<br>147. Blood sample (fasting glucose (GL), triglycerides (TGC), total cholesterol (TC), non-HDL cholesterol, HDL cholesterol, and LDL cholesterol; BDNF)[122]<br>148. Blood sample (Brain-derived neurotrophic factor (BDNF)[124]<br>149. Blood sample (fasting blood glucose (FBG), insulin, homeostasis model assessment of

	insulin resistance (HOMA-IR), hemoglobin A1c (HbA1c))[129]
	150. Blood sample (Glucose, Total Cholesterol, High-Density Lipoprotein-HDL, Low-Density Lipoprotein-LDL, Triglycerides)[130]
Gene Identification	151. APOE genotyping[127]

Notes: Most instruments include multidimensional assessments. We categorized them according to their primary focus and the definitions provided by the authors of the included studies.

Abbreviations:
BDNF: Plasma Brain-derived Neurotrophic Factor
APOE: The apolipoprotein E

References

1. Bisbe M, Fuente-Vidal A, López E, et al. Comparative Cognitive Effects of Choreographed Exercise and Multimodal Physical Therapy in Older Adults with Amnestic Mild Cognitive Impairment: Randomized Clinical Trial. *Journal of Alzheimer's Disease*. 2020;73(2):769-783. doi:10.3233/JAD-190552
2. Qi M, Zhu Y, Zhang L, Wu T, Wang J. The effect of aerobic dance intervention on brain spontaneous activity in older adults with mild cognitive impairment: A resting-state functional MRI study. *Exp Ther Med*. Published online 2018:715-722. doi:10.3892/etm.2018.7006
3. Lazarou I, Parastatidis T, Tsolaki A, et al. International Ballroom Dancing Against Neurodegeneration: A Randomized Controlled Trial in Greek Community-Dwelling Elders With Mild Cognitive impairment. *Am J Alzheimers Dis Other Demen*. 2017;32(8):489-499. doi:10.1177/1533317517725813
4. Franco MR, Sherrington C, Tiedemann A, et al. Effect of Senior Dance (DanSE) on Fall Risk Factors in Older Adults: A Randomized Controlled Trial. *Phys Ther*. 2020;100(4):600-608. doi:10.1093/ptj/pzz187
5. Doi T, Verghese J, Makizako H, et al. Effects of Cognitive Leisure Activity on Cognition in Mild Cognitive Impairment: Results of a Randomized Controlled Trial. *J Am Med Dir Assoc*. 2017;18(8):686-691. doi:10.1016/j.jamda.2017.02.013
6. Van de Winckel A, Feys H, De Weerdt W, Dom R. Cognitive and behavioural effects of music-based exercises in patients with dementia. *Clin Rehabil*. 2004;18(3):253-260. doi:10.1191/0269215504cr750oa
7. Bracco L, Pinto-Carral A, Hillaert L, Mourey F. Tango-therapy vs physical exercise in older people with dementia; a randomized controlled trial. *BMC Geriatr*. 2023;23(1):1-13. doi:10.1186/s12877-023-04342-x
8. Zhu Y, Gao Y, Guo C, et al. Effect of 3-Month Aerobic Dance on Hippocampal Volume and Cognition in Elderly People With Amnestic Mild Cognitive Impairment: A Randomized Controlled Trial. *Front Aging Neurosci*. 2022;14(March):1-10. doi:10.3389/fnagi.2022.771413
9. Sánchez-Alcalá M, Aibar-Almazán A, Hita-Contreras F, et al. Effects of Dance-Based Aerobic Training on Mental Health and Quality of Life in Older Adults with Mild Cognitive Impairment. *J Pers Med*. 2024;14(8):1-16. doi:10.3390/jpm14080844
10. Esmail A, Vrinceanu T, Lussier M, et al. Effects of Dance/Movement Training vs. Aerobic Exercise Training on cognition, physical fitness and quality of life in older adults: A randomized controlled trial. *J Bodyw Mov Ther*. 2020;24(1):212-220. doi:10.1016/j.jbmt.2019.05.004
11. Kropacova S, Mitterova K, Klobusiakova P, et al. Cognitive effects of dance-movement intervention in a mixed group of seniors are not dependent on hippocampal atrophy. *J Neural Transm*. 2019;126(11):1455-1463. doi:10.1007/s00702-019-02068-y
12. Zhu Y, Wu H, Qi M, et al. Effects of a specially designed aerobic dance routine on mild cognitive impairment. *Clin Interv Aging*. 2018;13:1691-1700. doi:10.2147/CIA.S163067

3. Song D, Yu D, Liu T, Wang J. Effect of an Aerobic Dancing Program on Sleep Quality for Older Adults With Mild Cognitive Impairment and Poor Sleep: A Randomized Controlled Trial. *J Am Med Dir Assoc*. 2024;25(3):494-499. doi:10.1016/j.jamda.2023.09.020
4. Chang J, Zhu W, Zhang J, et al. The Effect of Chinese Square Dance Exercise on Cognitive Function in Older Women With Mild Cognitive Impairment: The Mediating Effect of Mood Status and Quality of Life. *Front Psychiatry*. 2021;12(July). doi:10.3389/fpsyt.2021.711079
5. Ho RTH, Fong TCT, Chan WC, et al. Psychophysiological Effects of Dance Movement Therapy and Physical Exercise on Older Adults with Mild Dementia: A Randomized Controlled Trial. *Journals of Gerontology - Series B Psychological Sciences and Social Sciences*. 2018;75(3):560-570. doi:10.1093/geronb/gby145
6. Blumen HM, Ayers E, Wang C, Ambrose AF, Jayakody O, Verghese J. Randomized Controlled Trial of Social Ballroom Dancing and Treadmill Walking: Preliminary Findings on Executive Function and Neuroplasticity From Dementia-at-Risk Older Adults. *J Aging Phys Act*. 2023;31(4):589-599. doi:10.1123/japa.2022-0176
7. Thiel U, Stiebler M, Labott BK, et al. DiADEM—Dance against Dementia—Effect of a Six-Month Dance Intervention on Physical Fitness in Older Adults with Mild Cognitive Impairment: A Randomized, Controlled Trial. *J Pers Med*. 2024;14(8). doi:10.3390/jpm14080888
8. Padala KP, Padala PR, Malloy TR, et al. Wii-fit for improving gait and balance in an assisted living facility: A pilot study. *J Aging Res*. 2012;2012:6-11. doi:10.1155/2012/597573
9. van Santen J, Dröes RM, Twisk JWR, Blanson Henkemans OA, van Straten A, Meiland FJM. Effects of Exergaming on Cognitive and Social Functioning of People with Dementia: A Randomized Controlled Trial. *J Am Med Dir Assoc*. 2020;21(12):1958-1967.e5. doi:10.1016/j.jamda.2020.04.018
20. Zheng J, Yu P, Chen X. An Evaluation of the Effects of Active Game Play on Cognition, Quality of Life and Depression for Older People with Dementia. *Clin Gerontol*. 2022;45(4):1034-1043. doi:10.1080/07317115.2021.1980170
21. Uğur F, Sertel M. Wii Fit Exercise's Effects on Muscle Strength and Fear of Falling in Older Adults With Alzheimer Disease: A Randomized Controlled Trial. *J Aging Phys Act*. 2025;33(2):181-191. doi:10.1123/japa.2023-0428
22. Eggenberger P, Schumacher V, Angst M, Theill N, de Bruin ED. Does multicomponent physical exercise with simultaneous cognitive training boost cognitive performance in older adults? A 6-month randomized controlled trial with a 1-year follow-up. *Clin Interv Aging*. 2015;10:1335-1349. doi:10.2147/CIA.S87732
23. Sabbagh et al. Sensor-based balance training with motion feedback in people with mild cognitive impairment. 2016;53(6):945-958. doi:10.1682/JRRD.2015.05.0089.Sensor-based
24. Liu CL, Cheng FY, Wei MJ, Liao YY. Effects of Exergaming-Based Tai Chi on Cognitive Function and Dual-Task Gait Performance in Older Adults With Mild Cognitive Impairment: A Randomized Control Trial. *Front Aging Neurosci*. 2022;14(March). doi:10.3389/fnagi.2022.761053
25. Liao YY, Chen IH, Hsu WC, Tseng HY, Wang RY. Effect of exergaming versus combined exercise on cognitive function and brain activation in frail older adults: A randomised controlled trial. *Ann Phys Rehabil Med*. 2021;64(5):101492. doi:10.1016/j.rehab.2021.101492
26. Karssemeijer EGA, Aaronson JA, Bossers WJR, Donders R, Olde Rikkert MGM, Kessels RPC. The quest for synergy between physical exercise and cognitive stimulation via exergaming in people with dementia: A randomized controlled trial. *Alzheimers Res Ther*. 2019;11(1):1-13. doi:10.1186/s13195-018-0454-z
27. Swinnen N, Vandenbulcke M, de Bruin ED, et al. The efficacy of exergaming in people with major neurocognitive disorder residing in long-term care facilities: a pilot randomized controlled trial. *Alzheimers Res Ther*. 2021;13(1):1-13. doi:10.1186/s13195-021-00806-7
28. Wu S, Ji H, Won J, Jo EA, Kim YS, Park JJ. The Effects of Exergaming on Executive and Physical Functions in Older Adults With Dementia: Randomized Controlled Trial. *J Med Internet Res*. 2023;25:1-17. doi:10.2196/39993
29. Hughes TF, Flatt JD, Fu B, Butters MA, Chang CCH, Ganguli M. Interactive video gaming compared with health education in older adults with mild cognitive impairment: A feasibility study. *Int J Geriatr Psychiatry*. 2014;29(9):890-898. doi:10.1002/gps.4075
30. Khanthong P, Sriyakul K, Dechakhamphu A, Krajarng A, Kamalashiran C, Tungsukruthai P. Traditional Thai exercise (Ruesi Dadton) for improving motor and cognitive functions in mild cognitive impairment: a randomized controlled trial. *J Exerc Rehabil*. 2021;17(5):331-338. doi:10.12965/JER.2142542.271
31. Kashyap M, Rai NK, Singh R, et al. Effect of Early Yoga Practice on Post Stroke Cognitive Impairment. 2022;22(4):2019. doi:10.4103/aian.AIAN

[illegible]2. Tremont G, Davis J, Ott BR, et al. Feasibility of a Yoga Intervention for Individuals with Mild Cognitive Impairment: A Randomized Controlled Trial. *Journal of Integrative and Complementary Medicine*. 2022;28(3):250-260. doi:10.1089/jicm.2021.0204

[illegible]3. Grzenda A, Siddarth P, Milillo MM, Aguilar-Faustino Y, Khalsa DS, Lavretsky H. Cognitive and immunological effects of yoga compared to memory training in older women at risk for alzheimer's disease. *Transl Psychiatry*. 2024;14(1):1-11. doi:10.1038/s41398-024-02807-0

[illegible]4. Eyre HA, Siddarth P, Acevedo B, et al. A randomized controlled trial of Kundalini yoga in mild cognitive impairment. *Int Psychogeriatr*. 2017;29(4):557-567. doi:10.1017/S1041610216002155

[illegible]5. Lam LCW, Chau RCM, Wong BML, et al. A 1-Year Randomized Controlled Trial Comparing Mind Body Exercise (Tai Chi) With Stretching and Toning Exercise on Cognitive Function in Older Chinese Adults at Risk of Cognitive Decline. *J Am Med Dir Assoc*. 2012;13(6):568.e15-568.e20. doi:10.1016/j.jamda.2012.03.008

[illegible]6. Jiayuan Z, Xiang-Zi J, Li-Na M, Jin-Wei Y, Xue Y. Effects of Mindfulness-Based Tai Chi Chuan on Physical Performance and Cognitive Function among Cognitive Frailty Older Adults: A Six-Month Follow-Up of a Randomized Controlled Trial. *Journal of Prevention of Alzheimer's Disease*. 2022;9(1):104-112. doi:10.14283/jpad.2021.40

[illegible]7. Huang N, Li W, Rong X, et al. Effects of a Modified Tai Chi Program on Older People with Mild Dementia: A Randomized Controlled Trial. *Journal of Alzheimer's Disease*. 2019;72(3):947-956. doi:10.3233/JAD-190487

[illegible]8. Cheng ST, Chow PK, Song YQ, et al. Mental and physical activities delay cognitive decline in older persons with dementia. *American Journal of Geriatric Psychiatry*. 2014;22(1):63-74. doi:10.1016/j.jagp.2013.01.060

[illegible]9. Chan AWK, Yu DSF, Choi KC, Lee DTF, Sit JWH, Chan HYL. Tai chi qigong as a means to improve night-time sleep quality among older adults with cognitive impairment: A pilot randomized controlled trial. *Clin Interv Aging*. 2016;11:1277-1286. doi:10.2147/CIA.S111927

[illegible]0. Li K, Yu H, Kortas JA, Lin X, Lipowski M. The effect of 12 weeks of Baduanjin exercise on cognitive function, lower limb balance and quality of life of the elderly with mild cognitive impairment: a randomized controlled trial. *Gazzetta Medica Italiana Archivio per le Scienze Mediche*. 2022;181(11):811-823. doi:10.23736/S0393-3660.22.04802-1

[illegible]1. Hsu CY, Yeh ML, Liu YCE. Three-month Chan-Chuang qigong program improves physical performance and quality of life of patients with cognitive impairment: A randomized controlled trial. *Res Nurs Health*. 2022;45(3):327-336. doi:10.1002/nur.22219

[illegible]2. Li F, Harmer P, Fitzgerald K, Winters-Stone K. A cognitively enhanced online Tai Ji Quan training intervention for community-dwelling older adults with mild cognitive impairment: A feasibility trial. *BMC Geriatr*. 2022;22(1):1-13. doi:10.1186/s12877-021-02747-0

[illegible]3. Chen Y, Qin J, Tao L, et al. Effects of Tai Chi Chuan on Cognitive Function in Adults 60 Years or Older With Type 2 Diabetes and Mild Cognitive Impairment in China: A Randomized Clinical Trial. *JAMA Netw Open*. 2023;6(4):E237004. doi:10.1001/jamanetworkopen.2023.7004

[illegible]4. Lin M, Liu W, Ma C, et al. Tai Chi-Induced Exosomal LRP1 is Associated With Memory Function and Hippocampus Plasticity in aMCI Patients. *American Journal of Geriatric Psychiatry*. 2024;32(10):1215-1230. doi:10.1016/j.jagp.2024.04.012

[illegible]5. Yu AP, Chin EC, Yu DJ, et al. Tai Chi versus conventional exercise for improving cognitive function in older adults: a pilot randomized controlled trial. *Sci Rep*. 2022;12(1):1-15. doi:10.1038/s41598-022-12526-5

[illegible]6. Su H, Wang H, Meng L, Bush E. The effects of Baduanjin exercise on the subjective memory complaint of older adults: A randomized controlled trial. *Medicine (United States)*. 2021;100(30):E25442. doi:10.1097/MD.0000000000025442

[illegible]7. Zheng G, Ye B, Xia R, et al. Traditional Chinese Mind-Body Exercise Baduanjin Modulate Gray Matter and Cognitive Function in Older Adults with Mild Cognitive Impairment: A Brain Imaging Study. *Brain Plasticity*. 2021;7(2):131-142. doi:10.3233/bpl-210121

[illegible]8. Zheng G, Zheng Y, Xiong Z, Ye B. Effect of Baduanjin exercise on cognitive function in patients with post-stroke cognitive impairment: a randomized controlled trial. *Clin Rehabil*. 2020;34(8):1028-1039. doi:10.1177/0269215520930256

[illegible]9. Sungkarat S, Boripuntakul S, Kumfu S, Lord SR, Chattipakorn N. Tai Chi Improves Cognition and Plasma BDNF in Older Adults With Mild Cognitive Impairment: A

Randomized Controlled Trial. *Neurorehabil Neural Repair*. 2018;32(2):142-149. doi:10.1177/1545968317753682

[illegible]0. Luo SS, Chen L, Wang GB, Wang YG, Su XY. Effects of long-term Wuqinxi exercise on working memory in older adults with mild cognitive impairment. *Eur Geriatr Med*. 2022;13(6):1327-1333. doi:10.1007/s41999-022-00709-2

[illegible]1. Li F, Harmer P, Voit J, Chou LS. Implementing an online virtual falls prevention intervention during a public health pandemic for older adults with mild cognitive impairment: A feasibility trial. *Clin Interv Aging*. 2021;16:973-983. doi:10.2147/CIA.S306431

[illegible]2. Liu JYW, Kwan RYC, Lai CKY, Hill KD. A simplified 10-step Tai-chi programme to enable people with dementia to improve their motor performance: a feasibility study. *Clin Rehabil*. 2018;32(12):1609-1623. doi:10.1177/0269215518786530

[illegible]3. Nyman SR, Ingram W, Sanders J, et al. Randomised controlled trial of the effect of tai chi on postural balance of people with dementia. *Clin Interv Aging*. 2019;14:2017-2029. doi:10.2147/CIA.S228931

[illegible]4. Gao R, Greiner C, Ryuno H, Zhang X. Effects of Tai Chi on physical performance, sleep, and quality of life in older adults with mild to moderate cognitive impairment. *BMC Complement Med Ther*. 2024;24(1). doi:10.1186/s12906-024-04705-w

[illegible]5. Canan Okuyan ED. The effectiveness of Tai Chi Chuan on fear of movement, prevention of falls, physical activity, and cognitive status in older adults with mild cognitive impairment: A randomized controlled trial. *Perspect Psychiatr Care*. 2021;57(3):1273-1281. doi:10.1111/ppc.12684

[illegible]6. Chang CL, Lin TK, Pan CY, et al. Distinct effects of long-term Tai Chi Chuan and aerobic exercise interventions on motor and neurocognitive performance in early-stage Parkinson's disease: a randomized controlled trial. *Eur J Phys Rehabil Med*. 2024;60(4):621-633. doi:10.23736/S1973-9087.24.08166-8

[illegible]7. Tomoto T, Liu J, Tseng BY, et al. One-Year Aerobic Exercise Reduced Carotid Arterial Stiffness and Increased Cerebral Blood Flow in Amnestic Mild Cognitive Impairment. *Journal of Alzheimer's Disease*. 2021;80(2):841-853. doi:10.3233/JAD-201456

[illegible]8. Shimada H, Lee S, Akishita M, et al. Effects of golf training on cognition in older adults: A randomised controlled trial. *J Epidemiol Community Health (1978)*. 2018;72(10):944-950. doi:10.1136/jech-2017-210052

[illegible]9. Ihle-Hansen H, Langhammer B, Lydersen S, Gunnes M, Indredavik B, Askim T. A physical activity intervention to prevent cognitive decline after stroke: Secondary results from the life after stroke study, an 18-month randomized controlled trial. *J Rehabil Med*. 2019;51(9):646-651. doi:10.2340/16501977-2588

[illegible]0. Hsu CL, Best JR, Davis JC, et al. Aerobic exercise promotes executive functions and impacts functional neural activity among older adults with vascular cognitive impairment. *Br J Sports Med*. 2018;52(3):184-191. doi:10.1136/bjsports-2016-096846

[illegible]1. Makino T, Umegaki H, Ando M, et al. Effects of Aerobic, Resistance, or Combined Exercise Training among Older Adults with Subjective Memory Complaints: A Randomized Controlled Trial. *Journal of Alzheimer's Disease*. 2021;82(2):701-717. doi:10.3233/JAD-210047

[illegible]2. Wei X hong, Ji L li. Effect of handball training on cognitive ability in elderly with mild cognitive impairment. *Neurosci Lett*. 2014;566:98-101. doi:10.1016/j.neulet.2014.02.035

[illegible]3. Nakatsuka M, Nakamura K, Hamanosono R, et al. A Cluster Randomized Controlled Trial of Nonpharmacological Interventions for Old-Old Subjects with a Clinical Dementia Rating of 0.5: The Kurihara Project. *Dement Geriatr Cogn Dis Extra*. 2015;5(2):221-232. doi:10.1159/000380816

[illegible]4. Kohanpour MA, Peeri M, Azarbayjani MA. The effects of aerobic exercise with lavender essence use on cognitive state and serum brain-derived neurotrophic factor levels in elderly with mild cognitive impairment. *Journal of HerbMed Pharmacology*. 2017;6(2):80-84.

[illegible]5. Liu IT, Lee WJ, Lin SY, Chang ST, Kao CL, Cheng YY. Therapeutic Effects of Exercise Training on Elderly Patients With Dementia: A Randomized Controlled Trial. *Arch Phys Med Rehabil*. 2020;101(5):762-769. doi:10.1016/j.apmr.2020.01.012

[illegible]6. Yang SY, Shan CL, Qing H, et al. The Effects of Aerobic Exercise on Cognitive Function of Alzheimer's Disease Patients. *CNS Neurol Disord Drug Targets*. 2015;14(10):1292-1297. doi:10.2174/1871527315666151111123319

[illegible]7. Khattak HG, Ahmad Z, Arshad H, Anwar K. Effect of aerobic exercise on cognition in elderly persons with mild cognitive impairment. *Rawal Medical Journal*. 2022;47(3):698-701. doi:10.5455/rmj.20210713072242

68. Varela S, Ayán C, Cancela JM, Martín V. Effects of two different intensities of aerobic exercise on elderly people with mild cognitive impairment: A randomized pilot study. *Clin Rehabil*. 2012;26(5):442-450. doi:10.1177/0269215511425835

69. Miu D, Edin F, Szeto S, Mak Y. A randomised controlled trial on the effect of exercise on physical, cognitive and affective function in dementia subjects. *Asian Journal of Gerontology & Geriatrics*. 2008;3(1):8-16.

70. Angiolillo A, Leccese D, Ciccotelli S, et al. Effects of Nordic walking in Alzheimer's disease: A single-blind randomized controlled clinical trial. *Heliyon*. 2023;9(5):e15865. doi:10.1016/j.heliyon.2023.e15865

71. Enette L, Vogel T, Merle S, et al. Effect of 9 weeks continuous vs. interval aerobic training on plasma BDNF levels, aerobic fitness, cognitive capacity and quality of life among seniors with mild to moderate Alzheimer's disease: A randomized controlled trial. *European Review of Aging and Physical Activity*. 2020;17(1):1-16. doi:10.1186/s11556-019-0234-1

72. Phoemsapthawee et al. The Benefit of Arm Swing Exercise on Cognitive Performance in Older Women with Mild Cognitive Impairment. *Journal of Exercise Physiology*. 2016;8(1):11-25.

73. Guzel I, Can F. The effects of different exercise types on cognitive and physical functions in dementia patients: A randomized comparative study. *Arch Gerontol Geriatr*. 2024;119(18):105321. doi:10.1016/j.archger.2023.105321

74. Venturelli M, Scarsini R, Schena F. Six-month walking program changes cognitive and ADL performance in patients with Alzheimer. *Am J Alzheimers Dis Other Demen*. 2011;26(5):381-388. doi:10.1177/1533317511418956

75. Amjad I, Toor H, Niazi IK, et al. Therapeutic effects of aerobic exercise on EEG parameters and higher cognitive functions in mild cognitive impairment patients. *International Journal of Neuroscience*. 2019;129(6):551-562. doi:10.1080/00207454.2018.1551894

76. Abbas RL, Saab IM, Al-Sharif HK, Naja N, El-Khatib A. Effect of Adding Motorized Cycle Ergometer Over Exercise Training on Balance in Older Adults with Dementia: A Randomized Controlled Trial. *Exp Aging Res*. 2023;49(2):100-111. doi:10.1080/0361073X.2022.2046947

77. Cancela JM, Ayán C, Varela S, Seijo M. Effects of a long-term aerobic exercise intervention on institutionalized patients with dementia. *J Sci Med Sport*. 2016;19(4):293-298. doi:10.1016/j.jsams.2015.05.007

78. Dillon K, Prapavessis H. REducing SEDENTary behavior among mild to moderate cognitively impaired assisted living residents: A pilot randomized controlled trial (RESEDENT study). *J Aging Phys Act*. 2021;29(1):27-35. doi:10.1123/JAPA.2019-0440

79. Yu F, Salisbury D, Mathiason MA. Inter-individual differences in the responses to aerobic exercise in Alzheimer's disease: Findings from the FIT-AD trial. *J Sport Health Sci*. 2021;10(1):65-72. doi:10.1016/j.jshs.2020.05.007

80. Baker LD, Pa JA, Katula JA, et al. Effects of exercise on cognition and Alzheimer's biomarkers in a randomized controlled trial of adults with mild cognitive impairment: The EXERT study. *Alzheimer's and Dementia*. 2025;21(4):1-17. doi:10.1002/alz.14586

81. Shadyab AH, Aslanyan V, Jacobs DM, et al. Effects of exercise versus usual care on older adults with amnestic mild cognitive impairment: EXERT versus ADNI. *Alzheimer's and Dementia*. 2025;21(4):1-14. doi:10.1002/alz.70118

82. Huang X, Zhang S, Zhao X, et al. Feasibility and effects of remotely supervised aerobic training and resistance training in older adults with mild cognitive impairment: A pilot three-arm randomised controlled trial. *Gen Psychiatr*. 2025;38(2). doi:10.1136/gpsych-2024-101858

83. Stuckenschneider T, Sanders ML, Devenney KE, et al. NeuroExercise: The Effect of a 12-Month Exercise Intervention on Cognition in Mild Cognitive Impairment—A Multicenter Randomized Controlled Trial. *Front Aging Neurosci*. 2021;12(January):1-12. doi:10.3389/fnagi.2020.621947

84. Arcoverde C, Deslandes A, Moraes H, et al. Treadmill training as an augmentation treatment for Alzheimer's disease: A pilot randomized controlled study. *Arq Neuropsiquiatr*. 2014;72(3):190-196. doi:10.1590/0004-282X20130231

85. Song D, Yu DSF. Effects of a moderate-intensity aerobic exercise programme on the cognitive function and quality of life of community-dwelling elderly people with mild cognitive impairment: A randomised controlled trial. *Int J Nurs Stud*. 2019;93:97-105. doi:10.1016/j.ijnurstu.2019.02.019

[illegible]6. Rojasavastera R, Bovonsunthonchai S, Hiengkaew V, Senanarong V. Action observation combined with gait training to improve gait and cognition in elderly with mild cognitive impairment a randomized controlled trial. *Dementia e Neuropsychologia*. 2020;14(2):118-127. doi:10.1590/1980-57642020dn14-020004

[illegible]7. Karthikeyan T. Therapeutic effects of home-based exercise of geriatrics for the management of cognitive impairment. *ES J Public Health*. 2020;1(1):1003. www.escientificlibrary.com

[illegible]8. Krootnark K, Chaikeeree N, Saengsirisuwan V, Boonsinsukh R. Effects of low-intensity home-based exercise on cognition in older persons with mild cognitive impairment: a direct comparison of aerobic versus resistance exercises using a randomized controlled trial design. *Front Med (Lausanne)*. 2024;11(June):1-11. doi:10.3389/fmed.2024.1392429

[illegible]9. Choi W, Lee S. Ground kayak paddling exercise improves postural balance, muscle performance, and cognitive function in older adults with mild cognitive impairment: A randomized controlled trial. *Medical Science Monitor*. 2018;24:3909-3915. doi:10.12659/MSM.908248

[illegible]0. Yu DJ, Yu AP, Bernal JDK, et al. Effects of exercise intensity and frequency on improving cognitive performance in middle-aged and older adults with mild cognitive impairment: A pilot randomized controlled trial on the minimum physical activity recommendation from WHO. *Front Physiol*. 2022;13(September):1-12. doi:10.3389/fphys.2022.1021428

[illegible]1. Fischbacher M, Chocano-Bedoya PO, Meyer U, et al. Safety and feasibility of a Dalcroze eurhythmics and a simple home exercise program among older adults with mild cognitive impairment (MCI) or mild dementia: The MOVE for your MIND pilot trial. *Pilot Feasibility Stud*. 2020;6(1):1-8. doi:10.1186/s40814-020-00645-7

[illegible]2. Baker LD, Frank LL, Foster-Schubert K, et al. Effects of aerobic exercise on mild cognitive impairment: A controlled trial. *Arch Neurol*. 2010;67(1):71-79. doi:10.1001/archneurol.2009.307

[illegible]3. Scherder EJA, Van Paasschen J, Deijen JB, et al. Physical activity and executive functions in the elderly with mild cognitive impairment. *Aging Ment Health*. 2005;9(3):272-280. doi:10.1080/13607860500089930

[illegible]4. L.F. Law et al. Effects of functional task exercise on everyday problem-solving ability and functional status in older adults with mild cognitive impairment—a randomised controlled trial. *Age Ageing*. 2021;51(7):1-11. doi:10.1093/ageing/afac144

[illegible]5. Morris JK, Vidoni ED, Johnson DK, et al. Aerobic exercise for Alzheimer's disease: A randomized controlled pilot trial. *PLoS One*. 2017;12(2):1-14. doi:10.1371/journal.pone.0170547

[illegible]6. Donnezan et al. Effects of simultaneous aerobic and cognitive training on executive functions, cardiovascular fitness and functional abilities in older adults with mild cognitive impairment. *Ment Health Phys Act*. 2018;15(April):78-87. doi:10.1016/j.mhpa.2018.06.001

[illegible]7. Brydges CR, Liu-Ambrose T, Bielak AAM. Using intraindividual variability as an indicator of cognitive improvement in a physical exercise intervention of older women with mild cognitive impairment. *Neuropsychology*. 2020;34(8):825-834. doi:10.1037/neu0000638

[illegible]8. Damirchi A, Hosseini F, Babaei P. Mental Training Enhances Cognitive Function and BDNF More Than Either Physical or Combined Training in Elderly Women With MCI: A Small-Scale Study. *Am J Alzheimers Dis Other Demen*. 2018;33(1):20-29. doi:10.1177/1533317517727068

[illegible]9. Eggermont LHP, Swaab DF, Hol EM, Scherder EJA. Walking the line: A randomised trial on the effects of a short term walking programme on cognition in dementia. *J Neurol Neurosurg Psychiatry*. 2009;80(7):802-804. doi:10.1136/jnnp.2008.158444

[illegible]00. Nagamatsu LS, Chan A, Davis JC, et al. Physical activity improves verbal and spatial memory in older adults with probable mild cognitive impairment: A 6-month randomized controlled trial. *J Aging Res*. 2013;2013(Mci). doi:10.1155/2013/861893

[illegible]01. Abd El-Kader SM, Al-Jiffri OH. Aerobic exercise improves quality of life, psychological well-being and systemic inflammation in subjects with alzheimer's disease. *Afr Health Sci*. 2016;16(4):1045-1055. doi:10.4314/ahs.v16i4.22

[illegible]02. Lowery D, Cerga-Pashoja A, Iliffe S, et al. The effect of exercise on behavioural and psychological symptoms of dementia: The EVIDEM-E randomised controlled clinical trial. *Int J Geriatr Psychiatry*. 2014;29(8):819-827. doi:10.1002/gps.4062

[illegible]03. Lee DW, Yoon DH, Lee JY, Panday SB, Park J, Song W. Effects of High-Speed Power Training on Neuromuscular and Gait Functions in Frail Elderly with Mild

Cognitive Impairment Despite Blunted Executive Functions: A Randomized Controlled Trial. *Journal of Frailty and Aging*. 2020;9(3):179-184. doi:10.14283/jfa.2020.23

04. Tsai CL, Pai MC, Ukropec J, Ukropcová B. Distinctive Effects of Aerobic and Resistance Exercise Modes on Neurocognitive and Biochemical Changes in Individuals with Mild Cognitive Impairment. *Curr Alzheimer Res*. 2019;16(4):316-332. doi:10.2174/1567205016666190228125429
05. Yoon DH, Lee JY, Song W. Effects of Resistance Exercise Training on Cognitive Function and Physical Performance in Cognitive Frailty: A Randomized Controlled Trial. *Journal of Nutrition, Health and Aging*. 2018;22(8):944-951. doi:10.1007/s12603-018-1090-9
06. Venturelli M, Lanza M, Muti E, Schena F. Positive effects of physical training in activity of daily living-dependent older adults. *Exp Aging Res*. 2010;36(2):190-205. doi:10.1080/03610731003613771
07. Holthoff VA, Marschner K, Scharf M, et al. Effects of physical activity training in patients with alzheimer's dementia: Results of a pilot RCT study. *PLoS One*. 2015;10(4):1-11. doi:10.1371/journal.pone.0121478
08. Baek JE, Hyeon SJ, Kim M, Cho HY, Hahm SC. Effects of dual-task resistance exercise on cognition, mood, depression, functional fitness, and activities of daily living in older adults with cognitive impairment: a single-blinded, randomized controlled trial. *BMC Geriatr*. 2024;24(1):1-12. doi:10.1186/s12877-024-04942-1
09. Singh et al. The Study of Mental and Resistance Training (SMART) Study-Resistance Training and/or Cognitive Training in Mild Cognitive Impairment: A Randomized, Double-Blind, Double-Sham Controlled Trial. *J Am Med Dir Assoc*. 2014;15(12):873-880. doi:10.1016/j.jamda.2014.09.010
10. Lv J, Liu Y. Effects of momentum-based dumbbell training on motor control in older adults with mild cognitive impairment. *Chinese Journal of Rehabilitation Medicine*. 2019;34(5):544-550. doi:10.3969/j.issn.1001-1242.2019.05.009
11. Vints WAJ, Gökçe E, Šeikinaitė J, et al. Resistance training's impact on blood biomarkers and cognitive function in older adults with low and high risk of mild cognitive impairment: a randomized controlled trial. *European Review of Aging and Physical Activity*. 2024;21(1):1-15. doi:10.1186/s11556-024-00344-9
12. Kušleikienė S, Ziv G, Vints WAJ, et al. Cognitive gains and cortical thickness changes after 12 weeks of resistance training in older adults with low and high risk of mild cognitive impairment: Findings from a randomized controlled trial. *Brain Res Bull*. 2025;222(September 2024). doi:10.1016/j.brainresbull.2025.111249
13. Fernandez-Gonzalo R, Fernandez-Gonzalo S, Turon M, Prieto C, Tesch PA, García-Carreira MDC. Muscle, functional and cognitive adaptations after flywheel resistance training in stroke patients: A pilot randomized controlled trial. *J Neuroeng Rehabil*. 2016;13(1):1-11. doi:10.1186/s12984-016-0144-7
14. Hong SG, Kim JH, Jun TW. Effects of 12-week resistance exercise on electroencephalogram patterns and cognitive function in the elderly with mild cognitive impairment: A randomized controlled trial. *Clinical Journal of Sport Medicine*. 2018;28(6):500-508. doi:10.1097/JSM.0000000000000476
15. Wang L, Wu B, Tao H, et al. Effects and mediating mechanisms of a structured limbs-exercise program on general cognitive function in older adults with mild cognitive impairment: A randomized controlled trial. *Int J Nurs Stud*. 2020;110:103706. doi:10.1016/j.ijnurstu.2020.103706
16. Yoon DH, Kang D, Kim HJ, Kim JS, Song HS, Song W. Effect of elastic band-based high-speed power training on cognitive function, physical performance and muscle strength in older women with mild cognitive impairment. *Geriatr Gerontol Int*. 2017;17(5):765-772. doi:10.1111/ggi.12784
17. Yang JG, Thapa N, Park HJ, et al. Virtual Reality and Exercise Training Enhance Brain, Cognitive, and Physical Health in Older Adults with Mild Cognitive Impairment. *Int J Environ Res Public Health*. 2022;19(20). doi:10.3390/ijerph192013300
18. Li L, Liu M, Zeng H, Pan L. Multi-component exercise training improves the physical and cognitive function of the elderly with mild cognitive impairment: A six-month randomized controlled trial. *Ann Palliat Med*. 2021;10(8):8919-8929. doi:10.21037/apm-21-1809
19. Shimada H, Suzuki T, Makizako H, et al. Effects of multicomponent exercise on cognitive function in older adults with amnestic mild cognitive impairment: a randomized controlled trial. *Alzheimer's & Dementia*. 2012;8(4S_Part_4). doi:10.1016/j.jalz.2012.05.386
20. Avenali M, Picascia M, Tassorelli C, Sinforiani E, Bernini S. Evaluation of the efficacy of physical therapy on cognitive decline at 6-month follow-up in Parkinson disease patients with mild cognitive impairment: a randomized controlled trial. *Aging Clin Exp Res*. 2021;33(12):3275-3284. doi:10.1007/s40520-021-01865-4
21. Mak A, Delbaere K, Refshauge K, et al. Sunbeam Program Reduces Rate of Falls in Long-Term Care Residents With Mild to Moderate Cognitive Impairment or

Dementia: Subgroup Analysis of a Cluster Randomized Controlled Trial. *J Am Med Dir Assoc*. 2022;23(5):743-749.e1. doi:10.1016/j.jamda.2022.01.064
22. De Sá CA, Saretto CB, Cardoso AM, Remor A, Breda CO, da Silva Corralo V. Effects of a physical exercise or motor activity protocol on cognitive function, lipid profile, and BDNF levels in older adults with mild cognitive impairment. *Mol Cell Biochem*. 2024;479(3):499-509. doi:10.1007/s11010-023-04733-z
23. Langoni CDS, Resende TDL, Barcellos AB, et al. Effect of Exercise on Cognition, Conditioning, Muscle Endurance, and Balance in Older Adults with Mild Cognitive Impairment: A Randomized Controlled Trial. *Journal of Geriatric Physical Therapy*. 2019;42(2):E15-E22. doi:10.1519/JPT.0000000000000191
24. Zhang Q, Zhu M, Huang L, et al. A Study on the Effect of Traditional Chinese Exercise Combined With Rhythm Training on the Intervention of Older Adults With Mild Cognitive Impairment. *Am J Alzheimers Dis Other Demen*. 2023;38(48):1-12. doi:10.1177/15333175231190626
25. Hauer K, Schwenk M, Zieschang T, Essig M, Becker C, Oster P. Physical training improves motor performance in people with dementia: A randomized controlled trial. *J Am Geriatr Soc*. 2012;60(1):8-15. doi:10.1111/j.1532-5415.2011.03778.x
26. Suttanon P, Hill KD, Said CM, et al. Feasibility, safety and preliminary evidence of the effectiveness of a home-based exercise programme for older people with Alzheimer's disease: A pilot randomized controlled trial. *Clin Rehabil*. 2013;27(5):427-438. doi:10.1177/0269215512460877
27. Sanders LMJ, Hortobágyi T, Karssemeijer EGA, Van Der Zee EA, Scherder EJA, Van Heuvelen MJG. Effects of low- And high-intensity physical exercise on physical and cognitive function in older persons with dementia: A randomized controlled trial. *Alzheimers Res Ther*. 2020;12(1):1-15. doi:10.1186/s13195-020-00597-3
28. de Oliveira Silva F, Ferreira JV, Plácido J, et al. Three months of multimodal training contributes to mobility and executive function in elderly individuals with mild cognitive impairment, but not in those with Alzheimer's disease: A randomized controlled trial. *Maturitas*. 2019;126(April):28-33. doi:10.1016/j.maturitas.2019.04.217
29. Ghahfarrokhi MM, Shirvani H, Rahimi M, Bazgir B, Shamsadini A, Sobhani V. Feasibility and preliminary efficacy of different intensities of functional training in elderly type 2 diabetes patients with cognitive impairment: a pilot randomised controlled trial. *BMC Geriatr*. 2024;24(1):1-15. doi:10.1186/s12877-024-04698-8
30. Fonte C, Smania N, Pedrinolla A, et al. Comparison between physical and cognitive treatment in patients with MCI and Alzheimer's disease. *Aging*. 2019;11(10):3138-3155. doi:10.18632/aging.101970
31. Casas-Herrero Á, Sáez de Asteasu ML, Antón-Rodrigo I, et al. Effects of Vivifrail multicomponent intervention on functional capacity: a multicentre, randomized controlled trial. *J Cachexia Sarcopenia Muscle*. 2022;13(2):884-893. doi:10.1002/jcsm.12925
32. Shaw I, Cronje M, Shaw BS. Group-based exercise as a therapeutic strategy for the improvement of mental outcomes in mild to moderate alzheimer's patients in low resource care facilities. *Asian J Sports Med*. 2021;12(1):1-6. doi:10.5812/asjsm.106593
33. Cezar NO de C, Ansai JH, Oliveira MPB de, et al. Feasibility of improving strength and functioning and decreasing the risk of falls in older adults with Alzheimer's dementia: a randomized controlled home-based exercise trial. *Arch Gerontol Geriatr*. 2021;96(March). doi:10.1016/j.archger.2021.104476
34. Mollinedo Cardalda I, López A, Cancela Carral JM. The effects of different types of physical exercise on physical and cognitive function in frail institutionalized older adults with mild to moderate cognitive impairment. A randomized controlled trial. *Arch Gerontol Geriatr*. 2019;83(May):223-230. doi:10.1016/j.archger.2019.05.003
35. Kim MJ, Han CW, Min KY, et al. Physical Exercise with Multicomponent Cognitive Intervention for Older Adults with Alzheimer's Disease: A 6-Month Randomized Controlled Trial. *Dement Geriatr Cogn Dis Extra*. 2016;6(2):222-232. doi:10.1159/000446508
36. Bossers WJR, Van Der Woude LHV, Boersma F, Hortobágyi T, Scherder EJA, Van Heuvelen MJG. A 9-Week Aerobic and Strength Training Program Improves Cognitive and Motor Function in Patients with Dementia: A Randomized, Controlled Trial. *American Journal of Geriatric Psychiatry*. 2015;23(11):1106-1116. doi:10.1016/j.jagp.2014.12.191
37. Telenius EW, Engedal K, Bergland A. Long-term effects of a 12 weeks high-intensity functional exercise program on physical function and mental health in nursing home residents with dementia: A single blinded randomized controlled trial Physical functioning, physical health and activity. *BMC Geriatr*. 2015;15(1):1-11. doi:10.1186/s12877-015-0151-8
38. Toots A, Littbrand H, Boström G, et al. Effects of exercise on cognitive function in older people with dementia: A randomized controlled trial. *Journal of Alzheimer's Disease*. 2017;60(1):323-332. doi:10.3233/JAD-170014

39. de Souto Barreto P, Cesari M, Denormandie P, Armaingaud D, Vellas B, Rolland Y. Exercise or Social Intervention for Nursing Home Residents with Dementia: A Pilot Randomized, Controlled Trial. *J Am Geriatr Soc*. 2017;65(9):E123-E129. doi:10.1111/jgs.14947
40. Roach KE, Tappen RM, Kirk-Sanchez N, Williams CL, Loewenstein D. A randomized controlled trial of an activity specific exercise program for individuals with alzheimer disease in long-term care settings. *Journal of Geriatric Physical Therapy*. 2011;34(2):50-56. doi:10.1519/JPT.0b013e31820aab9c
41. Henskens M, Nauta IM, Van Eekeren MCA, Scherder EJA. Effects of Physical Activity in Nursing Home Residents with Dementia: A Randomized Controlled Trial. *Dement Geriatr Cogn Disord*. 2018;46(1-2):60-80. doi:10.1159/000491818
42. David S, Costa AS, Hohenfeld C, et al. Modulating effects of fitness and physical activity on Alzheimer's disease: Implications from a six-month randomized controlled sports intervention. *Journal of Alzheimer's Disease*. 2025;103(2):552-569. doi:10.1177/13872877241303764
43. David S, Costa AS, Hohenfeld C, et al. Modulating effects of fitness and physical activity on Alzheimer's disease: Implications from a six-month randomized controlled sports intervention. *Journal of Alzheimer's Disease*. 2025;103(2):552-569. doi:10.1177/13872877241303764
44. Shokri G, Mohammadian F, Noroozian M, Amani-Shalamzari S, Suzuki K. Effects of remote combine exercise-music training on physical and cognitive performance in patients with Alzheimer's disease: a randomized controlled trial. *Front Aging Neurosci*. 2023;15(January):1-9. doi:10.3389/fnagi.2023.1283927
45. Li PWC, Yu DSF, Siu PM, Wong SCK, Chan BS. Peer-supported exercise intervention for persons with mild cognitive impairment: A waitlist randomised controlled trial (the BRAin Vitality Enhancement trial). *Age Ageing*. 2022;51(10):1-10. doi:10.1093/ageing/afac213
46. Lamb SE, Sheehan B, Atherton N, et al. Dementia And Physical Activity (DAPA) trial of moderate to high intensity exercise training for people with dementia: Randomised controlled trial. *BMJ (Online)*. 2018;361. doi:10.1136/bmj.k1675
47. Vreugdenhil A, Cannell J, Davies A, Razay G. A community-based exercise programme to improve functional ability in people with Alzheimer's disease: A randomized controlled trial. *Scand J Caring Sci*. 2012;26(1):12-19. doi:10.1111/j.1471-6712.2011.00895.x
48. Verdelho A, Correia M, Gonçalves-Pereira M, et al. Physical Activity in Mild Vascular Cognitive Impairment: Results of the AFIVASC Randomized Controlled Trial at 6 Months. *Journal of Alzheimer's Disease*. 2024;101(4):1379-1392. doi:10.3233/JAD-240246
49. Bademli K, Lok N, Canbaz M, Lok S. Effects of Physical Activity Program on cognitive function and sleep quality in elderly with mild cognitive impairment: A randomized controlled trial. *Perspect Psychiatr Care*. 2019;55(3):401-408. doi:10.1111/ppc.12324
50. Padala KP, Padala PR, Lensing SY, et al. Home-Based Exercise Program Improves Balance and Fear of Falling in Community-Dwelling Older Adults with Mild Alzheimer's Disease: A Pilot Study. *Journal of Alzheimer's Disease*. 2017;59(2):565-574. doi:10.3233/JAD-170120
51. Kemoun G, Thibaud M, Roumagne N, et al. Effects of a physical training programme on cognitive function and walking efficiency in elderly persons with dementia. *Dement Geriatr Cogn Disord*. 2010;29(2):109-114. doi:10.1159/000272435
52. Prick AE, De Lange J, Scherder E, Twisk J, Pot AM. The effects of a multicomponent dyadic intervention with physical exercise on the cognitive functioning of people with dementia: A randomized controlled trial. *J Aging Phys Act*. 2017;25(4):539-552. doi:10.1123/japa.2016-0038
53. Greblo Jurakic Z, Krizanic V, Sarabon N, Markovic G. Effects of feedback-based balance and core resistance training vs. Pilates training on cognitive functions in older women with mild cognitive impairment: a pilot randomized controlled trial. *Aging Clin Exp Res*. 2017;29(6):1295-1298. doi:10.1007/s40520-017-0740-9
54. Kim J, Yim J. Effects of an exercise protocol for improving handgrip strength and walking speed on cognitive function in patients with chronic stroke. *Medical Science Monitor*. 2017;23:5402-5409. doi:10.12659/MSM.904723
55. Levinger P, Goh AMY, Dunn J, et al. Exercise interveNtion outdoor proJect in the cOmmunitY – results from the ENJOY program for independence in dementia: a feasibility pilot randomised controlled trial. *BMC Geriatr*. 2023;23(1):1-16. doi:10.1186/s12877-023-04132-5
56. Akbuga Koc E, Yazici-Mutlu Ç, Cinar N, Sahiner T. Comparison of the effect of online physical exercise and computerized cognitive stimulation in patients with Alzheimer's disease during the Covid-19 pandemic. *Complement Ther Clin Pract*. 2024;57(May):10-20. doi:10.1016/j.ctcp.2024.101881
57. Lok N, Tosun AS, Lok S, Temel V, Aydın Z. Effect of physical activity program applied to patients with Alzheimer's disease on cognitive functions and depression

level: a randomised controlled study. *Psychogeriatrics*. 2023;23(5):856-863. doi:10.1111/psyg.13010
58. Yan Y, Xu Y, Wang X, et al. The effect of multi-component exercise intervention in older people with Parkinson's disease and mild cognitive impairment: A randomized controlled study. *Geriatr Nurs (Minneap)*. 2024;60:137-145. doi:10.1016/j.gerinurse.2024.08.028
59. Papatsimpas V, Vrouva S, Papathanasiou G, et al. Does Therapeutic Exercise Support Improvement in Cognitive Function and Instrumental Activities of Daily Living in Patients with Mild Alzheimer's Disease? A Randomized Controlled Trial. *Brain Sci*. 2023;13(7). doi:10.3390/brainsci13071112
60. Dawson N, Judge KS, Gerhart H. Improved Functional Performance in Individuals with Dementia after a Moderate-Intensity Home-Based Exercise Program: A Randomized Controlled Trial. *Journal of Geriatric Physical Therapy*. 2019;42(1):18-27. doi:10.1519/JPT.0000000000000128
61. Bo W, Lei M, Tao S, et al. Effects of combined intervention of physical exercise and cognitive training on cognitive function in stroke survivors with vascular cognitive impairment: a randomized controlled trial. *Clin Rehabil*. 2019;33(1):54-63. doi:10.1177/0269215518791007
62. Sobol NA, Hoffmann K, Frederiksen KS, et al. Effect of aerobic exercise on physical performance in patients with Alzheimer's disease. *Alzheimer's and Dementia*. 2016;12(12):1207-1215. doi:10.1016/j.jalz.2016.05.004
63. Almeida S, Paixão C, da Silva MG, Marques A. Lifestyle-integrated functional exercise for people with Dementia: A pilot study. *J Aging Phys Act*. 2021;29(5):771-780. doi:10.1123/JAPA.2020-0349
64. Gebhard D, Mess F. Feasibility and Effectiveness of a Biography-Based Physical Activity Intervention in Institutionalized People With Dementia: Quantitative and Qualitative Results From a Randomized Controlled Trial. *J Aging Phys Act*. 2022;30(2):237-251. doi:10.1123/japa.2020-0343
65. Stevens J, Killeen M. A randomised controlled trial testing the impact of exercise on cognitive symptoms and disability of residents with dementia. *Contemporary nurse : a journal for the Australian nursing profession*. 2006;21(1):32-40. doi:10.5172/conu.2006.21.1.32
66. Ullrich P, Werner C, Schönstein A, et al. Effects of a Home-Based Physical Training and Activity Promotion Program in Community-Dwelling Older Persons with Cognitive Impairment after Discharge from Rehabilitation: A Randomized Controlled Trial. *Journals of Gerontology - Series A Biological Sciences and Medical Sciences*. 2022;77(12):2435-2444. doi:10.1093/gerona/glac005
67. Papamichail P, Sagredaki ML, Bouzineki C, Kanellopoulou S, Lyros E, Christakou A. The Effectiveness of an Exercise Program on Muscle Strength and Range of Motion on Upper Limbs, Functional Ability and Depression at Early Stage of Dementia. *J Clin Med*. 2024;13(14):1-10. doi:10.3390/jcm13144136
68. Rolland Y, Pillard F, Klapouszczak A, et al. Exercise program for nursing home residents with Alzheimer's disease: A 1-year randomized, controlled trial. *J Am Geriatr Soc*. 2007;55(2):158-165. doi:10.1111/j.1532-5415.2007.01035.x
69. Kovas et al. Effects of a multimodal exercise program on balance, functional mobility and fall risk in older adults with cognitive impairment: a randomized controlled single-blind study. 2011;47(3):381-390.
70. Brett L, Stapley P, Meedya S, Traynor V. Effect of physical exercise on physical performance and fall incidents of individuals living with dementia in nursing homes: a randomized controlled trial. *Physiother Theory Pract*. 2021;37(1):38-51. doi:10.1080/09593985.2019.1594470
71. Uemura K, Doi T, Shimada H, et al. Effects of Exercise Intervention on Vascular Risk Factors in Older Adults with Mild Cognitive Impairment: A Randomized Controlled Trial. *Dement Geriatr Cogn Dis Extra*. 2012;2(1):445-455. doi:10.1159/000343486
72. Doi T, Makizako H, Shimada H, et al. Effects of multicomponent exercise on spatial-temporal gait parameters among the elderly with amnestic mild cognitive impairment (aMCI): Preliminary results from a randomized controlled trial (RCT). *Arch Gerontol Geriatr*. 2013;56(1):104-108. doi:10.1016/j.archger.2012.09.003
73. Santana-Sosa E, Barriopedro MI, López-Mojares LM, Pérez M, Lucia A. Exercise training is beneficial for Alzheimer's patients. *Int J Sports Med*. 2008;29(10):845-850. doi:10.1055/s-2008-1038432
74. Rivas-Campo Y, Aibar-Almazán A, Afanador-Restrepo DF, et al. Effects of High-Intensity Functional Training (HIFT) on the Functional Capacity, Frailty, and Physical Condition of Older Adults with Mild Cognitive Impairment: A Blind Randomized Controlled Clinical Trial. *Life*. 2023;13(5):1-16. doi:10.3390/life13051224
