# Supplementary material for: Dance versus other exercise modalities in mild cognitive impairment and dementia: comparative efficacy from a systematic review and bayesian network meta-analysis
Source: Front Physiol. 2026 Mar 25;17:1782774. doi: 10.3389/fphys.2026.1782774 (PMC13056856; doi:10.3389/fphys.2026.1782774)
Supplement: Supplementary file 2 [file Table4.pdf]

Supplementary Table 4. Exercise (all types) Intervention Programs

Author/Year/Type of RCT	Training Intervention Programs	Training Outcomes
**Dance**		
**Kropacova et al.[1] 2019 2-arm RCT**	**Dance type:** Choreographed-mixed-dance intervention<br>**Intensity:** N/A<br>**Duration:** 24-week<br>**Training load:** 60-min, 3 times/week<br>**Dance proposal:** *Professional instructors* from the Faculty of Sport Studies at Masaryk University conducted the intervention. Dances such as *Irish country, African dance, Greek dance, and tango* were performed. The choreography was divided into several short learning segments, which were continually developed and combined into a final choreographed dance during ongoing lessons.	Subtle favorable effect on executive function
**Esmail et al.[2] 2020 3-arm RCT**	**Dance type:** Dance movement therapy (DMT)<br>**Intensity:** N/A<br>**Duration:** 12-week<br>**Training load:** 60-min, 3 times/week<br>**Dance proposal:** The DMT intervention used in this study was designed according to the standards of the *American Dance Therapy Association (ADTA)* and adapted to the needs of the healthy older participants. An *ADTA registered dance/movement therapist or a supervised trainee* led the group training. A common dance/movement therapy structure was followed for each session (*opening circle, warm up, development, and closure*). The program was comprised of expressive movement and guided gestures to expand participants' movement repertoire. Each facilitator was also given a list of themes pertinent, such as *lifestyle improvement, body awareness, relaxation, balance, self-care, socialization, rhythm and enjoyment*. Throughout the training program, props such as the *Octaband©, the CoOper Blanket©, the Elastablast©, colorful scarves, exercise balls, TheraBands™ and tennis balls* were used. Music of differing styles was chosen based on the objectives of the movement, sometimes proposed by the group facilitator or the participants.	1. Both executive and non-executive functioning improvement<br>2. Improved cardiovascular fitness
**Bisbe et al.[3] 2020 2-arm RCT**	**Dance type:** Choreography aerobic dance<br>**Intensity:** Without exceeding 2–3/10 RPE<br>**Duration:** 12-week<br>**Training load:** 60-min, 2 times/week<br>**Dance proposal:** Choreographed aerobic dances were prepared by a *professional choreographer,* and a variety of music styles were used (*salsa, rock, rumba, pop, and jive*). Choreography videos were projected on a screen for visual support, while the physiotherapists taught and re-iterated the steps to reinforce acquisition. Choreographed sessions followed an invariable structure: (1) *Learning the steps*: participants learned the steps fractionally, following the instructions and watching the tutorial videos without music (1 attempt); (2) *Performing choreography*: participants put the learned steps into practice, repeating the	Benefits in verbal recognition memory

	choreographed video with music (4–5 attempts); (3) *Music support only*: participants reproduced the sequence of learned steps without visual support, by only listening to the music (1 attempt). Periods of rest where participants could sit down, and drink water were included after each attempt.	
**Qi et al.[4]**<br>**2019**<br>**2-arm RCT**	**Dance type:** Aerobic dance<br>**Intensity:** 60%–80% of HRmax<br>**Duration:** 12-week<br>**Training load:** 35-min, 3 times/week<br>**Dance proposal**: Subjects were taught the new dance routine for 2 weeks, when they had learned the dance and could follow the routine correctly. The new dance routine was designed by a *physical therapist* who had > 5 years of physical therapy experience. The intervention lasted 35 mins, including *a 5-min warm-up, a 25-min dance with a target HR and a 5-min cool-down to music*. The warm-up consisted of 3 movements: stepping and breathing, head movements, side bending, and turning. The dance routines consisted of 8 movements: *knee bending, heel lifts, boxing, shoulder movements, kicking, square stepping, sculling and jumping*. *The cool-down consisted of 3 movements: shoulder movements, stepping and breathing, and low-speed stepping.* The dance routine was deemed to be quite complicated as it contained repetition and variety; therefore, it required memory, concentration and dual-task function to complete the dance correctly. During the dance, there *were two physical therapists monitoring the HR* with a cardiotachometer to ensure that all subjects maintain a pre-arranged intensity level during training.	Effectively improved the cognitive function
**Lazarou et al.[5]**<br>**2017**<br>**2-arm RCT**	**Dance type:** International Ballroom Dancing<br>**Intensity:** N/A<br>**Duration:** 40-week<br>**Training load:** 60-min, 2 times/week<br>**Dance proposal:** An *experienced dance instructor* supervised the dance class. Each 60-min dance class included *a 5-min warm-up, 45-min of new material (figures/dances), and a 10-min cool down* period with a dance of participants' preference. The participants familiarized with several international ballroom dances such as *Tango, Waltz, Viennese Waltz, Fox trot, Rumba, Chachacha, Swing, Salsa, Merengue, Disco–Hustle, as well as with Greek traditional ballroom dancing*. All dances were first introduced with their special music features and their origin. Each lesson included a combination of 2 or 3 dances in rotation. The combination was *based on movement similarities, so participants could remember step combination easier, and rhythm variation (slow and faster dances)* to make lesson easier to attend by elderly. Dance steps/figures presented, gradually became more complex.	Positive impacts of:<br>1. Cognitive functions<br>2. Mood<br>3. Behavior<br>4. Without increasing the risk of cognitive deterioration
**Franco et al.[6]**<br>**2020**<br>**2-arm RCT**	**Dance type:** Senior dance program<br>**Intensity:** Moderate-level intensity<br>**Duration:** 12-week<br>**Training load:** 60-min, 2 times/week<br>**Dance proposal:** The number of participants per class ranged from *10 to 15*. Senior Dance classes were led by 2 *certified instructors in Senior Dance* who were also *physical therapists*	Effective in improving:<br>1. Balance<br>2. Mobility

	and had the same level of training with at least 1 year experience as certified instructors. The dance classes consisted of *a range of choreographies, including rhythmic and simple movements with rhythmic folk songs.* During the classes, participants were required to memorize song lyrics and practiced the movements while sitting or standing or in a combination of both positions, quickly or slowly, *in circles, individually, in pairs, or in small groups*. The attention required to learn the choreographies challenges balance, motor coordination, and cognitive function. Participants in the Senior Dance group were instructed *to practice the choreographies at home* for 10 to 20 mins on at least 2 days/week.	
**Doi et al.[7]**<br>**2017**<br>**3-arm RCT**	**Dance type:** Ballroom dance<br>**Intensity:** N/A<br>**Duration:** 40-week<br>**Training load:** 60-min, 1 time/week<br>**Dance proposal:** The dance program was based on ballroom dance, including *salsa, rumba, waltz, cha-cha, blues, jitterbug, and tango*. One or 2 *professional dance instructors* taught each session at a *dance studio*. Between *11 to 16* individuals participated in each session, which started with a short warm-up and finished with a cool-down period. The instructor demonstrated a sequence of dance steps. Participants practiced the steps until they could dance the sequence satisfactorily. After participants mastered a sequence, they were taught a new set of dance steps. At the end of each session, the instructors gave participants *homework* to dance the sequence learned in that session. Different dances were taught, and participants were *paired* randomly into couples.	Improvements in:<br>1. Memory function<br>2. General cognitive status<br>3. Attention<br>4. Executive functions
**Blumen et al.[8]**<br>**2023**<br>**2-arm RCT**	**Dance type:** Ballroom dancing<br>**Intensity**: ≤ 14/20 RPE<br>**Duration:** 24-week<br>**Training load:** 90-min, 2 times/week<br>**Dance proposal:** Dance intervention consisted of *warm-up (10 mins stretching to music), low-intensity dances (30 mins; e.g., foxtrot, waltz), a break (10 mins), moderate or higher intensity dances (30 mins; e.g., salsa, east coast swing), and cool-down (10 mins).* Under the supervision of *experienced instructors* (in teaching older adults), participants practiced dancing in groups of *2 to 6*. Research staff trained by a board-certified Rehabilitation Medicine specialist *monitored HR and BP* prior to warm-up, during the break, and after cooling down. If systolic BP was > 150 mmHg, participants were not allowed to start or continue a session. Physical exertion was monitored with the subjective *Borg scale*.	Did not generate greater benefits than control groups
**Zhu et al.[9]**<br>**2018**<br>**2-arm RCT**	**Dance type:** Aerobic dance<br>**Intensity:** 60%–80% of HRmax<br>**Duration:** 12-week<br>**Training load:** 35-min, 3 times/week<br>**Dance proposal:** In the first 2 weeks, a *dance instructor* taught the participants a specially designed dance routine to ensure all participants could perform the dance correctly. Patients danced together with the dance instructor in each session. After 3 months, patients were encouraged to continue practicing the dance routine in their own *home*. The target HR was set	Improved cognitive function (especially episodic memory and processing speed)

	as 60-80% of the HRmax to ensure safety while producing the desired effect. Patients were asked to wear *cardiotachometers* (ONrhythm 50, GEONATURE) on their left wrists during each session, and their *HR were monitored* by 2 *physical therapists* during the dance class. The dance routine designed by an *experienced physical therapist*. The routine included *a 5-minute warm-up, a 25-min dance with the target HR, and a 5-minute cool-down*. Patients performed this dance routine in synchronization with a musical phrase of 8 rhythmical meters with a general value of 4/4 each. The warm-up session included stepping and breathing, head movement, side bending, and turning exercises. The cool-down session included slow shoulder movement, stepping, and breathing exercises. The dance session included 7 sub-sessions performed consecutively: *knee bending, heel up, boxing, shoulder movement, kicking, square-stepping, and sculling exercises*. Each sub-session comprised 6 movements (2 repetitions of stepping, first and second movements in the sub-session) and was repeated 3 times. For example, in the kicking sub-session, the first and second movements were left kicking and right kicking, respectively.	
**Song et al.[10]**<br>**2024**<br>**2-arm RCT**	**Dance type:** Aerobic dance<br>**Intensity:** N/A<br>**Duration:** 16-week<br>**Training load:** 60-min, 3 times/week<br>**Dance proposal:** Training sessions per week in a group-based format (*20 participants per group*). The exercise program was designed by a team comprising a *nurse academician*, a *physiotherapis*t, and *exercise physiologist* following the recommendations of the *ACSM* position stand on exercise and PA for older individuals. The training program started with *10-min warm-up session, after that a 40-min aerobic dancing session, and ended with a 10-min cool-down session*. Warm-up and cool-down exercise included walking and stationary stretching exercises for trunk and limb joints. The program involved dancing patterns of stepping up and down on a 10-cm stepping bench. Joyful music was integrated into the exercise regimen to facilitate adherence. Meanwhile, various sets of entertaining upper limb movements resembling daily activities, such as washing the hands, were added to facilitate overall body movement and adherence. The participants were instructed to adjust their movements to achieve exertion in moderate-intensity aerobic exercise, which was indicated by the feeling of "somewhat hard" at the Borg rating of 12–14. The aerobic dancing program was conducted by 2 *nurses*, one serving as an instructor and the other as a facilitator. Vital signs and contradictory symptoms were assessed before training.	Improved:<br>1. Sleep quality<br>2. Cognitive function
**Chang et al.[11,12]**<br>**2021**<br>**2-arm RCT**	**Dance type:** Chinese square dance<br>**Intensity:** HR 100–140 beats/min<br>**Duration:** 18-week<br>**Training load:** 40-min, 3 times/week<br>**Dance proposal:** Square dance music has a simple melody, low movement activity is required, and *the central movement structures are handclapping, high-fiving, chest expansion, arm extension, and leg kicking*. One week before starting the experiment, 2 *national social sports instructors* with professional dance training gave 3 lessons in 2 nursing homes to ensure that each participant had mastered the basic movements. After 1 week of study, we provided square	Significant improved:<br>1. Cognitive function<br>2. Health-related QoL<br>3. Reducing depressive symptoms

	dance instructional videos for the experimental group to review. During the teaching process, one instructor demonstrates in front of the participants, and another instructor assists in teaching and correcting incorrect movements. For the formal experiment, an instructor is responsible for leading the practice, and the nursing home arranges a *staff member* to assist with teaching and to ensure the safety of participants. The intervention group performed the square dance exercise program *outdoors* (during periods of inclement weather, square dance exercise was held indoors). The exercise was led by a professional national social sports instructor at the same time on the intervention days. There was *a 5-min warm-up exercise (joint finger movement, etc.) before the official start, followed by a 30-min square dance exercise and a 5-min relaxation exercise (i.e., deep breathing and stretching) at the end of the session*. During the square dance exercise, participants were required to wear a *sports watch* to monitor their HR.	
**Winckel et al.[13]**<br>**2004**<br>**2-arm RCT**	**Dance type:** Music-based dance therapy<br>**Intensity:** N/A<br>**Duration:** 12-week<br>**Training load:** 30-min, 7 times/week<br>**Dance proposal:** To standardize the patient-therapist relation, the same *therapist* was in charge of both the music-based dance therapy and the conversational session. The residents sat in a circle, facing the therapist, so they could follow the exercises through constantly mimicking the movements of the instructor. *The therapist used specific one-step verbal instructions, combined with continuous visual demonstration.* To enhance interest and participation, the music was chosen with consideration of their age. Therefore, folkloric accordion songs, such as *polka, folk, country and western music* was chosen. The exercises focused on upper and lower body strengthening, as well as balance, trunk movements and flexibility training.	A beneficial effect of cognition
**Bracco et al.[14]**<br>**2023**<br>**2-arm RCT**	**Dance type:** Ballroom dance<br>**Intensity:** N/A<br>**Duration:** 12-week<br>**Training load:** 60-min, 2 times/week<br>**Dance proposal:** The sessions were led by *nursing staff* who previously received training in therapeutic tango by the University of Burgundy. A list of reproductions of *tango, waltzes and milonga* was available, to adapt the music to the different types of exercise. Twice a month the sessions were conducted by a team of dance movement-therapist and musician, in order to maintain motivation, bring novelty and give cohesion to the interventions. The facilitator worked from non-verbal communication, relying on the remaining capacities of each participant. The therapeutic tango session was composed basically as follows: (1) *Scenario and warm-up*: seated exercises to mobilize the lower and upper limbs, the head and the trunk as well as singing to warm up the voice and promote social ties. (2) *Central part:* dance standing or sitting. Solo, couple or group. Different aspects of the tango were practiced: forward, backward, sidestep, square, rectangle, *ochos*, etc. Changes in pace, speed and direction. Improvisation through spontaneous expression. Physical connection through hugging or other interactions is also an important aspect of the intervention. (3) *Cool-down*: seated rituals such	1. Improved gait speed<br>2. Mitigated the decline in functional mobility and ADL skill capacities

	as chanting and breathing exercises. (4) *The farewells*: moment of exchange and feedback between the participants and the staff.	
**Zhu et al.[15] 2022 2-arm RCT**	**Dance type:** Aerobic dance<br>**Intensity:** Moderate, 60%–80% of HRmax<br>**Duration:** 12-week<br>**Training load:** 35-min, 3 times/week<br>**Dance proposal:** The dance routine, which was designed by an experienced *physical therapist* (PT) with more than 5 years of experience in exercise intervention administered the dance routine. The dance routine included *a 5-min warm-up, a 25-min dance with a target HR, and a 5-min cool-down period*. One PT led the group dance, and the other one was responsible for *monitoring HR* and dancing performance. During the training, *cardiotachometers* (ONrhythm 50, GEONATURE) were used to monitor the HR of each participant. Each training session consisted of *11–16* participants, and during the first 2 weeks, the PT demonstrated a sequence of dancing steps and taught the participants how to combine the steps and follow the music. This dance routine was composed of 7 sub-sessions, *namely, knee bending, heel up, boxing, shoulder movement, kicking, square-stepping, and sculling exercises.*	1. Increased the right and total hippocampal volumes<br>2. Improved:<br>a) Cognitive function<br>b) Improved episodic memory
**Ho et al.[16] 2018 3-arm RCT**	**Dance type:** Dance movement therapy (DMT)<br>**Intensity:** 40%–60% of VO_2max<br>**Duration:** 12-week<br>**Training load:** 60-min, 2 times/week<br>**Dance proposal:** The dance-movement intervention was led by a *registered dance-movement therapist* or one in training. To enhance the effectiveness, more emphasis was placed on easy steps and rhythm, and the movements were modified so that they could also be performed in *both standing and sitting positions* to cater to the participants' bodily needs and to reduce tiredness. Materials, such as *scarves, elastic bands, ribbons, and small musical instruments* were used more often to *enhance the sense of working with objects, elasticity, strength, and coordination*. DMT comprised 4 main elements: *namely, simple group dance, movement games, improvisational dance movement, and movement interactions* among group members. These elements aimed to encourage the *participants to remember steps and sequences, improve mood and vitality, foster imagination, creativity, and personal expression, and enhance communication and social exchange*, respectively. Finally, *verbal sharing* was done as a group discussion following the end of each session. All participants were invited to use 1 or 2 words or a gesture or movement to express their feelings about the movement experience or to say something supportive or caring to each other, which facilitated articulation and mutual support among the participants.	1. Demonstrated short-term multifaceted effects in better psychosocial functioning (decreased depression, loneliness, and negative mood)<br>2. Improved The Instrumental activities of daily living (IADL)<br>3. Improved neuroendocrine functioning
**Thiel et al.[17] 2024 2-arm RCT**	**Dance type:** A multitude of individual dance genres (line dance, jazz dance, square dance, and Latin-American dances)<br>**Intensity:** N/A<br>**Duration:** 12-week<br>**Training load:** 90-min, 2 times/week<br>**Dance proposal:** The training session was divided into four parts. The beginning was a *warm-up* with mobilization exercises for the whole body and the implementation of a coordinative	Preserved elements of physical fitness

	part using *various arm–leg combinations* with increasing complexity. In addition, the dances that had already been rehearsed were gradually integrated into the warm-up during the program to activate the cardiovascular system and set a stimulus that triggers adaptations of the cardiovascular system. The main part was divided into two sections. The first section of the main part was strongly characterized by dance combinations that focused on complex coordinative tasks. Some of the choreographies in this part were danced with *small equipment such as sticks, towels, or physio balls as drums*. The second section of the main part focused on the *strength-endurance aspects of dancing.* Choreographies were employed and small fitness *equipment such as brasils, fitness tires (1.2 kg), and small dumbbells were used for dancing to emphasize the physical demands of dancing.* The last part of each training was a *cool-down* phase including a feet-to-head stretching routine and a sequence of low-intensity mobility exercises that was maintained over the entirety of the intervention. The participants could therefore internalize the routine sequences and execution of the exercises. Different genres of music and rhythms were offered so that the training remained varied and different musical preferences catered to. *Challenging dance steps were broken down into progressions and put together piece by piece to form the entire choreography and dance sequence.*	
**Sanchez-Alcala et al.[18,19]**<br>**2025**<br>**2-arm RCT**	**Dance type:** Aerobic dance<br>**Intensity:** N/A<br>**Duration:** 12-week<br>**Training load:** 60-min, 2 times/week<br>**Dance proposal:** Each session was divided into three phases: (1) *Warm-up Phase (10 min):* This phase included low-intensity activities based on stretching and flexibility movements performed at a slow pace to facilitate the adaptation to rhythm and coordination; (2) *Core Phase (40 min)*: During this phase, participants performed moderate-intensity dance steps involving continuous movements involving continuous movements of the lower limbs, trunk, and intermittent arm movements. Each song lasted approximately 4 min with 2-min breaks between songs. There was a gradual progression in the complexity of choreographies over the weeks. The dance steps included flexion–extension, abduction and adduction, lateral displacements, rotations, changes in rhythm, forward and backward movements, foot position changes, heel lifts, and movements of the upper and/or lower extremities with a gradual progression in complexity over the weeks. Choreographies were carefully selected and sequenced to ensure a logical progression in technical complexity, and different musical styles (salsa, rock, rumba, pop, merengue, bachata) were used for each choreography; (3) *Cool-down Phase (10 min)*: This phase focused on gentle stretching exercises, accompanied by relaxing music, to facilitate a transition to a calm state. This intervention was supervised and led by a *qualified instructor* with training in physiotherapy and fitness training and previous experience in leading physical exercise programs for older adults. To ensure the safety of participants, the sessions were conducted in *suitable and safe facilities*, with equipment and spaces designed to minimize the risk of falls or injuries, and clear protocols were established to handle any emergency situation, including the *availability of first aid* and *rapid access to medical services.*	Improved mental health and quality of life
Exergaming		

**Eggenberger et al.[20,21]**<br>**2015**<br>**3-arm RCT**	**Intensity:** 5–7 /10 RPE<br>**Duration:** 24-week<br>**Training load:** 60-min, 2 times/week<br>**Intervention proposal:** The training sessions were performed in groups of *5 to 6* participants, under the instruction of *2 trained postgraduate student*s. At least 1 day was included between sessions for recovery. The 3 multicomponent programs consisted of 20-min aerobic endurance training either video game:<br>*Dancing:* This training component combines an attention demanding cognitive task with a simultaneous motor coordination aspect. Two *Impact Dance Platforms* (Positive Gaming BV, Haarlem, the Netherlands) were used and created various levels of difficulty in step patterns and frequency with *the StepMania Software*. Several styles of music were selected to add variety and meet preferences of participants. Participants stood on the one-by-one meter platform, which contained 4 pressure sensitive areas to detect steps forward, backward, to the left, and to the right, respectively. Stepping sequences were cued with arrows appearing on a large screen and had to be performed exactly when an arrow reached a highlighted area on the screen in order to achieve best scores in the game. Participants were holding on to ropes for security reasons. Training difficulty was adapted to each individual's coordination ability and was increased progressively.<br>*Treadmill memory training:* Program MEMORY comprised treadmill walking with verbal memory exercise as a simultaneous cognitive-physical training. Verbal memory training consisted of a computer-based serial position training that was presented on a computer screen in front of the treadmill, with a standard computer mouse as an input device. E-Prime 2.0 Professional software (*Psychology Software Tools*, Pittsburgh, PA, USA) was used to program the training. Participants were asked to memorize the correct sequence of 3–20 words lighting up one after the other for 3 seconds on the computer screen. Thereafter, a distraction task was followed where participants had to define if 3 presented words had a meaning or not. Then, the initially memorized words were presented again, either in the same or another sequence, and participants had to decide if the sequence remained the same or not, by pressing the mouse button. The initial level for this training was set at a sequence of 3 words and was extended by 1 word as soon as the participants reached 80% of correct answers within the level. Treadmill speed and inclination were set individually for each participant.<br>*A complementary strength and balance exercises (*20 mins each). The exercise training principles of progression and overload were applied for every training component, and they were adapted to each participant's abilities such that a moderate to vigorous intensity was achieved.	Advantageous to boots performance in executive function (switching attention and working memory)
**Hughes et al.[22]**<br>**2014**<br>**2-arm RCT**	**Intensity:** N/A<br>**Duration:** 24-week<br>**Training load:** 90-min, 1 time/week<br>**Intervention proposal:** *The Nintendo Wii^{TM} gaming console* was used for interactive video gaming. The Wii^{TM} uses a wireless remote device with motion-sensing capabilities. Players use their arms and/or bodies to simulate actions required for each game (e.g., swinging a golf club and throwing a bowling ball). Participants played the Wii^{TM} in stable groups of *3 or 4*	Medium-sized effects in favor of the intervention group for:<br>1. Objective and subjective cognitive functioning<br>2. Physical functioning

	members. The Wii Sports games, including *bowling, golf, tennis, and baseball, comprised the "core" games*. Each weekly session followed a regular schedule of first briefly (~10–15 mins) discussing healthy aging topics, followed by interactive video gaming for the remainder of the session. The first 6 weeks focused on training and developing competence with the Wii system and the Wii Sports games. Beginning in week 7, participants were introduced to new games (e.g., *Boom Blox, Wii Play, and Sports Resort*) for approximately the final 15–30 mins of the session to provide *novel gaming* conditions and to maintain motivation and interest. In weeks 10 and 20, the groups competed in a "Wii tournament" to encourage enhanced effort and social interaction.	
**Schwenk et al.[23]**<br>**2016**<br>**2-arm RCT**	**Intensity:** N/A<br>**Duration:** 4-week<br>**Training load:** 45-min, 2 times/week<br>**Intervention proposal:** The technology used in this study was specifically developed for measurement and improvement of balance control. It consisted of a 24-inch computer screen, an interactive virtual user interface, and 5 inertial sensors (*LegSysTM, BioSensics LLC*, MA, USA) including a tri-axial accelerometer, gyroscope and magnetometer for estimation of joint angles and position. Sensor data were acquired and transmitted at a 100 Hz frequency for real-time feedback in a virtual environment. The sensors were mounted on 2 places (the upper and lower leg) on both legs and on the lower back using elastic straps. The participant stood in front of the screen which was positioned at eye-level. A chair with backrest was in front of the participant to provide support if required. A *supervisor* gave instructions about the exercise tasks during the first training session. In subsequent sessions, subjects conducted exercises based on sensor feedback only; however, the supervisor remained with the participant during all sessions to guarantee safety. Sessions included: 1) *ankle point-to-point reaching tasks* and 2) *virtual obstacle crossing tasks*. The interface was designed to be intuitive and easy to navigate and to avoid complex animations that could distract cognitively impaired persons from observing relevant information related to motion performance and motor error.	Improved postural control
**Liu et al.[24]**<br>**2022**<br>**3-arm RCT**	**Intensity:** 12–14/20 RPE<br>**Duration:** 12-week<br>**Training load:** 50-min, 3 times/week<br>**Intervention proposal:** The infrared light component of *the Kinect system* (Microsoft Corporation, Redmond, WA, United States) was used to capture and track changes in limb segment motion. The system was then used to create a virtual full body 3D map. During the *Tai Chi (TC) exergames* (*LongGood software*), participants imitated a virtually presented *TC coach* and responded to instant feedback by real-time adjustments in movement. The program is also modified from *Yang Style TC* which includes changing standing from wide to narrow base, body mass weight shifting, squats, and slow symmetrical to diagonal coordination arm-leg movements. Movement accuracy scores for each participant were presented simultaneously on the monitor while TC was in progress. Each session consists of *10 mins warm-up, 35 mins main exercise, and 5 mins cool-down.*	1. Better performances in the:<br>a) Executive function<br>b) Attention<br>c) Gait speed<br>d) Dual-tack cost<br>2. More beneficial effects for:<br>a) The global cognition<br>b) Stroop color<br>c) Word test
**Padala et al.[25]**<br>**2012**	**Intensity:** N/A<br>**Duration:** 8-week	Improved:<br>1. Balance

**2-arm RCT**	**Training load:** 30-min, 5 times/week<br>**Intervention proposal:** *Research personnel* set up and navigated *the Nintendo Wii-Fit console and software*, and the subjects performed the exercises. Based on the physical therapist's recommendation, we encouraged patients to do exercises that included *strength training, yoga, and balance games*. The *Yoga exercises included the half-moon, warrior pose, chair, and sun salutation*. *Strength training included single leg extensions, lunges, and torso twists*. *Balance exercises included soccer heading, ski slalom, ski jump, table tilt, balance bubble, and penguin slide*. In the Wii-Fit group, all subjects spent 10 mins on yoga, 10 mins on strength training, and 10 mins on the balance games. They were allowed to rest only if they became unduly fatigued.	2. Gait<br>3. Physical performance
**Liao et al.[26]<br>2021<br>2-arm RCT**	**Intensity:** 50%–75% of HRmax, 13–14/20 RPE<br>**Duration:** 12-week<br>**Training load:** 60-min, 3 times/week<br>**Intervention proposal:** Participants were supervised by 2 *experienced physical therapists* in a small group setting (*3–4* participants). Participants preformed *resistance exercise, aerobic exercise, and Tai Chi and balance exercises* during exergaming. We used *the Kinect system* (Microsoft Corp., Redmond, WA, USA) because of its ability to capture and track changes in limb segment motions with infrared light and to create a full-body 3D virtual map. Exergaming programs developed by *Tano and LongGood* were used in the present study. During these games, participants imitated the virtual character and adjusted their movements in real time according to instantaneous visual and auditory feedback. The participants' *HR and RPE* were also *monitored* during training to ensure consistency between groups.	1. Improved:<br>a) Global cognition<br>b) Executive function<br>c) Attention<br>2. Significant improvements in:<br>d) Verbal/episodic memory<br>e) Working memory
**Karssemeijer et al.[27,28]<br>2019<br>3-arm RCT**	**Intensity:** 65%–75% of HRR<br>**Duration:** 12-week<br>**Training load:** 30-50-min, 3 times/week<br>**Intervention proposal:** Training sessions were given on a *one-on-one* basis, *and trained students or research assistants* supervised the participants. The exergame training consisted of a combined *cognitive–aerobic bicycle training* developed by Bike Labyrinth. The aerobic exercise was tailored to an individual fitness level and health status and aimed to achieve an intensity of 65–75% of HRR after 12 weeks. For participants on medication that attenuates HR (e.g. beta-blockers), the *RPE* was used to ensure that the intended training intensity was achieved. In addition, the *stationary bike* was connected to a video screen. Participants followed a route through a digital environment and simultaneously performed cognitive tasks targeting response inhibition, task switching and processing speed. The exergame training consisted of 7 different cognitive training levels. The difficulty of the cognitive tasks increased per level to ensure that the training remained cognitively challenging.	Improved psychomotor speed
**Santen et al.[29]<br>2020<br>2-arm RCT**	**Intensity:** N/A<br>**Duration:** 24-week<br>**Training load:** 60-min, 2–5 times/week<br>**Intervention proposal:** The exergaming intervention was *interactive cycling using a stationary bicycle* (i.e., home trainer) connected to a screen. While cycling, the participate sees a route on the screen. They can pick a route, and it mimics the experience of cycling outside, thus offering simultaneous physical and cognitive stimulation.	1. Small to moderate positive effects on:<br>a) Cognitive<br>b) Social functioning<br>2. Small positive effects on:<br>c) Distress

		d) Subjective burden<br>e) Sense of competence
**Wu et al.[30]**<br>**2023**<br>**2-arm RCT**	**Intensity:** 60%–70% of HRmax<br>**Duration:** 12-week<br>**Training load:** 35–50-min, 3 times/week<br>**Intervention proposal:** All participants underwent 2 weeks of a familiarization period before starting the intervention. All participants' HRs were measured during exercise using *HR monitors* (*Polar RS400sd*, Madison Height, MI, USA). Participants performed aerobic exercise using *ExerHeart devices* (D&J Humancare, Busan, South Korea) equipped with a *running/jumping mat* (950 width × 1300 depth × 1700 height). We used a game called *Alchemist's Treasure*, which is a running game based on the *Talesrunner IP* co-developed with ExerHeart. During the game, players run with the avatar, avoiding obstacles, and win items while running or jumping at speed on the mat.	1. Lager increases in neural activities that were related to:<br>a) Attention<br>b) working memory<br>2. Greater enhancements in<br>c) Lower body muscle strength<br>d) Cardiorespiratory endurance
**Swinnen et al.[31]**<br>**2021**<br>**2-arm RCT**	**Intensity:** N/A<br>**Duration:** 8-week<br>**Training load:** 15-min, 3 times/week<br>**Intervention proposal:** The exergame device "*DividatSenso*" (Dividat, Schindellegi, Switzerland) was administered. This device consisted of *a step training platform* (1.13 m × 1.13 m) which was sensitive to pressure changes (strain gauges measuring at 50 Hz). The *sensors* detected steps in 4 directions: left, right, top, and bottom. Participants could grasp the bars on waist height, when deemed necessary. Participants that used a wheelchair were assisted by the *guiding therapist* to stand up and walk to the Dividat Senso exergame device. The platform was connected via a USB cable to a computer and a frontal television screen (LG, 94.5 cm × 53 cm, model 43LJ500V, 43 in.) on which the exergames were displayed. The starting position was an upright stance with both feet in the middle of the platform. Participants interacted with the game interface by pushing one foot on one of the four different arrows. When the game required the player to perform a step to the left or right, the associated lower limb was used. For a step in the 2 other directions, the player used a lower limb of preference. The games trained the following cognitive abilities: *divided and selective attention, flexibility, postural control, and visuospatial working memory*. The device provided *real-time visual*, *auditory* and *somatosensory* (vibrating platform) cues, and *feedback* in order to enrich the game experience. The sessions consisted of multiple games and the duration of each video game varied between 120 and 200 s. The following training principles were implemented: *(a) task difficulty was individually adapted to facilitate retention, (b) training variability was provided in order to enhance task transfer, (c) a system of feedback to improve training effects, and (d) individual difficulty zones to match the training level of each participant*. The *physical therapist* designed an individual program for each participant, adapted to the participants' functionality, cognition, and health status. The exergames automatically adapted to the participants' capabilities during active exergaming, i.e., providing more difficult stimuli when the players reacted fast and correct. During the 8-week program, progress was also made regarding the reduction of manual support via the bars. All participants were individually supervised to ensure safety and comfort.	1. Significantly improved:<br>a) Gait speed<br>b) Mobility<br>c) Balance<br>d) Cognitive function<br>2. Significantly reduced symptoms of depression

**Zheng et al.[32] 2022 2-arm RCT**	**Intensity:** N/A<br>**Duration:** 8-week<br>**Training load:** 60-min, 5 times/week<br>**Intervention proposal:** The <ins>*box Kinect system*</ins> was selected for the active game. The game system consists of a Kinect sensor and a console. The console controls the game. First, a participant was asked to sit in a chair that was located 1 to 1.5 m away from the <ins>*Kinect sensor.*</ins> The position of the sensor was adjusted to ensure optimal capture of the person's position and motion by the infrared camera in the sensor. The training was provided by a <ins>*nursing researcher*</ins> in a group of <ins>*5 to 6*</ins> participants. Each participant played the game for 10 mins each time. <ins>*The Kinect game Fruit Ninja*</ins> was selected as the exercise intervention to train the person's hand-eye coordination and motor skills. In the game, a player was faced with a variety of fruits flying in all directions on the screen. The task for the player was to use their upper arm as an imaginary sword to cut the fruits before they dropped to the bottom of the screen. This required the player to focus attention on the fruits amidst distractors, and act rapidly upon viewing the targets. Points were awarded for each successful cut. Bonus points were given if several fruits were cut with one slashing motion. To standardize the length of play, the game was set to last 1 min before a bomb was exploded. A pomegranate would appear at the end of each game. The player could slash it multiple times to win bonus points. If the sword touched the bomb, the player would be penalized 10 points. In each session of the game, a <ins>*researcher accompanied*</ins> a participant to record the person's score and playing time. Motivational suggestions were given, such as paying attention to the score of the last game, trying to beat it and to achieve a personal best. The participants also <ins>*talked and joked with each other*</ins>, sharing experiences of playing and achievements in scores, and <ins>*competing*</ins> with each other in a joyful environment. Upon the appearance of any sign of fatigue, or any abnormalities in breathing, the game play was immediately stopped. To prevent a fall in the process of play, a participant could choose to take the <ins>*standing position or sitting in chair.*</ins>	1. A significant improvement in QoL<br>2. Significantly reduced depression<br>3. No significant change in overall cognitive
**Ugur and Sertel[33] 2025 2-arm RCT**	**Intensity:** N/A<br>**Duration:** 6-week<br>**Training load:** 30-min, 2 times/week<br>**Intervention proposal:** In the first session, each <ins>*Nintendo Wii component*</ins> was introduced to each participant. The <ins>*physiotherapist*</ins> taught each participant how to play the chosen games. In the first session, the games were played with the help of the physiotherapist so that the individuals could transfer the correct weight on the Nintendo Wii balance board and control the game console. In the following sessions, the participant was <ins>*supported with verbal and physical feedback*</ins> when they played incorrectly during the games. <ins>*The selected games' difficulty level was set to the simplest level for each participant.*</ins> The difficulty level of the games was increased as the participants became more successful. The games were played in a certain order. The number of games played in a session varied according to the participant's playing speed. However, at the end of the session, the last game played for each participant was noted, and the participants continued the cycle from where they left off in the next session. Each participant played the game in the same environment and with the same rules. The exercise program was implemented by a <ins>*physiotherapist with 2 years of experience*</ins>.	Reduced fear of falling

**Yoga**		
**Grzenda et al.[34] 2024 2-arm RCT**	**Intensity:** N/A<br>**Duration:** 12-week<br>**Training load:** 60-min, 1 time/week<br>**Intervention proposal:** *The Kundalini yoga (KY)* with a *certificated KY instructor* and each class of *6–10* participants followed the same structure: *(1) tuning in (5 mins); (2) warm-up (15 mins); (3) breathing techniques "Pranayama" (15 mins); (4) Kirtan Kriya (12 mins); (5) final resting pose "Savasana" (10 mins) and closing (3 mins).* In addition, each participant *received a CD* containing a recording with gentle background music and guidance for the exercise sequence. Participants performed this exercise at *home* every day. They were instructed to *chant* along with their eyes closed in a seated position, the feet at on the floor (i.e., relaxed with a straight spine), to visualize a beam of white light entering the center of the top of the head and exiting the middle of the forehead, which is spiritually considered the third eye. While chanting, the thumb of each hand would touch the other fingers sequentially ("mudras") along with the words "Saa" (thumb touches second finger), "Taa" (middle finger), "Naa" (ring finger), and "Maa" (fifth finger). Saa Taa Naa Maa translates to "Birth, Life, Death, and Rebirth". The first round is chanted out loud, the next round whispered, the third is thought silently, the fourth is also whispered, and the fifth round is chanted out loud again. This sequence is repeated for 11 mins with the last minute of energetic integration and meditation (total 12 mins). This technique is thought to engage different senses simultaneously (*visualization, vocalization, motor, and sensory stimulation*).	1. Significant improvements in seriousness of forgetting<br>2. Delayed recall significantly declined
**Khanthong et al.[35] 2021 2-arm RCT**	**Intensity:** N/A<br>**Duration:** 12-week<br>**Training load:** 60-min, 3 times/week<br>**Intervention proposal:** The participated in 2 days of *Ruesi Dadton (RSD)* exercise practice; afterward, they started performing RSD exercise using an *instruction video*. Two *Thai medicine instructors* led RSD practices for over a period of 2 days; the first day involved *breathing exercises and movement corrections*, while the second day *emphasized deep breathing and breath-holding with slow movements while performing RSD*. The video included 15 postures with 10 repetitions for each posture according to the performance guidelines of the *Thai Ministry of Public Health*.	1. A significant improvement in physical function<br>2. Reduced cognitive decline
**Kashyap et al.[36] 2022 2-arm RCT**	**Intensity:** N/A<br>**Duration:** 24-week<br>**Training load:** 60-min, 4-5 times/week<br>**Intervention proposal:** In the first week of intervention, patients were expected to attend *Hatha yoga* sessions for at least 5 days, to enable them to learn the asanas/breathing techniques. Each yoga session was supervised *by trained yoga teacher*. This consisted of *5 mins of breathing exercises, 10 mins of strengthening exercises, 30 mins of simple yoga asanas, 10 mins of pranayama, and 5 mins of meditation*. On first session, a single *10-min lecture* on yoga concepts and possible benefits was described. Thereafter, for the next 11 weeks, the intervention was supervised via *tele-yoga sessions* with the help of recorded video telecast on *Google Meet platform* for at least 1–2 days a week. All patients were encouraged to	Improvements in:<br>1. MoCA<br>2. Frontal Assessment Battery (FAB)

	continue practicing their respective rehabilitation activity for next 3 months with a frequency of at least 4–5 days/week at home. Once-weekly reminders were provided to them with the help of *text messages*.	
**Tremont et al.[37]**<br>**2022**<br>**2-arm RCT**	**Intensity:** N/A<br>**Duration:** 12-week<br>**Training load:** 60-min, 2 times/week<br>**Intervention proposal:** Before the start of the class, the instructor met new participants individually for *30 mins to review* class procedures, discuss any injuries or special needs, and answer any questions. Yoga classes were designed by a *senior yoga teacher*. Two instructors served as primary yoga teachers and each taught one class/week. A third instructor served as a substitute. Instructors were *registered yoga teachers* with the Yoga Alliance (i.e., national credentialing association) with *experience working with older adults*. It used the same set of postures for each class. It was based on *the Hatha style of yoga* but was *very gentle and appropriate* for sedentary older adults. This style of yoga strongly emphasized maintaining awareness and control of the breath as the individual performed physical postures. It incorporated mindfulness (i.e., attending to bodily sensations and thoughts), meditation, and relaxation. In addition, the teaching style was very supportive and flexible. Modifications of each posture were presented using chairs. Use of other props, such as *blocks, straps, and blankets*, was also encouraged. The teacher described and demonstrated the use of props for each pose and provided encouragement and positive affirmations. Participants were encouraged to use modifications, especially during the first few classes or if they had physical limitations. Each class consisted of the following elements: *(1) Pranayama (breathing practices)—active exhalations; (2) Seated Meditation—a brief guided meditation directed participants to focus on breath and body awareness as well as instilling feelings of well-being and acceptance; (3) Half Sun Salutations—a series of flowing yoga postures to warm the body and to prepare for standing postures. These sequences were physically easy and gentle; (4) Standing Postures—a series of standing postures linked together with the breath; (5) Seated Postures—floor or chair postures involving abdominal exercise, gentle forward and back bends and twists; (6) Inversions—basic inversion poses such as legs-up-the-wall pose. These poses were designed to cool and restore the body after the preceding poses and prepare for relaxation; and (7) Shavasana (relaxation)—guided relaxation*. Participants were provided with a *video of the yoga class* to use for practice at home. This video used the same sequence of poses as the in-person class, had 3 mock participants who demonstrated different modifications and was taught by a credentialed instructor. Study participants were encouraged to *incorporate yoga into their daily routine* and practice at least one other time during the week. They were asked to complete *home practice* logs.	1. Medium effect size in the visuospatial domain (vasoconstriction and visual planning and organization)<br>2. The latter score likely reflected an aspect of executive function
**Eyre et al.[38,39]**<br>**2017**<br>**2-arm RCT**	**Intensity:** N/A<br>**Duration:** 12-week<br>**Training load:** 60-min, 1 time/week<br>**Intervention proposal:** *The Kundalini Yoga (KY)* group engaged in a 60-min KY class per week, and a daily 12-min Kirtan Kriya (KK) *meditation* and *homework* assignment. Each 60-min KY was conducted by the same instructor and followed the following structure: *(1) tuning*	1. Enhanced neural activity and structural changes in brain circuits associated with executive function<br>2. Increased "brain fitness" with increased

	in (5 mins); (2) warm-up (12 mins); (3) breath techniques "Pranayama" (12 mins); (4) KK (12 mins); (5) meditation (15 mins); (6) rest "Shavasana" and closing (min). *Standardized handouts and CDs* were given to participants for their 12-min home practice of KK meditation, which includes repetitive finger movements (or mudras), as well as chanting of the mantra "Saa, Taa, Naa, Maa," meaning "Birth, Life, Death, and Rebirth," first *chanted* aloud, then in a whisper, and silently for the total of 11 mins followed by a final 1 min of deep breathing relaxation accompanied by the visualization of light.	connectivity and improved cognitive performance on the tests of:<br>a) Memory<br>b) Executive function<br>3. Along with improvements in:<br>c) Mood<br>d) Apathy<br>e) psychological resilience
**Chinese Traditional Exercise (CTE)**		
**Li et al.[40]**<br>**2022**<br>**3-arm RCT**	**Intensity:** Light to moderate<br>**Duration:** 16-week<br>**Training load:** 60-min, 2 times/week<br>**Intervention proposal:** This intervention consisted of the 8-form routine training protocol. The intervention followed a traditional *Tai Ji Quan* training approach, where *instructors play a dominant and leading role in teaching and practicing*, without instructor-participant interaction during exercise. Accordingly, each exercise session primarily involved teaching participants, via both *verbal and visual cues*, Tai Ji Quan forms and movements through *repeated* practice. The specifics of physical training activities included *controlled, self-initiated Tai Ji Quan-based exercises with synchronized breathing, including center-of-gravity displacement using a dynamic interplay of stabilizing and self-induced destabilizing postural actions involving unilateral weight-bearing and weight-shifting movements, trunk and pelvic rotation, ankle sway, multidirectional stepping, and eye-head-hand movements*. The exercise training in was taught progressively, with learning and practice of the *8* individual forms taking place during the first 10 weeks and repeated practice with variations thereafter.	Enhanced cognitive function
**Sungkarat et al.[41,42]**<br>**2018**<br>**2-arm RCT**	**Intensity:** N/A<br>**Duration:** 24-week<br>**Training load:** 50-min, 3 times/week<br>**Intervention proposal:** Before starting the 6-month home practice, participants in the *Tai Chi* (TC) group learned TC principles and the 10-form TC for 9 sessions (3 times/week for 3 weeks). TC classes with the group size of *6 to 7* participants per class were taught at the *Department of Physical Therapy* by one *certified TC instructor*. A 50-min *video* was provided to assist participants to undertake their TC home exercises. Each 50-mins session included *a 10-min warm-up (range of motion, muscle stretching), 30 mins of TC exercise, and a 10-min cool-down (muscle stretching and breathing exercise)*. Participants were instructed to fill in a *logbook* immediately after exercise to maximize the accuracy of their records. In addition, several *strategies* were implemented to assist with *exercise adherence*, including asking family	1. Improved cognitive performance (specifically memory and executive function)<br>2. Increased plasma BDNF levels

	members to remind participants, posting a note about their exercise schedule in the house as a visible reminder, and administering a weekly *reminder telephone call*. In addition, all participants received a phone call from a research staff member once a week to *monitor health and routine changes* (e.g., attending a recreation club, supplement and medication use, illnesses and hospital admissions). Intervention group members were also asked about the frequency and duration of their TC exercise and any adverse events in these calls.	
**Li et al.[43]**<br>**2021**<br>**2-arm RCT**	**Intensity:** N/A<br>**Duration:** 24-week<br>**Training load:** 60-min, 2 times/week<br>**Intervention proposal:** *Exercise instructors trained by the first author* taught all sessions. An *orientation* on the use of *Zoom* was provided to each participant at baseline. We also constrained the instructor's teaching movements to a square (marked on the floor) not exceeding 4 feet by 4 feet in order to provide (a) onscreen viewability, (b) instructional clarity, and (c) effective home space utilization and movement safety. On the participant end, we set an optimum physical distance of 8-10 feet for personal computer users and 6–8 feet for iPad or smart phone users for better viewability. On each class day, participants were given a *secured Zoom link* via an e-mail to allow them access to the scheduled online exercise session. Participants received *an augmented dual-task Tai Ji Quan* training program based on Tai Ji Quan: Moving for Better Balance, an evidence-based balance training falls prevention program. The protocol involved *10–15 mins of preparatory exercises, 45–50 mins of core training (learning, practicing) in the movements contained in an 8-form routine ("Move a Ball," "Part Wild Horse's Mane," "Repulse Monkey," "Brush Knees," "Fair Lady Works the Shuttle," "Grasp Peacock's Tail," "Waving Hands like Cloud," and "Single Whip"), and 1–2 mins of closing exercises*. Training focused on practicing symmetrical and coordinated forms/movements that emphasized movement and breathing synchronization, trunk rotation, bilateral weight shifts, controlled displacement of the body's center of mass over the varying sizes of the base of support, dynamic eyes-head movements, and multidirectional (anterior-posterior and medial-lateral) stepping. Additional training features involved form-based rotational ankle sway and self-induced reactive postural recovery exercises.	Improved:<br>1. Balance<br>2. Mobility<br>3. Dual-task performances
**Lam et al.[44,45]**<br>**2012**<br>**2-arm RCT**	**Intensity:** N/A<br>**Duration:** 48-week<br>**Training load:** 30-min, 3 times/week<br>**Intervention proposal:** The Intervention group had training on "*24-forms simplified Tai Chi*" by a *Tai Chi master and trained intervention therapists (qualified occupational therapists)*. (1) *Induction phase*: The instruction course for each exercise intervention spanned from 4 to 6 weeks. Instructors conducted regular weekly sessions at the training centers until participants were familiar with the exercise logistics. (2) *Maintenance phase*: After completion of the induction course, subjects were given a *video CD*. The participating centers were responsible for providing the physical settings and arranging the practice sessions with the video CD throughout the study period. The Tai Chi masters provided *refresher lessons* every month until the 12th month to booster adherence and ensure correct performance of posture sequence.	Affected preservation of functional abilities

**Jiayuan et al.[46]**<br>**2022**<br>**3-arm RCT**	**Intensity:** N/A<br>**Duration:** 24-week<br>**Training load:** 60-min, 2 times/week<br>**Intervention proposal:** All participants received *a picture booklet* about induction of Tai Chi Chuan (TCC). *24-Simplified TCC* was conducted. During group intervention, each session began *with a 10-min warm-up (including muscle stretching and joint movement) aiming to avoid injure, and then a 45-min exercises and a 5-min cool-down activity (deep breathing and relaxing)*. Participants were taught how to carry out different TCC forms such as *"Starting Posture", "Hold the Lute", "Cloud Hands" , "Turn and Kick with Left Heel"*, etc. During individual session, participants were required to continue TCC training under the supervision and guidance.	Increased physical function (gait speed, static and dynamic balance, range of motion, reflex control, and core strength)
**Chen et al.[47]**<br>**2023**<br>**3-arm RCT**	**Intensity:** N/A<br>**Duration:** 24-week<br>**Training load:** 60-min, 3 times/week<br>**Intervention proposal:** Participants received supervised *24-form Tai Chi Chuan* training. The intervention focuses on *controlled, self-initialed exercise with synchronized breathing, and the movement patterns include center of gravity displacement, weight bearing and shifting, trunk and pelvic rotation, and eye-hand coordinated movements*. The participants were also encouraged to *continue* exercise after completing their 24 weeks of supervised training, until the 36-week follow-up evaluation. The training was instructed by *certified instructors* with at least 5-year experiences.	Improved:<br>1. Cognitive function<br>2. Attention function
**Lin et al.[48]**<br>**2024**<br>**2-arm RCT**	**Intensity:** N/A<br>**Duration:** 12-week<br>**Training load:** 60-min, 5 times/week<br>**Intervention proposal:** The *Tai Chi* (TC) training was taught by masters with more than 20 years of TC teaching. TC consisted of 24 postures. Each session consisted of *10 mins warm-up, 40 mins core exercises, and 10 mins review of movements and relaxation*. Participants were encouraged to maintain practice by using instructional *DVD* of TC.	Enhanced:<br>1. Memory<br>2. Hippocampus function
**Yu et al.[49]**<br>**2022**<br>**3-arm RCT**	**Intensity:** 13/20 RPE<br>**Duration:** 24-week<br>**Training load:** 60-min, 3 times/week<br>**Intervention proposal:** Participants received the *24-form Yang-style Tai Chi*. Each 1-h training session began with *10 mins of standing pose meditation and Tai Chi relaxation exercises (i.e., stretching with a meditation element), followed by 40 mins of the Yang-style Tai Chi program, and concluding with 10 mins of standing pose meditation and Tai Chi relaxation exercises*. The Tai Chi training sessions were delivered by a *certified Tai Chi instructor*.	A improvement in global cognitive function
**Huang et al.[50]**<br>**2019**<br>**2-arm RCT**	**Intensity:** N/A<br>**Duration:** 40-week<br>**Training load:** 20-min, 3 times/week<br>**Intervention proposal:** *Tai Chi* program under the guidance of the professional *therapists*. The intervention was practiced in groups with 1 therapist to *5 to 8* participants.	Positive effects on:<br>1. Abstraction<br>2. Naming ability<br>3. Depression<br>4. Behavioral<br>5. Psychological symptoms

**Cheng et al.[51,52] 2014 3-arm RCT**	**Intensity:** N/A<br>**Duration:** 12-week<br>**Training load:** 60-min, 3 times/week<br>**Intervention proposal:** For Tai Chi, we used a *seated 12-form Yang style*. All activities were led by an *appropriate instructor affiliated with the research team*, with assistance from student helpers, for the purpose of standardization. Ratio of student helpers to participants was approximately 1:3 across all groups.	Positive effects on short-term memory of numerical unites
**Liu et al.[53] 2018 2-arm RCT**	**Intensity:** N/A<br>**Duration:** 16-week<br>**Training load:** 60-min, 2 times/week<br>**Intervention proposal:** A *10-step simplified Tai-chi* training program was arranged. This 10-step Tai-chi approach was derived from the *traditional Yang style*. This training protocol was modified by the research team by adding strategies to meet the special learning needs and characteristics of people with dementia. Each week, the *dyads* attended center-based Tai-chi training and were asked *to practice at least 3 x 30-min Tai-chi sessions at home*. All of the training sessions were conducted by the same *Tai-chi instructor* to ensure intervention consistency. The *Tai-chi master* started only one step in each session, *gradually adding new steps* according to the progress of the participants. The Tai-chi steps were practiced slowly and in a relaxed manner to promote engagement and awareness of body alignment for participants with poor attention. *Multiple sensory cues*, including *visual cues* (i.e. demonstrations by the Tai-chi instructor and photographs of different steps), *verbal cues, and touch*, were used to aid their learning. A *dyadic* approach involving family *caregivers as exercise partners* was used to provide support and assistance to the participants in practicing Tai-chi during the training sessions and the *home practice*. To prepare the caregivers to take on the role of exercise partner, and to motivate their family member with dementia to practice Tai-chi, all of the caregivers were required to attend 2 *pre-Tai-chi training workshops* run by the principal investigator. *Positive emotional motivation techniques* were taught in the workshops to enhance both the participants' and their caregivers' engagement in and enjoyment of the Tai-chi program. Verbal encouragement and individualized incentives were introduced to the caregivers. To ensure standardization and the adherence of both instructors in delivering Tai-chi training and caregiver workshops according to the protocols, checks of intervention fidelity were conducted weekly during the first month and then monthly during the intervention period based on the workshop/Tai-chi checklists.	There was no significant difference between the groups, however, a trend towards improvement was noted in the experimental group in the Step test
**Chan et al.[54] 2016 2-arm RCT 9**	**Intensity:** N/A<br>**Duration:** 8-week<br>**Training load:** 60-min, 2 times/week<br>**Intervention proposal:** The program included 10 forms selected from the *18 Tai Chi Qigong* (TCQ) movements. The 10 were chosen by an experienced Tai Chi master to emphasize the elements of mind-body coordination. The practice sessions were led by an *experienced and qualified TCQ instructor*, and the subjects replicated her motions, postures, and movement speed. The instructor also reviewed the skill mastery of the participants and rectified any incorrect performances. The number of participants in each session was limited to *less than 10*,	Benefited in the areas of:<br>1. Sleep duration<br>2. Sleep efficiency<br>3. QoL

	which was considered manageable and feasible for teaching and learning. A _research assistant_ was present during each class to monitor the consistency of participants' practice by using a skill competence checklist. The participants were encouraged to _self-practice TCQ every day_. An audiovisual _DVD_ and pictures of the 10 forms, printed on a piece of A3-sized paper, were provided to all participants to help their self-practice and serve as an important reminder for participants to self-practice.	
**Nyman et al.[55,56]**<br>**2019**<br>**2-arm RCT**	**Intensity:** N/A<br>**Duration:** 20-week<br>**Training load:** 45-min, 1 time/week<br>**Intervention proposal:** The intervention group received a _Tai Chi_ intervention comprising 3 components: _(1) Tai Chi classes, (2) home-based Tai Chi practice, and (3) behavior change techniques_. The intervention was designed for participants to accrue 50 hours or more Tai Chi in line with evidence that higher doses of exercise lead to greater reductions in falls. Classes were held in suitable venues (eg, _church halls_). Each session was booked for 90 mins_, with 45 mins instructor-led group Tai Chi followed by up to 45 mins informal discussion_. _Dyads_ were encouraged to participate in the informal discussions each week to foster mutual peer support and provide opportunity for ongoing advice from the Tai Chi instructor in relation to the _home-based practice_. Up to _10 dyads_ were recruited per class. The approach to teaching at each class was the repetition of movements and positive reinforcement. The course was delivered by either a lead instructor with experience in teaching people with dementia or an additional instructor. Both _instructors were experienced in teaching Tai Chi and had qualifications at senior instructor level for public Tai Chi classes_.	Improvements on:<br>1. Time and Go test<br>2. Berg balance<br>3. Postural sway<br>4. QoL
**Okuyan and Deveci[57]**<br>**2020**<br>**2-arm RCT**	**Intensity:** N/A<br>**Duration:** 12-week<br>**Training load:** 35-40-min, 2 times/week<br>**Intervention proposal:** Patients in the intervention group to do _Tai Chi Chuan_ exercises with the help of the researcher.	Positive affected:<br>1. Fall, falls-related safe or protective behaviors<br>2. Physical activity levels<br>3. Kinesophobia
**Su et al.[58]**<br>**2021**<br>**2-arm RCT**	**Intensity:** N/A<br>**Duration:** 12-week<br>**Training load:** 60-min, 5 times/week<br>**Intervention proposal:** _Baduanjin_ exercise including _15 mins warm-up, 40 mins Baduanjin training and 5 mins cool-down_. Baduanjin exercise was guided by 2 _qualified coaches_ who have been in the Baduanjin sport for at least 5 years. To ensure intervention fidelity, 2 coaches who provided the intervention underwent training provided by the investigators. The training scheme of Baduanjin exercise was in accordance with _the Health Qigong—Baduanjin_ published by _the General Administration of Sport of China_, which is mainly divided into _8_ steps: _2 hands support Tianli Sanjiao, left and right bow like shooting sculpture, recuperating spleen and stomach should lift alone, 5 labor and 7 injuries look back, shake head and tail to remove heart fire, 2 hands climb feet to take care of the kidney and waist, save fist to increase strength angrily, and eliminate all diseases in the back._	1. Significant improvements in:<br>a) Memory<br>b) Executive function abilities<br>c) General self-efficacy<br>2. Most of those significant effects sustained in the additional 12-week follow-up

**Zheng et al.[59] 2020 2-arm RCT**	**Intensity:** N/A<br>**Duration:** 24-week<br>**Training load:** 40-min, 3 times/week<br>**Intervention proposal:** The training scheme of *Baduanjin* exercise originates from the '*Health Qigong Baduanjin Standard'* enacted by *the State Sports General Administration* in 2003. The participants were gathered together to train at the same community and guided by the *Qualified Baduanjin exercise coaches* employed from the Fujian university of traditional Chinese medicine.	1. A moderate to high effect on the global cognitive function<br>2. There were small to moderate beneficial improvements for:<br>a) Memory<br>b) Executive ability<br>c) Attention<br>d) Activities of daily living (ADL)
**Zheng et al.[60–62] 2023 3-arm RCT**	**Intensity:** N/A<br>**Duration:** 24-week<br>**Training load:** 60-min, 3 times/week<br>**Intervention proposal:** Each Baduanjin session included: *15 mins warm-up, 40 mins Baduanjin training and 5 mins cool-down.* The training scheme was conducted according to the '*Health Qigong Baduanjin Standard*' published by *the State Sports General Administration of China* in 2003 and consisted of *8* inherent postures as well as the preparation and ending postures. *Professional coaches* were employed to guide and supervise their Baduanjin training.	1. A positive effect in increasing brain gray matter<br>2. Significantly improved:<br>a) Global cognition<br>b) Memory<br>c) Mental control<br>d) Comprehension subscores
**Li et al.[63] 2022 2-arm RCT**	**Intensity:** N/A<br>**Duration:** 12-week<br>**Training load:** 45-min, 5 times/week<br>**Intervention proposal:** *Baduanjin* exercise including 10-min warm-up and finish. The training scheme originated from *Health Qigong-Baduanjin*, published by *the General Administration of Sport of China*. The whole Baduanjin exercise training plan is divided into 2 periods. The main task of the first periods (1 week) as to get the whole set of Baduanjin exercises consisting of *8* postures. Two *professional Qigong Baduanjin coaches* guided them in the first week, until mastering moving and breathing methods. The second periods (11 weeks) were an essential part of the program. During this intervention period, the participants were exercising independently. The coach would guide subjects through a group exercise every week for 1 time during the 11 weeks.	1. A significant improvement in the cognitive function<br>2. Improvements in:<br>a) Lower limb balance function<br>b) QoL
**Luo et al.[64] 2022 2-arm RCT**	**Intensity:** N/A<br>**Duration:** 40-week<br>**Training load:** 60-min, 1 time/week<br>**Intervention proposal:** *Wuqinxi* included *tiger play, deer play, bear play, ape play and bird play*. The Wuqinxi intervention was taught and guided by 2 *professional fitness Qigong coaches*. Each exercise intervention comprised 2.5-min of warm-up, 55-min of Wuqinxi, and 2.5-min of cool-down (relaxation), for a total of 60-min. The first 8 weeks were considered the learning stage. At this stage, the coaches demonstrated the Wuqinxi movements, breaking each move into its components. The participants imitated the movements and practiced them during the coaching sessions while the coaches guided and corrected. At the end of this stage, each	1. A significant improvement in working memory<br>2. A beneficial effect of delaying the deterioration of performance for a simple working memory task

	participant was required to complete the whole set of exercises independently while music played in the background.	
**Chang et al.[65] 2024 3-arm RCT**	**Intensity:**<br>IG¹: N/A<br>IG²: 70%–75% HRR<br>**Duration:** 12-week<br>**Training load:**<br>IG¹: 60-min, 2 times/week<br>IG²: 30-mins, 3 times/week<br>**Intervention proposal:**<br>*Tai Chi Chuan group:* each session was led by a *Tai Chi Chuan master* with over 20 years of experience. The session consisted of a warm-up and the main exercise, and all movements were adapted from the *Yang-style short form*. The *warm-up* session included the bear swing, the Tai Chi walk, and a range of motion exercises. It focused on kinesthetic awareness, mind-body preparation, and stretching, and incorporated high-amplitude and diagonal movements. The main exercise phase encompassed all 24 postures in the Yang-style short form. Movements such as roll back, ward off, push, press, single whip, and others emphasized weight-shifting skills, bilateral coordination, and postural control. The Tai Chi master first demonstrated a movement, then deconstructed it and taught it step by step. To ensure accuracy, the master corrected each participant's movements. One to two new movements were taught in each session, and the entire sequence of learned postures was practiced together to reinforce the connection between the mental tracking and the physical aspect. In addition to the postures, the participants were taught other essential elements of Tai Chi Chuan, such as body orientation, breathing, and relaxation.<br>*Aerobic exercise group:* Before every training session, each participant's resting HR was measured to calculate their heart rate reserve (HRR). The aerobic exercise protocol included a 4 min *warm-up* phase, followed by 24 *min of moderate-to-high-intensity interval aerobic training* (eight cycles of 1 min with 70–75% HRR followed by a 2 min active recovery period), and finally a 2 min cool-down period. The target HR was monitored using a Polar HR monitor (rs800CX, Polar Electro Oy, Kempele, Finland). The participants were verbally encouraged to achieve the appropriate intensity for each phase, and they exercised under the medical supervision of a physical therapist.	Improved motor symptoms and provided neurocognitive benefits
**Gao et al.[66] 2024 3-arm RCT**	**Intensity:** N/A<br>**Duration:** 12-week<br>**Training load:** 50-min, 3 times/week<br>**Intervention proposal:** The sessions were guided by a *physiotherapist* with more than 5 years of teaching experience and appropriate qualifications. All physical therapists held a national advanced tai chi coach certificate. Research assistants monitored participants during the intervention sessions to ensure their safety. The training was performed on Mondays, Wednesdays, and Fridays. Each session included a warm-up and cool-down, and the participants were instructed to slowly stretch at a *comfortable intensity* rather than forcing themselves to perform the movements. They were also advised to focus on their lower body while fully extending their upper body.	Improved physical performance, sleep efficiency, and QoL

**Hsu et al.[67]**<br>**2021**<br>**2-arm RCT**	**Intensity:** N/A<br>**Duration:** 12-week<br>**Training load:** 10-min, 30 times/week<br>**Intervention proposal:** The program included a warmup, core qigong movements, and a cool down. The participants were instructed to relax their body and breathe naturally through the abdomen or chest. The core posture involved standing on both legs with knees slightly bent, arms raised in front of the chest, palms facing one another, and eyes focused into the distance. The maintenance time for each movement depended on how long the participants could maintain each movement. At the end of each movement, participants returned to the standing position and then continued to practice the subsequent movement. A modified Chan-Chuang qigong *guidebook* that included more images and a larger sized font than the standard manual was provided to the participants and their caregivers. The accuracy of the intervention was validated by two experts before the study commenced, and a pilot study with four eligible participants were conducted. In the main study, the researcher explained the movements to each participant in the qigong group individually. Before the movements were performed, the researcher *assessed* the participants' overall health by examining their neck, shoulders, waist, knees, ankles, elbows, and wrists and evaluating their independent standing ability. During the first month of the program, the researcher stood beside the participants to guide and confirm their posture and breathing and ensure a safe and relaxed environment. Thereafter, the participants practiced independently and were accompanied by their *caregiver*. If the participants felt tired during the practice, they could pause before continuing.	Improved muscle strength, muscle endurance, exercise capacity
**Aerobic Exercise (AE)**		
**Tomoto et al.[68]**<br>**2021**<br>**2-arm RCT**	**Intensity:** 75%–90% of HRmax<br>**Duration:** 48-week<br>**Training load:** 30–40-min, 3–5 times/week<br>**Intervention proposal:** The *aerobic exercise* training program was based on each individual's fitness level evaluated with VO_2peak treadmill testing. Exercise intensity, duration, and frequency were *gradually progressed* as participants adapted to their previous exercise level. Specifically, *the program began with 3 exercise sessions/week for 25–30 mins/session at the intensity of 75–85% of HRmax. At week 11, 3 or 4 aerobic exercise sessions/week for 30–35 mins/session were performed. In the week participants performed 3 exercise sessions/week, a high intensity exercise session was introduced which consists of 30 mins of exercise at the intensity of 85–90% of HRmax (e.g., brisk uphill walking). After week 26, participants performed 4 or 5 exercise sessions/week for 30–40 mins, including 2 high intensity sessions*. Each exercise session *included a 5-min warm-up and a 5-min cool-down*.	Reduced:<br>1. Carotid arterial stiffness<br>2. Cerebral flow plasticity<br>Improved:<br>1. Cardiorespiratory fitness<br>2. Executive function
**Shimada et al.[69]**<br>**2018**<br>**2-arm RCT**	**Intensity:** N/A<br>**Duration:** 24-week<br>**Training load:** 90–120-min, 1 time/week<br>**Intervention proposal:** *Golf training* focused on physical, cognitive and social activities. Between *7 and 10* individuals participated in each class at *Club*. One *professional golfer and 4 to 6 staff members* conducted each intervention session. The golf program included 14 practice sessions and 10 golf course sessions. During sessions 1–9, participants engaged in 70 mins of	A significantly improved logical memory function

	Starting New at Golf (SNAG) training. During sessions 7–14, participants practiced at a driving range. Golf course sessions *began with a 10-min warm-up period and stretching exercises, followed by a half-round of golf (100 mins) and a 10-min cool-down period*. The instructors facilitated cognitive activities such as learning swing form and golf rule and encouraged social interactions between the participants during the golf program. Participants also performed *home-based* golf practice each day and were encouraged to continue learning about golf. To improve health behavior, golf trainers lectured the participants on the methods of golf training and ways to self-monitor their regular PA.	
**Donnezan et al.[69]**<br>**2018**<br>**4-arm RCT**	**Intensity:** 60% of HRmax<br>**Duration:** 12-week<br>**Training load:** 60-min, 2 times/week<br>**Intervention proposal:** Each session began with a warm-up and finished with a cool-down. The main body of each session was focused on *aerobic training on bikes* (Domyos VA 300). Sessions were supervised by a *physiotherapist*. For participants who took HR attenuating medication, we used the modified formula (i.e., FCe = FCr + 0,8 (FCmax-FCr)), to target moderate aerobic intensity. Thus, for all participants, the difficulty and duration of the exercises were regularly increased over the 12 weeks. The *HR was monitored* objectively using *Polar FT2 monitors* to match the capacities of each individual.	Improved:<br>1. The performance of executive tasks<br>2. Motor test performance
**Baker et al.[70]**<br>**2010**<br>**2-arm RCT**	**Intensity:** 75%–85% of HRR<br>**Duration:** 24-week<br>**Training load:** 45–60-min, 4 times/week<br>**Intervention proposal:** Participants maintained a constant diet and extracurricular activity level for the duration of the study. Most exercise sessions (90%) were conducted *at local Young Mens Christian Associations* (YMCA). The first 8 sessions were supervised by the *trainer*. Thereafter, the trainer supervised 1 session/week per participant. Subjects also *received a weekly call* from the study coordinator to ensure compliance and completed *daily logs* tracking exercise duration and *HR monitor* measurements. Compliance data were reviewed weekly by an *exercise physiologist*. Exercise duration and intensity were gradually increased over the first 6 weeks of the program until participants were exercising at target HRR using a *treadmill, stationary bicycle, or elliptical trainer.* This intensity was then maintained for the study's duration. The *treadmill* was the most commonly selected machine, regardless of sex.	Improved cognitive function, in particular, for executive control abilities (selective attention, search efficiency, processing speed, and cognitive flexibility)
**Ihle-Hansen et al.[71,72]**<br>**2019**<br>**2-arm RCT**	**Intensity:** 15–17/20 RPE<br>**Duration:** 72-week<br>**Training load:** 30-min, 7 times/week<br>**Intervention proposal:** The intervention group received regular *individualized coaching* performed by *physiotherapists*, aiming to achieve *PA 30 mins daily, and 40–60 mins physical exercise including 2–3 bouts of vigorous activity every week*. The physiotherapists carried out *home visits* to provide education and tailored training programs, based on the patient's preferences and goals. The patients were encouraged to report their activities in standardized training *diaries*. The patients had a monthly meeting with their coach, going through training diaries, with reassessment according to needs. These meetings were face-to-face in the first 6 months. For the next 6 months, every second meeting could take place as a *phone meeting*. During the final 6 months, 4 of the 6 meetings could take place as a phone meeting.	No clinically relevant effect on cognitive or emotional function
**Nagamatsu et al.[73,74]**<br>**2013**	**Intensity:**<br>Aerobic Training (AT): 70%–80% of HRR<br>Resistance Training (RT): 7 RM	1. Improved verbal memory<br>2. Spatial memory performance appeared to

**3-arm RCT**	**Duration:** 24-week<br>**Training load:** 60-min, 2 times/week<br>**Intervention proposal:** Classes began 1 month after baseline assessments and were held at a *fully equipped gym* in a research center. Classes were led by *certified fitness instructors* who received additional training from the study investigators. The classes included *10 mins warm-up, 40 mins of core content, and 10 mins cool-down*. Strategies were implemented to promote participant engagement.<br>*Aerobic Training (AT)*<br>The AT program was an *outdoor walking* program. The intensity of the training stimulus was at approximately 40% of one's age specific target HR (i.e., HRR) and progressed over the first 12 weeks to the range of 70–80% of HRR. Exercise intensity was monitored through *HR monitors*. Participants also monitored the intensity of their workouts by the *RPE* and the *"talk" test*.<br>*Resistance Training (RT)*<br>For the RT program, both a *Keiser Pressurized Air system* and *free weights* were used. The Keiser-based exercises consisted of *biceps curls, triceps extension, seated row, latissimus dorsi pull downs, leg press, hamstring curls, and calf raises*. The intensity of the training stimulus was at a work range of 6 to 8 repetitions (2 sets). The training stimulus was subsequently increased using the 7RM method (when 2 sets of 6 to 8 repetitions were completed with proper form and without discomfort). Other key strength exercises included *Mini squats, Mini lunges, and lunge walks*.	be positively associated with physical performance in the aerobic training group<br>3. Both types of exercise improved reaction times during the spatial memory tests
**Tsai et al.[75]**<br>**2019**<br>**3-arm RCT**	**Intensity:**<br>Aerobic Exercise: 70%–75% of HRR<br>Resistance Exercise: 75% of 1RM<br>**Duration:** 16-week<br>**Training load:** 40-min, 3 times/week<br>**Intervention proposal:**<br>*Aerobic Exercise (AE)*<br>Participants in the AE group attended 3 supervised exercise sessions on either *a bicycle ergometer or a motor-driven treadmill* (Med-track ST55, Quinton Instrument Company, United States) according to the participant's lower-extremity function, with the exercise intensity corresponding to 50–60% of the individual target HRR during the first 2 weeks and 70–75% of the HRR for the remainder of the program. Each aerobic exercise session involved *a 5-min warm-up period, followed by 30 mins of continuous bike riding or brisk walking/jogging at an intensity that would maintain the HR within the assigned training range, followed by 5-min of cool-down*. A *Polar HR monitor* (RX800CX, Finland) was used to monitor each participant's HR during the exercise.<br>*Resistance Exercise (RE)*<br>The participants in the RE group attended supervised exercise sessions with the use of *free weights and bodybuilding machines*, with exercise intensity corresponding to 60–70% of the individual target 1-RM during the first 2 weeks and 75% of their target 1-RM for the remainder of the program. Each RE session involved *a 5-min warm-up period (e.g., slow-paced walking and active mobility exercises for the joints of the 4 limbs), followed by 30-min of core content, followed by 5-min of cool-down*. The core RE content consisted of the following circuit-exercise schedule: *biceps curls, vertical butterflies, leg presses, seated rowing, hamstring*	1. AE intervention significantly improved:<br>a) BMI<br>b) Lower-extremely strength<br>c) Cardiorespiratory fitness<br>2. RE significantly enhanced muscular strength in the upper and lower extremities<br>3. The BDNF level was significantly increased, and the level of insulin, tumor necrosis factor-alpha (TNF-α), and IL-15 were significantly decreased in the AE group;<br>4. The RE group showed significantly decreased IL-15 levels, pointing at a

	curls, and calf raises. The participants performed the RE at the target intensity for 3 sets of 10 repetitions with a 90-second rest between sets, and a 2-min interval between each apparatus.	distinct regulation of the molecular biomarkers by the two exercise modes
**Hsu et al.[76–80]**<br>**2017**<br>**2-arm RCT**	**Intensity:** 60%–70% of HRR<br>**Duration:** 24-week<br>**Training load:** 60-min, 3 times/week<br>**Intervention proposal:** *Aerobic walking* included *a 10-min warm-up, 40-min of walking and a 10-min cool-down*. Participants were monitored using *HR monitors, RPE, the 'talk test' and pedometers*; exercise intensity was progressed to the range of 60% to 70% of HRR, after which this was sustained for the remainder of the intervention period. The aerobic training group also received *monthly educational materials* about Vascular Cognitive Impairment and healthy diet.	1. Significantly improved behavioral performance on the flanker task<br>2. Improved executive functions performance<br>3. Significantly reduced task-related neural activity in the right superior temporal gyrus and left lateral occipital cortex
**Song et al.[81]**<br>**2019**<br>**2-arm RCT**	**Intensity:** 12–14/20 RPE<br>**Duration:** 16-week<br>**Training load:** 60-min, 3 times/week<br>**Intervention proposal:** The intervention was an *aerobic stepping* exercise program (20 participants/group). The overall exercise program was designed by a team comprising *a nurse academician*, *a physiotherapist and exercise physiologists* in accordance with the recommendations of the *ACSM* position stand on exercise and PA for the elderly. The moderate-intensity aerobic exercise training was *conducted by 2 registered nurses, one serving as an instructor and the other as a facilitator*, in accordance with a standardized intervention protocol. The 2 registered nurses attended a 4-week training, performed a return demonstration and exhibited good protocol compliance. For the *aerobic exercise training*, each session started with *a 10-min warm-up, which included walking and stationary stretching exercises for trunk and limb joints at the upper and lower bodies, followed by moderate-intensity stepping exercises*. *A stepping exercise program* that involved patterns of stepping up and down on a 10-cm-high stable stepping bench was adopted. Additionally, various sets of simple and entertaining upper limb movements *resembling daily activities*, such as washing the face, were added to enhance exercise motivation and to facilitate overall body movement. The aerobic exercise program was introduced in a *progressive* manner with the duration gradually increasing from 20 mins to the targeted 40 mins in 4 weeks' time to enhance adherence and avoid injury risks. Participants performed the stepping exercise in multiple bouts of at least 10 mins each rather than in a single continuous bout to reach the targeted duration, with a maximum of 5 mins of rest in between exercise bouts. Each training session *ended with a 10-min cool-down session with the same walking and stationary stretching exercises as those in the warm-up session*. The training was in accordance with the *ACSM* safety guidelines. BP, HR and contradictory symptoms were assessed before training was performed. *Motivational strategies*, including goal setting, verbal encouragement and emotional incentives based on the self-efficacy theory, were incorporated into this exercise intervention to ensure compliance. A practice *log* was also provided to the participants to allow self-monitoring.	Positive effects on:<br>1. Cognitive function<br>2. Depressive mood<br>3. Poor sleep quality<br>4. Preventing disease deterioration

**Stuckenschneider et al.[82] 2021 3-arm RCT**	**Intensity:** 13/20 RPE<br>**Duration:** 48-week<br>**Training load:** 45-min, 3 times/week<br>**Intervention proposal:** Participants *performed indoor and outdoor walking and running exercises*. Participants attended supervised instructor led classes and completed unsupervised *home exercises*. Participants were asked to participate in a supervised exercise session at least once a week. Exercise *diaries*, which were collected by the class instructors during supervised exercise sessions once a week, were also used to record unsupervised exercise sessions.	A medium effect size effect on physical fitness
**Rojasavastera et al.[83] 2020 3-arm RCT**	**Intensity:** N/A<br>**Duration:** 8-week<br>**Training load:** 65-min, 2–3 times/week<br>**Intervention proposal:** Participants in the *aerobic observation with gait training* group received the training program from an experienced *physiotherapist*. Training sessions comprised *observation (5 mins), warm-up (5 mins), gait training (40 mins), cool-down (5 mins), and stretching (10 mins)*, consecutively. For the observation section, the participants watched a *video* of walking acted out by a normal healthy individual. The actor walked at the speed of 120 beats/min identified by a metronome and stepped on markers placed on the floor 60 cm apart for each step.	Beneficial effects on:<br>1. Gait<br>2. Cognition improvement
**Makino et al.[84] 2021 4-arm RCT**	**Intensity:**<br>IG[1]: 15/20 RPE<br>IG[2]: 70% of HRR<br>**Duration:** 26-week<br>**Training load:** 60-min, 2 times/week<br>**Intervention proposal:** Participants in the *aerobic exercise training* (AT), resistance exercise training (RT), and combined exercise training (CT) groups exercised under the supervision of *well-trained fitness instructors*. The classes consisted of 3 parts: *10 mins warm-up, 40 mins of core training activities, and 10 mins cool-down*.<br>*Aerobic exercise training protocols*<br>This program comprised *40 mins of core content including 10–15 mins of step-in-place exercises, 10–15 mins of a walking program*, and intervals for rest and *monitoring HR*. To achieve the target HR, exercise intensity was increased after each monitoring interval until the HRR was achieved. The target HR zone was 40% HRR for weeks 1–2, 50% HRR for weeks 3–8, 60% HRR for weeks 9–12, and 70% HRR for the remainder of the program, regardless of the participants' demographic characteristics, medication, physical functions at the baseline, and prior levels of exercise experience. Resting HRs at the baseline were measured with an *automated BPsphygmomanometer*, and HRs before, during, and after workouts were measured using *wearable devices*. Aside from the structured program in class, *outdoor walking was recommended* to the AT group. The participants in the AT group were asked to keep a *notebook* where they recorded the amount of time spent walking, their HR before and after exercise, and their daily total steps according to a *pedometer*.<br>*Resistance exercise training protocols*<br>This program involved *elastic resistance training and bodyweight exercises*. Two types of *elastic tubes*, each with a different stiffness, were given to each participant. Participants were asked to advance their effort in a manner consistent with the progression identified above by	1. AT group showed the significant improvements in:<br>a) Delayed memory function<br>b) Physical activity (average daily step number and moderate intensity activity time)<br>2. All three treatment groups showed improvement in:<br>a) Strength<br>b) Gait speed<br>c) Basic mobility skill

	changing the grip width or rubber stiffness of the elastic tubes to achieve a greater resistance. Elastic resistance training consisted of _bicep curls, chest presses, side raises, seated rowing, leg presses, hip abduction, and side bends_. Body weight exercises consisted of _shrugs, knee ups, trunk curls, squats, kneeling kickbacks, toe raises, and calf raises_. Two sets of each exercise were performed with 10 repetitions each. Aside from the structured program in class, _home-based exercise was recommended_ to the RT group. The participants in the RT group were asked to keep a _notebook_ where they recorded the amount of time spent on home-based exercise, the number of times they completed the training sessions, and the _RPE_.<br>_Combined exercise training protocols_<br>This program _combined the AT and RT programs_. The progression of exercise intensity was the same as that in the AT and RT program, while the amount of time spent on each type of training was _halved_. For AT, step-in-place exercises or the walking program alternated between each class. For RT, each exercise was performed for one set. RT programs were performed before AT programs at each session. Aside from the structured program in class, both _outdoor walking and home-based exercise_ were recommended to those in the CT group.	
**Brydges et al.[85]**<br>**2020**<br>**3-arm RCT**	**Intensity:** 70%–80% of HRmax, 13–15/20 RPE<br>**Duration:** 24-week<br>**Training load:** 60-min, 2 times/week<br>**Intervention proposal:** The exercise classes began 1 month after baseline assessments. All conditions were run as group sessions conducted by _certified fitness instructors_ who received additional training from the study investigators, in a _gym_ in a research center. The classes consisted of _10 mins of warm-up, 40 mins of the training, and 10 mins of cool-down_. In the AE group, participants completed an _outdoor walking program_. Training intensity began at 40% of the participant's age-specific target HR and increased over the first 12 weeks to 70–80%. Exercise intensity was monitored through _HR monitors_, and participants subjectively rated the intensity of their exercise sessions with the _RPE_. Additionally, participants were asked to increase their walking speed, if they could, when they were able to speak comfortably during the session. In the RE program, exercises were performed using _both a Keiser Pressurized Air system (Keiser, Fresno, CA) and free weights_. In all groups, participants were asked to disclose any adverse effects (i.e., pain or discomfort), at session. Instructors monitored participants for shortness of breath and angina during the classes.	Improved of cognitive plasticity
**Morris et al.[86]**<br>**2017**<br>**2-arm RCT**	**Intensity:** 60%–75% of HRR<br>**Duration:** 26-week<br>**Training load:** 150-min/week, 3–5 sessions<br>**Intervention proposal:** Participants were asked not to alter current PA other than those prescribed by the study team. The participants began the intervention with a weekly goal of 60 mins in Week 1 and increased their weekly exercise duration by approximately 21 mins/week until they achieved the current public health recommended target duration of 150 mins/week, distributed over 3–5 sessions. Target HR zones were gradually increased from 40–55% to 60–75% of HRR based on resting and HRpeak during cardiorespiratory fitness testing. _HR was monitored_ by conventions _chest worn sensor_ (F4 or FT4, Polar Electro, Inc. Lake Success, NY). _Exercise trainers_ supervised all exercise sessions during Weeks 1–6 and gradually reduced supervised sessions to 1/week based on perceived ability to be safe and independent and in consultation with the participant's study partner and study staff. Study staff also performed bi-weekly visits to monitor fidelity to the intervention protocols.	1. Benefits in functional ability<br>2. Improvements in cardiorespiratory fitness were related to benefits in memory performance and brain volume change

**Wei and Ji[87]**<br>**2014**<br>**2-arm RCT**	**Intensity:** 60% of HRmax<br>**Duration:** 24-week<br>**Training load:** 30-min, 5 times/week<br>**Intervention proposal:** The handball training program contained following activities: (1) *toss training*: tossing a ball (or walnut instead) up with one hand and caught it with the other hand and practiced it repeatedly; (2) *hit training*: hitting a Ping-Pong ball with table tennis racket without dropping it down on the ground and counting the times f hitting (except dropping) by participants themselves. (3) *Bounce training*: forming groups of 2 or more, one participant patting a ball twice first and bouncing it to the other who should do the same thing; (4) *pass training*: all participants formed like a circle and the ball holder passed the ball to the named person; (5) *grab training*: scooping the table tennis balls into a bowl with a spoon, and the one who had the most balls in his/her bowls was the winner; (6) *field going training*: standing at a designated position and shoot a ball into specified basket by hitting it to the ground; (7) *roll training*: rotating two balls of different colors in one hand by fixing eyes on one ball only throughout the training; (8) *pinch training*: pinching beans into a narrow-mouth bottle with fingers and counting the number. The materials for the training were provided by the *researchers*. Subjects in the training group were divided into 2 groups with *15* participants in one group. The 2 groups exercised respectively under the supervision of the well-trained nurses. Two *physiotherapists* involved in geriatric rehabilitation and 3 *well-trained nurses* conducted each intervention. Each supervised session *began with "toss training" for 5 min to warm the participants up, followed by 25 min of other training programs. The other 7 training programs in addition to "toss training" were taken by turns over the 6 months training*. In addition to "toss training", *2 training programs were taken per day in the first 2 weeks for each supervised session and 4 programs per day for the rest of 6 months as they were getting easier for the participants*. The *HR was monitored* after aerobic exercise each session to take their pulse. Before and after each session of the program, physiotherapists conducted a health check of each subject. The physiotherapists and well-trained nurses provided ongoing *safety monitoring* to prevent adverse accidents such as falling during the program.	Improved cognitive function
**Damirchi et al.[88]**<br>**2017**<br>**4-arm RCT**	**Intensity:** 75% of HRmax, 13–15/20 RPE<br>**Duration:** 8-week<br>**Training load:** 30–60-min, 3 times/week<br>**Intervention proposal:** The physical training consisted of 2 sections: *5 mins warm-up followed by 6 mins walking with 55% HRR in the first session, incrementally reaching to 20 mins with 75% HRR at the eighth week; and muscular strength, range of movement (MSROM) Silver Sneakers*. The participants were being monitored throughout the session by a *HR meter* to guarantee the proper intervention zone. The MSROM Silver Sneakers section also involved a complex of body movements in a group fitness setting, following a similar 5-min stretching protocol. Equipment used here consisted of *chair, ball, 2 to 3 lb hand weights, and a resistance band with handles*. Participants should mimic the motions of an instructor and complete muscular range of motion exercises coupled with light cardio.	No significant change
**Nakatsuka et al.[89]**<br>**2015**<br>**3-arm RCT**	**Intensity:** 13–15/20 RPE<br>**Duration:** 12-week<br>**Training load:** 60-min, 1 time/week<br>**Intervention proposal:** Intervention consisted of 12 group sessions and 12 home assignments. *One instructor and 2 assistants* attended each session. Each session was interrupted by a tea *break* (5 mins). Instructions about the tasks for the *home assignment* were given at the end of each group session (5 mins). The home assignments were designed according to the	A positive effect on physical ability

	intervention methods and contained tasks that require about 1 hour to be completed. *Written instructions* were also given to the participants as an aid to successfully complete the home assignments. The sessions were held in *public halls or community centers* of the regions of living of the participants. The *home assignments were instructed to be done with the families* of the participants. Each session and home assignment consisted of 6 sets of exercises of 3-5 mins of duration that were done every 10 mins. The exercises were composed of walking and step aerobics using *STEPWELL 2* (Konami Sports & Life Co. Ltd., Tokyo, Japan).	
**Kohanpour et al.[90]**<br>**2017**<br>**4-arm RCT**	**Intensity:** 75%–85% of HRR<br>**Duration:** 12-week<br>**Training load:** 39-min, 3 times/week<br>**Intervention proposal:** The first exercise session *included 8-min warm-up and 8-min running, and 5-min cool-down*. After each 2 successive sessions, 1 min was added to the very later session. Therefore, the *running* span reached 26 mins at the end of the 12-week exercise period.	Significant increases in:<br>1. Cognitive state<br>2. Serum BDNF level
**Karthikeyan T[91]**<br>**2020**<br>**2-arm RCT**	**Intensity:** 60%–70% of HRmax, 11–14/20 RPE<br>**Duration:** 8-week<br>**Training load:** 40–50-min, 7 times/week<br>**Intervention proposal:** *Aerobic intervention* included home based aerobic program consisted of 3 components: *warm-up session for 10 mins, aerobic brisk walking program for 20 to 30 mins, and cool-down session for about 10 mins*. Warm-up and cool-down session included stretching exercises and gentle active range of motion exercises to larger muscle groups like lower limb and trunk muscles. The exercise was given based on principle of exercise prescription which consists of intensity, frequency, type and duration of exercises. Initial session of exercise prescription and training was given under supervision and then *the home program protocol* was given to the all the subjects to be performed at home. Follow up was done through *phone calls* and the subjects were visited once in a week. Progression was made by increasing the duration depending on subject's adaptation to the exercise. As the subjects became more conditioned to the exercise program, *progression* of the exercise intensity was done as per their needs. For the first 4 weeks exercise training duration 20 mins and from 5–8-week duration was increased from 20–30 mins. All the subjects were given an *exercise logbook* which included information pertaining to pulse rate, perceived exertion, frequency, duration, exercise protocols and type of exercise performed. Subjects were educated regarding *signs* and symptoms to be monitored while doing exercise programs and the do's and don'ts and the termination criteria for the exercise were explained property to them.	The increases in cognitive functions
**Krootnark et al.[92]**<br>**2024**<br>**3-arm RCT**	**Intensity:** 9–13/20 RPE<br>**Duration:** 12-week<br>**Training load:** 35-min, 5 times/week<br>**Intervention proposal:**<br>The *aerobic group* performed a *home-based aerobic exercise program* at low intensity with an exhaustion level of ≤13 points on the Borg scale, and they later progressed with increasing number of exercises, number of repetitions and complexity of the exercises.<br>The *resistance group* was subjected to a *home-based resistance training program* with an intensity appropriate to each participant's fitness level, according to bodyweight, *water bottles*	Both exercises improved:<br>1. Physical performance<br>2. Cognitive domains<br>3. The perceptual-motor function domain

	or items available at home, and subsequently progressed through increasing resistance, number of exercises, number of sets or repetitions and complexity of the exercises. Before beginning their home-based exercises, participants in both groups received an *explanation* of the training program; this guaranteed that the exercises could be completed safely and correctly when performed alone at home, and each group received a different exercise *handbook*. The *RPE* was also explained so that participants could accurately rate themselves and as a recheck on their level of exercise intensity. The participants received weekly *phone monitors* to encourage exercise and inquire about any problems that might occur.	
**Liu et al.[93]**<br>**2020**<br>**2-arm RCT**	**Intensity:** 5–6/10 RPE<br>**Duration:** 4-week<br>**Training load:** 30-min, 5 times/week<br>**Intervention proposal:**<br>The *strength training program* involved the use *of isotonic weight training machines* targeting *the biceps, triceps, and pectoralis* major over the course of 1 day and the *gluteus and quadriceps muscle* the following day. The weight used during this training was 40–50% of 1-RM, with the program constituting 2 sets of 12 repetitions in 1 day.<br>The *aerobic training program*, on the other hand, involved *stationary bicycle* training. Participants were asked to keep their pedaling rate at an intensity level of 5–6/10 scale of *RPE* during their 30-min sessions on the stationary bicycle. The exercise programs of the study were designed according to recommendations from the *ACSM* and were executed under the supervision of a *certified fitness trainer*.	Both training programs brought about significant benefits for:<br>1. ADLs<br>2. Cognitive performance
**Dillon and Prapavessis[94]**<br>**2021**<br>**2-arm RCT**	**Intensity:** N/A<br>**Duration:** 10-week<br>**Training load:** 30-min, 7 times/week<br>**Intervention proposal:** The intervention consisted of interrupting sedentary behavior and substituting it with a 10-min bout of light PA (*walking*) 3 times/day. The intervention group was given a *digital sports watch* (WobL vibrating watch, Knoxville, TN). Each watch was programmed to go off (15-s vibration) at 3 different times throughout the day, which functioned as the participants' prompt to start their 10 mins of light PA. The watch was also programmed to go off once the 10 mins were up, indicating that they could stop their exercise. The participants were not explicitly asked to partake in moderate to vigorous PA (MVPA); they were instructed to get up and walk around until the watch vibrated a second time. The participant was prompted to do their 10-min bouts approximately 30 mins *after each meal* (breakfast, lunch, and dinner) was completed. The intervention participants were also given a diary and were asked to document all activities performed, as well as any adverse events, defined as any physical or psychological symptoms that occurred any time after randomization. The participants with higher MMSE score (21+) were able to write in the *diary* themselves, and the other participants had personal support workers write in the diary for them.	Improved:<br>1. Cognitive function<br>2. Physical function<br>3. Overall QoL
**Yang et al.[95]**<br>**2015**<br>**2-arm RCT**	**Intensity:** 70% of HRmax<br>**Duration:** 12-week<br>**Training load:** 40-min, 3 times/week<br>**Intervention proposal:** *Cycling training* was performed in the *rehabilitation clinic* of Jiangsu Province Hospital. Each training consisted of *5 mins warm-up, 30 mins target intensity exercise, 5 mins reorganization movement*. The training time of the initial stage was 25–30	Improved:<br>1. Cognitive function<br>2. Mental status<br>3. Self-care ability

	mins, and the exercise load was 0.5kg·M. Then according to patient tolerance gradually increased training time and training intensity. One week later, the training time was increased to 40 mins (mainly increased the strength of the target intense exercise time, warm-up and reorganization movement were unchanged), cycling load was increased to 1.0kg·M (to reach the target HR) and subsequently maintained the training intensity to the end. The training progress was supervised by *therapists*.	
**Choi et al.[96,97]**<br>**2018**<br>**2-arm RCT**	**Intensity:** N/A<br>**Duration:** 6-week<br>**Training load:** 60-min, 2 times/week<br>**Intervention proposal:** A *Ground Kayak Paddling* (GKP) exercise was developed by modifying kayaking actions as performed for moving across water. The GKP exercise was conducted on the ground, and each session consisted of *10 mins of warm-up activities, 40 mins of GKP exercise, and 10 mins of cool-down activities*. The warm-up and cool-down activities were massage with a sensory ball, gentle stretching, and deep breathing exercises. The GKP exercise was a *group exercise* performed while sitting on chairs with and without a *balance foam* (soft blue, Thera-Band, USA), which increases the challenge by providing an unstable surface. A 2-min break was included in the exercise program to avoid muscle fatigue. The *break* was placed between the first and second parts of the training session, and each participant walked around the room while making a light paddling motion. One instructor led the program, and 2 assistants supervised and corrected the posture of the subjects. The participants learned each motion during the first week of training. When the instructor demonstrated the 5 types of exercise to participants, they followed the motions. Each type of exercise was conducted 4 times with 2 sets each time, with *rhythmic music* for interest.	Improved:<br>1. Postural balance<br>2. Muscle performance<br>3. Cognitive function
**Yu et al.[98]**<br>**2022**<br>**5-arm RCT**	**Intensity:** N/A<br>**Duration:** 12-week<br>**Training load:** 1 or 3 times/week<br>**Intervention proposal:** The *walking exercise training* was implemented under the supervision of *research personnel* in the *research center*. The intervention was conducted *individually* and there was *no social interaction* between the participants. The exercise intensity was set at 3.5 METs for moderate-intensity walking groups and 7 METs for vigorous-intensity walking groups in this study. The overall duration of the exercise sessions was set at 150 mins for *moderate-intensity walking groups* (150 mins each session in M1 and 50 mins each session in M3) and 75 mins for *vigorous-intensity walking groups* (75 mins each session in V1 and 25 mins each session in V3). Before each session, there was *a 5-min warm-up period*, in which participants performed static stretching exercises of the major muscle groups including chest, back, and lower limbs. Participants were then instructed to walk on a treadmill with the speed and inclination gradually increased to achieve the target HR range within 5 mins. The target HR was calculated according to the linear relationship between HR and VO_2 recorded in the VO_2max treadmill test (Achten and Jeukendrup 2003). Participants were required to maintain the HR within the target HR range. A validated *HR monitor* (*Polar* OH1) was used. At the end of each session, there was *a 5-min cool-down period*, in which the speed and inclination of the treadmill was gradually decreased, followed by static stretching exercises. As the duration of	Walking interventions at moderate and vigorous intensities and at once-a-week and thrice-a-week frequencies had a similar effect on improving global cognitive performance

	session in M1 and V1 were longer, participants were allowed 2 x 10-min *breaks* to rehydrate and rest during the training sessions. The weekly exercise volumes of the 4 walking exercise groups were equivalent (525 MET-Mins).	
**Fischbacher et al.[99]**<br>**2020**<br>**3-arm RCT**	**Intensity:** N/A<br>**Duration:** 48-week<br>**Training load:** 60-min, Dalcroze eurhythmics: 1 time/week; Simple home exercise program: 3 times/week<br>**Intervention proposal:**<br>The *Dalcroze eurhythmics* classes were taught by an *experienced instructor*. The exercises included different courses of motion, sometimes in combination with the handling of objects (e.g., *a ball or claves*), performed to the rhythm of piano music. The complexity of the exercises gradually increased during a session, starting with single tasks and then combining them into multi-task exercises.<br>The *simple home exercise* program was included 5 components: *sit-to-stand, single leg stance (balance component), stair climbing (functional mobility), pull back, and external shoulder rotation against elastic resistance*. It was instructed by a *physiotherapist* at the baseline visit and adapted to individual needs if necessary. Participants were provided a *booklet* with a detailed description and illustration of the program with the recommendation to do the exercises 3 times a week.	No difference between groups
**Khattak et al.[100]**<br>**2022**<br>**2-arm RCT**	**Intensity:** 11–14/20 RPE<br>**Duration:** 6-week<br>**Training load:** 40–50-min, 5 times/week<br>**Intervention proposal:** Aerobic program included *10-min warm up, followed by 20–30 mins aerobic walk and cool-down of 10 mins*. Warm-up and cool-down included stretching and active range of motion exercise for lower limb and trunk muscles. Participants walked on *treadmill* at a constant pace with zero inclination.	Improvements in neurocognitive domains
**Varela et al.[101]**<br>**2011**<br>**2-arm RCT**	**Intensity:**<br>Group A: 40% of HRmax<br>Group B: 60% of HRmax<br>**Duration:** 12-week<br>**Training load:** 30-min, 3 times/week<br>**Intervention proposal:** Three groups were created: groups A and B performed *aerobic exercise* at different intensity levels, while group C did not exercise but carried out recreational activities instead (*playing cards, reading newspapers, handicrafts*). With the aim of improving the comparability of the groups the sample was distributed in 4 strata. Groups A and B carried out the same aerobic exercise program except that exercise intensity was set at *40%* of participant's HRR in the first group, while in the second one they exercised at *60%* of it. The exercise program supervised by a *physiotherapist* while the patient exercised individually. The training included *a 5-min warm-up session, 20 mins of cycling in a recumbent bike at the established intensity for each group and a 5-min stretching routine to cool-down*. HR was measured by means of an *ergonomic hand pulse sensor* that registered the patient's thumb pulse and reflected it on a screen placed in the middle of the handlebars. Before the beginning of the intervention, 2 weeks were dedicated to *familiarizing* the participants with the exercise routine and the *recumbent bike*. Cycling time was gradually increased from the first sessions in order to reach 20 mins of continuous exercise per session.	1. Both exercises seemed to slow cognitive deterioration slightly and somehow improved functional level<br>2. There was no difference between the effects of three months of aerobic training whether the intensities
**Miu et al.[103]**<br>**2008**	**Intensity:** N/A<br>**Duration:** 12-week	Significantly improved physical performances

**2-arm RCT**	**Training load:** 45-60-min, 2 times/week<br>**Intervention proposal:** Aerobic exercise training included: *treadmill, bicycle and arm ergometry* (carried out in our *geriatric day hospital*), and 10-min flexibility training (performed at the start of each session). A *physiotherapist* supervised each participant.	
**Arcoverde et al.[104]**<br>**2013**<br>**2-arm RCT**	**Intensity:** 40%–60% of VO_2max<br>**Duration:** 16-week<br>**Training load:** 30-min, 2 times/week<br>**Intervention proposal:** The exercise group was composed by *10* patients who were submitted to a period of adaptation to the *treadmill* (BH fitness-Explorer Pro Action) for 4 weeks, with progressive intensity and duration, until reaching a percentage of 40% of VO_2max (initial speed 2km/h, initial duration 20 mins). During this period, the patients got *familiarized* with the RPE. The intervention was divided into 3 phases*: (1) Warm-up exercises* on the treadmill for 10 mins at an intensity of 40% of VO_2max; (*2) 20 mins at an intensity of 60% of VO_2max*; (*3) 5 mins of supervised stretching exercises* focused on the big muscles' groups. The patients were supervised by *physical therapist and physical educators* and monitored every 5 mins by frequency counters (*Polar1 display*) and *RPE*. Moreover some instructions, were used to them, such as to look in front, to hold on the treadmill, to give wide steps, do not talk, to keep adequate posture.	1. A beneficial effect on the global cognitive function<br>2. Significant improvements in:<br>a) Balance<br>b) Mobility
**Angiolillo et al.[105]**<br>**2023**<br>**2-arm RCT**	**Intensity:** Personal perception of maximum exertion<br>**Duration:** 24-week<br>**Training load:** 60-min, 2 times/week<br>**Intervention proposal:** Both control and exercise group (EG)s' patients underwent 2 hours a week of cognitive re-education based on formal *Reality Orientation Therapy* (ROT) with *physiotherapists* or *occupational therapists*; 8 hours a week of *cognitive re-education* based on informal ROT with *psychologists* and *socio-health professionals*; 4 hours a week of *music therapy* with *music therapists*; 2 hours a week of *motor, proprioceptive and postural rehabilitation* with physiotherapists. The *15* EG patients performed training sessions under the supervision of a *specialized trainer*, consisting of *10 mins of warm-up with stretching exercises, 40 mins of Nordic Walking (NW), and 10 mins of cool-down with stretching exercises*. NW training was not standardized in terms of intensity level but was carried out on the personal perception of maximum exertion.	Improved cognitive function (executive function, selective attention and processing speed, visual-spatial reasoning abilities, and verbal episodic memory)
**Enette et al.[106]**<br>**2020**<br>**3-arm RCT**	**Intensity:**<br>IG[1]: 70% of HRmax<br>IG[2]: 80% combined 60% of HRmax<br>**Duration:** 9-week<br>**Training load:** 30-min, 2 times/week<br>**Intervention proposal:** *Continuous aerobic training (CAT) and Interval aerobic training (IAT) consisted of a cycling workout on an upright electronically braked cycle ergometer* (Kettler GmbH & Co KG E5, Kleinblittersdorf, Germany). Each session was started with *a 2 mins warm-up and finalized with 1 min cool-down*. An *experienced and trained physiotherapist* supervised all the training sessions. HR was continuously monitored and automatically recorded (*Polar H10*, Kempele, Finland). *SBP and DBP* were also systematically measured (*Omron M3 V4*, Kyoto, Japan) before and after each session. In order	1. Compared to control, intervention groups were however effective to improve a) aerobic fitness parameters and b) functional capacities with globally no significant difference between them<br>2. Better QoL was also reported only in CAT group

	to determine the appropriate intensity of CAT and IAT for each participant, we applied exercises protocols using either a fixed percentage of HRmax or a fixed percentage of workload of MTP for participants with low fitness levels.	
**Phoemsapthawee et al.[107]<br>2016<br>2-arm RCT**	**Intensity:** 23% of VO_2 peak<br>**Duration:** 12-week<br>**Training load:** 30-min, 5 times/week<br>**Intervention proposal:** *A warm-up and a cool-down of each period were 5 mins of stretching exercise*. At the first week of the training, the speed of *swinging* was 20 times·min-1 and progressed to 25 times·min-1 during week 4 to week 5 and 30 times·min-1 beyond that. Exercise duration was 10 to 15 mins for the first 3 weeks and then, it was increased by 5 mins every week until the duration reached 30 mins. In starting position, each subject stood or sat on a stool. The feet were firmly placed on the ground at shoulder width apart. Subjects were asked to keep their trunk straight with their arms hung naturally. Then, both arms were swung forward about 30° with a smooth and even force and then backward to about 60°. During the Arm Swinging Exercise, the subjects were informed to maintain their control of breathing and to concentrate their minds on swinging their arm. Before and after the intervention, the *RPE* scale was asked.	Significant improvements in:<br>1. The cognitive performance<br>2. Aerobic capacity<br>3. Oxidative stress
**Eggermont et al.[108]<br>2010<br>2-arm RCT**	**Intensity:** N/A<br>**Duration:** 6-week<br>**Training load:** 30-min, 5 times/week<br>**Intervention proposal:** Intervention was applied *one-on-one* by *psychology students*. The participants *walked at a self-selected speed*. Short moments of rest were included, if necessary. Walks were primarily on the wards or in public places in the *nursing home*.	No positive effect
**Lowery et al.[109]<br>2013<br>2-arm RCT**	**Intensity:** 12–14/20 RPE<br>**Duration:** 12-week<br>**Training load:** 20–30-min, 5 times/week<br>**Intervention proposal:** Physical exercise was delivered as an *individually tailored regimen of walking* designed to become progressively more intensive and last between 20–30 mins. This was facilitated by *one registered exercise professional qualified in instructing physical activity and exercise* (National Vocational Qualification Level 3) and delivered to participants in the treatment arm of the trial in and around their own *home*. To help distinguish between a therapist effect operating through social contact and the effect of PA, the intervention was divided into 2 x 6-week epochs. The *exercise therapist* progressively withdrew support at the end of the first epoch, with the expectation that the *dyad* would perform the exercise regimen regularly and independently at least 5 times/week. The therapist trained participant dyads in its use and validated their *RPE* ratings.	No statistically significant
**Guzel et al.[110]<br>2024<br>3-arm RCT**	**Intensity:** N/A<br>**Duration:** 6-week<br>**Training load:** 40-min, 2 times/week<br>**Intervention proposal:** *Aerobic, balance, and combined exercise* programs were performed under the supervision of a *physiotherapist*. All exercise programs for 3 groups were performed in a bright, silent, and large room to prevent cognitive confusion and to keep attention.<br>*Aerobic Exercise*	1. Balance and combined exercise programs provided more beneficial effects on both a) cognitive and b) physical functions compare to aerobic exercise program

	The aerobic exercise program involved repetitive use of large muscle groups accompanied by deep breathing exercises. To fully differentiate these exercises from balance exercises, the entire aerobic exercise protocol was developed to be performed on a *chair*. An exercise session consisted of 3 parts and lasted 40 mins. First, *a 5-min warm-up* sequence with general active mobilization movements. Following the warm-up, a 30-min aerobic exercise sequence consisting *of simple, rhythmic, motor movements in a full range of motions using body weight and deep breathing*. Finally, *a 5-min cool-down* sequence with stretching movements accompanied by breathing.<br>*Balance Exercise*<br>The *Otago Exercise Program* is the basic concept of the balance exercise program. It includes 12 different balance movements, all of which are to be performed *standing position*. Participants were *allowed to sit and rest* between the movements. Each exercise session consisted of 3 parts and lasted 40 mins. First, a 5-min warm-up sequence with general active mobilization movements. Following the warm-up, a balance exercise sequence of 30 min, including *unipedal and bipedal stances, different walking patterns, and trunk rotations*. Finally, *a 5-min cool-down* sequence with stretches accompanied by breathing.<br>*Combined Exercise*<br>The combined exercise program included *both aerobic and balance exercises*. The combined exercise program included movements in both standing and sitting positions. Each exercise session consisted of 3 parts and lasted 40 mins. First, *a 5-min warm-up* sequence including general active mobilization movements. Following the warm-up, a *combined exercise sequence of 30 min*, including unipedal and bipedal stances, rhythmic large motor movements with deep breathing, different walking patterns, and trunk rotations. Finally, *a 5-min cool-down* sequence with<br>stretches accompanied by breathing.	2. Balance and combined exercise programs had similar effects both on a) cognitive and b) physical functions, except for visual memory
**Venturelli et al.[111]**<br>**2011**<br>**2-arm RCT**	**Intensity:** Moderate<br>**Duration:** 24-week<br>**Training load:** 30-min, 4 times/week<br>**Intervention proposal:** *Nursing home residents* and *caregivers* assigned to the *walking program* received detailed instructions from the research staff; exercise time and the walking modalities were demonstrated and tried before the intervention period in the presence of a *physiotherapist*. For the residents' safety, caregivers were instructed to maintain a constant walking speed and to avoid accelerating or stopping, but to try, if possible, to understand the *participant's own pace* each time. To do this, the walking session times, and number of laps were recorded in the *log* and checked before and after each visit. The program consisted of a simple aerobic walking activity. When the caregiver arrived at the Alzheimer's care unit, he or she met the resident in the adapted living area, usually between 3 and 5 p.m. After 1 or 2 mins of informal chatting, when the caregiver and the resident felt comfortable with each other, the caregiver asked the participant to accompany them for a walk. Both walked, arm in arm, for 30 mins along the hallway; the distance covered during the 30 mins was easily calculated because the length of the corridor was the same used for the 6-MWT (60 m). During the walking session, the caregiver was instructed to encourage the participant to maintain the ''fastest''	1. Increased the levels of physical activity<br>2. Improved:<br>a) Physical performance<br>b) ADLs<br>3. Intervention group maintained MMSE assessment values, while cognitive levels decreased in the control group

	walking speed possible. As positive psychological reinforcement for the exercise, at the end of the 30-min session, *cookies* were offered to the nursing home resident and caregiver.	
**Scherder et al.[112]**<br>**2005**<br>**3-arm RCT**	**Intensity:** N/A<br>**Duration:** 6-week<br>**Training load:** 30-min, 3 times/week<br>**Intervention proposal:** The '*walking*' treatment involved *self-paced* slow walking with an aid. With respect to the *hand/face exercises*: the hand exercises included, among other things, bending and stretching the fingers, and sliding a wooden club through the hand by moving the fingers. Facial activity consisted of producing seven different facial expressions, an exercise used with rehabilitation following paralysis of the facial nerve. All subjects of the 2 experimental groups received individual treatment for 30 mins a day (30 mins walking and 15 mins each for the hand/face exercises).	Improved excitative function (cognitive flexibility, self-shifting, planning, and purposive action (category Naming and Trail-making (A+B))
**Amjad et al.[113]**<br>**2018**<br>**2-arm RCT**	**Intensity:** 60%–80% of HRmax<br>**Duration:** 6-week<br>**Training load:** 60-min, 3 times/week<br>**Intervention proposal:** The patients were treated individually with exercise using *a treadmill and a stationary bicycle* in a *physical therapy department*. The intensity of patient exercise was also monitored and calculated from the HR by using *RPE*. Each exercise session consisted of *a 5–10 mins warm-up period at the beginning and a 5–10 mins cool-down period at the end*. During the entire exercise session the HR and oxygen saturation were monitored continuously by a *cardiac monitor*. Each exercise session was continued for a pre-determined time, or it was stopped early if the subject's heart Accepted Manuscript rate (calculated by Karvonen formula) became too high or they over-exerted themselves (assessed by Borg's scale). The *EEG* recordings were done before and after the first session of aerobic exercise to measure the short-term effects. The duration of each session of exercise was aimed to be *increased gradually* from 20 mins to 40 mins, from the first session to the last session; however, this would depend on the HR and exertion response of each individual patient as described above.	A improvement in cognition
**Abbas et al.[114]**<br>**2023**<br>**3-arm RCT**	**Intensity:**<br>IG[2]: 13–15 RM<br>**Duration:** 12-week<br>**Training load:** 50-min, 3 times/week<br>**Intervention proposal:** All sessions were implemented by *trained physical therapists*, and participants' health status was continuously monitored by *the medical system* of the older adult home with direct supervision by a *gerontologist*, part of the research team. Sessions were done *between 8:00 a.m. and 2:00 p.m. at least 1 hour after breakfast and before lunch meals.*<br>*The High-intensity Functional Exercises (HIFE) Training* Participants in both groups "Exercise (Ex)" and "Motorized cycle and Exercise (MoEx)" underwent the HIFE exercise program for 50 and 25 mins, respectively.<br>However, the "MoEx" group had additional training with *Motorized Cycle Ergometer* for 25 mins. This HIFE program as mentioned previously aimed to improve the strength of lower limbs and foster balance and mobility. The cycling was set at 60 cycles/minute. The device was calibrated by the supplier every 2 weeks to ensure stable functionality.	All trainings were effective in improving balance but not cognition

	For group "Ex," the session consisted of 50 mins training starting with *5 mins of warm-up* (walking on the spot, opposing arm-swings at the side of the body) followed by the exercise regimen that consisted of selected exercises performed in various weight-bearing and functional situations such as *standing from a chair, trunk exercises during standing, and walking*.<br>Training exercises were chosen to suit the *individual's capabilities* and were implemented individually to ensure proper execution in relation to the cognitive status and optimize safety. The *physical therapist* performed the exercises in front of the participant and asked her/him to mimic the movement done in order to overcome the cognitive status and facilitate movement execution. According to authors' recommendations, exercises were implemented 13 to 15 RM, and progression was done through increasing the difficulty of a specific exercise or shifting to a more challenging one. Exercise intensity was augmented by increasing step height, lowering the elevation of the chair, and adding a weighted belt (up to 12 kg). For postural stability purposes, progression was ensured by using an altered surface between parallel bars and through narrowing the base of support. Safety was assured using *worn belts with handles* throughout the session to prevent any fall.	
**Abd El-Kader and AI-Jiffri[115]**<br>**2016**<br>**2-arm RCT**	**Intensity:** 60%–70% of HRmax<br>**Duration:** 8-week<br>**Training load:** 25–45-min, 3 times/week<br>**Intervention proposal:** Participated in *treadmill aerobic exercise* (Enraf Nonium, Model display panel Standard, NR 1475.801, Holand). Training program *included 5 mins for warm-up in the form of range motion and stretching exercises, 10–30 mins of aerobic exercise training and 10 mins of cool-down* (on treadmill with low speed and without inclination). Participants with close supervision by *physical therapist*.	Improved:<br>1. QoL<br>2. Systemic inflammation<br>3. Psychological wellbeing
**Law et al.[116,117]**<br>**2021**<br>**4-arm RCT**	**Intensity:** 3–4/10 RPE<br>**Duration:** 8-week<br>**Training load:** 60-min, 1.5 times/week<br>**Intervention proposal:** The exercise group performed exercise sessions (4–6/group), facilitated by an *occupational therapist* and an assistant. All exercise sessions started with *a warm-up* (5–10 mins), followed by 30–40 mins *moderate intensity aerobic* exercise, including structured *whole-body movement exercise, bicycle and arm ergometry*, at 3–4/10 perceived exertion, ended with *a cool-down* (5–10 mins).	Exercise group showed higher performance in:<br>1. Functional status<br>2. Everyday problem-solving
**Cancela et al.[118]**<br>**2016**<br>**2-arm RCT**	**Intensity:** N/A<br>**Duration:** 60-week<br>**Training load:** 15-min, 7 times/week<br>**Intervention proposal:** The aerobic physical activity program consisted of *daily cycling* sessions. The participants attended the *gymnasium* daily and cycled continuously *alone, or in pairs*, in a *recumbent bicycle* geared to a very low resistance. They were instructed to pedal for a minimum of 15-min at a constant self-selected pace. A *physiotherapist* monitored each session registering the amount of time that each individual exercised each day as well as their adherence to the program. In order to adjust the on-going exercise program to each individual's capacity, their performance was assessed every 3 months.	Significant impacts on:<br>1. Improving cognitive functioning<br>2. Behavior<br>3. Functional mobility
**Yu et al.[119–121]**<br>**2021**	**Intensity:** 50%–75% of HRR, 9–15 RPE<br>**Duration:** 24-week	There were true inter-individual differences in:

**2-arm RCT**	**Training load:** 60-min, 3 times/week<br>**Intervention proposal:** The *cycling intervention* involved cycling on *recumbent stationary cycles* at moderate to vigorous intensity. The intensity and session durations were alternately increased by 5% of HRR (or 1 point on the RPE) or a 5-min duration increase over time. During a session, exercise intensity was monitored using the *Polar Wireless HR Monitor* (RS400; Polar, Lake Success, NY, USA), *RPE*, and "talk" test every 5 mins. BP was recorded every 10–15 mins, and overexertion signs and symptoms were continuously monitored. Participants left the site only after their *HR and BP* returned to pre-exercise levels. The investigators performed fidelity checks on randomly selected sessions.	1. Aerobic fitness<br>2. Cognitive responses
**Baker et al.[122,123]**<br>**2025**<br>**2-arm RCT**	**Intensity:** 70%–80% HRR<br>**Duration:** 8-week<br>**Training load:** 45-min, 4 times/week<br>**Intervention proposal:** A study-certified trainer provided *supervision* for the first eight sessions, and then for two of the four weekly sessions through month 12. In months 13–18, participants continued their assigned exercise programs, but without supervision. Participants were provided with a digital wrist-worn HR monitor with a chest strap for HR tracking while exercising. All participants were trained to use the RPE in the first 2 weeks of the program as an additional means of assessing exercise effort. The intervention aligned with standard *ACSM* recommendations and protocols tested in smaller-scale clinical trials. The prescription targeted aerobic exercise (includes warm-up and cool-down) and was introduced to participants by slowly increasing frequency and duration over the first 6 weeks to build self-efficacy and stamina, and to reduce risk of injury. Aerobic training targeted moderate-high cardiorespiratory intensity (70%–80% of HRR) and was completed on a treadmill (most common), elliptical trainer, or stationary cycle.	Cognition remained stable
**Huang et al.[124]**<br>**2025**<br>**3-arm RCT**	**Intensity:**<br>IG[1]: 60%–70% HR max, 12–14/20 RPE<br>IG[2]: 50%–70% 1RM, 5–6/10 RPE<br>**Duration:** 24-week<br>**Training load:** 30–50-min, 3–5 times/week<br>**Intervention proposal:** After baseline assessments, participants in the AE group or RE group receive: (1) *a handbook and a video* about AE or RE training, allowing them to review the training program details at home; (2) on-site guidance about how to exercise, how to assess the perceived exercise intensity using the corresponding scale and how to share electronic exercise records and complete exercise diaries; and (3) HR bracelets (Redmi band, Xiaomi Technology, MIUI Technology Park, Beijing, China), provided free of charge, to monitor intensity and share their exercise records with the researchers.<br>AE group was instructed to complete AE training by *walking or cycling* according to their preferences. Electronic exercise records derived from HR bracelets or self- reported RPE scores were submitted to researchers after each session of exercise. If the average HR was below 60%–70% HRmax or the RPE score was below 12 during exercise, timely feedback would be given to participants to recommend they elevate intensity by increasing the speed of walking or cycling. In the first 2 weeks, participants started AE training with light intensity (40%–50% HRmax), 20 min per session, 3–5 days/week and gradually increased to moderate	1. AE had a positive effect on global cognition<br>2. RE improved executive function

	intensity (60%–70% HRmax), 30–50 min per session, 3–5 days/week. From week 3 onwards, participants would be encouraged to maintain the target exercise dose.<br>RE group performed resistance training using elastic bands (Thera- band, The Hygenic, Akron, Ohio, USA) on two non- consecutive days per week. Each session consisted of a 5 min warm-up; approximately 50 min of elastic band training consisting of six exercises targeting key muscle groups: <u>*triceps, biceps, quadriceps, leg muscles, pectoralis muscles and back muscles*</u>; and a 5 min cool- down exercise. Each exercise initially consisted of three sets of 12–15 repetitions at an intensity of 30%–50% of one repetition maximum (1RM) and gradually progressed to three sets of 10–12 repetitions at an intensity of 50%–70% 1RM. The OMNI-Resistance Exercise Scale was used to evaluate the perceived exertion with a target score of 5–6. Exercise intensity was adjusted by changing the tension of elastic bands. The initial tension of the elastic band was chosen based on participants' muscle strength and their perceived intensity at baseline. If a participant completed RE training with the OMNI- Resistance Exercise Scale score of less than 5, the elastic band would be replaced with one of higher tension. In the first 2 weeks, participants started RE training with three sets of 12–15 repetitions at 30%–50% 1RM and gradually achieved the target intensity (three sets of 10–12 repetitions at 50%–70% 1RM). From week 3 onwards, participants were asked to complete RE training at the target RE dose by adjusting the tension of the elastic bands.	
**Resistance Exercise (RE)**		
**Fernandez-Gonzalo et al.[125] 2016**<br>**2-arm RCT**	**Intensity:** N/A<br>**Duration:** 12-week<br>**Training load:** N/A-min, 2 times/week<br>**Intervention proposal:** Participants from training group performed <u>*unilateral resistance exercise*</u> training using the more-affected leg on a <u>*flywheel leg press*</u> (YoYo™ Technology AB, Stockholm, Sweden) with 0.036 kg*m^2 moment inertia, with >48 h of rest between sessions. After <u>*a brief standardized warm-up*</u>, 4 sets of 7 RM were performed. Briefly, following an initial submaximal repetition to initiate <u>*flywheel momentum, 7 consecutive maximal repetitions were performed*</u>, accelerating rotation of the wheel during concentric, and decelerating in the subsequent eccentric action. The trainees were requested to push with maximal effort during the entire range of motion in the concentric action (from ~70° to almost full extension), where the strap about the flywheel shaft was rolled out. Then, as the strap rewound, they aimed at resisting the inertial force gently during the first third of the eccentric action, and then by applying maximal effort to stop the movement at about 70° knee flexion. This strategy allowed for an eccentric-overload in force/power values during the last two-thirds of the eccentric cycle. Once the flywheel stopped, a subsequent concentric action was instantly initiated. <u>*A 3-min recovery period*</u> was allowed between sets. Peak concentric and eccentric power was measured in all repetitions (SmartCoach™, Stockholm, Sweden). Real-time performance feedback on peak power was offered to the patients at all times.	1. The intervention offered the powerful aids to:<br>a) Regain muscle mass and function<br>b) Functional performance<br>c) Balance<br>d) Gait<br>2. Improved:<br>a) Cognitive functions<br>b) Cause-effect relationship
**Singh et al.[126] 2014**<br>**4-arm RCT**	**Intensity:** N/A<br>**Duration:** 24-week<br>**Training load:** 75-min, 2–3 times/week	Significant improved:<br>1. Global<br>2. Executive functions

	**Intervention proposal:** *Progress resistance training* was supervised by *experienced research assistants* (*exercise physiologists and physiotherapists*) in a *physician-supervised clinic* at the University of Sydney Exercise campus at a ratio of 1 trainer: *4–5* participants. *Pneumatic resistance machines* (Keiser Sports Health Equipment, Ltd) were used for training at high intensity, 3 sets of 8 repetitions of each of 5–6 exercises/session for most major muscle groups (*chest press, leg press, seated row, standing hip abduction, knee extension*).	
**Lu et al.[127]**<br>**2016**<br>**2-arm RCT**	**Intensity:** N/A<br>**Duration:** 12-week<br>**Training load:** 60-min, 3 times/week<br>**Intervention proposal:** Participants in the training group participated *momentum-based dumbbell-training* class. All classes were conducted at *local senior centers*. The momentum-based dumbbell is a handheld device with 2 built-in eccentric pendulums (~9 cm in length) at each end. Each dumbbell is 32 cm in length, 22 cm in diameter, and weighs 1.92 kg. Both the size and weight of the dumbbell were fixed for all participants. The dumbbell training involved instructor-led handheld dumbbell-spinning exercises performed on the front part or lateral side of the body. Specifically, the spinning motion was done in different directions (e.g., clockwise and counterclockwise, forward and backward) and body sides (front; left/right). Each individual spinning exercise lasted 1–2 mins with repetitions set at 4–5 mins. The initial exercise sessions focused on acclimatizing participants to the movement forms and followed an easy-difficult progression of short duration (1 min) with multiple rest breaks. Exercises progressed to longer intervals (1.5–2 mins), with a 1-min *break* between each exercise set. The level of exercise difficulty was tailored to the capabilities of participants.	1. Significantly improved cognition<br>2. A concomitant improvement in the mobility measure
**Wang et al.[128]**<br>**2020**<br>**2-arm RCT**	**Intensity:** 60%–80% of HRmax<br>**Duration:** 24-week<br>**Training load:** 60-min, 3 times/week<br>**Intervention proposal:** *Structured limbs-exercise* program was designed by a team comprising a *physiotherapist*, an exercise physiologist and a nurse, in accordance with the recommendations of *ACSM*. It was characterized as a structured exercise, which *including a 10-min limbering-up exercise, 40-min of upper and lower limbs exercise, followed by a 10-min relaxation exercise*. In addition to the same health promotion classes given to the wait list control group, the participants in the intervention group attended supervised intervention with a maximum number of *10* people per group. During each exercise session, HR was monitored continuously using a *HR monitor* (*Xiaomi Mi Band*, Beijing Xiaomi Technology Co., Ltd,) on participant's left wrist. Participants practiced this program at *community healthcare center*. During the first week, the researcher taught this program to the participants. Beginning in the second week, subgroups of participants were asked to practice this program, and 2 trained physiotherapists with 5-year geriatric rehabilitation experience, who attended a 3-day training in advance and exhibited good protocol compliance, supervised and conducted all sessions in each center. Physiotherapist in each group regularly inquired participants about their perceived fatigue levels and implemented risk management for accidents and other adverse events during the training. Two study coordinators *phoned* all the participants to remind participants of this program before each session. If one of the participants missed a session, an extra one was offered to him or her as soon as possible. After completion of the 12-week training sessions, participants then practiced 3 unsupervised limbs-exercise sessions /week, 60 mins /session for the following 12 weeks at *home*. A 20 mins *video* was provided to assist participants to conduct this program in home. The participants were also provided with an exercise *logbook* and were required to complete daily logs tracking exercise duration and HR monitor measurements, and	1. A significant and sustainable benefits for maintaining general cognitive function<br>2. Significant impacts on:<br>a) Depressive symptoms<br>b) Sleep quality<br>c) Processing speed

	they received a phone call from the study coordinators once a week to monitor health and the implementation of this program.	
**Vints et al.**[129]<br>**2024**<br>**2-arm RCT**	**Intensity:** 70%–85% of 1RM<br>**Duration:** 12-week<br>**Training load:** N/A -min, 2 times/week<br>**Intervention proposal:** *Resistance training* (RT) intervention was conducted in the Lithuanian Sport University *gym* in accordance with the National Strength and Conditioning Association (USA) position statement on resistance training for older adults. Two to 10 days prior to the exercise intervention, participants were familiarized with the RT procedure and underwent 1RM testing. Two resistance training sessions were with a minimum of 2 days apart. Warm-up consisted *of 5 mins cycling on a cycle ergometer at an intensity (in Watts) approximately equal to the participant's body weight in kilograms, followed by a few dynamic stretching and activation exercises including lunges, butt kicks, sidestep lunges, half-squats, and front and side cross swings. The training program comprised 4 exercises, namely knee extension (1), incline leg press (2), hamstring curls (3), and calf raise (4), using resistance training equipment from Technogym* (Italy). Each exercise was performed for 2–3 sets of 6–10 repetitions, at 70–85% of the baseline 1RM, with a 2-min rest between sets and a 3–min rest between exercises. From week 1 to 3, participants did 8–10 repetitions, starting at 70–75% 1-RM; from week 4 to 9, they worked 6-8 repetitions, starting at 75–80% 1-RM; and from week 10 to 12, they did six repetitions starting at 80–85% 1-RM. After the first session and during each of the 3 training blocks the weight was adjusted according to the participants' rate of *RPE* on a 10-point Borg scale. The weight was increased when the older adult indicated a score below 7 on 10. The exercise sequence was *periodically randomized*. *Qualified trainers* supervised all training sessions.	1. The intervention positively affected inhibitory control<br>2. Induced increases in the neurotrophic Insulin-like Growth Factor-1 (IGF-1) may play a role in mathematical processing
**Yoon et al.**[130]<br>**2018**<br>**2-arm RCT**	**Intensity:** 12–13/20 RPE<br>**Duration:** 16-week<br>**Training load:** 60-min, 3 times/week<br>**Intervention proposal:** A *high-speed resistance training* program is defined as a contraction phase expected to be accomplished as quickly as possible, a 1-seocnd pause, and an eccentric contraction exceeding 2 seconds. High-speed resistance exercise regimens were based on the use of *elastic exercise bands*. Each session included *a 10-min warm-up, 40-min high-speed resistance training (seated row, one leg press, applied pec deck flus, seated leg raise, lateral raise, semi squats, wide squats, bridging), and 10 min of cool-down*. The sessions were separated by a minimum of 48 hours and were performed under the direct *supervision of an exercise instructor* to ensure safety and adherence with the exercise protocol. Exercise intensities were set by the color of the elastic exercise band. In the high-speed resistance training group, blue elastic bands (tension: low, 20 Nm) were used and the participants were instructed to perform exercise training. The high-speed resistance exercise consisted of 2–3 sets of 12–15 repetitions.	Significantly improved:<br>1. Processing speed<br>2. Executive function
**Yoon et al.**[131]<br>**2017**<br>**3-arm RCT**	**Intensity:**<br>High-speed power training (HSPT): 12–13/20 RPE<br>Low-speed power training (LSST): 15–16/20 RPE<br>**Duration:** 12-week<br>**Training load:** 60-min, 2 times/week<br>**Intervention proposal:** Both exercise groups were based on the use of *elastic exercise bands*. Each session included *a 10-min warm-up, 40 mins of elastic band training and10 mins of cool-down exercises*. Resting periods of 1 min were allowed between sets, and 2 mins between	1. Two interventions produced significant and small-to-large improvements in:<br>a) Bodyweight<br>b) BMI<br>c) Skeletal muscle mass

	exercises. Participants were supervised by a *qualified instructor* who was also *certified in cardiopulmonary resuscitation and the use of an automated external defibrillator*. Exercise intensities were set by the color of the elastic band. For the HSPT, green elastic bands (tension: very low) were used, and the participants were instructed to carry out exercise training at a RPE of 12–13 ("*somewhat hard*"). Each HSPT exercise consisted of 2 to 3 sets of 12–15 repetitions. For the LSST, blue color elastic bands (*tension: high*) were used. For this group, the participants were instructed to carry out exercise training at a *RPE* of 15–16 ("hard"). Each LSST exercise consisted of 2 to 3 sets with 8–10 repetitions. The intensity and difficulty level of the exercises (i.e. number of sets and number of repetitions) progressively increased with time. Exercises in both groups were supervised for proper velocity and technique. HSPT included a contraction phase instructed to be carried out as quickly as possible, a 1-second pause and an eccentric contraction exceeding 2 seconds. LSST exercise movements consisted of a contraction phase longer than 2 seconds, a 1-second pause and an eccentric contraction exceeding 2 seconds.	d) % body fat<br>e) Waist-to-hip ratio<br>f) Systolic BP<br>g) Functional abilities<br>h) Grip strength<br>i) Isokinetic knee extension and flexion at 60°/s and 180°/s<br>2. Clinical improvements in cognitive functions
**Lee et al.[132]**<br>**2020**<br>**2-arm RCT**	**Intensity:** 12–13/20 RPE<br>**Duration:** 8-week<br>**Training load:** 50-min, 3 times/week<br>**Intervention proposal:** *High-speed power training* was conducted based on frequency, intensity, type, and time. The frequency of this intervention involved 10–12 repetitions of 2 to 3 sets. The training intervention was including a 10-min warm-up involving stretching, while the main exercise lasted for 30 mins, followed by a 10-min cool-down. The *resting period* between the different stretches was 30 seconds, and 1 min between sets. The intervention group was subjected to 1-week *body-weight training* in the early phase of intervention for *familiarization* of the main training in this study. The 1-week body-weight training program was composed of 8 movements such as *seated row exercises, one-leg press, applied pec dec flys or lateral raises, seated leg raises, normal squats, full squats, wide squats, and bridging exercises*. The subjects in the intervention group were trained in 8 movements with an *elastic band* from 2 to 4 weeks (*seated row, one-leg press, applied pec deck flys, seated leg raise, lateral raise, semi-squats, normal squats, and bridging*) and the movements were slightly altered from 5 to 8 weeks in order to maintain "somewhat" training intensity (*seated row, one-leg press, applied pec deck flys, seated leg raise with 5-s hold-up, lateral raise, normal squats, wide squats, and bridging*). The intensity of the exercise training was progressively increased with the number of repetitions and sets. The intervention group was instructed to perform concentric contractions for 1 second and eccentric contractions for 3 seconds by an exercise expert during the training. The *exercise expert* provided the technique and velocity of the training for the intervention group by using verbal counting signals.	The favorable effects on:<br>1. Neuromuscular function<br>2. Gait performance
**Hong et al.[133]**<br>**2018**<br>**2-arm RCT**	**Intensity:** 15RM<br>**Duration:** 12-week<br>**Training load:** 60-min, 2 times/week<br>**Intervention proposal:** The exercise groups performed *resistance exercises* for 1 hour/session with an *elastic band* (TheraBand; Hygenic Corporation, Akron, Ohio). The exercise program consisted *of a 10-min warm-up, a 40-min main exercise period with an elastic band at 15RM, about 65% of maximum), and a 10-min cool-down period with stretching*. Subjects were given a band selected on the basis of a strength test done at baseline with elastic bands of different elasticities, and the band was replaced with a higher-intensity band when subjects adapted to current elasticity.	1. Significantly enhanced physical fitness levels<br>2. Only showed the slight change in cognitive function

**Venturelli et al.[134] 2010 2-arm RCT**	**Intensity:** 53%–62% of HRmax<br>**Duration:** 12-week<br>**Training load:** 45-min, 3 times/week<br>**Intervention proposal:** Each exercise session consisted of *10 mins for warm-up, 25 mins for resistant circuit training, and 10 mins for cool-down*. Exercising tools used in the study were *resistance elastic bands* (yellow and red Therabands), *dumbbells* from 0.5 to 3 kg, *sticks, and sponge balls*. The *warm-up* program included active arm and shoulder mobilization by sponge balls and sticks. After the warm-up, *resistant circuit-training exercises* for the upper part of the body were performed at a moderate intensity: the initial workload with barbell was set at 50% of 1RM of the arm curl test and was subsequently enhanced by increasing the number of repetitions and/or the load, provided that medical conditions were stable, and exercises were executed without dyspnea. The initial workload for resistant training by the elastic band was set according to the 1RM arm curl test: subjects with low maximal strength (1st and 2nd quartiles of 1RM) used yellow Theraband, patients with high maximal strength (3rd and 4th quartiles of 1RM) used red Theraband. The elastic band workload was increased during the training period only by increasing repetitions. During the *cool-down* phase, subjects performed static stretching exercises for arms, shoulders, and neck, keeping the maximal muscle elongation for 30 seconds, and increasing the range of motion. In order to obtain a global evaluation of individual cardiocirculatory involvement during the exercise session, participants not assuming drugs interfering with the HR were monitored by a *HR monitor*. The exercises were performed under the supervision of a *kinesiologist* by small groups of *3–6* participants in sitting positions (on the wheelchair), exercise training was carried out in the long-term ward; no specific area was created, but a large room was adopted as gym during the experimental training period.	Showed improvements in:<br>1. Arm strength<br>2. Shoulder flexibility<br>3. ADLs<br>4. Maintained the MMSE scores
**Holthoff et al.[135] 2015 2-arm RCT**	**Intensity:** N/A<br>**Duration:** 12-week<br>**Training load:** 30-min, 3 times/week<br>**Intervention proposal:** Patients in the intervention group trained their *lower body* on a *movement trainer* (ReckMOTOmed) with a computer controlled and individually preassigned training flow. *Caregivers* were asked to choose a familiar chair prior to study begin. The movement trainer was positioned in front of the patient and PA training was performed from the comfort of that chair. We anticipated that using the familiar chair would encourage the patient's participation and reassure the caregiver that PA would not cause adverse reactions, e.g. falls. Participants were required to train at an individually chosen time with at least one day without training in between 2 training days. Times and dates of PA training were documented by *the movement trainer computer system*. The program changed between passive, motor-assisted or active resistive training of the legs as well as changes in direction (forward, reverse) every 5 mins. During a *familiarization* session prior to the study patients were asked to use the movement trainer and to respond to whether they felt that the training resistance level was appropriate (too easy, too high, just right). They started with level 1 and the level was increased as they made their choice. The movement trainer has 20 levels of motor resistance and the patients in the intervention group chose an activity level between 2 and 4. The level did not change over time. *Caregivers* were asked to act encouraging but to leave the room as soon as the patient started training. This design was chosen to control for social contact times between both groups.	The results demonstrated transfer benefits to:<br>1. ADL<br>2. Cognitive<br>3. Physical skill

**Baek et al.[136]**<br>**2024**<br>**2-arm RCT**	**Intensity:** 12RM<br>**Duration:** 6-week<br>**Training load:** 40-min, 3 times/week<br>**Intervention proposal:** Each group performed an exercise using a <u>*body spider*</u> (KOOPERA, Germany). A body spider is an exercise device that uses the <u>*elasticity of a rubber rope*</u>, and both concentric and eccentric contractions are possible. It comprises a 3-stage frame of the upper, middle, and lower parts; therefore, it is capable of whole-body exercise in various directions. Body spider was also used to confirm the effects of exercise in older adults. In both groups, <u>*warm-up exercise was performed for 10 mins before main exercise and cool-down exercise was performed for 10 mins after main exercise*</u>. The dual tasks group additional performed such as writing names, drawing pictures, and subtracting numbers while performing the same resistance exercise applied to the control group. The strength of the resistance exercise was measured at 1RM, and 3 sets were performed with approximately 10 repetitions per set, with a maximum of 12RM.	Significantly improved:<br>1. Mood<br>2. Depression<br>3. Functional fitness<br>4. ADL
**Kusleikiene et al.[137]**<br>**2025**<br>**2-arm RCT**	**Intensity:** 70%–85% 1RM, 7/10 RPE<br>**Duration:** 12-week<br>**Training load:** N/A, 2 times/week<br>**Dance proposal:** A progressive, lower-limb-focused resistance training program that was adjusted based on individual physical abilities was be implemented. Participants were trained with a maximum of two participants per fitness instructor simultaneously. The training sessions using resistance training equipment from Technogym (Italy). <u>*Warm-up*</u> consisted of 5-min cycling on a cycle ergometer, at an intensity (in Watts) approximately equal to the participants' body weight in kilograms, followed by a few dynamic stretching and activation exercises, including lunges, butt kicks, sidestep lunges, half squats, and front and side cross swings. The main resistance training program consisted of <u>*three sets of four lower limb exercises*</u>: (1) leg press, (2) leg curl, (3) leg extension, and (4) calf raises. The order of these exercises was not controlled, but in general, the participants were instructed to start with the leg press, as this is a multi-joint exercise, and end with a single-joint exercise such as the calf raises. The weight was adjusted during the three training blocks according to the participants' RPE on a 10-point Borg scale. The weight was increased when the older adult indicated a score below 7 on a scale of 1–10. The RPE was logged in a notebook by the fitness instructors, along with the number of repetitions and the weight lifted. The resistance training protocol was <u>*supervised by qualified fitness coaches*</u>.	Improved cognitive performance and slowed neuronal loss in the hippocampal complex
**Multicomponent Exercise (ME)**		
**Yang et al.[138]**<br>**2022**<br>**3-arm RCT**	**Intensity:** Moderate<br>**Duration:** 12-week<br>**Training load:** 30-min, 2 times/week<br>**Intervention proposal:** The exercise intervention included a warm-up, an <u>*aerobic and resistance training*</u> period, and a cool-down. Aerobic exercise intensity was monitored at a moderate intensity using <u>*RPE*</u> and a target <u>*HR*</u>. Participants <u>*performed indoor and outdoor walking, and jumping jack, skip jump step box walking*</u>. The protocol for resistance training programs was progressive in loading (2–3 sets and 65% of 1RM). Free weight (weight-bearing) and/or Thera-band were used to provide the training stimulus. The muscular endurance training stimulus was subsequently increased using the 10–15RM method: 2 sets of	1. Improved global cognitive function and<br>2. Showed positive change in different brain regions<br>3. Exercise program showed a greater improvement in physical function than other groups

	from 10 to 15 repetitions were completed with proper form and without discomfort. Resistance training was targeted the 6 large muscle groups: *hamstrings, quadriceps, gastrocnemius, biceps, triceps, erector spinae*. All the exercises were performed with the instructions and under the supervision of a *fitness instructor*.	
**Doi et al.**[139] **2013** **2-arm RCT**	**Intensity:** 60% of HRmax<br>**Duration:** 24-week<br>**Training load:** 90-min, 2 times/week<br>**Intervention proposal:** The multicomponent exercise program included combinations of *aerobic exercise, endurance walking, muscle strength training, postural balance retraining, and gait training*. HR was monitored during the aerobic sessions using *portable HR monitors* (*Pulse Plus*, TRYTECH Co. Ltd., Tokyo, Japan). 11 of the 40 classes during the 6-month intervention period included 20–30 mins of consecutive *outdoor walking*. Postural balance exercises, such *as tandem walking and side walking on balance boards*, were also included in the sessions. Gait training was conducted at the same intensity as the aerobic exercise and included *dual-task walking*, e.g., walking whilst conducting a conversation or creating a *poem*. For the ladder training exercise, subjects learned to step into consecutive square segments and were instructed to step as quickly and accurately as possible. In addition, participants performed combined exercises, e.g., *circuit training including stair stepping, ladder training and endurance walking*. The exercise program also included a focus on *promoting exercise and behavior change*. Two *trained physiotherapists*, with geriatric rehabilitation experience, supervised and conducted the sessions. Each session began with a 10-min warm-up period that included dynamic and static stretching, followed by 20 mins of strength training and then combinations of *aerobic exercise, gait training, postural balance training* for an additional 60 mins. The participants were provided with an exercise and physical activity *logbook* and were required to perform and record daily home-based exercises and walking. To promote exercise and behavior change, the subjects were required to self-monitor their own PA levels using a *pedometer*.	Improved gait abilities (gait speed, stride length, and trunk smoothness during gait)
**Uemura et al.**[140,141] **2012** **2-arm RCT**	**Intensity:** 60% of HRmax<br>**Duration:** 48-week<br>**Training load:** 90-min, 0.5 times/week<br>**Intervention proposal:** The exercise program involved *aerobic exercise, muscle strength training, postural balance retraining, and combined training*. In addition, the exercise program included a focus on *promoting exercise and behavior change*. Two trained physiotherapists involved in geriatric rehabilitation conducted each intervention. Each supervised session began with *a 10-min warm-up period and stretching exercise, followed by 20 mins of muscle strength exercise*. Then, the participants practiced *aerobic exercise, postural balance retraining, and combined training for 60 mins*. For the aerobic exercise, participants underwent stair stepping and endurance walking. Before and after each session of the program, the *physiotherapists* conducted a physical check of each participant. The participants were required to carry out *daily home-based* muscle strength exercises and walking, which were self-monitored using a *booklet and pedometer* based on the concept of promoting exercise and behavior change.	Positive changes in important vascular risk factors related to cognitive decline and vascular disease
**Li et al.**[142] **2021** **2-arm RCT**	**Intensity:** 4–5/10 RPE<br>**Duration:** 24-week<br>**Training load:** 30-min, 5 times/week<br>**Intervention proposal:** Participants in the experimental group received *multicomponent exercise training*. Multicomponent exercise training involved comprehensive exercise including *aerobic exercise, strength training, balance training, coordination training, and*	Improved:<br>1. Physical function<br>2. Improved cognitive function

	sensitivity training. *Warm-up*: the participant was guided to march on the spot, static stretches on the floor, lasting for 5 mins, 3–4/10 *RPE*. *Aerobic exercise training*: shoulder range of motion (shoulders rotating clockwise and counterclockwise), clapping hands, toe side pointing and knee lifting motion, lasting a total of 5 mins, 4–5/10 *RPE*. *Strength training*: half squats, bend standing, elbow and knee bend motion lasting for 5 mins, 4–5/10 *RPE*. *Balance training*: supported standing on one leg, kicking motion (leg and right kicking forward and backward with trunk straightened), lasting for 5 mins, 4–5/10 *RPE*. *Coordination training*: 3 steps forward and back, zig zag movements, weaving in and out of chairs, circuit, lasting for 5 mins, 4–5/10 *RPE*. *Sensitivity training*: fast, fixed-pattern foot tapping, walking ball bounces, lasting for 5 mins, 4–5/10 *RPE*. The multicomponent exercise group were *gathered* together to receive the intervention. Researchers explained and demonstrated exercise techniques to the participants, ensuring that they mastered the correct exercise methods. After the intensive training session, the exercise training group was subdivided into 5 groups, and participants who exhibited skillful use of the techniques and organizational ability were selected to lead the team. All of the exercise trainings were performed under the same 2 instructors to maximize the intervention compliance. A *pamphlet and videotape* which explained the mechanisms and benefits of the multi-component exercise training were given to the participants to guide their training at *home*.	
**Suzuki et al.[143]<br>2012<br>2-arm RCT**	**Intensity:** 60% of HRmax<br>**Duration:** 48-week<br>**Training load:** 90-min, 2 times/week<br>**Intervention proposal:** Subjects in the multicomponent exercise group exercised under the supervision of *physiotherapists*. Two physiotherapists involved in geriatric rehabilitation and 3 *well-trained instructors* conducted each intervention. The exercise class consisted of *16–17* participants, and each supervised session began with *a 10-min warm-up period, followed by 20 mins of muscle strength exercise*. The subjects then practiced *aerobic exercise, postural balance retraining, and dual-task training for 60 mins*. In the aerobic exercises and postural balance retraining, subjects completed circuit training including stair stepping, endurance walking, and walking on balance boards. *HR was monitored* after aerobic exercise each session to take their pulse. One out of every 4 classes during the intervention period included *outdoor walking* during approximately 20–30 mins. These exercises or training were also conducted under *multitask conditions*. For example, the subjects in the exercise group were asked to invent their own *poem* while walking. In the ladder training, subjects memorized a step pattern in consecutive square segments and were instructed to step as quickly and accurately as possible. Before and after each session of the program, physiotherapists conducted a health check of each subject. The physiotherapists and well-trained instructors provided ongoing *safety monitoring* to prevent adverse accidents such as falling during the program. *Daily home-based exercise* in addition to structured program and outdoor walking was recommended to the exercise group. The subjects allocated the exercise group were asked to be recording the amount of time spent on daily home-based exercise and the daily total steps for pedometer in a *notebook*.	Improved cognitive performance (immediate memory and language ability)
**Jurakic et al.[144]<br>2017<br>2-arm RCT**	**Intensity:** N/A<br>**Duration:** 8-week<br>**Training load:** Feedback-based balance and core resistance training: 30-min; Pilates training: 60-min, 3 times/week<br>**Intervention proposal:** *Feedback-based balance and core resistance training* which included *push and pull exercises on the handles in different postures, hand positions, and directions*. The force level ranged from 50% of MVC during the first 2 weeks, over 65% of MVC during	1. Both interventions:<br>a) Enhanced global cognitive functioning<br>b) Improved some of the specific cognitive domains

	the next 3 weeks, to 75% of MVC during the last 3 weeks. The duration of isometric actions ranged from 5 to 7 seconds, and participants performed between 30 and 60 contractions/session. In *Pilates training*, core stability was addressed by the use of abdominal *bracing and pelvic tilt*. A typical session included *supine, side-lying, sitting, and quadruped exercises*. The difficulty of these exercises was gradually increased, and the focus was maintained on keeping a neutral posture and stable core in different gravity orientations. Every session ended with lower- and upper-limb exercises using elastic bands. Each exercise was performed for 2–4 sets with 15–20 seconds contraction time (isometric exercise) or 15–20 repetitions (dynamic exercise).	2. Significant improvements only in Feedback-based balance and core resistance training group:<br>a) Visuospatial/executive functions<br>b) Orientation<br>3. Significant improvement in short-term memory recall task was obtained only in the Pilates group
**Kim and Yim[145]**<br>**2017**<br>**2-arm RCT**	**Intensity:** N/A<br>**Duration:** 6-week<br>**Training load:** 60-min, 3 times/week<br>**Intervention proposal:** The exercise protocol for *handgrip strength* improvement consisted of grasping training with *a hand exerciser*, grasping training with a *power web exerciser*, and the *Digi-Flex repetition training*. The grasping training was implemented by reorganizing the combined wrist and hand exercise method with 2-second holding and 1-second release for each time. The training was applied to the unaffected side first, and then to the affected side, 15 times in 3 sets, for 15 mins. This was performed for 3 weeks; to improve the exercise effect, the program frequency and intensity increased according to the participant's level. The hand exerciser, power web exerciser, and Digi-Flex have predefined levels for resistance. According to the participant's condition and handgrip strength, the levels were adjusted. Additionally, to help the participants better understand the task, a demonstration was provided along with feedback while the participants were following the movements for practice. To minimize any compensatory movements, the participants were not allowed to lean back in the chair and were asked to position their legs evenly with both feet on the ground. The exercise protocol for increasing walking speed in this study involved the use of a *motor-powered treadmill*. During *the treadmill walking training*, the *therapist* stayed close to ensure participant safety. The treadmill speed was adjusted by the participants according to their *comfort level*, and the speed was recorded. In each round of training, the speed was increased by 0.1 km/hour. Concerning weight loading, 5% of body weight was applied to the unaffected side to increase the walking speed. The total training time was 20 mins, which included *15 mins of actual training and preintervention/postintervention warm-up and cool-down movements* to ensure efficient training. The participants were allowed to take a break as needed. The training involved the application of central nervous system developmental therapy in patients with stroke. Specifically, it included *joint movements, mat exercises, and walking exercise* for 10 mins per round. The participants underwent physical therapy for 60 mins/session, 2 sessions/day, 5 times/week, for 6 weeks in line with the hospital prescription and schedule. The main purpose was functional recovery by facilitating proprioception in supine, sitting, and standing positions according to the participants' functional levels. The program also included walking training in the *therapy room*.	Positive effects on cognitive function
**Li et al.[146]**<br>**2022**<br>**2-arm RCT**	**Intensity:** N/A<br>**Duration:** 8-week<br>**Training load:** 60-min, 3 times/week	Improved:<br>1. Working memory

	**Intervention proposal:** In brief, the program comprised 3 components: a *mobile application* (*the BRAVE app*), *peer volunteer training*, and an *exercise program*. The BRAVE app provided updated information on brain health, benefits of exercise and an exhaustive list of exercise videos and served as an activity scheduler, tracker and social network for the participants, volunteers and the coach. Peer volunteers were selected based on having volunteering experience, cognitively intact and physically active (defined by the International Physical Activity Questionnaire as moderately active with 3 to 6 Metabolic Equivalent Tasks).<br>The exercise program included *supervised exercise sessions and community-based self-practice*. The supervised sessions were implemented in a small group format (*8–10* participants per group). The training was delivered by a *coach* with the support of 2 to 3 *peer volunteers* per group who lived in the same residential area as the MCI persons. We designed a multicomponent exercise protocol that was practice able to perform in a community setting using easily accessible exercise equipment and simulated daily functional tasks. The volunteers acted as role models to demonstrate exercises and to create a pleasant atmosphere during the sessions. During the last supervised session, each volunteer was paired up with *3 to 5* persons with MCI, and the coach assisted the volunteer-MCI groups to set exercise goals and creating an action plan for group exercise. To enhance cohesiveness within the group, volunteers and participants could communicate through the BRAVE app and access various exercise videos. After completion of all the supervised exercise sessions, the coach also provided continuous support over a 4-week period. Through the online platform, *video* updates and onsite visits to encourage community-based self-practice.	2. Processing speed<br>3. Executive function
**Avenali et al.[147]**<br>**2021**<br>**2-arm RCT**	**Intensity:** N/A<br>**Duration:** 4-week<br>**Training load:** 60-min, 6 times/week<br>**Intervention proposal:** The physical therapy program was carried out during the *hospitalization* in the *Neurorehabilitation Unit* of IRCCS Mondino Foundation in addition to their usual pharmacological therapy. The program included a variety of *different exercise modalities* (*aerobic exercises, coordination exercises, treadmill training and exercise intervention program*) performed under the supervision of a *physiotherapist*, to facilitate goal-directed learning through cognitive engagement (learning through verbal feedback, cues, maintaining motivation and attention, improving awareness).	A positive effect on cognitive functions
**Mak et al.[148]**<br>**2022**<br>**2-arm RCT**	**Intensity:** 12–14/20 RPE<br>First stage:<br>**Duration:** 25-week<br>**Training load:** 60-min, 2 times/week<br>Second stage:<br>**Duration:** 24-week<br>**Training load:** 30-min, 2 times/week<br>**Intervention proposal:** The intervention involved 2 stages. The first stage involved an individually prescribed, supervised, and *progressive resistance and balance training program*. Each participant completed exercises in a circuit that included *pneumatic resistance training* (*using HUR Health and Fitness Equipment*) and balance exercise stations. Exercise dose was prescribed by a *physiotherapist* and was tailored to the individual, accommodating any comorbidities to minimize the risk of harm. Initial exercise dose was set at a self-reported “moderate” level, with participants achieving 2 to 3 sets of 10 to 15 repetitions per exercise. Dosages were reviewed fortnightly by a physiotherapist, adjusting to each participant’s changing ability over the course of stage 1. Resistance was increased if the participant reported a less than “moderate” intensity on the *Borg Scale*. Participants exercised in groups of up to *10*	Significantly reduced:<br>1. The rate of falls (by 50%)<br>2. Risk of falls<br>3. Injurious falls

	people. Stretching was performed as a cool-down at the completion of each session. Participants were educated that a normal response to new exercise is delayed-onset muscle soreness. Physiotherapists modified exercises by adjusting dosage, range of motion performed, or alternative exercises targeting the same muscle group if symptoms were reported. The second stage consisted of a maintenance program that included *resistance, weight-bearing, balance, and functional group exercises* that were not progressed in dosage or intensity.	
**Sobol et al.[149–151]**<br>**2016**<br>**2-arm RCT**	**Intensity:** 70%–80% of HRmax<br>**Duration:** 16-week<br>**Training load:** 60-min, 3 times/week<br>**Intervention proposal:** Before starting the exercise, the participants and their *caregivers* were invited to an *information meeting* regarding the exercise program and how to prevent overuse injuries. The participants conducted exercise in groups of *2–5* participants supervised by a *qualified physical therapist*. All exercise sessions included *a general warm-up and cool-down period*. The first 4 weeks focused on adaptation to exercising and included *strength training of the lower extremity muscles and an introduction to aerobic exercise*. The following 12 weeks included moderate-to-high–intensity aerobic exercise on *ergometer bicycle*, *cross trainer*, and *treadmill* in 3 periods of 10 mins with 2–5 mins pause in-between. The aerobic exercise was individually tailored and planned to achieve an intensity of 70–80% HRmax. To ensure training at the intended intensity, the *HR was monitored* during the aerobic exercise, which included 3 x 10 mins exercise and pauses (in total 34–40 mins), and the average HR was registered in a training *logbook*. The average exercise intensity for each individual was defined as: average HR of all sessions/maximal HR (%). In patients who were treated with beta-blockers, the *RPE* was used to ensure that the intended training intensity was achieved.	Demonstrated the positive effects on:<br>1. Cardiorespiratory fitness<br>2. Exercise self-efficacy
**Papatsimpas et al.[152]**<br>**2023**<br>**3-arm RCT**	Aerobic:<br>**Intensity:** 64%–76% of HRmax<br>**Duration:** 12-week<br>**Training load:** 30-min, 5 times/week<br>Resistance:<br>**Intensity:** 50%–69% of 1RM<br>**Duration:** 12-week<br>**Training load:** 40–45-min, 3 times/week<br>**Intervention proposal:** The intervention included 3 groups. The first intervention group A performed a *combined program of aerobic and resistance exercise*, the second group B *only resistance exercise*, and the third group C was the control group. The intervention program of groups A and B had different duration and frequencies for each type of exercise but with moderate intensity for both groups. In the combined program intervention group A, the aerobic exercise was performed at *home* and included *walking*. The *resistance exercise* concerned major muscle groups with limb weights at moderate intensity. The resistance exercise included 2 sets of 10 exercises of 12 repetitions, with a break of 1–3 min between sets. 1 of the 3 days of resistance training was performed at home while the other 2 were performed at the participants' recruitment center under the supervision of a physical therapist. Group B performed only resistance exercise. The exercise program with resistance was the same for both intervention groups (group A and group B). Resistance exercises included most of the major muscle groups such as *bicep arm curl, shoulder flexion, shoulder abduction, shoulder extension/hyperextension, triceps extension, hip flexion, knee extension, hip abduction, hip extension, and hamstring curl*s.	1. Significant intervention effects were observed for:<br>a) Global cognitive function<br>b) Instrumental activities of daily living<br>2. No significant effects were found between the combined and the resistance groups in any of the outcome measures

**Ullrich et al.[153]**<br>**2022**<br>**2-arm RCT**	**Intensity:** N/A<br>**Duration:** 12-week<br>**Training load:** N/A -min, 7 times/week<br>**Intervention proposal:** Participants in the intervention group performed a *home-based training program*. In addition, a motivational program for activity promotion was included. The physical training was easy to apply and feasible in a home environment and was based on basic exercises to improve key motor performances relevant for mobility autonomy such as *postural control* (side by side, semi-tandem, and tandem stance) and *strength* (tiptoe stance, sit-to-stand transfers/chair-rise, stair rise), and an *individualized walking* course to improve overall PA. During the first home visit, exercises and walking course were explained to the participant and practiced under the supervision of a *trained study instructor* (*academic sports scientists* experienced in geriatric rehabilitation). A *large poster* with a graphical description of the exercises and a comprehensive training manual was provided to support the autonomous training (independent continuation). Difficulty levels of the indoor exercises and of the outdoor walking course were *individually tailored* to the functional status and the environmental setting, thereby also considering safety aspects, and were progressively adapted during the course of the program via 4 more home visits and weekly phone calls by the training instructors. Participants were instructed to perform the training on a daily basis.	Demonstrated significant benefits on:<br>1. Physical capacity<br>2. Life-space mobility<br>3. Psychosocial status related to fear of falling and associated activity avoidance
**Bademli et al.[154]**<br>**2018**<br>**2-arm RCT**	**Intensity:** N/A<br>**Duration:** 20-week<br>**Training load:** 80-min, 4–7 times/week<br>**Intervention proposal:** Elderly people in the experimental group were provided with the program by a *psychiatric nurse* and an *associate professor* in sports health science. The "PA Program" included *10 mins of warm-up activities as the initial segment, 20 mins of rhythmic exercises as the activity segment, 10 mins of cool-down exercises as the final segment and 40 mins of free walking*. *Warming* is the mild bodily and mental activity of the muscles, joints, respiratory, and circulatory system performed at the beginning of the exercise session to get ready for active exercise. *Rhythmic exercise* was the phase in which the exercise program was implemented. For this phase, moderate activities including 22 simple movements (3 to 6 ME) were chosen to speed up the respiratory, circulatory, and locomotor systems, the phase was first started with the correct breathing, then continued with stretching and stamina exercises (repeating each muscle group 4 or more). *Cool-down* exercises were performed to enable the lactic acid accumulated in muscles and blood to return to their normal levels quickly to lower gradually the HR and BP, which increased after the activity program. The exercises were finished after 10 mins of low intensity. All exercise sessions were conducted in the presence of researchers and special attention was paid to the participation of the entire experimental group. *For walking*, a suitable area was selected for the participants to walk easily with normal steps and in balance. This exercise should be performed in an appropriate time (apart from the appropriate time for the elderly, the weather conditions should be also appropriate, and the walking activity should be performed approximately *2 hours after the meals*). During the walking exercise, all of the participants wore a *pedometer*, and the number of their steps was recorded. The elderly were divided into 5 groups of *6* persons. All of the activities in the program were performed as a group of individuals. The sessions were conducted by 3 researchers. During the implementation, the psychiatric nurse had *presession meetings* with the elderly people. During each session, the psychiatric nurse performed physical activities with the participants. During each session, the associate professor in sports, health science showed how to perform these activities.	Increased:<br>1. Cognitive functions<br>2. Sleep quality

**Lok et al.[155]**<br>**2023**<br>**2-arm RCT**	**Intensity:** Moderate<br>**Duration:** 12-week<br>**Training load:** 30–40-min, 5 times/week<br>**Intervention proposal:** The PA program in the form of 30 mins of musical exercise (*10 mins warm-up, 20 mins of exercise, 10 mins of cool-down*) 3 days a week and 40 mins of walking 2 days a week. *Musical rhythmic* exercise, warm-up included light physical and mental activities performed at the beginning of the exercise session to prepare the muscles, joints, respiratory and circulatory system for active exercise. Rhythmic exercise with music is the stage where the exercise program is implemented. In order to accelerate the work of the respiratory, circulatory and movement systems, activities that include 22 simple movements of moderate intensity (3–6 ME) were selected and stared with correct breathing first, followed by *endurance, strengthening, stretching and balance exercise* (4 or with more repetitions per muscle group). Cool-down were finished after 10 min of low-intensity exercise. All exercise sessions were conducted in company with the researchers and special attention was paid to the participation of the entire experimental group. *Walking exercise* (40 mins): a suitable area was chosen in a way to walk with normal steps, balanced and easy, and it was done at the appropriate time, except for the appropriate time for the elderly, when the weather conditions are suitable, *2 hours after meals* and the environmental conditions are at suitable temperature. During the walking exercise, each elderly person was fitted with a pedometer, and their steps were checked using the *pedometer*.	1. Increased cognitive function<br>2. Reduced depression levels
**De Sa et al.[156]**<br>**2024**<br>**3-arm RCT**	**Intensity:**<br>Aerobic sessions: 60%–70% HRR<br>Strength sessions: 40%–70% 1RM<br>**Duration:** 24-week<br>**Training load:** 60-min, 2 times/week<br>**Intervention proposal:**<br>The *Physical exercise* (PE) group participated in a multimodal physical exercise protocol, predominantly aerobic and comprising the work of the components of functional capacity. The sessions were developed in a 1:2 ratio (*1 strength training session to 2 aerobic training sessions*), with 16 and 32 sessions, respectively. In order to conduct the operationalization of *strength training*, the exercises encompassed all major muscle groups and were divided into the adaptation phase, specific phase aimed at increasing muscle strength, and intensity adjustment throughout the phase; this phase was divided into 3 protocols in order to have variability in the types of exercises. The intensity of the strength training sessions was managed with variations from mild (40% of 1RM) to vigorous (70% of 1RM) and were preceded by a general warm-up. *The aerobic training protocol included sessions interspersed with strength exercises*. The initial duration of each session was 15 mins at an intensity corresponding to 60% of HRR, progressing systematically in volume and intensity until reaching a duration of each session of 40 mins in the last 12 weeks, at an intensity corresponding to 70% of HRR.<br>With the *Motor task complexity* (MT) group combined between 2 dimensions: (1) the environment in which the person performs the skill and (2) the function of the action characterizing the skill. Each level of the taxonomy was scheduled to be worked with the patients for 3 consecutive sessions, with 12 variations of complexity exercises to be addressed per session.<br>The PE + MT was formed with the intention of proposing activities that addressed both interventions, performed with the same frequency as the other groups and following the same definitions and care described above. However, the protocols were readjusted so that they	Physical activity alone or combined with Motor task complexity protocol was effective in promoting improvements in:<br>1. Overall cognitive function<br>2. Lipid profile

	could be performed with the same length of time per session. This activity protocol was proposed in order to improve the standard of comparison with the other groups that participated in the group of physical exercises or motor task complexity, since the participants had the *same social and food stimuli* and thus observe the isolated effect of the other protocols or their combination. The participants were instructed daily to perform the proposed activities according to the group to which they were allocated, and all the proposed activities were performed under the uninterrupted *face-to-face guidance* of the researchers. The groups were accompanied in person and also by *telephone* contact by the researchers and, in case of any doubts or discomforts, were reassessed at any time, regardless of the days predefined in the schedule of interventions.	
**Padala et al.[157]**<br>**2017**<br>**2-arm RCT**	**Intensity:** N/A<br>**Duration:** 8-week<br>**Training load:** 30-min, 5 times/week<br>**Intervention proposal:** Subjects in both groups exercised under *caregiver* supervision. The Wii-Fit group performed exercises from 5 categories of the *Wii-Fit program*: *yoga, strength training, aerobics, balance games, and training plus*, which includes more complex exercise tasks. Each session in the Wii-Fit included a *warm-up, exercise, and cool-down phase*. During the warm-up and cool-down phases, subjects walked for 5 mins at a self-selected comfortable pace using the program's '*basic walk'* activity. The exercise phase was designed to be participant centric. The specific exercises in each category were picked based on their ease of use and subject preference expressed in our prior clinical work. All subjects started each exercise at level one. Upon mastery, subsequent levels were opened automatically by the program. Subjects were encouraged to choose one or more exercises from every Wii-Fit category during each session. *Research assistants* did an initial *home-visit* to set-up the program at the patient's home and trained the subject and their caregiver on the use of the program. *Caregivers* were instructed to help the subjects in setting up the instruments if needed and supervise them during each exercise session. They were also instructed to help the subjects record the activities performed daily in their activity diary. Subjects in the walking program were instructed to walk at their self-selected pace either indoors or outdoors.	Significantly improved balance
**Langoni et al.[158,159]**<br>**2019**<br>**2-arm RCT**	**Intensity:** 60%–75% of HRmax of walking training<br>**Duration:** 24-week<br>**Training load:** 50–60-min, 2 times/week<br>**Intervention proposal:** Groups consisting of *5 to 8* participants group exercise session, with load and intensity adjusted regularly. At all times, training sessions were conducted by a fully *qualified physical therapist*, who was aided by 2 *physical therapy undergraduate*s in their last year at university. The undergraduate students helped the participants perform the exercise correctly. The *physical therapist* was responsible for individually assessing and progressing exercise load and intensity according to the training protocol and the participants' performance. At the beginning and the end of the exercise sessions, *arterial BP and radial pulse* were measured and the participants performed stretching exercises. Once a week, participants wore *a HR monitor* to ensure training safety and maintenance of HR within individually determined training zones that were based on HRmax. The equipment used for the strength exercises *included ankle weights, resistance latex bands, and dumbbells*. Aerobic training involved 20 mins of walking at 60–75% HRmax for the first 4 weeks, then gradually increased to 30 mins by the 12th week. As the intervention progressed, maintenance of the HR	Showed significant improvement of:<br>1. Cognitive<br>2. Functional

	at the training frequency was obtained with an increase in the walking speed, observed in the distance walked. At the beginning of the study, *phone calls* were made to the Intervention group participants to remind them of the scheduled training sessions.	
**Zhang et al.[160]**<br>**2023**<br>**3-arm RCT**	**Intensity:** 60% of HRmax<br>**Duration:** 12-week<br>**Training load:** 60-min, 3 times/week<br>**Intervention proposal:** *Traditional Chinese exercise combined with rhythm training* (TCE + RTG): After completing the standard warm-up, the TCE + RTG subjects performed *joint stretching, aerobic exercise, acupoint massage, and a "close your eyes and adjust your breath" exercise* at a session. The intensity of the TCE + RT was low to moderate aerobic exercise, with a *HR belt* used to ensure the subjects' HR reached target HR. Walking group (WG): After finishing the same warm-up as the TCE + RTG, the subjects performed a 10-min relaxation phase. The intensity of the exercise in the WG was similar to that of the TCE + RTG. In addition, all participants in the intervention and CG participated in monthly *health science knowledge dissemination activities*, which included knowledge on diseases related to Cognitive Impairment in older adults, memory care, seasonal disease prevention, and prevention of common chronic diseases in older adults.	Both groups improved the cognitive function
**Vreugdenhil et a.[161]**<br>**2012**<br>**2-arm RCT**	**Intensity:** N/A<br>**Duration:** 16-week<br>**Training load:** N/A-min, 7 times/week<br>**Intervention proposal:** The exercise program involved *daily home-based exercises and walking*, under the supervision of their *carer*. The exercise program incorporates 10 simple exercises, each with 3 progressively challenging levels, focusing on *upper and lower body strength and balance training in addition to at least 30 mins of brisk walking*. Participants *were asked to exercise daily* if possible. For those in the exercise group, the person with dementia and their carer were both trained in the exercise program and provided with an exercise *manual* containing descriptions and illustrations of the exercises as well as safety instructions. During the trial, all participants (treatment and control) received a *telephone call* at around 2 weeks and again at 2 months to check on their well-being. Those who were exercising were also asked about their progression through the levels of exercising.	Improved functional ability (cognition, physical function and ADL)
**Papamichail et al.[162]**<br>**2024**<br>**2-arm RCT**	**Intensity:** Moderate<br>**Duration:** 12-week<br>**Training load:** 45-min, 2 times/week<br>**Intervention proposal:** The intervention study was carried out in a *private clinical setting* by a team of *physiotherapists*. Initially, information was given on the purpose, importance, and process of the study. The participants carried out the *Otago exercise program* under the supervision of the same experienced trained physiotherapist at the Day Care Centre of the Alzheimer Association. The program included *a warm-up, the main part, and recovery exercises*. The main part consisted of *muscle strengthening and balance exercises for the upper and lower limbs*. In particular, the intervention consisted of a warm-up stage promoting circulation and preparing the body for the rest of the program. Finally, stretching exercises develop flexibility and promote relaxation. The exercise program may include the following: *(1) easy marching, (2) head movements, (3) back extensions, (4) ankle movements, (5) front and back knee strengthening, (6) slide hip strengthening, (7) calf and toe raises hold, (8) toe*	The intervention benefited:<br>1. Muscle strength<br>2. Range of motion of both upper limbs<br>3. Functional status

	<u>*and heel walking, (9) one leg stances, (10) sideways walking, (11) sit to stand, and (12) back of thigh and calf stretches.*</u>	
**Rivas-Campo et al.[163,164]**<br>**2023**<br>**2-arm RCT**	**Intensity:** 80%–85% of HRmax<br>**Duration:** 12-week<br>**Training load:** 45-min, 3 times/week<br>**Intervention proposal:** The exercise program had 3 phases: first, a <u>*10-min warm-up*</u> composed mainly of joint mobility exercises; second, <u>*a 25-min core phase*</u> divided into 4 intervals, in which participants performed exercises at an 80–85% intensity of their HRmax that included <u>*bicycle-like limb movements from a seated position*</u>, wall push-ups in a standing position, chair squats, and ball throws against the wall while performing lateral and front lunges. Each exercise was performed for 30 seconds and as fast as possible avoiding any impact on the joints; then, the subject rested for 15 seconds before repeating the exercise. Each work interval had a duration of 4 mins with an active rest for 3 mins at 50–70% of the HRmax that included lateral walking activities alternating with heel raises, lateral and frontal upper limb raises, and functional diagonal reaches with trunk rotation. Finally, there was <u>*a 10-min cool-down*</u> with muscle stretching and relaxation and breathing techniques. HR was monitored with <u>*pulse sensors*</u> (<u>*Polar*</u> RS300Xsd) placed on the wrist of each participant. The <u>*trainer*</u> who led the activity had the logistical support of <u>*nursing assistants or trained persons*</u> who were assigned to the participants. Each assistant was assigned to a maximum of <u>*2*</u> subjects, who were supervised both during the workout and rest phase to ensure that the required intensity was achieved.	Significantly improvements in:<br>1. Aerobic capacity<br>2. Lower body strength<br>3. Decreased the risk of falls
**Prick et al.[165]**<br>**2017**<br>**2-arm RCT**	**Intensity:** N/A<br>**Duration:** 12-week<br>**Training load:** 30-min, 3 times/week<br>**Intervention proposal:** <u>*Dyads*</u> allocated to the intervention group received a multicomponent dyadic intervention consisting of <u>*physical exercise training and support existing of psychoeducation, a communication skills training, and a pleasant activities training*</u>. A <u>*personal coach*</u> who visited the dyads in their own <u>*home*</u>s for eight 1-hour-long sessions for 3 months delivered this multicomponent dyadic intervention. During the first month, the coach visited the dyads weekly followed by biweekly sessions over the next 8 weeks. During the physical exercise training, 4 types of exercises (<u>*flexibility, strengthening, balance, and endurance*</u>) were gradually instructed and practiced. The goal of the physical exercise component was to motivate dyads who have not been physically active to complete 30 mins of active exercise at least 3 days a week. Dyads who were already active were encouraged to increase or maintain their activity to complete 30 mins of active physical exercise at least 3 days a week and preferable on most days of the week. Each physical exercise training started <u>*with careful stretching, providing a warm-up*</u> for the other physical exercises, and increasing participants' awareness of their muscles. The goal of <u>*strength training*</u> was to maintain mobility and to safely perform daily activities such as standing up from a chair and walking. <u>*Balance exercises*</u> were designed to improve the safeness of participants' mobility. <u>*Endurance exercises*</u> were intended to maintain or improve overall physical health and mood. During the instruction visits, <u>*caregivers*</u> were taught by the coach to guide the person with dementia during the performance of the physical exercises. Each physical exercise was first demonstrated by the coach and then practiced by the person with dementia while the caregiver	A significant effect on the attention

	assisted the person with dementia and, if possible, the caregiver performed the physical exercises as well. To improve the attractiveness of the physical exercises for people with dementia, we added and integrated materials such as *a ball, weights, and elastics* to the original physical exercises based on a Dutch exercise protocol for people with dementia designed by *physiotherapists*. Caregivers in the intervention group completed *daily logs* to monitor their adherence to the physical exercise homework in the absence of the coach.	
**Hauer et al.[166–168]**<br>**2012**<br>**2-arm RCT**	**Intensity:** 70%–80% of 1RM<br>**Duration:** 12-week<br>**Training load:** 120-min, 2 times/week<br>**Intervention proposal:** The training was individually adjusted for age- and illness-related deficits to prevent overexertion. The participants underwent a regimen of *progressive resistance and functional training*, as described previously. Resistance training was targeted at functionally relevant muscle groups at a submaximal intensity in groups of *4 to 6* participants supervised by a *qualified instructor*. In an adjacent functional training, participants were trained to perform basic ADL-related motor functions such as *walking, climbing stairs, sitting down and standing up*, progressing to advanced levels of functional tasks.	Overall improvements in:<br>1. All strength<br>2. Functional parameters related to trained muscle groups or functions such as pastural control and dynamic balance, transfer ability, and walking
**Suttanon et al.[169]**<br>**2012**<br>**2-arm RCT**	**Intensity:** N/A<br>**Duration:** 24-week<br>**Training load:** N/A -min, 5 times/week<br>**Intervention proposal:** The *individualized home-based exercise* program supervised by a *physiotherapist*. The program included *standing balance and strengthening exercises and a graduated walking program* and was based on an existing home exercise program. The total number of home visits by the physiotherapist was increased from 4 to 6 visits. This provided increased support throughout the 6-month duration and maximized the participants' and caregivers' understanding of the exercises and of safety issues, particularly during the early phase. Each participant also received an exercise *booklet* with illustrations and instructions and was encouraged to complete the exercises 5 times a week. At the first visit, the physiotherapist selected and modified exercises from the *Otago Program* to address the individual's balance and mobility problems as identified in the baseline balance and mobility assessment. At each subsequent home visit, the physiotherapist monitored and modified the exercise program as required and answered any questions. *Caregivers* were also instructed regarding the exercise program and asked to encourage regular (5 days/week) and correct performance of the exercises. Follow-up *phone calls* by the physiotherapist in between visits were also provided to offer reassurance, to enquire if there were any negative effects from the exercises (such as falling or other physical injuries) and to answer questions about the exercises (5 phone calls over the 6-month period).	1. May help improved:<br>a) Balance performance<br>b) Mobility performance and<br>2. Reduced falls risk
**Sanders et al.[170]**<br>**2020**<br>**2-arm RCT**	**Intensity:** 9–16/20 RPE, 57%–89% of HRmax<br>**Duration:** 24-week<br>**Training load:** 30-min, 3 times/week<br>**Intervention proposal:** The aerobic sessions consisted of *outdoor walking*. If the weather did not allow for outdoor walking, or the participant rejected outdoor walking, walking was performed indoors. Subjects in the exercise intervention exercised at low intensity (LI) for the first 12 weeks and at high intensity (HI) for the subsequent 12 weeks. The target intensity of sessions was determined in correspondence with the *ACSM* guidelines for "low" and "moderate-to-high" intensity exercise. The intensity of the aerobic sessions was monitored	Significantly improved gait speed

	objectively every 5 mins using a *HR Wrist Band*. Subsequently, training intensity was determined objectively using the percentage of HRmax and subjectively with observer-determined RPE. In the LI phase, the target RPE was 9–11 and target HR was 57–63% HRmax. In the HI phase, participants performed interval training with alternating 4-min peak performance at RPE 15–16 and 83%–89% HRmax and 3-min active rest at RPE 13–14 and 71%–77% HRmax. Although we do not deem the observer-rated *RPE* to be superior to HR measures when determining exercise intensity, we instructed the research assistants to rely on the observer-rated RPE in case of discrepancies between RPE and HR which could be due to beta blockers. Walking intensity could be increased or decreased by adapting walking speed and the number of passive or active rests. Four lower limb exercises were performed during the *strength sessions* in a fixed sequence: *(1) knee extension while sitting, (2) plantar flexion (toe standing), (3) hip abduction (side leg lifts), and (4) hip extension (back leg lifts).* A *chair* was used for support. Per session, the muscle contractions were either isometric, concentric, or eccentric (so that 12 isometric, 12 concentric, and 12 eccentric contraction sessions were offered throughout the exercise intervention). The target RPE was used only to determine the intensity because no significant increases in HR were expected. The intensity of the strength sessions was determined subjectively with the observer-determined RPE. In the LI phase, the target RPE was 9–11. In the HI phase, the RPE was 13–16. Exercise intensity could be increased or decreased by adapting the number of sets and repetitions. *Ankle weights* were added in the HI phase per 0.5 kg for all exercises except toe stands. The added weight was increased equally for all exercises (except toe stands).	
**Dawson et al.[171]**<br>**2019**<br>**2-arm RCT**	**Intensity:** 60%–80% of 1RM<br>**Duration:** 12-week<br>**Training load:** N/A -min, 2 times/week<br>**Intervention proposal:** The intervention was a moderate-intensity *home-based functional exercise* program, consisting of *strength and balance exercises*. Central tenets of the *Strength-Based* Approach include (1) *identifying strengths and abilities*, rather than deficits and limitations (using familiar and functional activities that rely on procedural and long-term memory as exercises instead of new and unfamiliar fitness equipment); (2) *including individuals as active* rather than passive participants in treatment process (allowing the individual to choose the activity, such as ballroom dancing instead of walking on a treadmill); and (3) *emphasizing current possibilities and options* rather than past events and performance (encouraging continuation of enjoyed activities even if it will require some modify cation, such as addition of adaptive equipment for gardening). The *functional strength and balance program* was delivered individually in the participant's home by a *trained practitioner*, with occasional assistance from the *caregiver*. Each of these practitioner-led sessions was composed of 4 elements: (1) *review*, which examined results from previous sessions and identified the barriers to exercise completion reported by the caregiver or participant; (2) *education*, which initially outlined the purpose of the intervention and provided subsequent education to improve adherence; (3) *planning*, which allowed the exercise practitioner to utilize available implementation strategies, based on the Strength-Based Approach, to overcome barriers reported by the participant or caregiver; and (4) *activity*, which delivered a tailored functional	Improvements were noted on:<br>1. Balance<br>2. Lower extremity strength<br>3. Fast gait speed

	strength and balance program based on the participant's functional status. Initial starting exercises were determined from baseline walking performance. The practitioner and participant chose exercises based on the individual's needs and preferences. External *memory aids* (e.g., *written instructions and spots for foot placement*) to compensate for deficits in short-term memory were implemented on an individual basis, allowing a standardized but flexible intervention protocol. Over the first 2 weeks of the program, a 15RM was targeted to allow acclimation to exercise and act as a build-up process. This intensity is representative of 50% of an individual's 1RM. Target intensity of strength exercises following this initial phase ranged from 8 to 12 repetitions, representing 60%–80% of a 1RM; therefore, as more repetitions can be completed, the exercise intensity was increased appropriately. Once a participant was able to complete more than 12 repetitions of a particular strengthening exercise, the intensity was either increased by addition of a weighted vest, weighted belt or medicine ball, or increased by progression of activity (e.g., progress to floor to stand tasks). Intensity of balance exercises was altered by variation of base of support or increased compliance of surface to continue to challenge the participant's postural stability.	
**Santana-Sosa et al.[172]<br>2008<br>2-arm RCT**	**Intensity:** N/A<br>**Duration:** 12 -week<br>**Training load:** 75-min, 3 times/week<br>**Intervention proposal:** Exercises began at individualized, very light intensities. *Music* (from the patients' youth years) accompanied each session. Each session was supervised by an *exercise scientist* working with groups of *4* patients and started and ended with *a very light 15-min warm-up and 15-min cool-down* period, respectively, consisting of walking without reaching breathlessness (on an inside walking trail) and "gentle" stretching exercises for all major muscle groups. The *core portion of the training session was divided into joint mobility, resistance, and coordination exercises* that were consistently performed in the aforementioned order. *Joint mobility exercises focused on shoulder, wrist, hip, knee and ankle joints*. *Resistance training included 9 exercises with elastic medium-resistance bands* (3 sets of 15 repetitions each) engaging some of the major muscle groups: *chest, biceps, triceps, shoulder, knee extensors, abductor and adductor muscles, and calf muscles*. All exercises were performed through the full range of motion normally associated with correct technique for each exercise. *Stretching exercises of muscles* involved in an exercise were performed at the end of each set of resistance exercises. *Coordination exercises* were performed with *foam balls* of gradually decreasing size over the program, e.g., *bouncing a ball with both hands, tossing and catching a ball*, etc.	Significant improved overall functional capacity (muscle strength, flexibility, agility and coordination while moving, endurance fitness and ADLs)
**Lamb et al.[173,174]<br>2018<br>2-arm RCT**	**Intensity:** N/A<br>**Duration:** 48-week<br>**Training load:** 60–90-min, 2 times/week<br>**Intervention proposal:** *Physiotherapists and exercise assistants* prescribed and delivered interventions in the exercise arm. People with dementia attended an individual assessment where the prescription of aerobic and strength exercises was tailored to their fitness and health status. Participants attended group sessions in a *gym*. We also asked the participants to do home exercises for one additional hour each week during this period. The supervised program lasted 4 months, after which we prescribed a more frequent home-based program with a target of unsupervised PA or exercise of 150 mins each week (total). We used behavioral strategies to promote adherence throughout, and up to 3 *telephone* motivational interviews were	1. Did not slow cognitive decline<br>2. Improved physical fitness in the short term, but this did not translate into improvements in activities of ADL

	administered after the supervised program. *The behavioral strategies included guiding participants to choose home exercises or activities that matched their preferences for venue, personal situation, and ease of completion*. During the supervised period, people with dementia were overseen in groups of *6 to 8* participants to minimize costs. In each group session, *aerobic exercise consisted of static cycling* with a 5-min warm-up period followed by up to 25 mins of moderate to hard intensity cycling, depending on tolerance level. We set target intensity using a 6-MWT. *Strength training consisted of arm exercises using handheld dumb bells*, including at least *a biceps curl and, for more able individuals, shoulder forward raise, lateral raise, or press exercises, and leg strength training exercises* using *a sit-to-stand weighted vest* (All Pro Exercise Products, FL) or a waist belt (Rehabus, Lerum, Sweden), or both. The starting weight for sit-to-stand varied between 0 and 12 kg depending on ability. The baseline target for strength training exercises was 3 sets of 20 repetitions. The sets had to be at least moderately difficult or hard to complete, and the weight was increased accordingly. In the ensuing sessions we added weight to ensure progression, with moderation of repetitions if needed.	
**Kovacs et al.[175]**<br>**2013**<br>**2-arm RCT**	**Intensity:** N/A<br>**Duration:** 48-week<br>**Training load:** N/A-min, 2 times/week<br>**Intervention proposal:** The multimodal exercise program was designed and delivered by 2 *physiotherapists* (with extensive experience in geriatric physiotherapy) with 2–4 participants in each group. The exercise class consisted of *5 mins of warm-up period with flexibility exercise of hips, knees and ankles followed by a progressive exercise program including strengthening and balance exercise and ended with cool-down for 5 mins*. The exercise program was based on *Otago Exercise Program* apart from that in our trial the exercises were assisted. The *strengthening exercises* directed to lower limb muscles playing role in the postural control such as *knee extensors, hip extensors, hip abductors and ankle muscles*, using the patients' body weight and ankle cuff weight starting with 0.5 kg (Thera-Team 0.5–1.0–2.0 kg). The resistance increased after the participants could complete sets of 10 repetitions for 3 consecutive serials. Two mins rest period was between sets, if needed. The *balance exercises* were closely related to everyday activity, including *stepping forward, sideway, and backward, walking with and without changing direction, timed stand practice (standing on one leg, tandem stance) and sit to stand practice*. Our exercise program included a *supervised walking program* to practice gait elements for once a week.	Significantly improved:<br>1. Static balance<br>2. Dynamic balance<br>3. Functional mobility
**Silva et al.[176]**<br>**2019**<br>**2-arm RCT**	**Intensity:** 70% of VO_2max or 80% of HRmax<br>**Duration:** 12-week<br>**Training load:** 60-min, 2 times/week<br>**Intervention proposal:** The multimodal training routine consisted of the *accomplishment of balance, aerobic, and strength training and stretching*. Balance training was performed for 5 mins and followed a protocol of *static balance exercises* of progressively increasing difficulty, with each position maintained for 30 seconds. The aerobic training was performed for 30 mins, consisting of a 5-min warm-up, followed by a 20-min main session of aerobic exercise and a final 5-min warm-down. The equipment used consisted of a *treadmill and weight-lifting machines*. Strength training was performed over 20 mins with 3 sets (8–12 repetitions, with and a 60-second interval between sets) of the following exercises: *horizontal leg press, sitting bench press, extensor chair, seated row paddling and exor chair*. Finally, participants	MCI patients showed a significant improvement in mobility and executive function, while these improvements were not observed in patients with AD

	performed a series of *stretching exercises* over a period of 5 mins (15 seconds in each position), prioritizing the mobility of the main joints (*ankles, knees, hip, lower back/spine and shoulders*). The training sessions were supervised by *physical trainers and physiotherapists*. The participants were monitored at 5-min intervals during the aerobic training and at the end of the third series of each exercise in the strength training using *Polar® frequency meters* and the *RPE*. *BP* measurements were recorded before, during and after each training session.	
**Levinger et al.[177]**<br>**2023**<br>**2-arm RCT**	**Intensity:** N/A<br>**Duration:** 24-week<br>**Training load:** 60–90-min, 2 times/week<br>**Intervention proposal:** In Exercise park intervention, participants underwent a supervised exercise intervention program using the *Seniors Exercise Park*. The *exercise park equipment* is *outdoor playground equipment* comprising multiple equipment stations that target a specific function or movement (upper and lower limb), range of movement, static and dynamic balance, or functional movement such as walking up/downstairs and sit to stand. Each class was supervised by a *qualified Accredited Exercise Physiologist* with the assistance of the *Diversional Therapist* (aged care staff). Each session consisted of 5–7 mins warm-up exercises, followed by 45–75 mins on the equipment stations. The exercise classes included 3–4 participants and was circuit based. *Morning tea (light refreshment)* was organized following the exercise sessions. The level of the exercise difficulty was tailored to the capabilities of each participant with the primary consideration of safety, with adjustment of the exercises difficulty throughout the program based on the individual participant progression. Exercise uptake and physical activity maintenance (maintenance phase: 12–24 weeks post baseline) After completion of the 12-week program, scheduled sessions were available for participants to access and use the Seniors Exercise Park under supervision. To facilitate independence and empowerment, participants were encouraged to exercise independently (supervised but unstructured sessions).	1. Increased:<br>a) Enjoyment<br>b) Positive attitude<br>2. High levels of engagement
**Ghahfarrokhi et al.[178]**<br>**2024**<br>**3-arm RCT**	**Intensity:**<br>High intensity functional training (HIFT): 75%–85% of HRR<br>Low intensity functional training (LIFT): 35%–45% of HRR<br>**Duration:** 6-week<br>**Training load:**<br>High intensity functional training (HIFT): 30–35-min, 3 times/week<br>Low intensity functional training (LIFT): 40–45-min, 5 times/week<br>**Intervention proposal:**<br>Subjects in the HIFT group performed functional training with an intensity higher than the lactate threshold (75%–85% of the HRR, equivalent to 80%–85% of the maximum oxygen consumption, equivalent to 120%–125% of the lactate threshold). Subjects were first to undergo a 1-week *acquaintance training course* (3 sessions/week) under direct supervision to learn about functional exercises and the *gym* environment and familiarize themselves with the equipment. Then, for 6 weeks, they continued functional exercises while being supervised by *physical trainers*. Each HIFT sessions included *1- endurance exercises, 2- upper and lower body strength, 3- balance exercises and maintaining posture, and 4- hip control exercises and mid-body stability*. The ratio of rest to activity time in this group was 1:1.<br>Subjects in the LIFT group performed functional training with an intensity lower than the lactate threshold (35%–45% of HRR, equivalent to 50%–60% of maximal oxygen consumption, equivalent to 70%–75% of lactate threshold). Subjects first undergo a 1-week acquaintance training course (5 sessions/week) under direct supervision to learn about functional exercises and the *gym* environment and familiarize themselves with the equipment.	HIFT improved variables related to cognitive function (global cognition, attention, speed, memory, and learning)

	Then, for 6 weeks, they continued functional exercises while being supervised by a *physical trainer*. The ratio of rest to activity time in the LIFT group was 3:1. Ten minutes of warm-up before each session and 5 mins of cool-down at the end of each session were considered. The HR was monitored by a *polar HR monitor* to control the training intensity during the training sessions. Additionally, lactate levels were evaluated after the end of each session to determine and adjust the level of intensity of exercise training.	
**Fonte et al.[179]**<br>**2019**<br>**3-arm RCT**	**Intensity:** 70% of HRmax, 85% of 1RM<br>**Duration:** 24-week<br>**Training load:** 90-min, 3 times/week<br>**Intervention proposal:** Exercise group included *7–8* patients with the same degree of cognitive decline. During the study patients were not allowed other types of physical training (PTs) or cognitive training (CTs). PT, conducted by *2 kinesiologists* (ratio 2:5), included moderate intensity *endurance and resistance training*. Sessions started with 15 mins of warm-up which included active joint mobilization and walking on treadmill at preferred speed. Then, patients performed 45-min of endurance exercises divided in: *15-min of cycling on cycle ergometer, 15-min of walking on a treadmill, 15-min of arm cranking on a specific ergometer with a random order*. Workload intensity was increased, if it was possible, by 5% every 6 weeks and was monitored by *HR monitor belt* and by the *RPE*. The RPE scale was not used to set the intensity, which was based on HR of the participant during each aerobic exercise, but it was used as an extra tool to monitor how patients perceived the effort during the aerobic training section. Furthermore, patients started with a low intensity aerobic training in the first PT sessions, aiming to reach the 70% intensity in 2 weeks. All the participants reached the required intensity within 2 weeks. Subsequently, patients performed 3 sets of 12 repetitions of resistance exercises for *isotonic ergometers* including *chest-press, lat-machine, leg-press*. Selected patients were all naïf to resistance training and due to the short *familiarization* (1 day) with exercise devices, the estimate of the one repetition maximum was likely underestimated. Therefore, during the first week of PT we asked the participants to perform as many repetitions as possible with the 85% of the estimated 1RM. Furthermore, as soon as participants were able to perform the 12 repetitions easily (that means they were able to execute more than 12 repetitions) the workload was increased by 5%. PT ended with *stretching exercises* for all the muscles involved in the training. The kinesiologists motivated the participants and gave patients time to perform the exercise as a whole.	1. The training slowed down the usual worsening of cognitive symptoms<br>2. Positive effects on memory and attention abilities in patients with MCI<br>3. General ameliorations of the cardiovascular risk factors and exercise capacity<br>4. Long term effects seemed to persist after the end of the treatments
**Gebhard and Mess[180]**<br>**2022**<br>**2-arm RCT**	**Intensity:** N/A<br>**Duration:** 12-week<br>**Training load:** 60-min, 2 times/week<br>**Intervention proposal:** The training sessions were held in small groups of *4 to 12* participants. The training regime was based on the *Otago Exercise Program* and included 10 simple and effective basic exercises to train *resistance and balance*: *knee extensors, sit-to-stand, climbing stairs, raise arms, raise legs sideward, toe-heel, moving sideward, moving backward, gure-of-8 walking, and tandem stance*. These basic exercises were *complemented by endurance sequences of walking*. Based on PA biographies and the wishes of the interviewed people with dementia, these 10 basic exercises and walking sequences were integrated into 4 meaningful activity topics, namely, *(a) nature, (b) dancing, (c) daily activities, and (d) sports and games*. To embrace the dementia specific approach, 6 key principles were defined for a didactical	Showed mixed effects on functional performance

	concept in institutionalized people with dementia: *(a) communicate in a tailored and respectful way, (b) convey joy and fun, (c) use meaningful music to trigger physical activity, (d) use nature as a stimulating environment for exercising, (e) have flexible timeframes, and (f) use familiar materials of a demanding character*. Together, these cornerstones led to the development of 33 exercise units, each combining basic exercises for resistance and balance with endurance sequences of walking. All exercise units were adaptable to 3 mobility levels (no impairments, mobile with stick or rolling walker, and mobile with wheelchair), and moreover, they were individually adjustable based on the participants' competencies, by changing the length of the walking distances, the sizes of the weights used, or the number of repetitions. All training sessions followed the same structure: all started and finished with *a 5-min activity poem for warm-up and cool-down and included at least 2 exercise units with a 5-min drinking break in between*. For training instruction, *specific communication strategies were applied. Communication strategies, for example, included simple and direct instructions, asking for one aspect at a time, using familiar words, speaking slowly, and repeating instructions several times*. *Verbal instructions* were complemented by demonstrating the movement patterns continuously and by tactile as well as rhythmic cues, or visual as well as acoustic signs. Furthermore, an individual *music* playlist was composed for each training group. The participants profiles, 24 individualized training sessions, and an *audio CD* were included in an individualized training manual for every training group, and this built the foundation for the implementation of the intervention. A team of at least 2 *educated nurses* led the training sessions, supported by weekly mentoring. Based on the training manual, an educational program was developed, which enabled nursing home staff to implement the physical activity program developed in a high-quality and safe way.	
**Koc et al.[181]**<br>**2024**<br>**3-arm RCT**	**Intensity:** N/A<br>**Duration:** 12-week<br>**Training load:** 60-min, 0.5 times/week<br>**Intervention proposal:** A Supervised Physical Exercise Program (SPEP) was conducted *online* with the patient under the supervision of an *expert physiotherapist*. This online SPEP included *a warm-up period, range of motion (ROM) and stretching exercises, strengthening exercises, balance and flexibility exercises, aerobic exercise and a cool-down period*. All exercises except strengthening were started with 3 repetitions once a day in the first week. The number of repetitions increased weekly until the 5th week and was kept constant between the 5th and 12th weeks. To observe the patients, strengthening exercises were not started in the first week, but were included in the exercise program from the 2nd week. Furthermore, the number of repetitions of strengthening exercises was increased per week as of the 2nd week and kept constant after the 6th week. SPEP was carried out with *videoconferencing one-on-one sessions* like synchronous video calls under the supervision of the primary physiotherapist using software such as *Zoom™* (Zoom Video Communications Inc. 2016) and *WhatsApp*. Patients and *caregivers* were instructed that they should connect in the same *home environment* as much as possible during the entire treatment period sessions. In the exercise program, the durations allocated to the exercises were determined to include resting periods. During the online exercise sessions, rest *breaks* were given according to the needs of the patients. Furthermore, it was stated that they should inform the physiotherapist in case of any pain, fatigue, dizziness, etc. When there were technical problems that could not be solved, such as connection problems, or when the patient said that he/she did not feel well at the beginning of	Improved:<br>1. Balance<br>2. Functional mobility<br>3. Upper extremity muscle strength

	the session, the session was canceled and postponed to another day. The patients' needs were taken into consideration during the 1-hour exercise sessions and caregiver support was not restricted during exercise. In addition, while the patients were doing the exercises, *caregivers* were told to stand by the patient in any adverse situation. Especially during balance exercises and complex movements, caregiver assistance was requested according to the needs of the patients. In addition, care was taken to ensure that patients wore non-slip shoes or socks and that no objects or items around them would cause them to slip during the online exercises.	
**Casas-Herrero et al.[182]**<br>**2022**<br>**2-arm RCT**	**Intensity:** N/A<br>**Duration:** 12-week<br>**Training load:** 30-min, 5 times/week<br>**Intervention proposal:** The intervention group received the recently developed *Vivifrail multicomponent exercise program*. The Vivifrail program is a *home-based exercise program* focused on individualized multicomponent exercise prescription according to the functional capacity of the older adults and consisted of resistance/power, balance, flexibility and cardiovascular endurance exercises (i.e. walking). 2 *phone calls* were performed during the intervention period to guarantee patient adherence and to address doubts and questions related to the intervention. At the end of the baseline visit, patients were familiarized with their specific exercise routine before the start of the intervention, and their *family members or caregivers* were instructed in monitoring the exercise intervention. After the baseline assessment, patients were enrolled into one of the following individualized Vivifrail training programs, according to their physical functional status: Disability (0–3 points in the SPPB score), Frailty (4–6 points), Prefrailty (7–9 points), and Robust (10–12 points). A copy of their specific exercise protocol was delivered for each patient. The initial load for *resistance exercises* was established according to the Vivifrail exercise prescription guidelines through a progressive loading protocol, adjusting the load until the patient was able to complete ~30 repetitions with some effort. Initial load was set at 0.5 kg (*dumbbells*) and gradually increased in 0.5 kg increments for upper-body exercise; lower-body leg extensions started with free weight repetitions and gradually increased in 0.5-kg increments using ankle weights to gradually increase the intensity of lower-body leg exercises based on the functional reserve of the older patients. The exercise intervention comprised a 5-day-a-week routine of multicomponent exercises (i.e. *resistance, balance, and flexibility exercise 3 days per week and walking 5 days per week*).	1. Provided significant benefit over usual clinical care<br>2. To prevent or reverse the functional decline<br>3. Promoted:<br>a) Mood<br>b) Cognitive<br>c) Muscle function enhancements
**Shaw et al.[183]**<br>**2021**<br>**2-arm RCT**	**Intensity:** N/A<br>**Duration:** 8-week<br>**Training load:** 45-min, 3 times/week<br>**Intervention proposal:** The sessions for the group exercise began with *a 5-min warm-up* period involving 5 gentle range of motion exercises, such as trunk rotations, performed for 3 sets of 10 repetitions each, followed by *15 mins of resistance training* using 12 exercises, such as straight leg raises, performed with *elastic bands and Pilates balls* for 3 sets of 10 repetitions each. The participants then went on to perform 9 *aerobic exercises*, such as *marching on the spot, for approximately 15 mins*, followed by *5 mins of flexibility exercises* using 5 exercises, such as a neck scapulae stretch, followed by *a final 1-min cool-down* consisting of inhalation	1. Positively influenced cognitive function<br>2. Improved ADLs

	and exhalation accompanied by relaxing arm movements. All intensities for the resistance, aerobic, and flexibility portions of the program were *self-selected*, which allowed for individual adaptation and progressive overload. In each training session, the *caregivers and/or legal guardians* were required to be on standby but were not required to be present. Each session was fully supervised by the same *researcher*.	
**Cezar et al.[184,185]**<br>**2021**<br>**2-arm RCT**	**Intensity:** N/A<br>**Duration:** 16-week<br>**Training load:** 60-min, 3 times/week<br>**Intervention proposal:** The participants were given a week to become *familiar* with the home-based protocol to enable their muscles to adapt to training as well as avoid pain and muscle injuries. Three sessions were held on non-consecutive days. The following week, the *AD-HOMEX* composed of functional exercises that resemble ADLs aimed at *strengthening, balance, aerobic endurance and dual cognitive-motor tasks*. Although the protocol involves the progressive load of strength and balance every 3 sessions, this protocol was individualized, taking into consideration self-reported tolerance, wellbeing (absence of reported pain and fatigue) and the execution quality of the exercises. Each session was performed in an *individual*, face-to-face manner. AD-HOMEX was administered by 4 *physiotherapists* who had undergone training exercises and had experience in geriatrics. Each participant with a single physiotherapist to ensure the creation of a bond. *HR was monitored* continuously throughout each session and *BP* was determined at both the beginning and end of the session. Each session involved *7 mins of warm-up, followed by 45 mins of strength and balance exercises, ending with approximately 8 mins of cool-down*. Strength training was directed at the main lower limb muscles. Resistance was obtained using ankle weights and the force of gravity for load. Strength training involved alternating between 2 options: *(A) squats, knee flexion, reaching exercise and going up and down stairs using a step platform; and (B) sitting down and standing up from a chair, knee extension, a reaching exercise and moving up & down on the balls of the feet and heels*. Three sets of 12 repetitions were performed for each exercise. Balance training consisted of a *semi-static exercise*, static to dynamic transference of weight, tandem gait along a line on unstable surfaces, the transference of objects and the avoidance of obstacles. Each participant was observed with regards to the correct execution of the exercises (when performed well, the degree of difficulty was increased). To enhance aerobic endurance and physical fitness, the physiotherapist instructed the participant to increase the velocity of the exercises while monitoring the *HR*. Other aspects included a reduction of sensory inputs (vision and tactile), reduction of the support base, an increase in the complexity of the movement, the addition of a dual cognitive-motor task and a change in the position of the center of gravity. *Postural guidance and tips* on how to recover one's balance, such as trunk and ankle strategies, were given. Manual support was provided, when necessary, such as holding onto the therapist in situations of imbalance. However, efforts were made to diminish this practice gradually. Commands were given in short phases to facilitate the participant's understanding.	1. Improvements in:<br>a) Lower limb muscle strength<br>b) Lower limb muscle functioning<br>2. Reductions in the risk of:<br>a) Falls<br>b) The severity of functional limitation
**Cardalda et al.[186]**<br>**2019**<br>**3-arm RCT**	**Intensity:** 15RM<br>**Duration:** 12-week<br>**Training load:** 60-min, 2 times/week<br>**Intervention proposal:**<br>The participants assigned to *Therabands*® (TG) carried out a program of strength-based physical exercise focused on the lower limbs, by means of *resistance elastic bands* (TheraBands®). The sessions consisted of *10 mins of warm-up* exercises working on the	TG group generated a tendency of improvements in:<br>1. Cognitive state<br>2. Functional independence<br>3. Mental and physical component of the SF-12

	mobility of the ankle, knee and hip joints; *a main part of 45 mins*, which consisted of 10 strengthening exercises for the lower limbs (*plantar and dorsal flexors, flexors and knee extensors, flexors, extensors, abductors and hip rotators*) of which 2–4 is used per session; *a 5-min cool-down*, with stretching of the aforementioned muscles. During weeks 1&2, 2 exercises were undertaken in the main part, which were 2 sets of 10 repetitions with *breaks* of 40 seconds between sets, and of 60 seconds between exercises. During weeks 4–6, 3 series of 3 exercises were undertaken, this time of 15 repetitions, with the same intervals. During weeks 7–8, 2 exercises were performed with the same number of repetitions, sets and amount of rest. Weeks 10–12, 4 exercises were performed with the same number of repetitions and sets, but shorter periods of rest, of 30 seconds per set, and 50 seconds between exercises. This program was undertaken in 3 groups, made up of *8, 8 and 9* participants respectively.<br>Participants in the *Multi-Calisthenics* (MG) took part in a traditional physical exercise program specifically focused on the lower limbs. The exercises in this program of multi-calisthenics were performed mostly in the seated position. The exercises that were performed in the standing position required assistance. It should be emphasized that the objectives of the session of playful callisthenic games were not only for enjoyment and social integration, but also for improvement in physical condition (strength, stamina, speed and flexibility). This program was undertaken in 3 groups, made up of *8 and 7* participants respectively. In both the TG and the MG, the activities were conducted and controlled by *health and fitness professionals* specializing in physical activity for older adults.	
**Bo et al.[187]**<br>**2019**<br>**4-arm RCT**	**Intensity:** 13–15/20 RPE<br>**Duration:** 12-week<br>**Training load:** 50-min, 3 times/week<br>**Intervention proposal:** The design of physical exercise included the following 3 sessions: *(1) a warm-up period (5–10 mins) of aerobic exercise, such as jogging or cycling; (2) the main component (30–35 mins) of exercises of endurance, strength, and balance; and (3) a cool-down period (5 mins) of stretching and exercises* to recover normal cardiac levels. Participants were monitored throughout the session using a *HR meter* to ensure the proper intervention zone. Each of the participants exercised at a self-determined moderate intensity level using the *RPE*.	Significant changes in cognitive performance
**Kim et al.[188]**<br>**2016**<br>**2-arm RCT**	**Intensity:** 40%–60% of HRmax, 11–13/20 RPE<br>**Duration:** 24-week<br>**Training load:** 60-min, 5 times/week<br>**Intervention proposal:** The Kohzuki exercise program (KEP) consisted of *15 mins of warm-up and stretching, 30 mins of lower-limb aerobic exercise using a TERASU-ERUGO ® (Showa Denki Co., Ltd., Osaka, Japan), and 15 mins of cool-down and relaxation*. The KEP was supervised by a *physical therapist*. All participants in the KEP+ Multi-component cognitive program (MCP) and MCP groups received the multicomponent intervention that consisted of *music therapy, art therapy, horticulture therapy, handicraft, recreational therapy, stretching, laughing therapy, and activity therapy*. All MCP interventions were supervised by 2 *caregivers* per each group. Each therapy was given equally to the KEP+MCP group and the MCP group quantitatively and qualitatively.	It was possible to improve cognitive function
**Bossers et al.[189,190]**<br>**2014**<br>**3-arm RCT**	**Intensity:** 12–15/20 RPE, 50%–85% of HRmax<br>**Duration:** 9-week<br>**Training load:** 30-min, 4 times/week<br>**Intervention proposal:**	1. Improved:<br>a) Cognitive function<br>b) motor function after

	Strength Exercises<br>Strength exercises for the combined group focused on *lower limb strengthening* because such large muscle group exercises are assumed to lead to enhanced positive responses in gait speed, balance, and mobility. The exercises were as follows: *seated knee extension, plantar flexion through toe raises while holding both hands of the trainer, hip abduction by moving the straight leg sideways while standing behind and holding onto a chair, and hip extension by moving the straight leg backward while standing behind and holding onto a chair*. Exercise intensity increased gradually by increasing the number of repetitions and by affixing weights around the ankles. To minimize the chance for injury, overload, and drop-out, all participants started with 3 sets of 8 repetitions for each leg without weights. When a participant performed an exercise with ease and according to protocol, the number of repetitions was increased to 10 in the next session and to 12 in the session thereafter. When a participant was able to correctly perform 12 repetitions without weights, at an *RPE* less than 12, a weight of 0.5 kg was attached to the ankles. After the weight was attached, participants performed 8 repetitions and progressed as prescribed above. The trainer increased weights from 0 kg to a maximum of 1.5 kg in 0.5-kg increments. For plantar flexion the number of repetitions increased in increments of 2 per session to a maximum of 30 repetitions.<br>*Aerobic Training*<br>The combined group and aerobic group performed moderate to high intensity *walking* sessions. Walking sessions usually took place indoors. However, if the weather permitted and the nurse gave permission, walking took place *out-doors*. If rest was requested, an appropriate rest period was included in the 30-min session. The training intensity was adjusted by varying the distances the participants walked per session.	2. An alternation form of aerobic and strength training sessions was more effective than aerobic-only training
**Telenius et al.[191,192]**<br>**2015**<br>**2-arm RCT**	**Intensity** 12RM with strengthening exercise<br>**Duration:** 12-week<br>**Training load:** 50–60-min, 2 times/week<br>**Intervention proposal:** *3 to 6* participants at each *nursing home* exercised with *physiotherapists* (1 physiotherapist per 3 participants). The exercise program was the *High Intensity Functional Exercises (HIFE)–program*. The exercise sessions consisted of *5 mins warm-up*, at least 2 *strengthening exercises* for the muscles of lower limb and 2 *balance exercises*. All exercises were *individually tailored*, instructed and supervised. The intensity of strengthening exercises aimed to be 12RM. The *balance exercises* intended to be “highly challenging”. Nine nursing homes (50%) received help from 1 or 2 external physiotherapists to be able to participate in the study. In total, 27 physiotherapists were involved in the intervention program and all of them had attended a course to learn about the HIFE-program. The course lasted 3 hours and included practical exercises. The importance of targeting high intensity was emphasized. Adjustable weighted belts (0.5–12 k) were made available to all participating nursing homes.	The positive effects on :<br>1. Balance function<br>2. Agitation
**Toots et al.[193–197]**<br>**2017**<br>**2-arm RCT**	**Intensity:** 13–15 RM<br>**Duration:** 16-week<br>**Training load:** 45-min, 2.5 times/week	No significant effect

	**Intervention proposal:** The exercise activities were conducted at the *nursing homes* in small groups (*n* = 3–8), each supervised by 2 *Physical Therapists* (PT) and 1 *Occupational Therapist* (OT) or *OT assistant*. The exercise intervention was based on the *High-Intensity Functional Exercise (HIFE) program*, which includes a model for exercise selection and a definition of exercise intensity, and it available from authors and described in detailed in elsewhere. The HIFE program comprises 39 functional exercise that aim to improve *lower limb strength, balance, and mobility*. Exercise is performed in weight-bearing position, e.g., *rising from a chair, stepping, and walking*, and selected depending on individual degree of functional deficit. The intensity in muscle strengthening exercises was defined relative to RM, i.e., "the maxima number of times a load can be lifted before fatigue using good form and technique". The supervising PTs estimated RM in each exercise set, based on observation of participant response and performance. High exercise intensity was aimed for and defined as 8-12RM, thus progressed when able to exceed 12 repetitions. *Strength exercises* were progressed by adjusted of load, for example, by altering the step or seat height, or by adding weights to weighted waist belt (maximum 12 kg). *High-intensity balance* exercises aimed to fully challenge postural stability, i.e., performed at or near the limit of maintaining an upright position, and were progressed by, for example, narrowing the base of support or changing the support surface. For safety, participants wore belts with handles so that PTs could provide support if needed when postural stability was fully challenged, thereby preventing falls. Participants were encouraged to exercise at moderate strength intensity (13–15RM) the first 2 weeks. At the end of each session leaders completed a structured protocol for each participant pertaining to adverse events and intensity achieved in muscle strength and balance exercises, which was estimated separately as high, moderate, or low according to the pre-defined scale.	
**Henskens et al.[198]**<br>**2018**<br>**4-arm RCT**	**Intensity:** N/A<br>**Duration:** 24-week<br>**Training load:** 30–45-min, 3 times/week<br>**Intervention proposal:** *Qualified movement teachers* guided the *multicomponent exercise training*. Sessions were alternated weekly (e.g., a week of 2 strength and 1 aerobic session, followed by a week of 1 strength and 2 aerobic sessions). Each session with a progressive increase in intensity*. Strength exercises* were performed from a *seated position* and concentrated on *upper extremities* (i.e., *chest press, side and front raises, triceps/biceps curl, seated row*), *lower extremities* (i.e., *squats, calf raises, seated leg extension, standing rear leg raise, hip abduction*), and the *torso* (i.e., *chair crunch, seated back extension*). Exercises were intended to start with 3 sets of 8 repetitions, which could be increased to 10, 12, or 15 repetitions, and subsequently with additional weights, only after correct execution. The *aerobic training* consisted of *outdoor walking* sessions. Participants were assigned to either a short walking route (500 m) or a long route (1 km), depending on their performance on the 6MWT. In a group of *4–6* participants, 1 movement therapist guided 2 participants to guarantee individual attention and safety, to optimally match participants on their walking pace and to minimize differences in fitness level within a group. The intensity of the training was increased by increasing either the walking distance or the walking speed by means of interval training.	Benefit of grip strength only of participants with mild-to-moderate cognitive impairment (MMSE score 15–23)
**Kemoun et al.[199]**<br>**2010**<br>**2-arm RCT**	**Intensity:** N/A<br>**Duration:** 15-week<br>**Training load:** 60-min, 3 times/week	1. Slowed cognitive decline<br>2. Improved quality of walking

	**Intervention proposal:** During the first 2 weeks, the participants were prepared for PA through exercises involving articular mobilization and muscle stimulation. Evaluation of individual aptitudes constituted the initial step of orienting content programming by defining an individual motor profile. Next, for 13 weeks, we elaborated a program based upon exercises *in walking, equilibrium and stamina*. Three themes were taken up in each session in accordance with our objectives, and they occasioned 40 mins of exercises per session. Each week,1 session was given over to walking and the amelioration of walking parameters through motor route exercises (e.g. walking by striding over boards, going up a step, zigzagging). After that, a second weekly session was devoted to stamina exercises, particularly on an *ergocycle* with the arms and the legs. Participants could thereby keep up a light to moderate effort over a relatively long-time span. During this effort, the patients were equipped with a *Beurer P-100 cardio frequency meter glove*. While unable to carry out an effort test, we nonetheless managed to determine the targeted cardiac frequencies encompassed between 60% and 70% of reserve cardiac frequency. Finally, the last session of the week involved activities (e.g. *dance and stepping*) that combined stamina, equilibrium and walking. This session was supposed to be enjoyable, as its objective was to maintain the motivation of one and all throughout the duration of the program. The hour was divided into 3 parts: *the first part consisting of 10 mins of contact, articular mobilization and warm-up, the second part was 40 mins of the previously described activities and then 10 mins of return to calm and relaxation*. The follow-up necessitated preliminary programming of each session and allowed for activity content to evolve in accordance with each person's progress.	
**Barreto et al.[200,201]**<br>**2017**<br>**2-arm RCT**	**Intensity:** Moderate<br>**Duration:** 24-week<br>**Training load:** 60-min, 2 times/week<br>**Intervention proposal:** *Exercise instructors* (3-year university diploma in PA) had experience working with institutionalized people with dementia. *Group-based* exercise interventions took place in the *Nursing Homes*. The exercise was a multicomponent training: *10 mins of warm-up (e.g., range of motion), 10 mins of coordination and balance exercises (e.g., short walks with direction changes), 10–15 mins of muscle strengthening (e.g., weightlifting), 20 mins of aerobic exercise (mostly walking), and 5–10 mins of cool-down*. Exercise intensity was targeted to be moderate. Instructors endeavored to establish progression individually; visual cues were used, and participants were regularly encouraged to improve their performance in the absence of pain or breathlessness. When a subject had improved the execution of an exercise, a progression was proposed by increasing exercise difficulty, the number of repetitions to be performed, or exercise load.	No significant difference
**Roach et al.[202]**<br>**2011**<br>**3-arm RCT**	**Intensity:** N/A<br>**Duration:** 16-week<br>**Training load:** 30-min, 5 times/week<br>**Intervention proposal:**<br>*Activity specific exercise*<br>The exercise program had 4 components: *strength, flexibility, balance, and endurance*. *The strength and flexibility exercises were designed to strengthen the trunk and lower extremities by performing eccentric and concentric work in moving the body against gravity*. The *first* of these exercises was hip and knee bends followed by toe rises performed with subject and intervener facing each other in dance position. The muscle groups involved in this activity are the same ones required to perform a sit-to-stand or stand-to-sit transfer. The second exercise	Preserved or improved the ability to perform transfer and mobility activities

	was performed with both the subject and intervener seated facing each other and required the subject to lean forward while pushing against the intervener and then lean backward while pulling against the intervener. This exercise was designed to increase trunk strength and as well as hip and trunk flexibility to facilitate the subject's ability to shift the center of gravity over the base of support as is required to transfer sit to stand. The *second* set of exercises focused on balance and weight shifting. The subject and intervener performed the exercises in standing with the intervener facing the subject as if in dance position. *Subjects placed their hands on the interveners' shoulders, and the interveners placed their hands on the gait belt fastened around the subject's waist*. The subjects and interveners then side stepped to the left and to the right, walked backward and turned in a complete circle. All exercises began with 2 to 3 repetitions and progressed to 7 to 9 repetitions over the 16-week intervention period based on a predetermined schedule. The amount of assistance provided was reduced and the amount of resistance was increased as the subject improved in their ability to perform the exercise. Total contact time with the subjects in the exercise group started at 15 mins and increased to 30 mins by the end of the intervention period. *The final aspect of the comprehensive exercise program was a supervised walk*. The subject was allowed to use an assistive device and/or moderate physical assistance and to rest as needed. Verbal cues and physical assistance were given to encourage the subject to attend to the walking task. The duration of the walk started at 10 mins and progressed to a maximum of 20 mins.<br>*Supervised Walking*<br>Subjects assigned to the walking group performed a *supervised walking* activity. They were allowed to use an *assistive device* and were provided with physical assistance as required. Participants were allowed to walk their usual pace and were permitted to rest as needed. Walking sessions initially lasted 15 mins. The pace, distance, and duration of the walking session were increased incrementally up to a maximum of 30 mins and were matched to the duration of contact in the activity specific exercise group.	
**Rolland et al.[203]**<br>**2007**<br>**2-arm RCT**	**Intensity:** N/A<br>**Duration:** 48-week<br>**Training load:** 60-min, 2 times/week<br>**Intervention proposal:** The same *occupational therapist* conducted all exercise sessions. Each exercise group consisted of *2 to 7* subjects (mean 5.2) selected according to their baseline physical performance scores, MMSE score, and behavior disturbances and affinity between participants. Exercises began at light intensity and gradually increased over the first month of the intervention. Exercises were *individualized*, based on the participants' behavioral readiness for the proposed program. *Music* accompanied the sessions. The exercise program included *aerobic, strength, flexibility, and balance training*. During enrollment in each nursing home, an *inside circular walking trail* was created and adapted for each exercise group. This trail went past the room of each exerciser to enhance adherence to the session. Participants were grouped on the course and encouraged by the occupational therapist. The same trail was used during the 12 months to ritualize the session and encourage confidence. Walking was required for at least half of the session. After stretching warm-up, *subjects were encouraged to walk fast to reach moderate breathlessness but not exhaustion. The session was interspersed with strength,*	A improvement in mean walking speed

	flexibility, and balance training at predetermined stations along the trail where *guardrails in the corridor or foam rubber ground sheets* could be used for safety. Strength training was adapted to the participant and focused on lower extremity strength. Exercises included *squatting at different levels (or repeated stand ups from a chair), lateral elevation of the legs in a standing position, and rising on the toes*. Participants were asked to imitate simple flexibility exercises demonstrated by the occupational therapist. Balance training consisted of *small step trial exercises using cones and hoops on the ground and one- or two-leg balance exercises on the ground or on foam-rubber ground sheets*.	
**Stevens and Killeen[204]**<br>**2006**<br>**3-arm RCT**	**Intensity:** N/A<br>**Duration:** 12-week<br>**Training load:** 30-min, 3 times/week<br>**Intervention proposal:** The program, accompanied with 'generation appropriate' *music*, is based on *joint and large muscle group* movement with an intention to create a gentle aerobic exertion. The exercise program was conducted in groups by the researchers on scheduled days and times within each of the participating aged care facilities.	1. Exercise slowed the rate of progression of the cognitive symptoms related to dementia<br>2. Exercise slowed and reversed disability in some of the ADL
**Brett et al.[205]**<br>**2021**<br>**3-arm RCT**	**Intensity:** N/A<br>**Duration:** 12-week<br>**Training load:**<br>Intervention Group (IG)[1]: 45-min, 1 time/week<br>Intervention Group (IG)[2]: 15-min, 3 times/week<br>**Intervention proposal:** The *tailed* exercise intervention used a variety of exercises to target *strength, balance, endurance, and flexibility*. Each intervention session had a maximum of *5* participants to help improve adherence and compliance. Participants were encouraged to complete the exercises at a moderate intensity, which was monitored by observation of slight breathlessness during exercise. Exercises consisted of a range of seated and standing exercises. Participants were encouraged to complete 1 to 2 sets of 10 repetitions of each exercise they were allocated. However, the specific exercise, number of repetitions, and sets completed were adapted to suit the needs and capacities of participants on an individual basis. Only simple equipment was used, including *hand weights, balls, cones, and static pedals*. This ensured the replicability and cost effectiveness of the study in the *nursing home* setting. The physical exercise intervention was implemented by the primary investigator, a *physiotherapist* with 5 years of specialist experience of working in aged care. The intervention was conducted in a *sitting room* where furniture could be moved to maximize space, and doors shut to reduce interruptions and noise from other areas. The same intervention was conducted in the morning and afternoon to *provide another opportunity*. Participants were able to choose which session they attended to accommodate preferences for the time of day. Both intervention groups completed the same type of exercises each week. All sessions involved a *warm-up and cool-down;* the time spent on these sections were altered dependent on the length of the class. Both groups completed strength, balance, and endurance exercises, for IG[1] they were all completed in the one class over 45 mins, and IG[2] completed a different component over the 3 days.	A positive impact on the physical performance
**Almeida et al.[206]**<br>**2021**	**Intensity:** N/A<br>**Duration:** 12-week	Promising results to improve the components of:

**2-arm RCT**	**Training load:** 60-min, 3 times/week in the first month, 2 times/week in the second month, and 1 time/week in the last month<br>**Intervention proposal:** The *LiFE4D* is a *home-based PA program* for people with dementia to promote engagement in PA and to reduce sedentary behavior during daily living activities. The participants were encouraged to *perform daily PA multiple times a day*. This intervention was adapted from LiFE with regard to duration and weekly frequency of sessions, was tailored to each participant, included activities focusing *not just on balance and muscle strength but also on flexibility and endurance*, included an *educational and psychosocial component*, and provided the opportunity for *carers* to get involved. LiFE4D including *face-to-face* sessions and *phone calls*. The participants received a phone call per week in the second and third months. Face-to-face sessions lasted approximately aimed to adapt the PA to everyday tasks, increase task frequency and/or intensity, monitor progress, clarify doubts, motivate energy expenditure, and manage expectations. Phone calls had a maximum duration of 15 mins and aimed to motivate and monitor progress, as well as clarify doubts. The tailoring process of PA to each participant aimed to improve *balance (e.g., reduce the base of support, step over objects)*; *flexibility (e.g., flexion, extension, abduction and adduction of the upper and lower limbs)*; *muscle strength (e.g., lift objects, bend the knees); and endurance (e.g., climbing stairs, gardening, walking) with different intensities (e.g., light to vigorous)*. The participants started with light activities and progressed to moderate and vigorous activities (when possible), and they reduced the base of support during balance activities. At the end of the first month, the participants received a *manual* with the activities that they could continue to perform on their own. This manual included a description of activities and how to perform them throughout the day (e.g., store toothpaste in the bathroom cabinet below your waist level, perform squats to reach the toothpaste, and try not to bend your back; while waiting for dinner, support your right hand on a stable surface and walk in a straight line, with the heel of one foot against the toes of the other foot). The role of the *carers* was to motivate the participants to be as physically active and as less sedentary as possible during their day, using the strategies that they trained with during the LiFE4D in their daily routines. However, it was up to the carer to become involved. This study was carried out by a team of *physiotherapists and gerontologists*; however, this intervention can be implemented by different professionals and/or even carers who have had simple training in PA. The professional who performed the intervention at home also performed the phone calls to facilitate responses of the participants.	1. Health-related physical fitness<br>2. Namely<br>3. Cardiorespiratory<br>4. Balance
**Verdelho et al.[207]**<br>**2024**<br>**2-arm RCT**	**Intensity:** 2-15/20 RPE<br>**Duration:** 24-week<br>**Training load:** 60-mins, 3 times/week<br>**Intervention proposal:** The supervised sessions consisted of a *combined aerobic (walking), resistance, balance, agility and coordinative exercises* and every session included a 10-min warm up plus 5 min active pause (balance, agility, and coordinative exercises) and a 5- to 7-min cool-down in the end. Sequence of the remaining schema of the sessions were as following: *In the first 2 months*, 15 min walking plus 5 min active pause (resistance exercises, 1 series of 12 repetitions, three callisthenic exercises) plus 15 min walking plus 5 min flexibility (1 series of 10 s in three different postures). The aimed intensity was 12/13 RPE.	Intervention improved physical function

	Between the second and fourth months, the duration of the walking period increased, as well as the intensity (13/14 RPE), with the following changes: 20 min walking plus 5 min active pause (resistance exercises, 1 series of 15 repetitions, three callisthenic exercises) plus 15 min walking plus 5 min flexibility (1 series of 10 s in three different postures). *During months 4 to 6*, the walking period again increased, as well as the intensity (RPE 14/15), as following: 25 min walking plus 5 min of aerobic functional exercises (1 series of 15 repetitions, three callisthenic dynamic exercises) plus 20 min walking plus 5 min flexibility (1 series of 10 s in three different postures). *For the non-supervised sessions*, participants were asked to accumulate in that day at least 3 bouts of 10 min walking, and RPE according to their training intensity phase.	
**Yan et al.[208]** **2024** **2-arm RCT**	**Intensity:** N/A<br>**Duration:** 12-week<br>**Training load:** 60-min, 3 times/week<br>**Intervention proposal:** This included centralized *teaching sessions* delivered twice (once offline and once online) by standardized *trained nurses* responsible for providing exercise to older people with PD-MCI, as well as one session of self-practice. Each participant was provided with a *printed exercise guide manual* and access to *video resources* for easy self-practice. Moreover, the WeChat applet included an *exercise score ranking system* to motivate training enthusiasm. Participants in the intervention group received multi-component exercise training developed by a multidisciplinary team using the *Delphi method*, comprising *resistance training, balance training, and gait training*. Each session included *warm-up, multi-component exercise training, and relaxation.* The 12-week intervention was divided into three stages: *initial stage (weeks 1 4), middle stage (weeks 5 8), and late stage (weeks 9 12)*, following the principle of progression from easy to difficult to adapt to participants' capabilities and exercise perseverance. To ensure safety during exercise training for older people with PD-MCI, electronic BP monitors were used before and after exercise to monitor BP and HR. Additionally, participants self-assessed their fatigue level using the RPE after each exercise session.	Improvements in cognitive function, physical motor function, and social and psychological health
**David et al.[209]** **2025** **2-arm RCT**	**Intensity:** N/A<br>**Duration:** 24-week<br>**Training load:** 105-min, 1 time/week<br>**Intervention proposal:** The program was homogenized between both sites and included *aerobic, resistance, and coordination exercises*. It was supervised by *physiotherapists* and members of the study team. Sports groups usually consisted of about *five* participants per group, therefore assuring an optimized supervision and individualized guidance of the sports program. During the first supervised training session, the optimal exercise load for the program was determined for each individual. Over the course of the intervention period, the exercise load was then gradually increased according to moderate-to-vigorous physical exertion regularly surveyed using the RPE-scale during the training sessions. In addition to the participation in the supervised training program, participants of the intervention group were further instructed to perform a moderate-to-vigorous intensity *home-based training* for at least 30 min per week, as well as stretching and toning exercises for at least 15 min twice per week,	Improved cardiorespiratory fitness

	following current recommendations on repetitive physical exercise. *Supporting training cards* from the German Federal Centre for Health Education (BZgA) for the stretching and toning exercises were distributed to the intervention participants. To optimize coordination training at home, the participants received an MFT Fun Disc® balance plate, which was also used during the training sessions at both study sites.	
**Shokri et al.[209]**<br>**2024**<br>**3-arm RCT (two arms were included)**	**Intensity:** 4–6/10 RPE<br>**Duration:** 12-week<br>**Training load:** 35–45-min, 3 times/week<br>**Intervention proposal:** Each session including *10 min of warm-up with dynamic movements, 20–35 min of main exercises, and 5 minutes of cool down* with stretching movements. Because of the Coronavirus pandemic, all workouts were conducted online via *WhatsApp video* call individually with the trainer (*researcher*). The *presence of a caregiver* in the training session was mandatory. The training group performed a combination of *sitting and standing* movements. The movements involved the whole body and large muscle groups. The break between sets started at 70 s, and gradually it reduced 10 s every two weeks until the tenth week eventually reduced by 5 s to reach 25 s. The number of sets began with two sets and progressively increased to one set every month, 5–10 repetitions per set depending on the difficulty of the movement and the patient's fitness. We used the examiner-choose music playlist based on expert cognitive neurologist opinion, including some sort of Mozart music Symphonia, and also traditional Iranian music by the patient's selection. The session started with Mozart's sonatas for 10–15 min, continued with Persian music, and then during the cool down, Mozart's sonatas were played. Music was played throughout the exercise session.	Improved cognitive and physical performance

Abbreviation:

ACSM: American College of Sports Medicine
PA: Physical Activity
HR: Heart Rate
RPE: Borg's Rating of Perceived Exertion
RM: Repetition Maximum
HRR: Heart rate reserve
DMT: Dance movement therapy
VO_{2peak}: Peak oxygen uptake
HR_{max}: Maximal heart rate
HRR: Heart Rate Reserve
RM: Repetitions Maximum
MMSE: Mini-Mental State Examination
METs: Maximal exercise tests
VO_{2max}: Maximal oxygen consumption
SBP: Systolic BP
DBP: Diastolic BP
ME: Metabolic equivalent
MTP: Maximal Tolerated Power

IMET: Incremental maximal exercise tests
"Talk" test: Ability to speak a sentence without catching breath multiple times
6-MWT: 6-minute walk test
MVC: Maximum voluntary contraction
MCI: Mild cognitive impairment
ADL: Activity of Daily Living
AD: Alzheimer's disease
SPPB: The Short Physical Performance Battery
SF-12: The SF-12 quality-of-life questionnaire
BDNF: The Brain-Derived Neurotrophic Factor level
QoL: Quality of Life
BP: Blood Pressure

References

1. Kropacova S, Mitterova K, Klobusiakova P, et al. Cognitive effects of dance-movement intervention in a mixed group of seniors are not dependent on hippocampal atrophy. *J Neural Transm*. 2019;126(11):1455-1463. doi:10.1007/s00702-019-02068-y

2. Esmail A, Vrinceanu T, Lussier M, et al. Effects of Dance/Movement Training vs. Aerobic Exercise Training on cognition, physical fitness and quality of life in older adults: A randomized controlled trial. *J Bodyw Mov Ther*. 2020;24(1):212-220. doi:10.1016/j.jbmt.2019.05.004

3. Bisbe M, Fuente-Vidal A, López E, et al. Comparative Cognitive Effects of Choreographed Exercise and Multimodal Physical Therapy in Older Adults with Amnestic Mild Cognitive Impairment: Randomized Clinical Trial. *Journal of Alzheimer's Disease*. 2020;73(2):769-783. doi:10.3233/JAD-190552

4. Qi M, Zhu Y, Zhang L, Wu T, Wang J. The effect of aerobic dance intervention on brain spontaneous activity in older adults with mild cognitive impairment: A resting-state functional MRI study. *Exp Ther Med*. Published online 2018:715-722. doi:10.3892/etm.2018.7006

5. Lazarou I, Parastatidis T, Tsolaki A, et al. International Ballroom Dancing Against Neurodegeneration: A Randomized Controlled Trial in Greek Community-Dwelling Elders With Mild Cognitive impairment. *Am J Alzheimers Dis Other Demen*. 2017;32(8):489-499. doi:10.1177/1533317517725813

6. Franco MR, Sherrington C, Tiedemann A, et al. Effect of Senior Dance (DanSE) on Fall Risk Factors in Older Adults: A Randomized Controlled Trial. *Phys Ther*. 2020;100(4):600-608. doi:10.1093/ptj/pzz187

7. Doi T, Verghese J, Makizako H, et al. Effects of Cognitive Leisure Activity on Cognition in Mild Cognitive Impairment: Results of a Randomized Controlled Trial. *J Am Med Dir Assoc*. 2017;18(8):686-691. doi:10.1016/j.jamda.2017.02.013

8. Blumen HM, Ayers E, Wang C, Ambrose AF, Jayakody O, Verghese J. Randomized Controlled Trial of Social Ballroom Dancing and Treadmill Walking: Preliminary Findings on Executive Function and Neuroplasticity From Dementia-at-Risk Older Adults. *J Aging Phys Act*. 2023;31(4):589-599. doi:10.1123/japa.2022-0176

9. Zhu Y, Wu H, Qi M, et al. Effects of a specially designed aerobic dance routine on mild cognitive impairment. *Clin Interv Aging*. 2018;13:1691-1700. doi:10.2147/CIA.S163067

10. Song D, Yu D, Liu T, Wang J. Effect of an Aerobic Dancing Program on Sleep Quality for Older Adults With Mild Cognitive Impairment and Poor Sleep: A Randomized Controlled Trial. *J Am Med Dir Assoc*. 2024;25(3):494-499. doi:10.1016/j.jamda.2023.09.020

11. Chang J, Zhu W, Zhang J, et al. The Effect of Chinese Square Dance Exercise on Cognitive Function in Older Women With Mild Cognitive Impairment: The Mediating Effect of Mood Status and Quality of Life. *Front Psychiatry*. 2021;12(July). doi:10.3389/fpsyt.2021.711079

12. Chang J, Chen Y, Liu C, et al. Effect of Square Dance Exercise on Older Women With Mild Mental Disorders. *Front Psychiatry*. 2021;12(July):1-9. doi:10.3389/fpsyt.2021.699778

13. Van de Winckel A, Feys H, De Weerdt W, Dom R. Cognitive and behavioural effects of music-based exercises in patients with dementia. *Clin Rehabil*. 2004;18(3):253-260. doi:10.1191/0269215504cr750oa

14. Bracco L, Pinto-Carral A, Hillaert L, Mourey F. Tango-therapy vs physical exercise in older people with dementia; a randomized controlled trial. *BMC Geriatr*. 2023;23(1):1-13. doi:10.1186/s12877-023-04342-x

15. Zhu Y, Gao Y, Guo C, et al. Effect of 3-Month Aerobic Dance on Hippocampal Volume and Cognition in Elderly People With Amnestic Mild Cognitive Impairment: A Randomized Controlled Trial. *Front Aging Neurosci*. 2022;14(March):1-10. doi:10.3389/fnagi.2022.771413

16. Ho RTH, Fong TCT, Chan WC, et al. Psychophysiological Effects of Dance Movement Therapy and Physical Exercise on Older Adults with Mild Dementia: A Randomized Controlled Trial. *Journals of Gerontology - Series B Psychological Sciences and Social Sciences*. 2018;75(3):560-570. doi:10.1093/geronb/gby145

17. Thiel U, Stiebler M, Labott BK, et al. DiADEM—Dance against Dementia—Effect of a Six-Month Dance Intervention on Physical Fitness in Older Adults with Mild Cognitive Impairment: A Randomized, Controlled Trial. *J Pers Med*. 2024;14(8). doi:10.3390/jpm14080888

18. Sánchez-Alcalá M, Aibar-Almazán A, Hita-Contreras F, et al. Effects of Dance-Based Aerobic Training on Mental Health and Quality of Life in Older Adults with Mild Cognitive Impairment. *J Pers Med*. 2024;14(8):1-16. doi:10.3390/jpm14080844

19. Sánchez-Alcalá M, Aibar-Almazán A, Carcelén-Fraile M del C, et al. Effects of Dance-Based Aerobic Training on Frailty and Cognitive Function in Older Adults with Mild Cognitive Impairment: A Randomized Controlled Trial. *Diagnostics*. 2025;15(3):1-13. doi:10.3390/diagnostics15030351

20. Eggenberger P, Schumacher V, Angst M, Theill N, de Bruin ED. Does multicomponent physical exercise with simultaneous cognitive training boost cognitive performance in older adults? A 6-month randomized controlled trial with a 1-year follow-up. *Clin Interv Aging*. 2015;10:1335-1349. doi:10.2147/CIA.S87732

21. Eggenberger P, Theill N, Holenstein S, Schumacher V, de Bruin ED. Multicomponent physical exercise with simultaneous cognitive training to enhance dual-task walking of older adults: A secondary analysis of a 6-month randomized controlled trial with I-year follow-up. *Clin Interv Aging*. 2015;10:1711-1732. doi:10.2147/CIA.S91997

22. Hughes TF, Flatt JD, Fu B, Butters MA, Chang CCH, Ganguli M. Interactive video gaming compared with health education in older adults with mild cognitive impairment: A feasibility study. *Int J Geriatr Psychiatry*. 2014;29(9):890-898. doi:10.1002/gps.4075

23. Sabbagh et al. Sensor-based balance training with motion feedback in people with mild cognitive impairment. 2016;53(6):945-958. doi:10.1682/JRRD.2015.05.0089.Sensor-based

24. Liu CL, Cheng FY, Wei MJ, Liao YY. Effects of Exergaming-Based Tai Chi on Cognitive Function and Dual-Task Gait Performance in Older Adults With Mild Cognitive Impairment: A Randomized Control Trial. *Front Aging Neurosci*. 2022;14(March). doi:10.3389/fnagi.2022.761053

25. Padala KP, Padala PR, Malloy TR, et al. Wii-fit for improving gait and balance in an assisted living facility: A pilot study. *J Aging Res*. 2012;2012:6-11. doi:10.1155/2012/597573

26. Liao YY, Chen IH, Hsu WC, Tseng HY, Wang RY. Effect of exergaming versus combined exercise on cognitive function and brain activation in frail older adults: A randomised controlled trial. *Ann Phys Rehabil Med*. 2021;64(5):101492. doi:10.1016/j.rehab.2021.101492

27. Karssemeijer EGA, Aaronson JA, Bossers WJR, Donders R, Olde Rikkert MGM, Kessels RPC. The quest for synergy between physical exercise and cognitive stimulation via exergaming in people with dementia: A randomized controlled trial. *Alzheimers Res Ther*. 2019;11(1):1-13. doi:10.1186/s13195-018-0454-z

28. Karssemeijer EGA, Bossers WJR, Aaronson JA, Sanders LMJ, Kessels RPC, Olde Rikkert MGM. Exergaming as a Physical Exercise Strategy Reduces Frailty in People With Dementia: A Randomized Controlled Trial. *J Am Med Dir Assoc*. 2019;20(12):1502-1508.e1. doi:10.1016/j.jamda.2019.06.026

29. van Santen J, Dröes RM, Twisk JWR, Blanson Henkemans OA, van Straten A, Meiland FJM. Effects of Exergaming on Cognitive and Social Functioning of People with Dementia: A Randomized Controlled Trial. *J Am Med Dir Assoc*. 2020;21(12):1958-1967.e5. doi:10.1016/j.jamda.2020.04.018

30. Wu S, Ji H, Won J, Jo EA, Kim YS, Park JJ. The Effects of Exergaming on Executive and Physical Functions in Older Adults With Dementia: Randomized Controlled Trial. *J Med Internet Res*. 2023;25:1-17. doi:10.2196/39993

31. Swinnen N, Vandenbulcke M, de Bruin ED, et al. The efficacy of exergaming in people with major neurocognitive disorder residing in long-term care facilities: a pilot randomized controlled trial. *Alzheimers Res Ther*. 2021;13(1):1-13. doi:10.1186/s13195-021-00806-7

32. Zheng J, Yu P, Chen X. An Evaluation of the Effects of Active Game Play on Cognition, Quality of Life and Depression for Older People with Dementia. *Clin Gerontol*. 2022;45(4):1034-1043. doi:10.1080/07317115.2021.1980170

33. Uğur F, Sertel M. Wii Fit Exercise's Effects on Muscle Strength and Fear of Falling in Older Adults With Alzheimer Disease: A Randomized Controlled Trial. *J Aging Phys Act*. 2025;33(2):181-191. doi:10.1123/japa.2023-0428

34. Grzenda A, Siddarth P, Milillo MM, Aguilar-Faustino Y, Khalsa DS, Lavretsky H. Cognitive and immunological effects of yoga compared to memory training in older women at risk for alzheimer's disease. *Transl Psychiatry*. 2024;14(1):1-11. doi:10.1038/s41398-024-02807-0

35. Khanthong P, Sriyakul K, Dechakhamphu A, Krajarng A, Kamalashiran C, Tungsukruthai P. Traditional Thai exercise (Ruesi Dadton) for improving motor and cognitive functions in mild cognitive impairment: a randomized controlled trial. *J Exerc Rehabil*. 2021;17(5):331-338. doi:10.12965/JER.2142542.271

36. Kashyap M, Rai NK, Singh R, et al. Effect of Early Yoga Practice on Post Stroke Cognitive Impairment. 2022;22(4):2019. doi:10.4103/aian.AIAN

37. Tremont G, Davis J, Ott BR, et al. Feasibility of a Yoga Intervention for Individuals with Mild Cognitive Impairment: A Randomized Controlled Trial. *Journal of Integrative and Complementary Medicine*. 2022;28(3):250-260. doi:10.1089/jicm.2021.0204

38. Eyre HA, Siddarth P, Acevedo B, et al. A randomized controlled trial of Kundalini yoga in mild cognitive impairment. *Int Psychogeriatr*. 2017;29(4):557-567. doi:10.1017/S1041610216002155

39. Eyre HA, Acevedo B, Yang H, et al. Changes in Neural Connectivity and Memory Following a Yoga Intervention for Older Adults: A Pilot Study. *Journal of Alzheimer's Disease*. 2016;52(2):673-684. doi:10.3233/JAD-150653

40. Li F, Harmer P, Fitzgerald K, Winters-Stone K. A cognitively enhanced online Tai Ji Quan training intervention for community-dwelling older adults with mild cognitive impairment: A feasibility trial. *BMC Geriatr*. 2022;22(1):1-13. doi:10.1186/s12877-021-02747-0

41. Sungkarat S, Boripuntakul S, Kumfu S, Lord SR, Chattipakorn N. Tai Chi Improves Cognition and Plasma BDNF in Older Adults With Mild Cognitive Impairment: A Randomized Controlled Trial. *Neurorehabil Neural Repair*. 2018;32(2):142-149. doi:10.1177/1545968317753682

42. Sungkarat S, Boripuntakul S, Chattipakorn N, Watcharasaksilp K, Lord SR. Effects of Tai Chi on Cognition and Fall Risk in Older Adults with Mild Cognitive Impairment: A Randomized Controlled Trial. *J Am Geriatr Soc*. 2017;65(4):721-727. doi:10.1111/jgs.14594

43. Li F, Harmer P, Voit J, Chou LS. Implementing an online virtual falls prevention intervention during a public health pandemic for older adults with mild cognitive impairment: A feasibility trial. *Clin Interv Aging*. 2021;16:973-983. doi:10.2147/CIA.S306431

44. Lam LCW, Chau RCM, Wong BML, et al. A 1-Year Randomized Controlled Trial Comparing Mind Body Exercise (Tai Chi) With Stretching and Toning Exercise on Cognitive Function in Older Chinese Adults at Risk of Cognitive Decline. *J Am Med Dir Assoc*. 2012;13(6):568.e15-568.e20. doi:10.1016/j.jamda.2012.03.008

45. Lam LCW, Chau RCM, Wong BML, et al. Interim follow-up of a randomized controlled trial comparing Chinese style mind body (Tai Chi) and stretching exercises on cognitive function in subjects at risk of progressive cognitive decline. *Int J Geriatr Psychiatry*. 2011;26(7):733-740. doi:10.1002/gps.2602

46. Jiayuan Z, Xiang-Zi J, Li-Na M, Jin-Wei Y, Xue Y. Effects of Mindfulness-Based Tai Chi Chuan on Physical Performance and Cognitive Function among Cognitive Frailty Older Adults: A Six-Month Follow-Up of a Randomized Controlled Trial. *Journal of Prevention of Alzheimer's Disease*. 2022;9(1):104-112. doi:10.14283/jpad.2021.40

47. Chen Y, Qin J, Tao L, et al. Effects of Tai Chi Chuan on Cognitive Function in Adults 60 Years or Older With Type 2 Diabetes and Mild Cognitive Impairment in China: A Randomized Clinical Trial. *JAMA Netw Open*. 2023;6(4):E237004. doi:10.1001/jamanetworkopen.2023.7004

48. Lin M, Liu W, Ma C, et al. Tai Chi-Induced Exosomal LRP1 is Associated With Memory Function and Hippocampus Plasticity in aMCI Patients. *American Journal of Geriatric Psychiatry*. 2024;32(10):1215-1230. doi:10.1016/j.jagp.2024.04.012

49. Yu AP, Chin EC, Yu DJ, et al. Tai Chi versus conventional exercise for improving cognitive function in older adults: a pilot randomized controlled trial. *Sci Rep*. 2022;12(1):1-15. doi:10.1038/s41598-022-12526-5

50. Huang N, Li W, Rong X, et al. Effects of a Modified Tai Chi Program on Older People with Mild Dementia: A Randomized Controlled Trial. *Journal of Alzheimer's Disease*. 2019;72(3):947-956. doi:10.3233/JAD-190487

51. Cheng ST, Chow PK, Song YQ, et al. Mental and physical activities delay cognitive decline in older persons with dementia. *American Journal of Geriatric Psychiatry*. 2014;22(1):63-74. doi:10.1016/j.jagp.2013.01.060

52. Cheng ST, Chow PK, Yu ECS, Chan ACM. Leisure activities alleviate depressive symptoms in nursing home residents with very mild or mild dementia. *American Journal of Geriatric Psychiatry*. 2012;20(10):904-908. doi:10.1097/JGP.0b013e3182423988

53. Liu JYW, Kwan RYC, Lai CKY, Hill KD. A simplified 10-step Tai-chi programme to enable people with dementia to improve their motor performance: a feasibility study. *Clin Rehabil*. 2018;32(12):1609-1623. doi:10.1177/0269215518786530

54. Chan AWK, Yu DSF, Choi KC, Lee DTF, Sit JWH, Chan HYL. Tai chi qigong as a means to improve night-time sleep quality among older adults with cognitive impairment: A pilot randomized controlled trial. *Clin Interv Aging*. 2016;11:1277-1286. doi:10.2147/CIA.S111927

55. Nyman SR, Ingram W, Sanders J, et al. Randomised controlled trial of the effect of tai chi on postural balance of people with dementia. *Clin Interv Aging*. 2019;14:2017-2029. doi:10.2147/CIA.S228931

56. Williams J, Nyman S. A secondary analysis of a randomised controlled trial to investigate the effect of Tai Chi on the instrumented timed up and go test in people with mild to moderate dementia. *Aging Clin Exp Res*. 2021;33(8):2175-2181. doi:10.1007/s40520-020-01741-7

57. Canan Okuyan ED. The effectiveness of Tai Chi Chuan on fear of movement, prevention of falls, physical activity, and cognitive status in older adults with mild cognitive impairment: A randomized controlled trial. *Perspect Psychiatr Care*. 2021;57(3):1273-1281. doi:10.1111/ppc.12684

58. Su H, Wang H, Meng L, Bush E. The effects of Baduanjin exercise on the subjective memory complaint of older adults: A randomized controlled trial. *Medicine (United States)*. 2021;100(30):E25442. doi:10.1097/MD.0000000000025442

59. Zheng G, Zheng Y, Xiong Z, Ye B. Effect of Baduanjin exercise on cognitive function in patients with post-stroke cognitive impairment: a randomized controlled trial. *Clin Rehabil*. 2020;34(8):1028-1039. doi:10.1177/0269215520930256

60. Xia R, Wan M, Lin H, Ye Y, Chen S, Zheng G. Effects of mind–body exercise Baduanjin on cognition in community-dwelling older people with mild cognitive impairment: A randomized controlled trial. *Neuropsychol Rehabil*. 2023;33(8):1368-1383. doi:10.1080/09602011.2022.2099909

61. Zheng G, Ye B, Xia R, et al. Traditional Chinese Mind-Body Exercise Baduanjin Modulate Gray Matter and Cognitive Function in Older Adults with Mild Cognitive Impairment: A Brain Imaging Study. *Brain Plasticity*. 2021;7(2):131-142. doi:10.3233/bpl-210121

62. Xia R, Qiu P, Lin H, et al. The effect of traditional chinese mind-body exercise (Baduanjin) and brisk walking on the dorsal attention network in older adults with mild cognitive impairment. *Front Psychol*. 2019;10(SEP):1-9. doi:10.3389/fpsyg.2019.02075

63. Li K, Yu H, Kortas JA, Lin X, Lipowski M. The effect of 12 weeks of Baduanjin exercise on cognitive function, lower limb balance and quality of life of the elderly with mild cognitive impairment: a randomized controlled trial. *Gazzetta Medica Italiana Archivio per le Scienze Mediche*. 2022;181(11):811-823. doi:10.23736/S0393-3660.22.04802-1

64. Luo SS, Chen L, Wang GB, Wang YG, Su XY. Effects of long-term Wuqinxi exercise on working memory in older adults with mild cognitive impairment. *Eur Geriatr Med*. 2022;13(6):1327-1333. doi:10.1007/s41999-022-00709-2

65. Chang CL, Lin TK, Pan CY, et al. Distinct effects of long-term Tai Chi Chuan and aerobic exercise interventions on motor and neurocognitive performance in early-stage Parkinson's disease: a randomized controlled trial. *Eur J Phys Rehabil Med*. 2024;60(4):621-633. doi:10.23736/S1973-9087.24.08166-8

66. Gao R, Greiner C, Ryuno H, Zhang X. Effects of Tai Chi on physical performance, sleep, and quality of life in older adults with mild to moderate cognitive impairment. *BMC Complement Med Ther*. 2024;24(1). doi:10.1186/s12906-024-04705-w

67. Hsu CY, Yeh ML, Liu YCE. Three-month Chan-Chuang qigong program improves physical performance and quality of life of patients with cognitive impairment: A randomized controlled trial. *Res Nurs Health*. 2022;45(3):327-336. doi:10.1002/nur.22219

68. Tomoto T, Liu J, Tseng BY, et al. One-Year Aerobic Exercise Reduced Carotid Arterial Stiffness and Increased Cerebral Blood Flow in Amnestic Mild Cognitive Impairment. *Journal of Alzheimer's Disease*. 2021;80(2):841-853. doi:10.3233/JAD-201456

69. Shimada H, Lee S, Akishita M, et al. Effects of golf training on cognition in older adults: A randomised controlled trial. *J Epidemiol Community Health (1978)*. 2018;72(10):944-950. doi:10.1136/jech-2017-210052

70. Donnezan et al. Effects of simultaneous aerobic and cognitive training on executive functions, cardiovascular fitness and functional abilities in older adults with mild cognitive impairment. *Ment Health Phys Act*. 2018;15(April):78-87. doi:10.1016/j.mhpa.2018.06.001

71. Baker LD, Frank LL, Foster-Schubert K, et al. Effects of aerobic exercise on mild cognitive impairment: A controlled trial. *Arch Neurol*. 2010;67(1):71-79. doi:10.1001/archneurol.2009.307

72. Ihle-Hansen H, Langhammer B, Lydersen S, Gunnes M, Indredavik B, Askim T. A physical activity intervention to prevent cognitive decline after stroke: Secondary results from the life after stroke study, an 18-month randomized controlled trial. *J Rehabil Med*. 2019;51(9):646-651. doi:10.2340/16501977-2588

73. Askim T, Langhammer B, Ihle-Hansen H, et al. Efficacy and safety of individualized coaching after stroke: The LAST study (life after stroke) a pragmatic randomized controlled trial. *Stroke*. 2018;49(2):426-432. doi:10.1161/STROKEAHA.117.018827

74. Nagamatsu LS, Chan A, Davis JC, et al. Physical activity improves verbal and spatial memory in older adults with probable mild cognitive impairment: A 6-month randomized controlled trial. *J Aging Res*. 2013;2013(Mci). doi:10.1155/2013/861893

75. Ten Brinke LF, Bolandzadeh N, Nagamatsu LS, et al. Aerobic exercise increases hippocampal volume in older women with probable mild cognitive impairment: A 6-month randomised controlled trial. *Br J Sports Med*. 2015;49(4):248-254. doi:10.1136/bjsports-2013-093184

76. Tsai CL, Pai MC, Ukropec J, Ukropcová B. Distinctive Effects of Aerobic and Resistance Exercise Modes on Neurocognitive and Biochemical Changes in Individuals with Mild Cognitive Impairment. *Curr Alzheimer Res*. 2019;16(4):316-332. doi:10.2174/1567205016666190228125429

77. Hsu CL, Best JR, Davis JC, et al. Aerobic exercise promotes executive functions and impacts functional neural activity among older adults with vascular cognitive impairment. *Br J Sports Med*. 2018;52(3):184-191. doi:10.1136/bjsports-2016-096846

78. ten Brinke LF, Hsu CL, Best JR, Barha CK, Liu-Ambrose T. Increased Aerobic Fitness Is Associated with Cortical Thickness in Older Adults with Mild Vascular Cognitive Impairment. *Journal of Cognitive Enhancement*. 2018;2(2):157-169. doi:10.1007/s41465-018-0077-0

79. Hsu CL, Best JR, Wang S, et al. The impact of aerobic exercise on fronto-parietal network connectivity and its relation to mobility: An exploratory analysis of a 6-month randomized controlled trial. *Front Hum Neurosci*. 2017;11(June):1-12. doi:10.3389/fnhum.2017.00344

80. Dao E, Best JR, Hsiung GYR, et al. Associations between cerebral amyloid and changes in cognitive function and falls risk in subcortical ischemic vascular cognitive impairment. *BMC Geriatr*. 2017;17(1):1-9. doi:10.1186/s12877-017-0522-4

81. Liu-Ambrose T, Best JR, Davis JC, et al. Aerobic exercise and vascular cognitive impairment. *Neurology*. 2016;87(20):2082-2090. doi:10.1212/WNL.0000000000003332

82. Song D, Yu DSF. Effects of a moderate-intensity aerobic exercise programme on the cognitive function and quality of life of community-dwelling elderly people with mild cognitive impairment: A randomised controlled trial. *Int J Nurs Stud*. 2019;93:97-105. doi:10.1016/j.ijnurstu.2019.02.019

83. Stuckenschneider T, Sanders ML, Devenney KE, et al. NeuroExercise: The Effect of a 12-Month Exercise Intervention on Cognition in Mild Cognitive Impairment—A Multicenter Randomized Controlled Trial. *Front Aging Neurosci*. 2021;12(January):1-12. doi:10.3389/fnagi.2020.621947

84. Rojasavastera R, Bovonsunthonchai S, Hiengkaew V, Senanarong V. Action observation combined with gait training to improve gait and cognition in elderly with mild cognitive impairment a randomized controlled trial. *Dementia e Neuropsychologia*. 2020;14(2):118-127. doi:10.1590/1980-57642020dn14-020004

85. Makino T, Umegaki H, Ando M, et al. Effects of Aerobic, Resistance, or Combined Exercise Training among Older Adults with Subjective Memory Complaints: A Randomized Controlled Trial. *Journal of Alzheimer's Disease*. 2021;82(2):701-717. doi:10.3233/JAD-210047

86. Brydges CR, Liu-Ambrose T, Bielak AAM. Using intraindividual variability as an indicator of cognitive improvement in a physical exercise intervention of older women with mild cognitive impairment. *Neuropsychology*. 2020;34(8):825-834. doi:10.1037/neu0000638

87. Morris JK, Vidoni ED, Johnson DK, et al. Aerobic exercise for Alzheimer's disease: A randomized controlled pilot trial. *PLoS One*. 2017;12(2):1-14. doi:10.1371/journal.pone.0170547

88. Wei X hong, Ji L li. Effect of handball training on cognitive ability in elderly with mild cognitive impairment. *Neurosci Lett*. 2014;566:98-101. doi:10.1016/j.neulet.2014.02.035

89. Damirchi A, Hosseini F, Babaei P. Mental Training Enhances Cognitive Function and BDNF More Than Either Physical or Combined Training in Elderly Women With MCI: A Small-Scale Study. *Am J Alzheimers Dis Other Demen*. 2018;33(1):20-29. doi:10.1177/1533317517727068

90. Nakatsuka M, Nakamura K, Hamanosono R, et al. A Cluster Randomized Controlled Trial of Nonpharmacological Interventions for Old-Old Subjects with a Clinical Dementia Rating of 0.5: The Kurihara Project. *Dement Geriatr Cogn Dis Extra*. 2015;5(2):221-232. doi:10.1159/000380816

91. Kohanpour MA, Peeri M, Azarbayjani MA. The effects of aerobic exercise with lavender essence use on cognitive state and serum brain-derived neurotrophic factor levels in elderly with mild cognitive impairment. *Journal of HerbMed Pharmacology*. 2017;6(2):80-84.

92. Karthikeyan T. Therapeutic effects of home-based exercise of geriatrics for the management of cognitive impairment. *ES J Public Health*. 2020;1(1):1003. www.escientificlibrary.com

93. Krootnark K, Chaikeeree N, Saengsirisuwan V, Boonsinsukh R. Effects of low-intensity home-based exercise on cognition in older persons with mild cognitive impairment: a direct comparison of aerobic versus resistance exercises using a randomized controlled trial design. *Front Med (Lausanne)*. 2024;11(June):1-11. doi:10.3389/fmed.2024.1392429

94. Liu IT, Lee WJ, Lin SY, Chang ST, Kao CL, Cheng YY. Therapeutic Effects of Exercise Training on Elderly Patients With Dementia: A Randomized Controlled Trial. *Arch Phys Med Rehabil*. 2020;101(5):762-769. doi:10.1016/j.apmr.2020.01.012

95. Dillon K, Prapavessis H. REducing SEDENTary behavior among mild to moderate cognitively impaired assisted living residents: A pilot randomized controlled trial (RESEDENT study). *J Aging Phys Act*. 2021;29(1):27-35. doi:10.1123/JAPA.2019-0440

96. Yang SY, Shan CL, Qing H, et al. The Effects of Aerobic Exercise on Cognitive Function of Alzheimer's Disease Patients. *CNS Neurol Disord Drug Targets*. 2015;14(10):1292-1297. doi:10.2174/1871527315666151111123319

97. Choi W, Lee S. Ground kayak paddling exercise improves postural balance, muscle performance, and cognitive function in older adults with mild cognitive impairment: A randomized controlled trial. *Medical Science Monitor*. 2018;24:3909-3915. doi:10.12659/MSM.908248

98. Choi W, Lee S. The effects of virtual kayak paddling exercise on postural balance, muscle performance, and cognitive function in older adults with mild cognitive impairment: A randomized controlled trial. *J Aging Phys Act*. 2019;27(6):861-870. doi:10.1123/japa.2018-0020

99. Yu DJ, Yu AP, Bernal JDK, et al. Effects of exercise intensity and frequency on improving cognitive performance in middle-aged and older adults with mild cognitive impairment: A pilot randomized controlled trial on the minimum physical activity recommendation from WHO. *Front Physiol*. 2022;13(September):1-12. doi:10.3389/fphys.2022.1021428

100. Fischbacher M, Chocano-Bedoya PO, Meyer U, et al. Safety and feasibility of a Dalcroze eurhythmics and a simple home exercise program among older adults with mild cognitive impairment (MCI) or mild dementia: The MOVE for your MIND pilot trial. *Pilot Feasibility Stud*. 2020;6(1):1-8. doi:10.1186/s40814-020-00645-7

101. Khattak HG, Ahmad Z, Arshad H, Anwar K. Effect of aerobic exercise on cognition in elderly persons with mild cognitive impairment. *Rawal Medical Journal*. 2022;47(3):698-701. doi:10.5455/rmj.20210713072242

102. Varela S, Ayán C, Cancela JM, Martín V. Effects of two different intensities of aerobic exercise on elderly people with mild cognitive impairment: A randomized pilot study. *Clin Rehabil*. 2012;26(5):442-450. doi:10.1177/0269215511425835

103. Miu D, Edin F, Szeto S, Mak Y. A randomised controlled trial on the effect of exercise on physical, cognitive and affective function in dementia subjects. *Asian Journal of Gerontology & Geriatrics*. 2008;3(1):8-16.

104. Arcoverde C, Deslandes A, Moraes H, et al. Treadmill training as an augmentation treatment for Alzheimer's disease: A pilot randomized controlled study. *Arq Neuropsiquiatr*. 2014;72(3):190-196. doi:10.1590/0004-282X20130231

105. Angiolillo A, Leccese D, Ciccotelli S, et al. Effects of Nordic walking in Alzheimer's disease: A single-blind randomized controlled clinical trial. *Heliyon*. 2023;9(5):e15865. doi:10.1016/j.heliyon.2023.e15865

106. Enette L, Vogel T, Merle S, et al. Effect of 9 weeks continuous vs. interval aerobic training on plasma BDNF levels, aerobic fitness, cognitive capacity and quality of life among seniors with mild to moderate Alzheimer's disease: A randomized controlled trial. *European Review of Aging and Physical Activity*. 2020;17(1):1-16. doi:10.1186/s11556-019-0234-1

107. Phoemsapthawee et al. The Benefit of Arm Swing Exercise on Cognitive Performance in Older Women with Mild Cognitive Impairment. *Journal of Exercise Physiology*. 2016;8(1):11-25.

108. Eggermont LHP, Swaab DF, Hol EM, Scherder EJA. Walking the line: A randomised trial on the effects of a short term walking programme on cognition in dementia. *J Neurol Neurosurg Psychiatry*. 2009;80(7):802-804. doi:10.1136/jnnp.2008.158444

109. Lowery D, Cerga-Pashoja A, Iliffe S, et al. The effect of exercise on behavioural and psychological symptoms of dementia: The EVIDEM-E randomised controlled clinical trial. *Int J Geriatr Psychiatry*. 2014;29(8):819-827. doi:10.1002/gps.4062

110. Guzel I, Can F. The effects of different exercise types on cognitive and physical functions in dementia patients: A randomized comparative study. *Arch Gerontol Geriatr*. 2024;119(18):105321. doi:10.1016/j.archger.2023.105321

111. Venturelli M, Scarsini R, Schena F. Six-month walking program changes cognitive and ADL performance in patients with Alzheimer. *Am J Alzheimers Dis Other Demen*. 2011;26(5):381-388. doi:10.1177/1533317511418956

112. Scherder EJA, Van Paasschen J, Deijen JB, et al. Physical activity and executive functions in the elderly with mild cognitive impairment. *Aging Ment Health*. 2005;9(3):272-280. doi:10.1080/13607860500089930

113. Amjad I, Toor H, Niazi IK, et al. Therapeutic effects of aerobic exercise on EEG parameters and higher cognitive functions in mild cognitive impairment patients. *International Journal of Neuroscience*. 2019;129(6):551-562. doi:10.1080/00207454.2018.1551894

114. Abbas RL, Saab IM, Al-Sharif HK, Naja N, El-Khatib A. Effect of Adding Motorized Cycle Ergometer Over Exercise Training on Balance in Older Adults with Dementia: A Randomized Controlled Trial. *Exp Aging Res*. 2023;49(2):100-111. doi:10.1080/0361073X.2022.2046947

115. Abd El-Kader SM, Al-Jiffri OH. Aerobic exercise improves quality of life, psychological well-being and systemic inflammation in subjects with alzheimer's disease. *Afr Health Sci*. 2016;16(4):1045-1055. doi:10.4314/ahs.v16i4.22

116. L.F. Law et al. Effects of functional task exercise on everyday problem-solving ability and functional status in older adults with mild cognitive impairment—a randomised controlled trial. *Age Ageing*. 2021;51(7):1-11. doi:10.1093/ageing/afac144

117. Law LLF, Mok VCT, Yau MMK. Effects of functional tasks exercise on cognitive functions of older adults with mild cognitive impairment: A randomized controlled pilot trial. *Alzheimers Res Ther*. 2019;11(1). doi:10.1186/s13195-019-0548-2

118. Cancela JM, Ayán C, Varela S, Seijo M. Effects of a long-term aerobic exercise intervention on institutionalized patients with dementia. *J Sci Med Sport*. 2016;19(4):293-298. doi:10.1016/j.jsams.2015.05.007

119. Yu F, Salisbury D, Mathiason MA. Inter-individual differences in the responses to aerobic exercise in Alzheimer's disease: Findings from the FIT-AD trial. *J Sport Health Sci*. 2021;10(1):65-72. doi:10.1016/j.jshs.2020.05.007

120. Yu F, Vock DM, Zhang L, et al. Cognitive Effects of Aerobic Exercise in Alzheimer's Disease: A Pilot Randomized Controlled Trial. *Journal of Alzheimer's Disease*. 2021;80(1):233-244. doi:10.3233/JAD-201100

121. Salisbury D, Mathiason MA, Yu F. Exercise Dose and Aerobic Fitness Response in Alzheimer's Dementia: Findings from the FIT-AD Trial. *Int J Sports Med*. 2022;43(10):850-858. doi:10.1055/a-1639-2307

122. Baker LD, Pa JA, Katula JA, et al. Effects of exercise on cognition and Alzheimer's biomarkers in a randomized controlled trial of adults with mild cognitive impairment: The EXERT study. *Alzheimer's and Dementia*. 2025;21(4):1-17. doi:10.1002/alz.14586

123. Shadyab AH, Aslanyan V, Jacobs DM, et al. Effects of exercise versus usual care on older adults with amnestic mild cognitive impairment: EXERT versus ADNI. *Alzheimer's and Dementia*. 2025;21(4):1-14. doi:10.1002/alz.70118

124. Huang X, Zhang S, Zhao X, et al. Feasibility and effects of remotely supervised aerobic training and resistance training in older adults with mild cognitive impairment: A pilot three-arm randomised controlled trial. *Gen Psychiatr*. 2025;38(2). doi:10.1136/gpsych-2024-101858

125. Fernandez-Gonzalo R, Fernandez-Gonzalo S, Turon M, Prieto C, Tesch PA, García-Carreira MDC. Muscle, functional and cognitive adaptations after flywheel resistance training in stroke patients: A pilot randomized controlled trial. *J Neuroeng Rehabil*. 2016;13(1):1-11. doi:10.1186/s12984-016-0144-7

126. Singh et al. The Study of Mental and Resistance Training (SMART) Study-Resistance Training and/or Cognitive Training in Mild Cognitive Impairment: A Randomized, Double-Blind, Double-Sham Controlled Trial. *J Am Med Dir Assoc*. 2014;15(12):873-880. doi:10.1016/j.jamda.2014.09.010

127. Lv J, Liu Y. Effects of momentum-based dumbbell training on motor control in older adults with mild cognitive impairment. *Chinese Journal of Rehabilitation Medicine*. 2019;34(5):544-550. doi:10.3969/j.issn.1001-1242.2019.05.009

128. Wang L, Wu B, Tao H, et al. Effects and mediating mechanisms of a structured limbs-exercise program on general cognitive function in older adults with mild cognitive impairment: A randomized controlled trial. *Int J Nurs Stud*. 2020;110:103706. doi:10.1016/j.ijnurstu.2020.103706

129. Vints WAJ, Gökçe E, Šeikinaitė J, et al. Resistance training's impact on blood biomarkers and cognitive function in older adults with low and high risk of mild cognitive impairment: a randomized controlled trial. *European Review of Aging and Physical Activity*. 2024;21(1):1-15. doi:10.1186/s11556-024-00344-9

130. Yoon DH, Lee JY, Song W. Effects of Resistance Exercise Training on Cognitive Function and Physical Performance in Cognitive Frailty: A Randomized Controlled Trial. *Journal of Nutrition, Health and Aging*. 2018;22(8):944-951. doi:10.1007/s12603-018-1090-9

131. Yoon DH, Kang D, Kim HJ, Kim JS, Song HS, Song W. Effect of elastic band-based high-speed power training on cognitive function, physical performance and muscle strength in older women with mild cognitive impairment. *Geriatr Gerontol Int*. 2017;17(5):765-772. doi:10.1111/ggi.12784

132. Lee DW, Yoon DH, Lee JY, Panday SB, Park J, Song W. Effects of High-Speed Power Training on Neuromuscular and Gait Functions in Frail Elderly with Mild Cognitive Impairment Despite Blunted Executive Functions: A Randomized Controlled Trial. *Journal of Frailty and Aging*. 2020;9(3):179-184. doi:10.14283/jfa.2020.23

133. Hong SG, Kim JH, Jun TW. Effects of 12-week resistance exercise on electroencephalogram patterns and cognitive function in the elderly with mild cognitive impairment: A randomized controlled trial. *Clinical Journal of Sport Medicine*. 2018;28(6):500-508. doi:10.1097/JSM.0000000000000476

134. Venturelli M, Lanza M, Muti E, Schena F. Positive effects of physical training in activity of daily living-dependent older adults. *Exp Aging Res*. 2010;36(2):190-205. doi:10.1080/03610731003613771

135. Holthoff VA, Marschner K, Scharf M, et al. Effects of physical activity training in patients with alzheimer's dementia: Results of a pilot RCT study. *PLoS One*. 2015;10(4):1-11. doi:10.1371/journal.pone.0121478

136. Baek JE, Hyeon SJ, Kim M, Cho HY, Hahm SC. Effects of dual-task resistance exercise on cognition, mood, depression, functional fitness, and activities of daily living in older adults with cognitive impairment: a single-blinded, randomized controlled trial. *BMC Geriatr*. 2024;24(1):1-12. doi:10.1186/s12877-024-04942-1

137. Kušleikienė S, Ziv G, Vints WAJ, et al. Cognitive gains and cortical thickness changes after 12 weeks of resistance training in older adults with low and high risk of mild cognitive impairment: Findings from a randomized controlled trial. *Brain Res Bull*. 2025;222(September 2024). doi:10.1016/j.brainresbull.2025.111249

138. Yang JG, Thapa N, Park HJ, et al. Virtual Reality and Exercise Training Enhance Brain, Cognitive, and Physical Health in Older Adults with Mild Cognitive Impairment. *Int J Environ Res Public Health*. 2022;19(20). doi:10.3390/ijerph192013300

139. Doi T, Makizako H, Shimada H, et al. Effects of multicomponent exercise on spatial-temporal gait parameters among the elderly with amnestic mild cognitive impairment (aMCI): Preliminary results from a randomized controlled trial (RCT). *Arch Gerontol Geriatr*. 2013;56(1):104-108. doi:10.1016/j.archger.2012.09.003

140. Uemura K, Doi T, Shimada H, et al. Effects of Exercise Intervention on Vascular Risk Factors in Older Adults with Mild Cognitive Impairment: A Randomized Controlled Trial. *Dement Geriatr Cogn Dis Extra*. 2012;2(1):445-455. doi:10.1159/000343486

141. Suzuki T, Shimada H, Makizako H, et al. A Randomized Controlled Trial of Multicomponent Exercise in Older Adults with Mild Cognitive Impairment. *PLoS One*. 2013;8(4). doi:10.1371/journal.pone.0061483

142. Li L, Liu M, Zeng H, Pan L. Multi-component exercise training improves the physical and cognitive function of the elderly with mild cognitive impairment: A six-month randomized controlled trial. *Ann Palliat Med*. 2021;10(8):8919-8929. doi:10.21037/apm-21-1809

143. Shimada H, Suzuki T, Makizako H, et al. Effects of multicomponent exercise on cognitive function in older adults with amnestic mild cognitive impairment: a randomized controlled trial. *Alzheimer's & Dementia*. 2012;8(4S_Part_4). doi:10.1016/j.jalz.2012.05.386

144. Greblo Jurakic Z, Krizanic V, Sarabon N, Markovic G. Effects of feedback-based balance and core resistance training vs. Pilates training on cognitive functions in older women with mild cognitive impairment: a pilot randomized controlled trial. *Aging Clin Exp Res*. 2017;29(6):1295-1298. doi:10.1007/s40520-017-0740-9

145. Kim J, Yim J. Effects of an exercise protocol for improving handgrip strength and walking speed on cognitive function in patients with chronic stroke. *Medical Science Monitor*. 2017;23:5402-5409. doi:10.12659/MSM.904723

146. Li PWC, Yu DSF, Siu PM, Wong SCK, Chan BS. Peer-supported exercise intervention for persons with mild cognitive impairment: A waitlist randomised controlled trial (the BRAin Vitality Enhancement trial). *Age Ageing*. 2022;51(10):1-10. doi:10.1093/ageing/afac213

147. Avenali M, Picascia M, Tassorelli C, Sinforiani E, Bernini S. Evaluation of the efficacy of physical therapy on cognitive decline at 6-month follow-up in Parkinson disease patients with mild cognitive impairment: a randomized controlled trial. *Aging Clin Exp Res*. 2021;33(12):3275-3284. doi:10.1007/s40520-021-01865-4

148. Mak A, Delbaere K, Refshauge K, et al. Sunbeam Program Reduces Rate of Falls in Long-Term Care Residents With Mild to Moderate Cognitive Impairment or Dementia: Subgroup Analysis of a Cluster Randomized Controlled Trial. *J Am Med Dir Assoc*. 2022;23(5):743-749.e1. doi:10.1016/j.jamda.2022.01.064

149. Sobol NA, Hoffmann K, Frederiksen KS, et al. Effect of aerobic exercise on physical performance in patients with Alzheimer's disease. *Alzheimer's and Dementia*. 2016;12(12):1207-1215. doi:10.1016/j.jalz.2016.05.004

150. Hoffmann K, Sobol NA, Frederiksen KS, et al. Moderate-to-high intensity physical exercise in patients with Alzheimer's disease: A randomized controlled trial. *Journal of Alzheimer's Disease*. 2016;50(2):443-453. doi:10.3233/JAD-150817

151. Sobol NA, Dall CH, Høgh P, et al. Change in fitness and the relation to change in cognition and neuropsychiatric symptoms after aerobic exercise in patients with mild Alzheimer's disease. *Journal of Alzheimer's Disease*. 2018;65(1):137-145. doi:10.3233/JAD-180253

152. Papatsimpas V, Vrouva S, Papathanasiou G, et al. Does Therapeutic Exercise Support Improvement in Cognitive Function and Instrumental Activities of Daily Living in Patients with Mild Alzheimer's Disease? A Randomized Controlled Trial. *Brain Sci*. 2023;13(7). doi:10.3390/brainsci13071112

153. Ullrich P, Werner C, Schönstein A, et al. Effects of a Home-Based Physical Training and Activity Promotion Program in Community-Dwelling Older Persons with Cognitive Impairment after Discharge from Rehabilitation: A Randomized Controlled Trial. *Journals of Gerontology - Series A Biological Sciences and Medical Sciences*. 2022;77(12):2435-2444. doi:10.1093/gerona/glac005

154. Bademli K, Lok N, Canbaz M, Lok S. Effects of Physical Activity Program on cognitive function and sleep quality in elderly with mild cognitive impairment: A randomized controlled trial. *Perspect Psychiatr Care*. 2019;55(3):401-408. doi:10.1111/ppc.12324

155. Lok N, Tosun AS, Lok S, Temel V, Aydın Z. Effect of physical activity program applied to patients with Alzheimer's disease on cognitive functions and depression level: a randomised controlled study. *Psychogeriatrics*. 2023;23(5):856-863. doi:10.1111/psyg.13010

156. De Sá CA, Saretto CB, Cardoso AM, Remor A, Breda CO, da Silva Corralo V. Effects of a physical exercise or motor activity protocol on cognitive function, lipid profile, and BDNF levels in older adults with mild cognitive impairment. *Mol Cell Biochem*. 2024;479(3):499-509. doi:10.1007/s11010-023-04733-z

157. Padala KP, Padala PR, Lensing SY, et al. Home-Based Exercise Program Improves Balance and Fear of Falling in Community-Dwelling Older Adults with Mild Alzheimer's Disease: A Pilot Study. *Journal of Alzheimer's Disease*. 2017;59(2):565-574. doi:10.3233/JAD-170120

158. Langoni CDS, Resende TDL, Barcellos AB, et al. Effect of Exercise on Cognition, Conditioning, Muscle Endurance, and Balance in Older Adults with Mild Cognitive Impairment: A Randomized Controlled Trial. *Journal of Geriatric Physical Therapy*. 2019;42(2):E15-E22. doi:10.1519/JPT.0000000000000191

159. Langoni C da S, Resende T de L, Barcellos AB, et al. The effect of group exercises on balance, mobility, and depressive symptoms in older adults with mild cognitive impairment: a randomized controlled trial. *Clin Rehabil*. 2019;33(3):439-449. doi:10.1177/0269215518815218

160. Zhang Q, Zhu M, Huang L, et al. A Study on the Effect of Traditional Chinese Exercise Combined With Rhythm Training on the Intervention of Older Adults With Mild Cognitive Impairment. *Am J Alzheimers Dis Other Demen*. 2023;38(48):1-12. doi:10.1177/15333175231190626

161. Vreugdenhil A, Cannell J, Davies A, Razay G. A community-based exercise programme to improve functional ability in people with Alzheimer's disease: A randomized controlled trial. *Scand J Caring Sci*. 2012;26(1):12-19. doi:10.1111/j.1471-6712.2011.00895.x

162. Papamichail P, Sagredaki ML, Bouzineki C, Kanellopoulou S, Lyros E, Christakou A. The Effectiveness of an Exercise Program on Muscle Strength and Range of Motion on Upper Limbs, Functional Ability and Depression at Early Stage of Dementia. *J Clin Med*. 2024;13(14):1-10. doi:10.3390/jcm13144136

163. Rivas-Campo Y, Aibar-Almazán A, Afanador-Restrepo DF, et al. Effects of High-Intensity Functional Training (HIFT) on the Functional Capacity, Frailty, and Physical Condition of Older Adults with Mild Cognitive Impairment: A Blind Randomized Controlled Clinical Trial. *Life*. 2023;13(5):1-16. doi:10.3390/life13051224

164. Rivas-Campo Y, Aibar-Almazán A, Rodríguez-López C, et al. Enhancing Cognition in Older Adults with Mild Cognitive Impairment through High-Intensity Functional Training: A Single-Blind Randomized Controlled Trial. *J Clin Med*. 2023;12(12):1-12. doi:10.3390/jcm12124049

165. Prick AE, De Lange J, Scherder E, Twisk J, Pot AM. The effects of a multicomponent dyadic intervention with physical exercise on the cognitive functioning of people with dementia: A randomized controlled trial. *J Aging Phys Act*. 2017;25(4):539-552. doi:10.1123/japa.2016-0038

166. Hauer K, Schwenk M, Zieschang T, Essig M, Becker C, Oster P. Physical training improves motor performance in people with dementia: A randomized controlled trial. *J Am Geriatr Soc*. 2012;60(1):8-15. doi:10.1111/j.1532-5415.2011.03778.x

167. Zieschang T, Schwenk M, Oster P, Hauer K. Sustainability of motor training effects in older people with dementia. *Journal of Alzheimer's Disease*. 2013;34(1):191-202. doi:10.3233/JAD-120814

168. Schwenk M, Zieschang T, Englert S, Grewal G, Najafi B, Hauer K. Improvements in gait characteristics after intensive resistance and functional training in people with dementia: A randomised controlled trial. *BMC Geriatr*. 2014;14(1):1-9. doi:10.1186/1471-2318-14-73

169. Suttanon P, Hill KD, Said CM, et al. Feasibility, safety and preliminary evidence of the effectiveness of a home-based exercise programme for older people with Alzheimer's disease: A pilot randomized controlled trial. *Clin Rehabil*. 2013;27(5):427-438. doi:10.1177/0269215512460877

170. Sanders LMJ, Hortobágyi T, Karssemeijer EGA, Van Der Zee EA, Scherder EJA, Van Heuvelen MJG. Effects of low- And high-intensity physical exercise on physical and cognitive function in older persons with dementia: A randomized controlled trial. *Alzheimers Res Ther*. 2020;12(1):1-15. doi:10.1186/s13195-020-00597-3

171. Dawson N, Judge KS, Gerhart H. Improved Functional Performance in Individuals with Dementia after a Moderate-Intensity Home-Based Exercise Program: A Randomized Controlled Trial. *Journal of Geriatric Physical Therapy*. 2019;42(1):18-27. doi:10.1519/JPT.0000000000000128

172. Santana-Sosa E, Barriopedro MI, López-Mojares LM, Pérez M, Lucia A. Exercise training is beneficial for Alzheimer's patients. *Int J Sports Med*. 2008;29(10):845-850. doi:10.1055/s-2008-1038432

173. Lamb SE, Sheehan B, Atherton N, et al. Dementia And Physical Activity (DAPA) trial of moderate to high intensity exercise training for people with dementia: Randomised controlled trial. *BMJ (Online)*. 2018;361. doi:10.1136/bmj.k1675

174. Smith TO, Mistry D, Lee H, et al. Moderators of Cognitive Outcomes from an Exercise Program in People with Mild to Moderate Dementia. *J Am Geriatr Soc*. 2020;68(9):2095-2100. doi:10.1111/jgs.16552

175. Kovas et al. Effects of a multimodal exercise program on balance, functional mobility and fall risk in older adults with cognitive impairment: a randomized controlled single-blind study. 2011;47(3):381-390.

176. de Oliveira Silva F, Ferreira JV, Plácido J, et al. Three months of multimodal training contributes to mobility and executive function in elderly individuals with mild cognitive impairment, but not in those with Alzheimer's disease: A randomized controlled trial. *Maturitas*. 2019;126(April):28-33. doi:10.1016/j.maturitas.2019.04.217

177. Levinger P, Goh AMY, Dunn J, et al. Exercise interveNtion outdoor proJect in the cOmmunitY – results from the ENJOY program for independence in dementia: a feasibility pilot randomised controlled trial. *BMC Geriatr*. 2023;23(1):1-16. doi:10.1186/s12877-023-04132-5

178. Ghahfarrokhi MM, Shirvani H, Rahimi M, Bazgir B, Shamsadini A, Sobhani V. Feasibility and preliminary efficacy of different intensities of functional training in elderly type 2 diabetes patients with cognitive impairment: a pilot randomised controlled trial. *BMC Geriatr*. 2024;24(1):1-15. doi:10.1186/s12877-024-04698-8

179. Fonte C, Smania N, Pedrinolla A, et al. Comparison between physical and cognitive treatment in patients with MCI and Alzheimer's disease. *Aging*. 2019;11(10):3138-3155. doi:10.18632/aging.101970

180. Gebhard D, Mess F. Feasibility and Effectiveness of a Biography-Based Physical Activity Intervention in Institutionalized People With Dementia: Quantitative and Qualitative Results From a Randomized Controlled Trial. *J Aging Phys Act*. 2022;30(2):237-251. doi:10.1123/japa.2020-0343

181. Akbuga Koc E, Yazici-Mutlu Ç, Cinar N, Sahiner T. Comparison of the effect of online physical exercise and computerized cognitive stimulation in patients with Alzheimer's disease during the Covid-19 pandemic. *Complement Ther Clin Pract*. 2024;57(May):10-20. doi:10.1016/j.ctcp.2024.101881

182. Casas-Herrero Á, Sáez de Asteasu ML, Antón-Rodrigo I, et al. Effects of Vivifrail multicomponent intervention on functional capacity: a multicentre, randomized controlled trial. *J Cachexia Sarcopenia Muscle*. 2022;13(2):884-893. doi:10.1002/jcsm.12925

183. Shaw I, Cronje M, Shaw BS. Group-based exercise as a therapeutic strategy for the improvement of mental outcomes in mild to moderate alzheimer's patients in low resource care facilities. *Asian J Sports Med*. 2021;12(1):1-6. doi:10.5812/asjsm.106593

184. Cezar NO de C, Ansai JH, Oliveira MPB de, et al. Feasibility of improving strength and functioning and decreasing the risk of falls in older adults with Alzheimer's dementia: a randomized controlled home-based exercise trial. *Arch Gerontol Geriatr*. 2021;96(March). doi:10.1016/j.archger.2021.104476

185. Cezar NO de C, Aprahamian I, Ansai JH, et al. Feasibility of reducing frailty components in older adults with Alzheimer's dementia: a randomized controlled home-based exercise trial (AD-HOMEX). *Exp Gerontol*. 2021;150(May). doi:10.1016/j.exger.2021.111390

186. Mollinedo Cardalda I, López A, Cancela Carral JM. The effects of different types of physical exercise on physical and cognitive function in frail institutionalized older adults with mild to moderate cognitive impairment. A randomized controlled trial. *Arch Gerontol Geriatr*. 2019;83(May):223-230. doi:10.1016/j.archger.2019.05.003

187. Bo W, Lei M, Tao S, et al. Effects of combined intervention of physical exercise and cognitive training on cognitive function in stroke survivors with vascular cognitive impairment: a randomized controlled trial. *Clin Rehabil*. 2019;33(1):54-63. doi:10.1177/0269215518791007

188. Kim MJ, Han CW, Min KY, et al. Physical Exercise with Multicomponent Cognitive Intervention for Older Adults with Alzheimer's Disease: A 6-Month Randomized Controlled Trial. *Dement Geriatr Cogn Dis Extra*. 2016;6(2):222-232. doi:10.1159/000446508

189. Bossers WJR, Van Der Woude LHV, Boersma F, Hortobágyi T, Scherder EJA, Van Heuvelen MJG. A 9-Week Aerobic and Strength Training Program Improves Cognitive and Motor Function in Patients with Dementia: A Randomized, Controlled Trial. *American Journal of Geriatric Psychiatry*. 2015;23(11):1106-1116. doi:10.1016/j.jagp.2014.12.191

190. Bossers WJR, van der Woude LHV, Boersma F, Hortobágyi T, Scherder EJA, van Heuvelen MJG. Comparison of Effect of Two Exercise Programs on Activities of Daily Living in Individuals with Dementia: A 9-Week Randomized, Controlled Trial. *J Am Geriatr Soc*. 2016;64(6):1258-1266. doi:10.1111/jgs.14160

191. Telenius EW, Engedal K, Bergland A. Long-term effects of a 12 weeks high-intensity functional exercise program on physical function and mental health in nursing home residents with dementia: A single blinded randomized controlled trial Physical functioning, physical health and activity. *BMC Geriatr*. 2015;15(1):1-11. doi:10.1186/s12877-015-0151-8

192. Telenius EW, Engedal K, Bergland A. Effect of a high-intensity exercise program on physical function and mental health in nursing home residents with dementia: An assessor blinded randomized controlled trial. *PLoS One*. 2015;10(5):1-18. doi:10.1371/journal.pone.0126102

193. Toots A, Littbrand H, Boström G, et al. Effects of exercise on cognitive function in older people with dementia: A randomized controlled trial. *Journal of Alzheimer's Disease*. 2017;60(1):323-332. doi:10.3233/JAD-170014

194. Boström G, Conradsson M, Hörnsten C, et al. Effects of a high-intensity functional exercise program on depressive symptoms among people with dementia in residential care: a randomized controlled trial. *Int J Geriatr Psychiatry*. 2016;31(8):868-878. doi:10.1002/gps.4401

195. Toots A, Lindelöf N, Littbrand H, et al. Effects of a High-Intensity Functional Exercise Program on Dependence in Activities of Daily Living and Balance in Older Adults with Dementia. *J Am Geriatr Soc*. 2016;64(1):55-64. doi:10.1111/jgs.13880

196. Toots A, Littbrand H, Holmberg H, et al. Walking Aids Moderate Exercise Effects on Gait Speed in People With Dementia: A Randomized Controlled Trial. *J Am Med Dir Assoc*. 2017;18(3):227-233. doi:10.1016/j.jamda.2016.09.003

197. Toots A, Lundin-Olsson L, Nordström P, Gustafson Y, Rosendahl E. Exercise effects on backward walking speed in people with dementia: A randomized controlled trial. *Gait Posture*. 2021;85(January):65-70. doi:10.1016/j.gaitpost.2020.12.028

198. Henskens M, Nauta IM, Van Eekeren MCA, Scherder EJA. Effects of Physical Activity in Nursing Home Residents with Dementia: A Randomized Controlled Trial. *Dement Geriatr Cogn Disord*. 2018;46(1-2):60-80. doi:10.1159/000491818

199. Kemoun G, Thibaud M, Roumagne N, et al. Effects of a physical training programme on cognitive function and walking efficiency in elderly persons with dementia. *Dement Geriatr Cogn Disord*. 2010;29(2):109-114. doi:10.1159/000272435

200. de Souto Barreto P, Cesari M, Denormandie P, Armaingaud D, Vellas B, Rolland Y. Exercise or Social Intervention for Nursing Home Residents with Dementia: A Pilot Randomized, Controlled Trial. *J Am Geriatr Soc*. 2017;65(9):E123-E129. doi:10.1111/jgs.14947

201. Maltais M, Rolland Y, Vellas B, et al. Effect of Exercise on Behavioral Symptoms and Pain in Patients With Dementia Living in Nursing Homes. *Am J Alzheimers Dis Other Demen*. 2019;34(2):89-94. doi:10.1177/1533317518803773

202. Roach KE, Tappen RM, Kirk-Sanchez N, Williams CL, Loewenstein D. A randomized controlled trial of an activity specific exercise program for individuals with alzheimer disease in long-term care settings. *Journal of Geriatric Physical Therapy*. 2011;34(2):50-56. doi:10.1519/JPT.0b013e31820aab9c

203. Rolland Y, Pillard F, Klapouszczak A, et al. Exercise program for nursing home residents with Alzheimer's disease: A 1-year randomized, controlled trial. *J Am Geriatr Soc*. 2007;55(2):158-165. doi:10.1111/j.1532-5415.2007.01035.x

204. Stevens J, Killeen M. A randomised controlled trial testing the impact of exercise on cognitive symptoms and disability of residents with dementia. *Contemporary nurse : a journal for the Australian nursing profession*. 2006;21(1):32-40. doi:10.5172/conu.2006.21.1.32

205. Brett L, Stapley P, Meedya S, Traynor V. Effect of physical exercise on physical performance and fall incidents of individuals living with dementia in nursing homes: a randomized controlled trial. *Physiother Theory Pract*. 2021;37(1):38-51. doi:10.1080/09593985.2019.1594470

206. Almeida S, Paixão C, da Silva MG, Marques A. Lifestyle-integrated functional exercise for people with Dementia: A pilot study. *J Aging Phys Act*. 2021;29(5):771-780. doi:10.1123/JAPA.2020-0349

207. Verdelho A, Correia M, Gonçalves-Pereira M, et al. Physical Activity in Mild Vascular Cognitive Impairment: Results of the AFIVASC Randomized Controlled Trial at 6 Months. *Journal of Alzheimer's Disease*. 2024;101(4):1379-1392. doi:10.3233/JAD-240246

208. Yan Y, Xu Y, Wang X, et al. The effect of multi-component exercise intervention in older people with Parkinson's disease and mild cognitive impairment: A randomized controlled study. *Geriatr Nurs (Minneap)*. 2024;60:137-145. doi:10.1016/j.gerinurse.2024.08.028

209. Shokri G, Mohammadian F, Noroozian M, Amani-Shalamzari S, Suzuki K. Effects of remote combine exercise-music training on physical and cognitive performance in patients with Alzheimer's disease: a randomized controlled trial. *Front Aging Neurosci*. 2023;15(January):1-9. doi:10.3389/fnagi.2023.1283927
