# Supplementary material for: Dance versus other exercise modalities in mild cognitive impairment and dementia: comparative efficacy from a systematic review and bayesian network meta-analysis
Source: Front Physiol. 2026 Mar 25;17:1782774. doi: 10.3389/fphys.2026.1782774 (PMC13056856; doi:10.3389/fphys.2026.1782774)
Supplement: Supplementary file 3 [file Table1.pdf]

Supplementary Table 1. Search Strategy in Databases

Step	MeSH terms
**#1**<br><br>**Title and Abstract**	(Dance OR Dancing OR Tango OR Waltz OR Ballet OR Jazz OR Folk dance OR Ballroom dance OR Sport-dance OR Square-dance OR Salsa OR Hip Pop OR Line Dancing OR Tap Dance OR Dance movement therapy OR Exercise OR Physical activit* OR training* OR Yoga OR Tai Chi OR Qigong OR Baduanjin)
**#2**<br><br>**Title and Abstract**	(Dementia OR Alzheimer's disease OR Cognitive impairment OR Neurocognitive disorder* OR Cognitive disorder*)
**#3**<br><br>**All Fields**	(Randomized controlled trial OR Randomized controlled trial OR RCT)
**#4**	#1 AND #2 AND #3
