# Supplementary material for: Dance versus other exercise modalities in mild cognitive impairment and dementia: comparative efficacy from a systematic review and bayesian network meta-analysis
Source: Front Physiol. 2026 Mar 25;17:1782774. doi: 10.3389/fphys.2026.1782774 (PMC13056856; doi:10.3389/fphys.2026.1782774)
Supplement: Supplementary file 4 [file Table2.pdf]

Supplementary Table 2. Summary of Characteristics of the Included Studies

Author/ Year/ Type of RCT	Diagnosis/ Instrument	MMSE (baseline)	MoCA (baseline)	Sample size (female)	Age	Adherence rate (%)	Education level	Intervention	Training intensity
**Dance**									
**Kropacova et al.[1] 2019 2-arm RCT**	**Diagnosis:** Healthy senior and MCI **Instrument:** Cognitive test battery	N/A	**IG:** 29.96 ± 2.88 **CG:** 26.06 ± 2.71	**IG:** 49 (41) **CG:** 50 (35)	**IG:** 69.16 ± 5.36 **CG:** 68.37 ± 6.10	**IG:** 82% **CG:** 83%	**IG:** 14.51 ± 2.56 **CG:** 14.80 ± 3.10	**IG:** Dance movement intervention **CG:** Life-as-usual	**Intensity:** N/A **Duration:** 24-week **Training load:** 60-min, 3 times/week
**Esmail et al.[2] 2020 3-arm RCT**	**Diagnosis:** Older inactive adults **Instrument:** MMSE	**IG[1]:** 27.83 ± 1.53 **IG[2]:** 27.80 ± 1.42 **CG:** 28.43 ± 1.45	N/A	**IG[1]:** 12 (8) **IG[2]:** 15 (11) **CG:** 14 (12)	**IG[1]:** 68.08 ± 7.59 **IG[2]:** 67.20 ± 4.20 **CG:** 67.21 ± 4.12	**IG[1]:** 52% **IG[2]:** 71% **CG:** 78%	**IG[1]:** 14.50 ± 3.12 **IG[2]:** 16.00 ± 4.47 **CG:** 15.21 ± 4.02	**IG[1]:** Dance/movement training (dance therapy program) **IG[2]:** Aerobic exercise training (seated recumbent bicycle) **CG:** Control	**Intensity:** N/A **Duration:** 12-week **Training load:** 60-min, 3 times/week
**Bisbe et al.[3] 2020 2-arm RCT**	**Diagnosis:** aMCI **Instrument:** MMSE, CDR	**IG:** 27.24 ± 1.92 **CG:** 27.36 ± 2.09	N/A	**IG:** 17 (8) **CG:** 14 (7)	**IG:** 72.88 ± 5.60 **CG:** 77.29 ± 5.16	**IG:** 94% **CG:** 78%	**IG:** 7.88 ± 3.79 **CG:** 8.64 ± 3.92	**IG:** Choreography aerobic dances **CG:** Multimodal physical therapy program (strength, endurance, flexibility, balance, coordination gait)	**Intensity:** Without exceeding 2–3/10 RPE **Duration:** 12-week **Training load:** 60-min, 2 times/week
**Qi et al.[4] 2018 2-arm RCT**	**Diagnosis:** MCI **Instrument:** NIA-AA, MMSE, MoCA	**IG:** 27.3 ± 1.3 **CG:** 27.1 ± 1.2	**IG:** 22.6 ± 2.1 **CG:** 22.9 ± 1.7	**IG:** 16 (11) **CG:** 16 (12)	**IG:** 70.6 ± 6.2 **CG:** 69.1 ± 8.1	**IG:** 84% **CG:** 84%	**IG:** 10.4 ± 2.6 **CG:** 9.7 ± 2.7	**IG:** Aerobic dance intervention **CG:** Usual care	**Intensity:** 60%–80% of HRmax **Duration:** 12-week **Training load:** 35-min, 3 times/week
**Lazarou et al.[5] 2017 2-arm RCT**	**Diagnosis:** MCI **Instrument:** Petersen criteria, GDS	**IG:** 27.6 ± 2.19 **CG:** 26.88 ± 2.1	**IG:** 24.70 ± 2.25 **CG:** 23.81 ± 3.1	**IG:** 66 (53) **CG:** 63 (48)	**IG:** 65.89 ± 10.76 **CG:** 67.92 ± 9.47	**IG:** 74% **CG:** 97%	**IG:** 11.40 ± 4.47 **CG:** 10.31 ± 4.68	**IG:** International Ballroom Dancing **CG:** Control	**Intensity:** N/A **Duration:** 40-week **Training load:** 60-min, 2 times/week
**Franco et al.[6] 2020**	**Diagnosis:** MCI	**IG:** 26.9 ± 1.9	**IG:** 22.7 ± 3.8	**IG:** 35 (34)	**IG:** 68.6 ±	**IG:** 85% **CG:** 88%	N/A	**IG:** Senior dance program and education class	**Intensity:** Moderate

**2-arm RCT**	**Instrument:** MMSE	**CG:** 26.4 ± 3.0	**CG:** 22.0 ± 4.2	**CG:** 36 (31)	7.2<br>**CG:** 70.0 ± 6.2			**CG:** Education class only	**Duration:** 12-week<br>**Training load:** 60-min, 2 times/week
**Doi et al.**[7]<br>**2017**<br>**3-arm RCT**	**Diagnosis:** MCI<br>**Instrument:** Petersen criteria	**IG^1:** 26.0 ± 2.6<br>**IG^2:** 25.9 ± 2.6<br>**CG:** 25.8 ± 2.4	N/A	**IG^1:** 67 (34)<br>**IG^2:** 67 (39)<br>**CG:** 67 (31)	**IG^1:** 75.7 ± 4.1<br>**IG^2:** 76.2 ± 4.6<br>**CG:** 76.0 ± 4.9	**IG^1:** 82%<br>**IG^2:** 81%<br>**CG:** 94%	**IG^1:** 11.3 ± 2.5<br>**IG^2:** 11.6 ± 3.0<br>**CG:** 12.0 ± 2.4	**IG^1:** Dance program<br>**IG^2:** Playing musical instruments (percussion instruments)<br>**CG:** Health education control (information regarding the aging process, falls, health diet, oral care, and frailty)	**Intensity:** N/A<br>**Duration:** 40-week<br>**Training load:** 60-min, 1 time/week
**Blumen et al.**[8]<br>**2023**<br>**2-arm RCT**	**Diagnosis:** Dementia-at-risk<br>**Instrument:** MoCA	N/A	**IG:** 22.54 ± 3.93<br>**CG:** 23.17 ± 3.90	**IG:** 13 (8)<br>**CG:** 12 (6)	**IG:** 77.40 ± 5.82<br>**CG:** 75.43 ± 5.84	**IG:** 62%<br>**CG:** 67%	**IG:** 16.62 ± 4.61<br>**CG:** 14.67 ± 4.23	**IG:** Social dance<br>**CG:** Treadmill walking	**Intensity**: ≤ 14/20 RPE<br>**Duration:** 24-week<br>**Training load:** 90-min, 2 times/week
**Zhu et al.**[9]<br>**2018**<br>**2-arm RCT**	**Diagnosis:** MCI<br>**Instrument:** NIA-AA, MMSE, MoCA	N/A	**IG:** 23.2 ± 1.9<br>**CG:** 22.9 ± 2.1	**IG:** 29 (15)<br>**CG:** 31 (21)	**IG:** 70.3 ± 6.7<br>**CG:** 69.0 ± 7.3	**IG:** 93%<br>**CG:** 100%	N/A	**IG:** Aerobic brain dance routine<br>**CG:** Usual care	**Intensity:** 60%–80% of HRmax<br>**Duration:** 12-week<br>**Training load:** 35-min, 3 times/week
**Song et al.**[10]<br>**2024**<br>**2-arm RCT**	**Diagnosis:** MCI<br>**Instrument:** MoCA	N/A	**IG:** 21.11 ± 2.03<br>**CG:** 22.02 ± 1.96	**IG:** 45 (36)<br>**CG:** 44 (32)	**IG:** 76.71 ± 5.96<br>**CG:** 75.20 ± 6.63	**IG:** 80%<br>**CG:** 77%	N/A	**IG:** Aerobic dancing program<br>**CG:** Health education	**Intensity:** N/A<br>**Duration:** 16-week<br>**Training load:** 60-min, 3 times/week
**Chang et al.**[11,12]<br>**2021**<br>**2-arm RCT**	**Diagnosis:** MCI<br>**Instrument:** MoCA	N/A	**IG:** 21.61 ± 2.11<br>**CG:** 21.49 ± 2.39	**IG:** 62 (62)<br>**CG:** 47 (47)	**IG:** 76.56 ± 3.60<br>**CG:** 75.94 ± 3.61	**IG:** 86%<br>**CG:** 73%	**IG:** 8.73 ± 2.05<br>**CG:** 8.28 ± 2.06	**IG:** Chinese square dancing<br>**CG:** Control	**Intensity:** 100–140 beats/min HR<br>**Duration:** 18-week<br>**Training load:** 40-min, 3 times/week
**Winckel et al.**[13]<br>**2004**<br>**2-arm RCT**	**Diagnosis:** Moderate to severe dementia<br>**Instrument:**	**IG:** 12.87 ± 5.01<br>**CG:** 10.80 ± 5.01	N/A	**IG:** 15 (15)<br>**CG:** 10 (10)	**IG:** 81.33 ± 4.24<br>**CG:**	**IG:** 100%<br>**CG:** 100%	N/A	**IG:** Dance therapy<br>**CG:** Conversation control	**Intensity:** N/A<br>**Duration:** 12-week<br>**Training load:** 30-

	NINCDS-ARDRA, MMSE				81.90 ± 4.18				min, 7 times/week
**Bracco et al.**[14]<br>**2023**<br>**2-arm RCT**	**Diagnosis:** Dementia<br>**Instrument:** MMSE	**IG:** 7 ± 5<br>**CG:** 4 ± 5	N/A	**IG:** 15 (11)<br>**CG:** 16 (15)	**IG:** 81 ± 8<br>**CG:** 85 ± 5	**IG:** 87%<br>**CG:** 81%	N/A	**IG:** Therapeutic tango program<br>**CG:** Physical exercise	**Intensity:** N/A<br>**Duration:** 12-week<br>**Training load:** 60-min, 2 times/week
**Zhu et al.**[15]<br>**2022**<br>**2-arm RCT**	**Diagnosis:** aMCI<br>**Instrument:** NIA-AA, MMSE, MoCA	≥ 25	≤ 26	**IG:** 35 (18)<br>**CG:** 33 (23)	**IG:** 71.51 ± 6.62<br>**CG:** 69.82 ± 7.74	**IG:** 94%<br>**CG:** 100%	**IG:** 10.49 ± 3.94<br>**CG:** 9.49 ± 4.14	**IG:** Aerobic dance and health education<br>**CG:** Health education only	**Intensity:** 60%–80% of HRmax<br>**Duration:** 12-week<br>**Training load:** 35-min, 3 times/week
**Ho et al.**[16]<br>**2018**<br>**3-arm RCT**	**Diagnosis:** Mild dementia<br>**Instrument:** CDR	N/A	N/A	**IG^1:** 69 (56)<br>**IG^2:** 67 (56)<br>**CG:** 68 (55)	**IG^1:** 79.4 ± 7.6<br>**IG^2:** 79.3 ± 8.1<br>**CG:** 78.3 ± 8.4	N/A	N/A	**IG^1:** Dance movement therapy<br>**IG^2:** Mild to moderate exercise<br>**CG:** Regular medication and routine care	**Intensity:** 40–60% of VO_2max<br>**Duration:** 12-week<br>**Training load:** 60-min, 2 times/week
**Thiel et al.**[17]<br>**2024**<br>**2-arm RCT**	**Diagnosis:** MCI<br>**Instrument:** MMSE	**IG:** 27.1 ± 1.5<br>**CG:** 27.3 ± 1.2	N/A	**IG:** 26 (16)<br>**CG:** 24 (11)	**IG:** 70.7 ± 5.6<br>**CG:** 69.1 ± 6.8	91%	**IG:** 15.8 ± 2.7<br>**CG:** 15.3 ± 2.1	**IG:** Dance training (line dance, jazz, square dance, and Latin-American dances)<br>**CG:** Control	**Intensity:** N/A<br>**Duration:** 24-week<br>**Training load:** 90-min, 2 times/week
**Sanchez-Alcala et al.**[18,19]<br>**2025**<br>**2-arm RCT**	**Diagnosis:** MCI<br>**Instrument:** MMSE	**IG:** 21.49 ± 1.74<br>**CG:** 21.36 ± 1.76	**IG:** 21.36 ± 1.07<br>**CG:** 21.42 ± 1.03	**IG:** 47 (29)<br>**CG:** 45 (29)	**IG:** 71.43 ± 2.97<br>**CG:** 72.24 ± 2.92	**IG:** 98%<br>**CG:** 94%	N/A	**IG:** Dance-based aerobic training program<br>**CG:** Control	**Intensity:** N/A<br>**Duration:** 12-week<br>**Training load:** 60-min, 2 times/week
**Exergaming**									
**Eggenberger et al.**[20,21]<br>**2015**<br>**3-arm RCT**	**Diagnosis:** Older people<br>**Instrument:** MMSE	**IG^1:** 28.4 ± 1.4<br>**IG^2:** 28.3 ± 1.2<br>**IG^3:** 28.0	N/A	**IG^1:** 24 (14)<br>**IG^2:** 22 (16)<br>**IG^3:** 25	**IG^1:** 77.3 ± 6.3<br>**IG^2:** 78.5 ±	**IG^1:** 80%<br>**IG^2:** 76%<br>**IG^3:** 83%	**IG^1:** 13.7 ± 1.5<br>**IG^2:** 13.9 ± 2.1<br>**IG^3:** 12.0	**IG^1:** Video game dancing and complementary strength and balance exercises<br>**IG^2:** Treadmill memory training and complementary	**Intensity:** 5–7/10 RPE<br>**Duration:** 24-week<br>**Training load:** 60-

		± 1.7		(16)	5.1<br>**IG^3:**<br>80.8 ±<br>4.7		± 2.1	strength and balance exercises<br>**IG^3:** Treadmill walking and complementary strength and balance exercises	min, 2 times/week
**Hughes et al.[22]<br>2014<br>2-arm RCT**	**Diagnosis:**<br>MCI<br>**Instrument:**<br>MMSE	**IG:** 27.2 ± 1.9<br>**CG:** 27.1 ± 1.8	N/A	**IG:** 10 (8)<br>**CG:** 10 (6)	**IG:**<br>78.5 ±<br>7.1<br>**CG:**<br>76.2 ±<br>4.3	**IG:** 100%<br>**CG:** 100%	**IG:** 13.8 ± 2.4<br>**CG:** 13.1 ± 1.9	**IG:** Nintendo Wii intervention<br>**CG:** Health aging education program (age-specific health-related topics)	**Intensity:** N/A<br>**Duration:** 24-week<br>**Training load:** 90-min, 1 time/week
**Schwenk et al.[23]<br>2016<br>2-arm RCT**	**Diagnosis:**<br>MCI<br>**Instrument:**<br>MoCA	N/A	**IG:** 23.3 ± 3.1<br>**CG:** 22.4 ± 3.0	**IG:** 12 (7)<br>**CG:** 10 (5)	**IG:**<br>77.8 ±<br>6.9<br>**CG:**<br>79.0 ±<br>10.4	**IG:** 92%<br>**CG:** 90%	**IG:** 14.2 ± 2.3<br>**CG:** 15.9 ± 2.7	**IG:** Sensor-based balance training<br>**CG:** Control	**Intensity:** N/A<br>**Duration:** 4-week<br>**Training load:** 45-min, 2 times/week
**Liu et al.[24]<br>2022<br>3-arm RCT**	**Diagnosis:**<br>MCI<br>**Instrument:**<br>Petersen criteria	**IG^1:** 25.1 ± 1.7<br>**IG^2:** 25.8 ± 2.4<br>**CG:** 26.6 ± 2.2	**IG^1:** 22.6 ± 2.5<br>**IG^2:** 21.8 ± 3.6<br>**CG:** 23.2 ± 2.8	**IG^1:** 16 (12)<br>**IG^2:** 17 (12)<br>**CG:** 17 (11)	**IG^1:**<br>74.6 ±<br>6.1<br>**IG^2:**<br>73.2 ±<br>6.3<br>**CG:**<br>73.4 ±<br>6.5	**IG^1:** 89%<br>**IG^2:** 94%<br>**CG:** 94%	N/A	**IG^1:** Exergaming-based Tai Chi<br>**IG^2:** Traditional Tai Chi<br>**CG:** Control	**Intensity:** 12–14/20 RPE<br>**Duration:** 12-week<br>**Training load:** 50-min, 3 times/week
**Padala et al.[25]<br>2012<br>2-arm RCT**	**Diagnosis:**<br>Mild AD<br>**Instrument:**<br>DSM, MMSE	**IG:** 22.6 ± 4.3<br>**CG:** 24.9 ± 3.6	N/A	**IG:** 11 (8)<br>**CG:** 11 (8)	**IG:**<br>79.3 ±<br>9.8<br>**CG:**<br>81.6 ±<br>5.2	**IG:** 100%<br>**CG:** 100%	**IG:** 13.8 ± 2.1<br>**CG:** 14.0 ± 2.0	**IG:** Wii-Fit intervention<br>**CG:** Walking	**Intensity:** N/A<br>**Duration:** 8-week<br>**Training load:** 30-min, 5 times/week
**Liao et al.[26]<br>2021<br>2-arm RCT**	**Diagnosis:**<br>MCI<br>**Instrument:**<br>MMSE, MoCA	**IG:** 22.5 ± 4.0<br>**CG:** 23.4 ± 4.4	**IG:** 18.0 ± 5.8<br>**CG:** 19.0 ± 5.9	**IG:** 25 (16)<br>**CG:** 21 (15)	**IG:**<br>79.6 ±<br>9.0<br>**CG:**<br>83.8 ±<br>5.1	**IG:** 81%<br>**CG:** 70%	N/A	**IG:** Kinect based exergaming<br>**CG:** Combined physical exercise (resistance exercise, aerobic exercise, and balance exercise)	**Intensity:** 50%–75% of HRmax, 13–14/20 RPE<br>**Duration:** 12-week<br>**Training load:** 60-min, 3 times/week
**Karssemeijer et al.[27,28]<br>2019**	**Diagnosis:**<br>Dementia<br>**Instrument:**	**IG^1:** 22.9 ± 3.4<br>**IG^2:** 22.5	N/A	**IG^1:** 38 (18)<br>**IG^2:** 38	**IG^1:**<br>79.0 ±<br>6.9	**IG^1:** 89%<br>**IG^2:** 87%<br>**CG:** 87%	N/A	**IG^1:** Exergame training<br>**IG^2:** Single aerobic exercise cycling training	**Intensity:** 65%–75% of HRR<br>**Duration:** 12-

**3-arm RCT**	DSM, MMSE	± 3.1<br>**CG:** 21.9 ± 3.1		(17)<br>**CG:** 39 (18)	**IG[2]:** 80.9 ± 6.1<br>**CG:** 79.8 ± 6.5			**CG:** Active control: relaxation and flexibility exercise	week<br>**Training load:** 30-50-min, 3 times/week
**Santen et al.[29] 2020 2-arm RCT**	**Diagnosis:** Dementia<br>**Instrument:** MMSE	**IG:** 18.1 ± 6.7<br>**CG:** 19.4 ± 6.5	N/A	**IG:** 73 (36)<br>**CG:** 39 (16)	**IG:** 79.0 ± 6.0<br>**CG:** 79.0 ± 7.0	**IG:** 76%<br>**CG:** 84%	N/A	**IG:** Exergaming intervention (*caregiver involved*)<br>**CG:** Regular activity program (arts and crafts, music, and walking)	**Intensity:** N/A<br>**Duration:** 24-week<br>**Training load:** 60-min, 2–5 times/week
**Wu et al.[30] 2023 2-arm RCT**	**Diagnosis:** Dementia<br>**Instrument:** MMSE, Korean consortium to establish a registry for Alzheimer's Disease test battery	**IG:** 17.8 ± 3.7<br>**CG:** 18.8 ± 2.5	N/A	**IG:** 13 (8)<br>**CG:** 11 (8)	**IG:** 78.8 ± 4.8<br>**CG:** 81.2 ± 4.4	**IG:** 50%<br>**CG:** 42%	N/A	**IG:** Exergaming intervention<br>**CG:** Aerobic exercise internation (recumbent cycling)	**Intensity:** 60%–70% of HRmax<br>**Duration:** 12-week<br>**Training load:** 35-50-min, 3 times/week
**Swinnen et al.[31] 2021 2-arm RCT**	**Diagnosis:** Major neurocognitive disorder<br>**Instrument:** MMSE	**IG:** 18 ± 4.4<br>**CG:** 17 ± 4.2	**IG:** 9.4 ± 4.1<br>**CG:** 8.5 ± 5.2	**IG:** 23 (18)<br>**CG:** 22 (17)	**IG:** 84.7 ± 5.6<br>**CG:** 85.3 ± 6.5	**IG:** 82%<br>**CG:** 81%	N/A	**IG:** Exergame intervention<br>**CG:** Usual care	**Intensity:** N/A<br>**Duration:** 8-week<br>**Training load:** 15-min, 3 times/week
**Zheng et al.[32] 2022 2-arm RCT**	**Diagnosis:** Dementia<br>**Instrument:** Diagnosed by a specialist	**IG:** 14.06 ± 6.66<br>**CG:** 13.95 ± 7.37	N/A	**IG:** 18 (15)<br>**CG:** 20 (14)	**IG:** 81.74 ± 5.79<br>**CG:** 84.26 ± 5.48	**IG:** 75%<br>**CG:** 83%	N/A	**IG:** Exercise intervention (Kinect game)<br>**CG:** Usual care	**Intensity:** N/A<br>**Duration:** 8-week<br>**Training load:** 60-min, 5 times/week
**Ugur and Sertel[33] 2025 2-arm RCT**	**Diagnosis:** AD<br>**Instrument:** NINCDS-ADRDA	**IG:** 17.06 ± 2.57<br>**CG:** 16.13 ± 3.36	N/A	**IG:** 16 (5)<br>**CG:** 16 (4)	**IG:** 73.75 ± 5.16<br>**CG:** 73.13 ± 3.54	**IG:** 100%<br>**CG:** 100%	N/A	**IG:** Game-based exercise<br>**CG:** Control	**Intensity:** N/A<br>**Duration:** 6-week<br>**Training load:** 30-min, 2 times/week
**Yoga**									
**Grzenda et al.[34]**	**Diagnosis:**	**IG:** 28.46	N/A	**IG:** 40	**IG:**	N/A	**IG:** 16.15	**IG:** Kundalini yoga training	**Intensity:** N/A

**2024**<br>**2-arm RCT**	Older women at risk of AD<br>**Instrument:** MMSE	± 1.71<br>**CG:** 28.41 ± 1.09		(40)<br>**CG:** 39 (39)	65.45 ± 9.11<br>**CG:** 67.54 ± 9.30		± 1.90<br>**CG:** 15.72 ± 1.99	**CG:** Memory enhancement training (memory strategies)	**Duration:** 12-week<br>**Training load:** 60-min, 1 time/week
**Khanthong et al.[35]**<br>**2021**<br>**2-arm RCT**	**Diagnosis:** MCI<br>**Instrument:** Diagnosed by a neurologist, MMSE, MoCA	**IG:** 26.91 ± 1.85<br>**CG:** 26.19 ± 1.67	**IG:** 20.31 ± 3.31<br>**CG:** 18.50 ± 2.92	**IG:** 35 (31)<br>**CG:** 36 (25)	**IG:** 60.26 ± 5.67<br>**CG:** 61.47 ± 7.49	**IG:** 76%<br>**CG:** 80%	N/A	**IG:** Traditional Thai exercise (Ruesi Dadton)<br>**CG:** Control	**Intensity:** N/A<br>**Duration:** 12-week<br>**Training load:** 60-min, 3 times/week
**Kashyap et al.[36]**<br>**2022**<br>**2-arm RCT**	**Diagnosis:** MCI due to stroke<br>**Instrument:** MoCA	N/A	**IG:** 20 (17-22.25)<br>**CG:** 19 (15.75-21)	**IG:** 40 (12)<br>**CG:** 40 (13)	**IG:** 52.85 ± 13.70<br>**CG:** 55.18 ± 13.24	75%	N/A	**IG:** Hatha yoga<br>**CG:** Routine standard care	**Intensity:** N/A<br>**Duration:** 24-week<br>**Training load:** 60-min, 4–5 times/week
**Tremont et al.[37]**<br>**2022**<br>**2-arm RCT**	**Diagnosis:** MCI<br>**Instrument:** NIA-AA	N/A	N/A	**IG:** 25 (15)<br>**CG:** 21 (1)	**IG:** 71.56 ± 5.80<br>**CG:** 71.67 ± 6.27	**IG:** 100%<br>**CG:** 100%	**IG:** 15.52 ± 3.72<br>**CG:** 14.67 ± 2.96	**IG:** Yoga intervention<br>**CG:** Healthy living education	**Intensity:** N/A<br>**Duration:** 12-week<br>**Training load:** 60-min, 2 times/week
**Eyre et al.[38,39]**<br>**2017**<br>**2-arm RCT**	**Diagnosis:** MCI<br>**Instrument:** CDR	N/A	N/A	**IG:** 38 (25)<br>**CG:** 41 (27)	**IG:** 68.1 ± 8.7<br>**CG:** 67.6 ± 8.0	**IG:** 76%<br>**CG:** 78%	**IG:** 17.4 ± 3.4<br>**CG:** 16.7 ± 3.3	**IG:** Kundalini yoga program<br>**CG:** Memory enhancement training	**Intensity:** N/A<br>**Duration:** 12-week<br>**Training load:** 60-min, 1 time/week
**Chinese traditional exercise (CTE)**									
**Li et al.[40]**<br>**2022**<br>**3-arm RCT**	**Diagnosis:** MCI<br>**Instrument:** CDR, MMSE	**IG¹:** 26.95 ± 1.91<br>**IG²:** 26.96 ± 1.87<br>**CG:** 26.88 ± 2.19	**IG¹:** 25.09 ± 2.43<br>**IG²:** 25.04 ± 2.87<br>**CG:** 25.13 ± 2.19	**IG¹:** 22 (8)<br>**IG²:** 23 (16)<br>**CG:** 24 (15)	**IG¹:** 74.5 ± 5.6<br>**IG²:** 74.4 ± 5.1<br>**CG:** 74.9 ± 6.3	**IG¹:** 91%<br>**IG²:** 83%<br>**CG:** 88%	N/A	**IG¹:** Cognitive enhanced Tai Ji Quan training<br>**IG²:** Standard Tai Ji Quan training (online)<br>**CG:** Stretching exercise	**Intensity:** Light to moderate<br>**Duration:** 16-week<br>**Training load:** 60-min, 2 times/week

**Sungkarat et al.**[41,42] **2018** **2-arm RCT**	**Diagnosis:** aMCI **Instrument:** Petersen criteria, MMSE, MoCA	**IG:** 26.5 ± 1.7 **CG:** 25.8 ± 2.3	**IG:** 21.2 ± 3.4 **CG:** 20.4 ± 3.8	**IG:** 33 (31) **CG:** 33 (26)	**IG:** 68.3 ± 6.7 **CG:** 67.5 ± 7.3	**IG:** 88% **CG:** 82%	**IG:** 11.4 ± 5.1 **CG:** 9.3 ± 5.5	**IG:** Tai Chi practice **CG:** Education control (received information related to cognitive impairment and fall prevention)	**Intensity:** N/A **Duration:** 24-week **Training load:** 50-min, 3 times/week
**Li et al.**[43] **2021** **2-arm RCT**	**Diagnosis:** MCI **Instrument:** CDR, MMSE	**IG:** 25.07 ± 0.80 **CG:** 25.27 ± 1.03	N/A	**IG:** 15 (9) **CG:** 15 (12)	**IG:** 76.13 ± 6.2 **CG:** 76.20 ± 6.3	**IG:** 87% **CG:** 87%	N/A	**IG:** Tai Ji Quan (online) **CG:** Stretching exercise	**Intensity:** N/A **Duration:** 24-week **Training load:** 60-min, 2 times/week
**Lam et al.**[44,45] **2012** **2-arm RCT**	**Diagnosis:** Risk of cognitive decline **Instrument:** CDR	**IG:** 24.7 ± 3.0 **CG:** 24.3 ± 2.9	N/A	**IG:** 171 (125) **CG:** 218 (172)	**IG:** 77.2 ± 6.3 **CG:** 78.3 ± 6.6	**IG:** 54% **CG:** 78%	**IG:** 4.1 ± 4.3 **CG:** 2.6 ± 3.2	**IG:** Tai Chi intervention **CG:** Active control	**Intensity:** N/A **Duration:** 48-week **Training load:** 30-min, 3 times/week
**Jiayuan et al.**[46] **2022** **3-arm RCT**	**Diagnosis:** Frailty older adults **Instrument:** CDR	**IG^1:** 23.9 ± 2.6 **IG^2:** 24.2 ± 1.7 **IG^3:** 24.5 ± 1.6	N/A	**IG^1:** 30 (17) **IG^2:** 31 (18) **IG^3:** 30 (16)	**IG^1:** 70.8 ± 4.2 **IG^2:** 71.7 ± 3.9 **IG^3:** 71.3 ± 5.0	**IG^1:** 97% **IG^2:** 100% **IG^3:** 100%	N/A	**IG^1:** Mindfulness (body scan, walking meditation, gentle yoga, sitting mediation) **IG^2:** Tai Chi Chuan (24-Simplified Tai Chi Chuan) **IG^3:** Mindfulness-based Tai Chi Chuan	**Intensity:** N/A **Duration:** 24-week **Training load:** 60-min, 2 times/week
**Chen et al.**[47] **2023** **3-arm RCT**	**Diagnosis:** MCI due to T2D **Instrument:** MoCA	N/A	**IG^1:** 21.38 ± 2.77 **IG^2:** 21.52 ± 2.57 **CG:** 21.34 ± 2.85	**IG^1:** 107 (58) **IG^2:** 110 (49) **CG:** 111 (60)	**IG^1:** 67.56 ± 4.99 **IG^2:** 67.46 ± 4.73 **CG:** 67.62 ± 5.35	**IG^1:** 98% **IG^2:** 95% **CG:** 94%	**IG^1:** 10.36 ± 3.27 **IG^2:** 9.92 ± 3.59 **CG:** 10.13 ± 3.47	**IG^1:** Tai Chi Chuan **IG^2:** Fitness walking **CG:** Usual care	**Intensity:** N/A **Duration:** 24-week **Training load:** 60-min, 3 times/week
**Lin et al.**[48] **2024** **2-arm RCT**	**Diagnosis:** aMCI **Instrument:** GDS, MoCA	N/A	**IG:** 21 (20, 23) **CG:** 21 (19.5, 23)	**IG:** 48 (26) **CG:** 48 (30)	**IG:** 67.38 ± 3.91 **CG:** 68.38 ±	**IG:** 96% **CG:** 96%	**IG:** 12 (9,12.75) **CG:** 11 (7.25, 12.75)	**IG:** Tai Chi intervention **CG:** Health education	**Intensity:** N/A **Duration:** 12-week **Training load:** 60-min, 5 times/week

					4.13				
**Yu et al.**[49] **2022** **3-arm RCT**	**Diagnosis:** MCI **Instrument:** MoCA	N/A	**IG^1:** 19.7 ± 1.5 **IG^2:** 19.3 ± 2.0 **CG:** 18.2 ± 3.8	**IG^1:** 10 (7) **IG^2:** 12 (8) **CG:** 12 (10)	**IG^1:** 67.3 ± 4.2 **IG^2:** 67.2 ± 6.8 **CG:** 67.6 ± 8.1	**IG^1:** 83% **IG^2:** 92% **CG:** 100%	**IG^1:** 11.8 ± 2.4 **IG^2:** 11.4 ± 3.8 **CG:** 10.9 ± 3.7	**IG^1:** Tai Chi intervention **IG^2:** Conventional exercise training (muscle-strengthening and aerobic exercises) **CG:** Control	**Intensity:** 13/20 RPE **Duration:** 24-week **Training load:** 60-min, 3 times/week
**Huang et al.**[50] **2019** **2-arm RCT**	**Diagnosis:** Mild dementia **Instrument:** DSM, CDR	**IG:** 20.73 ± 6.57 **CG:** 20.80 ± 5.16	**IG:** 13.08 ± 5.35 **CG:** 13.32 ± 4.56	**IG:** 40 (28) **CG:** 40 (26)	**IG:** 81.9 ± 6.0 **CG:** 81.9 ± 6.1	**IG:** 90% **CG:** 95%	N/A	**IG:** Tai Chi intervention **CG:** Routine treatments and personalized daily care	**Intensity:** N/A **Duration:** 40-week **Training load:** 20-min, 3 times/week
**Cheng et al.**[51,52] **2014** **3-arm RCT**	**Diagnosis:** Mild dementia **Instrument:** MMSE, CDR	**IG^1:** 18.7 ± 3.9 **IG^2:** 19.0 ± 3.2 **CG:** 18.9 ± 4.1	N/A	**IG^1:** 39 (25) **IG^2:** 36 (23) **CG:** 35 (23)	**IG^1:** 81.8 ± 7.4 **IG^2:** 81.9 ± 6.2 **CG:** 80.9 ± 7.2	**IG^1:** 93% **IG^2:** 97% **CG:** 92%	N/A	**IG^1:** Tai Chi intervention **IG^2:** Mahjong (136-tile version) **CG:** Handicraft (connected beads to create different shapes)	**Intensity:** N/A **Duration:** 12-week **Training load:** 60-min, 3 times/week
**Liu et al.**[53] **2018** **2-arm RCT**	**Diagnosis:** Dementia **Instrument:** MoCA	N/A	**IG:** 15.8 ± 5.37 **CG:** 11.8 ± 3.07	**IG:** 13 (8) **CG:** 13 (9)	**IG:** 79.8 ± 8.16 **CG:** 80.5 ± 6.94	**IG:** 85% **CG:** 85%	N/A	**IG:** 10-step simplified Tai-chi training (*caregiver involved*) **CG:** Recreational activities (watching movies and listening to music) and keep usual lifestyles and levels of physical activity	**Intensity:** N/A **Duration:** 16-week **Training load:** 60-min, 2 times/week
**Chan et al.**[54] **2016** **2-arm RCT**	**Diagnosis:** CI **Instrument:** MMSE	13-26	N/A	**IG:** 27 (27) **CG:** 25 (17)	**IG:** 78.4 ± 7.1 **CG:** 82.2 ± 6.7	**IG:** 81% **CG:** 60%	N/A	**IG:** Tai chi qigong **CG:** Maintain routine activities	**Intensity:** N/A **Duration:** 8-week **Training load:** 60-min, 2 times/week
**Nyman et al.**[55,56] **2019** **2-arm RCT**	**Diagnosis:** Dementia **Instrument:**	N/A	N/A	**IG:** 42 (18) **CG:** 43	**IG:** 77.9 ± 8.3	**IG:** 86% **CG:** 81%	N/A	**IG:** Tai Chi intervention **CG:** Usual care	**Intensity:** N/A **Duration:** 20-week

	M-ACE			(16)	**CG:** 78.2 ± 7.5				**Training load:** 45-min, 1 time/week
**Okuyan and Deveci[57] 2020 2-arm RCT**	**Diagnosis:** MCI **Instrument:** MMSE, MoCA, CDR	< 25	N/A	**IG:** 20 (7) **CG:** 22 (8)	74.21 ± 6.93	**IG:** 87% **CG:** 92%	N/A	**IG:** Tai Chi **CG:** Control	**Intensity:** N/A **Duration:** 12-week **Training load:** 35-40-min, 2 times/week
**Su et al.[58] 2021 2-arm RCT**	**Diagnosis:** Older adults with memory complaint **Instrument:** MoCA	N/A	**IG:** 26.97 ± 1.15 **CG:** 27.23 ± 1.14	**IG:** 32 (19) **CG:** 33 (17)	**IG:** 64.40 ± 6.57 **CG:** 65.37 ± 6.31	**IG:** 92% **CG:** 90%	N/A	**IG:** Baduanjin intervention **CG:** Active control (gymnastics practice and physical education class)	**Intensity:** N/A **Duration:** 12-week **Training load:** 60-min, 5 times/week
**Zheng et al.[59] 2020 2-arm RCT**	**Diagnosis:** Cognitive impairment due to stroke **Instrument:** MRI, DSM	N/A	**IG:** 21.21 ± 0.84 **CG:** 20.71 ± 0.51	**IG:** 24 (5) **CG:** 24 (2)	**IG:** 61.63 ± 9.21 **CG:** 62.75 ± 6.41	**IG:** 92% **CG:** 79%	**IG:** 11.04 ± 3.03 **CG:** 10.63 ± 2.34	**IG:** Baduanjin training **CG:** Control	**Intensity:** N/A **Duration:** 24-week **Training load:** 40-min, 3 times/week
**Zheng et al.[60–62] 2021 3-arm RCT**	**Diagnosis:** MCI **Instrument:** MoCA	N/A	**IG[1]:** 22.30 ± 2.40 **IG[2]:** 21.65 ± 2.35 **CG:** 20.83 ± 3.27	**IG[1]:** 23 (17) **IG[2]:** 23 (12) **CG:** 23 (17)	**IG[1]:** 65.79± 4.45 **IG[2]:** 64.88 ± 3.30 **CG:** 65.86 ± 5.28	**IG[1]:** 87% **IG[2]:** 87% **CG:** 87%	N/A	**IG[1]:** Baduanjin training and health education **IG[2]:** Brisk walking and health education **CG:** Non-exercise health education control (nutrition, health care for older individuals and cognitive decline with aging)	**Intensity:** N/A **Duration:** 24-week **Training load:** 60-min, 3 times/week
**Li et al.[63] 2022 2-arm RCT**	**Diagnosis:** MCI **Instrument:** MMSE	**IG:** 21.83 ± 2.22 **CG:** 21.56 ± 2.06	**IG:** 19.96 ± 1.92 **CG:** 20.61 ± 1.86	**IG:** 30 (15) **CG:** 30 (14)	**IG:** 57.18 ± 1.69 **CG:** 57.8 ± 1.54	**IG:** 100% **CG:** 100%	N/A	**IG:** Baduanjin exercise **CG:** Leisure walking	**Intensity:** N/A **Duration:** 12-week **Training load:** 45-min, 5 times/week
**Luo et al.[64] 2022 2-arm RCT**	**Diagnosis:** MCI **Instrument:** MoCA	N/A	**IG:** 22.21 ± 0.46 **CG:** 23.33 ± 0.37	**IG:** 25 (21) **CG:** 25 (17)	**IG:** 66.71 ± 0.95 **CG:** 65.08 ±	**IG:** 96% **CG:** 96%	**IG:** 9.83 ± 0.53 **CG:** 10.67 ± 0.44	**IG:** Wuqinxi intervention **CG:** Non-exercise control	**Intensity:** N/A **Duration:** 40-week **Training load:** 60-min, 1 time/week

					0.70				
**Chang et al.[65]**<br>**2024**<br>**3-arm RCT**	**Diagnosis:**<br>MCI due to PD<br>**Instrument:**<br>Diagnosed by a specialist	N/A	**IG^1:** 26.75 ± 3.53<br>**IG^2:** 27.36 ± 3.07<br>**CG:** 26.92 ± 2.75	**IG^1:** 16 (9)<br>**IG^2:** 14 (9)<br>**CG:** 13 (7)	**IG^1:** 66.31 ± 6.54<br>**IG^2:** 64.43 ± 7.37<br>**CG:** 63.15 ± 7.95	**IG^1:** 80%<br>**IG^2:** 78%<br>**CG:** 72%	N/A	**IG^1:** Tai Chi Chuan<br>**IG^2:** Aerobic exercise<br>**CG:** Normal daily PA control	**Intensity:**<br>IG^1: N/A<br>IG^2: 70%–75% HRR<br>**Duration:** 12-week<br>**Training load:**<br>IG^1: 60-min, 2 times/week<br>IG^2: 30-mins, 3 times/week
**Gao et al.[66]**<br>**2024**<br>**3-arm RCT**	**Diagnosis:**<br>Mild to moderate cognitive impairment<br>**Instrument:**<br>MMSE	**IG^1:** 17.2 ± 3.2<br>**IG^2:** 16.1 ± 3.0<br>**CG:** 17.2 ± 3.7	N/A	**IG^1:** 27 (18)<br>**IG^2:** 27 (23)<br>**CG:** 27 (19)	**IG^1:** 78.6 ± 7.0<br>**IG^2:** 83.3 ± 6.2<br>**CG:** 80.9 ± 8.4	**IG^1:** 96%<br>**IG^2:** 89%<br>**CG:** 93%	N/A	**IG^1:** Tai Chi<br>**IG^2:** Conventional exercise<br>**CG:** Waitlist control	**Intensity:** N/A<br>**Duration:** 12-week<br>**Training load:** 50-min, 3 times/week
**Hsu et al.[67]**<br>**2021**<br>**2-arm RCT**	**Diagnosis:**<br>MCI<br>**Instrument:**<br>MMSE	**IG:** 22.83 ± 3.53<br>**CG:** 22.82 ± 3.55	N/A	**IG:** 41 (23)<br>**CG:** 39 (28)	**IG:** 72.83 ± 6.97<br>**CG:** 74.28 ± 7.80	**IG:** 89%<br>**CG:** 85%	N/A	**IG:** Chan-Chuang Qigong<br>**CG:** Control	**Intensity:** N/A<br>**Duration:** 12-week<br>**Training load:** 30-min, 3 times/week
**Aerobic exercise (AE)**									
**Tomoto et al.[67]**<br>**2021**<br>**2-arm RCT**	**Diagnosis:**<br>aMCI<br>**Instrument:**<br>Petersen criteria, CDR, MMSE	**IG:** 29.0 ± 1.5<br>**CG:** 28.8 ± 1.4	N/A	**IG:** 22 (12)<br>**CG:** 30 (16)	**IG:** 64.8 ± 6.4<br>**CG:** 66.1 ± 6.8	N/A	**IG:** 16.2 ± 2.2<br>**CG:** 15.9 ± 2.1	**IG:** Aerobic exercise training<br>**CG:** Stretching and toning	**Intensity:** 75%–90% of HRmax<br>**Duration:** 48-week<br>**Training load:** 30–40-min, 3–5 times/week
**Shimada et al.[68]**<br>**2018**<br>**2-arm RCT**	**Diagnosis:**<br>Older adults<br>**Instrument:**<br>MMSE	**IG:** 28.4 ± 1.8<br>**CG:** 28.7 ± 1.4	N/A	**IG:** 53 (25)<br>**CG:** 53 (24)	**IG:** 70.1 ± 4.0<br>**CG:** 70.7 ±	**IG:** 96%<br>**CG:** 89%	**IG:** 12.8 ± 2.8<br>**CG:** 13.7 ± 2.6	**IG:** Golf program<br>**CG:** Control health education (information regarding exercise and healthy diets)	**Intensity:** N/A<br>**Duration:** 24-week<br>**Training load:** 90–120-min, 1

					4.7				time/week
**Donnezan et al.**[69]<br>**2018**<br>**4-arm RCT**	**Diagnosis:**<br>MCI<br>**Instrument:**<br>Diagnosed by a neuropsychologist	**IG^1:** 28.2 ± 0.43<br>**IG^2:** 27.3 ± 0.42<br>**IG^3:** 28.1 ± 0.36<br>**CG:** 27.3 ± 0.5	N/A	**IG^1:** 18<br>**IG^2:** 16<br>**IG^3:** 21<br>**CG:** 14	**IG^1:** 77.1 ± 1.44<br>**IG^2:** 76.3 ± 1.5<br>**IG^3:** 75.2 ± 1.3<br>**CG:** 79.2 ± 4	**IG^1:** 86%<br>**IG^2:** 84%<br>**IG^3:** 100%<br>**CG:** 93%	**IG^1:** 6.1 ± 0.34<br>**IG^2:** 5.5 ± 0.36<br>**IG^3:** 5.9 ± 0.31<br>**CG:** 5.8 ± 0.4	**IG^1:** Physical training<br>**IG^2:** Cognitive training (game stimulation by using words, numbers, colors, shapes, and logical exercise)<br>**IG^3:** Physical combine cognitive training (two training simultaneously)<br>**CG:** No-intervention control (usual lifestyle)	**Intensity:** 60% of HRmax<br>**Duration:** 12-week<br>**Training load:** 60-min, 2 times/week
**Baker et al.**[70]<br>**2010**<br>**2-arm RCT**	**Diagnosis:** aMCI<br>**Instrument:**<br>Petersen criteria	**IG:** F 28.4 ± 1.7, M 25.6 ± 2.4<br>**CG:** F 28.6 ± 1.7, M 27.2 ± 1.8	N/A	**IG:** 19 (10)<br>**CG:** 10 (5)	**IG:** F 65.3 ± 9.4, M 70.9 ± 6.7<br>**CG:** F 74.6 ± 11.1, M 70.6 ± 6.1	**IG:** 83%<br>**CG:** 100%	N/A	**IG:** Aerobic training<br>**CG:** Stretching and balance exercise	**Intensity:** 75%–85% of HRR<br>**Duration:** 24-week<br>**Training load:** 45–60-min, 4 times/week
**Ihle-Hansen et al.**[71,72]<br>**2019**<br>**2-arm RCT**	**Diagnosis:**<br>MCI due to stroke<br>**Instrument:**<br>MMSE	**IG:** 27.9 ± 0.2<br>**CG:** 28.0 ± 0.2	N/A	**IG:** 177 (78)<br>**CG:** 185 (65)	**IG:** 71.4 ± 11.3<br>**CG:** 72.0 ± 11.3	**IG:** 95%<br>**CG:** 95%	N/A	**IG:** Physical exercise intervention (tailored training program based on the participants' preferences and goals)<br>**CG:** Usual care	**Intensity:** 15–17/20 RPE<br>**Duration:** 72-week<br>**Training load:** 30-min, 7 times/week
**Nagamatsu et al.**[73,74]<br>**2013**<br>**3-arm RCT**	**Diagnosis:**<br>MCI<br>**Instrument:**<br>MMSE, MoCA	**IG^1:** 27.4 ± 1.5<br>**IG^2:** 27.0 ± 1.8<br>**CG:** 27.1 ± 1.7	**IG^1:** 22.2 ± 2.8<br>**IG^2:** 21.4 ± 3.4<br>**CG:** 22.5 ± 2.8	**IG^1:** 30 (30)<br>**IG^2:** 28 (28)<br>**CG:** 28 (28)	**IG^1:** 75.6 ± 3.6<br>**IG^2:** 73.9 ± 3.4<br>**CG:** 75.1 ± 3.6	**IG^1:** 80%<br>**IG^2:** 93%<br>**CG:** 96%	N/A	**IG^1:** Aerobic training<br>**IG^2:** Resistance training<br>**CG:** Balance and tone (stretching, range of motion, balance exercise, functional and relaxation techniques)	**Intensity:**<br>IG^1: 70%–80 % HRR<br>IG^2: 7 RM<br>**Duration:** 24-week<br>**Training load:** 60-min, 2 times/week
**Tsai et al.**[75]<br>**2019**<br>**3-arm RCT**	**Diagnosis:**<br>aMCI<br>**Instrument:**<br>MMSE	**IG^1:** 27.16 ± 1.26<br>**IG^2:** 26.56 ± 1.34	N/A	**IG^1:** 19 (14)<br>**IG^2:** 18 (11)	**IG^1:** 66.00 ± 7.68<br>**IG^2:**	**IG^1:** 86%<br>**IG^2:** 82%<br>**CG:** 82%	**IG^1:** 11.63 ± 3.37<br>**IG^2:** 11.72 ± 3.51	**IG^1:** Aerobic exercise<br>**IG^2:** Resistance exercise<br>**CG:** Control (social interactions, stretching and	**Intensity:**<br>IG^1: 70%–75% of HRR<br>IG^2: 75% of 1RM

		**CG:** 27.00 ± 1.65		**CG:** 18 (13)	65.44 ± 6.76<br>**CG:** 65.17 ± 7.00		**CG:** 11.83 ± 2.98	balance exercise)	**Duration:** 16-week<br>**Training load:** 40-min, 3 times/week
**Hsu et al.**[76–80]<br>**2018**<br>**2-arm RCT**	**Diagnosis:**<br>Vascular CI<br>**Instrument:**<br>MMSE, MoCA	**IG:** 26.8 ± 2.3<br>**CG:** 27.7 ± 1.5	**IG:** 22.2 ± 2.4<br>**CG:** 24.1 ± 2.1	**IG:** 10 (6)<br>**CG:** 11 (7)	**IG:** 71.7 ± 8.8<br>**CG:** 72.3 ± 8.8	**IG:** 53%<br>**CG:** 58%	N/A	**IG:** Aerobic walking training<br>**CG:** Usual care plus educational materials about VCI and health diet (same materials provided to intervention group)	**Intensity:** 60%–70% of HRR<br>**Duration:** 24-week<br>**Training load:** 60-min, 3 times/week
**Song et al.**[81]<br>**2019**<br>**2-arm RCT**	**Diagnosis:**<br>MCI<br>**Instrument:**<br>MoCA	N/A	**IG:** 22.03 ± 1.97<br>**CG:** 22.10 ± 1.92	**IG:** 60 (48)<br>**CG:** 60 (42)	**IG:** 76.22 ± 5.76<br>**CG:** 75.33 ± 6.78	**IG:** 80%<br>**CG:** 78%	N/A	**IG:** Aerobic stepping exercise program<br>**CG:** Health education program (no information relating to brain health and physical exercise)	**Intensity:** 12–14/20 RPE<br>**Duration:** 16-week<br>**Training load:** 60-min, 3 times/week
**Stuckenschneider et al.**[82]<br>**2021**<br>**3-arm RCT**	**Diagnosis:**<br>aMCI<br>**Instrument:**<br>MoCA	N/A	**IG^1:** 22.6 ± 2.5<br>**IG^2:** 22.9 ± 2.2<br>**CG:** 22.4 ± 2.1	**IG^1:** 60 (28)<br>**IG^2:** 65 (23)<br>**CG:** 58 (35)	**IG^1:** 70.6 ± 6.1<br>**IG^2:** 72.3 ± 6.6<br>**CG:** 71.6 ± 6.9	**IG^1:** 91%<br>**IG^2:** 91%<br>**CG:** 90%	N/A	**IG^1:** Aerobic exercise<br>**IG^2:** Non-aerobic stretching and toing<br>**CG:** Usual care	**Intensity:**<br>IG^1: 13/20 RPE<br>IG^2: < 10/20 RPE<br>**Duration:** 48-week<br>**Training load:** 45-min, 3 times/week
**Rojasavastera et al.**[83]<br>**2020**<br>**3-arm RCT**	**Diagnosis:**<br>aMCI<br>**Instrument:**<br>MoCA	N/A	**IG^1:** 22.18 ± 1.47<br>**IG^2:** 22.64 ± 1.36<br>**CG:** 22.18 ± 1.94	**IG^1:** 11 (9)<br>**IG^2:** 11 (8)<br>**CG:** 11 (8)	**IG^1:** 67.64 ± 4.64<br>**IG^2:** 67.50 ± 5.60<br>**CG:** 65.71 ± 2.45	**IG^1:** 85%<br>**IG^2:** 85%<br>**CG:** 85%	**IG^1:** 13.50 ± 3.25<br>**IG^2:** 13.82 ± 4.35<br>**CG:** 12.73 ± 4.43	**IG^1:** Action observation with gait training<br>**IG^2:** Gait training<br>**CG:** Control	**Intensity:** N/A<br>**Duration:** 8-week<br>**Training load:** 65-min, 2–3 times/week
**Makino et al.**[84]<br>**2021**<br>**4-arm RCT**	**Diagnosis:**<br>Older adults with memory complaints<br>**Instrument:**<br>MMSE	**IG^1:** 26.42 ± 2.49<br>**IG^2:** 26.07 ± 2.46<br>**IG^3:** 26.37 ± 2.66	N/A	**IG^1:** 104 (49)<br>**IG^2:** 102 (49)<br>**IG^3:** 104 (43)	**IG^1:** 72.25 ± 4.56<br>**IG^2:** 72.33 ± 4.77	**IG^1:** 88%<br>**IG^2:** 93%<br>**IG^3:** 94%<br>**CG:** 89%	**IG^1:** 11.48 ± 2.47<br>**IG^2:** 11.47 ± 2.42<br>**IG^3:** 11.56 ± 2.14	**IG^1:** Aerobic exercise training<br>**IG^2:** Resistance exercise training<br>**IG^3:** Combined exercise training (combined **IG^1** and **IG$^{2)}$**	**Intensity:**<br>**IG^1:** 15/20 RPE<br>**IG^2:** 70% of HRR<br>**Duration:** 26-week<br>**Training load:** 60-

		**CG:** 26.30 ± 2.72		**CG:** 105 (54)	**IG³:** 72.61 ± 4.52<br>**CG:** 72.10 ± 4.61		**CG:** 11.30 ± 2.62	**CG:** Control (education class provided information about health promotion of aging, heathy diet, and prevention of cerebrovascular disease)	min, 2 times/week
**Brydges et al.[85] 2020 3-arm RCT**	**Diagnosis:** MCI<br>**Instrument:** MMSE, MoCA	**IG¹:** 25.96 ± 5.78<br>**IG²:** 27.49 ± 1.42<br>**CG:** 27.03 ± 1.66	**IG¹:** 21.52 ± 3.33<br>**IG²:** 22.81 ± 2.47<br>**CG:** 22.88 ± 2.46	**IG¹:** 25 (25)<br>**IG²:** 27 (27)<br>**CG:** 25 (25)	**IG¹:** 74.08 ± 2.90<br>**IG²:** 74.93 ± 3.06<br>**CG:** 74.36 ± 3.09	N/A	N/A	**IG¹:** Resistance exercise<br>**IG²:** Aerobic exercise<br>**CG:** Balance and tone (traveling to training centers and social interaction)	**Intensity:** 70%–80% of HRmax, 13–15/20 RPE<br>**Duration:** 24-week<br>**Training load:** 60-min, 2 times/week
**Morris et al.[86] 2017 2-arm RCT**	**Diagnosis:** AD<br>**Instrument:** CDR	**IG:** 25.8 ± 3.3<br>**CG:** 25.0 ± 3.2	N/A	**IG:** 39 (18)<br>**CG:** 37 (21)	**IG:** 74.4 ± 6.7<br>**CG:** 71.4 ± 8.4	**IG:** 87%<br>**CG:** 92%	**IG:** 15.5 ± 3.3<br>**CG:** 16.1 ± 3.1	**IG:** Aerobic exercise<br>**CG:** Non-aerobic stretching and toning exercise	**Intensity:** 60%–75% of HRR<br>**Duration:** 26-week<br>**Training load:** 150-min/week, 3–5 sessions
**Wei and Ji[87] 2014 2-arm RCT**	**Diagnosis:** MCI<br>**Instrument:** MMSE, GDS	**IG:** 24.33 ± 1.65<br>**CG:** 25.00 ± 1.29	N/A	**IG:** 30 (9)<br>**CG:** 30 (11)	**IG:** 66.73 ± 5.48<br>**CG:** 65.27 ± 4.63	N/A	N/A	**IG:** Handball training program aerobic exercise<br>**CG:** Maintained the original life entertainment	**Intensity:** 60% of HRmax<br>**Duration:** 24-week<br>**Training load:** 30-min, 5 times/week
**Damirchi et al.[88] 2017 4-arm RCT**	**Diagnosis:** MCI<br>**Instrument:** GDS	**IG¹:** 23.18 ± 2.18<br>**IG²:** 23.81 ± 2.04<br>**IG³:** 23.30 ± 1.84<br>**CG:** 23.44 ± 2.06	N/A	**IG¹:** 11 (11)<br>**IG²:** 11 (11)<br>**IG³:** 13 (13)<br>**CG:** 9 (9)	**IG¹:** 68.81 ± 3.68<br>**IG²:** 67.90 ± 3.75<br>**IG³:** 67.76 ± 4.69<br>**CG:** 69.11 ± 4.93	**IG¹:** 73%<br>**IG²:** 73%<br>**IG³:** 87%<br>**CG:** 100%	**IG¹:** 3.45 ± 1.03<br>**IG²:** 3.54 ± 1.29<br>**IG³:** 2.76 ± 0.92<br>**CG:** 3.22 ± 1.20	**IG¹:** Physical training<br>**IG²:** Mental training (4 different games)<br>**IG³:** Combined IG¹ and IG² training<br>**CG:** Control	**Intensity:** 75% of HRmax, 13–15/20 RPE<br>**Duration:** 8-week<br>**Training load:** 30-60-min, 3 times/week
**Nakatsuka et al.[89]**	**Diagnosis:**	**IG¹:** 23.5	N/A	**IG¹:** 38	**IG¹:**	**IG¹:** 63%	**IG¹:** 8.8 ±	**IG¹:** Physical activities	**Intensity:** 13–

**2015**<br>**3-arm RCT**	Older adults<br>**Instrument:** MMSE, CDR	± 2.4<br>**IG^2:** 22.2 ± 3.2<br>**CG:** 25.1 ± 2.7		(25)<br>**IG^2:** 45 (21)<br>**CG:** 44 (21)	81.3 ± 3.8<br>**IG^2:** 82.2 ± 3.8<br>**CG:** 81.2 ± 4.0	**IG^2:** 71%<br>**CG:** 89%	1.5<br>**IG^2:** 8.1 ± 1.1<br>**CG:** 9.4 ± 1.9	**IG^2:** Cognitive interventions<br>**CG:** Reminiscence approach	15/20 RPE<br>**Duration:** 12-week<br>**Training load:** 60-min, 1 time/week
**Kohanpour et al.[90]**<br>**2017**<br>**4-arm RCT**	**Diagnosis:** MCI<br>**Instrument:** MMSE	**IG^1:** 22.70 ± 1.63<br>**IG^2:** 22.50 ± 1.35<br>**IG^3:** 22.80 ± 1.75<br>**CG:** 24.30 ± 0.94	N/A	**IG^1:** 10 (0)<br>**IG^2:** 10 (0)<br>**IG^3:** 10 (0)<br>**CG:** 10 (0)	67.85 ± 3.89	N/A	N/A	**IG^1:** Training (running)<br>**IG^2:** Training + Lavender<br>**IG^3:** Lavender<br>**CG:** Placebo	**Intensity:** 75%–85% of HRR<br>**Duration:** 12-week<br>**Training load:** 39-min, 3 times/week
**Karthikeyan T[91]**<br>**2020**<br>**2-arm RCT**	**Diagnosis:** MCI<br>**Instrument:** Diagnosed by a neuropsychiatrist	N/A	**IG:** 20.73 ± 1.70<br>**CG:** 20.93 ± 1.43	**IG:** 15 (6)<br>**CG:** 15 (7)	**IG:** 64.86 ± 2.87<br>**CG:** 64.40 ± 2.66	N/A	N/A	**IG:** Aerobic brisk walking<br>**CG:** Home-based exercise (gentle active range of motion exercise and stretching exercise)	**Intensity:** 60%–70% of HRmax, 11–14/20 RPE<br>**Duration:** 8-week<br>**Training load:** 40–50-min, 7 times/week
**Krootnark et al.[92]**<br>**2024**<br>**3-arm RCT**	**Diagnosis:** MCI<br>**Instrument:** MoCA	N/A	**IG^1:** 20.17 ± 2.09<br>**IG^2:** 19.60 ± 1.83<br>**CG:** 20.40 ± 1.87	**IG^1:** 30 (24)<br>**IG^2:** 30 (23)<br>**CG:** 30 (24)	**IG^1:** 68.60 ± 4.86<br>**IG^2:** 68.70 ± 4.72<br>**CG:** 69.70 ± 5.55	**IG^1:** 100%<br>**IG^2:** 100%<br>**CG:** 100%	N/A	**IG^1:** Home based aerobic exercise<br>**IG^2:** Home based resistance training<br>**CG:** Usual life	**Intensity:** 9–13/20 RPE<br>**Duration:** 12-week<br>**Training load:** 35-min, 5 times/week
**Liu et al.[93]**<br>**2020**<br>**2-arm RCT**	**Diagnosis:** Dementia<br>**Instrument:** MMSE	**IG:** 23.87 ± 4.65<br>**CG:** 22.7 ± 4.28	**IG:** 19.78 ± 4.69<br>**CG:** 19.12 ± 3.79	**IG:** 31 (5)<br>**CG:** 30 (6)	**IG:** 84.68 ± 6.74<br>**CG:** 86.77 ± 6.99	**IG:** 91%<br>**CG:** 86%	N/A	**IG:** Aerobic training<br>**CG:** Strength training (isotonic weight training machines)	**Intensity:** 5–6/10 RPE<br>**Duration:** 4-week<br>**Training load:** 30-min, 5 times/week
**Dillon and Prapavessis[94]**<br>**2021**	**Diagnosis:** Mild to moderate CI<br>**Instrument:**	**IG:** 22.4 ± 1.4<br>**CG:** 20.8	N/A	**IG:** 14 (7)<br>**CG:** 11	**IG:** 87.6 ± 5.5	**IG:** 78%<br>**CG:** 92%	N/A	**IG:** Walking intervention<br>**CG:** Control	**Intensity:** N/A<br>**Duration:** 10-week

**2-arm RCT**	MMSE	± 2.8		(6)	**CG:**<br>85.45 ±<br>5.0				**Training load:** 30-min, 7 times/week
**Yang et al.[95]**<br>**2015**<br>**2-arm RCT**	**Diagnosis:**<br>AD<br>**Instrument:**<br>MMSE, NINDS-IAREN	**IG:** 21.33 ± 2.24<br>**CG:** 20.00 ± 3.50	N/A	**IG:** 25 (15)<br>**CG:** 25 (18)	**IG:**<br>72.00 ±<br>6.69<br>**CG:**<br>71.92 ±<br>7.28	N/A	N/A	**IG:** Cycling training<br>**CG:** Health education	**Intensity:** 70% of HRmax<br>**Duration:** 12-week<br>**Training load:** 40-min, 3 times/week
**Choi et al.[96,97]**<br>**2018**<br>**2-arm RCT**	**Diagnosis:**<br>MCI<br>**Instrument:**<br>MoCA	N/A	**IG:** 21.66 ± 3.24<br>**CG:** 20.76 ± 3.02	**IG:** 30 (24)<br>**CG:** 30 (25)	**IG:**<br>74.90 ±<br>5.10<br>**CG:**<br>74.23 ±<br>4.38	**IG:** 97%<br>**CG:** 90%	N/A	**IG:** Ground Kayak Paddling exercise (with *music*)<br>**CG:** Home exercise program (William exercise and curl-ups, sideways leg lifts, prone leg lifts, supine leg lifts, and prone trunk hyperextensions)	**Intensity:** N/A<br>**Duration:** 6-week<br>**Training load:** 60-min, 2 times/week
**Yu et al.[98]**<br>**2022**<br>**5-arm RCT**	**Diagnosis:**<br>MCI<br>**Instrument:**<br>Mayo Clinic diagnostic criteria	N/A	**IG¹:** 19.7 ± 1.9<br>**IG²:** 18.9 ± 1.9<br>**IG³:** 20.1 ± 1.7<br>**IG⁴:** 19.6 ± 2.5<br>**CG:** 19.9 ± 3.5	**IG¹:** 7 (6)<br>**IG²:** 7 (6)<br>**IG³:** 8 (7)<br>**IG⁴:** 8 (8)<br>**CG:** 7 (6)	**IG¹:**<br>63.5 ±<br>7.0<br>**IG²:**<br>63.5 ±<br>5.7<br>**IG³:**<br>63.4 ±<br>5.2<br>**IG⁴:**<br>63.3 ±<br>5.1<br>**CG:**<br>63.7 ±<br>4.7	**IG¹:** 70%<br>**IG²:** 70%<br>**IG³:** 80%<br>**IG⁴:** 80%<br>**CG:** 70%	N/A	**IG¹:** M1-150-min moderate-intensity walking<br>**IG²:** M3-50-min moderate-intensity walking<br>**IG³:** V1-75-min vigorous-intensity walking<br>**IG⁴:** V3-25-min vigorous-intensity walking<br>**CG:** Stretching exercise intervention	**Intensity:** N/A<br>**Duration:** 12-week<br>**Training load:** 1 or 3 times/week
**Fischbacher et al.[99]**<br>**2020**<br>**3-arm RCT**	**Diagnosis:**<br>MCI or early dementia<br>**Instrument:**<br>MoCA	N/A	**IG¹:** 19.6 ± 2.9<br>**IG²:** 22.6 ± 3.0<br>**CG:** 20.0 ± 1.9	**IG¹:** 7 (4)<br>**IG²:** 5 (3)<br>**CG:** 6 (4)	**IG¹:**<br>75.9 ±<br>5.1<br>**IG²:**<br>73.8 ±<br>3.6<br>**CG:**<br>76.7 ±<br>4.8	**IG¹:** 100%<br>**IG²:** 80%<br>**CG:** 100%	N/A	**IG¹:** Dalcroze eurhythmics classes<br>**IG²:** Simple home exercise program<br>**CG:** No-exercise control	**Intensity:** N/A<br>**Duration:** 48-week<br>**Training load:** 60-min,<br>IG¹: 1 time/week<br>IG²: 3 times/week
**Khattak et al.[100]**	**Diagnosis:**	**IG:** 20.28	**IG:** 21.17	**IG:** 29	62.49 ±	N/A	N/A	**IG:** Aerobic walk on treadmill	**Intensity:** 11–

**2022**<br>**2-arm RCT**	MCI<br>**Instrument:**<br>MMSE	± 1.06<br>**CG:** 20.87 ± 1.19	± 1.39<br>**CG:** 21.13 ± 1.63	**CG:** 30	1.82			**CG:** Gentle active motion exercises and stretching exercise	14/20 RPE<br>**Duration:** 6-week<br>**Training load:** 40–50-min, 5 times/week
**Varela et al.[101]**<br>**2011**<br>**3-arm RCT**	**Diagnosis:**<br>MCI<br>**Instrument:**<br>Spanish society of geriatrics and gerontology	**IG^1:** 19.86 ± 5.12<br>**IG^2:** 20.81 ± 4.69<br>**CG:** 21.08 ± 3.32	N/A	**IG^1:** 17<br>**IG^2:** 16<br>**CG:** 15	78.3 ± 9.5	**IG^1:** 63%<br>**IG^2:** 62%<br>**CG:** 100%	N/A	**IG^1:** Aerobic exercise<br>**IG^2:** Aerobic exercise<br>**CG:** Recreational activities (playing cards reading newspapers, handcrafts)	**Intensity:**<br>IG^1: 40% of HRmax<br>IG^2: 60% of HRmax<br>**Duration:** 12-week<br>**Training load:** 30-min, 3 times/week
**Miu et al.[102]**<br>**2008**<br>**2-arm RCT**	**Diagnosis:**<br>Mild to moderate dementia<br>**Instrument:**<br>DSM, MMSE	**IG:** 19.53 ± 4.59<br>**CG:** 19.69 ± 4.00	N/A	**IG:** 36 (15)<br>**CG:** 49 (31)	**IG:** 75 ± 7<br>**CG:** 78 ± 6	**IG:** 97%<br>**CG:** 98%	N/A	**IG:** Aerobic exercise training<br>**CG:** Usual care	**Intensity:** N/A<br>**Duration:** 12-week<br>**Training load:** 45–60-min, 2 times/week
**Arcoverde et al.[103]**<br>**2013**<br>**2-arm RCT**	**Diagnosis:** AD and Mixed dementia<br>**Instrument:**<br>NINCDS-ADRDA, NINDS-IAREN, MMSE, CDR	**IG:** 20.4 ± 2.7<br>**CG:** 19.9 ± 3.4	N/A	**IG:** 10 (6)<br>**CG:** 10 (5)	**IG:** 78.5 ± 17.2<br>**CG:** 79 ± 7.5	**IG:** 100%<br>**CG:** 100%	**IG:** 8.7 ± 4.7<br>**CG:** 8.4 ± 4.0	**IG:** Treadmill walking<br>**CG:** Maintained only clinical and pharmacological treatment	**Intensity:** 40–60% of VO_2max<br>**Duration:** 16-week<br>**Training load:** 30-min, 2 times/week
**Angiolillo et al.[104]**<br>**2023**<br>**2-arm RCT**	**Diagnosis:**<br>Mild to moderate AD<br>**Instrument:**<br>NIA-AA, MMSE, CDR	**IG:** 20.24 ± 1.99<br>**CG:** 17.49 ± 3.89	N/A	**IG:** 9 (6)<br>**CG:** 13 (8)	**IG:** 78.89 ± 6.68<br>**CG:** 78.92 ± 8.04	**IG:** 60%<br>**CG:** 87%	**IG:** 7.89 ± 5.49<br>**CG:** 8.31 ± 5.17	**IG:** Aerobic exercise<br>**CG:** Both groups underwent cognitive re-education, music therapy, and motor, proprioceptive and postural rehabilitation	**Intensity:**<br>Personal perception of maximum exertion<br>**Duration:** 24-week<br>**Training load:** 60-min, 2 times/week
**Enette et al.[105]**<br>**2020**<br>**3-arm RCT**	**Diagnosis:**<br>Mild to moderate AD<br>**Instrument:**<br>DSM, MMSE	**IG^1:** 18 ± 5<br>**IG^2:** 18 ± 2<br>**CG:** 21 ± 6	N/A	**IG^1:** 14 (11)<br>**IG^2:** 17 (11)<br>**CG:** 21 (10)	**IG^1:** 74 ± 15<br>**IG^2:** 79 ± 7<br>**CG:** 79 ± 9	**IG^1:** 88%<br>**IG^2:** 100%<br>**CG:** 100%	**IG^1:** 10 ± 3<br>**IG^2:** 7<br>**CG:** 7 ± 3	**IG^1:** Continuous aerobic training<br>**IG^2:** Interval aerobic training<br>**CG:** Interactive information sessions	**Intensity:**<br>IG^1: 70% of HRmax<br>IG^2: 80% combined 60% of HRmax<br>**Duration:** 9-week

									**Training load:** 30-min, 2 times/week
**Phoemsapthawee et al.[106] 2016 2-arm RCT**	**Diagnosis:** MCI **Instrument:** MMSE	**IG:** 18.8 ± 4.0 **CG:** 19.5 ± 4.7	N/A	**IG:** 12 (12) **CG:** 12 (12)	65-87	N/A	N/A	**IG:** Arm swing exercise **CG:** Control	**Intensity:** 23% of VO_2peak **Duration:** 12-week **Training load:** 30-min, 5 times/week
**Eggermont et al.[107] 2009 2-arm RCT**	**Diagnosis:** Moderate dementia **Instrument:** MMSE	17.7		97 (79)	85.4	N/A	N/A	**IG:** Walking **CG:** Social visit	**Intensity:** N/A **Duration:** 6-week **Training load:** 30-min, 5 times/week
**Lowery et al.[108] 2013 2-arm RCT**	**Diagnosis:** Dementia **Instrument:** ICD-10, NPI	**IG:** 16.3 ± 7.4 **CG:** 14.9 ± 8.7	N/A	**IG:** 67 (35) **CG:** 64 (39)	**IG:** 79 ± 6.8 **CG:** 78 ± 7.4	**IG:** 88% **CG:** 89%	N/A	**IG:** Individually tailored progressively walking (*caregiver involved*) **CG:** Control	**Intensity:** 12–14/20 RPE **Duration:** 12-week **Training load:** 20-30-min, 5 times/week
**Guzel et al.[109] 2024 3-arm RCT**	**Diagnosis:** Dementia **Instrument:** Patients' records	**IG^1:** 13.10 ± 2.72 **IG^2:** 14.63 ± 3.10 **IG^3:** 12.10 ± 3.72	N/A	**IG^1:** 10 (10) **IG^2:** 11 (10) **IG^3:** 10 (7)	**IG^1:** 86.6 ± 3.8 **IG^2:** 82.3 ± 6.7 **IG^3:** 82.3 ± 7.8	**IG^1:** 83% **IG^2:** 85% **IG^3:** 77%	N/A	**IG^1:** Aerobic exercise **IG^2:** Combined aerobic and balance exercise **IG^3:** Balance exercise	**Intensity:** N/A **Duration:** 6-week **Training load:** 40-min, 2 times/week
**Venturelli et al.[110] 2011 2-arm RCT**	**Diagnosis:** AD **Instrument:** MMSE	**IG:** 13 ± 2 **CG:** 12 ± 2	N/A	**IG:** 12 **CG:** 12	**IG:** 83 ± 6 **CG:** 85 ± 5	**IG:** 92% **CG:** 83%	N/A	**IG:** Moderate walking (*caregivers involved*) **CG:** Participated in the daily activities like bingo, patchwork sewing, and music therapy	**Intensity:** Moderate **Duration:** 24-week **Training load:** 30-min, 4 times/week
**Scherder et al.[111] 2005 3-arm RCT**	**Diagnosis:** MCI **Instrument:** MMSE	**IG^1:** 9.73 ± 1.94 **IG^2:** 9.23 ± 1.30 **CG:** 9.87 ± 1.41	N/A	**IG^1:** 15 (13) **IG^2:** 13 (11) **CG:** 15 (14)	**IG^1:** 84 ± 6.38 **IG^2:** 89 ± 2.40 **CG:** 86 ± 5.50	N/A	N/A	**IG^1:** Walking **IG^2:** Hand and face exercise (bending and stretching the fingers, producing seven different facial expressions) **CG:** Social visits or continued normal social activities	**Intensity:** N/A **Duration:** 6-week **Training load:** 30-min, 3 times/week

**Amjad et al.**[112] **2018** **2-arm RCT**	**Diagnosis:** MCI **Instrument:** Diagnosed by an expert, MMSE, MoCA	< 25	< 25	**IG:** 21 (10) **CG:** 19 (9)	**IG:** 58.23 ± 2.31 **CG:** 59.56 ± 2.65	**IG:** 81% **CG:** 84%	N/A	**IG:** Aerobic exercise treatment **CG:** No-aerobic exercise control	**Intensity:** 60%–80% of HRmax **Duration:** 6-week **Training load:** 60-min, 3 times/week
**Abbas et al.**[113] **2023** **3-arm RCT**	**Diagnosis:** Moderate to severe dementia **Instrument:** MMSE	< 20	N/A	**IG^1:** 20 (10) **IG^2:** 20 (10) **IG^3:** 20 (10)	**IG^1:** 79 ± 6.67 **IG^2:** 81.55 ± 7.42 **IG^3:** 78.4 ± 6.21	N/A	N/A	**IG^1:** Motorized cycle ergometer training **IG^2:** High-intensity functional exercise training **IG^3:** Combine both IG^1 and IG^2	**Intensity:** IG^2: 13–15RM **Duration:** 12-week **Training load:** 50-min, 3 times/week
**Abd El-Kader and AI-Jiffri**[114] **2016** **2-arm RCT**	**Diagnosis:** AD **Instrument:** N/A	N/A	N/A	**IG:** 20 (6) **CG:** 20 (5)	**IG:** 68.94 ± 5.76 **CG:** 69.13 ± 6.12	**IG:** 92% **CG:** 89%	N/A	**IG:** Treadmill aerobic exercise **CG:** Control	**Intensity:** 60%–70% of HRmax **Duration:** 8-week **Training load:** 25–45-min, 3 times/week
**Law et al.**[115,116] **2021** **4-arm RCT**	**Diagnosis:** MCI **Instrument:** N/A	N/A	N/A	**IG^1:** 37 (21) **IG^2:** 34 (23) **IG^3:** 38 (25) **CG:** 36 (24)	**IG^1:** 77.35 ± 6.66 **IG^2:** 73.21 ± 7.27 **IG^3:** 76.32 ± 7.21 **CG:** 74.14 ± 7.53	**IG^1:** 92% **IG^2:** 94% **IG^3:** 89% **CG:** 97%	N/A	**IG^1:** Exercise training **IG^2:** Functional task exercise **IG^3:** Cognitive training (training of attention, memory, executive function, and visual perceptual function) **CG:** Waiting control (maintaining normal activity or exercise pattern)	**Intensity:** 4–5/10 RPE **Duration:** 8-week **Training load:** 60-min, 1.5 times/week
**Cancela et al.**[117] **2016** **2-arm RCT**	**Diagnosis:** Dementia **Instrument:** DSM	N/A	N/A	**IG:** 73 (32) **CG:** 116 (94)	**IG:** 80.63 ± 8.32 **CG:** 82.90 ± 7.42	**IG:** 70% **CG:** 54%	N/A	**IG:** Aerobic cycling **CG:** Non-physical distractive recreational activities (card-playing, reading, craftwork)	**Intensity**: N/A **Duration:** 60-week **Training load:** 15-min, 7 times/week
**Yu et al.**[118–120] **2021** **2-arm RCT**	**Diagnosis:** Mild to moderate dementia due to AD	N/A	N/A	**IG:** 53 (21) **CG:** 25	**IG:** 77.0 ± 6.6	N/A	**IG:** 15.6 ± 2.8 **CG:** 15.8	**IG:** Aerobic exercise **CG:** Attention control (stretching)	**Intensity:** 50%–75% of HRR, 9–15/20 RPE

	**Instrument:** MMSE, CDR			(11)	**CG:** 78.9 ± 5.6		± 3.0		**Duration:** 24-week **Training load:** 60-min, 3 times/week
**Baker et al.[121,122] 2025 2-arm RCT**	**Diagnosis:** MCI **Instrument:** CDR, MMSE	**IG:** 27.9 ± 1.9 **CG:** 28.0 ± 1.8	N/A	**IG:** 148 (85) **CG:** 148 (84)	**IG:** 74.3 ± 5.7 **CG:** 74.7 ± 6.2	**IG:** 77% **CG:** 78%	**IG:** 16.2 ± 2.4 **CG:** 16.3 ± 2.4	**IG:** Moderate-high intensity aerobic exercise **CG:** Low-intensity stretching, balance, range of motion exercise	**Intensity:** 70%–80% HRR **Duration:** 48-week **Training load:** 45-min, 4 times/week
**Huang et al.[123] 2025 3-arm RCT**	**Diagnosis:** MCI **Instrument:** Peterson criteria	N/A	**IG[1]:** 22.08 ± 2.67 **IG[2]:** 22.44 ± 2.75 **CG:** 22.28 ± 3.53	**IG[1]:** 36 (23) **IG[2]:** 36 (28) **CG:** 36 (26)	**IG[1]:** 71.11 ± 7.65 **IG[2]:** 71.75 ± 6.71 **CG:** 73.86 ± 7.50	**IG[1]:** 81% **IG[2]:** 92% **CG:** 86%	N/A	**IG[1]:** Aerobic exercise **IG[2]:** Resistance exercise **CG:** Control	**Intensity:** IG[1]: 60%–70% HR max, 12–14/20 RPE IG[2]: 50–70% 1RM, 5–6/10 RPE **Duration:** 24-week **Training load:** 30–50-min, 3–5 times/week
**Resistance exercise (RE)**									
**Fernandez-Gonzalo et al.[124] 2016 2-arm RCT**	**Diagnosis:** MCI due to stroke **Instrument:** MMSE	**IG:** 28.1 ± 1.8 **CG:** 27.7 ± 2.3	N/A	**IG:** 14 (3) **CG:** 15 (4)	**IG:** 61.2 ± 9.8 **CG:** 65.7 ± 12.7	**IG:** 88% **CG:** 94%	N/A	**IG:** Flywheel resistance training **CG:** Control	**Intensity:** N/A **Duration:** 12-week **Training load:** N/A-min, 2 times/week
**Singh et al.[125] 2014 4-arm RCT**	**Diagnosis:** MCI **Instrument:** Petersen criteria	**IG[1]:** 27 ± 1 **IG[2]:** 28 ± 2 **IG[3]:** 27 ± 2 **CG:** 27 ± 2	N/A	**IG[1]:** 22 (15) **IG[2]:** 24 (17) **IG[3]:** 27 (18) **CG:** 27 (18)	**IG[1]:** 69.5 ± 5.2 **IG[2]:** 68.4 ± 7.9 **IG[3]:** 71.2 ± 6.2 **CG:** 70.9 ± 7.1	**IG[1]:** 73% **IG[2]:** 92% **IG[3]:** 89% **CG:** 89%	**IG[1]:** 12.5 ± 3.8 **IG[2]:** 13.5 ± 3.2 **IG[3]:** 13.8 ± 4.0 **CG:** 13.4 ± 3.3	**IG[1]:** Progressive resistance training **IG[2]:** Cognitive training **IG[3]:** Combined progressive resistance and cognitive training **CG:** Control	**Intensity:** N/A **Duration:** 24-week **Training load:** 75-min, 2–3 times/week

**Lu et al.[126]<br>2016<br>2-arm RCT**	**Diagnosis:** MCI<br>**Instrument:** MMSE, MoCA	**IG:** 27.23 ± 1.63<br>**CG:** 26.43 ± 2.00	**IG:** 20.59 ± 2.92<br>**CG:** 20.96 ± 2.70	**IG:** 22 (16)<br>**CG:** 23 (16)	**IG:** 69.00 ± 3.83<br>**CG:** 70.43 ± 5.53	**IG:** 95%<br>**CG:** 91%	**IG:** 9.82 ± 2.75<br>**CG:** 9.52 ± 2.61	**IG:** Momentum-based dumbbell training<br>**CG:** Maintain regular lifestyle	**Intensity:** N/A<br>**Duration:** 12-week<br>**Training load:** 60-min, 3 times/week
**Wang et al.[127]<br>2020<br>2-arm RCT**	**Diagnosis:** MCI<br>**Instrument:** MMSE, MoCA	N/A	**IG:** 21.65 ± 2.22<br>**CG:** 21.41 ± 2.11	**IG:** 57 (36)<br>**CG:** 54 (32)	**IG:** 68.37 ± 5.27<br>**CG:** 68.24 ± 5.15	**IG:** 98%<br>**CG:** 96%	N/A	**IG:** Structured limb-exercise program and health promotion classes<br>**CG:** Health promotion classes	**Intensity:** 60%–80% of HRmax<br>**Duration:** 24-week<br>**Training load:** 60-min, 3 times/week
**Vints et al.[128]<br>2024<br>2-arm RCT**	**Diagnosis:** MCI<br>**Instrument:** MoCA	N/A	**IG:** 25.6 ± 2.5<br>**CG:** 24.5 ± 3.4	**IG:** 27 (15)<br>**CG:** 25 (13)	**IG:** 70.7 ± 5.6<br>**CG:** 69.0 ± 5.9	**IG:** 87%<br>**CG:** 64%	N/A	**IG:** Resistance training<br>**CG:** Control	**Intensity:** 70%–85% of 1RM<br>**Duration:** 12-week<br>**Training load:** N/A<br>-min, 2 times/week
**Yoon et al.[129]<br>2018<br>2-arm RCT**	**Diagnosis:** Older adults with cognitive frailty<br>**Instrument:** CDR	**IG:** 24.23 ± 2.89<br>**CG:** 24.22 ± 1.86	N/A	**IG:** 20 (14)<br>**CG:** 23 (16)	**IG:** 73.82 ± 4.37<br>**CG:** 74.03 ± 4.27	**IG:** 61%<br>**CG:** 70%	**IG:** 8.09 ± 3.50<br>**CG:** 9.77 ± 4.44	**IG:** High-speed resistance exercise training<br>**CG:** Control (static and dynamic stretching)	**Intensity:** 12–13/20 RPE<br>**Duration:** 16-week<br>**Training load:** 60-min, 3 times/week
**Yoon et al.[130]<br>2017<br>3-arm RCT**	**Diagnosis:** MCI<br>**Instrument:** MMSE, MoCA	**IG[1]:** 21.00 ± 1.04<br>**IG[2]:** 21.56 ± 0.73<br>**CG:** 22.29 ± 1.11	**IG[1]:** 18.29 ± 2.81<br>**IG[2]:** 16.44 ± 4.22<br>**CG:** 18.71 ± 2.63	**IG[1]:** 14 (14)<br>**IG[2]:** 9 (9)<br>**CG:** 7 (7)	**IG[1]:** 75.00 ± 3.46<br>**IG[2]:** 76.00 ± 3.94<br>**CG:** 78.00 ± 2.77	**IG[1]:** 70%<br>**IG[2]:** 47%<br>**CG:** 37%	N/A	**IG[1]:** High-speed power training<br>**IG[2]:** Low-speed strength training<br>**CG:** Continue their routine daily activities, and carried out static and dynamic stretching once a week for 1 hour	**Intensity:**<br>IG[1]: 12–13/20 RPE<br>IG[2]: 15–16/20 RPE<br>**Duration:** 12-week<br>**Training load:** 60-min, 2 times/week
**Lee et al.[131]<br>2020<br>2-arm RCT**	**Diagnosis:** MCI<br>**Instrument:** CDR, MMSE	**IG:** 23.8 ± 2.9<br>**CG:** 23.4 ± 1.3	N/A	**IG:** 18 (11)<br>**CG:** 22 (13)	**IG:** 73.7 ± 4.6<br>**CG:** 74.2 ± 4.4	**IG:** 90%<br>**CG:** 100%	N/A	**IG:** Elastic band-based High-speed power training<br>**CG:** Lecture to encourage physical activity, exercise counselling	**Intensity:** 12–13/20 RPE<br>**Duration:** 8-week<br>**Training load:** 50-min, 3 times/week
**Hong et al.[132]**	**Diagnosis:**	N/A	**IG:** 20.70	**IG:** 10	**IG[F]:**	**IG:** 80%	N/A	**IG:** Resistance exercise with	**Intensity:** 15RM

**2018**<br>**2-arm RCT**	MCI<br>**Instrument:**<br>MoCA, DSM		± 3.46<br>**CG:**<br>20.08 ± 4.44	(7)<br>**CG:** 12 (9)	77.71 ± 3.40<br>**IG^{M}:**<br>78.33 ± 3.21<br>**CG^{F}:**<br>75.11 ± 4.45<br>**CG^{M}:**<br>78.33 ± 5.50	**CG:** 92%		an elastic band<br>**CG:** Maintain current lifestyle	**Duration:** 12-week<br>**Training load:** 60-min, 2 times/week
**Venturelli et al.[133]**<br>**2010**<br>**2-arm RCT**	**Diagnosis:**<br>MCI<br>**Instrument:**<br>MMSE	**IG:** 22.3 ± 2.1<br>**CG:** 22.1 ± 1.7	N/A	**IG:** 15<br>**CG:** 15	**IG:**<br>83.3 ± 6.7<br>**CG:**<br>84.1 ± 5.8	**IG:** 80%<br>**CG:** 73%	N/A	**IG:** Training program<br>**CG:** Control	**Intensity:** 53–62% of HRmax<br>**Duration:** 12-week<br>**Training load:** 45-min, 3 times/week
**Holthoff et al.[134]**<br>**2015**<br>**2-arm RCT**	**Diagnosis:**<br>Mild to moderate AD<br>**Instrument:**<br>NINCDS-ADRDA, MMSE<br>MRI, Laboratory testing	**IG:** 22.05 ± 0.54<br>**CG:** 21.95 ± 0.54		**IG:** 15 (8)<br>**CG:** 15 (7)	**IG:**<br>72.40 ± 4.34<br>**CG:**<br>70.67 ± 5.41	**IG:** 100%<br>**CG:** 100%	**IG:** 12.33 ± 2.13<br>**CG:** 13.13 ± 2.70	**IG:** Lower body resistive training by a movement trainer computer system<br>**CG:** Clinical visits and counselling about how to change inactive habits and increase the PA level	**Intensity:** N/A<br>**Duration:** 12-week<br>**Training load:** 30-min, 3 times/week
**Baek et al.[135]**<br>**2024**<br>**2-arm RCT**	**Diagnosis:**<br>CI<br>**Instrument:**<br>MMSE	**IG:** 16.09 ± 1.97<br>**CG:** 16.04 ± 2.23	N/A	**IG:** 22 (14)<br>**CG:** 22 (15)	**IG:**<br>81.04 ± 4.93<br>**CG:**<br>82.40 ± 4.46	100%	N/A	**IG^{1}:** Resistance exercise program (a body spider exercise device)<br>**IG^{2}:** Dual-task Resistance exercise program (dual tasks such as writing names, drawing pictures, and subtracting numbers while performing the same resistance exercise applied to the IG^{1})	**Intensity:** 12RM<br>**Duration:** 6-week<br>**Training load:** 40-min, 3 times/week
**Kusleikiene et al.[136]**<br>**2025**<br>**2-arm RCT**	**Diagnosis:**<br>MCI<br>**Instrument:**<br>MoCA	N/A	**IG:** 25.4 ± 2.5<br>**CG:** 24.5 ± 3.4	**IG:** 26 (15)<br>**CG:** 24 (13)	**IG:**<br>70.5<br>**CG:**<br>68.7	**IG:** 84%<br>**CG:** 62%	N/A	**IG:** Lower-limb-focused resistance training<br>**CG:** Control	**Intensity:** 70%–85% 1RM, 7/10 RPE<br>**Duration:** 12-week<br>**Training load:**

									N/A, 2 times/week
**Multicomponent exercise (ME)**									
**Yang et al.[137] 2022 3-arm RCT**	**Diagnosis:** MCI **Instrument:** Diagnosed by a specialist	**IG^{1}:** 26.9 ± 1.7 **IG^{2}:** 27.21 ± 1.9 **CG:** 26.5 ± 2.8	N/A	**IG^{1}:** 33 (30) **IG^{2}:** 33 (20) **CG:** 33 (27)	**IG^{1}:** 67.9 ± 3.6 **IG^{2}:** 72.5 ± 5 **CG:** 72.6 ± 5.6	**IG^{1}:** 91% **IG^{2}:** 94% **CG:** 100%	**IG^{1}:** 8.5 ± 3.9 **IG^{2}:** 9.5 ± 3.7 **CG:** 8.5 ± 3.6	**IG^{1}:** Exercise intervention **IG^{2}:** Virtual-reality-based cognitive training (games contents different domains of brain) **CG:** Education seminars (nutrition and exercise tips on prevention of geriatric diseases)	**Intensity:** Moderate **Duration:** 12-week **Training load:** 30-min, 2 times/week
**Doi et al.[138] 2013 2-arm RCT**	**Diagnosis:** aMCI **Instrument:** MMSE	**IG:** 26.8 ± 1.8 **CG:** 26.6 ± 1.6	N/A	**IG:** 25 (12) **CG:** 25 (11)	**IG:** 75.3 ± 7.5 **CG:** 76.8 ± 6.8	**IG:** 92% **CG:** 96%	**IG:** 11.1 ± 2.4 **CG:** 10.8 ± 2.7	**IG:** Multicomponent exercise program **CG:** 2 education classes about health promotion	**Intensity:** 60% of HRmax **Duration:** 24-week **Training load:** 90-min, 2 times/week
**Uemura et al.[139,140] 2012 2-arm RCT**	**Diagnosis:** MCI **Instrument:** CDR, Petersen criteria	**IG:** 26.8 ± 2.3 **CG:** 26.3 ± 2.7	N/A	**IG:** 50 (25) **CG:** 50 (24)	**IG:** 74.8 ± 7.4 **CG:** 75.8 ± 6.1	**IG:** 88% **CG:** 90%	**IG:** 10.9 ± 2.8 **CG:** 10.3 ± 2.3	**IG:** Exercise program **CG:** Education control (3 education classed about health promoting)	**Intensity:** 60% of HRmax **Duration:** 48-week **Training load:** 90-min, 0.5 times/week
**Li et al.[141] 2021 2-arm RCT**	**Diagnosis:** MCI **Instrument:** MoCA	**IG:** 26.50 ± 1.33 **CG:** 26.62 ± 1.46	**IG:** 21.52 ± 2.05 **CG:** 21.14 ± 1.97	**IG:** 42 (27) **CG:** 42 (24)	N/A	**IG:** 93% **CG:** 93%	N/A	**IG:** Multi-component exercise training **CG:** Regular community heath instruction (how to keep fit)	**Intensity:** 4–5/10 RPE **Duration:** 24-week **Training load:** 30-min, 5 times/week
**Suzuki et al.[142] 2012 2-arm RCT**	**Diagnosis:** MCI **Instrument:** CDR, Petersen criteria	**IG:** 26.8 ± 1.8 **CG:** 26.6 ± 1.6	N/A	**IG:** 25 (12) **CG:** 25 (11)	**IG:** 75.3 ± 7.5 **CG:** 76.8 ± 6.8	**IG:** 96% **CG:** 92%	**IG:** 11.1 ± 2.4 **CG:** 10.8 ± 2.7	**IG:** Multicomponent exercise **CG:** Education control (regarding health promotion)	**Intensity:** 60% of HRmax **Duration:** 48-week **Training load:** 90-min, 2 times/week
**Jurakic et al.[143] 2017 2-arm RCT**	**Diagnosis:** MCI **Instrument:**	N/A	**IG:** 23.43 ± 1.70 **CG:**	**IG:** 14 (14) **CG:** 14	70.4 ± 3.93	N/A	N/A	**IG:** Feedback-based balance and core resistance training **CG:** Pilates training	**Intensity:** N/A **Duration:** 8-week **Training load:** IG:

	MoCA		22.71 ± 1.64	(14)					30-min, CG: 60-min, 3 times/week
**Kim and Yim**[144] **2017** **2-arm RCT**	**Diagnosis:** MCI due to stroke **Instrument:** MMSE	**IG:** 26.35 ± 5.31 **CG:** 25.53 ± 3.27	**IG:** 20.78 ± 7.11 **CG:** 21.4 ± 4.82	**IG:** 14 (5) **CG:** 15 (5)	**IG:** 50.71 ± 14.81 **CG:** 51.87 ± 17.42	**IG:** 93% **CG:** 100%	N/A	**IG:** Exercise intervention and conventional physical therapy **CG:** Conventional physical therapy	**Intensity:** N/A **Duration:** 6-week **Training load:** 60-min, 3 times/week
**Li et al.**[145] **2022** **2-arm RCT**	**Diagnosis:** MCI **Instrument:** NIA-AA, MoCA	N/A	**IG:** 22.61 ± 2.59 **CG:** 23.21 ± 2.40	**IG:** 116 (100) **CG:** 113 (96)	**IG:** 73.93 ± 7.40 **CG:** 74.83 ± 7.57	**IG:** 91% **CG:** 97%	N/A	**IG:** Multicomponent exercise protocol (using mobile application) **CG:** Usual care (social and leisure activities)	**Intensity:** N/A **Duration:** 8-week **Training load:** 60-min, 3 times/week
**Avenali et al.**[146] **2021** **2-arm RCT**	**Diagnosis:** MCI due to PD **Instrument:** MMSE, MoCA	**IG:** 24.46 ± 2.64 **CG:** 24.36 ± 2.62	**IG:** 18.76 ± 3.04 **CG:** 17.99 ± 1.50	**IG:** 15 (8) **CG:** 19 (5)	**IG:** 73.2 ± 7.1 **CG:** 71.6 ± 6.0	**IG:** 94% **CG:** 100%	**IG:** 6.8 ± 3.1 **CG:** 8.2 ± 3.9	**IG:** Physical therapy program **CG:** No specific intervention	**Intensity:** N/A **Duration:** 4-week **Training load:** 60-min, 6 times/week
**Mak et al.**[147] **2022** **2-arm RCT**	**Diagnosis:** Mild to moderate CI/dementia **Instrument:** ACE, MMSE	**IG:** 23.5 (22.6–24.4) **CG:** 24.1 (23.1–25.0)	N/A	**IG:** 76 (47) **CG:** 72 (47)	**IG:** 86.0 (84.8–87.3) **CG:** 87.2 (85.7–88.7)	**IG:** 82% **CG:** 76%	N/A	**IG:** Exercise intervention **CG:** Control	**Intensity:** 12–14/20 RPE First stage: **Duration:** 25-week **Training load:** 60-min, 2 times/week Second stage: **Duration:** 24-week **Training load:** 30-min, 2 times/week
**Sobol et al.**[148–150] **2016** **2-arm RCT**	**Diagnosis:** AD **Instrument:** NINCDS-ADRAD, MMSE	**IG:** 23.8 ± 3.4 **CG:** 24.1 ± 3.8	N/A	**IG:** 107 (51) **CG:** 93 (36)	**IG:** 69.8 ± 7.4 **CG:** 71.3 ± 7.3	**IG:** 96% **CG:** 92%	N/A	**IG:** Exercise intervention **CG:** Usual care	**Intensity:** 70%–80% of HRmax **Duration:** 16-week **Training load:** 60-min, 3 times/week
**Papatsimpas et al.**[151] **2023**	**Diagnosis:** Mild AD **Instrument:**	20–24	N/A	**IG[1]:** 57 (36) **IG[2]:** 57	**IG[1]:** 76.82 ± 5.73	**IG[1]:** 97% **IG[2]:** 98% **CG:** 98%	**IG[1]:** 14.07 ± 2.00 **IG[2]:** 14.54	**IG[1]:** Combined aerobic and resistance exercise **IG[2]:** Resistance exercise	Aerobic: **Intensity:** 64–76% of HRmax

**3-arm RCT**	MMSE			(40)<br>**CG:** 57 (50)	**IG²:** 76.07 ± 5.7<br>**CG:** 78.75 ± 7.06		± 2.08<br>**CG:** 13.61 ± 2.06	**CG:** Usual care	**Training load:** 30-min, 5 times/week<br>Resistance:<br>**Intensity:** 50%–69% of 1RM<br>**Duration:** 12-week<br>**Training load:** 40–45-min, 3 times/week
**Ullrich et al.[152] 2022<br>2-arm RCT**	**Diagnosis:** Mild to moderate CI<br>**Instrument:** MMSE	**IG:** 23.3 ± 2.7<br>**CG:** 23.3 ± 2.1	N/A	**IG:** 63 (48)<br>**CG:** 55 (42)	**IG:** 82.2 ± 5.8<br>**CG:** 82.4 ± 6.2	**IG:** 86%<br>**CG:** 93%	N/A	**IG:** Home-based training program<br>**CG:** Seated exercises (unspecific flexibility and strength exercise, and generic information on nutrition and relaxation)	**Intensity:** N/A<br>**Duration:** 12-week<br>**Training load:** N/A<br>-min, 7 times/week
**Bademli et al.[153] 2018<br>2-arm RCT**	**Diagnosis:** MCI<br>**Instrument:** sMMSE	sMMSE<br>**IG:** 23.27 ± 2.17<br>**CG:** 23.42 ± 1.07	N/A	**IG:** 30 (18)<br>**CG:** 30 (17)	**IG:** 72.24 ± 7.16<br>**CG:** 70.67 ± 8.34	**IG:** 100%<br>**CG:** 100%	N/A	**IG:** Physical activity program (*with music*)<br>**CG:** Control	**Intensity:** N/A<br>**Duration:** 20-week<br>**Training load:** 80-min, 4–7 times/week
**Lok et al.[154] 2023<br>2-arm RCT**	**Diagnosis:** AD<br>**Instrument:** MMSE	**IG:** 23.36 ± 0.48<br>**CG:** 23.50 ± 0.50	N/A	**IG:** 36 (18)<br>**CG:** 36 (19)	**IG:** 72.80 ± 3.99<br>**CG:** 73.88 ± 4.85	**IG:** 100%<br>**CG:** 100%	N/A	**IG:** 30-min *musical* exercise and walking<br>**CG:** Control	**Intensity:** Moderate<br>**Duration:** 12-week<br>**Training load:** 30–40-min, 5 times/week
**De Sa et al.[155] 2024<br>3-arm RCT**	**Diagnosis:** MCI<br>**Instrument:** CDR	**IG¹:** 22.56 ± 0.94<br>**IG²:** 23.89 ± 0.94<br>**IG³:** 24.14 ± 1.06	N/A	**IG¹:** 9 (7)<br>**IG²:** 9 (7)<br>**IG³:** 9 (7)	**IG¹:** 70.0 ± 6.7<br>**IG²:** 73.6 ± 7.4<br>**IG³:** 70.3 ± 5.6	**IG¹:** 60%<br>**IG²:** 56%<br>**IG³:** 60%	**IG¹:** 5.2 ± 2.6<br>**IG²:** 7.8 ± 5.1<br>**IG³:** 5.1 ± 2.6	**IG¹:** Multimodal physical exercise<br>**IG²:** Motor task complexity protocol<br>**IG³:** Combined IG¹ and IG²	**Intensity:** Aerobic sessions: 60%–70% of HRR<br>Strength sessions: 40%–70% of 1RM<br>**Duration:** 24-week<br>**Training load:** 60-min, 2 times/week
**Padala et al.[156] 2017**	**Diagnosis:** Mild AD	**IG:** 23.3 ± 2.2	N/A	**IG:** 15 (5)	**IG:** 72.1 ±	**IG:** 80%<br>**CG:** 80%	N/A	**IG:** Wii-Fit program<br>**CG:** Walking program	**Intensity:** N/A<br>**Duration:** 8-week

**2-arm RCT**	**Instrument:**<br>DSM, MMSE	**CG:** 22.7<br>± 2.3		**CG:** 15<br>(6)	5.3<br>**CG:**<br>73.9 ±<br>7.1				**Training load:** 30-min, 5 times/week
**Langoni et al.**[157,158]<br>**2019**<br>**2-arm RCT**	**Diagnosis:**<br>MCI<br>**Instrument:**<br>MMSE	**IG:** 21.9 ±<br>4.8<br>**CG:** 23.7<br>± 3.7	N/A	**IG:** 26<br>(20)<br>**CG:** 26<br>(20)	**IG:**<br>72.6 ±<br>7.8<br>**CG:**<br>71.9 ±<br>7.9	**IG:** 87%<br>**CG:** 87%	N/A	**IG:** Strength and aerobic walking training<br>**CG:** Control	**Intensity:** 60%–75% of HRmax of walking training<br>**Duration:** 24-week<br>**Training load:** 50–60-min, 2 times/week
**Zhang et al.**[159]<br>**2023**<br>**3-arm RCT**	**Diagnosis:**<br>MCI<br>**Instrument:**<br>MMSE, MoCA	**IG^{1}:** 22.44<br>± 2.33<br>**IG^{2}:** 21.61<br>± 5.15<br>**CG:** 22.94<br>± 5.88	**IG^{1}:**<br>18.33 ±<br>4.18<br>**IG^{2}:**<br>19.06 ±<br>5.20<br>**CG:**<br>19.76 ±<br>4.45	**IG^{1}:** 14<br>(12)<br>**IG^{2}:** 14<br>(13)<br>**CG:** 14<br>(13)	**IG^{1}:**<br>66.67 ±<br>6.04<br>**IG^{2}:**<br>66.22 ±<br>5.51<br>**CG:**<br>69.75 ±<br>7.02	**IG^{1}:** 100%<br>**IG^{2}:** 93%<br>**CG:** 71%	**IG^{1}:** 11.56<br>± 2.45<br>**IG^{2}:** 9.83<br>± 3.96<br>**CG:** 11.25<br>± 4.31	**IG^{1}:** Traditional Chinese exercise combined with Rhythm training (*with music*)<br>**IG^{2}:** 4 km walking exercise<br>**CG:** Control	**Intensity:** 60% of HRmax<br>**Duration:** 12-week<br>**Training load:** 60-min, 3 times/week
**Vreugdenhil et a.**[160]<br>**2012**<br>**2-arm RCT**	**Diagnosis:**<br>AD<br>**Instrument:**<br>DSM	**IG:** 22.9 ±<br>5.0<br>**CG:** 21.0<br>± 6.3	N/A	**IG:** 20<br>(9)<br>**CG:** 20<br>(15)	**IG:**<br>73.5<br>(51-83)<br>**CG:**<br>74.7<br>(58-89)	**IG:** 100%<br>**CG:** 100%	**IG:** 10.1<br>(6–17)<br>**CG:** 10.3<br>(7–17)	**IG:** Home-based exercises and walking (*caregiver involved*)<br>**CG:** Usual treatment	**Intensity:** N/A<br>**Duration:** 16-week<br>**Training load:** N/A-min, 7 times/week
**Papamichail et al.**[161]<br>**2024**<br>**2-arm RCT**	**Diagnosis:**<br>Early dementia due to AD<br>**Instrument:**<br>Diagnosed by a neurologist, DSM, NINCDS-ADRDA, ACE, MMSE	**IG:** 21.50<br>± 1.79<br>**CG:** 22.00<br>± 2.13	N/A	**IG:** 30<br>(20)<br>**CG:** 30<br>(17)	**IG:**<br>80.17 ±<br>6.37<br>**CG:**<br>79.83 ±<br>5.83	N/A	N/A	**IG:** Physiotherapy exercise program<br>**CG:** Control	**Intensity:** Moderate<br>**Duration:** 12-week<br>**Training load:** 45-min, 2 times/week
**Rivas-Campo et al.**[162,163]<br>**2023**<br>**2-arm RCT**	**Diagnosis:**<br>MCI<br>**Instrument:**<br>MMSE	**IG:** 21.5 ±<br>1.5<br>**CG:** 21.1<br>± 1.2	N/A	**IG:** 82<br>(50)<br>**CG:** 87<br>(53)	**IG:**<br>77.4 ±<br>7.3<br>**CG:**<br>76.8 ±<br>7.4	**IG:** 91%<br>**CG:** 97%	N/A	**IG:** High-intensity functional training<br>**CG:** Control	**Intensity:** 80%–85% of HRmax<br>**Duration:** 12-week<br>**Training load:** 45-min, 3 times/week

**Prick et al.**[164]<br>**2017**<br>**2-arm RCT**	**Diagnosis:**<br>Dementia<br>**Instrument:**<br>Diagnosed by physician	**IG:** 21 ± 4.86<br>**CG:** 21 ± 5.56	N/A	**IG:** 57 (26)<br>**CG:** 54 (15)	**IG:** 76 ± 7.61<br>**CG:** 78 ± 7.17	**IG:** 91%<br>**CG:** 85%	**IG:** 3.91 ± 1.42<br>**CG:** 4.11 ± 1.45	**IG:** Exercise intervention (*caregiver involved*)<br>**CG:** Minimal intervention (provide general information about dementia and phone calls)	**Intensity:** N/A<br>**Duration:** 12-week<br>**Training load:** 30-min, 3 times/week
**Hauer et al.**[165–167]<br>**2012**<br>**2-arm RCT**	**Diagnosis:**<br>Dementia<br>**Instrument:**<br>MMSE, CERAD	**IG:** 21.7 ± 2.8<br>**CG:** 21.9 ± 3.2	N/A	**IG:** 62 (46)<br>**CG:** 60 (44)	**IG:** 82.3 ± 6.6<br>**CG:** 82.9 ± 7.0	**IG:** 96%<br>**CG:** 95%	**IG:** 11.0 (5–19)<br>**CG:** 11.0 (7–19)	**IG:** Progressive resistance and functional training<br>**CG:** Motor placebo training (flexibility exercise, calisthenics, low-intensity training with hand-held weights, and ball games while seated)	**Intensity:** 70%–80% of 1RM<br>**Duration:** 12-week<br>**Training load:** 120-min, 2 times/week
**Suttanon et al.**[168]<br>**2013**<br>**2-arm RCT**	**Diagnosis:**<br>AD<br>**Instrument:**<br>MMSE	**IG:** 20.89 ± 4.74<br>**CG:** 21.67 ± 4.43	N/A	**IG:** 19 (13)<br>**CG:** 21 (12)	**IG:** 83.42 ± 5.10<br>**CG:** 80.52 ± 6.01	**IG:** 58%<br>**CG:** 86%	N/A	**IG:** Tailored balance, strengthening and walking exercise<br>**CG:** Education/information program	**Intensity:** N/A<br>**Duration:** 24-week<br>**Training load:** N/A<br>-min, 5 times/week
**Sanders et al.**[169]<br>**2020**<br>**2-arm RCT**	**Diagnosis:**<br>Dementia<br>**Instrument:**<br>Diagnosed by a physician or geriatrician, DSM, MMSE	**IG:** 21.4 ± 3.94<br>**CG:** 19.5 ± 4.77	N/A	**IG:** 39 (21)<br>**CG:** 30 (22)	**IG:** 81.7 ± 7.16<br>**CG:** 82.1 ± 7.51	**IG:** 85%<br>**CG:** 67%	N/A	**IG:** Aerobic outdoor walking and strength exercises<br>**CG:** Flexibility exercise and recreational activities	**Intensity:** 9–16/20 RPE, 57%–89% of HRmax<br>**Duration:** 24-week<br>**Training load:** 30-min, 3 times/week
**Dawson et al.**[170]<br>**2019**<br>**2-arm RCT**	**Diagnosis:**<br>Early-onset dementia<br>**Instrument:**<br>MMSE	**IG:** 19.9 ± 6.1<br>**CG:** 22.0 ± 3.1	N/A	**IG:** 13 (5)<br>**CG:** 10 (6)	**IG:** 73.8 ± 8.5<br>**CG:** 74.0 ± 10.4	**IG:** 83%<br>**CG:** 91%	N/A	**IG:** Home-based functional exercise-strength and balance training<br>**CG:** Continuation of current levels of activity	**Intensity:** 60%–80% of 1RM<br>**Duration:** 12-week<br>**Training load:** N/A<br>-min, 2 times/week
**Santana-Sosa et al.**[171]<br>**2008**<br>**2-arm RCT**	**Diagnosis:**<br>AD<br>**Instrument:**<br>MMSE	18–23	N/A	**IG:** 8 (5)<br>**CG:** 8 (5)	**IG:** 76 ± 4<br>**CG:** 73 ± 4	N/A	N/A	**IG:** Exercise training (*with music*)<br>**CG:** Control	**Intensity:** N/A<br>**Duration:**12-week<br>**Training load:** 75-min, 3 times/week
**Lamb et al.**[172,173]<br>**2018**<br>**2-arm RCT**	**Diagnosis:**<br>Mild to moderate dementia	sMMSE<br>**IG:** 22.0 ± 4.7	N/A	**IG:** 329 (134)<br>**CG:** 165	**IG:** 76.9 ± 7.9	**IG:** 91%<br>**CG:** 88%	N/A	**IG:** Aerobic exercise and strength training<br>**CG:** Usual care	**Intensity:** N/A<br>**Duration:** 48-week

	**Instrument:** sMMSE	**CG:** 21.6 ± 4.6		(59)	**CG:** 78.4 ± 7.6				**Training load:** 60–90-min, 2 times/week
**Kovacs et al.**[174] **2013** **2-arm RCT**	**Diagnosis:** CI **Instrument:** MMSE	**IG:** 20.85 ± 3.2 **CG:** 20.93 ± 3.8	N/A	**IG:** 43 (36) **CG:** 43 (34)	**IG:** 76.39 ± 9.63 **CG:** 79.29 ± 12.67	**IG:** 74% **CG:** 70%	N/A	**IG:** Multimodal exercise program **CG:** Usual care (social activities such as board games, viewing pictures or films, listening to music, arts and crafts activities: embroidery, needlework, conversations)	**Intensity:** N/A **Duration:** 48-week **Training load:** N/A-min, 2 times/week
**Silva et al.**[175] **2019** **2-arm RCT**	**Diagnosis:** MCI and AD **Instrument:** DSM, CDR	**MCI** **IG:** 29 (28–30) **CG:** 29 (26–30) **AD** **IG:** 20.66 ± 5.19 **CG:** 20.90 ± 4.34	N/A	**MCI** **IG:** 7 (6) **CG:** 12 (5) **AD** **IG:** 13 (5) **CG:** 14 (11)	**MCI** **IG:** 71.85 ± 5.69 **CG:** 78.20 ± 5.26 **AD** **IG:** 81.22 ± 8.88 **CG:** 77.54 ± 8.05	N/A	**MCI** **IG:** 13.57 ± 3.90 **CG:** 13.60 ± 4.00 **AD** **IG:** 12 (4–16) **CG:** 8 (4–16)	**IG:** Multimodal training session **CG:** Clinical follow-up, no physical training	**Intensity:** 70% of VO_2max or 80% of HRmax **Duration:** 12-week **Training load:** 60-min, 2 times/week
**Levinger et al.**[176] **2023** **2-arm RCT**	**Diagnosis:** Mild to moderate dementia **Instrument:** sMMSE, MoCA	sMMSE **IG:** 20.2 ± 2.2 **CG:** 19.8 ± 4.7	**IG:** 15.1 ± 3.2 **CG:** 11.5 ± 6.3	**IG:** 8 (7) **CG:** 8 (7)	**IG:** 83.3 ± 7.5 **CG:** 87.5 ± 3.0	**IG:** 78% **CG:** 100%	N/A	**IG:** Exercise park intervention **CG:** Control (recreation and leisure-based group activities)	**Intensity:** N/A **Duration:** 24-week **Training load:** 60–90-min, 2 times/week
**Ghahfarrokhi et al.**[177] **2024** **3-arm RCT**	**Diagnosis:** CI due to T2D **Instrument:** MMSE	**IG**[1]**:** 18.62 ± 2.84 **IG**[2]**:** 19.08 ± 2.45 **CG:** 18.86 ± 2.57	N/A	**IG**[1]**:** 16 **IG**[2]**:** 16 **CG:** 16	**IG**[1]**:** 66.47 ± 6.61 **IG**[2]**:** 68.35 ± 5.44 **CG:** 67.76 ± 5.49	**IG**[1]**:** 94% **IG**[2]**:** 88% **CG:** 88%	N/A	**IG**[1]**:** High intensity functional training **IG**[2]**:** Low intensity functional training **CG:** No exercise intervention	**Intensity:** IG[1]: 75%–85% of HRR IG[2]: 35%–45% of HRR **Duration:** 6-week **Training load:** IG[1]: 30–35-min, 3 times/week

									IG2: 40–45-min, 5 times/week
**Fonte et al.**[178]<br>**2019**<br>**3-arm RCT**	**Diagnosis:**<br>MCI and AD<br>**Instrument:**<br>NIA-AA	**MCI**<br>**IG^1:** 27 ± 2.2<br>**IG^2:** 26.4 ± 1.4<br>**CG:** 25.7 ± 1.8<br>**AD**<br>**IG^1:** 17.8 ± 5.7<br>**IG^2:** 19.6 ± 4.3<br>**CG:** 18.7 ± 2.3	N/A	**MCI**<br>**IG^1:** 7 (3)<br>**IG^2:** 11 (6)<br>**CG:** 9 (7)<br>**AD**<br>**IG^1:** 20 (14)<br>**IG^2:** 19 (12)<br>**CG:** 21 (13)	**MCI**<br>**IG^1:** 75 ± 5<br>**IG^2:** 76 ± 5<br>**CG:** 79 ± 3<br>**AD**<br>**IG^1:** 79 ± 9<br>**IG^2:** 79 ± 7<br>**CG:** 80 ± 7	**IG^1:** 100%<br>**IG^2:** 100%<br>**CG:** 100%	**MCI**<br>**IG^1:** 10 ± 4<br>**IG^2:** 9 ± 4<br>**CG:** 8 ± 4<br>**AD**<br>**IG^1:** 7 ± 4<br>**IG^2:** 8 ± 5<br>**CG:** 7 ± 3	**IG^1:** Physical training<br>**IG^2:** Cognitive training<br>**CG:** Control	**Intensity:** 70% of HRmax, 85% of 1RM<br>**Duration:** 24-week<br>**Training load:** 90-min, 3 times/week
**Gebhard and Mess**[179]<br>**2022**<br>**2-arm RCT**	**Diagnosis:**<br>Dementia<br>**Instrument:**<br>MMSE	**IG:** 18.59 ± 4.60<br>**CG:** 19.90 ± 4.60	N/A	**IG:** 34 (27)<br>**CG:** 29 (22)	**IG:** 86.09 ± 7.64<br>**CG:** 86.34 ± 7.49	**IG:** 79%<br>**CG:** 83%	N/A	**IG:** Multicomponent training program (*with music*)<br>**CG:** Standard care	**Intensity:** N/A<br>**Duration:** 12-week<br>**Training load:** 60-min, 2 times/week
**Koc et al.**[180]<br>**2024**<br>**3-arm RCT**	**Diagnosis:**<br>Mild to moderate AD<br>**Instrument:**<br>MoCA, CDR	N/A	**IG^1:** 16.8 ± 2.5<br>**IG^2:** 16.8 ± 2.0<br>**CG:** 17.4 ± 2.6	**IG^1:** 20 (11)<br>**IG^2:** 20 (12)<br>**CG:** 20 (17)	**IG^1:** 78.9 ± 6.9<br>**IG^2:** 77.9 ± 6.6<br>**CG:** 76.7 ± 6.9	**IG^1:** 100%<br>**IG^2:** 100%<br>**CG:** 100%	N/A	**IG^1:** *Online* supervised physical activity<br>**IG^2:** Physical exercise (same with IG^1) and cognitive stimulation (gamified exercise for visual perception, problem-solving, memory, flexible thinking, attention, language, and speed)<br>**CG:** Control	**Intensity:** N/A<br>**Duration:** 12-week<br>**Training load:** 60-min, 0.5 times/week
**Casas-Herrero et al.**[181]<br>**2022**<br>**2-arm RCT**	**Diagnosis:**<br>MCI or Mild dementia<br>**Instrument:**<br>DSM, GDS	N/A	**IG:** 15.8 ± 5.2<br>**CG:** 15.4 ± 5.2	**IG:** 88 (63)<br>**CG:** 100 (69)	**IG:** 84.2 ± 4.8<br>**CG:** 84.0 ± 4.8	**IG:** 52%<br>**CG:** 72%	N/A	**IG:** Vivifrail multicomponent exercise program<br>**CG:** Usual care	**Intensity:** N/A<br>**Duration:** 12-week<br>**Training load:** 30-min, 5 times/week
**Shaw et al.**[182]<br>**2021**<br>**2-arm RCT**	**Diagnosis:**<br>Mild to moderate AD<br>**Instrument:**	**IG:** 18.36 ± 3.34<br>**CG:** 18.8	N/A	**IG:** 14<br>**CG:** 20	**IG:** 82.21 ± 6.62	N/A	N/A	**IG:** Exercise training<br>**CG:** Control	**Intensity:** N/A<br>**Duration:** 8-week<br>**Training load:** 45-

	NINCDS-ADRAD	± 5.77			**CG:** 78.5 ± 6.69				min, 3 times/week
**Cezar et al.**[183,184] **2021** **2-arm RCT**	**Diagnosis:** Mild to moderate AD **Instrument:** CDR	**IG:** 18.2 ± 3.5 **CG:** 18.4 ± 5.1	N/A	**IG:** 16 (14) **CG:** 19 (9)	**IG:** 79.7 ± 5.9 **CG:** 79.0 ± 5.4	**IG:** 80% **CG:** 95%	N/A	**IG:** Functional exercise **CG:** Maintain habitual levels of physical activity and received telephone calls	**Intensity:** N/A **Duration:** 16-week **Training load:** 60-min, 3 times/week
**Cardalda et al.**[185] **2019** **3-arm RCT**	**Diagnosis:** Mild to moderate CI **Instrument:** MMSE	**IG¹:** 17.04 ± 5.14 **IG²:** 20.48 ± 2.87 **CG:** 17.34 ± 5.38	N/A	**IG¹:** 25 (20) **IG²:** 23 (16) **CG:** 29 (19)	**IG¹:** 85.54 ± 8.09 **IG²:** 83.76 ± 8.33 **CG:** 85.17 ± 7.38	**IG¹:** 100% **IG²:** 100% **CG:** 100%	N/A	**IG¹:** TheraBand **IG²:** Multi-Calisthenics (performed mostly in the seated position) **CG:** Normal activities in nursing home (crafts, reading comprehension and cognitive stimulation)	**Intensity:** 15RM **Duration:** 12-week **Training load:** 60-min, 2 times/week
**Bo et al.**[186] **2019** **4-arm RCT**	**Diagnosis:** Vascular CI **Instrument:** MMSE	**IG¹:** 16.82 ± 5.83 **IG²:** 17.45 ± 5.62 **IG³:** 15.69 ± 6.21 **CG:** 16.79 ± 6.35	N/A	**IG¹:** 42 (19) **IG²:** 44 (19) **IG³:** 45 (21) **CG:** 47 (20)	**IG¹:** 65.12 ± 2.56 **IG²:** 66.68 ± 2.44 **IG³:** 67.51 ± 2.24 **CG:** 64.36 ± 2.31	**IG¹:** 75% **IG²:** 80% **IG³:** 79% **CG:** 82%	**IG¹:** 4.36 ± 1.38 **IG²:** 5.32 ± 1.25 **IG³:** 5.26 ± 1.03 **CG:** 4.48 ± 1.49	**IG¹:** Physical exercise **IG²:** Combined IG¹ and IG³ **IG³:** Cognitive training **CG:** Control	**Intensity:** 13–15/20 RPE **Duration:** 12-week **Training load:** 50-min, 3 times/week
**Kim et al.**[187] **2016** **2-arm RCT**	**Diagnosis:** Moderate to severe AD **Instrument:** Diagnosed by a neurologist, MMSE	**IG:** 13.4 ± 4.2 **CG:** 16.6 ± 4.0	N/A	**IG:** 19 (13) **CG:** 14 (12)	**IG:** 81.9 ± 7.0 **CG:** 80.9 ± 6.1	**IG:** 89% **CG:** 100%	**IG:** 7.4 ± 5.8 **CG:** 8.0 ± 4.2	**IG:** Physical exercise + multicomponent cognitive program **CG:** Multicomponent cognitive program (music therapy, art therapy, horticulture therapy, handicraft, recreational therapy, stretching, laughing therapy, and activity therapy)	**Intensity:** 40%–60% of HRmax, 11–13/20 RPE **Duration:** 24-week **Training load:** 60-min, 5 times/week
**Bossers et al.**[188,189]	**Diagnosis:**	**IG¹:** 15.8	N/A	**IG¹:** 37	**IG¹:**	**IG¹:** 90%	N/A	**IG¹:** Combined Aerobic and	**Intensity:** 12–

**2015**<br>**3-arm RCT**	Mild to severe Dementia<br>**Instrument:** Diagnosed by a specialist, MMSE	± 4.3<br>**IG^{2}:** 15.2 ± 4.8<br>**CG:** 15.9 ± 4.2		(29)<br>**IG^{2}:** 36 (28)<br>**CG:** 36 (25)	85.7 ± 5.1<br>**IG^{2}:** 85.4 ± 5.4<br>**CG:** 85.4 ± 5.0	**IG^{2}:** 88%<br>**CG:** 88%		strength exercise<br>**IG^{2}:** Aerobic exercise (high-intensity walking)<br>**CG:** Social visit	15/20 RPE, 50%–85% of HRmax<br>**Duration:** 9-week<br>**Training load:** 30-min, 4 times/week
**Telenius et al.[190,191]**<br>**2015**<br>**2-arm RCT**	**Diagnosis:** Mild to moderate dementia<br>**Instrument:** CDR	**IG:** 15.5 ± 0.6<br>**CG:** 15.7 ± 4.9	N/A	**IG:** 87 (63)<br>**CG:** 83 (62)	**IG:** 87.3 ± 7.0<br>**CG:** 86.5 ± 7.7	**IG:** 89%<br>**CG:** 93%	N/A	**IG:** High intensity functional exercise<br>**CG:** Light physical activity (reading, playing games, listening to music and making conversations)	**Intensity** 12RM of strengthening exercise<br>**Duration:** 12-week<br>**Training load:** 50-60-min, 2 times/week
**Toots et al.[192–196]**<br>**2017**<br>**2-arm RCT**	**Diagnosis:** Dementia<br>**Instrument:** MMSE	**IG:** 15.4 ± 3.4<br>**CG:** 14.4 ± 3.5	N/A	**IG:** 93 (70)<br>**CG:** 93 (71)	**IG:** 84.4 ± 6.2<br>**CG:** 85.9 ± 7.8	**IG:** 87%<br>**CG:** 91%	N/A	**IG:** High intensity functional exercise program<br>**CG:** Attention control (seated in a group, activities structured topics, conversed, sang, listened to music or readings, and/or looked at pictures and objects)	**Intensity:** 13–15RM<br>**Duration:** 16-week<br>**Training load:** 45-min, 2.5 times/week
**Henskens et al.[197]**<br>**2018**<br>**4-arm RCT**	**Diagnosis:** Dementia<br>**Instrument:** MMSE	**IG^{1}:** 13.6 ± 5.6<br>**IG^{2}:** 13.2 ± 3.7<br>**IG^{3}:** 12.1 ± 6.4<br>**CG:** 10.2 ± 5.7	N/A	**IG^{1}:** 22 (14)<br>**IG^{2}:** 21 (19)<br>**IG^{3}:** 22 (17)<br>**CG:** 22 (17)	**IG^{1}:** 86.95 ± 7.21<br>**IG^{2}:** 86.05 ± 5.86<br>**IG^{3}:** 85.14 ± 4.64<br>**CG:** 84.73 ± 4.55	**IG^{1}:** 73%<br>**IG^{2}:** 81%<br>**IG^{3}:** 73%<br>**CG:** 73%	N/A	**IG^{1}:** Activity of daily living training combined multicomponent exercise<br>**IG^{2}:** Activity of daily living training<br>**IG^{3}:** Multicomponent exercise<br>**CG:** Social activity usual care	**Intensity:** N/A<br>**Duration:** 24-week<br>**Training load:** 30-45-min, 3 times/week
**Kemoun et al.[198]**<br>**2010**<br>**2-arm RCT**	**Diagnosis:** AD dementia<br>**Instrument:** Diagnosis by a neurologist,	**IG:** 12.6 (7–20)<br>**CG:** 12.9 (8–19)	N/A	**IG:** 16 (12)<br>**CG:** 15 (11)	**IG:** 82.0 ± 5.8<br>**CG:** 81.7 ±	**IG:** 80%<br>**CG:** 83%	N/A	**IG:** Physical training<br>**CG:** Control	**Intensity:** N/A<br>**Duration:** 15-week<br>**Training load:** 60-min, 3 times/week

	DSM, MMSE				5.1				
**Barreto et al.[199,200] 2017 2-arm RCT**	**Diagnosis:** AD, or vascular, or mixed dementia **Instrument:** DSM, MMSE	**IG:** 11.4 ± 6.2 **CG:** 10.8 ± 5.5	N/A	**IG:** 44 (41) **CG:** 47 (36)	**IG:** 88.3 ± 5.1 **CG:** 86.9 ± 5.8	**IG:** 85% **CG:** 90%	N/A	**IG:** Group-based exercise multicomponent training **CG:** Group-based therapeutic music mediation or arts and crafts	**Intensity:** Moderate **Duration:** 24-week **Training load:** 60-min, 2 times/week
**Roach et al.[201] 2011 3-arm RCT**	**Diagnosis:** Mild to severe AD **Instrument:** NINCDS-ADRAD	**IG[1]:** 8.71 ± 7.83 **IG[2]:** 12.20 ± 7.47 **CG:** 9.44 ± 7.21	N/A	**IG[1]:** 28 **IG[2]:** 29 **CG:** 25	**IG[1]:** 89.18 ± 6.54 **IG[2]:** 87.31 ± 6.08 **CG:** 88.24 ± 5.80	N/A	N/A	**IG[1]:** Exercise training **IG[2]:** Walking **CG:** Social conversation	**Intensity:** N/A **Duration:** 16-week **Training load:** 30-min, 5 times/week
**Rolland et al.[202] 2007 2-arm RCT**	**Diagnosis:** Mild to severe AD **Instrument:** MMSE, NINCD-ADRDA	**IG:** 9.7 ± 6.8 **CG:** 7.9 ± 6.4	N/A	**IG:** 67 (48) **CG:** 67 (53)	**IG:** 82.8 ± 7.8 **CG:** 83.1 ± 7.0	**IG:** 84% **CG:** 81%	N/A	**IG:** Exercise program (*with music*) **CG:** Routine medical care	**Intensity:** N/A **Duration:** 48-week **Training load:** 60-min, 2 times/week
**Stevens and Killeen[203] 2006 3-arm RCT**	**Diagnosis:** Mild to moderate dementia **Instrument:** MMSE	< 23	N/A	**IG:** 24 (23) **CG[1]:** 21 (10) **CG[2]:** 30 (23)	**IG:** 79 **CG[1]:** 81.5 **CG[2]:** 81	N/A	N/A	**IG:** Exercise intervention (*with music*) **CG[1]:** Equivalent social visit (interactive discission on health-related issues) **CG[2]:** No intervention	**Intensity:** N/A **Duration:** 12-week **Training load:** 30-min, 3 times/week
**Brett et al.[204] 2021 3-arm RCT**	**Diagnosis:** Dementia **Instrument:** N/A	N/A	N/A	**IG[1]:** 17 (13) **IG[2]:** 19 (13) **CG:** 19 (10)	**IG[1]:** 86 **IG[2]:** 84 **CG:** 86	**IG[1]:** 85% **IG[2]:** 95% **CG:** 95%	N/A	**IG[1]:** Variety of exercise **IG[2]:** Variety of exercise **CG:** Low intensity group activities (bingo, observing concerts, watching movies, carpet bowls, quizzes, and gentle range of movement exercises)	**Intensity:** N/A **Duration:** 12-week **Training load:** **IG[1]:** 45-min, 1 time/week **IG[2]:** 15-min, 3 times/week
**Almeida et al.[205] 2021 2-arm RCT**	**Diagnosis:** Dementia **Instrument:** DSM	N/A	N/A	**IG:** 6 (4) **CG:** 6 (4)	**IG:** 82.5 (78.5–86.2) **CG:** 80	**IG:** 100% **CG:** 100%	N/A	**IG:** Home-based physical activity program **CG:** Usual care (prescribed medication)	**Intensity:** N/A **Duration:** 12-week **Training load:** 60-min, 3 times/week

					(68.8–87.8)				in the first month, 2 times/week in the second month, and 1 time/week in the last month
**Verdelho et al.[206] 2024 2-arm RCT**	**Diagnosis:** Mild vascular cognitive impairment **Instrument:** N/A	N/A	**IG:** 23 (21.5-25) **CG:** 24 (22-26)	**IG:** 53 (29) **CG:** 51 (24)	**IG:** 73 (68-77) **CG:** 71 (65-77)	**IG:** 87% **CG:** 90%	**IG:** 9 (4-15) **CG:** 9 (4-15)	**IG:** Combine aerobic (walking), resistance, balance, agility, and coordinative exercises **CG:** Usual care	**Intensity:** 12-15/20 RPE **Duration:** 24-week **Training load:** 60-mins, 3 times/week
**Yan et al.[207] 2024 2-arm RCT**	**Diagnosis:** MCI due to PD **Instrument:** MoCA, MMSE	**IG:** 21.0 ± 3.17 **CG:** 19.6 ± 3.0	**IG:** 26.6 ± 1.7 **CG:** 26.2 ± 2.1	**IG:** 23 (8) **CG:** 23 (6)	**IG:** 70.5 ± 5.4 **CG:** 68.2 ± 5.8	**IG:** 83% **CG:** 78%	N/A	**IG:** Comprising resistance, balance, and gait trainings (online) **CG:** Active control	**Intensity:** N/A **Duration:** 12-week **Training load:** 60-min, 3 times/week
**David et al.[208] 2025 2-arm RCT**	**Diagnosis:** MCI or mild AD **Instrument:** Biomarkers, Neuroimaging, Clinical evaluation	**IG:** 24.09 ± 3.16 **CG:** 25.05 ± 3.6	**IG:** 17.14 ± 3.97 **CG:** 21.26 ± 4.78	**IG:** 22 (9) **CG:** 19 (7)	**IG:** 72.1 ± 5.8 **CG:** 68 ± 8.2	**IG:** 85% **CG:** 95%	**IG:** 12.5 ± 3.8 **CG:** 14.8 ± 2.4	**IG:** Aerobic, resistance, and coordination exercises **CG:** Control	**Intensity:** N/A **Duration:** 24-week **Training load:** 105-min, 1 time/week
**Shokri et al.[209] 2024 3-arm RCT (two arms were included)**	**Diagnosis:** Mild to moderate dementia **Instrument:** DSM-5	N/A	N/A	**IG:** 15 **CG:** 15	50-75 years	**IG:** 87% **CG:** 100%	N/A	**IG:** Balance, coordination, stretching, endurance, and aerobic exercises (*caregiver involved*, *online, with music*) **CG:** Control	**Intensity:** 4–6/10 RPE **Duration:** 12-week **Training load:** 35–45-min, 3 times/week

Notes:
*Adherence rate is the posttest point.
*All diagnosis is according to the publication criteria, rather than MMSE or MoCA.

Abbreviations:
RCT: Randomized controlled trial
EG: Experiment group
CG: Control group
CI: Cognitive Impairment
MCI: Mild Cognitive Impairment
aMCI: Amnestic Mild Cognitive Impairment

AD: Alzheimer’s disease
T2D: Type 2 diabetes
PD: Parkinson’s disease
HR: Heart rate
HRR: Heart Rate Reserve
RM: Repetition maximum
RPE: Rate of perceived exertion (RPE)
MMSE: Mini-Mental State Examination
MoCA: Montreal Cognitive Assessment
CDR: Clinical Dementia Rating
NIA-AA: National Institute on Ageing and Alzheimer’s Association guidelines
GDS: Global Deterioration Scale
DSM: Diagnostic and Statistical Manual
NINCDS-ADRDA: Neurological and Communicative Disorders and Stroke-Alzheimer’s Disease and Related Disorders Association
NINDS-IAREN: National Institute of Neurological Disorders and Stroke- International Association for Education in Neurosciences
CERAD: Consortium to Establish a Registry for Alzheimer’s Disease
ICD-10: 10th revision of the International Classification of Diseases
ACE: Mini Addenbrooke’s Cognitive Examination
NPI: Neuropsychiatric Inventory

References

1. Kropacova S, Mitterova K, Klobusiakova P, et al. Cognitive effects of dance-movement intervention in a mixed group of seniors are not dependent on hippocampal atrophy. *J Neural Transm*. 2019;126(11):1455-1463. doi:10.1007/s00702-019-02068-y
2. Esmail A, Vrinceanu T, Lussier M, et al. Effects of Dance/Movement Training vs. Aerobic Exercise Training on cognition, physical fitness and quality of life in older adults: A randomized controlled trial. *J Bodyw Mov Ther*. 2020;24(1):212-220. doi:10.1016/j.jbmt.2019.05.004
3. Bisbe M, Fuente-Vidal A, López E, et al. Comparative Cognitive Effects of Choreographed Exercise and Multimodal Physical Therapy in Older Adults with Amnestic Mild Cognitive Impairment: Randomized Clinical Trial. *Journal of Alzheimer’s Disease*. 2020;73(2):769-783. doi:10.3233/JAD-190552
4. Qi M, Zhu Y, Zhang L, Wu T, Wang J. The effect of aerobic dance intervention on brain spontaneous activity in older adults with mild cognitive impairment: A resting-state functional MRI study. *Exp Ther Med*. Published online 2018:715-722. doi:10.3892/etm.2018.7006
5. Lazarou I, Parastatidis T, Tsolaki A, et al. International Ballroom Dancing Against Neurodegeneration: A Randomized Controlled Trial in Greek Community-Dwelling Elders With Mild Cognitive impairment. *Am J Alzheimers Dis Other Demen*. 2017;32(8):489-499. doi:10.1177/1533317517725813
6. Franco MR, Sherrington C, Tiedemann A, et al. Effect of Senior Dance (DanSE) on Fall Risk Factors in Older Adults: A Randomized Controlled Trial. *Phys Ther*. 2020;100(4):600-608. doi:10.1093/ptj/pzz187
7. Doi T, Verghese J, Makizako H, et al. Effects of Cognitive Leisure Activity on Cognition in Mild Cognitive Impairment: Results of a Randomized Controlled Trial. *J Am Med Dir Assoc*. 2017;18(8):686-691. doi:10.1016/j.jamda.2017.02.013
8. Blumen HM, Ayers E, Wang C, Ambrose AF, Jayakody O, Verghese J. Randomized Controlled Trial of Social Ballroom Dancing and Treadmill Walking: Preliminary Findings on Executive Function and Neuroplasticity From Dementia-at-Risk Older Adults. *J Aging Phys Act*. 2023;31(4):589-599.

doi:10.1123/japa.2022-0176
9. Zhu Y, Wu H, Qi M, et al. Effects of a specially designed aerobic dance routine on mild cognitive impairment. *Clin Interv Aging*. 2018;13:1691-1700. doi:10.2147/CIA.S163067
10. Song D, Yu D, Liu T, Wang J. Effect of an Aerobic Dancing Program on Sleep Quality for Older Adults With Mild Cognitive Impairment and Poor Sleep: A Randomized Controlled Trial. *J Am Med Dir Assoc*. 2024;25(3):494-499. doi:10.1016/j.jamda.2023.09.020
11. Chang J, Zhu W, Zhang J, et al. The Effect of Chinese Square Dance Exercise on Cognitive Function in Older Women With Mild Cognitive Impairment: The Mediating Effect of Mood Status and Quality of Life. *Front Psychiatry*. 2021;12(July). doi:10.3389/fpsyt.2021.711079
12. Chang J, Chen Y, Liu C, et al. Effect of Square Dance Exercise on Older Women With Mild Mental Disorders. *Front Psychiatry*. 2021;12(July):1-9. doi:10.3389/fpsyt.2021.699778
13. Van de Winckel A, Feys H, De Weerdt W, Dom R. Cognitive and behavioural effects of music-based exercises in patients with dementia. *Clin Rehabil*. 2004;18(3):253-260. doi:10.1191/0269215504cr750oa
14. Bracco L, Pinto-Carral A, Hillaert L, Mourey F. Tango-therapy vs physical exercise in older people with dementia; a randomized controlled trial. *BMC Geriatr*. 2023;23(1):1-13. doi:10.1186/s12877-023-04342-x
15. Zhu Y, Gao Y, Guo C, et al. Effect of 3-Month Aerobic Dance on Hippocampal Volume and Cognition in Elderly People With Amnestic Mild Cognitive Impairment: A Randomized Controlled Trial. *Front Aging Neurosci*. 2022;14(March):1-10. doi:10.3389/fnagi.2022.771413
16. Ho RTH, Fong TCT, Chan WC, et al. Psychophysiological Effects of Dance Movement Therapy and Physical Exercise on Older Adults with Mild Dementia: A Randomized Controlled Trial. *Journals of Gerontology - Series B Psychological Sciences and Social Sciences*. 2018;75(3):560-570. doi:10.1093/geronb/gby145
17. Thiel U, Stiebler M, Labott BK, et al. DiADEM—Dance against Dementia—Effect of a Six-Month Dance Intervention on Physical Fitness in Older Adults with Mild Cognitive Impairment: A Randomized, Controlled Trial. *J Pers Med*. 2024;14(8). doi:10.3390/jpm14080888
18. Sánchez-Alcalá M, Aibar-Almazán A, Hita-Contreras F, et al. Effects of Dance-Based Aerobic Training on Mental Health and Quality of Life in Older Adults with Mild Cognitive Impairment. *J Pers Med*. 2024;14(8):1-16. doi:10.3390/jpm14080844
19. Sánchez-Alcalá M, Aibar-Almazán A, Carcelén-Fraile M del C, et al. Effects of Dance-Based Aerobic Training on Frailty and Cognitive Function in Older Adults with Mild Cognitive Impairment: A Randomized Controlled Trial. *Diagnostics*. 2025;15(3):1-13. doi:10.3390/diagnostics15030351
20. Eggenberger P, Schumacher V, Angst M, Theill N, de Bruin ED. Does multicomponent physical exercise with simultaneous cognitive training boost cognitive performance in older adults? A 6-month randomized controlled trial with a 1-year follow-up. *Clin Interv Aging*. 2015;10:1335-1349. doi:10.2147/CIA.S87732
21. Eggenberger P, Theill N, Holenstein S, Schumacher V, de Bruin ED. Multicomponent physical exercise with simultaneous cognitive training to enhance dual-task walking of older adults: A secondary analysis of a 6-month randomized controlled trial with I-year follow-up. *Clin Interv Aging*. 2015;10:1711-1732. doi:10.2147/CIA.S91997
22. Hughes TF, Flatt JD, Fu B, Butters MA, Chang CCH, Ganguli M. Interactive video gaming compared with health education in older adults with mild cognitive impairment: A feasibility study. *Int J Geriatr Psychiatry*. 2014;29(9):890-898. doi:10.1002/gps.4075
23. Sabbagh et al. Sensor-based balance training with motion feedback in people with mild cognitive impairment. 2016;53(6):945-958. doi:10.1682/JRRD.2015.05.0089.Sensor-based

24. Liu CL, Cheng FY, Wei MJ, Liao YY. Effects of Exergaming-Based Tai Chi on Cognitive Function and Dual-Task Gait Performance in Older Adults With Mild Cognitive Impairment: A Randomized Control Trial. *Front Aging Neurosci*. 2022;14(March). doi:10.3389/fnagi.2022.761053
25. Padala KP, Padala PR, Malloy TR, et al. Wii-fit for improving gait and balance in an assisted living facility: A pilot study. *J Aging Res*. 2012;2012:6-11. doi:10.1155/2012/597573
26. Liao YY, Chen IH, Hsu WC, Tseng HY, Wang RY. Effect of exergaming versus combined exercise on cognitive function and brain activation in frail older adults: A randomised controlled trial. *Ann Phys Rehabil Med*. 2021;64(5):101492. doi:10.1016/j.rehab.2021.101492
27. Karssemeijer EGA, Aaronson JA, Bossers WJR, Donders R, Olde Rikkert MGM, Kessels RPC. The quest for synergy between physical exercise and cognitive stimulation via exergaming in people with dementia: A randomized controlled trial. *Alzheimers Res Ther*. 2019;11(1):1-13. doi:10.1186/s13195-018-0454-z
28. Karssemeijer EGA, Bossers WJR, Aaronson JA, Sanders LMJ, Kessels RPC, Olde Rikkert MGM. Exergaming as a Physical Exercise Strategy Reduces Frailty in People With Dementia: A Randomized Controlled Trial. *J Am Med Dir Assoc*. 2019;20(12):1502-1508.e1. doi:10.1016/j.jamda.2019.06.026
29. van Santen J, Dröes RM, Twisk JWR, Blanson Henkemans OA, van Straten A, Meiland FJM. Effects of Exergaming on Cognitive and Social Functioning of People with Dementia: A Randomized Controlled Trial. *J Am Med Dir Assoc*. 2020;21(12):1958-1967.e5. doi:10.1016/j.jamda.2020.04.018
30. Wu S, Ji H, Won J, Jo EA, Kim YS, Park JJ. The Effects of Exergaming on Executive and Physical Functions in Older Adults With Dementia: Randomized Controlled Trial. *J Med Internet Res*. 2023;25:1-17. doi:10.2196/39993
31. Swinnen N, Vandenbulcke M, de Bruin ED, et al. The efficacy of exergaming in people with major neurocognitive disorder residing in long-term care facilities: a pilot randomized controlled trial. *Alzheimers Res Ther*. 2021;13(1):1-13. doi:10.1186/s13195-021-00806-7
32. Zheng J, Yu P, Chen X. An Evaluation of the Effects of Active Game Play on Cognition, Quality of Life and Depression for Older People with Dementia. *Clin Gerontol*. 2022;45(4):1034-1043. doi:10.1080/07317115.2021.1980170
33. Uğur F, Sertel M. Wii Fit Exercise's Effects on Muscle Strength and Fear of Falling in Older Adults With Alzheimer Disease: A Randomized Controlled Trial. *J Aging Phys Act*. 2025;33(2):181-191. doi:10.1123/japa.2023-0428
34. Grzenda A, Siddarth P, Milillo MM, Aguilar-Faustino Y, Khalsa DS, Lavretsky H. Cognitive and immunological effects of yoga compared to memory training in older women at risk for alzheimer's disease. *Transl Psychiatry*. 2024;14(1):1-11. doi:10.1038/s41398-024-02807-0
35. Khanthong P, Sriyakul K, Dechakhamphu A, Krajarng A, Kamalashiran C, Tungsukruthai P. Traditional Thai exercise (Ruesi Dadton) for improving motor and cognitive functions in mild cognitive impairment: a randomized controlled trial. *J Exerc Rehabil*. 2021;17(5):331-338. doi:10.12965/JER.2142542.271
36. Kashyap M, Rai NK, Singh R, et al. Effect of Early Yoga Practice on Post Stroke Cognitive Impairment. 2022;22(4):2019. doi:10.4103/aian.AIAN
37. Tremont G, Davis J, Ott BR, et al. Feasibility of a Yoga Intervention for Individuals with Mild Cognitive Impairment: A Randomized Controlled Trial. *Journal of Integrative and Complementary Medicine*. 2022;28(3):250-260. doi:10.1089/jicm.2021.0204
38. Eyre HA, Siddarth P, Acevedo B, et al. A randomized controlled trial of Kundalini yoga in mild cognitive impairment. *Int Psychogeriatr*. 2017;29(4):557-567. doi:10.1017/S1041610216002155
39. Eyre HA, Acevedo B, Yang H, et al. Changes in Neural Connectivity and Memory Following a Yoga Intervention for Older Adults: A Pilot Study. *Journal of Alzheimer's Disease*. 2016;52(2):673-684. doi:10.3233/JAD-150653
40. Li F, Harmer P, Fitzgerald K, Winters-Stone K. A cognitively enhanced online Tai Ji Quan training intervention for community-dwelling older adults with mild cognitive impairment: A feasibility trial. *BMC Geriatr*. 2022;22(1):1-13. doi:10.1186/s12877-021-02747-0

41. Sungkarat S, Boripuntakul S, Kumfu S, Lord SR, Chattipakorn N. Tai Chi Improves Cognition and Plasma BDNF in Older Adults With Mild Cognitive Impairment: A Randomized Controlled Trial. *Neurorehabil Neural Repair*. 2018;32(2):142-149. doi:10.1177/1545968317753682
42. Sungkarat S, Boripuntakul S, Chattipakorn N, Watcharasaksilp K, Lord SR. Effects of Tai Chi on Cognition and Fall Risk in Older Adults with Mild Cognitive Impairment: A Randomized Controlled Trial. *J Am Geriatr Soc*. 2017;65(4):721-727. doi:10.1111/jgs.14594
43. Li F, Harmer P, Voit J, Chou LS. Implementing an online virtual falls prevention intervention during a public health pandemic for older adults with mild cognitive impairment: A feasibility trial. *Clin Interv Aging*. 2021;16:973-983. doi:10.2147/CIA.S306431
44. Lam LCW, Chau RCM, Wong BML, et al. A 1-Year Randomized Controlled Trial Comparing Mind Body Exercise (Tai Chi) With Stretching and Toning Exercise on Cognitive Function in Older Chinese Adults at Risk of Cognitive Decline. *J Am Med Dir Assoc*. 2012;13(6):568.e15-568.e20. doi:10.1016/j.jamda.2012.03.008
45. Lam LCW, Chau RCM, Wong BML, et al. Interim follow-up of a randomized controlled trial comparing Chinese style mind body (Tai Chi) and stretching exercises on cognitive function in subjects at risk of progressive cognitive decline. *Int J Geriatr Psychiatry*. 2011;26(7):733-740. doi:10.1002/gps.2602
46. Jiayuan Z, Xiang-Zi J, Li-Na M, Jin-Wei Y, Xue Y. Effects of Mindfulness-Based Tai Chi Chuan on Physical Performance and Cognitive Function among Cognitive Frailty Older Adults: A Six-Month Follow-Up of a Randomized Controlled Trial. *Journal of Prevention of Alzheimer's Disease*. 2022;9(1):104-112. doi:10.14283/jpad.2021.40
47. Chen Y, Qin J, Tao L, et al. Effects of Tai Chi Chuan on Cognitive Function in Adults 60 Years or Older With Type 2 Diabetes and Mild Cognitive Impairment in China: A Randomized Clinical Trial. *JAMA Netw Open*. 2023;6(4):E237004. doi:10.1001/jamanetworkopen.2023.7004
48. Lin M, Liu W, Ma C, et al. Tai Chi-Induced Exosomal LRP1 is Associated With Memory Function and Hippocampus Plasticity in aMCI Patients. *American Journal of Geriatric Psychiatry*. 2024;32(10):1215-1230. doi:10.1016/j.jagp.2024.04.012
49. Yu AP, Chin EC, Yu DJ, et al. Tai Chi versus conventional exercise for improving cognitive function in older adults: a pilot randomized controlled trial. *Sci Rep*. 2022;12(1):1-15. doi:10.1038/s41598-022-12526-5
50. Huang N, Li W, Rong X, et al. Effects of a Modified Tai Chi Program on Older People with Mild Dementia: A Randomized Controlled Trial. *Journal of Alzheimer's Disease*. 2019;72(3):947-956. doi:10.3233/JAD-190487
51. Cheng ST, Chow PK, Song YQ, et al. Mental and physical activities delay cognitive decline in older persons with dementia. *American Journal of Geriatric Psychiatry*. 2014;22(1):63-74. doi:10.1016/j.jagp.2013.01.060
52. Cheng ST, Chow PK, Yu ECS, Chan ACM. Leisure activities alleviate depressive symptoms in nursing home residents with very mild or mild dementia. *American Journal of Geriatric Psychiatry*. 2012;20(10):904-908. doi:10.1097/JGP.0b013e3182423988
53. Liu JYW, Kwan RYC, Lai CKY, Hill KD. A simplified 10-step Tai-chi programme to enable people with dementia to improve their motor performance: a feasibility study. *Clin Rehabil*. 2018;32(12):1609-1623. doi:10.1177/0269215518786530
54. Chan AWK, Yu DSF, Choi KC, Lee DTF, Sit JWH, Chan HYL. Tai chi qigong as a means to improve night-time sleep quality among older adults with cognitive impairment: A pilot randomized controlled trial. *Clin Interv Aging*. 2016;11:1277-1286. doi:10.2147/CIA.S111927
55. Nyman SR, Ingram W, Sanders J, et al. Randomised controlled trial of the effect of tai chi on postural balance of people with dementia. *Clin Interv Aging*. 2019;14:2017-2029. doi:10.2147/CIA.S228931
56. Williams J, Nyman S. A secondary analysis of a randomised controlled trial to investigate the effect of Tai Chi on the instrumented timed up and go test in

people with mild to moderate dementia. *Aging Clin Exp Res*. 2021;33(8):2175-2181. doi:10.1007/s40520-020-01741-7
57. Canan Okuyan ED. The effectiveness of Tai Chi Chuan on fear of movement, prevention of falls, physical activity, and cognitive status in older adults with mild cognitive impairment: A randomized controlled trial. *Perspect Psychiatr Care*. 2021;57(3):1273-1281. doi:10.1111/ppc.12684
58. Su H, Wang H, Meng L, Bush E. The effects of Baduanjin exercise on the subjective memory complaint of older adults: A randomized controlled trial. *Medicine (United States)*. 2021;100(30):E25442. doi:10.1097/MD.0000000000025442
59. Zheng G, Zheng Y, Xiong Z, Ye B. Effect of Baduanjin exercise on cognitive function in patients with post-stroke cognitive impairment: a randomized controlled trial. *Clin Rehabil*. 2020;34(8):1028-1039. doi:10.1177/0269215520930256
60. Xia R, Wan M, Lin H, Ye Y, Chen S, Zheng G. Effects of mind–body exercise Baduanjin on cognition in community-dwelling older people with mild cognitive impairment: A randomized controlled trial. *Neuropsychol Rehabil*. 2023;33(8):1368-1383. doi:10.1080/09602011.2022.2099909
61. Zheng G, Ye B, Xia R, et al. Traditional Chinese Mind-Body Exercise Baduanjin Modulate Gray Matter and Cognitive Function in Older Adults with Mild Cognitive Impairment: A Brain Imaging Study. *Brain Plasticity*. 2021;7(2):131-142. doi:10.3233/bpl-210121
62. Xia R, Qiu P, Lin H, et al. The effect of traditional chinese mind-body exercise (Baduanjin) and brisk walking on the dorsal attention network in older adults with mild cognitive impairment. *Front Psychol*. 2019;10(SEP):1-9. doi:10.3389/fpsyg.2019.02075
63. Li K, Yu H, Kortas JA, Lin X, Lipowski M. The effect of 12 weeks of Baduanjin exercise on cognitive function, lower limb balance and quality of life of the elderly with mild cognitive impairment: a randomized controlled trial. *Gazzetta Medica Italiana Archivio per le Scienze Mediche*. 2022;181(11):811-823. doi:10.23736/S0393-3660.22.04802-1
64. Luo SS, Chen L, Wang GB, Wang YG, Su XY. Effects of long-term Wuqinxi exercise on working memory in older adults with mild cognitive impairment. *Eur Geriatr Med*. 2022;13(6):1327-1333. doi:10.1007/s41999-022-00709-2
65. Chang CL, Lin TK, Pan CY, et al. Distinct effects of long-term Tai Chi Chuan and aerobic exercise interventions on motor and neurocognitive performance in early-stage Parkinson's disease: a randomized controlled trial. *Eur J Phys Rehabil Med*. 2024;60(4):621-633. doi:10.23736/S1973-9087.24.08166-8
66. Gao R, Greiner C, Ryuno H, Zhang X. Effects of Tai Chi on physical performance, sleep, and quality of life in older adults with mild to moderate cognitive impairment. *BMC Complement Med Ther*. 2024;24(1). doi:10.1186/s12906-024-04705-w
67. Tomoto T, Liu J, Tseng BY, et al. One-Year Aerobic Exercise Reduced Carotid Arterial Stiffness and Increased Cerebral Blood Flow in Amnestic Mild Cognitive Impairment. *Journal of Alzheimer's Disease*. 2021;80(2):841-853. doi:10.3233/JAD-201456
68. Shimada H, Lee S, Akishita M, et al. Effects of golf training on cognition in older adults: A randomised controlled trial. *J Epidemiol Community Health (1978)*. 2018;72(10):944-950. doi:10.1136/jech-2017-210052
69. Donnezan et al. Effects of simultaneous aerobic and cognitive training on executive functions, cardiovascular fitness and functional abilities in older adults with mild cognitive impairment. *Ment Health Phys Act*. 2018;15(April):78-87. doi:10.1016/j.mhpa.2018.06.001
70. Baker LD, Frank LL, Foster-Schubert K, et al. Effects of aerobic exercise on mild cognitive impairment: A controlled trial. *Arch Neurol*. 2010;67(1):71-79. doi:10.1001/archneurol.2009.307
71. Ihle-Hansen H, Langhammer B, Lydersen S, Gunnes M, Indredavik B, Askim T. A physical activity intervention to prevent cognitive decline after stroke: Secondary results from the life after stroke study, an 18-month randomized controlled trial. *J Rehabil Med*. 2019;51(9):646-651. doi:10.2340/16501977-2588
72. Askim T, Langhammer B, Ihle-Hansen H, et al. Efficacy and safety of individualized coaching after stroke: The LAST study (life after stroke) a pragmatic

randomized controlled trial. *Stroke*. 2018;49(2):426-432. doi:10.1161/STROKEAHA.117.018827
73. Nagamatsu LS, Chan A, Davis JC, et al. Physical activity improves verbal and spatial memory in older adults with probable mild cognitive impairment: A 6-month randomized controlled trial. *J Aging Res*. 2013;2013(Mci). doi:10.1155/2013/861893
74. Ten Brinke LF, Bolandzadeh N, Nagamatsu LS, et al. Aerobic exercise increases hippocampal volume in older women with probable mild cognitive impairment: A 6-month randomised controlled trial. *Br J Sports Med*. 2015;49(4):248-254. doi:10.1136/bjsports-2013-093184
75. Tsai CL, Pai MC, Ukropec J, Ukropcová B. Distinctive Effects of Aerobic and Resistance Exercise Modes on Neurocognitive and Biochemical Changes in Individuals with Mild Cognitive Impairment. *Curr Alzheimer Res*. 2019;16(4):316-332. doi:10.2174/1567205016666190228125429
76. Hsu CL, Best JR, Davis JC, et al. Aerobic exercise promotes executive functions and impacts functional neural activity among older adults with vascular cognitive impairment. *Br J Sports Med*. 2018;52(3):184-191. doi:10.1136/bjsports-2016-096846
77. ten Brinke LF, Hsu CL, Best JR, Barha CK, Liu-Ambrose T. Increased Aerobic Fitness Is Associated with Cortical Thickness in Older Adults with Mild Vascular Cognitive Impairment. *Journal of Cognitive Enhancement*. 2018;2(2):157-169. doi:10.1007/s41465-018-0077-0
78. Hsu CL, Best JR, Wang S, et al. The impact of aerobic exercise on fronto-parietal network connectivity and its relation to mobility: An exploratory analysis of a 6-month randomized controlled trial. *Front Hum Neurosci*. 2017;11(June):1-12. doi:10.3389/fnhum.2017.00344
79. Dao E, Best JR, Hsiung GYR, et al. Associations between cerebral amyloid and changes in cognitive function and falls risk in subcortical ischemic vascular cognitive impairment. *BMC Geriatr*. 2017;17(1):1-9. doi:10.1186/s12877-017-0522-4
80. Liu-Ambrose T, Best JR, Davis JC, et al. Aerobic exercise and vascular cognitive impairment. *Neurology*. 2016;87(20):2082-2090. doi:10.1212/WNL.0000000000003332
81. Song D, Yu DSF. Effects of a moderate-intensity aerobic exercise programme on the cognitive function and quality of life of community-dwelling elderly people with mild cognitive impairment: A randomised controlled trial. *Int J Nurs Stud*. 2019;93:97-105. doi:10.1016/j.ijnurstu.2019.02.019
82. Stuckenschneider T, Sanders ML, Devenney KE, et al. NeuroExercise: The Effect of a 12-Month Exercise Intervention on Cognition in Mild Cognitive Impairment—A Multicenter Randomized Controlled Trial. *Front Aging Neurosci*. 2021;12(January):1-12. doi:10.3389/fnagi.2020.621947
83. Rojasavastera R, Bovonsunthonchai S, Hiengkaew V, Senanarong V. Action observation combined with gait training to improve gait and cognition in elderly with mild cognitive impairment a randomized controlled trial. *Dementia e Neuropsychologia*. 2020;14(2):118-127. doi:10.1590/1980-57642020dn14-020004
84. Makino T, Umegaki H, Ando M, et al. Effects of Aerobic, Resistance, or Combined Exercise Training among Older Adults with Subjective Memory Complaints: A Randomized Controlled Trial. *Journal of Alzheimer's Disease*. 2021;82(2):701-717. doi:10.3233/JAD-210047
85. Brydges CR, Liu-Ambrose T, Bielak AAM. Using intraindividual variability as an indicator of cognitive improvement in a physical exercise intervention of older women with mild cognitive impairment. *Neuropsychology*. 2020;34(8):825-834. doi:10.1037/neu0000638
86. Morris JK, Vidoni ED, Johnson DK, et al. Aerobic exercise for Alzheimer's disease: A randomized controlled pilot trial. *PLoS One*. 2017;12(2):1-14. doi:10.1371/journal.pone.0170547
87. Wei X hong, Ji L li. Effect of handball training on cognitive ability in elderly with mild cognitive impairment. *Neurosci Lett*. 2014;566:98-101. doi:10.1016/j.neulet.2014.02.035
88. Damirchi A, Hosseini F, Babaei P. Mental Training Enhances Cognitive Function and BDNF More Than Either Physical or Combined Training in Elderly Women With MCI: A Small-Scale Study. *Am J Alzheimers Dis Other Demen*. 2018;33(1):20-29. doi:10.1177/1533317517727068

89. Nakatsuka M, Nakamura K, Hamanosono R, et al. A Cluster Randomized Controlled Trial of Nonpharmacological Interventions for Old-Old Subjects with a Clinical Dementia Rating of 0.5: The Kurihara Project. *Dement Geriatr Cogn Dis Extra*. 2015;5(2):221-232. doi:10.1159/000380816
90. Kohanpour MA, Peeri M, Azarbayjani MA. The effects of aerobic exercise with lavender essence use on cognitive state and serum brain-derived neurotrophic factor levels in elderly with mild cognitive impairment. *Journal of HerbMed Pharmacology*. 2017;6(2):80-84.
91. Karthikeyan T. Therapeutic effects of home-based exercise of geriatrics for the management of cognitive impairment. *ES J Public Health*. 2020;1(1):1003. www.escientificlibrary.com
92. Krootnark K, Chaikeeree N, Saengsirisuwan V, Boonsinsukh R. Effects of low-intensity home-based exercise on cognition in older persons with mild cognitive impairment: a direct comparison of aerobic versus resistance exercises using a randomized controlled trial design. *Front Med (Lausanne)*. 2024;11(June):1-11. doi:10.3389/fmed.2024.1392429
93. Liu IT, Lee WJ, Lin SY, Chang ST, Kao CL, Cheng YY. Therapeutic Effects of Exercise Training on Elderly Patients With Dementia: A Randomized Controlled Trial. *Arch Phys Med Rehabil*. 2020;101(5):762-769. doi:10.1016/j.apmr.2020.01.012
94. Dillon K, Prapavessis H. REducing SEDENTary behavior among mild to moderate cognitively impaired assisted living residents: A pilot randomized controlled trial (RESEDENT study). *J Aging Phys Act*. 2021;29(1):27-35. doi:10.1123/JAPA.2019-0440
95. Yang SY, Shan CL, Qing H, et al. The Effects of Aerobic Exercise on Cognitive Function of Alzheimer's Disease Patients. *CNS Neurol Disord Drug Targets*. 2015;14(10):1292-1297. doi:10.2174/1871527315666151111123319
96. Choi W, Lee S. Ground kayak paddling exercise improves postural balance, muscle performance, and cognitive function in older adults with mild cognitive impairment: A randomized controlled trial. *Medical Science Monitor*. 2018;24:3909-3915. doi:10.12659/MSM.908248
97. Choi W, Lee S. The effects of virtual kayak paddling exercise on postural balance, muscle performance, and cognitive function in older adults with mild cognitive impairment: A randomized controlled trial. *J Aging Phys Act*. 2019;27(6):861-870. doi:10.1123/japa.2018-0020
98. Yu DJ, Yu AP, Bernal JDK, et al. Effects of exercise intensity and frequency on improving cognitive performance in middle-aged and older adults with mild cognitive impairment: A pilot randomized controlled trial on the minimum physical activity recommendation from WHO. *Front Physiol*. 2022;13(September):1-12. doi:10.3389/fphys.2022.1021428
99. Fischbacher M, Chocano-Bedoya PO, Meyer U, et al. Safety and feasibility of a Dalcroze eurhythmics and a simple home exercise program among older adults with mild cognitive impairment (MCI) or mild dementia: The MOVE for your MIND pilot trial. *Pilot Feasibility Stud*. 2020;6(1):1-8. doi:10.1186/s40814-020-00645-7
100. Khattak HG, Ahmad Z, Arshad H, Anwar K. Effect of aerobic exercise on cognition in elderly persons with mild cognitive impairment. *Rawal Medical Journal*. 2022;47(3):698-701. doi:10.5455/rmj.20210713072242
101. Varela S, Ayán C, Cancela JM, Martín V. Effects of two different intensities of aerobic exercise on elderly people with mild cognitive impairment: A randomized pilot study. *Clin Rehabil*. 2012;26(5):442-450. doi:10.1177/0269215511425835
102. Miu D, Edin F, Szeto S, Mak Y. A randomised controlled trial on the effect of exercise on physical, cognitive and affective function in dementia subjects. *Asian Journal of Gerontology & Geriatrics*. 2008;3(1):8-16.
103. Arcoverde C, Deslandes A, Moraes H, et al. Treadmill training as an augmentation treatment for Alzheimer's disease: A pilot randomized controlled study. *Arq Neuropsiquiatr*. 2014;72(3):190-196. doi:10.1590/0004-282X20130231

104. Angiolillo A, Leccese D, Ciccotelli S, et al. Effects of Nordic walking in Alzheimer's disease: A single-blind randomized controlled clinical trial. *Heliyon*. 2023;9(5):e15865. doi:10.1016/j.heliyon.2023.e15865
105. Enette L, Vogel T, Merle S, et al. Effect of 9 weeks continuous vs. interval aerobic training on plasma BDNF levels, aerobic fitness, cognitive capacity and quality of life among seniors with mild to moderate Alzheimer's disease: A randomized controlled trial. *European Review of Aging and Physical Activity*. 2020;17(1):1-16. doi:10.1186/s11556-019-0234-1
106. Phoemsapthawee et al. The Benefit of Arm Swing Exercise on Cognitive Performance in Older Women with Mild Cognitive Impairment. *Journal of Exercise Physiology*. 2016;8(1):11-25.
107. Eggermont LHP, Swaab DF, Hol EM, Scherder EJA. Walking the line: A randomised trial on the effects of a short term walking programme on cognition in dementia. *J Neurol Neurosurg Psychiatry*. 2009;80(7):802-804. doi:10.1136/jnnp.2008.158444
108. Lowery D, Cerga-Pashoja A, Iliffe S, et al. The effect of exercise on behavioural and psychological symptoms of dementia: The EVIDEM-E randomised controlled clinical trial. *Int J Geriatr Psychiatry*. 2014;29(8):819-827. doi:10.1002/gps.4062
109. Guzel I, Can F. The effects of different exercise types on cognitive and physical functions in dementia patients: A randomized comparative study. *Arch Gerontol Geriatr*. 2024;119(18):105321. doi:10.1016/j.archger.2023.105321
110. Venturelli M, Scarsini R, Schena F. Six-month walking program changes cognitive and ADL performance in patients with Alzheimer. *Am J Alzheimers Dis Other Demen*. 2011;26(5):381-388. doi:10.1177/1533317511418956
111. Scherder EJA, Van Paasschen J, Deijen JB, et al. Physical activity and executive functions in the elderly with mild cognitive impairment. *Aging Ment Health*. 2005;9(3):272-280. doi:10.1080/13607860500089930
112. Amjad I, Toor H, Niazi IK, et al. Therapeutic effects of aerobic exercise on EEG parameters and higher cognitive functions in mild cognitive impairment patients. *International Journal of Neuroscience*. 2019;129(6):551-562. doi:10.1080/00207454.2018.1551894
113. Abbas RL, Saab IM, Al-Sharif HK, Naja N, El-Khatib A. Effect of Adding Motorized Cycle Ergometer Over Exercise Training on Balance in Older Adults with Dementia: A Randomized Controlled Trial. *Exp Aging Res*. 2023;49(2):100-111. doi:10.1080/0361073X.2022.2046947
114. Abd El-Kader SM, Al-Jiffri OH. Aerobic exercise improves quality of life, psychological well-being and systemic inflammation in subjects with alzheimer's disease. *Afr Health Sci*. 2016;16(4):1045-1055. doi:10.4314/ahs.v16i4.22
115. L.F. Law et al. Effects of functional task exercise on everyday problem-solving ability and functional status in older adults with mild cognitive impairment—a randomised controlled trial. *Age Ageing*. 2021;51(7):1-11. doi:10.1093/ageing/afac144
116. Law LLF, Mok VCT, Yau MMK. Effects of functional tasks exercise on cognitive functions of older adults with mild cognitive impairment: A randomized controlled pilot trial. *Alzheimers Res Ther*. 2019;11(1). doi:10.1186/s13195-019-0548-2
117. Cancela JM, Ayán C, Varela S, Seijo M. Effects of a long-term aerobic exercise intervention on institutionalized patients with dementia. *J Sci Med Sport*. 2016;19(4):293-298. doi:10.1016/j.jsams.2015.05.007
118. Yu F, Salisbury D, Mathiason MA. Inter-individual differences in the responses to aerobic exercise in Alzheimer's disease: Findings from the FIT-AD trial. *J Sport Health Sci*. 2021;10(1):65-72. doi:10.1016/j.jshs.2020.05.007
119. Yu F, Vock DM, Zhang L, et al. Cognitive Effects of Aerobic Exercise in Alzheimer's Disease: A Pilot Randomized Controlled Trial. *Journal of Alzheimer's Disease*. 2021;80(1):233-244. doi:10.3233/JAD-201100

120. Salisbury D, Mathiason MA, Yu F. Exercise Dose and Aerobic Fitness Response in Alzheimer's Dementia: Findings from the FIT-AD Trial. *Int J Sports Med*. 2022;43(10):850-858. doi:10.1055/a-1639-2307
121. Baker LD, Pa JA, Katula JA, et al. Effects of exercise on cognition and Alzheimer's biomarkers in a randomized controlled trial of adults with mild cognitive impairment: The EXERT study. *Alzheimer's and Dementia*. 2025;21(4):1-17. doi:10.1002/alz.14586
122. Shadyab AH, Aslanyan V, Jacobs DM, et al. Effects of exercise versus usual care on older adults with amnestic mild cognitive impairment: EXERT versus ADNI. *Alzheimer's and Dementia*. 2025;21(4):1-14. doi:10.1002/alz.70118
123. Huang X, Zhang S, Zhao X, et al. Feasibility and effects of remotely supervised aerobic training and resistance training in older adults with mild cognitive impairment: A pilot three-arm randomised controlled trial. *Gen Psychiatr*. 2025;38(2). doi:10.1136/gpsych-2024-101858
124. Fernandez-Gonzalo R, Fernandez-Gonzalo S, Turon M, Prieto C, Tesch PA, García-Carreira MDC. Muscle, functional and cognitive adaptations after flywheel resistance training in stroke patients: A pilot randomized controlled trial. *J Neuroeng Rehabil*. 2016;13(1):1-11. doi:10.1186/s12984-016-0144-7
125. Singh et al. The Study of Mental and Resistance Training (SMART) Study-Resistance Training and/or Cognitive Training in Mild Cognitive Impairment: A Randomized, Double-Blind, Double-Sham Controlled Trial. *J Am Med Dir Assoc*. 2014;15(12):873-880. doi:10.1016/j.jamda.2014.09.010
126. Lv J, Liu Y. Effects of momentum-based dumbbell training on motor control in older adults with mild cognitive impairment. *Chinese Journal of Rehabilitation Medicine*. 2019;34(5):544-550. doi:10.3969/j.issn.1001-1242.2019.05.009
127. Wang L, Wu B, Tao H, et al. Effects and mediating mechanisms of a structured limbs-exercise program on general cognitive function in older adults with mild cognitive impairment: A randomized controlled trial. *Int J Nurs Stud*. 2020;110:103706. doi:10.1016/j.ijnurstu.2020.103706
128. Vints WAJ, Gökçe E, Šeikinaitė J, et al. Resistance training's impact on blood biomarkers and cognitive function in older adults with low and high risk of mild cognitive impairment: a randomized controlled trial. *European Review of Aging and Physical Activity*. 2024;21(1):1-15. doi:10.1186/s11556-024-00344-9
129. Yoon DH, Lee JY, Song W. Effects of Resistance Exercise Training on Cognitive Function and Physical Performance in Cognitive Frailty: A Randomized Controlled Trial. *Journal of Nutrition, Health and Aging*. 2018;22(8):944-951. doi:10.1007/s12603-018-1090-9
130. Yoon DH, Kang D, Kim HJ, Kim JS, Song HS, Song W. Effect of elastic band-based high-speed power training on cognitive function, physical performance and muscle strength in older women with mild cognitive impairment. *Geriatr Gerontol Int*. 2017;17(5):765-772. doi:10.1111/ggi.12784
131. Lee DW, Yoon DH, Lee JY, Panday SB, Park J, Song W. Effects of High-Speed Power Training on Neuromuscular and Gait Functions in Frail Elderly with Mild Cognitive Impairment Despite Blunted Executive Functions: A Randomized Controlled Trial. *Journal of Frailty and Aging*. 2020;9(3):179-184. doi:10.14283/jfa.2020.23
132. Hong SG, Kim JH, Jun TW. Effects of 12-week resistance exercise on electroencephalogram patterns and cognitive function in the elderly with mild cognitive impairment: A randomized controlled trial. *Clinical Journal of Sport Medicine*. 2018;28(6):500-508. doi:10.1097/JSM.0000000000000476
133. Venturelli M, Lanza M, Muti E, Schena F. Positive effects of physical training in activity of daily living-dependent older adults. *Exp Aging Res*. 2010;36(2):190-205. doi:10.1080/03610731003613771
134. Holthoff VA, Marschner K, Scharf M, et al. Effects of physical activity training in patients with alzheimer's dementia: Results of a pilot RCT study. *PLoS One*. 2015;10(4):1-11. doi:10.1371/journal.pone.0121478
135. Baek JE, Hyeon SJ, Kim M, Cho HY, Hahm SC. Effects of dual-task resistance exercise on cognition, mood, depression, functional fitness, and activities of

daily living in older adults with cognitive impairment: a single-blinded, randomized controlled trial. *BMC Geriatr*. 2024;24(1):1-12. doi:10.1186/s12877-024-04942-1

136. Kušleikienė S, Ziv G, Vints WAJ, et al. Cognitive gains and cortical thickness changes after 12 weeks of resistance training in older adults with low and high risk of mild cognitive impairment: Findings from a randomized controlled trial. *Brain Res Bull*. 2025;222(September 2024). doi:10.1016/j.brainresbull.2025.111249
137. Yang JG, Thapa N, Park HJ, et al. Virtual Reality and Exercise Training Enhance Brain, Cognitive, and Physical Health in Older Adults with Mild Cognitive Impairment. *Int J Environ Res Public Health*. 2022;19(20). doi:10.3390/ijerph192013300
138. Doi T, Makizako H, Shimada H, et al. Effects of multicomponent exercise on spatial-temporal gait parameters among the elderly with amnestic mild cognitive impairment (aMCI): Preliminary results from a randomized controlled trial (RCT). *Arch Gerontol Geriatr*. 2013;56(1):104-108. doi:10.1016/j.archger.2012.09.003
139. Uemura K, Doi T, Shimada H, et al. Effects of Exercise Intervention on Vascular Risk Factors in Older Adults with Mild Cognitive Impairment: A Randomized Controlled Trial. *Dement Geriatr Cogn Dis Extra*. 2012;2(1):445-455. doi:10.1159/000343486
140. Suzuki T, Shimada H, Makizako H, et al. A Randomized Controlled Trial of Multicomponent Exercise in Older Adults with Mild Cognitive Impairment. *PLoS One*. 2013;8(4). doi:10.1371/journal.pone.0061483
141. Li L, Liu M, Zeng H, Pan L. Multi-component exercise training improves the physical and cognitive function of the elderly with mild cognitive impairment: A six-month randomized controlled trial. *Ann Palliat Med*. 2021;10(8):8919-8929. doi:10.21037/apm-21-1809
142. Shimada H, Suzuki T, Makizako H, et al. Effects of multicomponent exercise on cognitive function in older adults with amnestic mild cognitive impairment: a randomized controlled trial. *Alzheimer's & Dementia*. 2012;8(4S_Part_4). doi:10.1016/j.jalz.2012.05.386
143. Greblo Jurakic Z, Krizanic V, Sarabon N, Markovic G. Effects of feedback-based balance and core resistance training vs. Pilates training on cognitive functions in older women with mild cognitive impairment: a pilot randomized controlled trial. *Aging Clin Exp Res*. 2017;29(6):1295-1298. doi:10.1007/s40520-017-0740-9
144. Kim J, Yim J. Effects of an exercise protocol for improving handgrip strength and walking speed on cognitive function in patients with chronic stroke. *Medical Science Monitor*. 2017;23:5402-5409. doi:10.12659/MSM.904723
145. Li PWC, Yu DSF, Siu PM, Wong SCK, Chan BS. Peer-supported exercise intervention for persons with mild cognitive impairment: A waitlist randomised controlled trial (the BRAin Vitality Enhancement trial). *Age Ageing*. 2022;51(10):1-10. doi:10.1093/ageing/afac213
146. Avenali M, Picascia M, Tassorelli C, Sinforiani E, Bernini S. Evaluation of the efficacy of physical therapy on cognitive decline at 6-month follow-up in Parkinson disease patients with mild cognitive impairment: a randomized controlled trial. *Aging Clin Exp Res*. 2021;33(12):3275-3284. doi:10.1007/s40520-021-01865-4
147. Mak A, Delbaere K, Refshauge K, et al. Sunbeam Program Reduces Rate of Falls in Long-Term Care Residents With Mild to Moderate Cognitive Impairment or Dementia: Subgroup Analysis of a Cluster Randomized Controlled Trial. *J Am Med Dir Assoc*. 2022;23(5):743-749.e1. doi:10.1016/j.jamda.2022.01.064
148. Sobol NA, Hoffmann K, Frederiksen KS, et al. Effect of aerobic exercise on physical performance in patients with Alzheimer's disease. *Alzheimer's and Dementia*. 2016;12(12):1207-1215. doi:10.1016/j.jalz.2016.05.004
149. Hoffmann K, Sobol NA, Frederiksen KS, et al. Moderate-to-high intensity physical exercise in patients with Alzheimer's disease: A randomized controlled

trial. *Journal of Alzheimer's Disease*. 2016;50(2):443-453. doi:10.3233/JAD-150817
150. Sobol NA, Dall CH, Høgh P, et al. Change in fitness and the relation to change in cognition and neuropsychiatric symptoms after aerobic exercise in patients with mild Alzheimer's disease. *Journal of Alzheimer's Disease*. 2018;65(1):137-145. doi:10.3233/JAD-180253
151. Papatsimpas V, Vrouva S, Papathanasiou G, et al. Does Therapeutic Exercise Support Improvement in Cognitive Function and Instrumental Activities of Daily Living in Patients with Mild Alzheimer's Disease? A Randomized Controlled Trial. *Brain Sci*. 2023;13(7). doi:10.3390/brainsci13071112
152. Ullrich P, Werner C, Schönstein A, et al. Effects of a Home-Based Physical Training and Activity Promotion Program in Community-Dwelling Older Persons with Cognitive Impairment after Discharge from Rehabilitation: A Randomized Controlled Trial. *Journals of Gerontology - Series A Biological Sciences and Medical Sciences*. 2022;77(12):2435-2444. doi:10.1093/gerona/glac005
153. Bademli K, Lok N, Canbaz M, Lok S. Effects of Physical Activity Program on cognitive function and sleep quality in elderly with mild cognitive impairment: A randomized controlled trial. *Perspect Psychiatr Care*. 2019;55(3):401-408. doi:10.1111/ppc.12324
154. Lok N, Tosun AS, Lok S, Temel V, Aydın Z. Effect of physical activity program applied to patients with Alzheimer's disease on cognitive functions and depression level: a randomised controlled study. *Psychogeriatrics*. 2023;23(5):856-863. doi:10.1111/psyg.13010
155. De Sá CA, Saretto CB, Cardoso AM, Remor A, Breda CO, da Silva Corralo V. Effects of a physical exercise or motor activity protocol on cognitive function, lipid profile, and BDNF levels in older adults with mild cognitive impairment. *Mol Cell Biochem*. 2024;479(3):499-509. doi:10.1007/s11010-023-04733-z
156. Padala KP, Padala PR, Lensing SY, et al. Home-Based Exercise Program Improves Balance and Fear of Falling in Community-Dwelling Older Adults with Mild Alzheimer's Disease: A Pilot Study. *Journal of Alzheimer's Disease*. 2017;59(2):565-574. doi:10.3233/JAD-170120
157. Langoni CDS, Resende TDL, Barcellos AB, et al. Effect of Exercise on Cognition, Conditioning, Muscle Endurance, and Balance in Older Adults with Mild Cognitive Impairment: A Randomized Controlled Trial. *Journal of Geriatric Physical Therapy*. 2019;42(2):E15-E22. doi:10.1519/JPT.0000000000000191
158. Langoni C da S, Resende T de L, Barcellos AB, et al. The effect of group exercises on balance, mobility, and depressive symptoms in older adults with mild cognitive impairment: a randomized controlled trial. *Clin Rehabil*. 2019;33(3):439-449. doi:10.1177/0269215518815218
159. Zhang Q, Zhu M, Huang L, et al. A Study on the Effect of Traditional Chinese Exercise Combined With Rhythm Training on the Intervention of Older Adults With Mild Cognitive Impairment. *Am J Alzheimers Dis Other Demen*. 2023;38(48):1-12. doi:10.1177/15333175231190626
160. Vreugdenhil A, Cannell J, Davies A, Razay G. A community-based exercise programme to improve functional ability in people with Alzheimer's disease: A randomized controlled trial. *Scand J Caring Sci*. 2012;26(1):12-19. doi:10.1111/j.1471-6712.2011.00895.x
161. Papamichail P, Sagredaki ML, Bouzineki C, Kanellopoulou S, Lyros E, Christakou A. The Effectiveness of an Exercise Program on Muscle Strength and Range of Motion on Upper Limbs, Functional Ability and Depression at Early Stage of Dementia. *J Clin Med*. 2024;13(14):1-10. doi:10.3390/jcm13144136
162. Rivas-Campo Y, Aibar-Almazán A, Afanador-Restrepo DF, et al. Effects of High-Intensity Functional Training (HIFT) on the Functional Capacity, Frailty, and Physical Condition of Older Adults with Mild Cognitive Impairment: A Blind Randomized Controlled Clinical Trial. *Life*. 2023;13(5):1-16. doi:10.3390/life13051224
163. Rivas-Campo Y, Aibar-Almazán A, Rodríguez-López C, et al. Enhancing Cognition in Older Adults with Mild Cognitive Impairment through High-Intensity Functional Training: A Single-Blind Randomized Controlled Trial. *J Clin Med*. 2023;12(12):1-12. doi:10.3390/jcm12124049
164. Prick AE, De Lange J, Scherder E, Twisk J, Pot AM. The effects of a multicomponent dyadic intervention with physical exercise on the cognitive functioning of people with dementia: A randomized controlled trial. *J Aging Phys Act*. 2017;25(4):539-552. doi:10.1123/japa.2016-0038

165. Hauer K, Schwenk M, Zieschang T, Essig M, Becker C, Oster P. Physical training improves motor performance in people with dementia: A randomized controlled trial. *J Am Geriatr Soc*. 2012;60(1):8-15. doi:10.1111/j.1532-5415.2011.03778.x
166. Zieschang T, Schwenk M, Oster P, Hauer K. Sustainability of motor training effects in older people with dementia. *Journal of Alzheimer's Disease*. 2013;34(1):191-202. doi:10.3233/JAD-120814
167. Schwenk M, Zieschang T, Englert S, Grewal G, Najafi B, Hauer K. Improvements in gait characteristics after intensive resistance and functional training in people with dementia: A randomised controlled trial. *BMC Geriatr*. 2014;14(1):1-9. doi:10.1186/1471-2318-14-73
168. Suttanon P, Hill KD, Said CM, et al. Feasibility, safety and preliminary evidence of the effectiveness of a home-based exercise programme for older people with Alzheimer's disease: A pilot randomized controlled trial. *Clin Rehabil*. 2013;27(5):427-438. doi:10.1177/0269215512460877
169. Sanders LMJ, Hortobágyi T, Karssemeijer EGA, Van Der Zee EA, Scherder EJA, Van Heuvelen MJG. Effects of low- And high-intensity physical exercise on physical and cognitive function in older persons with dementia: A randomized controlled trial. *Alzheimers Res Ther*. 2020;12(1):1-15. doi:10.1186/s13195-020-00597-3
170. Dawson N, Judge KS, Gerhart H. Improved Functional Performance in Individuals with Dementia after a Moderate-Intensity Home-Based Exercise Program: A Randomized Controlled Trial. *Journal of Geriatric Physical Therapy*. 2019;42(1):18-27. doi:10.1519/JPT.0000000000000128
171. Santana-Sosa E, Barriopedro MI, López-Mojares LM, Pérez M, Lucia A. Exercise training is beneficial for Alzheimer's patients. *Int J Sports Med*. 2008;29(10):845-850. doi:10.1055/s-2008-1038432
172. Lamb SE, Sheehan B, Atherton N, et al. Dementia And Physical Activity (DAPA) trial of moderate to high intensity exercise training for people with dementia: Randomised controlled trial. *BMJ (Online)*. 2018;361. doi:10.1136/bmj.k1675
173. Smith TO, Mistry D, Lee H, et al. Moderators of Cognitive Outcomes from an Exercise Program in People with Mild to Moderate Dementia. *J Am Geriatr Soc*. 2020;68(9):2095-2100. doi:10.1111/jgs.16552
174. Kovas et al. Effects of a multimodal exercise program on balance, functional mobility and fall risk in older adults with cognitive impairment: a randomized controlled single-blind study. 2011;47(3):381-390.
175. de Oliveira Silva F, Ferreira JV, Plácido J, et al. Three months of multimodal training contributes to mobility and executive function in elderly individuals with mild cognitive impairment, but not in those with Alzheimer's disease: A randomized controlled trial. *Maturitas*. 2019;126(April):28-33. doi:10.1016/j.maturitas.2019.04.217
176. Levinger P, Goh AMY, Dunn J, et al. Exercise interveNtion outdoor proJect in the cOmmunitY – results from the ENJOY program for independence in dementia: a feasibility pilot randomised controlled trial. *BMC Geriatr*. 2023;23(1):1-16. doi:10.1186/s12877-023-04132-5
177. Ghahfarrokhi MM, Shirvani H, Rahimi M, Bazgir B, Shamsadini A, Sobhani V. Feasibility and preliminary efficacy of different intensities of functional training in elderly type 2 diabetes patients with cognitive impairment: a pilot randomised controlled trial. *BMC Geriatr*. 2024;24(1):1-15. doi:10.1186/s12877-024-04698-8
178. Fonte C, Smania N, Pedrinolla A, et al. Comparison between physical and cognitive treatment in patients with MCI and Alzheimer's disease. *Aging*. 2019;11(10):3138-3155. doi:10.18632/aging.101970
179. Gebhard D, Mess F. Feasibility and Effectiveness of a Biography-Based Physical Activity Intervention in Institutionalized People With Dementia: Quantitative and Qualitative Results From a Randomized Controlled Trial. *J Aging Phys Act*. 2022;30(2):237-251. doi:10.1123/japa.2020-0343

180. Akbuga Koc E, Yazici-Mutlu Ç, Cinar N, Sahiner T. Comparison of the effect of online physical exercise and computerized cognitive stimulation in patients with Alzheimer's disease during the Covid-19 pandemic. *Complement Ther Clin Pract*. 2024;57(May):10-20. doi:10.1016/j.ctcp.2024.101881
181. Casas-Herrero Á, Sáez de Asteasu ML, Antón-Rodrigo I, et al. Effects of Vivifrail multicomponent intervention on functional capacity: a multicentre, randomized controlled trial. *J Cachexia Sarcopenia Muscle*. 2022;13(2):884-893. doi:10.1002/jcsm.12925
182. Shaw I, Cronje M, Shaw BS. Group-based exercise as a therapeutic strategy for the improvement of mental outcomes in mild to moderate alzheimer's patients in low resource care facilities. *Asian J Sports Med*. 2021;12(1):1-6. doi:10.5812/asjsm.106593
183. Cezar NO de C, Ansai JH, Oliveira MPB de, et al. Feasibility of improving strength and functioning and decreasing the risk of falls in older adults with Alzheimer's dementia: a randomized controlled home-based exercise trial. *Arch Gerontol Geriatr*. 2021;96(March). doi:10.1016/j.archger.2021.104476
184. Cezar NO de C, Aprahamian I, Ansai JH, et al. Feasibility of reducing frailty components in older adults with Alzheimer's dementia: a randomized controlled home-based exercise trial (AD-HOMEX). *Exp Gerontol*. 2021;150(May). doi:10.1016/j.exger.2021.111390
185. Mollinedo Cardalda I, López A, Cancela Carral JM. The effects of different types of physical exercise on physical and cognitive function in frail institutionalized older adults with mild to moderate cognitive impairment. A randomized controlled trial. *Arch Gerontol Geriatr*. 2019;83(May):223-230. doi:10.1016/j.archger.2019.05.003
186. Bo W, Lei M, Tao S, et al. Effects of combined intervention of physical exercise and cognitive training on cognitive function in stroke survivors with vascular cognitive impairment: a randomized controlled trial. *Clin Rehabil*. 2019;33(1):54-63. doi:10.1177/0269215518791007
187. Kim MJ, Han CW, Min KY, et al. Physical Exercise with Multicomponent Cognitive Intervention for Older Adults with Alzheimer's Disease: A 6-Month Randomized Controlled Trial. *Dement Geriatr Cogn Dis Extra*. 2016;6(2):222-232. doi:10.1159/000446508
188. Bossers WJR, Van Der Woude LHV, Boersma F, Hortobágyi T, Scherder EJA, Van Heuvelen MJG. A 9-Week Aerobic and Strength Training Program Improves Cognitive and Motor Function in Patients with Dementia: A Randomized, Controlled Trial. *American Journal of Geriatric Psychiatry*. 2015;23(11):1106-1116. doi:10.1016/j.jagp.2014.12.191
189. Bossers WJR, van der Woude LHV, Boersma F, Hortobágyi T, Scherder EJA, van Heuvelen MJG. Comparison of Effect of Two Exercise Programs on Activities of Daily Living in Individuals with Dementia: A 9-Week Randomized, Controlled Trial. *J Am Geriatr Soc*. 2016;64(6):1258-1266. doi:10.1111/jgs.14160
190. Telenius EW, Engedal K, Bergland A. Long-term effects of a 12 weeks high-intensity functional exercise program on physical function and mental health in nursing home residents with dementia: A single blinded randomized controlled trial Physical functioning, physical health and activity. *BMC Geriatr*. 2015;15(1):1-11. doi:10.1186/s12877-015-0151-8
191. Telenius EW, Engedal K, Bergland A. Effect of a high-intensity exercise program on physical function and mental health in nursing home residents with dementia: An assessor blinded randomized controlled trial. *PLoS One*. 2015;10(5):1-18. doi:10.1371/journal.pone.0126102
192. Toots A, Littbrand H, Boström G, et al. Effects of exercise on cognitive function in older people with dementia: A randomized controlled trial. *Journal of Alzheimer's Disease*. 2017;60(1):323-332. doi:10.3233/JAD-170014
193. Boström G, Conradsson M, Hörnsten C, et al. Effects of a high-intensity functional exercise program on depressive symptoms among people with dementia in residential care: a randomized controlled trial. *Int J Geriatr Psychiatry*. 2016;31(8):868-878. doi:10.1002/gps.4401
194. Toots A, Lindelöf N, Littbrand H, et al. Effects of a High-Intensity Functional Exercise Program on Dependence in Activities of Daily Living and Balance in

Older Adults with Dementia. *J Am Geriatr Soc*. 2016;64(1):55-64. doi:10.1111/jgs.13880
195. Toots A, Littbrand H, Holmberg H, et al. Walking Aids Moderate Exercise Effects on Gait Speed in People With Dementia: A Randomized Controlled Trial. *J Am Med Dir Assoc*. 2017;18(3):227-233. doi:10.1016/j.jamda.2016.09.003
196. Toots A, Lundin-Olsson L, Nordström P, Gustafson Y, Rosendahl E. Exercise effects on backward walking speed in people with dementia: A randomized controlled trial. *Gait Posture*. 2021;85(January):65-70. doi:10.1016/j.gaitpost.2020.12.028
197. Henskens M, Nauta IM, Van Eekeren MCA, Scherder EJA. Effects of Physical Activity in Nursing Home Residents with Dementia: A Randomized Controlled Trial. *Dement Geriatr Cogn Disord*. 2018;46(1-2):60-80. doi:10.1159/000491818
198. Kemoun G, Thibaud M, Roumagne N, et al. Effects of a physical training programme on cognitive function and walking efficiency in elderly persons with dementia. *Dement Geriatr Cogn Disord*. 2010;29(2):109-114. doi:10.1159/000272435
199. de Souto Barreto P, Cesari M, Denormandie P, Armaingaud D, Vellas B, Rolland Y. Exercise or Social Intervention for Nursing Home Residents with Dementia: A Pilot Randomized, Controlled Trial. *J Am Geriatr Soc*. 2017;65(9):E123-E129. doi:10.1111/jgs.14947
200. Maltais M, Rolland Y, Vellas B, et al. Effect of Exercise on Behavioral Symptoms and Pain in Patients With Dementia Living in Nursing Homes. *Am J Alzheimers Dis Other Demen*. 2019;34(2):89-94. doi:10.1177/1533317518803773
201. Roach KE, Tappen RM, Kirk-Sanchez N, Williams CL, Loewenstein D. A randomized controlled trial of an activity specific exercise program for individuals with alzheimer disease in long-term care settings. *Journal of Geriatric Physical Therapy*. 2011;34(2):50-56. doi:10.1519/JPT.0b013e31820aab9c
202. Rolland Y, Pillard F, Klapouszczak A, et al. Exercise program for nursing home residents with Alzheimer's disease: A 1-year randomized, controlled trial. *J Am Geriatr Soc*. 2007;55(2):158-165. doi:10.1111/j.1532-5415.2007.01035.x
203. Stevens J, Killeen M. A randomised controlled trial testing the impact of exercise on cognitive symptoms and disability of residents with dementia. *Contemporary nurse : a journal for the Australian nursing profession*. 2006;21(1):32-40. doi:10.5172/conu.2006.21.1.32
204. Brett L, Stapley P, Meedya S, Traynor V. Effect of physical exercise on physical performance and fall incidents of individuals living with dementia in nursing homes: a randomized controlled trial. *Physiother Theory Pract*. 2021;37(1):38-51. doi:10.1080/09593985.2019.1594470
205. Almeida S, Paixão C, da Silva MG, Marques A. Lifestyle-integrated functional exercise for people with Dementia: A pilot study. *J Aging Phys Act*. 2021;29(5):771-780. doi:10.1123/JAPA.2020-0349
206. Verdelho A, Correia M, Gonçalves-Pereira M, et al. Physical Activity in Mild Vascular Cognitive Impairment: Results of the AFIVASC Randomized Controlled Trial at 6 Months. *Journal of Alzheimer's Disease*. 2024;101(4):1379-1392. doi:10.3233/JAD-240246
207. Yan Y, Xu Y, Wang X, et al. The effect of multi-component exercise intervention in older people with Parkinson's disease and mild cognitive impairment: A randomized controlled study. *Geriatr Nurs (Minneap)*. 2024;60:137-145. doi:10.1016/j.gerinurse.2024.08.028
208. David S, Costa AS, Hohenfeld C, et al. Modulating effects of fitness and physical activity on Alzheimer's disease: Implications from a six-month randomized controlled sports intervention. *Journal of Alzheimer's Disease*. 2025;103(2):552-569. doi:10.1177/13872877241303764
209. Shokri G, Mohammadian F, Noroozian M, Amani-Shalamzari S, Suzuki K. Effects of remote combine exercise-music training on physical and cognitive performance in patients with Alzheimer's disease: a randomized controlled trial. *Front Aging Neurosci*. 2023;15(January):1-9. doi:10.3389/fnagi.2023.1283927
