# Supplementary material for: Dance versus other exercise modalities in mild cognitive impairment and dementia: comparative efficacy from a systematic review and bayesian network meta-analysis
Source: Front Physiol. 2026 Mar 25;17:1782774. doi: 10.3389/fphys.2026.1782774 (PMC13056856; doi:10.3389/fphys.2026.1782774)
Supplement: Supplementary file 5 [file Table9.pdf]

Supplementary Table 9. The Inconsistency Tests Results

1. **The Mini-Mental State Examination (MMSE)** outcome includes dance[1–4], exergaming[5–7], CTE[8–13], AE[14–27], RE[28–31], and ME[32–52].

Inconsistency analysis

Local inconsistency

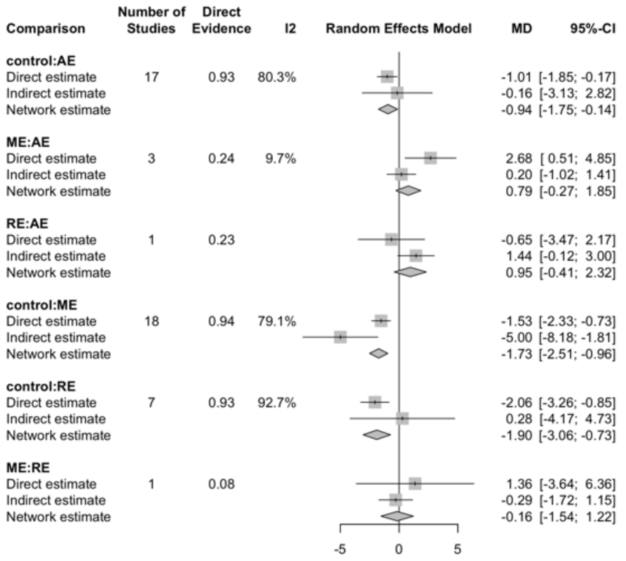

Global inconsistency

```
> print(global_inconsistency)
Q statistics to assess homogeneity / consistency

                    Q df  p-value
Total          299.67 48 < 0.0001
Within designs 280.47 42 < 0.0001
Between designs 19.20  6   0.0038

Design-specific decomposition of within-designs Q statistic

             Design     Q df  p-value
         control:RE 71.12  4 < 0.0001
         AE:control 76.70 12 < 0.0001
         control:ME 76.03 13 < 0.0001
        control:CTE 33.97  4 < 0.0001
      control:Dance 11.62  3   0.0088
  control:Exergaming  6.06  2   0.0484
      AE:control:ME  4.96  4   0.2912

Between-designs Q statistic after detaching of single designs
(influential designs have p-value markedly different from 0.0038)

 Detached design     Q df p-value
   AE:control:RE  8.67  4  0.0699
      control:RE 10.52  5  0.0619
      control:ME 13.81  5  0.0168
   AE:control:ME 13.29  4  0.0099
   control:ME:RE 15.88  4  0.0032
      AE:control 19.20  5  0.0018

Q statistic to assess consistency under the assumption of
a full design-by-treatment interaction random effects model

                  Q df p-value tau.within tau2.within
Between designs 9.05  6  0.1711     1.4611      2.1349
```

Bayesian NMA inconsistency model

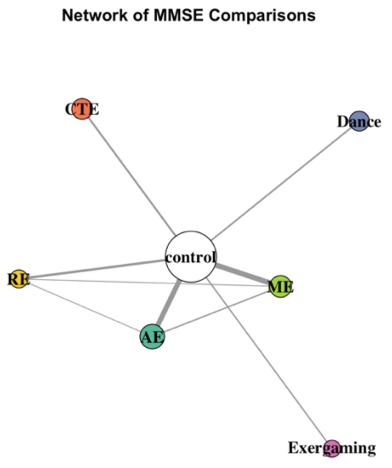

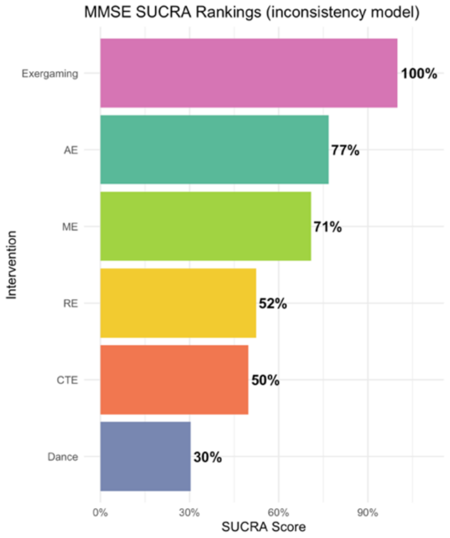

	AE	control	CTE	Dance	Exergaming	ME	RE	Yoga
**AE**	—	****1.84 [0.86, 2.77]**	-0.34 [-1.69, 0.99]	-0.05 [-1.53, 1.41]	0.34 [-1.54, 2.27]	-0.25 [-1.71, 1.25]	0.03 [-1.68, 1.79]	0.18 [-2.06, 2.44]
**control**	****-1.84 [-2.77, -0.86]**	—	****-2.17 [-3.25, -1.08]**	****-1.88 [-3.08, -0.68]**	-1.49 [-3.12, 0.24]	****-2.09 [-3.26, -0.86]**	****-1.79 [-3.33, -0.25]**	-1.65 [-3.68, 0.45]
**CTE**	0.34 [-0.99, 1.69]	****2.17 [1.08, 3.25]**	—	0.29 [-1.32, 1.90]	0.68 [-1.17, 2.61]	0.09 [-1.42, 1.63]	0.39 [-1.49, 2.27]	0.52 [-1.73, 2.86]
**Dance**	0.05 [-1.41, 1.53]	****1.88 [0.68, 3.08]**	-0.29 [-1.90, 1.32]	—	0.39 [-1.62, 2.52]	-0.20 [-1.87, 1.50]	0.09 [-1.88, 2.04]	0.23 [-2.09, 2.62]
**Exergaming**	-0.34 [-2.27, 1.54]	1.49 [-0.24, 3.12]	-0.68 [-2.61, 1.17]	-0.39 [-2.52, 1.62]	—	-0.59 [-2.62, 1.40]	-0.31 [-2.59, 1.90]	-0.15 [-2.80, 2.45]
**ME**	0.25 [-1.25, 1.71]	****2.09 [0.86, 3.26]**	-0.09 [-1.63, 1.42]	0.20 [-1.50, 1.87]	0.59 [-1.40, 2.62]	—	0.29 [-1.66, 2.22]	0.43 [-1.95, 2.78]
**RE**	-0.03 [-1.79, 1.68]	****1.79 [0.25, 3.33]**	-0.39 [-2.27, 1.49]	-0.09 [-2.04, 1.88]	0.31 [-1.90, 2.59]	-0.29 [-2.22, 1.66]	—	0.14 [-2.39, 2.73]
**Yoga**	-0.18 [-2.44, 2.06]	1.65 [-0.45, 3.68]	-0.52 [-2.86, 1.73]	-0.23 [-2.62, 2.09]	0.15 [-2.45, 2.80]	-0.43 [-2.78, 1.95]	-0.14 [-2.73, 2.39]	—

Notes: Values represent standardized mean differences (SMDs) with 95% credible intervals. Bolded values with double asterisks ** indicate statistically significant differences at the 95% level, where the credible interval does not include zero.

2. **The Montreal Cognitive Assessment (MoCA)** outcome includes dance[2,3,53–57], exergaming[58–61], yoga[62,63], CTE[10,12,64–68], AE[19,21,69–74], RE[28,75,76], and ME[33,34,43,77–81].

Inconsistency analysis

Local inconsistency

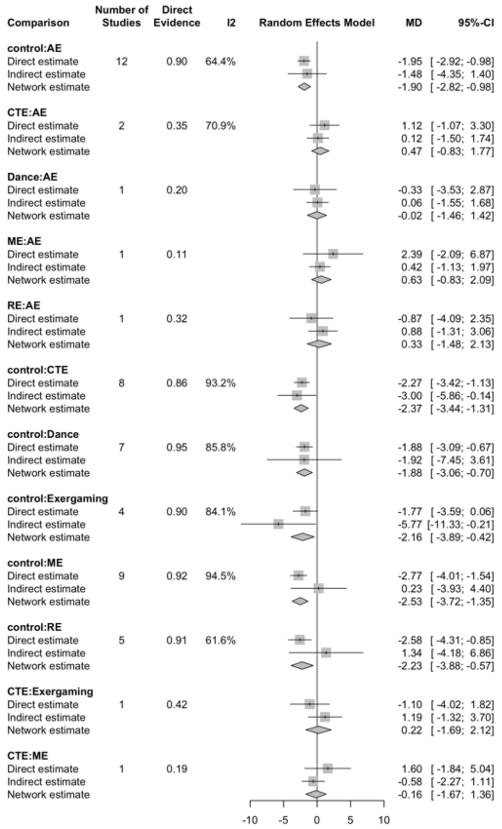

Global inconsistency

```
> print(global_inconsistency)
Q statistics to assess homogeneity / consistency

                    Q df  p-value
Total          357.10 40 < 0.0001
Within designs 235.99 28 < 0.0001
Between designs 121.11 12 < 0.0001

Design-specific decomposition of within-designs Q statistic

             Design      Q df  p-value
         control:ME 138.11  6 < 0.0001
      control:Dance  41.78  5 < 0.0001
 control:Exergaming  18.87  2 < 0.0001
         AE:control  16.30  6   0.0122
         control:RE   7.89  3   0.0483
     AE:control:CTE   5.24  2   0.0726
        control:CTE   6.62  3   0.0851
       control:Yoga   1.17  1   0.2788

Between-designs Q statistic after detaching of single designs
(influential designs have p-value markedly different from < 0.0001)

        Detached design      Q df  p-value
 control:CTE:Exergaming  29.15 10   0.0012
            control:CTE  65.47 11 < 0.0001
         AE:control:CTE 103.27 10 < 0.0001
         control:CTE:ME 105.12 10 < 0.0001
             AE:control 112.91 11 < 0.0001
          AE:control:RE 113.45 10 < 0.0001
     control:Exergaming 120.65 11 < 0.0001
          control:Dance 120.98 11 < 0.0001
             control:ME 121.05 11 < 0.0001
             control:RE 121.10 11 < 0.0001
          AE:control:ME 118.86 10 < 0.0001
       AE:control:Dance 120.72 10 < 0.0001

Q statistic to assess consistency under the assumption of
a full design-by-treatment interaction random effects model

                   Q df p-value tau.within tau2.within
Between designs 9.18 12  0.6878     1.7024      2.8982
```

Bayesian NMA inconsistency model

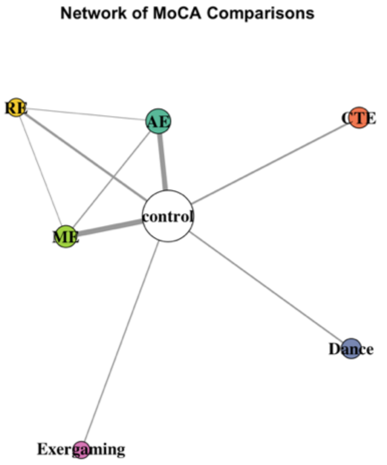

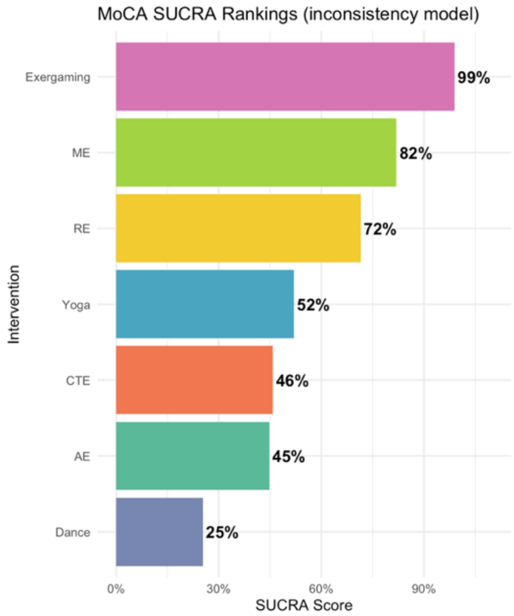

	AE	control	CTE	Dance	Exergaming	ME	RE	Yoga
**AE**	—	****1.83 [0.87, 2.78]**	-0.34 [-1.69, 0.99]	-0.04 [-1.56, 1.42]	0.36 [-1.51, 2.33]	-0.25 [-1.70, 1.26]	0.03 [-1.72, 1.82]	0.18 [-2.04, 2.45]
**control**	****-1.83 [-2.78, -0.87]**	—	****-2.17 [-3.27, -1.08]**	****-1.88 [-3.10, -0.67]**	-1.47 [-3.10, 0.30]	****-2.07 [-3.24, -0.85]**	****-1.80 [-3.36, -0.19]**	-1.65 [-3.68, 0.40]
**CTE**	0.34 [-0.99, 1.69]	****2.17 [1.08, 3.27]**	—	0.29 [-1.34, 1.91]	0.71 [-1.17, 2.69]	0.10 [-1.44, 1.67]	0.37 [-1.52, 2.29]	0.52 [-1.78, 2.82]
**Dance**	0.04 [-1.42, 1.56]	****1.88 [0.67, 3.10]**	-0.29 [-1.91, 1.34]	—	0.41 [-1.62, 2.56]	-0.19 [-1.85, 1.54]	0.08 [-1.88, 2.10]	0.24 [-2.11, 2.65]
**Exergaming**	-0.36 [-2.33, 1.51]	1.47 [-0.30, 3.10]	-0.71 [-2.69, 1.17]	-0.41 [-2.56, 1.62]	—	-0.60 [-2.69, 1.37]	-0.33 [-2.64, 1.90]	-0.19 [-2.83, 2.45]
**ME**	0.25 [-1.26, 1.70]	****2.07 [0.85, 3.24]**	-0.10 [-1.67, 1.44]	0.19 [-1.54, 1.85]	0.60 [-1.37, 2.69]	—	0.27 [-1.66, 2.23]	0.43 [-1.94, 2.78]
**RE**	-0.03 [-1.82, 1.72]	****1.80 [0.19, 3.36]**	-0.37 [-2.29, 1.52]	-0.08 [-2.10, 1.88]	0.33 [-1.90, 2.64]	-0.27 [-2.23, 1.66]	—	0.15 [-2.43, 2.74]
**Yoga**	-0.18 [-2.45, 2.04]	1.65 [-0.40, 3.68]	-0.52 [-2.82, 1.78]	-0.24 [-2.65, 2.11]	0.19 [-2.45, 2.83]	-0.43 [-2.78, 1.94]	-0.15 [-2.74, 2.43]	—

Notes: Values represent standardized mean differences (SMDs) with 95% credible intervals. Bolded values with double asterisks ** indicate statistically significant differences at the 95% level, where the credible interval does not include zero.

3. **The Trail Making Test-A (TMT-A)** outcome includes dance[1,2,55,82], exergaming[6,58,59], yoga[62], CTE[67,83], AE[17,21,72,74], RE[84,85], and ME[32,34,44,48,78,79,86].

Local inconsistency

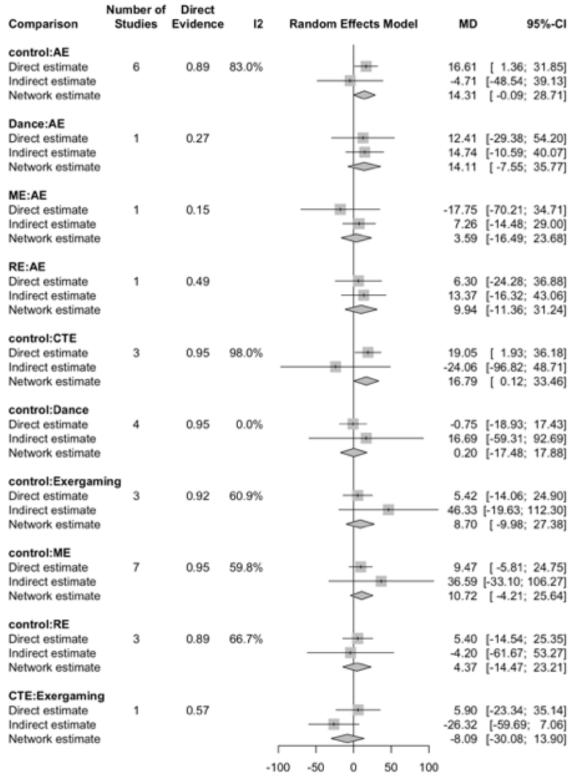

Global inconsistency

```
> print(global_inconsistency)
Q statistics to assess homogeneity / consistency

                     Q df  p-value
Total           157.05 20 < 0.0001
Within designs  103.00 12 < 0.0001
Between designs  54.05  8 < 0.0001

Design-specific decomposition of within-designs Q statistic

          Design     Q df  p-value
     control:CTE 61.57  1 < 0.0001
      AE:control 26.60  2 < 0.0001
      control:ME 14.32  5   0.0137
      control:RE  0.21  1   0.6505
   control:Dance  0.28  2   0.8686
 control:Exergaming  0.02  1   0.8882

Between-designs Q statistic after detaching of single designs
(influential designs have p-value markedly different from < 0.0001)

     Detached design     Q df  p-value
 control:CTE:Exergaming  8.50  6   0.2040
         control:CTE 13.59  7   0.0589
  control:Exergaming 48.58  7 < 0.0001
          control:RE 50.30  7 < 0.0001
       AE:control:RE 48.23  6 < 0.0001
          control:ME 52.42  7 < 0.0001
          AE:control 53.86  7 < 0.0001
       control:Dance 53.92  7 < 0.0001
       AE:control:ME 52.32  6 < 0.0001
    AE:control:Dance 53.15  6 < 0.0001

Q statistic to assess consistency under the assumption of
a full design-by-treatment interaction random effects model

                   Q df p-value tau.within tau2.within
Between designs 3.26  8  0.9172    19.0417    362.5874
```

4. **The Trail Making Test-B (TMT-B)** outcome includes dance[1,2,55,82], exergaming[6,58–60], yoga[62,87], CTE[64,65,67,83], AE[21,72,74], RE[84,85,88], and ME[34,78,79,86,89,90].

Inconsistency analysis

Local inconsistency

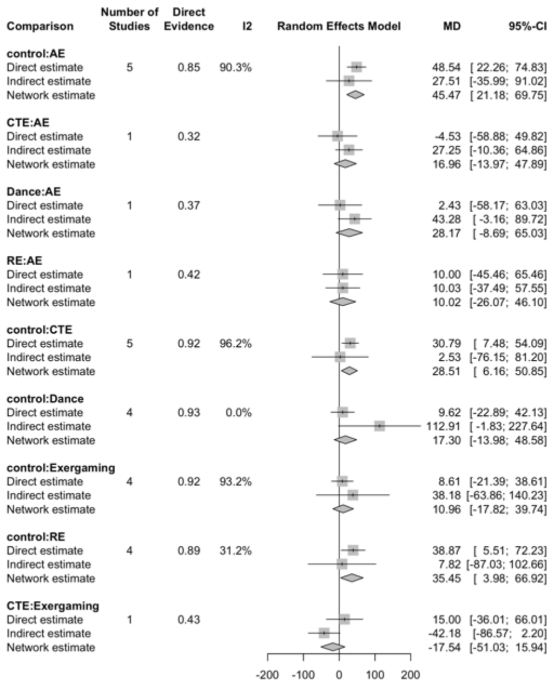

Global inconsistency

```
> print(global_inconsistency)
Q statistics to assess homogeneity / consistency

                     Q df  p-value
Total           210.36 23 < 0.0001
Within designs  112.36 15 < 0.0001
Between designs  98.00  8 < 0.0001

Design-specific decomposition of within-designs Q statistic

             Design     Q df  p-value
        control:CTE 83.43  2 < 0.0001
         control:ME 16.90  5   0.0047
       control:Yoga  5.17  1   0.0229
         AE:control  2.28  1   0.1314
 control:Exergaming  2.62  2   0.2696
         control:RE  1.88  2   0.3903
      control:Dance  0.08  2   0.9623

Between-designs Q statistic after detaching of single designs
(influential designs have p-value markedly different from < 0.0001)

      Detached design     Q df  p-value
 control:CTE:Exergaming 43.10  6 < 0.0001
     control:Exergaming 61.37  7 < 0.0001
             AE:control 70.06  7 < 0.0001
            control:CTE 81.15  7 < 0.0001
         AE:control:CTE 83.04  6 < 0.0001
       AE:control:Dance 86.62  6 < 0.0001
             control:RE 97.41  7 < 0.0001
          control:Dance 98.00  7 < 0.0001
          AE:control:RE 95.20  6 < 0.0001

Q statistic to assess consistency under the assumption of
a full design-by-treatment interaction random effects model

                    Q df p-value tau.within tau2.within
Between designs 12.82  8  0.1181    26.2609    689.6364
```

5. **The Timed Up and Go (TUG)** outcome includes dance[1,53,91], exergaming[5,92], yoga[62], CTE[9,12,93–95], AE[22,72,73,96–98], RE[28,84,85,88], and ME[36,41,44,45,48,52,81,99–104].

Inconsistency analysis

Local inconsistency

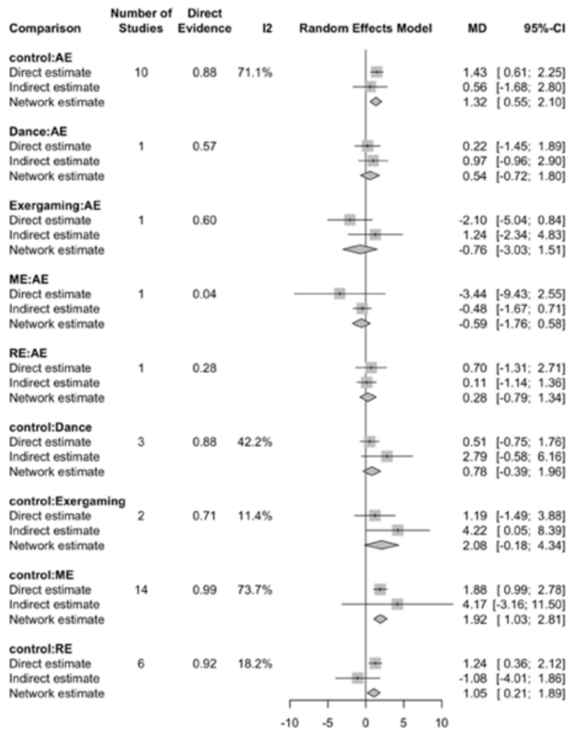

Global inconsistency

```
> print(global_inconsistency)
Q statistics to assess homogeneity / consistency

                    Q df  p-value
Total          127.00 34 < 0.0001
Within designs 103.55 26 < 0.0001
Between designs  23.45  8   0.0028

Design-specific decomposition of within-designs Q statistic

       Design     Q df  p-value
  control:CTE 40.71  4 < 0.0001
   control:ME 45.96 12 < 0.0001
   AE:control 14.20  5   0.0144
control:Dance  1.21  1   0.2717
   control:RE  1.48  4   0.8301

Between-designs Q statistic after detaching of single designs
(influential designs have p-value markedly different from 0.0028)

        Detached design     Q df p-value
          AE:control:RE 13.36  6  0.0376
       AE:control:Dance 14.47  6  0.0248
             control:ME 19.77  7  0.0061
          AE:control:ME 19.24  6  0.0038
             AE:control 21.02  7  0.0037
     control:Exergaming 21.71  7  0.0028
          control:Dance 22.75  7  0.0019
             control:RE 23.08  7  0.0017
  AE:control:Exergaming 21.31  6  0.0016

Q statistic to assess consistency under the assumption of
a full design-by-treatment interaction random effects model

                    Q df p-value tau.within tau2.within
Between designs 11.94  8  0.1541     0.7815      0.6107
```

6. **The Digit Span Forward (DSF)** outcome includes dance[82], CTE[8,64,66], AE[72,96,105], RE[76,84,88], and ME[44,48,52,90,106,107].

Inconsistency analysis

Local inconsistency

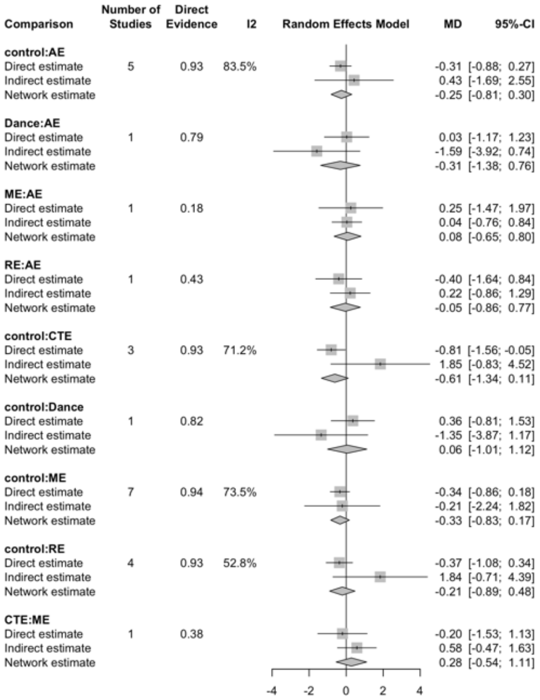

Global inconsistency

```
> print(global_inconsistency)
Q statistics to assess homogeneity / consistency

                    Q df  p-value
Total           51.38 15 < 0.0001
Within designs  15.25  8   0.0545
Between designs 36.13  7 < 0.0001

Design-specific decomposition of within-designs Q statistic

      Design    Q df p-value
  AE:control 4.38  1  0.0364
  control:ME 7.99  4  0.0921
 control:CTE 1.80  1  0.1795
  control:RE 1.08  2  0.5819

Between-designs Q statistic after detaching of single designs
(influential designs have p-value markedly different from < 0.0001)

 Detached design     Q df  p-value
   AE:control:RE 17.69  5   0.0034
  control:CTE:ME 21.68  5   0.0006
      control:ME 27.29  6   0.0001
      control:RE 34.72  6 < 0.0001
     control:CTE 35.85  6 < 0.0001
      AE:control 35.95  6 < 0.0001
   AE:control:ME 35.90  5 < 0.0001

Q statistic to assess consistency under the assumption of
a full design-by-treatment interaction random effects model

                    Q df p-value tau.within tau2.within
Between designs 17.25  7  0.0159     0.3374      0.1138
```

7. **The Digit Span Backward (DSB)** outcome include dance[82], CTE[8,64,66], AE[72,96,105], RE[76,84,88], and ME[44,48,52,106,107].

Local inconsistency

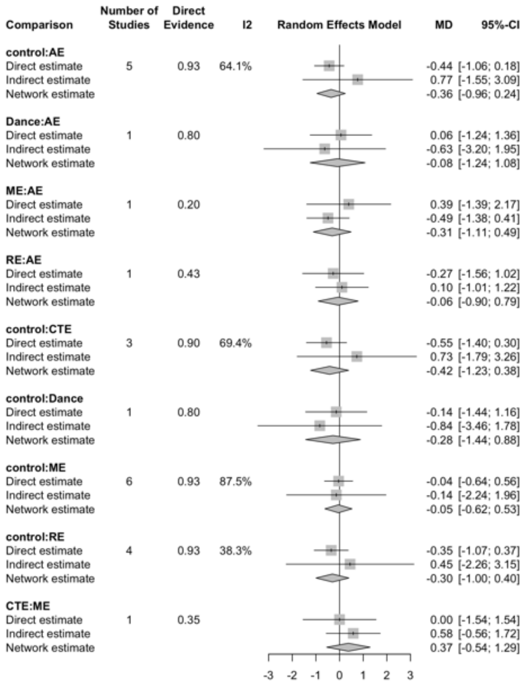

Global inconsistency

```
> print(global_inconsistency)
Q statistics to assess homogeneity / consistency

                    Q df  p-value
Total           59.65 14 < 0.0001
Within designs  46.41  7 < 0.0001
Between designs 13.24  7   0.0664

Design-specific decomposition of within-designs Q statistic

      Design     Q df  p-value
  control:ME 31.15  3 < 0.0001
  AE:control  6.08  1   0.0136
 control:CTE  5.00  1   0.0253
  control:RE  4.17  2   0.1241

Between-designs Q statistic after detaching of single designs
(influential designs have p-value markedly different from 0.0664)

 Detached design     Q df p-value
   AE:control:ME  6.64  5  0.2487
      control:ME  8.40  6  0.2104
   AE:control:RE  9.19  5  0.1017
      AE:control 12.40  6  0.0536
  control:CTE:ME 11.07  5  0.0500
      control:RE 12.92  6  0.0443
     control:CTE 13.11  6  0.0413

Q statistic to assess consistency under the assumption of
a full design-by-treatment interaction random effects model

                   Q df p-value tau.within tau2.within
Between designs 3.65  7  0.8193     0.8305      0.6897
```

8. **The Berg Balance Scale (BBS)** outcome includes Dance[1,2,57], Exergaming[5], Chinese Traditional Exercise (CTE)[8,12,95], Aerobic exercise (AE)[73,98,108], Resistance exercise (RE)[84], and Multicomponent exercise (ME)[41,50,81,89,109].

No inconsistency analysis was conducted, as the network lacked closed loops.

9. **The Geriatric Depression Scale (GDS)** outcome includes Dance[55,57], Chinese Traditional Exercise (CTE)[10,110], Aerobic exercise (AE)[17], Resistance exercise (RE)[75], and Multicomponent exercise (ME)[80,81,99,100].

No inconsistency analysis was conducted, as the network lacked closed loops.

10. **The Short Physical Performance Battery (SPPB)** outcome includes Dance[91], Exergaming[6,61,92], Chinese Traditional Exercise (CTE)[9], Resistance exercise (RE),[28,85] and Multicomponent exercise (ME)[35,44,99,103].

No inconsistency analysis was conducted, as the network lacked closed loops.

11. **The Quality of Life in Alzheimer's Disease (QoL-AD)** outcome includes Dance[91], Exergaming[5,7], Aerobic exercise (AE)[20,24,69], and Multicomponent exercise (ME)[46,80,111].

No inconsistency analysis was conducted, as the network lacked closed loops.

12. **The Gait Speed** outcome includes Dance[91], Exergaming[58,61], Aerobic exercise (AE)[70], and Multicomponent exercise (ME)[89,112].

No inconsistency analysis was conducted, as the network lacked closed loops.

References:

1. Bisbe M, Fuente-Vidal A, López E, et al. Comparative Cognitive Effects of Choreographed Exercise and Multimodal Physical Therapy in Older Adults with Amnestic Mild Cognitive Impairment: Randomized Clinical Trial. *Journal of Alzheimer's Disease*. 2020;73(2):769-783. doi:10.3233/JAD-190552
2. Qi M, Zhu Y, Zhang L, Wu T, Wang J. The effect of aerobic dance intervention on brain spontaneous activity in older adults with mild cognitive impairment: A resting-state functional MRI study. *Exp Ther Med*. Published online 2018:715-722. doi:10.3892/etm.2018.7006
3. Lazarou I, Parastatidis T, Tsolaki A, et al. International Ballroom Dancing Against Neurodegeneration: A Randomized Controlled Trial in Greek Community-Dwelling Elders With Mild Cognitive impairment. *Am J Alzheimers Dis Other Demen*. 2017;32(8):489-499. doi:10.1177/1533317517725813
4. Van de Winckel A, Feys H, De Weerdt W, Dom R. Cognitive and behavioural effects of music-based exercises in patients with dementia. *Clin Rehabil*. 2004;18(3):253-260. doi:10.1191/0269215504cr750oa
5. Padala KP, Padala PR, Malloy TR, et al. Wii-fit for improving gait and balance in an assisted living facility: A pilot study. *J Aging Res*. 2012;2012:6-11. doi:10.1155/2012/597573
6. van Santen J, Dröes RM, Twisk JWR, Blanson Henkemans OA, van Straten A, Meiland FJM. Effects of Exergaming on Cognitive and Social Functioning of People with Dementia: A Randomized Controlled Trial. *J Am Med Dir Assoc*. 2020;21(12):1958-1967.e5. doi:10.1016/j.jamda.2020.04.018
7. Zheng J, Yu P, Chen X. An Evaluation of the Effects of Active Game Play on Cognition, Quality of Life and Depression for Older People with Dementia. *Clin Gerontol*. 2022;45(4):1034-1043. doi:10.1080/07317115.2021.1980170
8. Lam LCW, Chau RCM, Wong BML, et al. A 1-Year Randomized Controlled Trial Comparing Mind Body Exercise (Tai Chi) With Stretching and Toning Exercise on Cognitive Function in Older Chinese Adults at Risk of Cognitive Decline. *J Am Med Dir Assoc*. 2012;13(6):568.e15-568.e20. doi:10.1016/j.jamda.2012.03.008
9. Jiayuan Z, Xiang-Zi J, Li-Na M, Jin-Wei Y, Xue Y. Effects of Mindfulness-Based Tai Chi Chuan on Physical Performance and Cognitive Function among Cognitive Frailty Older Adults: A Six-Month Follow-Up of a Randomized Controlled Trial. *Journal of Prevention of Alzheimer's Disease*. 2022;9(1):104-112. doi:10.14283/jpad.2021.40
10. Huang N, Li W, Rong X, et al. Effects of a Modified Tai Chi Program on Older People with Mild Dementia: A Randomized Controlled Trial. *Journal of Alzheimer's Disease*. 2019;72(3):947-956. doi:10.3233/JAD-190487
11. Chan AWK, Yu DSF, Choi KC, Lee DTF, Sit JWH, Chan HYL. Tai chi qigong as a means to improve night-time sleep quality among older adults with cognitive impairment: A pilot randomized controlled trial. *Clin Interv Aging*. 2016;11:1277-1286. doi:10.2147/CIA.S111927
12. Li K, Yu H, Kortas JA, Lin X, Lipowski M. The effect of 12 weeks of Baduanjin exercise on cognitive function, lower limb balance and quality of life of the elderly with mild cognitive impairment: a randomized controlled trial. *Gazzetta Medica Italiana Archivio per le Scienze Mediche*. 2022;181(11):811-823. doi:10.23736/S0393-3660.22.04802-1
13. Lam LCW, Chau RCM, Wong BML, et al. Interim follow-up of a randomized controlled trial comparing Chinese style mind body (Tai Chi) and stretching exercises on cognitive function in subjects at risk of progressive cognitive decline. *Int J Geriatr Psychiatry*. 2011;26(7):733-740. doi:10.1002/gps.2602

14. Tomoto T, Liu J, Tseng BY, et al. One-Year Aerobic Exercise Reduced Carotid Arterial Stiffness and Increased Cerebral Blood Flow in Amnestic Mild Cognitive Impairment. *Journal of Alzheimer's Disease*. 2021;80(2):841-853. doi:10.3233/JAD-201456
15. Tsai CL, Pai MC, Ukropec J, Ukropcová B. Distinctive Effects of Aerobic and Resistance Exercise Modes on Neurocognitive and Biochemical Changes in Individuals with Mild Cognitive Impairment. *Curr Alzheimer Res*. 2019;16(4):316-332. doi:10.2174/1567205016666190228125429
16. Wei X hong, Ji L li. Effect of handball training on cognitive ability in elderly with mild cognitive impairment. *Neurosci Lett*. 2014;566:98-101. doi:10.1016/j.neulet.2014.02.035
17. Nakatsuka M, Nakamura K, Hamanosono R, et al. A Cluster Randomized Controlled Trial of Nonpharmacological Interventions for Old-Old Subjects with a Clinical Dementia Rating of 0.5: The Kurihara Project. *Dement Geriatr Cogn Dis Extra*. 2015;5(2):221-232. doi:10.1159/000380816
18. Kohanpour MA, Peeri M, Azarbayjani MA. The effects of aerobic exercise with lavender essence use on cognitive state and serum brain-derived neurotrophic factor levels in elderly with mild cognitive impairment. *Journal of HerbMed Pharmacology*. 2017;6(2):80-84.
19. Liu IT, Lee WJ, Lin SY, Chang ST, Kao CL, Cheng YY. Therapeutic Effects of Exercise Training on Elderly Patients With Dementia: A Randomized Controlled Trial. *Arch Phys Med Rehabil*. 2020;101(5):762-769. doi:10.1016/j.apmr.2020.01.012
20. Yang SY, Shan CL, Qing H, et al. The Effects of Aerobic Exercise on Cognitive Function of Alzheimer's Disease Patients. *CNS Neurol Disord Drug Targets*. 2015;14(10):1292-1297. doi:10.2174/1871527315666151111123319
21. Khattak HG, Ahmad Z, Arshad H, Anwar K. Effect of aerobic exercise on cognition in elderly persons with mild cognitive impairment. *Rawal Medical Journal*. 2022;47(3):698-701. doi:10.5455/rmj.20210713072242
22. Varela S, Ayán C, Cancela JM, Martín V. Effects of two different intensities of aerobic exercise on elderly people with mild cognitive impairment: A randomized pilot study. *Clin Rehabil*. 2012;26(5):442-450. doi:10.1177/0269215511425835
23. Miu D, Edin F, Szeto S, Mak Y. A randomised controlled trial on the effect of exercise on physical, cognitive and affective function in dementia subjects. *Asian Journal of Gerontology & Geriatrics*. 2008;3(1):8-16.
24. Angiolillo A, Leccese D, Ciccotelli S, et al. Effects of Nordic walking in Alzheimer's disease: A single-blind randomized controlled clinical trial. *Heliyon*. 2023;9(5):e15865. doi:10.1016/j.heliyon.2023.e15865
25. Phoemsapthawee et al. The Benefit of Arm Swing Exercise on Cognitive Performance in Older Women with Mild Cognitive Impairment. *Journal of Exercise Physiology*. 2016;8(1):11-25.
26. Guzel I, Can F. The effects of different exercise types on cognitive and physical functions in dementia patients: A randomized comparative study. *Arch Gerontol Geriatr*. 2024;119(18):105321. doi:10.1016/j.archger.2023.105321
27. Venturelli M, Scarsini R, Schena F. Six-month walking program changes cognitive and ADL performance in patients with Alzheimer. *Am J Alzheimers Dis Other Demen*. 2011;26(5):381-388. doi:10.1177/1533317511418956
28. Yoon DH, Kang D, Kim HJ, Kim JS, Song HS, Song W. Effect of elastic band-based high-speed power training on cognitive function, physical performance and muscle strength in older women with mild cognitive impairment. *Geriatr Gerontol Int*. 2017;17(5):765-772. doi:10.1111/ggi.12784
29. Holthoff VA, Marschner K, Scharf M, et al. Effects of physical activity training in

patients with alzheimer's dementia: Results of a pilot RCT study. *PLoS One*. 2015;10(4):1-11. doi:10.1371/journal.pone.0121478

30. Baek JE, Hyeon SJ, Kim M, Cho HY, Hahm SC. Effects of dual-task resistance exercise on cognition, mood, depression, functional fitness, and activities of daily living in older adults with cognitive impairment: a single-blinded, randomized controlled trial. *BMC Geriatr*. 2024;24(1):1-12. doi:10.1186/s12877-024-04942-1
31. Venturelli M, Lanza M, Muti E, Schena F. Positive effects of physical training in activity of daily living-dependent older adults. *Exp Aging Res*. 2010;36(2):190-205. doi:10.1080/03610731003613771
32. Yang JG, Thapa N, Park HJ, et al. Virtual Reality and Exercise Training Enhance Brain, Cognitive, and Physical Health in Older Adults with Mild Cognitive Impairment. *Int J Environ Res Public Health*. 2022;19(20). doi:10.3390/ijerph192013300
33. Li L, Liu M, Zeng H, Pan L. Multi-component exercise training improves the physical and cognitive function of the elderly with mild cognitive impairment: A six-month randomized controlled trial. *Ann Palliat Med*. 2021;10(8):8919-8929. doi:10.21037/apm-21-1809
34. Avenali M, Picascia M, Tassorelli C, Sinforiani E, Bernini S. Evaluation of the efficacy of physical therapy on cognitive decline at 6-month follow-up in Parkinson disease patients with mild cognitive impairment: a randomized controlled trial. *Aging Clin Exp Res*. 2021;33(12):3275-3284. doi:10.1007/s40520-021-01865-4
35. Mak A, Delbaere K, Refshauge K, et al. Sunbeam Program Reduces Rate of Falls in Long-Term Care Residents With Mild to Moderate Cognitive Impairment or Dementia: Subgroup Analysis of a Cluster Randomized Controlled Trial. *J Am Med Dir Assoc*. 2022;23(5):743-749.e1. doi:10.1016/j.jamda.2022.01.064
36. Sobol NA, Hoffmann K, Frederiksen KS, et al. Effect of aerobic exercise on physical performance in patients with Alzheimer's disease. *Alzheimer's and Dementia*. 2016;12(12):1207-1215. doi:10.1016/j.jalz.2016.05.004
37. Hoffmann K, Sobol NA, Frederiksen KS, et al. Moderate-to-high intensity physical exercise in patients with Alzheimer's disease: A randomized controlled trial. *Journal of Alzheimer's Disease*. 2016;50(2):443-453. doi:10.3233/JAD-150817
38. Sobol NA, Dall CH, Høgh P, et al. Change in fitness and the relation to change in cognition and neuropsychiatric symptoms after aerobic exercise in patients with mild Alzheimer's disease. *Journal of Alzheimer's Disease*. 2018;65(1):137-145. doi:10.3233/JAD-180253
39. Lok N, Tosun AS, Lok S, Temel V, Aydın Z. Effect of physical activity program applied to patients with Alzheimer's disease on cognitive functions and depression level: a randomised controlled study. *Psychogeriatrics*. 2023;23(5):856-863. doi:10.1111/psyg.13010
40. De Sá CA, Saretto CB, Cardoso AM, Remor A, Breda CO, da Silva Corralo V. Effects of a physical exercise or motor activity protocol on cognitive function, lipid profile, and BDNF levels in older adults with mild cognitive impairment. *Mol Cell Biochem*. 2024;479(3):499-509. doi:10.1007/s11010-023-04733-z
41. Langoni CDS, Resende TDL, Barcellos AB, et al. Effect of Exercise on Cognition, Conditioning, Muscle Endurance, and Balance in Older Adults with Mild Cognitive Impairment: A Randomized Controlled Trial. *Journal of Geriatric Physical Therapy*. 2019;42(2):E15-E22. doi:10.1519/JPT.0000000000000191
42. Langoni C da S, Resende T de L, Barcellos AB, et al. The effect of group exercises on balance, mobility, and depressive symptoms in older adults with mild cognitive impairment: a randomized controlled trial. *Clin Rehabil*. 2019;33(3):439-449. doi:10.1177/0269215518815218

43. Zhang Q, Zhu M, Huang L, et al. A Study on the Effect of Traditional Chinese Exercise Combined With Rhythm Training on the Intervention of Older Adults With Mild Cognitive Impairment. *Am J Alzheimers Dis Other Demen*. 2023;38(48):1-12. doi:10.1177/15333175231190626
44. Sanders LMJ, Hortobágyi T, Karssemeijer EGA, Van Der Zee EA, Scherder EJA, Van Heuvelen MJG. Effects of low- And high-intensity physical exercise on physical and cognitive function in older persons with dementia: A randomized controlled trial. *Alzheimers Res Ther*. 2020;12(1):1-15. doi:10.1186/s13195-020-00597-3
45. Ghahfarrokhi MM, Shirvani H, Rahimi M, Bazgir B, Shamsadini A, Sobhani V. Feasibility and preliminary efficacy of different intensities of functional training in elderly type 2 diabetes patients with cognitive impairment: a pilot randomised controlled trial. *BMC Geriatr*. 2024;24(1):1-15. doi:10.1186/s12877-024-04698-8
46. Shaw I, Cronje M, Shaw BS. Group-based exercise as a therapeutic strategy for the improvement of mental outcomes in mild to moderate alzheimer's patients in low resource care facilities. *Asian J Sports Med*. 2021;12(1):1-6. doi:10.5812/asjsm.106593
47. Mollinedo Cardalda I, López A, Cancela Carral JM. The effects of different types of physical exercise on physical and cognitive function in frail institutionalized older adults with mild to moderate cognitive impairment. A randomized controlled trial. *Arch Gerontol Geriatr*. 2019;83(May):223-230. doi:10.1016/j.archger.2019.05.003
48. Bossers WJR, Van Der Woude LHV, Boersma F, Hortobágyi T, Scherder EJA, Van Heuvelen MJG. A 9-Week Aerobic and Strength Training Program Improves Cognitive and Motor Function in Patients with Dementia: A Randomized, Controlled Trial. *American Journal of Geriatric Psychiatry*. 2015;23(11):1106-1116. doi:10.1016/j.jagp.2014.12.191
49. Bossers WJR, van der Woude LHV, Boersma F, Hortobágyi T, Scherder EJA, van Heuvelen MJG. Comparison of Effect of Two Exercise Programs on Activities of Daily Living in Individuals with Dementia: A 9-Week Randomized, Controlled Trial. *J Am Geriatr Soc*. 2016;64(6):1258-1266. doi:10.1111/jgs.14160
50. Telenius EW, Engedal K, Bergland A. Long-term effects of a 12 weeks high-intensity functional exercise program on physical function and mental health in nursing home residents with dementia: A single blinded randomized controlled trial Physical functioning, physical health and activity. *BMC Geriatr*. 2015;15(1):1-11. doi:10.1186/s12877-015-0151-8
51. Telenius EW, Engedal K, Bergland A. Effect of a high-intensity exercise program on physical function and mental health in nursing home residents with dementia: An assessor blinded randomized controlled trial. *PLoS One*. 2015;10(5):1-18. doi:10.1371/journal.pone.0126102
52. Henskens M, Nauta IM, Van Eekeren MCA, Scherder EJA. Effects of Physical Activity in Nursing Home Residents with Dementia: A Randomized Controlled Trial. *Dement Geriatr Cogn Disord*. 2018;46(1-2):60-80. doi:10.1159/000491818
53. Esmail A, Vrinceanu T, Lussier M, et al. Effects of Dance/Movement Training vs. Aerobic Exercise Training on cognition, physical fitness and quality of life in older adults: A randomized controlled trial. *J Bodyw Mov Ther*. 2020;24(1):212-220. doi:10.1016/j.jbmt.2019.05.004
54. Franco MR, Sherrington C, Tiedemann A, et al. Effect of Senior Dance (DanSE) on Fall Risk Factors in Older Adults: A Randomized Controlled Trial. *Phys Ther*. 2020;100(4):600-608. doi:10.1093/ptj/pzz187
55. Zhu Y, Wu H, Qi M, et al. Effects of a specially designed aerobic dance routine on mild cognitive impairment. *Clin Interv Aging*. 2018;13:1691-1700. doi:10.2147/CIA.S163067

56. Song D, Yu D, Liu T, Wang J. Effect of an Aerobic Dancing Program on Sleep Quality for Older Adults With Mild Cognitive Impairment and Poor Sleep: A Randomized Controlled Trial. *J Am Med Dir Assoc*. 2024;25(3):494-499. doi:10.1016/j.jamda.2023.09.020
57. Chang J, Zhu W, Zhang J, et al. The Effect of Chinese Square Dance Exercise on Cognitive Function in Older Women With Mild Cognitive Impairment: The Mediating Effect of Mood Status and Quality of Life. *Front Psychiatry*. 2021;12(July). doi:10.3389/fpsyt.2021.711079
58. Sabbagh et al. Sensor-based balance training with motion feedback in people with mild cognitive impairment. 2016;53(6):945-958. doi:10.1682/JRRD.2015.05.0089.Sensor-based
59. Liu CL, Cheng FY, Wei MJ, Liao YY. Effects of Exergaming-Based Tai Chi on Cognitive Function and Dual-Task Gait Performance in Older Adults With Mild Cognitive Impairment: A Randomized Control Trial. *Front Aging Neurosci*. 2022;14(March). doi:10.3389/fnagi.2022.761053
60. Liao YY, Chen IH, Hsu WC, Tseng HY, Wang RY. Effect of exergaming versus combined exercise on cognitive function and brain activation in frail older adults: A randomised controlled trial. *Ann Phys Rehabil Med*. 2021;64(5):101492. doi:10.1016/j.rehab.2021.101492
61. Swinnen N, Vandenbulcke M, de Bruin ED, et al. The efficacy of exergaming in people with major neurocognitive disorder residing in long-term care facilities: a pilot randomized controlled trial. *Alzheimers Res Ther*. 2021;13(1):1-13. doi:10.1186/s13195-021-00806-7
62. Khanthong P, Sriyakul K, Dechakhamphu A, Krajarng A, Kamalashiran C, Tungsukruthai P. Traditional Thai exercise (Ruesi Dadton) for improving motor and cognitive functions in mild cognitive impairment: a randomized controlled trial. *J Exerc Rehabil*. 2021;17(5):331-338. doi:10.12965/JER.2142542.271
63. Kashyap M, Rai NK, Singh R, et al. Effect of Early Yoga Practice on Post Stroke Cognitive Impairment. 2022;22(4):2019. doi:10.4103/aian.AIAN
64. Li F, Harmer P, Fitzgerald K, Winters-Stone K. A cognitively enhanced online Tai Ji Quan training intervention for community-dwelling older adults with mild cognitive impairment: A feasibility trial. *BMC Geriatr*. 2022;22(1):1-13. doi:10.1186/s12877-021-02747-0
65. Chen Y, Qin J, Tao L, et al. Effects of Tai Chi Chuan on Cognitive Function in Adults 60 Years or Older With Type 2 Diabetes and Mild Cognitive Impairment in China: A Randomized Clinical Trial. *JAMA Netw Open*. 2023;6(4):E237004. doi:10.1001/jamanetworkopen.2023.7004
66. Yu AP, Chin EC, Yu DJ, et al. Tai Chi versus conventional exercise for improving cognitive function in older adults: a pilot randomized controlled trial. *Sci Rep*. 2022;12(1):1-15. doi:10.1038/s41598-022-12526-5
67. Zheng G, Zheng Y, Xiong Z, Ye B. Effect of Baduanjin exercise on cognitive function in patients with post-stroke cognitive impairment: a randomized controlled trial. *Clin Rehabil*. 2020;34(8):1028-1039. doi:10.1177/0269215520930256
68. Zheng G, Ye B, Xia R, et al. Traditional Chinese Mind-Body Exercise Baduanjin Modulate Gray Matter and Cognitive Function in Older Adults with Mild Cognitive Impairment: A Brain Imaging Study. *Brain Plasticity*. 2021;7(2):131-142. doi:10.3233/bpl-210121
69. Song D, Yu DSF. Effects of a moderate-intensity aerobic exercise programme on the cognitive function and quality of life of community-dwelling elderly people with mild cognitive impairment: A randomised controlled trial. *Int J Nurs Stud*. 2019;93:97-105.

doi:10.1016/j.ijnurstu.2019.02.019
70. Rojasavastera R, Bovonsunthonchai S, Hiengkaew V, Senanarong V. Action observation combined with gait training to improve gait and cognition in elderly with mild cognitive impairment a randomized controlled trial. *Dementia e Neuropsychologia*. 2020;14(2):118-127. doi:10.1590/1980-57642020dn14-020004
71. Karthikeyan T. Therapeutic effects of home-based exercise of geriatrics for the management of cognitive impairment. *ES J Public Health*. 2020;1(1):1003. www.escientificlibrary.com
72. Krootnark K, Chaikeeree N, Saengsirisuwan V, Boonsinsukh R. Effects of low-intensity home-based exercise on cognition in older persons with mild cognitive impairment: a direct comparison of aerobic versus resistance exercises using a randomized controlled trial design. *Front Med (Lausanne)*. 2024;11(June):1-11. doi:10.3389/fmed.2024.1392429
73. Choi W, Lee S. Ground kayak paddling exercise improves postural balance, muscle performance, and cognitive function in older adults with mild cognitive impairment: A randomized controlled trial. *Medical Science Monitor*. 2018;24:3909-3915. doi:10.12659/MSM.908248
74. Amjad I, Toor H, Niazi IK, et al. Therapeutic effects of aerobic exercise on EEG parameters and higher cognitive functions in mild cognitive impairment patients. *International Journal of Neuroscience*. 2019;129(6):551-562. doi:10.1080/00207454.2018.1551894
75. Wang L, Wu B, Tao H, et al. Effects and mediating mechanisms of a structured limbs-exercise program on general cognitive function in older adults with mild cognitive impairment: A randomized controlled trial. *Int J Nurs Stud*. 2020;110:103706. doi:10.1016/j.ijnurstu.2020.103706
76. Hong SG, Kim JH, Jun TW. Effects of 12-week resistance exercise on electroencephalogram patterns and cognitive function in the elderly with mild cognitive impairment: A randomized controlled trial. *Clinical Journal of Sport Medicine*. 2018;28(6):500-508. doi:10.1097/JSM.0000000000000476
77. Greblo Jurakic Z, Krizanic V, Sarabon N, Markovic G. Effects of feedback-based balance and core resistance training vs. Pilates training on cognitive functions in older women with mild cognitive impairment: a pilot randomized controlled trial. *Aging Clin Exp Res*. 2017;29(6):1295-1298. doi:10.1007/s40520-017-0740-9
78. Kim J, Yim J. Effects of an exercise protocol for improving handgrip strength and walking speed on cognitive function in patients with chronic stroke. *Medical Science Monitor*. 2017;23:5402-5409. doi:10.12659/MSM.904723
79. Rivas-Campo Y, Aibar-Almazán A, Afanador-Restrepo DF, et al. Effects of High-Intensity Functional Training (HIFT) on the Functional Capacity, Frailty, and Physical Condition of Older Adults with Mild Cognitive Impairment: A Blind Randomized Controlled Clinical Trial. *Life*. 2023;13(5):1-16. doi:10.3390/life13051224
80. Levinger P, Goh AMY, Dunn J, et al. Exercise interveNtion outdoor proJect in the cOmmunitY – results from the ENJOY program for independence in dementia: a feasibility pilot randomised controlled trial. *BMC Geriatr*. 2023;23(1):1-16. doi:10.1186/s12877-023-04132-5
81. Akbuga Koc E, Yazici-Mutlu Ç, Cinar N, Sahiner T. Comparison of the effect of online physical exercise and computerized cognitive stimulation in patients with Alzheimer's disease during the Covid-19 pandemic. *Complement Ther Clin Pract*. 2024;57(May):10-20. doi:10.1016/j.ctcp.2024.101881
82. Ho RTH, Fong TCT, Chan WC, et al. Psychophysiological Effects of Dance Movement Therapy and Physical Exercise on Older Adults with Mild Dementia: A Randomized

Controlled Trial. *Journals of Gerontology - Series B Psychological Sciences and Social Sciences*. 2018;75(3):560-570. doi:10.1093/geronb/gby145
83. Su H, Wang H, Meng L, Bush E. The effects of Baduanjin exercise on the subjective memory complaint of older adults: A randomized controlled trial. *Medicine (United States)*. 2021;100(30):E25442. doi:10.1097/MD.0000000000025442
84. Fernandez-Gonzalo R, Fernandez-Gonzalo S, Turon M, Prieto C, Tesch PA, García-Carreira MDC. Muscle, functional and cognitive adaptations after flywheel resistance training in stroke patients: A pilot randomized controlled trial. *J Neuroeng Rehabil*. 2016;13(1):1-11. doi:10.1186/s12984-016-0144-7
85. Yoon DH, Lee JY, Song W. Effects of Resistance Exercise Training on Cognitive Function and Physical Performance in Cognitive Frailty: A Randomized Controlled Trial. *Journal of Nutrition, Health and Aging*. 2018;22(8):944-951. doi:10.1007/s12603-018-1090-9
86. Fonte C, Smania N, Pedrinolla A, et al. Comparison between physical and cognitive treatment in patients with MCI and Alzheimer's disease. *Aging*. 2019;11(10):3138-3155. doi:10.18632/aging.101970
87. Eyre HA, Siddarth P, Acevedo B, et al. A randomized controlled trial of Kundalini yoga in mild cognitive impairment. *Int Psychogeriatr*. 2017;29(4):557-567. doi:10.1017/S1041610216002155
88. Lv J, Liu Y. Effects of momentum-based dumbbell training on motor control in older adults with mild cognitive impairment. *Chinese Journal of Rehabilitation Medicine*. 2019;34(5):544-550. doi:10.3969/j.issn.1001-1242.2019.05.009
89. Dawson N, Judge KS, Gerhart H. Improved Functional Performance in Individuals with Dementia after a Moderate-Intensity Home-Based Exercise Program: A Randomized Controlled Trial. *Journal of Geriatric Physical Therapy*. 2019;42(1):18-27. doi:10.1519/JPT.0000000000000128
90. Bo W, Lei M, Tao S, et al. Effects of combined intervention of physical exercise and cognitive training on cognitive function in stroke survivors with vascular cognitive impairment: a randomized controlled trial. *Clin Rehabil*. 2019;33(1):54-63. doi:10.1177/0269215518791007
91. Bracco L, Pinto-Carral A, Hillaert L, Mourey F. Tango-therapy vs physical exercise in older people with dementia; a randomized controlled trial. *BMC Geriatr*. 2023;23(1):1-13. doi:10.1186/s12877-023-04342-x
92. Karssemeijer EGA, Bossers WJR, Aaronson JA, Sanders LMJ, Kessels RPC, Olde Rikkert MGM. Exergaming as a Physical Exercise Strategy Reduces Frailty in People With Dementia: A Randomized Controlled Trial. *J Am Med Dir Assoc*. 2019;20(12):1502-1508.e1. doi:10.1016/j.jamda.2019.06.026
93. Li F, Harmer P, Voit J, Chou LS. Implementing an online virtual falls prevention intervention during a public health pandemic for older adults with mild cognitive impairment: A feasibility trial. *Clin Interv Aging*. 2021;16:973-983. doi:10.2147/CIA.S306431
94. Liu JYW, Kwan RYC, Lai CKY, Hill KD. A simplified 10-step Tai-chi programme to enable people with dementia to improve their motor performance: a feasibility study. *Clin Rehabil*. 2018;32(12):1609-1623. doi:10.1177/0269215518786530
95. Nyman SR, Ingram W, Sanders J, et al. Randomised controlled trial of the effect of tai chi on postural balance of people with dementia. *Clin Interv Aging*. 2019;14:2017-2029. doi:10.2147/CIA.S228931
96. Donnezan et al. Effects of simultaneous aerobic and cognitive training on executive functions, cardiovascular fitness and functional abilities in older adults with mild cognitive impairment. *Ment Health Phys Act*. 2018;15(April):78-87.

doi:10.1016/j.mhpa.2018.06.001
97. Dillon K, Prapavessis H. REducing SEDENTary behavior among mild to moderate cognitively impaired assisted living residents: A pilot randomized controlled trial (RESEDENT study). *J Aging Phys Act*. 2021;29(1):27-35. doi:10.1123/JAPA.2019-0440
98. Abbas RL, Saab IM, Al-Sharif HK, Naja N, El-Khatib A. Effect of Adding Motorized Cycle Ergometer Over Exercise Training on Balance in Older Adults with Dementia: A Randomized Controlled Trial. *Exp Aging Res*. 2023;49(2):100-111. doi:10.1080/0361073X.2022.2046947
99. Ullrich P, Werner C, Schönstein A, et al. Effects of a Home-Based Physical Training and Activity Promotion Program in Community-Dwelling Older Persons with Cognitive Impairment after Discharge from Rehabilitation: A Randomized Controlled Trial. *Journals of Gerontology - Series A Biological Sciences and Medical Sciences*. 2022;77(12):2435-2444. doi:10.1093/gerona/glac005
100. Vreugdenhil A, Cannell J, Davies A, Razay G. A community-based exercise programme to improve functional ability in people with Alzheimer's disease: A randomized controlled trial. *Scand J Caring Sci*. 2012;26(1):12-19. doi:10.1111/j.1471-6712.2011.00895.x
101. Hauer K, Schwenk M, Zieschang T, Essig M, Becker C, Oster P. Physical training improves motor performance in people with dementia: A randomized controlled trial. *J Am Geriatr Soc*. 2012;60(1):8-15. doi:10.1111/j.1532-5415.2011.03778.x
102. Suttanon P, Hill KD, Said CM, et al. Feasibility, safety and preliminary evidence of the effectiveness of a home-based exercise programme for older people with Alzheimer's disease: A pilot randomized controlled trial. *Clin Rehabil*. 2013;27(5):427-438. doi:10.1177/0269215512460877
103. Gebhard D, Mess F. Feasibility and Effectiveness of a Biography-Based Physical Activity Intervention in Institutionalized People With Dementia: Quantitative and Qualitative Results From a Randomized Controlled Trial. *J Aging Phys Act*. 2022;30(2):237-251. doi:10.1123/japa.2020-0343
104. Cezar NO de C, Ansai JH, Oliveira MPB de, et al. Feasibility of improving strength and functioning and decreasing the risk of falls in older adults with Alzheimer's dementia: a randomized controlled home-based exercise trial. *Arch Gerontol Geriatr*. 2021;96(March). doi:10.1016/j.archger.2021.104476
105. Eggermont LHP, Swaab DF, Hol EM, Scherder EJA. Walking the line: A randomised trial on the effects of a short term walking programme on cognition in dementia. *J Neurol Neurosurg Psychiatry*. 2009;80(7):802-804. doi:10.1136/jnnp.2008.158444
106. Li PWC, Yu DSF, Siu PM, Wong SCK, Chan BS. Peer-supported exercise intervention for persons with mild cognitive impairment: A waitlist randomised controlled trial (the BRAin Vitality Enhancement trial). *Age Ageing*. 2022;51(10):1-10. doi:10.1093/ageing/afac213
107. Prick AE, De Lange J, Scherder E, Twisk J, Pot AM. The effects of a multicomponent dyadic intervention with physical exercise on the cognitive functioning of people with dementia: A randomized controlled trial. *J Aging Phys Act*. 2017;25(4):539-552. doi:10.1123/japa.2016-0038
108. L.F. Law et al. Effects of functional task exercise on everyday problem-solving ability and functional status in older adults with mild cognitive impairment—a randomised controlled trial. *Age Ageing*. 2021;51(7):1-11. doi:10.1093/ageing/afac144
109. Toots A, Littbrand H, Boström G, et al. Effects of exercise on cognitive function in older people with dementia: A randomized controlled trial. *Journal of Alzheimer's Disease*. 2017;60(1):323-332. doi:10.3233/JAD-170014

110. Cheng ST, Chow PK, Song YQ, et al. Mental and physical activities delay cognitive decline in older persons with dementia. *American Journal of Geriatric Psychiatry*. 2014;22(1):63-74. doi:10.1016/j.jagp.2013.01.060
111. Lamb SE, Sheehan B, Atherton N, et al. Dementia And Physical Activity (DAPA) trial of moderate to high intensity exercise training for people with dementia: Randomised controlled trial. *BMJ (Online)*. 2018;361. doi:10.1136/bmj.k1675
112. Doi T, Makizako H, Shimada H, et al. Effects of multicomponent exercise on spatial-temporal gait parameters among the elderly with amnestic mild cognitive impairment (aMCI): Preliminary results from a randomized controlled trial (RCT). *Arch Gerontol Geriatr*. 2013;56(1):104-108. doi:10.1016/j.archger.2012.09.003
