# Supplementary material for: Dance versus other exercise modalities in mild cognitive impairment and dementia: comparative efficacy from a systematic review and bayesian network meta-analysis
Source: Front Physiol. 2026 Mar 25;17:1782774. doi: 10.3389/fphys.2026.1782774 (PMC13056856; doi:10.3389/fphys.2026.1782774)
Supplement: Supplementary file 6 [file Table6.pdf]

Supplementary Table 6. Summary of Research Limitations and Future Research Directions

Intervention Categories	Existing Limitations	Future Directions
**Dance**		
	1. Small sample sizes (fewer than 100 participants)[1–11,12,13]<br>2. Uneven gender distribution across groups[1,3–8,10,11,14–16,12,13]<br>3. Absence of a non-interventional control group[2,6,10]<br>4. High attrition rates (greater than 20% in either intervention or control groups)[1,2,6,8,14,15]<br>5. Lake of follow-up assessments[1–6,8–11,14,15,17,13,18,12,13]<br>6. Limited generalizability due to specific population characteristics[2,5,7,15,16]<br>7. Risk of cross-contamination between groups[1]<br>8. No rigorous monitoring of participants' lifestyle behaviours[1]<br>9. Inability to assess the impact of adherence on intervention effect sizes[5]<br>10. Use of subjective measures as primary outcomes[2]<br>11. Sampling bias (e.g., high educated individuals, physical active participants)[11,16]<br>12. Manual calculation of outcome measures[11]<br>13. Lack of detailed etiological classification (e.g., dementia subtype not identified)[16]<br>14. Data collection limitations or biases (e.g., reliance on self-reported timing, biomarker samples collected on a single day)[16]<br>15. During the cardiopulmonary exercise test, psychological factors and varying levels of motivation among participates may have impacted their performance during these tests[12]<br>16. Did not control external exercise load[12]<br>17. COVID-19 impacts[12]	1. Conduct larger-sample, multicenter studies to enhance statistical power and generalizability.<br>2. Ensure sex-balanced study designs to explore potential gender-specific effects of interventions.<br>3. Design randomized controlled trials that include non-intervention control groups to isolate treatment effects.<br>4. Develop retention strategies (e.g., flexible scheduling, regular follow-ups) to minimize dropouts and maintain sample integrity.<br>5. Incorporate longitudinal designs with extended follow-up periods to assess the long-term sustainability of intervention effects.<br>6. Recruit demographically diverse participants to improve external validity and applicability to broader populations.<br>7. Employ study designs that prevent cross-group contamination (e.g., separate facilities or time slots).<br>8. Implement standardized monitoring or control for confounding lifestyle factors such as diet, sleep, and physical activity.<br>9. Track and analyze adherence levels to better understand dose-response relationships and their effect on outcomes.<br>10. Utilize objective, validated assessment tools to improve the reliability and reproducibility of findings.<br>11. Target underrepresented groups and varied socioeconomic backgrounds to reduce sampling bias.<br>12. Adopt automated or standardized scoring systems to reduce human error and increase accuracy.<br>13. Classify participants by dementia subtypes to enable subgroup analyses and condition-specific recommendations.<br>14. Apply rigorous data collection protocols (e.g., multiple data points, objective biomarkers) to minimize bias.<br>15. Investigate the effects of different intervention types and intensities (e.g., dance, aerobic, resistance) across various cognitive and psychosocial domains. Explore dose-response relationships to determine optimal exercise intensity and frequency. Conduct studies

		examining the specific effects of dance interventions on different types of cognitive impairment.
**Exergaming**		
	1. Small sample sizes (fewer than 100 participants)[19–26,27]<br>2. Uneven gender distribution across groups[19–26,28–30,27]<br>3. Absence of a non-interventional control group[23–25,29]<br>4. High attrition rates (greater than 20% in either intervention or control groups)[21,24,25,29,30]<br>5. Lack of follow-up assessments[20,22–26,29,30.27]<br>6. Short intervention duration (less than 12 weeks)[20,23,26,30,27]<br>7. Combined with multiple components, making it difficult to isolate training effects[20,21]<br>8. Limited generalizability due to specific population characteristics [26,28–30]<br>9. Use of Boger Balance Score as the primary outcome, prone to ceiling effect in high-functioning participants[23]<br>10. Non-blinded assessors[23]<br>11. Complex and burdensome measurement protocols[29]<br>12. Concerns about reliability of certain measures[29]<br>13. Lack of control for concurrent medication use[26,30]<br>14. Convenience sampling[30]<br>15. Potential researcher effects influencing interpretation[30]<br>16. Potential influence of social interaction rather than the intervention itself[30]	1. Conduct larger sample size studies to improve statistical power and generalizability. Multicenter trials may enhance population diversity.<br>2. Ensure balanced gender representation to explore sex-specific responses to exergaming interventions.<br>3. Design studies with non-intervention control groups to isolate intervention-specific effects.<br>4. Identify barriers to adherence and investigate how positive perceptions of the training system influence long-term compliance.<br>5. Conduct longitudinal studies to assess the sustainability of cognitive and physical benefits over time.<br>6. Implement longer-term interventions to evaluate dose-response relationships and determine optimal duration.<br>7. Examine the unique and combined influences of cognitive, social, and physical components of exergaming using factorial or dismantling study designs.<br>8. Expand recruitment to include more diverse populations to improve external validity.<br>9. Use biomechanical assessments or alternative balance measures with greater sensitivity to avoid ceiling effects in high-functioning individuals.<br>10. Apply blinded outcome assessments to minimize evaluator bias.<br>11. Simplify assessment protocols using user-friendly technology and automation (e.g., Bluetooth-enabled sensors for in-home training).<br>12. Validate tools in target populations and consider incorporating physiological and neurobiological markers (e.g., BDNF levels, brain imaging).<br>13. Control for medication use in study design and analysis to reduce potential confounding.<br>14. Use probabilistic or stratified sampling to ensure representative samples.<br>15. Minimize researcher bias through protocol standardization, blinding, and independent data analysis.<br>16. Include comparison groups with similar social interaction to distinguish cognitive improvements due to the intervention versus social

		engagement.<br>17. Future studies should explore the effectiveness of different games and training platforms, measure exercise intensity and engagement (e.g., perceived exertion, distance traveled) and integrate neuroimaging and physiological biomarkers to evaluate underlying mechanisms.
**Yoga**		
	1. Small sample sizes (fewer than 100 participants)[31–33]<br>2. Uneven gender distribution across groups[31–35]<br>3. Absence of a non-interventional control group[31,33–35]<br>4. High attrition rates (greater than 20% in either intervention or control groups)[33–35]<br>5. Lack of follow-up assessments[32–34]<br>6. Limited generalizability due to specific population characteristics[31]<br>7. Overrepresentation of white, college-educated participants[33]<br>8. Lack of home practice requirements[33,35]<br>9. Combined with multiple components, making it difficult to isolate training effects[35]	1. Conduct larger-scale studies to improve statistical power, reduce sampling error, and enhance generalizability.<br>2. Design sex-balanced studies to assess potential gender-specific responses to yoga interventions.<br>3. Include non-intervention control groups in randomized designs to isolate the specific effects of the yoga program.<br>4. Improve engagement and monitor adherence through personalized support, flexible scheduling, and ongoing feedback. Investigate how engagement and perceptions of yoga influence retention.<br>5. Conduct long-term follow-up assessments (e.g., beyond 6 months) to examine maintenance effects and sustained benefits.<br>6. Expand recruitment strategies to include more diverse racial, educational, and socioeconomic groups.<br>7. Prioritize inclusive sampling methods to reach underrepresented populations and improve external validity.<br>8. Incorporate structured home-based components (e.g., meditation, breathing) and assess their independent and combined contributions to outcomes.<br>9. Use dismantling or factorial designs to separate and examine the unique effects of class-based yoga, home meditation, and social interaction.<br>10. Future studies should investigate the roles of social interaction and group engagement as active components of yoga-based interventions. Researchers should also evaluate program adherence metrics and their relationship to long-term outcomes.
**Chinese Traditional Exercise (CTE)**		
	1. Small sample sizes (fewer than 100 participants)[36–51,52,53,54]<br>2. Uneven gender distribution across groups[36–51,55–57,52,53,54]<br>3. Absence of a non-interventional control group[36,38,39,47,55]<br>4. High attrition rate (greater than 20% in either intervention or	1. Conduct larger-sample, adequately powered trials to improve statistical validity and generalizability.<br>2. Design gender-balanced studies to identify sex-specific intervention effects and ensure equitable representation.

control groups)[44,48,55,52]	3. Include passive, non-exercise control groups to isolate true intervention effects.
5. Lack of follow-up assessments[36–43,45–47,49–51,55,54,53,52]	4. Develop strategies to improve retention, such as enhanced participant engagement and support; assess adherence and its relationship to outcomes.
6. Short intervention duration (less than 12 weeks)[44]	5. Conduct long-term follow-up assessments (e.g., beyond 6 months) to evaluate maintenance of benefits over time.
7. Limited generalizability due to specific population characteristics[37,38,43,48,57,53,54]	6. Extend intervention periods to explore dose-response effects and maximize potential outcomes.
8. No a priori sample size calculation[36]	7. Recruit more diverse populations, including those from varied ethnic, socioeconomic, and educational backgrounds.
9. Self-report bias[38]	8. Conduct formal sample size estimations during study design to ensure sufficient power for detecting meaningful effects.
10. Recruitment bias (e.g., exclusion of high fall-risk individuals)[38]	9. Use validated, objective outcome measures and triangulate with self-reported data to reduce bias.
11. COVID-19-related limitations on outdoor activities[38]	10. Expand inclusion criteria to enroll participants at increased risk (e.g., older adults with fall risk) for greater relevance.
12. Overrepresenting of highly education participants[55]	11. Replicate studies under normal conditions to control for pandemic-related confounds and better reflect real-world functioning.
13. High resource demands for study administration[56]	12. Ensure recruitment across education levels to enhance representativeness.
14. Weakness in diagnostic criteria application (e.g., delayed recall in MoCA)[40]	13. Explore digital or hybrid delivery models to reduce human resource demands and improve scalability.
15. Loss of social interaction due to COVID-19 precautions[41,53]	14. Use more rigorous and standardized cognitive assessments to confirm diagnostic classifications (e.g., aMCI).
16. Control group social interaction level not assessed[41]	15. Include socially engaging components in future studies and assess their contributions to cognitive and emotional outcomes.
17. Environmental stimulation from traveling to intervention venues[41]	16. Quantify and control for social interaction across all study arms to clarify its role in observed improvements.
18. Selection bias (e.g., recruitment after randomization)[43,57]	17. Account for incidental cognitive stimulation (e.g., travel exposure) in study design and interpretation.
19. Lack of confirmation the home practice adherence[45]	18. Implement rigorous randomization procedures prior to recruitment to reduce bias and improve internal validity.
20. No control for concurrent medications or rehabilitation[48]	19. Develop systems to monitor and confirm home practice (e.g., digital tracking, homework logs).
21. Absence of biological or neurophysiological measures[50]	20. Screen for and statistically adjust for external medical or rehabilitation interventions to avoid confounding.
22. Single-task tests may not fully capture functional performance[50]	
23. Medication effects[52]	
24. Detection bias[54]	

		21. Incorporate physiological and neurobiological markers (e.g., BDNF, brain imaging) to explore mechanisms of action.<br>22. Use multi-task or dual-task paradigms to better assess real-life functional performance and mobility.<br>23. Cluster or multi-center study could provide more representative results.
**Aerobic Exercise (AE)**		
	1. Small sample sizes (fewer than 100 participants)[58–92]<br>2. Uneven gender distribution across groups[58,61–71,73–76,79–86,88,91–100,101,102]<br>3. Absence of a non-interventional control group[58,60,61,64,65,69,71,74,75,78,86,87,89,90,92,98,103,101]<br>4. High attrition rate (greater than 20% in either intervention or control groups)[58,62,67,72,75,78,81,86,95,98,101,96]<br>5. Short intervention duration (under 12 weeks)[63,67,69,71,72,74,77,82,84,86,88,89,91,99]<br>6. Lack of follow-up assessments[58–66,68,69,71–78,80–96,98,100,103,101,102]<br>7. Limited generalizability due to specific population characteristics [60,63,66,70,71,74,77,86,91,93–95,99,102]<br>8. Unknown group differences in risk factors (e.g., APOE-ε4, baseline physical activity)[93]<br>9. Influence of social components (group interaction)[59,62,73,80,82,97,101]<br>10. Measurement tools may lack sensitivity (e.g., post-stroke outcomes)[94]<br>11. Heterogeneous samples with high mean age and comorbidities[94]<br>12. Lack of supervised training[94]<br>13. Self-reported training details (e.g., length, intensity)[94]<br>14. Reliance on subjective self-report measures[85,95]<br>15. No mid-point or post-intervention assessments during the follow-up[97]<br>16. Potential confounding from transportation-related environmental exposure[97]<br>17. Baseline group differences[98]<br>18. Daily physical activity not monitored[71]<br>19. Simplified depression questionnaire[71]<br>20. No objective intensity monitors (e.g., heart rate)[71]	1. Conduct larger, adequately powered trials to improve statistical robustness, enable subgroup analyses, and enhance generalizability across populations.<br>2. Recruit gender-balanced samples and examine sex-specific responses to aerobic exercise interventions.<br>3. Include passive and active control groups to isolate specific contributions of aerobic training from social or placebo effects.<br>4. Implement strategies to improve adherence (e.g., motivational support, progress feedback) and explore reasons for dropout.<br>5. Conduct long-term interventions and explore optimal dose-response relationships, including frequency, intensity, and duration.<br>6. Incorporate long-term follow-up (6–12 months or more) to evaluate sustainability and delayed effects of intervention.<br>7. Conduct studies in racially, ethnically, and socioeconomically diverse populations to confirm findings across groups. Examine whether exercise responses vary by race, gender, or education.<br>8. Collect genetic and lifestyle data to examine risk-related moderators (e.g., APOE genotype) and their impact on exercise efficacy.<br>9. Compare individual versus group training formats to evaluate the role of social interaction and investigate its independent contribution to cognitive outcomes.<br>10. Utilize a broader range of cognitive and functional assessments, including more sensitive and condition-specific tools.<br>11. Stratify analyses or conduct subgroup comparisons to account for clinical and demographic variability.<br>12. Include supervised or partially supervised sessions to improve adherence, ensure safety, and verify intensity levels.<br>13. Utilize wearable devices (e.g., HR monitors, accelerometers) to objectively track exercise adherence and intensity.<br>14. Combine subjective data with objective performance- or biomarker-

21. Medication history not recorded[72]<br>22. Heterogeneity in participant profiles[72,78]<br>23. Laboratory-based intervention may limit ecological validity[75]<br>24. Inaccurate control of training intensity (e.g., for BDNF responses)[82]<br>25. Genetic factors not accounted[82]<br>26. Dementia subtype not specified[84]<br>27. Influence of medication and comorbidities not controlled[86]<br>28. The existing medication confounding effects[89]<br>29. Dementia diagnosis based on MMSE only[90]<br>30. Socioeconomic confounders not controlled[91,92]<br>31. Performance bias due to outcome test similarity to training task[99]<br>32. COVID-19 impacts[101,102]<br>33. No blind data collectors[102]	based assessments.<br>15. Investigate adherence barriers and facilitators and design supportive measures to sustain participation in long-term programs.<br>16. Include interim assessments to monitor progress and identify when benefits plateau or decline.<br>17. Standardize or control for exposure to environmental stimuli (e.g., sensory enrichment from travel).<br>18. Use proper randomization and statistical adjustment to account for baseline imbalances.<br>19. Monitor background activity using wearable technologies to isolate effects of the intervention.<br>20. Use validated, comprehensive tools (e.g., PHQ-9, GDS) to capture mood changes over time<br>21. Use HR monitors, VO_2 testing, or other physiological markers to confirm training intensity and standardize protocols.<br>22. Collect and control for medication use to reduce confounding and clarify effects of exercise alone.<br>23. Apply inclusion criteria or stratified analyses to improve sample consistency or interpret subgroup effects.<br>24. Transition to home- or community-based training to improve ecological validity and scalability.<br>25. Standardize exercise intensity and investigate neurobiological mechanisms through biomarkers (e.g., BDNF, cortisol).<br>26. Explore gene–exercise interactions by collecting APOE and other relevant genetic data.<br>27. Use standardized diagnostic criteria to differentiate dementia subtypes and tailor interventions accordingly.<br>28. Adjust for or restrict inclusion based on medical history to minimize confounding.<br>29. Use comprehensive diagnostic tools and multiple cognitive domains for accurate classification.<br>30. Collect and adjust for SES variables (e.g., education, income, occupation, race) in analyses.<br>31. Use outcome measures that are not directly practiced during training to reduce task-specific learning effects.<br>32. Use neuroimaging (MRI, EEG) and biomarkers to explore structural and functional brain changes induced by aerobic training.

		33. Investigate whether aerobic fitness drives cognitive improvement or is influenced by cognitive decline.<br>34. Compare different exercise types (e.g., aerobic vs. resistance) to explore their distinct and combined benefits.<br>35. Employ sophisticated EEG and neuroimaging techniques to examine inter-segmental brain pathway changes.
**Resistance Exercise (RE)**		
	1. Small sample sizes (fewer than 100 participants)[104–113,114]<br>2. Uneven gender distribution across groups[104,106–110,113,115,116,114]<br>3. Absence of a non-interventional control group[107,108,113]<br>4. High attrition rates (greater than 20% in either intervention or control groups)[106–108,111,115,114]<br>5. Short intervention duration (less than 12 weeks)[109,113]<br>6. Lack of follow-up assessments[104–110,113,116,114]<br>7. Limited generalizability due to specific population characteristics[116]<br>8. Hemispheric lateralization entered as covariate may not fully resolve design bias[104]<br>9. Influence of social attention from staff[105]<br>10. Reliance on self-reported data[116]<br>11. Mediating variables (e.g., fatigue, stress, self-efficacy) not accounted for[116]<br>12. Inclusion of participants with prior COVID-19 history[106]<br>13. Potentially uncontrolled parameters: sleep, diet, physical activity, education, social background, and gender equity[109,110,113]<br>14. Potential measurement error in EEG electrode placement[109]	1. Conduct larger, adequately powered trials to enhance statistical validity, enable subgroup analyses, and increase generalizability.<br>2. Ensure balanced gender representation or stratify analyses to explore sex-specific effects of resistance training.<br>3. Develop better-controlled study designs using passive or attention-matched control groups to isolate resistance training effects.<br>4. Implement adherence strategies (e.g., participant engagement programs, flexible scheduling) and monitor reasons for dropout.<br>5. Conduct long-term interventions (≥ 12 weeks) to explore sustained benefits and optimal training parameters.<br>6. Include long-term follow-up (6–12 months or more) to evaluate maintenance of cognitive and physical improvements.<br>7. Expand recruitment to racially, culturally, and socioeconomically diverse populations to improve external validity.<br>8. Ensure balanced randomization and consider individual neuroanatomical variability in future models beyond statistical covariates.<br>9. Use attention-matched control groups or measure social interaction to distinguish effects of interpersonal engagement.<br>10. Incorporate objective measurement tools (e.g., cognitive testing, functional assessments, biomarkers) to reduce self-report bias.<br>11. Include psychological and behavioral mediators in study designs to better understand pathways of change.<br>12. Screen for COVID-19 exposure and stratify analyses due to its potential long-term neurological impacts.<br>13. Measure and statistically adjust for these potential confounders to isolate the effects of the intervention.<br>14. Use standardized EEG setup protocols and training to reduce variability and improve signal reliability.

		15. Incorporate individualized, pathophysiology-based strategies into resistance training to maximize relevance and effectiveness.<br>16. Identify biological and psychosocial causes of cognitive frailty to inform targeted, multidomain interventions based on personalized needs.<br>17. Evaluate interactions between resistance training and pharmacologic treatments, as well as protein or vitamin supplementation.<br>18. Explore the pathophysiological mechanisms of cognitive frailty and how resistance training may influence them.<br>19. Integrate neuroimaging methods (e.g., MRI, PET) in longitudinal studies to investigate how resistance training affects brain structure (e.g., atrophy, white matter changes, amyloid burden).
**Multicomponent Exercise (ME)**		
	1. Small sample sizes (fewer than 100 participants)[117–152,153,154,155]<br>2. Uneven gender distribution across groups[117–119,121–132,134–137,139–141,143–148,150–152,156–172,173,153,154]<br>3. Lack of non-interventional or placebo control group[120,121,125,126,132,145,148,151,165,170,153]<br>4. High attrition rates (greater than 20% in either intervention or control groups)[125,128,131,132,135,137,140,146,149,159,167,168,153]<br>5. Short intervention duration (less than 12 weeks)[120–122,126,138,142,158,169]<br>6. No follow-up assessments[117–119,121,123–125,127–129,131,133–138,140,142–150,152,156,157,159–161,163,166,167,172,154,153,155]<br>7. Effects of combined components cannot be isolated[144]<br>8. Limited generalizability due to narrow sample characteristics[123,124,127,130,136,144,159,160,165,167–170]<br>9. Non-fasting Blood samples were collected[157]<br>10. No dietary intake assessment or control[157]<br>11. Potential for contamination due to shared community of intervention and control groups[118]<br>12. Unknown group differences in the risk factors (e.g., APOE, Vitamin B12, thyroid, inflammation)[119,142]<br>13. Possible influence of social contact[119,129,160]<br>14. Lack of blinding outcome assessors[126,137,140,148]<br>15. Physical activity levels and fall history at baseline not	1. Conduct large, multicenter trials across diverse settings to enhance statistical power and generalizability.<br>2. Recruit gender-balanced samples and explore gender-specific responses to multicomponent interventions.<br>3. Design control conditions with comparable social contact to isolate the true effect of exercise interventions.<br>4. Implement adherence strategies and evaluate participant engagement, satisfaction, and dropout reasons.<br>5. Explore optimal combinations of exercise duration, frequency, and intensity. Conduct longer-term interventions.<br>6. Include intermediate and long-term follow-up to assess sustainability of benefits and caregiver burden.<br>7. Use factorial designs to test individual and synergistic effects of specific exercise components.<br>8. Expand to more heterogeneous populations; consider restricting samples (e.g., only older adults) for focus.<br>9. Standardize biomarker collection procedures (e.g., fasting samples) for biochemical validity.<br>10. Monitor nutrient intake and explore effects of dietary supplementation on intervention outcomes.<br>11. Use cluster randomization or separate recruitment streams to avoid experimental contamination.<br>12. Collect biomarker and genotypic data to control for individual-level

recorded[126]
16. Reliance on self-evaluations as primary outcome[130]
17. Large heterogeneity or unclear dementia diagnoses[132,152]
18. Recruitment bias from Alzheimer's' Association chapters[133]
19. Limited executive function testing (e.g., only TMT-B)[133]
20. Underpowered subgroup analyses[166]
21. No subgroup analysis conducted[135]
22. Confounding from polypharmacy[138]
23. Comorbidities not accounted[139,146]
24. RPE used as sole measure of intensity[139]
25. No a prior power calculation[140]
26. Unclear whether effects stem from physical activity or other aspects (e.g., staff engagement)[140]
27. Physical measurements collected by caregivers[141]
28. Baseline group differences[167]
29. Missing post-intervention data at key time points (e.g., 1 and 3 months)[167]
30. Heterogeneity in intervention delivery (e.g., 27 physiotherapists)[170]
31. No habitual physical activity data collected[170]
32. Questionnaires may have been completed by untrained staff [170]
33. MMSE conducted up to 1 month before other assessments[171]
34. Attention control may include cognitive stimulation elements[171]
35. Mixed cognitive profiles among participants[171]
36. Inconsistent monitoring/reporting of exercise intensity[146,151]
37. Incomplete cognitive diagnostic data in care notes[151]
38. Limited resources for cognitive testing across sites[151]
39. Selection bias[153]
40. COVID-19 impact[155]

risk factors of cognitive decline.

13. Control for social interaction by designing attention-matched control conditions.
14. Use blinded outcome assessors to reduce measurement bias.
15. Collect detailed baseline health and mobility data to adjust for pre-existing differences.
16. Include validated, performance-based, and objective outcome measures.
17. Use standardized diagnostic criteria and stratify by dementia or MCI subtype (amnestic/non-amnestic).
18. Diversify recruitment sources to reduce selection bias.
19. Use comprehensive cognitive batteries to detect subtle changes in executive function.
20. Ensure sufficient power for subgroup analyses and conduct them a priori by dementia type, gender, or age.
21. Track and adjust for use of multiple medications in outcome models.
22. Screen for and adjust comorbidities and body composition to reduce confounding.
23. Use objective tools (e.g., HR monitors, accelerometers) to monitor and maintain exercise intensity.
24. Conduct sample size calculations based on intended outcomes to ensure adequate power.
25. Disentangle physical activity effects from environmental/contextual components using multi-arm designs.
26. Ensure assessments are conducted by trained, blinded professionals.
27. Use proper randomization and baseline-adjusted analyses.
28. Improve data retention and use imputation methods where appropriate.
29. Standardize intervention delivery using manuals, training, and fidelity checks.
30. Record background PA to control for additional activity outside the intervention.
31. Train all assessors or require supervision by qualified personnel.
32. Align cognitive and physical testing timelines to ensure consistency.
33. Carefully design control interventions to avoid overlap with active components.
34. Stratify by cognitive subtype (e.g., MCI vs dementia; amnestic vs non-amnestic) to clarify intervention effects.

35. Report and monitor exercise intensity consistently across sites and participants.
36. Use standardized assessments to collect detailed cognitive diagnostic data.
37. Secure adequate resources and standardize procedures across locations.
38. Include economic evaluations and cost-effectiveness analyses to inform policy and sustainability.
39. Evaluate participant satisfaction, enjoyment, and acceptability as part of feasibility outcomes.
40. Explore novel interventions (e.g., dual-task, VR, cognitive-motor integration) for enhanced cognitive engagement.
41. Incorporate biomarkers to understand physiological pathways and intervention mechanisms.
42. Investigate how exercise dose (duration, frequency, intensity) relates to cognitive and physical outcomes.

[illegible]eferences

[illegible]. Esmail A, Vrinceanu T, Lussier M, et al. Effects of Dance/Movement Training vs. Aerobic Exercise Training on cognition, physical fitness and quality of life in older adults: A randomized controlled trial. *J Bodyw Mov Ther*. 2020;24(1):212-220. doi:10.1016/j.jbmt.2019.05.004

[illegible]. Bisbe M, Fuente-Vidal A, López E, et al. Comparative Cognitive Effects of Choreographed Exercise and Multimodal Physical Therapy in Older Adults with Amnestic Mild Cognitive Impairment: Randomized Clinical Trial. *Journal of Alzheimer's Disease*. 2020;73(2):769-783. doi:10.3233/JAD-190552

[illegible]. Kropacova S, Mitterova K, Klobusiakova P, et al. Cognitive effects of dance-movement intervention in a mixed group of seniors are not dependent on hippocampal atrophy. *J Neural Transm*. 2019;126(11):1455-1463. doi:10.1007/s00702-019-02068-y

[illegible]. Qi M, Zhu Y, Zhang L, Wu T, Wang J. The effect of aerobic dance intervention on brain spontaneous activity in older adults with mild cognitive impairment: A resting-state functional MRI study. *Exp Ther Med*. Published online 2018:715-722. doi:10.3892/etm.2018.7006

[illegible]. Franco MR, Sherrington C, Tiedemann A, et al. Effect of Senior Dance (DanSE) on Fall Risk Factors in Older Adults: A Randomized Controlled Trial. *Phys Ther*. 2020;100(4):600-608. doi:10.1093/ptj/pzz187

[illegible]. Blumen HM, Ayers E, Wang C, Ambrose AF, Jayakody O, Verghese J. Randomized Controlled Trial of Social Ballroom Dancing and Treadmill Walking: Preliminary Findings on Executive Function and Neuroplasticity From Dementia-at-Risk Older Adults. *J Aging Phys Act*. 2023;31(4):589-599. doi:10.1123/japa.2022-0176

[illegible]. Zhu Y, Wu H, Qi M, et al. Effects of a specially designed aerobic dance routine on mild cognitive impairment. *Clin Interv Aging*. 2018;13:1691-1700. doi:10.2147/CIA.S163067

[illegible]. Song D, Yu D, Liu T, Wang J. Effect of an Aerobic Dancing Program on Sleep Quality for Older Adults With Mild Cognitive Impairment and Poor Sleep: A Randomized Controlled Trial. *J Am Med Dir Assoc*. 2024;25(3):494-499. doi:10.1016/j.jamda.2023.09.020

[illegible]. Van de Winckel A, Feys H, De Weerdt W, Dom R. Cognitive and behavioural effects of music-based exercises in patients with dementia. *Clin Rehabil*. 2004;18(3):253-260. doi:10.1191/0269215504cr750oa

[illegible]0. Bracco L, Pinto-Carral A, Hillaert L, Mourey F. Tango-therapy vs physical exercise in older people with dementia; a randomized controlled trial. *BMC Geriatr*. 2023;23(1):1-13. doi:10.1186/s12877-023-04342-x

1. Zhu Y, Gao Y, Guo C, et al. Effect of 3-Month Aerobic Dance on Hippocampal Volume and Cognition in Elderly People With Amnestic Mild Cognitive Impairment: A Randomized Controlled Trial. *Front Aging Neurosci*. 2022;14(March):1-10. doi:10.3389/fnagi.2022.771413
2. Thiel U, Stiebler M, Labott BK, et al. DiADEM—Dance against Dementia—Effect of a Six-Month Dance Intervention on Physical Fitness in Older Adults with Mild Cognitive Impairment: A Randomized, Controlled Trial. *J Pers Med*. 2024;14(8). doi:10.3390/jpm14080888
3. Sánchez-Alcalá M, Aibar-Almazán A, Carcelén-Fraile M del C, et al. Effects of Dance-Based Aerobic Training on Frailty and Cognitive Function in Older Adults with Mild Cognitive Impairment: A Randomized Controlled Trial. *Diagnostics*. 2025;15(3):1-13. doi:10.3390/diagnostics15030351
4. Lazarou I, Parastatidis T, Tsolaki A, et al. International Ballroom Dancing Against Neurodegeneration: A Randomized Controlled Trial in Greek Community-Dwelling Elders With Mild Cognitive impairment. *Am J Alzheimers Dis Other Demen*. 2017;32(8):489-499. doi:10.1177/1533317517725813
5. Chang J, Zhu W, Zhang J, et al. The Effect of Chinese Square Dance Exercise on Cognitive Function in Older Women With Mild Cognitive Impairment: The Mediating Effect of Mood Status and Quality of Life. *Front Psychiatry*. 2021;12(July). doi:10.3389/fpsyt.2021.711079
6. Ho RTH, Fong TCT, Chan WC, et al. Psychophysiological Effects of Dance Movement Therapy and Physical Exercise on Older Adults with Mild Dementia: A Randomized Controlled Trial. *Journals of Gerontology - Series B Psychological Sciences and Social Sciences*. 2018;75(3):560-570. doi:10.1093/geronb/gby145
7. Doi T, Verghese J, Makizako H, et al. Effects of Cognitive Leisure Activity on Cognition in Mild Cognitive Impairment: Results of a Randomized Controlled Trial. *J Am Med Dir Assoc*. 2017;18(8):686-691. doi:10.1016/j.jamda.2017.02.013
8. Sánchez-Alcalá M, Aibar-Almazán A, Hita-Contreras F, et al. Effects of Dance-Based Aerobic Training on Mental Health and Quality of Life in Older Adults with Mild Cognitive Impairment. *J Pers Med*. 2024;14(8):1-16. doi:10.3390/jpm14080844
9. Hughes TF, Flatt JD, Fu B, Butters MA, Chang CCH, Ganguli M. Interactive video gaming compared with health education in older adults with mild cognitive impairment: A feasibility study. *Int J Geriatr Psychiatry*. 2014;29(9):890-898. doi:10.1002/gps.4075
[illegible]0. Sabbagh et al. Sensor-based balance training with motion feedback in people with mild cognitive impairment. 2016;53(6):945-958. doi:10.1682/JRRD.2015.05.0089.Sensor-based
[illegible]1. Eggenberger P, Schumacher V, Angst M, Theill N, de Bruin ED. Does multicomponent physical exercise with simultaneous cognitive training boost cognitive performance in older adults? A 6-month randomized controlled trial with a 1-year follow-up. *Clin Interv Aging*. 2015;10:1335-1349. doi:10.2147/CIA.S87732
[illegible]2. Liu CL, Cheng FY, Wei MJ, Liao YY. Effects of Exergaming-Based Tai Chi on Cognitive Function and Dual-Task Gait Performance in Older Adults With Mild Cognitive Impairment: A Randomized Control Trial. *Front Aging Neurosci*. 2022;14(March). doi:10.3389/fnagi.2022.761053
[illegible]3. Padala KP, Padala PR, Malloy TR, et al. Wii-fit for improving gait and balance in an assisted living facility: A pilot study. *J Aging Res*. 2012;2012:6-11. doi:10.1155/2012/597573
[illegible]4. Liao YY, Chen IH, Hsu WC, Tseng HY, Wang RY. Effect of exergaming versus combined exercise on cognitive function and brain activation in frail older adults: A randomised controlled trial. *Ann Phys Rehabil Med*. 2021;64(5):101492. doi:10.1016/j.rehab.2021.101492
[illegible]5. Wu S, Ji H, Won J, Jo EA, Kim YS, Park JJ. The Effects of Exergaming on Executive and Physical Functions in Older Adults With Dementia: Randomized Controlled Trial. *J Med Internet Res*. 2023;25:1-17. doi:10.2196/39993
[illegible]6. Swinnen N, Vandenbulcke M, de Bruin ED, et al. The efficacy of exergaming in people with major neurocognitive disorder residing in long-term care facilities: a pilot randomized controlled trial. *Alzheimers Res Ther*. 2021;13(1):1-13. doi:10.1186/s13195-021-00806-7
[illegible]7. Uğur F, Sertel M. Wii Fit Exercise's Effects on Muscle Strength and Fear of Falling in Older Adults With Alzheimer Disease: A Randomized Controlled Trial. *J Aging Phys Act*. 2025;33(2):181-191. doi:10.1123/japa.2023-0428
[illegible]8. Karssemeijer EGA, Aaronson JA, Bossers WJR, Donders R, Olde Rikkert MGM, Kessels RPC. The quest for synergy between physical exercise and cognitive stimulation via exergaming in people with dementia: A randomized controlled trial. *Alzheimers Res Ther*. 2019;11(1):1-13. doi:10.1186/s13195-018-0454-z
[illegible]9. van Santen J, Dröes RM, Twisk JWR, Blanson Henkemans OA, van Straten A, Meiland FJM. Effects of Exergaming on Cognitive and Social Functioning of People

with Dementia: A Randomized Controlled Trial. *J Am Med Dir Assoc*. 2020;21(12):1958-1967.e5. doi:10.1016/j.jamda.2020.04.018

[illegible]0. Zheng J, Yu P, Chen X. An Evaluation of the Effects of Active Game Play on Cognition, Quality of Life and Depression for Older People with Dementia. *Clin Gerontol*. 2022;45(4):1034-1043. doi:10.1080/07317115.2021.1980170

[illegible]1. Grzenda A, Siddarth P, Milillo MM, Aguilar-Faustino Y, Khalsa DS, Lavretsky H. Cognitive and immunological effects of yoga compared to memory training in older women at risk for alzheimer's disease. *Transl Psychiatry*. 2024;14(1):1-11. doi:10.1038/s41398-024-02807-0

[illegible]2. Khanthong P, Sriyakul K, Dechakhamphu A, Krajarng A, Kamalashiran C, Tungsukruthai P. Traditional Thai exercise (Ruesi Dadton) for improving motor and cognitive functions in mild cognitive impairment: a randomized controlled trial. *J Exerc Rehabil*. 2021;17(5):331-338. doi:10.12965/JER.2142542.271

[illegible]3. Tremont G, Davis J, Ott BR, et al. Feasibility of a Yoga Intervention for Individuals with Mild Cognitive Impairment: A Randomized Controlled Trial. *Journal of Integrative and Complementary Medicine*. 2022;28(3):250-260. doi:10.1089/jicm.2021.0204

[illegible]4. Kashyap M, Rai NK, Singh R, et al. Effect of Early Yoga Practice on Post Stroke Cognitive Impairment. 2022;22(4):2019. doi:10.4103/aian.AIAN

[illegible]5. Eyre HA, Siddarth P, Acevedo B, et al. A randomized controlled trial of Kundalini yoga in mild cognitive impairment. *Int Psychogeriatr*. 2017;29(4):557-567. doi:10.1017/S1041610216002155

[illegible]6. Li F, Harmer P, Fitzgerald K, Winters-Stone K. A cognitively enhanced online Tai Ji Quan training intervention for community-dwelling older adults with mild cognitive impairment: A feasibility trial. *BMC Geriatr*. 2022;22(1):1-13. doi:10.1186/s12877-021-02747-0

[illegible]7. Sungkarat S, Boripuntakul S, Kumfu S, Lord SR, Chattipakorn N. Tai Chi Improves Cognition and Plasma BDNF in Older Adults With Mild Cognitive Impairment: A Randomized Controlled Trial. *Neurorehabil Neural Repair*. 2018;32(2):142-149. doi:10.1177/1545968317753682

[illegible]8. Li F, Harmer P, Voit J, Chou LS. Implementing an online virtual falls prevention intervention during a public health pandemic for older adults with mild cognitive impairment: A feasibility trial. *Clin Interv Aging*. 2021;16:973-983. doi:10.2147/CIA.S306431

[illegible]9. Jiayuan Z, Xiang-Zi J, Li-Na M, Jin-Wei Y, Xue Y. Effects of Mindfulness-Based Tai Chi Chuan on Physical Performance and Cognitive Function among Cognitive Frailty Older Adults: A Six-Month Follow-Up of a Randomized Controlled Trial. *Journal of Prevention of Alzheimer's Disease*. 2022;9(1):104-112. doi:10.14283/jpad.2021.40

[illegible]0. Lin M, Liu W, Ma C, et al. Tai Chi-Induced Exosomal LRP1 is Associated With Memory Function and Hippocampus Plasticity in aMCI Patients. *American Journal of Geriatric Psychiatry*. 2024;32(10):1215-1230. doi:10.1016/j.jagp.2024.04.012

[illegible]1. Yu AP, Chin EC, Yu DJ, et al. Tai Chi versus conventional exercise for improving cognitive function in older adults: a pilot randomized controlled trial. *Sci Rep*. 2022;12(1):1-15. doi:10.1038/s41598-022-12526-5

[illegible]2. Huang N, Li W, Rong X, et al. Effects of a Modified Tai Chi Program on Older People with Mild Dementia: A Randomized Controlled Trial. *Journal of Alzheimer's Disease*. 2019;72(3):947-956. doi:10.3233/JAD-190487

[illegible]3. Liu JYW, Kwan RYC, Lai CKY, Hill KD. A simplified 10-step Tai-chi programme to enable people with dementia to improve their motor performance: a feasibility study. *Clin Rehabil*. 2018;32(12):1609-1623. doi:10.1177/0269215518786530

[illegible]4. Chan AWK, Yu DSF, Choi KC, Lee DTF, Sit JWH, Chan HYL. Tai chi qigong as a means to improve night-time sleep quality among older adults with cognitive impairment: A pilot randomized controlled trial. *Clin Interv Aging*. 2016;11:1277-1286. doi:10.2147/CIA.S111927

[illegible]5. Nyman SR, Ingram W, Sanders J, et al. Randomised controlled trial of the effect of tai chi on postural balance of people with dementia. *Clin Interv Aging*. 2019;14:2017-2029. doi:10.2147/CIA.S228931

[illegible]6. Canan Okuyan ED. The effectiveness of Tai Chi Chuan on fear of movement, prevention of falls, physical activity, and cognitive status in older adults with mild cognitive impairment: A randomized controlled trial. *Perspect Psychiatr Care*. 2021;57(3):1273-1281. doi:10.1111/ppc.12684

[illegible]7. Su H, Wang H, Meng L, Bush E. The effects of Baduanjin exercise on the subjective memory complaint of older adults: A randomized controlled trial. *Medicine (United States)*. 2021;100(30):E25442. doi:10.1097/MD.0000000000025442

[illegible]8. Zheng G, Zheng Y, Xiong Z, Ye B. Effect of Baduanjin exercise on cognitive function in patients with post-stroke cognitive impairment: a randomized controlled trial. *Clin Rehabil*. 2020;34(8):1028-1039. doi:10.1177/0269215520930256

[illegible]9. Zheng G, Ye B, Xia R, et al. Traditional Chinese Mind-Body Exercise Baduanjin Modulate Gray Matter and Cognitive Function in Older Adults with Mild Cognitive Impairment: A Brain Imaging Study. *Brain Plasticity*. 2021;7(2):131-142. doi:10.3233/bpl-210121

[illegible]0. Li K, Yu H, Kortas JA, Lin X, Lipowski M. The effect of 12 weeks of Baduanjin exercise on cognitive function, lower limb balance and quality of life of the elderly with mild cognitive impairment: a randomized controlled trial. *Gazzetta Medica Italiana Archivio per le Scienze Mediche*. 2022;181(11):811-823. doi:10.23736/S0393-3660.22.04802-1

[illegible]1. Luo SS, Chen L, Wang GB, Wang YG, Su XY. Effects of long-term Wuqinxi exercise on working memory in older adults with mild cognitive impairment. *Eur Geriatr Med*. 2022;13(6):1327-1333. doi:10.1007/s41999-022-00709-2

[illegible]2. Chang CL, Lin TK, Pan CY, et al. Distinct effects of long-term Tai Chi Chuan and aerobic exercise interventions on motor and neurocognitive performance in early-stage Parkinson's disease: a randomized controlled trial. *Eur J Phys Rehabil Med*. 2024;60(4):621-633. doi:10.23736/S1973-9087.24.08166-8

[illegible]3. Gao R, Greiner C, Ryuno H, Zhang X. Effects of Tai Chi on physical performance, sleep, and quality of life in older adults with mild to moderate cognitive impairment. *BMC Complement Med Ther*. 2024;24(1). doi:10.1186/s12906-024-04705-w

[illegible]4. Hsu CY, Yeh ML, Liu YCE. Three-month Chan-Chuang qigong program improves physical performance and quality of life of patients with cognitive impairment: A randomized controlled trial. *Res Nurs Health*. 2022;45(3):327-336. doi:10.1002/nur.22219

[illegible]5. Lam LCW, Chau RCM, Wong BML, et al. A 1-Year Randomized Controlled Trial Comparing Mind Body Exercise (Tai Chi) With Stretching and Toning Exercise on Cognitive Function in Older Chinese Adults at Risk of Cognitive Decline. *J Am Med Dir Assoc*. 2012;13(6):568.e15-568.e20. doi:10.1016/j.jamda.2012.03.008

[illegible]6. Chen Y, Qin J, Tao L, et al. Effects of Tai Chi Chuan on Cognitive Function in Adults 60 Years or Older With Type 2 Diabetes and Mild Cognitive Impairment in China: A Randomized Clinical Trial. *JAMA Netw Open*. 2023;6(4):E237004. doi:10.1001/jamanetworkopen.2023.7004

[illegible]7. Cheng ST, Chow PK, Song YQ, et al. Mental and physical activities delay cognitive decline in older persons with dementia. *American Journal of Geriatric Psychiatry*. 2014;22(1):63-74. doi:10.1016/j.jagp.2013.01.060

[illegible]8. Tomoto T, Liu J, Tseng BY, et al. One-Year Aerobic Exercise Reduced Carotid Arterial Stiffness and Increased Cerebral Blood Flow in Amnestic Mild Cognitive Impairment. *Journal of Alzheimer's Disease*. 2021;80(2):841-853. doi:10.3233/JAD-201456

[illegible]9. Donnezan et al. Effects of simultaneous aerobic and cognitive training on executive functions, cardiovascular fitness and functional abilities in older adults with mild cognitive impairment. *Ment Health Phys Act*. 2018;15(April):78-87. doi:10.1016/j.mhpa.2018.06.001

[illegible]0. Baker LD, Frank LL, Foster-Schubert K, et al. Effects of aerobic exercise on mild cognitive impairment: A controlled trial. *Arch Neurol*. 2010;67(1):71-79. doi:10.1001/archneurol.2009.307

[illegible]1. Tsai CL, Pai MC, Ukropec J, Ukropcová B. Distinctive Effects of Aerobic and Resistance Exercise Modes on Neurocognitive and Biochemical Changes in Individuals with Mild Cognitive Impairment. *Curr Alzheimer Res*. 2019;16(4):316-332. doi:10.2174/1567205016666190228125429

[illegible]2. Hsu CL, Best JR, Davis JC, et al. Aerobic exercise promotes executive functions and impacts functional neural activity among older adults with vascular cognitive impairment. *Br J Sports Med*. 2018;52(3):184-191. doi:10.1136/bjsports-2016-096846

[illegible]3. Rojasavastera R, Bovonsunthonchai S, Hiengkaew V, Senanarong V. Action observation combined with gait training to improve gait and cognition in elderly with mild cognitive impairment a randomized controlled trial. *Dementia e Neuropsychologia*. 2020;14(2):118-127. doi:10.1590/1980-57642020dn14-020004

[illegible]4. Brydges CR, Liu-Ambrose T, Bielak AAM. Using intraindividual variability as an indicator of cognitive improvement in a physical exercise intervention of older women with mild cognitive impairment. *Neuropsychology*. 2020;34(8):825-834. doi:10.1037/neu0000638

[illegible]5. Morris JK, Vidoni ED, Johnson DK, et al. Aerobic exercise for Alzheimer's disease: A randomized controlled pilot trial. *PLoS One*. 2017;12(2):1-14. doi:10.1371/journal.pone.0170547

66. Wei X hong, Ji L li. Effect of handball training on cognitive ability in elderly with mild cognitive impairment. *Neurosci Lett*. 2014;566:98-101. doi:10.1016/j.neulet.2014.02.035
67. Damirchi A, Hosseini F, Babaei P. Mental Training Enhances Cognitive Function and BDNF More Than Either Physical or Combined Training in Elderly Women With MCI: A Small-Scale Study. *Am J Alzheimers Dis Other Demen*. 2018;33(1):20-29. doi:10.1177/1533317517727068
68. Kohanpour MA, Peeri M, Azarbayjani MA. The effects of aerobic exercise with lavender essence use on cognitive state and serum brain-derived neurotrophic factor levels in elderly with mild cognitive impairment. *Journal of HerbMed Pharmacology*. 2017;6(2):80-84.
69. Karthikeyan T. Therapeutic effects of home-based exercise of geriatrics for the management of cognitive impairment. *ES J Public Health*. 2020;1(1):1003. www.escientificlibrary.com
70. Krootnark K, Chaikeeree N, Saengsirisuwan V, Boonsinsukh R. Effects of low-intensity home-based exercise on cognition in older persons with mild cognitive impairment: a direct comparison of aerobic versus resistance exercises using a randomized controlled trial design. *Front Med (Lausanne)*. 2024;11(June):1-11. doi:10.3389/fmed.2024.1392429
71. Liu IT, Lee WJ, Lin SY, Chang ST, Kao CL, Cheng YY. Therapeutic Effects of Exercise Training on Elderly Patients With Dementia: A Randomized Controlled Trial. *Arch Phys Med Rehabil*. 2020;101(5):762-769. doi:10.1016/j.apmr.2020.01.012
72. Dillon K, Prapavessis H. REducing SEDENTary behavior among mild to moderate cognitively impaired assisted living residents: A pilot randomized controlled trial (RESEDENT study). *J Aging Phys Act*. 2021;29(1):27-35. doi:10.1123/JAPA.2019-0440
73. Yang SY, Shan CL, Qing H, et al. The Effects of Aerobic Exercise on Cognitive Function of Alzheimer's Disease Patients. *CNS Neurol Disord Drug Targets*. 2015;14(10):1292-1297. doi:10.2174/1871527315666151111123319
74. Choi W, Lee S. Ground kayak paddling exercise improves postural balance, muscle performance, and cognitive function in older adults with mild cognitive impairment: A randomized controlled trial. *Medical Science Monitor*. 2018;24:3909-3915. doi:10.12659/MSM.908248
75. Yu DJ, Yu AP, Bernal JDK, et al. Effects of exercise intensity and frequency on improving cognitive performance in middle-aged and older adults with mild cognitive impairment: A pilot randomized controlled trial on the minimum physical activity recommendation from WHO. *Front Physiol*. 2022;13(September):1-12. doi:10.3389/fphys.2022.1021428
76. Fischbacher M, Chocano-Bedoya PO, Meyer U, et al. Safety and feasibility of a Dalcroze eurhythmics and a simple home exercise program among older adults with mild cognitive impairment (MCI) or mild dementia: The MOVE for your MIND pilot trial. *Pilot Feasibility Stud*. 2020;6(1):1-8. doi:10.1186/s40814-020-00645-7
77. Khattak HG, Ahmad Z, Arshad H, Anwar K. Effect of aerobic exercise on cognition in elderly persons with mild cognitive impairment. *Rawal Medical Journal*. 2022;47(3):698-701. doi:10.5455/rmj.20210713072242
78. Varela S, Ayán C, Cancela JM, Martín V. Effects of two different intensities of aerobic exercise on elderly people with mild cognitive impairment: A randomized pilot study. *Clin Rehabil*. 2012;26(5):442-450. doi:10.1177/0269215511425835
79. Miu D, Edin F, Szeto S, Mak Y. A randomised controlled trial on the effect of exercise on physical, cognitive and affective function in dementia subjects. *Asian Journal of Gerontology & Geriatrics*. 2008;3(1):8-16.
80. Arcoverde C, Deslandes A, Moraes H, et al. Treadmill training as an augmentation treatment for Alzheimer's disease: A pilot randomized controlled study. *Arq Neuropsiquiatr*. 2014;72(3):190-196. doi:10.1590/0004-282X20130231
81. Angiolillo A, Leccese D, Ciccotelli S, et al. Effects of Nordic walking in Alzheimer's disease: A single-blind randomized controlled clinical trial. *Heliyon*. 2023;9(5):e15865. doi:10.1016/j.heliyon.2023.e15865
82. Enette L, Vogel T, Merle S, et al. Effect of 9 weeks continuous vs. interval aerobic training on plasma BDNF levels, aerobic fitness, cognitive capacity and quality of life among seniors with mild to moderate Alzheimer's disease: A randomized controlled trial. *European Review of Aging and Physical Activity*. 2020;17(1):1-16. doi:10.1186/s11556-019-0234-1

3. Phoemsapthawee et al. The Benefit of Arm Swing Exercise on Cognitive Performance in Older Women with Mild Cognitive Impairment. *Journal of Exercise Physiology*. 2016;8(1):11-25.
4. Eggermont LHP, Swaab DF, Hol EM, Scherder EJA. Walking the line: A randomised trial on the effects of a short term walking programme on cognition in dementia. *J Neurol Neurosurg Psychiatry*. 2009;80(7):802-804. doi:10.1136/jnnp.2008.158444
5. Lowery D, Cerga-Pashoja A, Iliffe S, et al. The effect of exercise on behavioural and psychological symptoms of dementia: The EVIDEM-E randomised controlled clinical trial. *Int J Geriatr Psychiatry*. 2014;29(8):819-827. doi:10.1002/gps.4062
6. Guzel I, Can F. The effects of different exercise types on cognitive and physical functions in dementia patients: A randomized comparative study. *Arch Gerontol Geriatr*. 2024;119(18):105321. doi:10.1016/j.archger.2023.105321
7. Venturelli M, Scarsini R, Schena F. Six-month walking program changes cognitive and ADL performance in patients with Alzheimer. *Am J Alzheimers Dis Other Demen*. 2011;26(5):381-388. doi:10.1177/1533317511418956
8. Scherder EJA, Van Paasschen J, Deijen JB, et al. Physical activity and executive functions in the elderly with mild cognitive impairment. *Aging Ment Health*. 2005;9(3):272-280. doi:10.1080/13607860500089930
9. Amjad I, Toor H, Niazi IK, et al. Therapeutic effects of aerobic exercise on EEG parameters and higher cognitive functions in mild cognitive impairment patients. *International Journal of Neuroscience*. 2019;129(6):551-562. doi:10.1080/00207454.2018.1551894
0. Abbas RL, Saab IM, Al-Sharif HK, Naja N, El-Khatib A. Effect of Adding Motorized Cycle Ergometer Over Exercise Training on Balance in Older Adults with Dementia: A Randomized Controlled Trial. *Exp Aging Res*. 2023;49(2):100-111. doi:10.1080/0361073X.2022.2046947
1. Abd El-Kader SM, Al-Jiffri OH. Aerobic exercise improves quality of life, psychological well-being and systemic inflammation in subjects with alzheimer's disease. *Afr Health Sci*. 2016;16(4):1045-1055. doi:10.4314/ahs.v16i4.22
2. Yu F, Salisbury D, Mathiason MA. Inter-individual differences in the responses to aerobic exercise in Alzheimer's disease: Findings from the FIT-AD trial. *J Sport Health Sci*. 2021;10(1):65-72. doi:10.1016/j.jshs.2020.05.007
3. Shimada H, Lee S, Akishita M, et al. Effects of golf training on cognition in older adults: A randomised controlled trial. *J Epidemiol Community Health (1978)*. 2018;72(10):944-950. doi:10.1136/jech-2017-210052
4. Ihle-Hansen H, Langhammer B, Lydersen S, Gunnes M, Indredavik B, Askim T. A physical activity intervention to prevent cognitive decline after stroke: Secondary results from the life after stroke study, an 18-month randomized controlled trial. *J Rehabil Med*. 2019;51(9):646-651. doi:10.2340/16501977-2588
5. Song D, Yu DSF. Effects of a moderate-intensity aerobic exercise programme on the cognitive function and quality of life of community-dwelling elderly people with mild cognitive impairment: A randomised controlled trial. *Int J Nurs Stud*. 2019;93:97-105. doi:10.1016/j.ijnurstu.2019.02.019
6. Stuckenschneider T, Sanders ML, Devenney KE, et al. NeuroExercise: The Effect of a 12-Month Exercise Intervention on Cognition in Mild Cognitive Impairment—A Multicenter Randomized Controlled Trial. *Front Aging Neurosci*. 2021;12(January):1-12. doi:10.3389/fnagi.2020.621947
7. Makino T, Umegaki H, Ando M, et al. Effects of Aerobic, Resistance, or Combined Exercise Training among Older Adults with Subjective Memory Complaints: A Randomized Controlled Trial. *Journal of Alzheimer's Disease*. 2021;82(2):701-717. doi:10.3233/JAD-210047
8. Nakatsuka M, Nakamura K, Hamanosono R, et al. A Cluster Randomized Controlled Trial of Nonpharmacological Interventions for Old-Old Subjects with a Clinical Dementia Rating of 0.5: The Kurihara Project. *Dement Geriatr Cogn Dis Extra*. 2015;5(2):221-232. doi:10.1159/000380816
9. L.F. Law et al. Effects of functional task exercise on everyday problem-solving ability and functional status in older adults with mild cognitive impairment—a randomised controlled trial. *Age Ageing*. 2021;51(7):1-11. doi:10.1093/ageing/afac144
00. Cancela JM, Ayán C, Varela S, Seijo M. Effects of a long-term aerobic exercise intervention on institutionalized patients with dementia. *J Sci Med Sport*. 2016;19(4):293-298. doi:10.1016/j.jsams.2015.05.007
01. Baker LD, Pa JA, Katula JA, et al. Effects of exercise on cognition and Alzheimer's biomarkers in a randomized controlled trial of adults with mild cognitive impairment:

The EXERT study. *Alzheimer's and Dementia*. 2025;21(4):1-17. doi:10.1002/alz.14586
02. Huang X, Zhang S, Zhao X, et al. Feasibility and effects of remotely supervised aerobic training and resistance training in older adults with mild cognitive impairment: A pilot three-arm randomised controlled trial. *Gen Psychiatr*. 2025;38(2). doi:10.1136/gpsych-2024-101858
03. Nagamatsu LS, Chan A, Davis JC, et al. Physical activity improves verbal and spatial memory in older adults with probable mild cognitive impairment: A 6-month randomized controlled trial. *J Aging Res*. 2013;2013(Mci). doi:10.1155/2013/861893
04. Fernandez-Gonzalo R, Fernandez-Gonzalo S, Turon M, Prieto C, Tesch PA, García-Carreira MDC. Muscle, functional and cognitive adaptations after flywheel resistance training in stroke patients: A pilot randomized controlled trial. *J Neuroeng Rehabil*. 2016;13(1):1-11. doi:10.1186/s12984-016-0144-7
05. Lv J, Liu Y. Effects of momentum-based dumbbell training on motor control in older adults with mild cognitive impairment. *Chinese Journal of Rehabilitation Medicine*. 2019;34(5):544-550. doi:10.3969/j.issn.1001-1242.2019.05.009
06. Vints WAJ, Gökçe E, Šeikinaitė J, et al. Resistance training's impact on blood biomarkers and cognitive function in older adults with low and high risk of mild cognitive impairment: a randomized controlled trial. *European Review of Aging and Physical Activity*. 2024;21(1):1-15. doi:10.1186/s11556-024-00344-9
07. Yoon DH, Lee JY, Song W. Effects of Resistance Exercise Training on Cognitive Function and Physical Performance in Cognitive Frailty: A Randomized Controlled Trial. *Journal of Nutrition, Health and Aging*. 2018;22(8):944-951. doi:10.1007/s12603-018-1090-9
08. Yoon DH, Kang D, Kim HJ, Kim JS, Song HS, Song W. Effect of elastic band-based high-speed power training on cognitive function, physical performance and muscle strength in older women with mild cognitive impairment. *Geriatr Gerontol Int*. 2017;17(5):765-772. doi:10.1111/ggi.12784
09. Lee DW, Yoon DH, Lee JY, Panday SB, Park J, Song W. Effects of High-Speed Power Training on Neuromuscular and Gait Functions in Frail Elderly with Mild Cognitive Impairment Despite Blunted Executive Functions: A Randomized Controlled Trial. *Journal of Frailty and Aging*. 2020;9(3):179-184. doi:10.14283/jfa.2020.23
10. Hong SG, Kim JH, Jun TW. Effects of 12-week resistance exercise on electroencephalogram patterns and cognitive function in the elderly with mild cognitive impairment: A randomized controlled trial. *Clinical Journal of Sport Medicine*. 2018;28(6):500-508. doi:10.1097/JSM.0000000000000476
11. Venturelli M, Lanza M, Muti E, Schena F. Positive effects of physical training in activity of daily living-dependent older adults. *Exp Aging Res*. 2010;36(2):190-205. doi:10.1080/03610731003613771
12. Holthoff VA, Marschner K, Scharf M, et al. Effects of physical activity training in patients with alzheimer's dementia: Results of a pilot RCT study. *PLoS One*. 2015;10(4):1-11. doi:10.1371/journal.pone.0121478
13. Baek JE, Hyeon SJ, Kim M, Cho HY, Hahm SC. Effects of dual-task resistance exercise on cognition, mood, depression, functional fitness, and activities of daily living in older adults with cognitive impairment: a single-blinded, randomized controlled trial. *BMC Geriatr*. 2024;24(1):1-12. doi:10.1186/s12877-024-04942-1
14. Kušleikienė S, Ziv G, Vints WAJ, et al. Cognitive gains and cortical thickness changes after 12 weeks of resistance training in older adults with low and high risk of mild cognitive impairment: Findings from a randomized controlled trial. *Brain Res Bull*. 2025;222(September 2024). doi:10.1016/j.brainresbull.2025.111249
15. Singh et al. The Study of Mental and Resistance Training (SMART) Study-Resistance Training and/or Cognitive Training in Mild Cognitive Impairment: A Randomized, Double-Blind, Double-Sham Controlled Trial. *J Am Med Dir Assoc*. 2014;15(12):873-880. doi:10.1016/j.jamda.2014.09.010
16. Wang L, Wu B, Tao H, et al. Effects and mediating mechanisms of a structured limbs-exercise program on general cognitive function in older adults with mild cognitive impairment: A randomized controlled trial. *Int J Nurs Stud*. 2020;110:103706. doi:10.1016/j.ijnurstu.2020.103706
17. Doi T, Makizako H, Shimada H, et al. Effects of multicomponent exercise on spatial-temporal gait parameters among the elderly with amnestic mild cognitive impairment (aMCI): Preliminary results from a randomized controlled trial (RCT). *Arch Gerontol Geriatr*. 2013;56(1):104-108. doi:10.1016/j.archger.2012.09.003
18. Li L, Liu M, Zeng H, Pan L. Multi-component exercise training improves the physical and cognitive function of the elderly with mild cognitive impairment: A six-month randomized controlled trial. *Ann Palliat Med*. 2021;10(8):8919-8929. doi:10.21037/apm-21-1809
19. Shimada H, Suzuki T, Makizako H, et al. Effects of multicomponent exercise on cognitive function in older adults with amnestic mild cognitive impairment: a

randomized controlled trial. *Alzheimer's & Dementia*. 2012;8(4S_Part_4). doi:10.1016/j.jalz.2012.05.386
20. Greblo Jurakic Z, Krizanic V, Sarabon N, Markovic G. Effects of feedback-based balance and core resistance training vs. Pilates training on cognitive functions in older women with mild cognitive impairment: a pilot randomized controlled trial. *Aging Clin Exp Res*. 2017;29(6):1295-1298. doi:10.1007/s40520-017-0740-9
21. Kim J, Yim J. Effects of an exercise protocol for improving handgrip strength and walking speed on cognitive function in patients with chronic stroke. *Medical Science Monitor*. 2017;23:5402-5409. doi:10.12659/MSM.904723
22. Avenali M, Picascia M, Tassorelli C, Sinforiani E, Bernini S. Evaluation of the efficacy of physical therapy on cognitive decline at 6-month follow-up in Parkinson disease patients with mild cognitive impairment: a randomized controlled trial. *Aging Clin Exp Res*. 2021;33(12):3275-3284. doi:10.1007/s40520-021-01865-4
23. Bademli K, Lok N, Canbaz M, Lok S. Effects of Physical Activity Program on cognitive function and sleep quality in elderly with mild cognitive impairment: A randomized controlled trial. *Perspect Psychiatr Care*. 2019;55(3):401-408. doi:10.1111/ppc.12324
24. Lok N, Tosun AS, Lok S, Temel V, Aydın Z. Effect of physical activity program applied to patients with Alzheimer's disease on cognitive functions and depression level: a randomised controlled study. *Psychogeriatrics*. 2023;23(5):856-863. doi:10.1111/psyg.13010
25. De Sá CA, Saretto CB, Cardoso AM, Remor A, Breda CO, da Silva Corralo V. Effects of a physical exercise or motor activity protocol on cognitive function, lipid profile, and BDNF levels in older adults with mild cognitive impairment. *Mol Cell Biochem*. 2024;479(3):499-509. doi:10.1007/s11010-023-04733-z
26. Padala KP, Padala PR, Lensing SY, et al. Home-Based Exercise Program Improves Balance and Fear of Falling in Community-Dwelling Older Adults with Mild Alzheimer's Disease: A Pilot Study. *Journal of Alzheimer's Disease*. 2017;59(2):565-574. doi:10.3233/JAD-170120
27. Langoni CDS, Resende TDL, Barcellos AB, et al. Effect of Exercise on Cognition, Conditioning, Muscle Endurance, and Balance in Older Adults with Mild Cognitive Impairment: A Randomized Controlled Trial. *Journal of Geriatric Physical Therapy*. 2019;42(2):E15-E22. doi:10.1519/JPT.0000000000000191
28. Zhang Q, Zhu M, Huang L, et al. A Study on the Effect of Traditional Chinese Exercise Combined With Rhythm Training on the Intervention of Older Adults With Mild Cognitive Impairment. *Am J Alzheimers Dis Other Demen*. 2023;38(48):1-12. doi:10.1177/15333175231190626
29. Vreugdenhil A, Cannell J, Davies A, Razay G. A community-based exercise programme to improve functional ability in people with Alzheimer's disease: A randomized controlled trial. *Scand J Caring Sci*. 2012;26(1):12-19. doi:10.1111/j.1471-6712.2011.00895.x
30. Papamichail P, Sagredaki ML, Bouzineki C, Kanellopoulou S, Lyros E, Christakou A. The Effectiveness of an Exercise Program on Muscle Strength and Range of Motion on Upper Limbs, Functional Ability and Depression at Early Stage of Dementia. *J Clin Med*. 2024;13(14):1-10. doi:10.3390/jcm13144136
31. Suttanon P, Hill KD, Said CM, et al. Feasibility, safety and preliminary evidence of the effectiveness of a home-based exercise programme for older people with Alzheimer's disease: A pilot randomized controlled trial. *Clin Rehabil*. 2013;27(5):427-438. doi:10.1177/0269215512460877
32. Sanders LMJ, Hortobágyi T, Karssemeijer EGA, Van Der Zee EA, Scherder EJA, Van Heuvelen MJG. Effects of low- And high-intensity physical exercise on physical and cognitive function in older persons with dementia: A randomized controlled trial. *Alzheimers Res Ther*. 2020;12(1):1-15. doi:10.1186/s13195-020-00597-3
33. Dawson N, Judge KS, Gerhart H. Improved Functional Performance in Individuals with Dementia after a Moderate-Intensity Home-Based Exercise Program: A Randomized Controlled Trial. *Journal of Geriatric Physical Therapy*. 2019;42(1):18-27. doi:10.1519/JPT.0000000000000128
34. Santana-Sosa E, Barriopedro MI, López-Mojares LM, Pérez M, Lucia A. Exercise training is beneficial for Alzheimer's patients. *Int J Sports Med*. 2008;29(10):845-850. doi:10.1055/s-2008-1038432
35. Kovas et al. Effects of a multimodal exercise program on balance, functional mobility and fall risk in older adults with cognitive impairment: a randomized controlled single-blind study. 2011;47(3):381-390.
36. de Oliveira Silva F, Ferreira JV, Plácido J, et al. Three months of multimodal training contributes to mobility and executive function in elderly individuals with mild cognitive impairment, but not in those with Alzheimer's disease: A randomized controlled trial. *Maturitas*. 2019;126(April):28-33. doi:10.1016/j.maturitas.2019.04.217
37. Levinger P, Goh AMY, Dunn J, et al. Exercise interveNtion outdoor proJect in the cOmmunitY – results from the ENJOY program for independence in dementia: a feasibility pilot randomised controlled trial. *BMC Geriatr*. 2023;23(1):1-16. doi:10.1186/s12877-023-04132-5

38. Ghahfarrokhi MM, Shirvani H, Rahimi M, Bazgir B, Shamsadini A, Sobhani V. Feasibility and preliminary efficacy of different intensities of functional training in elderly type 2 diabetes patients with cognitive impairment: a pilot randomised controlled trial. *BMC Geriatr*. 2024;24(1):1-15. doi:10.1186/s12877-024-04698-8
39. Fonte C, Smania N, Pedrinolla A, et al. Comparison between physical and cognitive treatment in patients with MCI and Alzheimer's disease. *Aging*. 2019;11(10):3138-3155. doi:10.18632/aging.101970
40. Gebhard D, Mess F. Feasibility and Effectiveness of a Biography-Based Physical Activity Intervention in Institutionalized People With Dementia: Quantitative and Qualitative Results From a Randomized Controlled Trial. *J Aging Phys Act*. 2022;30(2):237-251. doi:10.1123/japa.2020-0343
41. Akbuga Koc E, Yazici-Mutlu Ç, Cinar N, Sahiner T. Comparison of the effect of online physical exercise and computerized cognitive stimulation in patients with Alzheimer's disease during the Covid-19 pandemic. *Complement Ther Clin Pract*. 2024;57(May):10-20. doi:10.1016/j.ctcp.2024.101881
42. Shaw I, Cronje M, Shaw BS. Group-based exercise as a therapeutic strategy for the improvement of mental outcomes in mild to moderate alzheimer's patients in low resource care facilities. *Asian J Sports Med*. 2021;12(1):1-6. doi:10.5812/asjsm.106593
43. Cezar NO de C, Ansai JH, Oliveira MPB de, et al. Feasibility of improving strength and functioning and decreasing the risk of falls in older adults with Alzheimer's dementia: a randomized controlled home-based exercise trial. *Arch Gerontol Geriatr*. 2021;96(March). doi:10.1016/j.archger.2021.104476
44. Mollinedo Cardalda I, López A, Cancela Carral JM. The effects of different types of physical exercise on physical and cognitive function in frail institutionalized older adults with mild to moderate cognitive impairment. A randomized controlled trial. *Arch Gerontol Geriatr*. 2019;83(May):223-230. doi:10.1016/j.archger.2019.05.003
45. Kim MJ, Han CW, Min KY, et al. Physical Exercise with Multicomponent Cognitive Intervention for Older Adults with Alzheimer's Disease: A 6-Month Randomized Controlled Trial. *Dement Geriatr Cogn Dis Extra*. 2016;6(2):222-232. doi:10.1159/000446508
46. Henskens M, Nauta IM, Van Eekeren MCA, Scherder EJA. Effects of Physical Activity in Nursing Home Residents with Dementia: A Randomized Controlled Trial. *Dement Geriatr Cogn Disord*. 2018;46(1-2):60-80. doi:10.1159/000491818
47. Kemoun G, Thibaud M, Roumagne N, et al. Effects of a physical training programme on cognitive function and walking efficiency in elderly persons with dementia. *Dement Geriatr Cogn Disord*. 2010;29(2):109-114. doi:10.1159/000272435
48. de Souto Barreto P, Cesari M, Denormandie P, Armaingaud D, Vellas B, Rolland Y. Exercise or Social Intervention for Nursing Home Residents with Dementia: A Pilot Randomized, Controlled Trial. *J Am Geriatr Soc*. 2017;65(9):E123-E129. doi:10.1111/jgs.14947
49. Roach KE, Tappen RM, Kirk-Sanchez N, Williams CL, Loewenstein D. A randomized controlled trial of an activity specific exercise program for individuals with alzheimer disease in long-term care settings. *Journal of Geriatric Physical Therapy*. 2011;34(2):50-56. doi:10.1519/JPT.0b013e31820aab9c
50. Stevens J, Killeen M. A randomised controlled trial testing the impact of exercise on cognitive symptoms and disability of residents with dementia. *Contemporary nurse : a journal for the Australian nursing profession*. 2006;21(1):32-40. doi:10.5172/conu.2006.21.1.32
51. Brett L, Stapley P, Meedya S, Traynor V. Effect of physical exercise on physical performance and fall incidents of individuals living with dementia in nursing homes: a randomized controlled trial. *Physiother Theory Pract*. 2021;37(1):38-51. doi:10.1080/09593985.2019.1594470
52. Almeida S, Paixão C, da Silva MG, Marques A. Lifestyle-integrated functional exercise for people with Dementia: A pilot study. *J Aging Phys Act*. 2021;29(5):771-780. doi:10.1123/JAPA.2020-0349
53. Yan Y, Xu Y, Wang X, et al. The effect of multi-component exercise intervention in older people with Parkinson's disease and mild cognitive impairment: A randomized controlled study. *Geriatr Nurs (Minneap)*. 2024;60:137-145. doi:10.1016/j.gerinurse.2024.08.028
54. David S, Costa AS, Hohenfeld C, et al. Modulating effects of fitness and physical activity on Alzheimer's disease: Implications from a six-month randomized controlled sports intervention. *Journal of Alzheimer's Disease*. 2025;103(2):552-569. doi:10.1177/13872877241303764
55. Shokri G, Mohammadian F, Noroozian M, Amani-Shalamzari S, Suzuki K. Effects of remote combine exercise-music training on physical and cognitive performance in patients with Alzheimer's disease: a randomized controlled trial. *Front Aging Neurosci*. 2023;15(January):1-9. doi:10.3389/fnagi.2023.1283927
56. Yang JG, Thapa N, Park HJ, et al. Virtual Reality and Exercise Training Enhance Brain, Cognitive, and Physical Health in Older Adults with Mild Cognitive Impairment.

Int J Environ Res Public Health. 2022;19(20). doi:10.3390/ijerph192013300
57. Uemura K, Doi T, Shimada H, et al. Effects of Exercise Intervention on Vascular Risk Factors in Older Adults with Mild Cognitive Impairment: A Randomized Controlled Trial. *Dement Geriatr Cogn Dis Extra*. 2012;2(1):445-455. doi:10.1159/000343486
58. Li PWC, Yu DSF, Siu PM, Wong SCK, Chan BS. Peer-supported exercise intervention for persons with mild cognitive impairment: A waitlist randomised controlled trial (the BRAin Vitality Enhancement trial). *Age Ageing*. 2022;51(10):1-10. doi:10.1093/ageing/afac213
59. Mak A, Delbaere K, Refshauge K, et al. Sunbeam Program Reduces Rate of Falls in Long-Term Care Residents With Mild to Moderate Cognitive Impairment or Dementia: Subgroup Analysis of a Cluster Randomized Controlled Trial. *J Am Med Dir Assoc*. 2022;23(5):743-749.e1. doi:10.1016/j.jamda.2022.01.064
60. Sobol NA, Hoffmann K, Frederiksen KS, et al. Effect of aerobic exercise on physical performance in patients with Alzheimer's disease. *Alzheimer's and Dementia*. 2016;12(12):1207-1215. doi:10.1016/j.jalz.2016.05.004
61. Papatsimpas V, Vrouva S, Papathanasiou G, et al. Does Therapeutic Exercise Support Improvement in Cognitive Function and Instrumental Activities of Daily Living in Patients with Mild Alzheimer's Disease? A Randomized Controlled Trial. *Brain Sci*. 2023;13(7). doi:10.3390/brainsci13071112
62. Ullrich P, Werner C, Schönstein A, et al. Effects of a Home-Based Physical Training and Activity Promotion Program in Community-Dwelling Older Persons with Cognitive Impairment after Discharge from Rehabilitation: A Randomized Controlled Trial. *Journals of Gerontology - Series A Biological Sciences and Medical Sciences*. 2022;77(12):2435-2444. doi:10.1093/gerona/glac005
63. Rivas-Campo Y, Aibar-Almazán A, Afanador-Restrepo DF, et al. Effects of High-Intensity Functional Training (HIFT) on the Functional Capacity, Frailty, and Physical Condition of Older Adults with Mild Cognitive Impairment: A Blind Randomized Controlled Clinical Trial. *Life*. 2023;13(5):1-16. doi:10.3390/life13051224
64. Prick AE, De Lange J, Scherder E, Twisk J, Pot AM. The effects of a multicomponent dyadic intervention with physical exercise on the cognitive functioning of people with dementia: A randomized controlled trial. *J Aging Phys Act*. 2017;25(4):539-552. doi:10.1123/japa.2016-0038
65. Hauer K, Schwenk M, Zieschang T, Essig M, Becker C, Oster P. Physical training improves motor performance in people with dementia: A randomized controlled trial. *J Am Geriatr Soc*. 2012;60(1):8-15. doi:10.1111/j.1532-5415.2011.03778.x
66. Lamb SE, Sheehan B, Atherton N, et al. Dementia And Physical Activity (DAPA) trial of moderate to high intensity exercise training for people with dementia: Randomised controlled trial. *BMJ (Online)*. 2018;361. doi:10.1136/bmj.k1675
67. Casas-Herrero Á, Sáez de Asteasu ML, Antón-Rodrigo I, et al. Effects of Vivifrail multicomponent intervention on functional capacity: a multicentre, randomized controlled trial. *J Cachexia Sarcopenia Muscle*. 2022;13(2):884-893. doi:10.1002/jcsm.12925
68. Bo W, Lei M, Tao S, et al. Effects of combined intervention of physical exercise and cognitive training on cognitive function in stroke survivors with vascular cognitive impairment: a randomized controlled trial. *Clin Rehabil*. 2019;33(1):54-63. doi:10.1177/0269215518791007
69. Bossers WJR, Van Der Woude LHV, Boersma F, Hortobágyi T, Scherder EJA, Van Heuvelen MJG. A 9-Week Aerobic and Strength Training Program Improves Cognitive and Motor Function in Patients with Dementia: A Randomized, Controlled Trial. *American Journal of Geriatric Psychiatry*. 2015;23(11):1106-1116. doi:10.1016/j.jagp.2014.12.191
70. Telenius EW, Engedal K, Bergland A. Long-term effects of a 12 weeks high-intensity functional exercise program on physical function and mental health in nursing home residents with dementia: A single blinded randomized controlled trial Physical functioning, physical health and activity. *BMC Geriatr*. 2015;15(1):1-11. doi:10.1186/s12877-015-0151-8
71. Toots A, Littbrand H, Boström G, et al. Effects of exercise on cognitive function in older people with dementia: A randomized controlled trial. *Journal of Alzheimer's Disease*. 2017;60(1):323-332. doi:10.3233/JAD-170014
72. Rolland Y, Pillard F, Klapouszczak A, et al. Exercise program for nursing home residents with Alzheimer's disease: A 1-year randomized, controlled trial. *J Am Geriatr Soc*. 2007;55(2):158-165. doi:10.1111/j.1532-5415.2007.01035.x
73. Verdelho A, Correia M, Gonçalves-Pereira M, et al. Physical Activity in Mild Vascular Cognitive Impairment: Results of the AFIVASC Randomized Controlled Trial

at 6 Months. *Journal of Alzheimer's Disease*. 2024;101(4):1379-1392. doi:10.3233/JAD-240246
