# Supplementary material for: Dance versus other exercise modalities in mild cognitive impairment and dementia: comparative efficacy from a systematic review and bayesian network meta-analysis
Source: Front Physiol. 2026 Mar 25;17:1782774. doi: 10.3389/fphys.2026.1782774 (PMC13056856; doi:10.3389/fphys.2026.1782774)
Supplement: Supplementary file 7 [file Table3.pdf]

Supplementary Table 3. Summary of Outcomes of the Studies Included in Analysis

MMSE	Mini-Mental State Examination														
	**Dance**														
	1.Bisbe et al. 2020[1] IG: 27.47 ± 2.15 CG: 27.57 ± 2.03	2.Qi et al. 2018[2] IG: 28.2 ± 1.0 CG: 27.3 ± 1.7	3.Lazarou et al. 2017[3] IG: 28.00 ± 2.39 CG: 25.65 ± 3.27	4.Winckel et al. 2004[4] IG: 15.53 ± 4.44 CG: 11.00 ± 4.30											
	**Exergaming**														
	5.Padala et al. 2012[5] IG: 22.4 ± 2.8 CG: 25.5 ± 4.1	6.Santen et al. 2020[6] IG: 17.9 ± 7.3 CG: 17.0 ± 5.9	7.Zheng et al. 2022[7] IG: 16.78 ± 7.18 CG: 13.9 ± 7.98												
	**Chinese Traditional Exercise (CTE)**														
	8.Lam et al. 2012[8,9] IG: 25.4 ± 3.3 CG: 24.2 ± 3.4	9.Jiayuan et al. 2022[10] IG[1] (Mindfulness training): 25.4 ± 2.3 IG[2] (Tai Chi): 24.7 ± 1.6 IG[3] (Mindfulness + Tai Chi): 26.1 ± 1.5	10.Huang et al. 2019[11] IG: 21.17 ± 5.47 CG: 19.47 ± 5.73	11.Chan et al. 2016[12] IG: 24.9 ± 3.9 CG: 24.4 ± 2.6	12.Li et al. 2022[13] IG: 27.4 ± 2.07 CG: 23.83 ± 2.11										
	**Aerobic exercise (AE)**														
	13.Tomoto et al. 2021[14] IG: 28.6 ± 1.3 CG: 28.5 ± 1.8	14.Tsai et al. 2019[15] IG[1] (AE): 27.26 ± 1.10 IG[2] (RE): 26.61 ± 1.20 CG: 26.89 ± 1.41	15.Wei and Ji 2014[16] IG: 25.53 ± 0.82 CG: 24.67 ± 1.42	16.Nakatsuka et al. 2015[17] IG[1] (AE): 24.2 ± 2.2 IG[2] (Cognitive training): 24.5 ± 3.4 CG: 25.4 ± 2.5	17.Kohanpour et al. 2017[18] IG[1] (AE): 24.40 ± 1.42 IG[2] (AE + Lavender): 24.90 ± 0.87 IG[3] (Lavender): 24.30 ± 1.05 CG: 24.20 ± 0.63	18.Liu et al. 2020[19] IG: 25.87 ± 3.36 CG: 24.2 ± 4.87	19.Yang et al. 2015[20] IG: 22.83 ± 2.75 CG: 19.54 ± 3.43	20.Khattak et al. 2022[21] IG: 21.69 ± 1.10 CG: 21.03 ± 1.18	21.Varela et al. 2011[22] IG[1] (40% of HR_{max} AE): 20.66 ± 7.39 IG[2] (60% of HR_{max} AE): 21.06 ± 5.40	22.Varela et al. 2011[22] IG[1] (40% of HR_{max} AE): 20.66 ± 7.39 IG[2] (60% of HR_{max} AE): 21.06 ± 5.40	23.Miu et al. 2008[23] IG: 18.47 ± 5.27 CG: 19.65 ± 3.97	24.Angiolillo et al. 2023[24] IG: 20.37 ± 3.60 CG: 15.31 ± 5.79	25.Phoemsapthawee et al. 2016[25] IG: 20.9 ± 5.1 CG: 19.1 ± 4.8	26.Guzel et al. 2024[26] IG[1] (AE): 12.80 ± 2.52 IG[2] (ME): 17.00 ± 3.43 CG (Balance training): 18.20 ± 9.37	27.Venturelli et al. 2011[27] IG: 12 ± 2 CG: 6 ± 2

									CG: 19.53 ± 5.50	CG: 19.53 ± 5.50							
	**Resistance Exercise (RE)**																
	30.Yoon et al. 2017[28]<br>IG^1 (High speed): 25.36 ± 1.78<br>IG^2 (Low speed): 24.56 ± 3.21<br>CG: 21.14 ± 1.57	31.Yoon et al. 2017[28]<br>IG^1 (High speed): 25.36 ± 1.78<br>IG^2 (Low speed): 24.56 ± 3.21<br>CG: 21.14 ± 1.57	32.Venturelli et al. 2010[29]<br>IG: 23.0 ± 1.4<br>CG: 17.5 ± 2.1	33.Holthoff et al. 2015[30]<br>IG: 21.99 ± 0.54<br>CG: 21.28 ± 0.54	34.Baek et al. 2024[31]<br>IG: 17.68 ± 2.10<br>CG: 18.27 ± 2.12												
	**Multicomponent Exercise (ME)**																
	37.Yang et al. 2022[32]<br>IG^1 (ME): 27.8 ± 1.6<br>IG^2 (Cognitive training): 28.1 ± 1.7<br>CG: 26.7 ± 1.9	38.Li et al. 2021[33]<br>IG: 27.79 ± 1.18<br>CG: 25.42 ± 2.28	39.Avenali et al. 2021[34]<br>IG: 25.29 ± 2.04<br>CG: 24.26 ± 2.57	40.Mak et al. 2022[35]<br>IG: 23.4 ± 4.8<br>CG: 23.3 ± 4.7	41.Sobol et al. 2016[36–38]<br>IG: 23.9 ± 3.4<br>CG: 23.9 ± 3.9	42.Lok et al. 2023[39]<br>IG: 25.50 ± 0.73<br>CG: 23.27 ± 0.45	43.De Sa et al. 2024[40]<br>IG^1 (ME): 24.11 ± 0.91<br>CG (Motor task): 24.89 ± 0.91<br>IG^3 (ME + Motor task): 26.14 ± 1.03	44.Langoni et al. 2019[41,42]<br>IG: 25.0 ± 4.7<br>CG: 20.4 ± 4.1	45.Zhang et al. 2023[43]<br>IG^1 (ME): 25.78 ± 1.89<br>IG^2 (AE): 23.56 ± 5.36<br>CG: 22.71 ± 3.23	46.Sanders et al. 2020[44]<br>IG: 20.4 ± 4.77<br>CG: 18.8 ± 5.88	47.Ghahfarrokhi et al. 2024[45]<br>IG^1 (High intensity): 20.45 ± 2.16<br>IG^2 (Low intensity): 20.83 ± 2.47<br>CG: 18.63 ± 2.81	48.Ghahfarrokhi et al. 2024[45]<br>IG^1 (High intensity): 20.45 ± 2.16<br>IG^2 (Low intensity): 20.83 ± 2.47<br>CG: 18.63 ± 2.81	49.Shaw et al. 2021[46]<br>IG: 20.36 ± 4.05<br>CG: 18.3 ± 6.26	50.Cardalda et al. 2019[47]<br>IG^1 (RE): 19.32 ± 7.10<br>IG^2 (ME): 20.68 ± 7.68<br>CG: 15.44 ± 7.55-14.5	51.Bossers et al. 2015[48,49]<br>IG^1 (ME): 17.16 ± 4.33<br>IG^2 (AE): 15.50 ± 5.51<br>CG: 15.17 ± 4.48	52.Telenius et al. 2015[50,51]<br>IG: 15.5 ± 5.5<br>CG: 15.2 ± 5.4	53.Henskens et al. 2018[52]<br>IG^1 (ADL + ME)): 13.8 ± 6.5<br>IG^2 (ADL): 12.3 ± 5.4<br>IG^3 (ME): 11.6 ± 6.5<br>CG: 9.4 ± 5.8

**MoCA**	**Montreal Cognitive Assessment**						
	**Dance**						
	1. Esmail et al. 2020[53]<br>IG^1 (Dance): 26.67 ± 1.72<br>IG^2 (AE): 27 ± 1.81<br>CG: 25.5 ± 2.53	2.Qi et al. 2018[2]<br>IG: 24.3 ± 2.2<br>CG: 23.7 ± 2.0	3.Lazarou et al. 2017[3]<br>IG: 26.00 ± 2.94<br>CG: 19.82 ± 5.18	4.Franco et al. 2020[54]<br>IG: 23.6 ± 3.2<br>CG: 22.2 ± 4.9	5.Zhu et al. 2018[55]<br>IG: 24.7 ± 2.2<br>CG: 23.6 ± 1.8	6.Song et al. 2024[56]<br>IG: 23.50 ± 2.00<br>CG: 21.77 ± 2.27	7.Chang et al. 2021[57,58]<br>IG: 22.34 ± 1.87<br>CG: 21.21 ± 2.13
	**Exergaming**						
	8.Schwenk et al. 2016[59]<br>IG: 23.7 ± 3.9<br>CG: 25.3 ± 1.9	9.Liu et al. 2022[60]<br>IG^1 (Exergaming Tai Chi): 24.5 ± 0.38<br>IG^2 (Traditional Tai Chi): 23.4 ± 0.38<br>CG: 22.7 ± 0.39	10.Liao et al. 2021[61]<br>IG: 20.7 ± 5.1<br>CG: 20.3 ± 6.3	11.Swinnen et al. 2021[62]<br>IG: 12.1 ± 5.2<br>CG: 5.7 ± 4.0			
	**Yoga**						

	12.Khanthong et al. 2021[63]<br>IG: 22.09 ± 3.47<br>CG: 19.33 ± 2.77	13.Kashyap et al. 2022[64]<br>IG: 24.60 ± 3.08<br>CG: 22.94 ± 3.08						
	**Chinese Traditional Exercise (CTE)**							
	14.Li et al. 2022[65]<br>IG^{1} (Cognitive enhanced Tai Chi): 27.39 ± 1.37<br>IG^{2} (Standard Tai Chi): 26.82 ± 1.84<br>CG: 25.54 ± 1.89	15.Chen et al. 2023[66]<br>IG^{1} (Tai Chi): 23.99 ± 3.10<br>IG^{2} (AE): 23.55 ± 3.34<br>CG: 22.54 ± 3.29	16.Yu et al. 2022[67]<br>IG^{1} (Tai Chi): 26.6 ± 1.9<br>IG^{2} (ME): 25.0 ± 2.5<br>CG: 18.9 ± 5.2	17.Huang et al. 2019[11]<br>IG: 14.83 ± 5.71<br>CG: 12.16 ± 4.72	18.Zheng et al. 2020[68]<br>IG: 24.83 ± 0.52<br>CG: 22.29 ± 0.54	19.Zheng et al. 2021[69–73]<br>IG^{1} (Baduanjin): 24.30 ± 1.78<br>IG^{2} (AE): 22.45 ± 2.40<br>CG: 21.08 ± 3.65	20.Li et al. 2022[13]<br>IG: 24.48 ± 2.33<br>CG: 22.79 ± 2.17	
	**Aerobic Exercise (AE)**							
	21.Song et al. 2019[74]<br>IG: 23.66 ± 1.92<br>CG: 21.40 ± 2.77	22.Rojasavastera et al. 2020[75]<br>IG^{1} (Action observation with gait training): 24.64 ± 2.34<br>IG^{2} (Gait training): 24.00 ± 2.86<br>CG: 24.45 ± 2.91	23.Karthikeyan T 2020[76]<br>IG: 21.60 ± 1.68<br>CG: 20.20 ± 0.94	24.Krootnark et al. 2024[77]<br>IG^{1} (AE): 25.40 ± 2.54<br>IG^{2} (RE): 24.53 ± 2.90<br>CG: 21.17 ± 2.77	25.Liu et al. 2020[19]<br>IG: 21.74 ± 4.55<br>CG: 20.76 ± 5.39	26.Choi et al. 2018[78,79]<br>IG: 25.13 ± 2.78<br>CG: 21.46 ± 3.11	27.Khattak et al. 2022[21]<br>IG: 24.07 ± 1.60<br>CG: 21.07 ± 1.23	28.Amjad et al. 2018[80]<br>IG: 22.88 ± 1.65<br>CG: 20.94 ± 3.27
	**Resistance Exercise (RE)**							
	29.Wang et al. 2020[81]<br>IG: 21.72 ± 2.19<br>CG: 20.13 ± 2.43	30.Yoon et al. 2017[28]<br>IG^{1} (High speed): 24.29 ± 2.58<br>IG^{2} (Low speed): 18.33 ± 5.29<br>CG: 18.14 ± 2.97	31.Yoon et al. 2017[28]<br>IG^{1} (High speed): 24.29 ± 2.58<br>IG^{2} (Low speed): 18.33 ± 5.29<br>CG: 18.14 ± 2.97	32.Hong et al. 2018[82]<br>IG: 21.70 ± 3.05<br>CG: 20.50 ± 5.05				
	**Multicomponent Exercise (ME)**							
	33.Li et al. 2021[33]<br>IG: 25.19 ± 1.29<br>CG: 19.45 ± 2.00	34.Jurakic et al. 2017[83]<br>CG: 25.79 ± 1.53<br>IG: 24.29 ± 1.98	35.Kim and Yim 2017[84]<br>IG: 23.42 ± 6.85<br>CG: 22.26 ± 4.97	36.Avenali et al. 2021[34]<br>IG: 20.53 ± 3.54<br>CG: 17.22 ± 2.48	37.Zhang et al. 2023[43]<br>IG^{1} (ME): 23.28 ± 3.69<br>IG^{2} (AE): 20.89 ± 5.35<br>CG: 19.59 ± 4.43	38.Rivas-Campo et al. 2023[85,86]<br>IG: 22.58 ± 1.41<br>CG: 21.68 ± 1.27	39.Levinger et al. 2023[87]<br>IG: 12.3 ± 4.2<br>CG: 8.8 ± 6.1	40.Koc et al. 2024[88]<br>IG^{1} (ME): 16.10 ± 3.44<br>IG^{2} (ME + cognitive stimulation): 17.30 ± 3.19<br>CG: 16.10 ± 3.22

TMT-A	Trail Making Test-A			
	**Dance**			
	1.Bisbe et al. 2020[1]<br>IG: 76.29 ± 42.07<br>CG: 80.64 ± 38.07	2.Qi et al. 2018[2]<br>IG: 71.0 ± 29.3<br>CG: 68.8 ± 19.1	3.Zhu et al. 2018[55]<br>IG: 66 ± 25<br>CG: 69 ± 20	4.Ho et al. 2018[89]<br>IG^{1} (DMT): 131.31 ± 92.34<br>IG^{2} (AE): 118.90 ± 85.40<br>CG: 120.03 ± 98.31
	**Exergaming**			
	5.Schwenk et al. 2016[59]<br>IG: 46.0 ± 14.1<br>CG: 45.1 ± 21.0	6.Liu et al. 2022[60]<br>IG^{1} (Exergaming Tai Chi): 65.8 ± 1.9<br>IG^{2} (Traditional Tai Chi): 71.7 ± 1.9<br>CG: 80.3 ± 1.9	7.Santen et al. 2020[6]<br>IG: 140.2 ± 82.8<br>CG: 136.8 ± 79.4	
	**Yoga**			
	8.Khanthong et al. 2021[63]<br>IG: 58.20 ± 21.27<br>CG: 62.95 ± 25.78			
	**Chinese Traditional Exercise (CTE)**			
	9.Su et al. 2021[90]	10.Zheng et al. 2020[68]		

	IG: 43.49 ± 12.56	IG: 85.81 ± 9.70					
	CG: 50.20 ± 12.64	CG: 128.22 ± 12.97					
	**Aerobic Exercise (AE)**						
	11.Nakatsuka et al. 2015[17]	12.Krootnark et al. 2024[77]	13.Khattak et al. 2022[21]	14.Amjad et al. 2018[80]			
	IG^1 (AE): 86.3 ± 38.7	IG^1 (AE): 39.53 ± 10.70	IG: 72.6 ± 19.2	IG: 84.6 ± 39			
	IG^2 (Cognitive training): 85.8 ± 39.1	IG^2 (RE): 45.83 ± 22.92	CG: 102 ± 25.2	CG: 132 ± 51.6			
	CG: 71 ± 29.2	CG: 65.68 ± 36.37					
	**Resistance Exercise (RE)**						
	15.Fernandez-Gonzalo et al. 2016[91]	16.Yoon et al. 2018[92]					
	IG: 65.9 ± 35.1	IG: 48.26 ± 27.33					
	CG: 67.2 ± 38.8	CG: 42.59 ± 15.92					
	**Multicomponent Exercise (ME)**						
	17.Yang et al. 2022[32]	18.Kim and Yim 2017[84]	19.Avenali et al. 2021[34]	20.Rivas-Campo et al. 2023[85,86]	21.Sanders et al. 2020[44]	22.Fonte et al. 2019[93]	23.Bossers et al. 2015[48,49]
	IG^1 (ME): 42.4 ± 20.1	IG: 66.38 ± 80.33	IG: 155.80 ± 73.82	IG: 85.86 ± 10.72	IG: 126 ± 65.3	MCI	IG^1 (ME): 161.85 ± 38.52
	IG^2 (Cognitive training): 36.4 ± 18.1	CG: 35.37 ± 30.05	CG: 129.35 ± 39.24	CG: 93.99 ± 10.37	CG: 153 ± 56.6	IG^1 (ME): 82 ± 12.2	IG^2 (AE): 179.60 ± 127.88
	CG: 46.1 ± 22.1					IG^2 (Cognitive training): 97 ± 47.1	CG: 185.83 ± 117.03
						CG: 149.1 ± 68.8	

**TMT-B**	**Trail Making Test-B**			
	**Dance**			
	1.Bisbe et al. 2020[1]	2.Qi et al. 2018[2]	3.Zhu et al. 2018[55]	4.Ho et al. 2018[89]
	IG: 223.47 ± 98.57	IG: 161.6 ± 53.8	IG: 158 ± 49	IG^1 (DMT): 200.66 ± 97.09
	CG: 230.50 ± 137.40	CG: 181.6 ± 46.7	CG: 177 ± 48	IG^2 (AE): 198.23 ± 100.42
				CG: 189.89 ± 107.94
	**Exergaming**			
	5.Schwenk et al. 2016[59]	6.Liu et al. 2022[60]	7.Liao et al. 2021[61]	8.Santen et al. 2020[6]
	IG: 155.6 ± 101.3	IG^1 (Exergaming Tai Chi): 137.1 ± 8.5	IG: 115.2 ± 69.1	IG: 236.0 ± 59.0
	CG: 99.8 ± 39.5	IG^2 (Traditional Tai Chi): 152.1 ± 8.2	CG: 118.3 ± 51.4	CG: 223.1 ± 70.9
		CG: 192.7 ± 8.2		
	**Yoga**			
	9.Khanthong et al. 2021[63]	10.Eyre et al. 2017[94,95]		
	IG: 133.11 ± 69.60	IG: 96.28 ± 41.25		
	CG: 172.61 ± 83.88	CG: 89.89 ± 34.17		
	**Chinese Traditional Exercise (CTE)**			
	11.Li et al. 2022[65]	12.Chen et al. 2023[66]	13.Su et al. 2021[90]	14.Zheng et al. 2020[68]
	IG^1 (Cognitive enhanced Tai Chi): 69.81 ± 13.91	IG^1 (Tai Chi): 202.25 ± 72.19	IG: 91.06 ± 21.51	IG: 170.11 ± 15.90
	IG^2 (Standard Tai Chi): 84.67 ± 14.67	IG^2 (AE): 206.78 ± 75.11	CG: 102.34 ± 20.42	CG: 241.44 ± 24.76
	CG: 93.39 ± 11.59	CG: 223.86 ± 85.00		
	**Aerobic Exercise (AE)**			
	15.Krootnark et al. 2024[77]	16.Khattak et al. 2022[21]	17.Amjad et al. 2018[80]	
	IG^1 (AE): 78.26 ± 34.08	IG: 178.2 ± 36.6	IG: 168 ± 69.6	
	IG^2 (RE): 88.26 ± 52.83	CG: 271.2 ± 39	CG: 228.6 ± 50.4	
	CG: 154.36 ± 92.64			
	**Resistance Exercise (RE)**			
	18.Fernandez-Gonzalo et al. 2016[91]	19.Lu et al. 2016[96]	20.Yoon et al. 2018[92]	
	IG: 196.6 ± 129.7	IG: 95.45 ± 45.00	IG: 140.82 ± 34.65	
	CG: 186.0 ± 141.9	CG: 117.85 ± 54.01	CG: 187.20 ± 70.14	
	**Multicomponent Exercise (ME)**			

21.Kim and Yim 2017[84]	22.Avenali et al. 2021[34]	23.Rivas-Campo et al. 2023[85,86]	24.Dawson et al. 2019[97]	25.Fonte et al. 2019[93]	26.Bo et al. 2019[98]
IG: 97.23 ± 104.86<br>CG: 71.50 ± 69.90	IG: 290.00 ± 47.70<br>CG: 304.64 ± 122.31	IG: 186.83 ± 24.12<br>CG: 199.49 ± 41.91	IG: 462 ± 335<br>CG: 351 ± 192	MCI<br>IG^1 (ME): 190.1 ± 30.6<br>IG^2 (Cognitive training): 173.1 ± 53.3<br>CG: 297.3 ± 71	IG^1 (ME): 178.56 ± 25.77<br>IG^2 (ME + Cognitive training): 167.34 ± 23.94<br>IG^3 (Cognitive training): 175.91 ± 21.95<br>CG: 183.30 ± 32.54

**TUG**	**Timed Up and Go**						
	**Dance**						
	1.Esmail et al. 2020[53]<br>IG^1 (Dance): 5.82 ± 0.85<br>IG^2 (AE): 5.6 ± 0.67<br>CG: 5.79 ± 0.92	2.Bisbe et al. 2020[1]<br>IG: 8.15 ± 1.37<br>CG: 9.01 ± 1.75	3.Bracco et al. 2023[107]<br>IG: 24.75 ± 6.04<br>CG: 29.36 ± 11.92				
	**Exergaming**						
	4.Padala et al. 2012[5]<br>IG: 13.9 ± 7.9<br>CG: 12.8 ± 3.2	5.Karssemeijer et al. 2019[108,109]<br>IG^1 (Exergaming): 13.0 ± 4.2<br>IG^2 (AE): 15.1 ± 6.6<br>CG: 15.0 ± 7.5					
	**Yoga**						
	6.Khanthong et al. 2021[63]<br>IG: 9.81 ± 1.22<br>CG: 10.58 ± 1.85						
	**Chinese Traditional Exercise**						
	7.Li et al. 2021[110]<br>IG: 11.86 ± 0.56<br>CG: 13.04 ± 0.89	8.Jiayuan et al. 2022[10]<br>IG^1 (Mindfulness training): 10.2 ± 0.7<br>IG^2 (Tai Chi): 9.6 ± 0.6<br>IG^3 (Mindfulness + Tai Chi): 9.1 ± 0.5	9.Liu et al. 2018[111]<br>IG: 14.86 ± 1.03<br>CG: 18.97 ± 1.97	10.Nyman et al. 2019[112,113]<br>IG: 21.1 ± 8.7<br>CG: 19.7 ± 5.3	11.Li et al. 2022[13]<br>IG: 8.64 ± 0.21<br>CG: 9.16 ± 0.15		
	**Aerobic Exercise**						
	12.Donnezan et al. 2018[99]<br>IG^1 (AE): 8.90 ± 1.21<br>IG^2 (Cognitive training): 9.97 ± 3.44<br>IG^3 (AE + Cognitive training): 9.84 ± 1.18	13.Krootnark et al. 2024[77]<br>IG^1 (AE): 11.24 ± 1.79<br>IG^2 (RE): 11.94 ± 3.07<br>CG: 15.38 ± 5.55	14.Dillon and Prapavessis 2021[114]<br>IG: 18.7 ± 5.7<br>CG: 44.0 ± 32.3	15.Choi et al. 2018[78,79]<br>IG: 7.59 ± 1.14<br>CG: 8.38 ± 1.25	16.Varela et al. 2011[22]<br>IG^1 (40% of HR_{max} AE): 18.58 ± 5.55<br>IG^2 (60% of HR_{max} AE): 14.39 ± 5.19	17.Varela et al. 2011[22]<br>IG^1 (40% of HR_{max} AE): 18.58 ± 5.55<br>IG^2 (60% of HR_{max} AE): 14.39 ± 5.19	18.Abbas et al. 2023[115]<br>IG^1 (AE): 13.19 ± 2.65<br>IG^2 (Functional exercise): 14.62 ± 2.18<br>IG^3 (AE +

	CG: 11.58 ± 2.18				CG: 17.65 ± 6.81	CG: 17.65 ± 6.81-	Functional exercise): 13.44 ± 4.17							
	**Resistance Exercise**													
	19.Fernandez-Gonzalo et al. 2016[91] IG: 18.2 ± 13.9 CG: 17.6 ± 14.8	20.Lu et al. 2016[96] CG: 7.29 ± 0.77 IG: 8.10 ± 1.01	21.Yoon et al. 2018[92] IG: 9.26 ± 2.03 CG: 9.89 ± 1.59	22.Yoon et al. 2017[28] IG¹ (High speed): 9.14 ± 1.42 IG² (Low speed): 9.36 ± 0.83 CG: 10.59 ± 1.03	23.Yoon et al. 2017[28] IG¹ (High speed): 9.14 ± 1.42 IG² (Low speed): 9.36 ± 0.83 CG: 10.59 ± 1.03									
	**Multicomponent Exercise**													
	24.Sobol et al. 2016[36–38] IG: 6.52 ± 1.72 CG: 6.61 ± 1.86	25.Ullrich et al. 2022[105] IG: 17.40 ± 6.80 CG: 26.95 ± 17.07	26.Langoni et al. 2019[41,42] IG: 8.3 ± 2 CG: 9.7 ± 1.4	27.Vreugdenhil et al. 2012[106] IG: 9.1 ± 3.8 CG: 12.8 ± 4.1	28.Hauer et al. 2012[116–118] IG: 11.2 ± 4.5 CG: 17.5 ± 17.3	29.Suttanon et al. 2013[119] IG: 16.18 ± 5.61 CG: 16.55 ± 6.22	30.Sanders et al. 2020[44] IG: 14.1 ± 6.62 CG: 18.0 ± 7.20	31.Ghahfarrokhi et al. 2024[45] IG¹ (High intensity): 10.24 ± 4.79 IG² (Low intensity): 10.29 ± 4.56 CG: 11.48 ± 5.01	32.Ghahfarrokhi et al. 2024[45] IG¹ (High intensity): 10.24 ± 4.79 IG² (Low intensity): 10.29 ± 4.56 CG: 11.48 ± 5.01	33.Gebhard and Mess 2022[120] IG: 31.96 ± 20.31 CG: 34.92 ± 19.15	34.Cezar et al. 2021[121,122] IG: 20.2 ± 7.7 CG: 19.5 ± 5.4	35.Bossers et al. 2015[48,49] IG¹ (ME): 20.40 ± 9.20 IG² (AE): 23.84 ± 15.20 CG: 27.73 ± 19.16	36.Henskens et al. 2018[52] IG¹ (ADL + ME)): 44.3 ± 12.7 IG² (ADL): 43.8 ± 21.8 IG³ (ME): 50.0 ± 11.3 CG: 48.2 ± 8.6	37.Koc et al. 2024[88] IG¹ (ME): 17.85 ± 6.00 IG² (ME + cognitive stimulation): 19.55 ± 8.45 CG: 26.90 ± 11.37

**BBS**	**Berg Balance Scale**		
	**Dance**		
	1.Bisbe et al. 2020[1] IG: 53.94 ± 1.92 CG: 52.64 ± 2.13	2.Qi et al. 2018[2] IG: 55.3 ± 0.9 CG: 54.8 ± 1.5	3.Chang et al. 2021[57,58] IG: 37.27 ± 5.40 CG: 35.45 ± 4.72
	**Exergaming**		
	4.Padala et al. 2012[5] IG: 49.6 ± 5.7 CG: 46.6 ± 8.7		
	**Chinese Traditional Exercise**		
	5.Lam et al. 2012[8,9] IG: 53.4 ± 2.3 CG: 52.3 ± 3.4	6.Nyman et al. 2019[112,113] IG: 44.8 ± 5.7 CG: 44.7 ± 7.2	7.Li et al. 2022[13] IG: 52.38 ± 2.19 CG: 47.64 ± 2.21
	**Aerobic Exercise**		
	8.Choi et al. 2018[78,79] IG: 55.43 ± 1.33 CG: 54.86 ± 2.02	9.Abbas et al. 2023[115] IG¹ (AE): 36.85 ± 5.01 IG² (Functional training): 36.85 ± 3.77 IG³ (AE + Functional training): 40.6 ± 7.48	10.Law et al. 2021[123,124] IG¹ (AE): 47.92 ± 1.13 IG² (Functional task): 53.65 ± 4.49 IG³ (Cognitive training): 49.32 ± 8.59 CG: 50.19 ± 7.10
	**Resistance Exercise**		

	11.Fernandez-Gonzalo et al. 2016[91]				
	IG: 45.9 ± 9.1				
	CG: 44.0 ± 9.6				
	**Multicomponent Exercise**				
	12.Langoni et al. 2019[41,42]	13.Dawson et al. 2019[97]	14.Telenius et al. 2015[50,51]	15.Toots et al. 2017[125–129]	16.Koc et al. 2024[88]
	IG: 55.1 ± 1.1	IG: 41.5 ± 2.2	IG: 37.2 ± 14.0	IG: 31.2 ± 15.3	IG^1 (ME): 39.40 ± 11.37
	CG: 53.3 ± 3	CG: 36.6 ± 8.7	CG: 36.6 ± 14.4	CG: 28.2 ± 15.8	IG^2 (ME + cognitive stimulation): 41.05 ± 13.75
					CG: 27.80 ± 14.47

**GDS**	**Geriatric Depression Scale**			
	**Dance**			
	1.Zhu et al. 2018[55]	2.Chang et al. 2021[57,58]		
	IG: 10.4 ± 6.0	IG: 4.31 ± 1.14		
	CG: 11.2 ± 6.0	CG: 5.02 ± 1.67		
	**Chinese Traditional Exercise -Tai Chi**			
	3.Huang et al. 2019[11]	4.Cheng et al. 2014[103,104]		
	IG: 2.44 ± 1.04	IG^1 (Tai Chi): 7.75 ± 2.83		
	CG: 5.37 ± 1.89	IG^2 (Majiang): 5.17 ± 4.57		
		CG: 9.17 ± 2.76		
	**Aerobic Exercise**			
	5.Nakatsuka et al. 2015[17]			
	IG^1 (AE): 3.2 ± 2.5			
	IG^2 (Cognitive training): 5 ± 2.5			
	CG: 4.9 ± 3.7			
	**Resistance Exercise**			
	6.Wang et al. 2020[81]			
	IG: 5.11 ± 2.63			
	CG: 6.74 ± 2.81			
	**Multicomponent Exercise**			
	7.Ullrich et al. 2022[105]	8.Vreugdenhil et al. 2012[106]	9.Levinger et al. 2023[87]	10.Koc et al. 2024[88]
	IG: 5.15 ± 3.44	IG: 2.0 ± 1.5	IG: 4.1 ± 2.9	IG^1 (ME): 7.25 ± 2.57
	CG: 5.41 ± 3.21	CG: 2.3 ± 1.4	CG: 4.5 ± 3.2	IG^2 (ME + Cognitive training): 6.05 ± 2.74
				CG: 7.10 ± 3.64

**DSF**	**Digit Span Forward**		
	**Dance**		
	1.Ho et al. 2018[89]		
	IG^1 (DMT): 6.54 ± 1.52		
	IG^2 (AE): 6.51 ± 1.69		
	CG: 6.90 ± 1.23		
	**Chinese Traditional Exercise**		
	2.Li et al. 2022[65]	3.Lam et al. 2012[8,9]	4.Yu et al. 2022[67]
	IG^1 (Cognitive enhanced Tai Chi): 12.48 ± 1.73	IG: 6.6 ± 1.2	IG^1 (Tai Chi): 8.0 ± 1.1
	IG^2 (Standard Tai Chi): 12.32 ± 1.78	CG: 6.3 ± 1.3	IG^2 (ME): 8.2 ± 0.7
	CG: 11.25 ± 2.05		CG: 6.6 ± 1.0
	**Aerobic Exercise**		
	5.Donnezan et al. 2018[99]	6.Krootnark et al. 2024[77]	7.Eggermont et al. 2009[100]

	IG^1 (AE): 5.94 ± 0.87<br>IG^2 (Cognitive training): 6.18 ± 1.11<br>IG^3 (AE + Cognitive training): 6.15 ± 1.06<br>CG: 5.36 ± 0.84	IG^1 (AE): 7.90 ± 1.27<br>IG^2 (RE): 7.50 ± 1.20<br>CG: 6.53 ± 1.11	IG: 4.63 ± 1.64<br>CG: 4.98 ± 1.57			
	**Resistance Exercise**					
	8.Fernandez-Gonzalo et al. 2016[91]<br>IG: 7.4 ± 2.1<br>CG: 6.7 ± 2.1	9.Lu et al. 2016[96]<br>CG: 7.41 ± 1.44<br>IG: 7.65 ± 1.61	10.Hong et al. 2018[82]<br>IG: 4.0 ± 0.81<br>CG: 4.0 ± 1.34			
	**Multicomponent Exercise**					
	11.Li et al. 2022[101]<br>IG: 8.73 ± 1.34<br>CG: 8.53 ± 1.46	12.Prick et al. 2017[102]<br>IG: 10.92 ± 3.35<br>CG: 10.39 ± 2.86	13.Sanders et al. 2020[44]<br>IG: 6.67 ± 1.80<br>CG: 6.64 ± 1.91	14.Bo et al. 2019[98]<br>IG^1 (ME): 7.64 ± 0.93<br>IG^2 (ME + Cognitive training): 8.52 ± 1.23<br>IG^3 (Cognitive training): 8.18 ± 1.43<br>CG: 7.36 ± 0.90	15.Bossers et al. 2015[48,49]<br>IG^1 (ME): 9.08 ± 2.73<br>IG^2 (AE): 8.83 ± 3.13<br>CG: 8.42 ± 3.06	16.Henskens et al. 2018[52]<br>IG^1 (ADL + ME): 7.9 ± 4.4<br>IG^2 (ADL): 7.9 ± 2.6<br>IG^3 (ME): 8 ± 2.3<br>CG: 9.9 ± 2.8

DSB	Digit Span Backward				
	**Dance**				
	1.Ho et al. 2018[89]<br>IG^1 (DMT): 3.54 ± 1.40<br>IG^2 (AE): 3.48 ± 1.59<br>CG: 3.40 ± 1.47				
	**Chinese Traditional Exercise -Tai Chi**				
	2.Li et al. 2022[65]<br>IG^1 (Cognitive enhanced Tai Chi): 9.87 ± 1.25<br>IG^2 (Standard Tai Chi): 9.32 ± 1.56<br>CG: 8.13 ± 1.98	3.Lam et al. 2012[8,9]<br>IG: 2.4 ± 1.2<br>CG: 2.4 ± 1.1	4.Yu et al. 2022[67]<br>IG^1 (Tai Chi): 5.2 ± 1.2<br>IG^2 (ME): 5.2 ± 1.1<br>CG: 4.3 ± 1.9		
	**Aerobic Exercise**				
	5.Donnezan et al. 2018[99]<br>IG^1 (AE): 4.72 ± 1.18<br>IG^2(Cognitive training): 4.63 ± 1.09<br>IG^3 (AE + Cognitive training): 4.95 ± 1.12<br>CG: 3.79 ± 0.97	6.Krootnark et al. 2024[77]<br>IG^1 (AE): 3.67± 1.09<br>IG^2 (RE): 3.40 ± 0.77<br>CG: 3.00 ± 0.59	7.Eggermont et al. 2009[100]<br>IG: 3.69 ± 1.28<br>CG: 3.89 ± 1.25		
	**Resistance Exercise**				
	8.Fernandez-Gonzalo et al. 2016[91]<br>IG: 4.7 ± 1.6<br>CG: 4.4 ± 1.7	9.Lu et al. 2016[96]<br>CG: 3.82 ± 0.91<br>IG: 4.00 ± 1.13	10.Hong et al. 2018[82]<br>IG: 2.17 ± 1.52<br>CG: 1.08 ± 0.91		
	**Multicomponent Exercise**				
	11.Li et al. 2022[101]<br>IG: 4.32 ± 1.46<br>CG: 3.94 ± 1.22	12.Prick et al. 2017[102]<br>IG: 5.42 ± 2.39<br>CG: 5.58 ± 2.91	13.Sanders et al. 2020[44]<br>IG: 4.01 ± 1.31<br>CG: 4.04 ± 1.60	14.Bossers et al. 2015[48,49]<br>IG^1 (ME): 5.44 ± 2.43<br>IG^2 (AE): 5.05 ± 3.22<br>CG: 3.97 ± 2.56	15.Henskens et al. 2018[52]<br>IG^1 (ADL + ME)): 4.5 ± 1.6<br>IG^2 (ADL): 4.3 ± 2.3<br>IG^3 (ME): 3.4 ± 0.8<br>CG: 5 ± 1.2

SPPB	Short Physical Performance Battery
	**Dance**
	1.Bracco et al. 2023[107]

IG: 4.3 ± 2.2			
CG: 4.1 ± 1.8			
**Exergaming**			
2.Karssemeijer et al. 2019[108,109]	3.Santen et al. 2020[6]	4.Swinnen et al. 2021[62]	
IG^1 (Exergaming): 9.5 ± 1.8	IG: 7.6 ± 2.2	IG: 8.5 ± 2.5	
IG^2 (AE): 9.2 ± 2.6	CG: 8.0 ± 2.2	CG: 3.8 ± 2.5	
CG: 9.2 ± 2.4			
**Chinese Traditional Exercise**			
5.Jiayuan et al. 2022[10]			
IG^1 (Mindfulness training): 9.7 ± 1.1			
IG^2 (Tai Chi): 10.4 ± 1.1			
IG^3 (Mindfulness + Tai Chi): 10.6 ± 1.1			
**Resistance Exercise**			
6.Yoon et al. 2018[92]	7.Yoon et al. 2017[28]	8.Yoon et al. 2017[28]	
IG: 10.85 ± 1.60	IG^1 (High speed): 10.79 ± 1.58	IG^1 (High speed): 10.79 ± 1.58	
CG: 10.91 ± 1.20	IG^2 (Low speed): 10.56 ± 1.59	IG^2 (Low speed): 10.56 ± 1.59	
	CG: 7.57 ± 0.98	CG: 7.57 ± 0.98	
**Multicomponent Exercise**			
9.Mak et al. 2022[35]	10.Ullrich et al. 2022[105]	11.Sanders et al. 2020[44]	12.Gebhard and Mess 2022[120]
IG: 5.4 ± 3.0	IG: 7.48 ± 2.04	IG: 8.96 ± 2.31	IG: 4.50 ± 2.86
CG: 4.0 ± 2.7	CG: 4.86 ± 2.51	CG: 7.61 ± 2.41	CG: 2.50 ± 2.40

QoL-AD	Quality of Life in Alzheimer's Disease		
	**Dance**		
	1.Bracco et al. 2023[107]		
	IG: 37.8 ± 5.3		
	CG: 36.9 ± 5.4		
	**Exergaming**		
	2.Padala et al. 2012[5]	3.Zheng et al. 2022[7]	
	IG: 35.9 ± 2.8	IG: 31.83 ± 3.99	
	CG: 35.6 ± 5.6	CG: 27.25 ± 5.5	
	**Aerobic Exercise**		
	4.Song et al. 2019[74]	5.Yang et al. 2015[20]	6.Angiolillo et al. 2023[24]
	IG: 30.29 ± 3.41	IG: 30.67 ± 4.84	IG: 26.16 ± 9.35
	CG: 28.97 ± 2.72	CG: 30.19 ± 4.53	CG: 28.38 ± 9.36
	**Multicomponent Exercise**		
	7.Lamb et al. 2018[131,132]	8.Levinger et al. 2023[87]	9.Shaw et al. 2021[46]
	IG: 38.4 ± 5.8	IG: 35.5 ± 7.3	IG: 40.93 ± 3.93
	CG: 39.1 ± 5.7	CG: 35.0 ± 7.4	CG: 38.95 ± 5.23

Gait Speed m/s	
	**Dance**
	1.Bracco et al. 2023[107]
	IG: 0.62 ± 0.22
	CG: 0.47 ± 0.14
	**Exergaming**

2.Swinnen et al. 2021[62]	3.Schwenk et al. 2016[59]
IG: 0.8 ± 0.3	IG: 1.34 ± 0.37
CG: 0.5 ± 0.2	CG: 1.43 ± 0.34
**Aerobic Exercise**	
4.Rojasavastera et al. 2020[75]	
IG[1] (Action observation with gait training): 1.02 ± 0.15	
IG[2] (Gait training): 1.03 ± 0.10	
CG: 0.88 ± 0.15	
**Multicomponent Exercise**	
5.Doi et al. 2013[130]	6.Dawson et al. 2019[97]
IG: 1.38 ± 0.32	IG: 0.7 ± 0.1
CG: 1.26 ± 0.21	CG: 0.6 ± 0.3

References:

1. Bisbe M, Fuente-Vidal A, López E, et al. Comparative Cognitive Effects of Choreographed Exercise and Multimodal Physical Therapy in Older Adults with Amnestic Mild Cognitive Impairment: Randomized Clinical Trial. *Journal of Alzheimer's Disease*. 2020;73(2):769-783. doi:10.3233/JAD-190552
2. Qi M, Zhu Y, Zhang L, Wu T, Wang J. The effect of aerobic dance intervention on brain spontaneous activity in older adults with mild cognitive impairment: A resting-state functional MRI study. *Exp Ther Med*. Published online 2018:715-722. doi:10.3892/etm.2018.7006
3. Lazarou I, Parastatidis T, Tsolaki A, et al. International Ballroom Dancing Against Neurodegeneration: A Randomized Controlled Trial in Greek Community-Dwelling Elders With Mild Cognitive impairment. *Am J Alzheimers Dis Other Demen*. 2017;32(8):489-499. doi:10.1177/1533317517725813
4. Van de Winckel A, Feys H, De Weerdt W, Dom R. Cognitive and behavioural effects of music-based exercises in patients with dementia. *Clin Rehabil*. 2004;18(3):253-260. doi:10.1191/0269215504cr750oa
5. Padala KP, Padala PR, Malloy TR, et al. Wii-fit for improving gait and balance in an assisted living facility: A pilot study. *J Aging Res*. 2012;2012:6-11. doi:10.1155/2012/597573
6. van Santen J, Dröes RM, Twisk JWR, Blanson Henkemans OA, van Straten A, Meiland FJM. Effects of Exergaming on Cognitive and Social Functioning of People with Dementia: A Randomized Controlled Trial. *J Am Med Dir Assoc*. 2020;21(12):1958-1967.e5. doi:10.1016/j.jamda.2020.04.018
7. Zheng J, Yu P, Chen X. An Evaluation of the Effects of Active Game Play on Cognition, Quality of Life and Depression for Older People with Dementia. *Clin Gerontol*. 2022;45(4):1034-1043. doi:10.1080/07317115.2021.1980170
8. Lam LCW, Chau RCM, Wong BML, et al. A 1-Year Randomized Controlled Trial Comparing Mind Body Exercise (Tai Chi) With Stretching and Toning Exercise on Cognitive Function in Older Chinese Adults at Risk of Cognitive Decline. *J Am Med Dir Assoc*. 2012;13(6):568.e15-568.e20. doi:10.1016/j.jamda.2012.03.008
9. Lam LCW, Chau RCM, Wong BML, et al. Interim follow-up of a randomized controlled trial comparing Chinese style mind body (Tai Chi) and stretching exercises on cognitive function in subjects at risk of progressive cognitive decline. *Int J Geriatr Psychiatry*. 2011;26(7):733-740. doi:10.1002/gps.2602
10. Jiayuan Z, Xiang-Zi J, Li-Na M, Jin-Wei Y, Xue Y. Effects of Mindfulness-Based Tai Chi Chuan on Physical Performance and Cognitive Function among Cognitive Frailty Older Adults: A Six-Month Follow-Up of a Randomized Controlled Trial. *Journal of Prevention of Alzheimer's Disease*. 2022;9(1):104-112. doi:10.14283/jpad.2021.40
11. Huang N, Li W, Rong X, et al. Effects of a Modified Tai Chi Program on Older People with Mild Dementia: A Randomized Controlled Trial. *Journal of Alzheimer's Disease*. 2019;72(3):947-956. doi:10.3233/JAD-190487
12. Chan AWK, Yu DSF, Choi KC, Lee DTF, Sit JWH, Chan HYL. Tai chi qigong as a means to improve night-time sleep quality among older adults with cognitive impairment: A pilot randomized controlled trial. *Clin Interv Aging*. 2016;11:1277-1286. doi:10.2147/CIA.S111927
13. Li K, Yu H, Kortas JA, Lin X, Lipowski M. The effect of 12 weeks of Baduanjin exercise on cognitive function, lower limb balance and quality of life of the elderly with mild cognitive impairment: a randomized controlled trial. *Gazzetta Medica Italiana Archivio per le Scienze Mediche*. 2022;181(11):811-823. doi:10.23736/S0393-3660.22.04802-1
14. Tomoto T, Liu J, Tseng BY, et al. One-Year Aerobic Exercise Reduced Carotid Arterial Stiffness and Increased Cerebral Blood Flow in Amnestic Mild Cognitive Impairment. *Journal of Alzheimer's Disease*. 2021;80(2):841-853. doi:10.3233/JAD-201456
15. Tsai CL, Pai MC, Ukropec J, Ukropcová B. Distinctive Effects of Aerobic and Resistance Exercise Modes on Neurocognitive and Biochemical Changes in Individuals with Mild Cognitive Impairment. *Curr Alzheimer Res*. 2019;16(4):316-332. doi:10.2174/1567205016666190228125429
16. Wei X hong, Ji L li. Effect of handball training on cognitive ability in elderly with mild cognitive impairment. *Neurosci Lett*. 2014;566:98-101. doi:10.1016/j.neulet.2014.02.035
17. Nakatsuka M, Nakamura K, Hamanosono R, et al. A Cluster Randomized Controlled Trial of Nonpharmacological Interventions for Old-Old Subjects with a Clinical Dementia Rating of 0.5: The Kurihara Project. *Dement Geriatr Cogn Dis Extra*. 2015;5(2):221-232. doi:10.1159/000380816
18. Kohanpour MA, Peeri M, Azarbayjani MA. The effects of aerobic exercise with lavender essence use on cognitive state and serum brain-derived neurotrophic factor levels in elderly with mild cognitive impairment. *Journal of HerbMed Pharmacology*. 2017;6(2):80-84.
19. Liu IT, Lee WJ, Lin SY, Chang ST, Kao CL, Cheng YY. Therapeutic Effects of Exercise Training on Elderly Patients With Dementia: A Randomized Controlled Trial. *Arch Phys Med Rehabil*. 2020;101(5):762-769. doi:10.1016/j.apmr.2020.01.012
20. Yang SY, Shan CL, Qing H, et al. The Effects of Aerobic Exercise on Cognitive Function of Alzheimer's Disease Patients. *CNS Neurol Disord Drug Targets*. 2015;14(10):1292-1297. doi:10.2174/1871527315666151111123319
21. Khattak HG, Ahmad Z, Arshad H, Anwar K. Effect of aerobic exercise on cognition in elderly persons with mild cognitive impairment. *Rawal Medical Journal*. 2022;47(3):698-701. doi:10.5455/rmj.20210713072242
22. Varela S, Ayán C, Cancela JM, Martín V. Effects of two different intensities of aerobic exercise on elderly people with mild cognitive impairment: A randomized pilot study. *Clin Rehabil*. 2012;26(5):442-450. doi:10.1177/0269215511425835
23. Miu D, Edin F, Szeto S, Mak Y. A randomised controlled trial on the effect of exercise on physical, cognitive and affective function in dementia subjects. *Asian Journal of Gerontology & Geriatrics*. 2008;3(1):8-16.
24. Angiolillo A, Leccese D, Ciccotelli S, et al. Effects of Nordic walking in Alzheimer's disease: A single-blind randomized controlled clinical trial. *Heliyon*. 2023;9(5):e15865. doi:10.1016/j.heliyon.2023.e15865
25. Jatuporn Phoemsapthawee, Watthanaree Ammawat NL. The Benefit of Arm Swing Exercise on Cognitive Performance in Older Women with Mild Cognitive Impairment. *Journal of Exercise Physiology*. 2016;8(1):11-25.
26. Guzel I, Can F. The effects of different exercise types on cognitive and physical functions in dementia patients: A randomized comparative study. *Arch Gerontol Geriatr*. 2024;119(18):105321. doi:10.1016/j.archger.2023.105321
27. Venturelli M, Scarsini R, Schena F. Six-month walking program changes cognitive and ADL performance in patients with Alzheimer. *Am J Alzheimers Dis Other Demen*. 2011;26(5):381-388. doi:10.1177/1533317511418956
28. Yoon DH, Kang D, Kim HJ, Kim JS, Song HS, Song W. Effect of elastic band-based high-speed power training on cognitive function, physical performance and muscle strength in older women with mild cognitive impairment. *Geriatr Gerontol Int*. 2017;17(5):765-772. doi:10.1111/ggi.12784
29. Venturelli M, Lanza M, Muti E, Schena F. Positive effects of physical training in activity of daily living-dependent older adults. *Exp Aging Res*. 2010;36(2):190-205. doi:10.1080/03610731003613771
30. Holthoff VA, Marschner K, Scharf M, et al. Effects of physical activity training in patients with alzheimer's dementia: Results of a pilot RCT study. *PLoS One*. 2015;10(4):1-11. doi:10.1371/journal.pone.0121478
31. Baek JE, Hyeon SJ, Kim M, Cho HY, Hahm SC. Effects of dual-task resistance exercise on cognition, mood, depression, functional fitness, and activities of daily living in older adults with cognitive impairment: a single-blinded, randomized controlled trial. *BMC Geriatr*. 2024;24(1):1-12. doi:10.1186/s12877-024-04942-1
32. Yang JG, Thapa N, Park HJ, et al. Virtual Reality and Exercise Training Enhance Brain, Cognitive, and Physical Health in Older Adults with Mild Cognitive Impairment. *Int J Environ Res Public Health*. 2022;19(20). doi:10.3390/ijerph192013300
33. Li L, Liu M, Zeng H, Pan L. Multi-component exercise training improves the physical and cognitive function of the elderly with mild cognitive impairment: A six-month randomized controlled trial. *Ann Palliat Med*. 2021;10(8):8919-8929. doi:10.21037/apm-21-1809
34. Avenali M, Picascia M, Tassorelli C, Sinforiani E, Bernini S. Evaluation of the efficacy of physical therapy on cognitive decline at 6-month follow-up in Parkinson disease patients with mild cognitive impairment: a randomized controlled trial. *Aging Clin Exp Res*. 2021;33(12):3275-3284. doi:10.1007/s40520-021-01865-4
35. Mak A, Delbaere K, Refshauge K, et al. Sunbeam Program Reduces Rate of Falls in Long-Term Care Residents With Mild to Moderate Cognitive Impairment or Dementia: Subgroup Analysis of a Cluster Randomized Controlled Trial. *J Am Med Dir Assoc*. 2022;23(5):743-749.e1. doi:10.1016/j.jamda.2022.01.064
36. Sobol NA, Hoffmann K, Frederiksen KS, et al. Effect of aerobic exercise on physical performance in patients with Alzheimer's disease. *Alzheimer's and Dementia*. 2016;12(12):1207-1215. doi:10.1016/j.jalz.2016.05.004
37. Hoffmann K, Sobol NA, Frederiksen KS, et al. Moderate-to-high intensity physical exercise in patients with Alzheimer's disease: A randomized controlled trial. *Journal of Alzheimer's Disease*. 2016;50(2):443-453. doi:10.3233/JAD-150817
38. Sobol NA, Dall CH, Høgh P, et al. Change in fitness and the relation to change in cognition and neuropsychiatric symptoms after aerobic exercise in patients with mild Alzheimer's disease. *Journal of Alzheimer's Disease*. 2018;65(1):137-145. doi:10.3233/JAD-180253
39. Lok N, Tosun AS, Lok S, Temel V, Aydın Z. Effect of physical activity program applied to patients with Alzheimer's disease on cognitive functions and depression level: a randomised controlled study. *Psychogeriatrics*. 2023;23(5):856-863. doi:10.1111/psyg.13010
40. De Sá CA, Saretto CB, Cardoso AM, Remor A, Breda CO, da Silva Corralo V. Effects of a physical exercise or motor activity protocol on cognitive function, lipid profile, and BDNF levels in older adults with mild cognitive impairment. *Mol Cell Biochem*. 2024;479(3):499-509. doi:10.1007/s11010-023-04733-z
41. Langoni CDS, Resende TDL, Barcellos AB, et al. Effect of Exercise on Cognition, Conditioning, Muscle Endurance, and Balance in Older Adults with Mild Cognitive Impairment: A Randomized Controlled Trial. *Journal of Geriatric Physical Therapy*. 2019;42(2):E15-E22. doi:10.1519/JPT.0000000000000191
42. Langoni C da S, Resende T de L, Barcellos AB, et al. The effect of group exercises on balance, mobility, and depressive symptoms in older adults with mild cognitive impairment: a randomized controlled trial. *Clin Rehabil*. 2019;33(3):439-449. doi:10.1177/0269215518815218
43. Zhang Q, Zhu M, Huang L, et al. A Study on the Effect of Traditional Chinese Exercise Combined With Rhythm Training on the Intervention of Older Adults With Mild Cognitive Impairment. *Am J Alzheimers Dis Other Demen*. 2023;38(48):1-12. doi:10.1177/15333175231190626

44. Sanders LMJ, Hortobágyi T, Karssemeijer EGA, Van Der Zee EA, Scherder EJA, Van Heuvelen MJG. Effects of low- And high-intensity physical exercise on physical and cognitive function in older persons with dementia: A randomized controlled trial. *Alzheimers Res Ther*. 2020;12(1):1-15. doi:10.1186/s13195-020-00597-3
45. Ghahfarrokhi MM, Shirvani H, Rahimi M, Bazgir B, Shamsadini A, Sobhani V. Feasibility and preliminary efficacy of different intensities of functional training in elderly type 2 diabetes patients with cognitive impairment: a pilot randomised controlled trial. *BMC Geriatr*. 2024;24(1):1-15. doi:10.1186/s12877-024-04698-8
46. Shaw I, Cronje M, Shaw BS. Group-based exercise as a therapeutic strategy for the improvement of mental outcomes in mild to moderate alzheimer's patients in low resource care facilities. *Asian J Sports Med*. 2021;12(1):1-6. doi:10.5812/asjsm.106593
47. Mollinedo Cardalda I, López A, Cancela Carral JM. The effects of different types of physical exercise on physical and cognitive function in frail institutionalized older adults with mild to moderate cognitive impairment. A randomized controlled trial. *Arch Gerontol Geriatr*. 2019;83(May):223-230. doi:10.1016/j.archger.2019.05.003
48. Bossers WJR, Van Der Woude LHV, Boersma F, Hortobágyi T, Scherder EJA, Van Heuvelen MJG. A 9-Week Aerobic and Strength Training Program Improves Cognitive and Motor Function in Patients with Dementia: A Randomized, Controlled Trial. *American Journal of Geriatric Psychiatry*. 2015;23(11):1106-1116. doi:10.1016/j.jagp.2014.12.191
49. Bossers WJR, van der Woude LHV, Boersma F, Hortobágyi T, Scherder EJA, van Heuvelen MJG. Comparison of Effect of Two Exercise Programs on Activities of Daily Living in Individuals with Dementia: A 9-Week Randomized, Controlled Trial. *J Am Geriatr Soc*. 2016;64(6):1258-1266. doi:10.1111/jgs.14160
50. Telenius EW, Engedal K, Bergland A. Long-term effects of a 12 weeks high-intensity functional exercise program on physical function and mental health in nursing home residents with dementia: A single blinded randomized controlled trial Physical functioning, physical health and activity. *BMC Geriatr*. 2015;15(1):1-11. doi:10.1186/s12877-015-0151-8
51. Telenius EW, Engedal K, Bergland A. Effect of a high-intensity exercise program on physical function and mental health in nursing home residents with dementia: An assessor blinded randomized controlled trial. *PLoS One*. 2015;10(5):1-18. doi:10.1371/journal.pone.0126102
52. Henskens M, Nauta IM, Van Eekeren MCA, Scherder EJA. Effects of Physical Activity in Nursing Home Residents with Dementia: A Randomized Controlled Trial. *Dement Geriatr Cogn Disord*. 2018;46(1-2):60-80. doi:10.1159/000491818
53. Esmail A, Vrinceanu T, Lussier M, et al. Effects of Dance/Movement Training vs. Aerobic Exercise Training on cognition, physical fitness and quality of life in older adults: A randomized controlled trial. *J Bodyw Mov Ther*. 2020;24(1):212-220. doi:10.1016/j.jbmt.2019.05.004
54. Franco MR, Sherrington C, Tiedemann A, et al. Effect of Senior Dance (DanSE) on Fall Risk Factors in Older Adults: A Randomized Controlled Trial. *Phys Ther*. 2020;100(4):600-608. doi:10.1093/ptj/pzz187
55. Zhu Y, Wu H, Qi M, et al. Effects of a specially designed aerobic dance routine on mild cognitive impairment. *Clin Interv Aging*. 2018;13:1691-1700. doi:10.2147/CIA.S163067
56. Song D, Yu D, Liu T, Wang J. Effect of an Aerobic Dancing Program on Sleep Quality for Older Adults With Mild Cognitive Impairment and Poor Sleep: A Randomized Controlled Trial. *J Am Med Dir Assoc*. 2024;25(3):494-499. doi:10.1016/j.jamda.2023.09.020
57. Chang J, Zhu W, Zhang J, et al. The Effect of Chinese Square Dance Exercise on Cognitive Function in Older Women With Mild Cognitive Impairment: The Mediating Effect of Mood Status and Quality of Life. *Front Psychiatry*. 2021;12(July). doi:10.3389/fpsyt.2021.711079
58. Chang J, Chen Y, Liu C, et al. Effect of Square Dance Exercise on Older Women With Mild Mental Disorders. *Front Psychiatry*. 2021;12(July):1-9. doi:10.3389/fpsyt.2021.699778
59. Michael Schwenk, Sabbagh M, Lin I, et al. Sensor-based balance training with motion feedback in people with mild cognitive impairment. 2016;53(6):945-958. doi:10.1682/JRRD.2015.05.0089.Sensor-based
60. Liu CL, Cheng FY, Wei MJ, Liao YY. Effects of Exergaming-Based Tai Chi on Cognitive Function and Dual-Task Gait Performance in Older Adults With Mild Cognitive Impairment: A Randomized Control Trial. *Front Aging Neurosci*. 2022;14(March). doi:10.3389/fnagi.2022.761053
61. Liao YY, Chen IH, Hsu WC, Tseng HY, Wang RY. Effect of exergaming versus combined exercise on cognitive function and brain activation in frail older adults: A randomised controlled trial. *Ann Phys Rehabil Med*. 2021;64(5):101492. doi:10.1016/j.rehab.2021.101492
62. Swinnen N, Vandenbulcke M, de Bruin ED, et al. The efficacy of exergaming in people with major neurocognitive disorder residing in long-term care facilities: a pilot randomized controlled trial. *Alzheimers Res Ther*. 2021;13(1):1-13. doi:10.1186/s13195-021-00806-7
63. Khanthong P, Sriyakul K, Dechakhamphu A, Krajarng A, Kamalashiran C, Tungsukruthai P. Traditional Thai exercise (Ruesi Dadton) for improving motor and cognitive functions in mild cognitive impairment: a randomized controlled trial. *J Exerc Rehabil*. 2021;17(5):331-338. doi:10.12965/JER.2142542.271
64. Kashyap M, Rai NK, Singh R, et al. Effect of Early Yoga Practice on Post Stroke Cognitive Impairment. 2022;22(4):2019. doi:10.4103/aian.AIAN
65. Li F, Harmer P, Fitzgerald K, Winters-Stone K. A cognitively enhanced online Tai Ji Quan training intervention for community-dwelling older adults with mild cognitive impairment: A feasibility trial. *BMC Geriatr*. 2022;22(1):1-13. doi:10.1186/s12877-021-02747-0
66. Chen Y, Qin J, Tao L, et al. Effects of Tai Chi Chuan on Cognitive Function in Adults 60 Years or Older With Type 2 Diabetes and Mild Cognitive Impairment in China: A Randomized Clinical Trial. *JAMA Netw Open*. 2023;6(4):E237004. doi:10.1001/jamanetworkopen.2023.7004
67. Yu AP, Chin EC, Yu DJ, et al. Tai Chi versus conventional exercise for improving cognitive function in older adults: a pilot randomized controlled trial. *Sci Rep*. 2022;12(1):1-15. doi:10.1038/s41598-022-12526-5
68. Zheng G, Zheng Y, Xiong Z, Ye B. Effect of Baduanjin exercise on cognitive function in patients with post-stroke cognitive impairment: a randomized controlled trial. *Clin Rehabil*. 2020;34(8):1028-1039. doi:10.1177/0269215520930256
69. Xia R, Wan M, Lin H, Ye Y, Chen S, Zheng G. Effects of mind–body exercise Baduanjin on cognition in community-dwelling older people with mild cognitive impairment: A randomized controlled trial. *Neuropsychol Rehabil*. 2023;33(8):1368-1383. doi:10.1080/09602011.2022.2099909
70. Zheng G, Ye B, Xia R, et al. Traditional Chinese Mind-Body Exercise Baduanjin Modulate Gray Matter and Cognitive Function in Older Adults with Mild Cognitive Impairment: A Brain Imaging Study. *Brain Plasticity*. 2021;7(2):131-142. doi:10.3233/bpl-210121
71. Liu J, Tao J, Xia R, et al. Mind-Body Exercise Modulates Locus Coeruleus and Ventral Tegmental Area Functional Connectivity in Individuals With Mild Cognitive Impairment. *Front Aging Neurosci*. 2021;13(June):1-12. doi:10.3389/fnagi.2021.646807
72. Tao J, Liu J, Chen X, et al. Mind-body exercise improves cognitive function and modulates the function and structure of the hippocampus and anterior cingulate cortex in patients with mild cognitive impairment. *Neuroimage Clin*. 2019;23(April):101834. doi:10.1016/j.nicl.2019.101834
73. Xia R, Qiu P, Lin H, et al. The effect of traditional chinese mind-body exercise (Baduanjin) and brisk walking on the dorsal attention network in older adults with mild cognitive impairment. *Front Psychol*. 2019;10(SEP):1-9. doi:10.3389/fpsyg.2019.02075
74. Song D, Yu DSF. Effects of a moderate-intensity aerobic exercise programme on the cognitive function and quality of life of community-dwelling elderly people with mild cognitive impairment: A randomised controlled trial. *Int J Nurs Stud*. 2019;93:97-105. doi:10.1016/j.ijnurstu.2019.02.019
75. Rojasavastera R, Bovonsunthonchai S, Hiengkaew V, Senanarong V. Action observation combined with gait training to improve gait and cognition in elderly with mild cognitive impairment a randomized controlled trial. *Dementia e Neuropsychologia*. 2020;14(2):118-127. doi:10.1590/1980-57642020dn14-020004
76. Karthikeyan T. Therapeutic effects of home-based exercise of geriatrics for the management of cognitive impairment. *ES J Public Health*. 2020;1(1):1003. www.escientificlibrary.com
77. Krootnark K, Chaikeeree N, Saengsirisuwan V, Boonsinsukh R. Effects of low-intensity home-based exercise on cognition in older persons with mild cognitive impairment: a direct comparison of aerobic versus resistance exercises using a randomized controlled trial design. *Front Med (Lausanne)*. 2024;11(June):1-11. doi:10.3389/fmed.2024.1392429
78. Choi W, Lee S. Ground kayak paddling exercise improves postural balance, muscle performance, and cognitive function in older adults with mild cognitive impairment: A randomized controlled trial. *Medical Science Monitor*. 2018;24:3909-3915. doi:10.12659/MSM.908248
79. Choi W, Lee S. The effects of virtual kayak paddling exercise on postural balance, muscle performance, and cognitive function in older adults with mild cognitive impairment: A randomized controlled trial. *J Aging Phys Act*. 2019;27(6):861-870. doi:10.1123/japa.2018-0020
80. Amjad I, Toor H, Niazi IK, et al. Therapeutic effects of aerobic exercise on EEG parameters and higher cognitive functions in mild cognitive impairment patients. *International Journal of Neuroscience*. 2019;129(6):551-562. doi:10.1080/00207454.2018.1551894
81. Wang L, Wu B, Tao H, et al. Effects and mediating mechanisms of a structured limbs-exercise program on general cognitive function in older adults with mild cognitive impairment: A randomized controlled trial. *Int J Nurs Stud*. 2020;110:103706. doi:10.1016/j.ijnurstu.2020.103706
82. Hong SG, Kim JH, Jun TW. Effects of 12-week resistance exercise on electroencephalogram patterns and cognitive function in the elderly with mild cognitive impairment: A randomized controlled trial. *Clinical Journal of Sport Medicine*. 2018;28(6):500-508. doi:10.1097/JSM.0000000000000476
83. Greblo Jurakic Z, Krizanic V, Sarabon N, Markovic G. Effects of feedback-based balance and core resistance training vs. Pilates training on cognitive functions in older women with mild cognitive impairment: a pilot randomized controlled trial. *Aging Clin Exp Res*. 2017;29(6):1295-1298. doi:10.1007/s40520-017-0740-9
84. Kim J, Yim J. Effects of an exercise protocol for improving handgrip strength and walking speed on cognitive function in patients with chronic stroke. *Medical Science Monitor*. 2017;23:5402-5409. doi:10.12659/MSM.904723
85. Rivas-Campo Y, Aibar-Almazán A, Afanador-Restrepo DF, et al. Effects of High-Intensity Functional Training (HIFT) on the Functional Capacity, Frailty, and Physical Condition of Older Adults with Mild Cognitive Impairment: A Blind Randomized Controlled Clinical Trial. *Life*. 2023;13(5):1-16. doi:10.3390/life13051224
86. Rivas-Campo Y, Aibar-Almazán A, Rodríguez-López C, et al. Enhancing Cognition in Older Adults with Mild Cognitive Impairment through High-Intensity Functional Training: A Single-Blind Randomized Controlled Trial. *J Clin Med*. 2023;12(12):1-12. doi:10.3390/jcm12124049
87. Levinger P, Goh AMY, Dunn J, et al. Exercise interveNtion outdoor proJect in the cOmmunitY – results from the ENJOY program for independence in dementia: a feasibility pilot randomised controlled trial. *BMC Geriatr*. 2023;23(1):1-16. doi:10.1186/s12877-023-04132-5
88. Akbuga Koc E, Yazici-Mutlu Ç, Cinar N, Sahiner T. Comparison of the effect of online physical exercise and computerized cognitive stimulation in patients with Alzheimer's disease during the Covid-19 pandemic. *Complement Ther Clin Pract*. 2024;57(May):10-20. doi:10.1016/j.ctcp.2024.101881
89. Ho RTH, Fong TCT, Chan WC, et al. Psychophysiological Effects of Dance Movement Therapy and Physical Exercise on Older Adults with Mild Dementia: A Randomized Controlled Trial. *Journals of Gerontology - Series B Psychological Sciences and Social Sciences*. 2018;75(3):560-570. doi:10.1093/geronb/gby145
90. Su H, Wang H, Meng L, Bush E. The effects of Baduanjin exercise on the subjective memory complaint of older adults: A randomized controlled trial. *Medicine (United States)*. 2021;100(30):E25442. doi:10.1097/MD.0000000000025442
91. Fernandez-Gonzalo R, Fernandez-Gonzalo S, Turon M, Prieto C, Tesch PA, García-Carreira MDC. Muscle, functional and cognitive adaptations after flywheel resistance training in stroke patients: A pilot randomized controlled trial. *J Neuroeng Rehabil*. 2016;13(1):1-11. doi:10.1186/s12984-016-0144-7
92. Yoon DH, Lee JY, Song W. Effects of Resistance Exercise Training on Cognitive Function and Physical Performance in Cognitive Frailty: A Randomized Controlled Trial. *Journal of Nutrition, Health and Aging*. 2018;22(8):944-951. doi:10.1007/s12603-018-1090-9
93. Fonte C, Smania N, Pedrinolla A, et al. Comparison between physical and cognitive treatment in patients with MCI and Alzheimer's disease. *Aging*. 2019;11(10):3138-3155. doi:10.18632/aging.101970
94. Eyre HA, Siddarth P, Acevedo B, et al. A randomized controlled trial of Kundalini yoga in mild cognitive impairment. *Int Psychogeriatr*. 2017;29(4):557-567. doi:10.1017/S1041610216002155
95. Eyre HA, Acevedo B, Yang H, et al. Changes in Neural Connectivity and Memory Following a Yoga Intervention for Older Adults: A Pilot Study. *Journal of Alzheimer's Disease*. 2016;52(2):673-684. doi:10.3233/JAD-150653
96. Lv J, Liu Y. Effects of momentum-based dumbbell training on motor control in older adults with mild cognitive impairment. *Chinese Journal of Rehabilitation Medicine*. 2019;34(5):544-550. doi:10.3969/j.issn.1001-1242.2019.05.009
97. Dawson N, Judge KS, Gerhart H. Improved Functional Performance in Individuals with Dementia after a Moderate-Intensity Home-Based Exercise Program: A Randomized Controlled Trial. *Journal of Geriatric Physical Therapy*. 2019;42(1):18-27. doi:10.1519/JPT.0000000000000128
98. Bo W, Lei M, Tao S, et al. Effects of combined intervention of physical exercise and cognitive training on cognitive function in stroke survivors with vascular cognitive impairment: a randomized controlled trial. *Clin Rehabil*. 2019;33(1):54-63. doi:10.1177/0269215518791007
99. Combourieu Donnezan L, Perrot A, Belleville S, Bloch F, Kemoun G. Effects of simultaneous aerobic and cognitive training on executive functions, cardiovascular fitness and functional abilities in older adults with mild cognitive impairment. *Ment Health Phys Act*. 2018;15(April):78-87. doi:10.1016/j.mhpa.2018.06.001
100. Eggermont LHP, Swaab DF, Hol EM, Scherder EJA. Walking the line: A randomised trial on the effects of a short term walking programme on cognition in dementia. *J Neurol Neurosurg Psychiatry*. 2009;80(7):802-804. doi:10.1136/jnnp.2008.158444
101. Li PWC, Yu DSF, Siu PM, Wong SCK, Chan BS. Peer-supported exercise intervention for persons with mild cognitive impairment: A waitlist randomised controlled trial (the BRAin Vitality Enhancement trial). *Age Ageing*. 2022;51(10):1-10. doi:10.1093/ageing/afac213
102. Prick AE, De Lange J, Scherder E, Twisk J, Pot AM. The effects of a multicomponent dyadic intervention with physical exercise on the cognitive functioning of people with dementia: A randomized controlled trial. *J Aging Phys Act*. 2017;25(4):539-552. doi:10.1123/japa.2016-0038
103. Cheng ST, Chow PK, Song YQ, et al. Mental and physical activities delay cognitive decline in older persons with dementia. *American Journal of Geriatric Psychiatry*. 2014;22(1):63-74. doi:10.1016/j.jagp.2013.01.060
104. Cheng ST, Chow PK, Yu ECS, Chan ACM. Leisure activities alleviate depressive symptoms in nursing home residents with very mild or mild dementia. *American Journal of Geriatric Psychiatry*. 2012;20(10):904-908. doi:10.1097/JGP.0b013e3182423988
105. Ullrich P, Werner C, Schönstein A, et al. Effects of a Home-Based Physical Training and Activity Promotion Program in Community-Dwelling Older Persons with Cognitive Impairment after Discharge from Rehabilitation: A Randomized Controlled Trial. *Journals of Gerontology* -

Series A Biological Sciences and Medical Sciences. 2022;77(12):2435-2444. doi:10.1093/gerona/glac005

106. Vreugdenhil A, Cannell J, Davies A, Razay G. A community-based exercise programme to improve functional ability in people with Alzheimer's disease: A randomized controlled trial. *Scand J Caring Sci*. 2012;26(1):12-19. doi:10.1111/j.1471-6712.2011.00895.x
107. Bracco L, Pinto-Carral A, Hillaert L, Mourey F. Tango-therapy vs physical exercise in older people with dementia; a randomized controlled trial. *BMC Geriatr*. 2023;23(1):1-13. doi:10.1186/s12877-023-04342-x
108. Karssemeijer EGA, Aaronson JA, Bossers WJR, Donders R, Olde Rikkert MGM, Kessels RPC. The quest for synergy between physical exercise and cognitive stimulation via exergaming in people with dementia: A randomized controlled trial. *Alzheimers Res Ther*. 2019;11(1):1-13. doi:10.1186/s13195-018-0454-z
109. Karssemeijer EGA, Bossers WJR, Aaronson JA, Sanders LMJ, Kessels RPC, Olde Rikkert MGM. Exergaming as a Physical Exercise Strategy Reduces Frailty in People With Dementia: A Randomized Controlled Trial. *J Am Med Dir Assoc*. 2019;20(12):1502-1508.e1. doi:10.1016/j.jamda.2019.06.026
110. Li F, Harmer P, Voit J, Chou LS. Implementing an online virtual falls prevention intervention during a public health pandemic for older adults with mild cognitive impairment: A feasibility trial. *Clin Interv Aging*. 2021;16:973-983. doi:10.2147/CIA.S306431
111. Liu JYW, Kwan RYC, Lai CKY, Hill KD. A simplified 10-step Tai-chi programme to enable people with dementia to improve their motor performance: a feasibility study. *Clin Rehabil*. 2018;32(12):1609-1623. doi:10.1177/0269215518786530
112. Nyman SR, Ingram W, Sanders J, et al. Randomised controlled trial of the effect of tai chi on postural balance of people with dementia. *Clin Interv Aging*. 2019;14:2017-2029. doi:10.2147/CIA.S228931
113. Williams J, Nyman S. A secondary analysis of a randomised controlled trial to investigate the effect of Tai Chi on the instrumented timed up and go test in people with mild to moderate dementia. *Aging Clin Exp Res*. 2021;33(8):2175-2181. doi:10.1007/s40520-020-01741-7
114. Dillon K, Prapavessis H. REducing SEDENTary behavior among mild to moderate cognitively impaired assisted living residents: A pilot randomized controlled trial (RESEDENT study). *J Aging Phys Act*. 2021;29(1):27-35. doi:10.1123/JAPA.2019-0440
115. Abbas RL, Saab IM, Al-Sharif HK, Naja N, El-Khatib A. Effect of Adding Motorized Cycle Ergometer Over Exercise Training on Balance in Older Adults with Dementia: A Randomized Controlled Trial. *Exp Aging Res*. 2023;49(2):100-111. doi:10.1080/0361073X.2022.2046947
116. Hauer K, Schwenk M, Zieschang T, Essig M, Becker C, Oster P. Physical training improves motor performance in people with dementia: A randomized controlled trial. *J Am Geriatr Soc*. 2012;60(1):8-15. doi:10.1111/j.1532-5415.2011.03778.x
117. Zieschang T, Schwenk M, Oster P, Hauer K. Sustainability of motor training effects in older people with dementia. *Journal of Alzheimer's Disease*. 2013;34(1):191-202. doi:10.3233/JAD-120814
118. Schwenk M, Zieschang T, Englert S, Grewal G, Najafi B, Hauer K. Improvements in gait characteristics after intensive resistance and functional training in people with dementia: A randomised controlled trial. *BMC Geriatr*. 2014;14(1):1-9. doi:10.1186/1471-2318-14-73
119. Suttanon P, Hill KD, Said CM, et al. Feasibility, safety and preliminary evidence of the effectiveness of a home-based exercise programme for older people with Alzheimer's disease: A pilot randomized controlled trial. *Clin Rehabil*. 2013;27(5):427-438. doi:10.1177/0269215512460877
120. Gebhard D, Mess F. Feasibility and Effectiveness of a Biography-Based Physical Activity Intervention in Institutionalized People With Dementia: Quantitative and Qualitative Results From a Randomized Controlled Trial. *J Aging Phys Act*. 2022;30(2):237-251. doi:10.1123/japa.2020-0343
121. Cezar NO de C, Ansai JH, Oliveira MPB de, et al. Feasibility of improving strength and functioning and decreasing the risk of falls in older adults with Alzheimer's dementia: a randomized controlled home-based exercise trial. *Arch Gerontol Geriatr*. 2021;96(March). doi:10.1016/j.archger.2021.104476
122. Cezar NO de C, Aprahamian I, Ansai JH, et al. Feasibility of reducing frailty components in older adults with Alzheimer's dementia: a randomized controlled home-based exercise trial (AD-HOMEX). *Exp Gerontol*. 2021;150(May). doi:10.1016/j.exger.2021.111390
123. Lawla L.F. Law, Vincent C.T. Mok, Matthew K.S. Yau KNKF. Effects of functional task exercise on everyday problem-solving ability and functional status in older adults with mild cognitive impairment—a randomised controlled trial. *Age Ageing*. 2021;51(7):1-11. doi:10.1093/ageing/afac144
124. Law LLF, Mok VCT, Yau MMK. Effects of functional tasks exercise on cognitive functions of older adults with mild cognitive impairment: A randomized controlled pilot trial. *Alzheimers Res Ther*. 2019;11(1). doi:10.1186/s13195-019-0548-2
125. Toots A, Littbrand H, Boström G, et al. Effects of exercise on cognitive function in older people with dementia: A randomized controlled trial. *Journal of Alzheimer's Disease*. 2017;60(1):323-332. doi:10.3233/JAD-170014
126. Boström G, Conradsson M, Hörnsten C, et al. Effects of a high-intensity functional exercise program on depressive symptoms among people with dementia in residential care: a randomized controlled trial. *Int J Geriatr Psychiatry*. 2016;31(8):868-878. doi:10.1002/gps.4401
127. Toots A, Lindelöf N, Littbrand H, et al. Effects of a High-Intensity Functional Exercise Program on Dependence in Activities of Daily Living and Balance in Older Adults with Dementia. *J Am Geriatr Soc*. 2016;64(1):55-64. doi:10.1111/jgs.13880
128. Toots A, Littbrand H, Holmberg H, et al. Walking Aids Moderate Exercise Effects on Gait Speed in People With Dementia: A Randomized Controlled Trial. *J Am Med Dir Assoc*. 2017;18(3):227-233. doi:10.1016/j.jamda.2016.09.003
129. Toots A, Lundin-Olsson L, Nordström P, Gustafson Y, Rosendahl E. Exercise effects on backward walking speed in people with dementia: A randomized controlled trial. *Gait Posture*. 2021;85(January):65-70. doi:10.1016/j.gaitpost.2020.12.028
130. Doi T, Makizako H, Shimada H, et al. Effects of multicomponent exercise on spatial-temporal gait parameters among the elderly with amnestic mild cognitive impairment (aMCI): Preliminary results from a randomized controlled trial (RCT). *Arch Gerontol Geriatr*. 2013;56(1):104-108. doi:10.1016/j.archger.2012.09.003
131. Lamb SE, Sheehan B, Atherton N, et al. Dementia And Physical Activity (DAPA) trial of moderate to high intensity exercise training for people with dementia: Randomised controlled trial. *BMJ (Online)*. 2018;361. doi:10.1136/bmj.k1675
132. Smith TO, Mistry D, Lee H, et al. Moderators of Cognitive Outcomes from an Exercise Program in People with Mild to Moderate Dementia. *J Am Geriatr Soc*. 2020;68(9):2095-2100. doi:10.1111/jgs.16552
133. Wu VX, Chi Y, Lee JK, et al. The effect of dance interventions on cognition, neuroplasticity, physical function, depression, and quality of life for older adults with mild cognitive impairment: A systematic review and meta-analysis. *Int J Nurs Stud*. 2021;122:104025. doi:10.1016/j.ijnurstu.2021.104025
134. Rolland Y, Pillard F, Klapouszczak A, et al. Exercise program for nursing home residents with Alzheimer's disease: A 1-year randomized, controlled trial. *J Am Geriatr Soc*. 2007;55(2):158-165. doi:10.1111/j.1532-5415.2007.01035.x
