# Supplementary material for: Dance versus other exercise modalities in mild cognitive impairment and dementia: comparative efficacy from a systematic review and bayesian network meta-analysis
Source: Front Physiol. 2026 Mar 25;17:1782774. doi: 10.3389/fphys.2026.1782774 (PMC13056856; doi:10.3389/fphys.2026.1782774)
Supplement: Supplementary file 8 [file Table7.pdf]

Supplementary Table 7. Numerical Summary of Study Characteristics

Items	Number	(%)
**Dance** (Total 17 studies)		
**<u>Mean Age of Participants</u>** (We used only the mean value and ignored the standard deviation)		
66–70 [1–6,7]	7	41%
71–75 [8–10,11]	4	23.5%
76–80 [12,13,14,15]	4	23.5%
81–85 [16,17]	2	12%
**<u>Gender</u>**		
**Female**		
76[1] 31[2] 15[8] 23[3] 101[4] 65[5] 104[12] 14[13] 36[6] 68[9] 109[14] 25[16] 26[17] 41[10] 167[15] 27[7] 58[11]	986	73%
**Male**		
23[1] 10[2] 16[8] 9[3] 28[4] 6[5] 97[12] 11[13] 24[6] 21[9] 5[17] 27[10] 37[15] 23[7] 34[11]	371	27%
**Total**	1357	
**<u>Sample Size</u>**		
21–25 [13,16]	2	12%
31–35 [3,8,17]	3	18%
41–45 [2]	1	5.8%
46-50 [7]	1	5.8%
56–60 [6]	1	5.8%
66–70 [10]	1	5.8%
71–75 [5]	1	5.8%
86–90 [9]	1	5.8%
91-95 [11]	1	5.8%
96–100 [1]	1	5.8%
106–110 [14]	1	5.8%
126–130 [4]	1	5.8%
201–205 [12,15]	2	12%
**<u>Diseases Categories</u>** (As defined by the included studies)		
MCI [1,3–6,9,12,14,7]	9	53%
Dementia [15–17]	3	18%
Healthy/older inactive senior [1,2]	2	12%
aMCI [8,10]	2	12%
Dementia at risk [13]	1	5%
**<u>Diagnosis Instruments</u>**		
MMSE [2,3,5,6,8,10,16,11,7]	8	47%
MoCA [3,6,9,10,13,14]	6	35%
NIA-AA [3,6,10]	3	18%
CDR [8,15]	2	12%
Petersen criteria [4,12]	2	12%
GDS [4]	1	6%
NINCDS-ARDRA [16]	1	6%
Cognitive test battery [1]	1	6%
**N/A** [17]	1	6%
**<u>Cognitive Impairment Level</u>** (We used only the mean value and ignored the standard deviation)		
**MMSE**		
≤ 9 Sever cognitive impairment [17]	1	6%
10–20 Moderate cognitive impairment [16]	1	6%
21–26 Mild cognitive impairment [5,12]	2	12%
27–30 Normal cognition [2–4,8,7,11]	6	35%
**MoCA**		
18–25 Mild cognitive impairment [3–6,9,13,14,11]	8	47%

26–30 Normal cognition [1]	1	6%
**N/A** [15,10]	2	12%
**Mean Education Years of Participants** (Only included the studies that provided education background data in Mean ± SD format)		
6–10 [3,8,10,14]	4	24%
11–15 [1,2,4,12,7]	5	29%
16–20 [13]	1	6%
**N/A** [5,6,9,15–17,11]	7	41%
**Training Period**		
12 weeks [2,3,5,6,8,10,15–17,11]	10	59%
16 weeks [9]	1	5.5%
18 weeks [14]	1	5.5%
24 weeks [1,13,7]	3	18%
40 weeks [4,12]	2	12%
**Duration of Training Session**		
30-mins/time [16]	1	5.5%
35-mins/time [3,6,10]	3	18%
40-mins/time [14]	1	5.5%
60-mins/time [1,2,4,5,8,9,12,15,17,11]	10	59%
90-mins/time [13,7]	2	12%
**Training Frequency**		
1 time/week [12]	1	6%
2 times/week [4,5,8,13,15,17,11,7]	8	47%
3 times/week [1–3,6,9,10,14]	7	41%
7 times/week [16]	1	6%
**Training Intensity**		
60–80% of HRmax [3,6,10]	3	17.6%
100–140 beats/min HR [14]	1	5.85%
≤ 14/20 RPE [13]	1	5.85%
2–3/10 RPE [8]	1	5.85%
40–60% of VO_2max [15]	1	5.85%
**N/A** [1,2,4,5,12,9,16,17,7,11]	10	59%
**Training Load Monitoring**		
HR monitor [3,6,10,14]	4	23%
RPE [8,13]	2	12%
**N/A** [1,2,4,5,9,12,15–17,7,11]	11	65%
**Adherence Rate%** (If there was more than one intervention group, we used the mean value of the groups)		
**IG**		
61–65% [2,13]	2	12%
71–75% [4]	1	5.6%
76–80% [9]	1	5.6%
81–85% [1,3,5,12]	4	23.5%
86–90% [14,17]	2	12%
91–95% [6,8,10,7]	4	23.5%
96–100% [16,11]	2	12%
**CG**		
66–70% [13]	1	5.6%
71–75% [14]	1	5.6%
76–80% [2,8,9]	3	18%
81-85% [1,3,17]	3	18%

86–90% [5]	1	5.6%
91–95% [4,12,7,11]	4	23.5%
96–100% [6,10,16]	3	18%
**N/A** [15]	1	5.6%
**Publication Year**		
2004 [16]	1	5.6%
2017 [4,12]	2	12%
2018 [3,6,15]	3	18%
2019 [1]	1	5.6%
2020 [2,5,8]	3	18%
2021 [14]	1	5.6%
2022 [10]	1	5.6%
2023 [13,17]	2	12%
2024 [9,7]	2	12%
2025 [11]	1	5.6%
**Exergaming** (Total 12 studies)		
**Mean Age of Participants** (We used only the mean value and ignored the standard deviation)		
71–75 [18,19]	2	17%
76–80 [20–26]	7	58%
81–85 [27–29]	3	25%
**Gender**		
**Female**		
46[20] 14[21] 12[22] 35[18] 16[23] 31[27] 53[24] 52[25] 16[26] 35[28] 29[29] 9[19]	348	58%
**Male**		
25[20] 6[21] 10[22] 15[18] 6[23] 15[27] 62[24] 60[25] 8[26] 10[28] 9[29] 23[19]	249	42%
**Total**	597	
**Sample Size**		
16–20 [21]	1	8.2%
21–25 [22,23,26]	3	25%
31-35 [19]	1	8.2%
36–40 [29]	1	8.2%
41–45 [28]	1	8.2%
46–50 [18,27]	2	17%
71–75 [20]	1	8.2%
111–115 [24,25]	2	17%
**Diseases Categories** (As defined by the included studies)		
MCI [18,21,22,27]	4	33%
Dementia [24–26,29]	4	33%
Healthy/older inactive senior [20]	1	8.5%
AD [23,19]	2	17%
Major neurocognitive disorder [28]	1	8.5%
**Diagnosis Instruments**		
MMSE [23,24,26,28]	4	33%
DSM [23,24]	2	17%
Diagnosed by a specialist [29]	1	8%
MoCA [22]	1	8%
Petersen criteria [18]	1	8%
Korean consortium to establish a registry for Alzheimer's Disease test battery [26]	1	8%
NINCDS-ADRDA [19]	1	8%
**N/A** [20,21,25,27]	4	33%

**Cognitive Impairment Level** (We used only the mean value and ignored the standard deviation)		
**MMSE**		
10–20 Moderate cognitive impairment [25,26,28,29,19]	5	42%
21–26 Mild cognitive impairment [18,23,24,27]	4	33%
27–30 Normal cognition [20,21]	2	17%
**MoCA**		
≤ 9 Sever cognitive impairment [28]	1	8%
18–25 Mild cognitive impairment [18,22,27]	3	25%
**Mean Education Years of Participants** (Only included the studies that provided education background data in Mean ± SD format)		
11–15 [20–23]	4	33%
**N/A** [18,24–29,19]	8	67%
**Training Period**		
4 weeks [22]	1	8.5%
6-week [19]	1	8.5%
8 weeks [23,28,29]	3	25%
12 weeks [18,24,26,27]	4	33%
24 weeks [20,21,25]	3	25%
**Duration of Training Session**		
15-mins/time [28]	1	8.3%
30-mins/time [23,19]	2	17%
45-mins/time [22]	1	8.3%
50-mins/time [18,24,26]	3	25%
60-mins/time [20,25,27,29]	4	33%
90-mins/time [21]	1	8.3%
**Training Frequency**		
1 time/week [21]	1	8%
2 times/week [20,22,19]	3	25%
3 times/week [18,24,26–28]	5	42%
5 times/week [23,25,29]	3	25%
**Training Intensity**		
50–75% of HRmax [27]	1	8%
60–70% of HRmax [26]	1	8%
65–75% of HRR [24]	1	8%
5–7/10 RPE [20]	1	8%
12–14/20 RPE [18]	1	8%
13–14/20 RPE [27]	1	8%
**N/A** [22,21,23,25,28,29,19]	7	58%
**Training Load Monitoring**		
HR monitor [24,26,27]	3	25%
RPE [18,27]	2	17%
**N/A** [20–23,25,28,29,19]	8	67%
**Adherence Rate%** (If there was more than one intervention group, we used the mean value of the groups)		
**IG**		
50–55% [26]	1	8%
71–75% [29]	1	8%
76–80% [20,25]	2	17%
81–85% [27,28]	2	17%
86–90% [24]	1	8%
91–95% [18,22]	2	17%
96–100% [21,23,19]	3	25%

**CG**		
41–45% [26]	1	8.25%
66–70% [27]	1	8.25%
75-80% [20]	1	8.25%
81–85% [25,28,29]	3	25%
86-90% [22,24]	2	17%
91–95% [18]	1	8.25%
96–100% [21,23,19]	3	25%
**Publication Year**		
2012 [23]	1	8.25%
2014 [21]	1	8.25%
2015 [20]	1	8.25%
2016 [22]	1	8.25%
2019 [24]	1	8.25%
2020 [25]	1	8.25%
2021 [27,28]	2	17%
2022 [18,29]	2	17%
2023 [26]	1	8.25%
2025 [19]	1	8.25%
**Yoga** (Total 5 studies)		
**Mean Age of Participants** (We used only the mean value and ignored the standard deviation)		
51–55 [30]	1	20%
56–60 [31]	1	20%
66–70 [32,33]	2	40%
71–75 [34]	1	20%
**Gender**		
**Female**		
79[32] 56[31] 25[30] 16[34] 52[33]	228	64%
**Male**		
15[31] 55[30] 30[34] 27[33]	127	36%
**Total**	355	
**Sample Size**		
46–50 [34]	1	20%
71–75 [31]	1	20%
76–80 [30,32,33]	3	60%
**Diseases Categories** (As defined by the included studies)		
MCI [31,33,34]	3	60%
AD at risk [32]	1	20%
MCI due to stroke [30]	1	20%
**Diagnosis Instruments**		
Diagnosed by a specialist [31]	1	20%
MMSE [31]	1	20%
MoCA [31]	1	20%
CDR [33]	1	20%
NIA-AA [34]	1	20%
**N/A** [30,32]	2	40%
**Cognitive Impairment Level** (We used only the mean value and ignored the standard deviation)		
**MMSE**		
21–26 Mild cognitive impairment [31]	1	20%
27–30 Normal cognition [32]	1	20%

**MoCA**		
18–25 Mild cognitive impairment [30,31]	2	40%
**N/A** [33,34]	2	40%
**Mean Education Years of Participants** (Only included the studies that provided education background data in Mean ± SD format)		
11–15 [32,34]	2	40%
16–20 [33]	1	20%
**N/A** [30,31]	2	40%
**Training Period**		
12 weeks [31–34]	4	80%
24 weeks [30]	1	20%
**Duration of Training Session**		
60-mins/time [30–34]	5	100%
**Training Frequency**		
1 time/week [32,33]	2	40%
2 times/week [34]	1	20%
3 times/week [31]	1	20%
5 times/week [30]	1	20%
**Training Intensity**		
**N/A** [30–34]	5	100%
**Training Load Monitoring**		
**N/A** [30–34]	5	100%
**Adherence %** (If there was more than one intervention group, we used the mean value of the groups)		
**IG**		
71–75% [30]	1	20%
76–80% [31,33]	2	40%
96–100% [34]	1	20%
**CG**		
71–75% [30]	1	20%
76–80% [31,33]	2	40%
96–100% [34]	1	20%
**N/A** [32]	1	20%
**Publication Year**		
2017 [33]	1	20%
2021 [31]	1	20%
2022 [30,34]	2	40%
2024 [32]	1	20%
**Chinese Traditional Exercises (CTE)** (Total 22 studies)		
**Mean Age of Participants** (We used only the mean value and ignored the standard deviation)		
56–60 [35]	1	5%
61–65 [36–39,40]	5	23%
66–70 [41–44]	4	18%
71–75 [45–47,48]	4	18%
76–80 [49–53,54]	6	27%
81–85 [55,56]	2	9%
**Gender**		
**Female**		

39[45] 57[41] 21[49] 297[50] 51[46] 167[42] 56[43] 25[44] 54[55] 71[56] 17[51] 44[52] 34[53] 15[47] 36[36] 7[37] 46[38] 29[35] 38[39] 25[40] 60[54] 51[48]	1240	62%
**Male**		
30[45] 9[41] 9[49] 92[50] 40[46] 161[42] 40[43] 9[44] 26[55] 39[56] 9[51] 8[52] 51[53] 27[47] 29[36] 41[37] 23[38] 31[35] 12[39] 18[40] 21[54] 29[48]	754	38%
**Total**	1994	
**Sample Size**		
26–30 [49,51]	2	9%
31–35 [44]	1	4.55%
41–45 [47,40]	2	9%
46–50 [37,39]	2	9%
51–55 [52]	1	4.55%
56–60 [35]	1	4.55%
61–65 [36]	1	4.55%
66–70 [38,41,45]	3	14%
76–80 [55,48]	2	9%
81–85 [53,54]	2	9%
91–95 [46]	1	4.55%
96–100 [43]	1	4.55%
106–110 [56]	1	4.55%
326–330 [42]	1	4.55%
386–390 [50]	1	4.55%
**Diseases Categories** (As defined by the included studies)		
MCI [35,38,39,44,45,47,49,48]	8	36%
Dementia [51,53,55,56]	4	18%
aMCI [41,43]	2	9%
Healthy/older inactive/frailty senior [46]	1	4.6%
Older adults with memory complaint [36]	1	4.6%
CI [52]	1	4.6%
Risk of cognitive decline [50]	1	4.6%
CI due to stroke [37]	1	4.6%
MCI due to T2D [42]	1	4.6%
MCI due to PD [40]	1	4.6%
Mild to moderate cognitive impairment [54]	1	4.6%
**Diagnosis Instruments**		
MMSE [35,41,45,47,49,52,56,54,48]	9	41%
MoCA [38,39,41,43,44,47,51]	7	32%
CDR [45–47,49,50,55,56]	7	32%
DSM [37,55]	2	9%
Petersen criteria [41]	1	4.5%
GDS [43]	1	4.5%
M-ACE [53]	1	4.5%
MRI [37]	1	4.5%
Diagnosed by a specialist [40]	1	4.5%
**N/A** [36,42]	2	9%
**Cognitive Impairment Level** (We used only the mean value and ignored the standard deviation)		
**MMSE**		
10–20 Moderate cognitive impairment [52,55,56,54]	4	18%
21–26 Mild cognitive impairment [45,41,49,50,46,52,35,48]	8	36%
**MoCA**		
10–17 Moderate cognitive impairment [51,55]	2	9%
18–25 Mild cognitive impairment [35,37–39,41–45]	9	41%
26–30 Normal cognition [36,40]	2	9%
**N/A** [53,47]	2	9%

**Mean Education Years of Participants** (Only included the studies that provided education background data in Mean ± SD format)		
0–5 [50]	1	5%
6–10 [37,39,41,42]	4	18%
11–15 [43,44]	2	9%
**N/A** [35,36,38,45–47,49,51–53,55,56,40,54,48]	15	68%
**Training Period**		
8 weeks [52]	1	4.5%
12 weeks [35,36,43,47,56,40,54,48]	8	36%
16 weeks [45,51]	2	9%
20 weeks [53]	1	4.5%
24 weeks [37,38,41,42,44,46,49]	7	32%
40 weeks [39,55]	2	9%
48 weeks [50]	1	4.5%
**Duration of Training Session**		
20-mins/time [55]	1	5%
30-mins/time [50,48]	2	9%
40-mins/time [37,47]	2	9%
45-mins/time [35,53]	2	9%
50-mins/time [41,54]	2	9%
60-mins/time [36,38,39,42–46,49,51,52,56,40]	13	59%
**Training Frequency**		
1 time/week [39,53]	2	9%
2 times/week [45–47,49,51,52,40]	7	32%
3 times/week [37,38,41,42,44,50,55,56,54,48]	10	45%
5 times/week [35,36,43]	3	14%
**Training Intensity**		
13/20 RPE [44]	1	5%
**N/A** [35–39,41–43,45–47,49–53,55,56,40,54,48]	21	95%
**Training Load Monitoring**		
RPE [44]	1	5%
**N/A** [35–39,41–43,45–47,49–53,55,56,40,54,48]	21	95%
**Adherence Rate%** (If there was more than one intervention group, we used the mean value of the groups)		
**IG**		
51–55% [50]	1	4.5%
76-80% [40]	1	4.5%
81–85% [51,52]	2	9%
86–90% [38,41,44,45,47,49,53,55,48]	9	41%
91–95% [36,37,56,54]	4	18%
96–100% [35,39,42,43,46]	5	23%
**CG**		
56–60% [52]	1	4.5%
71-75% [40]	1	4.5%
76–80% [37,50]	2	9%
81–85% [41,51,53,48]	4	18%
86–90% [36,38,45,49]	4	18%
91–95% [42,47,55,56,54]	5	23%
96–100% [35,39,43,44,46]	5	23%

**Publication Year**		
2012 [50]	1	4.5%
2014 [56]	1	4.5%
2016 [52]	1	4.5%
2018 [41,51]	2	9%
2019 [53,55]	2	9%
2020 [37,47]	2	9%
2021 [36,38,49,48]	4	18%
2022 [35,39,44–46]	5	23%
2023 [42]	1	4.5%
2024 [43,40,54]	3	14%
**Aerobic Exercise (AE)** (Total 46 studies)		
**Mean Age of Participants** (We used only the mean value and ignored the standard deviation)		
56–60 [57]	1	2%
61–65 [58,59,60,61]	4	9%
66–70 [62,63,64,65,66,67,68,69,70]	9	19.5%
71–75 [71,72,73,74,75,76,77,78,79,80,81,82,83]	13	28%
76–80 [84,85,86,87,88,89,90,91,92,93,94]	11	24%
81–85 [95,96,97,98,99]	5	11%
86–90 [100,101,102]	3	6.5%
**Gender**		
**Female**		
28[58] 49[62] 15[63] 143[71] 86[72] 38[64] 13[73] 90[85] 86[74] 25[65] 195[75] 77[76] 39[77] 20[66] 44[67] 67[95] 13[59] 71[69] 11[100] 13[101] 33[78] 49[79] 33[60] 11[80] 46[87] 11[88] 14[89] 32[90] 24[91] 79[96] 74[92] 27[97] 38[102] 19[57] 30[93] 11[70] 93[81] 126[99] 32[94] 169[82] 77[83]	2151	55%
**Male**		
24[58] 57[62] 14[63] 219[71] 17[64] 8[73] 30[85] 97[74] 8[65] 220[75] 37[77] 40[66] 60[95] 40[68] 17[59] 19[69] 50[100] 12[101] 17[78] 11[79] 4[60] 7[80] 39[87] 9[88] 8[89] 20[90] 18[96] 57[92] 4[97] 5[102] 21[57] 30[93] 29[70] 52[81] 63[99] 46[94] 127[82] 31[83]	1567	40%
**N/A**		
69[84] 59[61] 48[86] 24[98]	200	5%
**Total**	3918	
**Sample Size**		
16–20 [80,88]	2	4.3%
21–25 [73,89,91,98,101]	5	11%
26–30 [63,59]	2	4.3%
31–35 [65,97]	2	4.3%
36–40 [57,60,68,70]	4	8.7%
41–45 [67,102]	2	4.3%
46–50 [78,86]	2	4.3%
51–55 [58,64,90]	3	6.5%
56–60 [61,66,79,93]	4	8.7%
61–65 [100]	1	2.2%
66–70 [84]	1	2.2%
76–80 [76,77,94]	3	6.5%
81–85 [87]	1	2.2%
86–90 [69,72]	2	4.3%
96–100 [96]	1	2.2%
106–110 [62,83]	2	4.3%
116–120 [85]	1	2.2%
126–130 [95]	1	2.2%
131–135 [92]	1	2.2%
141–145 [81]	1	2.2%
181–185 [74]	1	2.2%
186–190 [99]	1	2.2%
295-300 [82]	1	2.2%
361–365 [71]	1	2.2%

411–415 [75]	1	2.2%
**Diseases Categories** (As defined by the included studies)		
MCI [57,59–61,66–69,72,76,79–81,84–86,91,102,82,83]	20	43%
Dementia [80,87,88,92,93,96,97,99,100]	9	20%
AD [70,77,78,88–90,94,98]	8	17%
aMCI [58,63–65,74]	5	11%
Healthy/older inactive senior [62,95]	2	4%
MCI due to stroke [71]	1	2%
CI due to vascular [73]	1	2%
Older adults with memory complaints [75]	1	2%
Mild to moderate CI [101]	1	2%
**Diagnosis Instruments**		
MMSE [57,58,61,64,66,68,71,72,76,78,87–91,93–96,98,100–102,82]	24	52%
MoCA [57,65,69,72,74,76,79,85]	8	17%
CDR [58,77,88,89,94,95,82]	7	15%
DSM [87,90,99]	3	6.5%
Diagnosed by a specialist [57,59,84]	3	6.5%
NINDS-IAREN [78,88]	2	4%
Petersen criteria [58,63]	2	4%
GDS [66,67]	2	4%
Patients' records [97,83]	2	4%
NIA-AA [89]	1	2%
NINCDS-ARDRA [88]	1	2%
Mayo Clinic diagnostic criteria [60]	1	2%
Spanish society of geriatrics and gerontology [86]	1	2%
ICD-10 [92]	1	2%
NPI [92]	1	2%
**N/A** [62,70,73,75,80,81]	6	13%
**Cognitive Impairment Level** (We used only the mean value and ignored the standard deviation)		
**MMSE**		
≤ 9 Sever cognitive impairment [102]	1	2%
10–20 Moderate cognitive impairment [78,61,86,87,88,89,90,91,92,97,98]	11	24%
21–26 Mild cognitive impairment [64,75,76,77,66,67,95,68,100,101]	10	22%
27–30 Normal cognition [58,62,63,71–73,84,82]	8	17%
**MoCA**		
18–25 Mild cognitive impairment [72,73,85,74,65,76,59,69,100,79,60,80,61,83]	14	30%
**N/A** [70,81,94,99,57,93]	6	13%
**Mean Education Years of Participants** (Only included the studies that provided education background data in Mean ± SD format)		
0–5 [67,84]	2	4%
6–10 [88–90,95]	4	9%
11–15 [62,64,65,75,77,94]	6	13%
16–20 [58,82]	2	4%
**N/A** [57,59–61,63,66,68–74,76,78–81,85–87,91–93,96–102,83]	32	70%
**Training Period**		
4 weeks [100]	1	2%
6 weeks [57,61,79,96,97,102]	6	13%
8 weeks [59,65,67,70,81]	5	11%
9 weeks [90]	1	2.1%
10 weeks [101]	1	2.1%
12 weeks [60,68,69,78,84,86,87,91–93,95]	11	24%
16 weeks [64,85,88]	3	6.5%
24 weeks [62,63,66,72,73,76,89,94,98,83]	10	22%
26 weeks [75,77]	2	4%

48 weeks [58,74,80,82]	4	9%
60 weeks [99]	1	2.1%
72 weeks [71]	1	2.1%
**Duration of Training Session**		
15-mins/time [99]	1	2%
25-mins/time [60]	1	2%
30-mins/time [66,71,77,86,88,90–92,96,98,100–102,83]	14	30%
35-mins/time [69]	1	2%
40-mins/time [58,64,68,78,97]	5	11%
45-mins/time [70,74,82]	3	6.5%
50-mins/time [59–61,93,83]	5	11%
60-mins/time [57,63,67,72,73,75,76,79–81,84,85,87,89,94,95]	16	35%
65-mins/time [65]	1	2%
75-mins/time [60]	1	2%
120-mins/time [62]	1	2%
150-mins/time [60]	1	2%
**Training Frequency**		
1 time/week [60,62,80,95]	4	9%
1.5 time/week [81]	1	2%
2 times/week [72,75,76,79,84,87–90,97]	10	22%
3 times/week [57,60,64,65,67,68,70,73,74,78,80,85,86,93,94,102,83]	17	37%
4 times/week [63,98,82]	3	6.5%
5 times/week [58,61,66,69,77,91,92,96,100,83]	10	22%
7 times/week [59,71,99,101]	4	9%
**Training Intensity**		
40% of HRmax [86]	1	2%
60% of HRmax [84,66,86]	3	6.5%
60–70% of HRmax [59,70,83]	3	6.5%
70% of HRmax [78,90]	2	4%
60–80% of HRmax [57]	1	2%
70–80% of HRmax [76]	1	2%
75% of HRmax [67]	1	2%
75–90% of HRmax [58]	1	2%
80% combined 60% of HRmax [90]	1	2%
50–75% of HRR [94]	1	2%
60–70% of HRR [73,77]	2	4%
70% of HRR [75]	1	2%
70–75% of HRR [64]	1	2%
70–80 % HRR [72,82]	2	4%
75–85% of HRR [63,68]	2	4%
4–5/10 RPE [81]	1	2%
5–6/10 RPE [100]	1	2%
9–13/20 RPE [69]	1	2%
13/20 RPE [74]	1	2%
9–15/20 RPE [94]	1	2%
11–14/20 RPE [59,61]	2	4%
12–14/20 RPE [85,92,83]	3	6.5%
13–15/20 RPE [76,67,95]	3	6.5%
15/20 RPE [75]	1	2%
15–17/20 RPE [71]	1	2%
75% of 1RM [64]	1	2%
7 RM [72]	1	2%
13–15RM [93]	1	2%
40–60% of VO_2max [88]	1	2%
23% of VO_2peak [91]	1	2%
Personal perception of maximum exertion [89]	1	2%
N/A [62,65,101,79,60,80,87,96,97,98,102,99]	12	26%
**Training Load Monitoring**		
HR monitor [57–59,63,64,66–68,70,72,73,75–78,84,86,90,94,82,83]	21	46%

RPE [59,61,67,69,71,74–76,81,85,92,94,95,100,83]	15	33%
**N/A** [60,62,65,79,80,87–89,91,93,96–99,101,102]	16	35%
**Adherence Rate%** (If there was more than one intervention group, we used the mean value of the groups)		
51–55% [73]	1	2%
56–60% [89]	1	2%
61–65% [86]	1	2%
66–70% [95,99]	2	4%
71–75% [60]	1	2%
76–80% [67,85,101,82]	4	9%
81–85% [57,63–65,97]	5	11%
86–90% [72,77,80,84,92,83]	6	13%
91–95% [70,71,74,75,81,90,98,100]	8	17%
96–100% [62,69,79,87,88]	5	11%
**CG**		
51–55% [99]	1	2%
56–60% [73]	1	2%
66–70% [60]	1	2%
76–80% [85,97,82]	3	6.5%
81–85% [57,64,65,98]	4	9%
86–90% [62,70,74,75,79,89,92,95,100,83]	10	22%
91–95% [71,77,84,101]	4	9%
96–100% [63,67,69,72,80,81,86–88,90]	10	22%
**N/A** [58,76,66,68,59,78,61,91,96,102,93,94]	12	26%
**Publication Year**		
2005 [102]	1	2%
2008 [87]	1	2%
2009 [96]	1	2%
2010 [63]	1	2%
2011 [86,98]	2	4%
2013 [72,88,92]	3	6.5%
2014 [66]	1	2%
2015 [78,95]	2	4%
2016 [70,91,99]	3	6.5%
2017 [67,68,77]	3	6.5%
2018 [57,62,73,79,84]	5	11%
2019 [71,64,85]	3	6.5%
2020 [65,76,59,100,80,90]	6	13%
2021 [58,74,75,81,94,101]	6	13%
2022 [60,61]	2	4%
2023 [89,93]	2	4%
2024 [69,97]	2	4%
2025 [82,83]	2	4%
**Resistance Exercise (RE)** (Total 13 studies)		
**Mean Age of Participants** (We used only the mean value and ignored the standard deviation)		
61–65 [103]	1	8%
66–70 [104–107,108]	5	38%
71–75 [109–111]	3	23%
76–80 [112,113]	2	15.5%
81–85 [114,115]	2	15.5%
**Gender**		
**Female**		
7[103] 68[104] 32[105] 68[106] 28[107] 30[109] 30[112] 24[110] 16[113] 15[111] 29[115] 28[108]	375	60%
**Male**		
22[103] 32[104] 13[105] 43[106] 24[107] 13[109] 16[110] 6[113] 15[111] 15[115] 22[108]	221	35%

**N/A**		
30[114]	30	5%
**Total**	626	
**Sample Size**		
21–25 [113]	1	7.7%
26–30 [103,111,112,114]	4	31%
36–40 [110]	1	7.7%
41–45 [105,109,115]	3	23%
46-50 [108]	1	7.7%
51–55 [107]	1	7.7%
96–100 [104]	1	7.7%
111–115 [106]	1	7.7%
**Diseases Categories** (As defined by the included studies)		
MCI [104–107,110,112–114,108]	9	69%
CI [115]	1	7.75%
AD [111]	1	7.75%
MCI due to stroke [103]	1	7.75%
Older adults with cognitive frailty [109]	1	7.75%
**Diagnosis Instruments**		
MMSE [103,105,106,110–112,114,115]	8	62%
MoCA [105,106,112,113,108]	5	38%
CDR [109,110]	2	15%
Petersen criteria [104]	1	7.7%
NINCDS-ARDRA [111]	1	7.7%
DSM [113]	1	7.7%
MRI [111]	1	7.7%
Laboratory testing [111]	1	7.7%
**N/A** [107]	1	7.7%
**Cognitive Impairment Level** (We used only the mean and ignored the standard deviation)		
**MMSE**		
10–20 Moderate cognitive impairment [115]	1	7.7%
21–26 Mild cognitive impairment [105,109–112,114,108]	7	54%
27–30 Normal cognition [103,104]	2	15%
**MoCA**		
10–17 Moderate cognitive impairment [112]	1	7.7%
18–25 Mild cognitive impairment [105–107,113]	4	31%
**Mean Education Years of Participants** (Only included the studies that provided education background data in Mean ± SD format)		
6–10 [105,109]	2	15.5%
11–15 [104,111]	2	15.5%
**N/A** [103,106,107,110,112–115,108]	9	69%
**Training Period**		
6 weeks [115]	1	7.7%
8 weeks [110]	1	7.7%
12 weeks [103,105,107,111–114,108]	8	62%
16 weeks [109]	1	7.7%
24 weeks [104,106]	2	15%
**Duration of Training Session**		
30-mins/time [111]	1	7.7%
40-mins/time [115]	1	7.7%
45-mins/time [114]	1	7.7%

50-mins/time [110]	1	7.7%
60-mins/time [105,106,109,112,113]	5	38%
75-mins/time [104]	1	7.7%
**N/A** [103,107,108]	3	23%
**Training Frequency**		
2 times/week [103,107,112,113,108]	5	38%
3 times/week [104–106,109–111,114,115]	8	62%
**Training Intensity**		
53–62% of HRmax [114]	1	7.7%
60–80% of HRmax [106]	1	7.7%
7/10 RPE [108]	1	7.7%
12–13/20 RPE [109,112,110]	3	23%
15–16/20 RPE [112]	1	7.7%
70–85% of 1RM [107,108]	2	15%
12RM [115]	1	7.7%
15RM [113]	1	7.7%
**N/A** [103,104,105,111]	4	31%
**Training Load Monitoring**		
HR monitor [106,114]	2	15%
RPE [109,110,112,108]	4	31%
**N/A** [103–105,107,111,113,115]	7	54%
**Adherence Rate%** (If there was more than one intervention group, we used the mean value of the groups)		
56–60% [112]	1	8%
61–65% [109]	1	8%
76–80% [113,114]	2	15%
81–85% [104,108]	2	15%
86–90% [103,107,110]	3	23%
91–95% [105]	1	8%
96–100% [106,111,115]	3	23%
**CG**		
36–40% [112]	1	8%
61–65% [107,108]	2	15%
66–70% [109]	1	8%
71–75% [114]	1	8%
86–90% [104]	1	8%
91–95% [103,105,113]	3	23%
96–100% [106,110,111,115]	4	30%
**Publication Year**		
2010 [114]	1	8%
2014 [104]	1	8%
2015 [111]	1	8%
2016 [103,105]	2	15%
2017 [112]	1	8%
2018 [109,113]	2	15%
2020 [106,110]	2	15%
2024 [107,115]	2	15%
2025 [108]	1	8%
**Multicomponent Exercise (ME)** (Total 57 studies)		
**Mean Age of Participants** (We used only the mean value and ignored the standard deviation)		
51–55 [116]	1	1.75%
61–65 [117,118]	2	3.5%
66–70 [119–122,123,124]	6	11%

71–75 [125–136,137,118]	14	24.5%
76–80 [138–150,151]	14	24.5%
81–85 [152–158,159,160,161,162,163,164,165,166]	15	26%
86–90 [167,168,169,170]	4	7%
**N/A** [171]	1	1.75%
**<u>Gender</u>**		
**Female**<br>77[125] 23[138] 49[126] 51[171] 23[139] 28[119] 10[116] 196[127] 13[128] 94[167] 87[120] 126[140] 90[152] 35[129] 37[130] 21[131] 11[132] 40[133] 38[121] 24[134] 37[141] 103[142] 41[143] 90[153] 25[154] 43[155] 11[135] 10[136] 193[144] 70[145] 27[146] 14[156] 55[147] 49[168] 40[148] 132[157] 23[150] 55[158] 79[117] 25[159] 82[160] 125[169] 141[161] 67[162] 23[163] 77[170] 101[164] 56[151] 36[165] 8[166] 53[137] 14[123] 16[124]	2994	61%
**Male**<br>22[125] 27[138] 51[126] 33[171] 27[139] 19[116] 33[127] 21[128] 54[167] 113[120] 45[140] 28[152] 25[129] 35[130] 6[131] 19[132] 12[133] 4[121] 16[134] 23[141] 66[142] 70[143] 32[153] 15[154] 26[155] 12[135] 6[136] 301[144] 16[145] 19[146] 2[156] 32[147] 14[168] 20[148] 56[157] 12[150] 22[158] 99[117] 8[159] 27[160] 45[161] 20[162] 8[163] 14[170] 33[164] 19[151] 19[165] 4[166] 51[137] 32[123] 25[124]	1738	35%
**N/A**<br>48[122] 34[149] 82[172] 30[118]	194	4%
**Total**	4926	
**<u>Sample Size</u>**		
11–15 [166]	1	1.75%
16–20 [136,156]	2	3.5%
21–25 [135]	1	1.75%
26–30 [116,119,131,132,118]	5	8.8%
31–35 [128,149,150,159,163]	5	8.8%
36–40 [134,154]	2	3.5%
41–45 [121,124]	2	3.5%
46–50 [122,138,139,146,123]	5	8.8%
51–55 [133,165]	2	3.5%
56–60 [129,141,148]	3	5.3%
61–65 [168]	1	1.75%
66–70 [155]	1	1.75%
71–75 [130,151]	2	3.5%
76–80 [158]	1	1.75%
81–85 [171,172]	2	3.5%
86–90 [145,147,162]	3	5.3%
91–95 [170]	1	1.75%
96–100 [125,126]	2	3.5%
101–105 [137]	1	1.75%
106–110 [160]	1	1.75%
111–115 [143]	1	1.75%
116–120 [152]	1	1.75%
121–125 [153]	1	1.75%
131–135 [164]	1	1.75%
146–150 [167]	1	1.75%
166–170 [142,169]	2	3.5%
171–175 [140]	1	1.75%
176–180 [117]	1	1.75%
186–190 [157,161]	2	3.5%
201–205 [120]	1	1.75%
226–230 [127]	1	1.75%
491–495 [144]	1	1.75%
**<u>Diseases Categories</u>** (As defined by the included studies)		
Dementia [135,143,144,153,155–157,167,168,160,169,161,162,163,170,151,165,166,118]	19	33%
AD [120,130,132,134,136,140,141,146–150,154,159,170,172,164]	17	30%
MCI [119,121,125–127,129,131,133,139,142,146,147,171]	13	23%

CI [145,152,158]	3	5%
MCI due to stroke [116]	1	1.75%
MCI due to PD [128,123]	2	3.5%
CI due to T2D [122]	1	1.75%
CI due to Vascular [117]	1	1.75%
aMCI [138]	1	1.75%
Mild vascular cognitive impairment[137]	1	1.75%
MCI or mild AD[124]	1	1.75%
**Diagnosis Instruments**		
MMSE [116,120,122,130,132,135,136,138,140–142,152–155,167,168,159,160,161,162,163,170,164,151,123]	26	46%
DSM [132,134,141,146,155,157,163,170,166,118]	10	17.5%
Diagnosed by a specialist [125,141,143,155,159,160,163]	7	12%
CDR [126,131,139,146,148,150,169]	7	12%
NINCDS-ARDRA [120,141,149,172,164]	5	9%
MoCA [119,127,148,171,123]	5	9%
NIA-AA [127,147]	2	3.5%
Petersen criteria [126,139]	2	3.5%
ACE [141,167]	2	3.5%
sMMSE [129,156]	2	3.5%
CERAD [153]	1	1.75%
GDS [157]	1	1.75%
Biomarkers Neuroimaging Clinical evaluation[124]	1	1.75%
**N/A** [117,121,128,133,144,145,158,165,137]	9	16%
**Cognitive Impairment Level** (We used only the mean value and ignored the standard deviation)		
**MMSE**		
≤ 9 Sever cognitive impairment [164]	1	1.75%
10–20 Moderate cognitive impairment [117,122,135,136,140,145–147,149,150,155,158,168,159,160,169,161,162,163,170,172,123]	22	39%
21–26 Mild cognitive impairment [116,120,121,125,126,128,130–134,136,138–143,147,152–154,167,171,124]	25	44%
27–30 Normal cognition [146]	1	1.75%
**MoCA**		
10–17 Moderate cognitive impairment [148,156,157]	3	5%
18–25 Mild cognitive impairment [116,118,120,126,127,171,136,123]	8	14%
26–30 Normal cognition[122]	1	1.75%
**N/A** [128,144,151,165,166,137]	6	11%
**Mean Education Years of Participants** (Only included the studies that provided education background data in Mean ± SD format)		
0–5 [117,143]	2	4%
6–10 [120,124,125,127,130,133,138,139,147,159,136]	11	19%
11–15 [140,146,153,123]	4	7%
**N/A** [116,118,119,121,126,128,129,131,132,134,135,141,142,144,145,148–150,152,154–158,167,168,171,160,169,161,162,163,170,172,164,151,165,166,122,137]	40	70%
**Training Period**		
4 weeks [127]	1	1.75%
6 weeks [116,121]	2	3.5%
8 weeks [118,126,131,149]	4	7%
9 weeks [160]	1	1.75%
12 weeks [117,120,124,129,134,135,140–143,146,148,152,153,157,158,168,169,151,165,166,122,137]	23	40%
15 weeks [163]	1	1.75%
16 weeks [119,133,150,161,172]	5	9%
20 weeks [128]	1	1.75%
24 weeks [130,132,138,147,154–156,171,159,162,170,136,123]	13	23%
48 weeks [125,139,144,145,164]	5	9%
49 weeks [167]	1	1.75%

**Duration of Training Session**		
15-mins/time [165]	1	1.75%
30-mins/time [118,124,131,140,143,155,157,171,160,172,151,167]	12	21%
35-mins/time [121]	1	1.75%
40-mins/time [129]	1	1.75%
45-mins/time [121,140–142,149,161,162,165,137]	9	16%
50-mins/time [117]	1	1.75%
60-mins/time [116,118–120,126,127,130,132,146,148,150,158,167,168,159,169,163,170,164,166,136,122]	22	39%
75-mins/time [135]	1	1.75%
80-mins/time [128]	1	1.75%
90-mins/time [125,138,139,144,147,156]	6	10.5%
105-mins/time [123]	1	1.75%
120-mins/time [153]	1	1.75%
**N/A** [133,134,145,152,154]	5	9%
**Training Frequency**		
0.5 time/week [125,148]	2	3.5%
1 time/week [165,166,123]	3	5%
2 times/week [124,130,132,134,138,139,141,144–146,153,156,158,167,168,169,170,164,166]	19	33%
2.5 times/week [161]	1	1.7%
3 times/week [116–121,126,135,140,142,143,147,149,150,155,162,163,151,165,166,136,122,137]	23	40%
4 times/week [160]	1	1.7%
5 times/week [121,129,131,140,154,157,171,159,172]	9	16%
6 times/week [127]	1	1.7%
7 times/week [128,133,152]	3	5%
**Training Intensity**		
40–60% of HRmax [159]	1	1.75%
50–85% of HRmax [160]	1	1.75%
57–89% of HRmax [155]	1	1.75%
60% of HRmax [138,125,139]	3	5%
64–76% of HRmax [140]	1	1.75%
60–75% of HRmax [132]	1	1.75%
70% of HRmax [147]	1	1.75%
70–80% of HRmax [119]	1	1.75%
80% of HRmax [146]	1	1.75%
80–85% of HRmax [142]	1	1.75%
35–45% of HRR [121]	1	1.75%
60–70% of HRR [130]	1	1.75%
75–85% of HRR [121]	1	1.75%
12–15/20 RPE [160]	1	1.75%
4–5/10 RPE [171]	1	1.75%
4-6/10 RPE	1	1.75%
11–13/20 RPE [159]	1	1.75%
12–14/20 RPE [167]	1	1.75%
13–15/20 RPE [117]	1	1.75%
12–15/20 RPE [136]	1	1.75%
9–16/20 RPE [155]	1	1.75%
40–70% of 1RM [130]	1	1.75%
50–69% of 1RM [140]	1	1.75%
60–80% of 1 RM [134]	1	1.75%
70–80% of 1 RM [153]	1	1.75%
85% of 1RM [147]	1	1.75%
12RM [160]	1	1.75%
15RM [158]	1	1.75%
13–15 RM [161]	1	1.75%
70% of VO_2max [146]	1	1.75%
**N/A** 124,118,116,126,127,152,128,129,131,132,120,133,141,143,154,135,144,145,146,168,148,157,149,150,162,163,170 172,164,151,165,166,122,123	34	60%

**<u>Training Load Monitoring</u>**		
HR monitor [119–121,125,130,132,138–140,142,146,147,155,159,160]	15	26%
RPE [117,155,167,171,159,160,136,137]	8	14%
**N/A** [116,118,124,126–129,131,133–135,141,143–145,148–150,152–154,156–158,168,169,161,162,163,170,172,164,151,165,166,122,123]	37	65%
**<u>Adherence Rate%</u>** (If there was more than one intervention group, we used the mean value of the groups)		
51–55% [157]	1	1.8%
56–60% [130,154]	2	3.5%
71–75% [145,162]	2	3.5%
76–80% [117,131,150,156,168,163]	6	10.5%
81–85% [134,155,167,170,164,136,122,123]	8	14%
86–90% [125,132,152,159,160,169,161,165,137]	9	16%
91–95% [116,121,124,126,127,138,142–144,171]	10	17.5%
96–100% [119,120,128,129,133,139,140,147,148,153,158,166]	12	21%
**CG**		
56-60 [130]	1	1.8%
66–70% [145,155]	2	3.5%
71–75% [120,157,162]	3	5%
76–80% [131,167,136,122]	4	7%
81–85% [143,168,117,163,164]	5	9%
86–90% [121,125,132,144,154,160,170]	7	12.2
91–95% [119,134,139,150,152,153,171,169,161,165,123]	11	19.3%
96–100% [116,124,126–129,133,138,140,142,147,148,156,158,159,166,137]	17	30%
**N/A** [118,135,141,146,149,172,151]	7	12.2%
**<u>Publication Year</u>**		
2006 [151]	1	1.75%
2007 [164]	1	1.75%
2008 [135]	1	1.75%
2010 [163]	1	1.75%
2011 [172]	1	1.75%
2012 [125,133,139,153,154]	5	9%
2013 [138,145]	2	3.5%
2014 [160]	1	1.75%
2015 [169]	1	1.75%
2016 [119,159]	2	3.5%
2017 [116,118,131,143,161,170]	6	10.5%
2018 [128,144,162]	3	6%
2019 [117,132,134,146,147,158]	6	10.5%
2020 [155]	1	1.75%
2021 [127,149,150,171,165,166]	6	10.5%
2022 [124,126,152,157,167,168]	6	10.5%
2023 [120,129,140,142,156]	5	9%
2024 [121,130,141,148,136,122,137]	7	12%
2025 [123]	1	1.75%

Notes:

1. When training time duration and frequency were given as a rang, we used the maximum value.
2. The percentage of some domains is above 100% because they included multiple items.

Abbreviations:

RCT: Randomized controlled trial
EG: Experiment group
CG: Control group
CI: Cognitive Impairment
MCI: Mild Cognitive Impairment
aMCI: Amnestic Mild Cognitive Impairment
AD: Alzheimer’s disease

T2D: Type 2 diabetes
PD: Parkinson's disease
HR: Heart rate
HRR: Heart Rate Reserve
MMSE: Mini-Mental State Examination
MoCA: Montreal Cognitive Assessment
CDR: Clinical Dementia Rating
NIA-AA: National Institute on Ageing and Alzheimer's Association guidelines
GDS: Global Deterioration Scale
DSM: Diagnostic and Statistical Manual
NINCDS-ADRDA: Neurological and Communicative Disorders and Stroke-Alzheimer's Disease and Related Disorders Association
NINDS-IAREN: National Institute of Neurological Disorders and Stroke- International Association for Education in Neurosciences
CERAD: Consortium to Establish a Registry for Alzheimer's Disease
ICD-10: 10th revision of the International Classification of Diseases
ACE: Mini Addenbrooke's Cognitive Examination
NPI: Neuropsychiatric Inventory
1RM: One Repetition Maximum

References

1. Kropacova S, Mitterova K, Klobusiakova P, et al. Cognitive effects of dance-movement intervention in a mixed group of seniors are not dependent on hippocampal atrophy. *J Neural Transm*. 2019;126(11):1455-1463. doi:10.1007/s00702-019-02068-y

2. Esmail A, Vrinceanu T, Lussier M, et al. Effects of Dance/Movement Training vs. Aerobic Exercise Training on cognition, physical fitness and quality of life in older adults: A randomized controlled trial. *J Bodyw Mov Ther*. 2020;24(1):212-220. doi:10.1016/j.jbmt.2019.05.004

3. Qi M, Zhu Y, Zhang L, Wu T, Wang J. The effect of aerobic dance intervention on brain spontaneous activity in older adults with mild cognitive impairment: A resting-state functional MRI study. *Exp Ther Med*. Published online 2018:715-722. doi:10.3892/etm.2018.7006

4. Lazarou I, Parastatidis T, Tsolaki A, et al. International Ballroom Dancing Against Neurodegeneration: A Randomized Controlled Trial in Greek Community-Dwelling Elders With Mild Cognitive impairment. *Am J Alzheimers Dis Other Demen*. 2017;32(8):489-499. doi:10.1177/1533317517725813

5. Franco MR, Sherrington C, Tiedemann A, et al. Effect of Senior Dance (DanSE) on Fall Risk Factors in Older Adults: A Randomized Controlled Trial. *Phys Ther*. 2020;100(4):600-608. doi:10.1093/ptj/pzz187

6. Zhu Y, Wu H, Qi M, et al. Effects of a specially designed aerobic dance routine on mild cognitive impairment. *Clin Interv Aging*. 2018;13:1691-1700. doi:10.2147/CIA.S163067

7. Thiel U, Stiebler M, Labott BK, et al. DiADEM—Dance against Dementia—Effect of a Six-Month Dance Intervention on Physical Fitness in Older Adults with Mild Cognitive Impairment: A Randomized, Controlled Trial. *J Pers Med*. 2024;14(8). doi:10.3390/jpm14080888

8. Bisbe M, Fuente-Vidal A, López E, et al. Comparative Cognitive Effects of Choreographed Exercise and Multimodal Physical Therapy in Older Adults with Amnestic Mild Cognitive Impairment: Randomized Clinical Trial. *Journal of Alzheimer's Disease*. 2020;73(2):769-783. doi:10.3233/JAD-190552

9. Song D, Yu D, Liu T, Wang J. Effect of an Aerobic Dancing Program on Sleep Quality for Older Adults With Mild Cognitive Impairment and Poor Sleep: A Randomized Controlled Trial. *J Am Med Dir Assoc*. 2024;25(3):494-499. doi:10.1016/j.jamda.2023.09.020

10. Zhu Y, Gao Y, Guo C, et al. Effect of 3-Month Aerobic Dance on Hippocampal Volume and Cognition in Elderly People With Amnestic Mild Cognitive Impairment: A Randomized Controlled Trial. *Front Aging Neurosci*. 2022;14(March):1-10. doi:10.3389/fnagi.2022.771413

11. Sánchez-Alcalá M, Aibar-Almazán A, Carcelén-Fraile M del C, et al. Effects of Dance-Based Aerobic Training on Frailty and Cognitive Function in Older Adults with Mild Cognitive Impairment: A Randomized Controlled Trial. *Diagnostics*. 2025;15(3):1-13. doi:10.3390/diagnostics15030351
12. Doi T, Verghese J, Makizako H, et al. Effects of Cognitive Leisure Activity on Cognition in Mild Cognitive Impairment: Results of a Randomized Controlled Trial. *J Am Med Dir Assoc*. 2017;18(8):686-691. doi:10.1016/j.jamda.2017.02.013
13. Blumen HM, Ayers E, Wang C, Ambrose AF, Jayakody O, Verghese J. Randomized Controlled Trial of Social Ballroom Dancing and Treadmill Walking: Preliminary Findings on Executive Function and Neuroplasticity From Dementia-at-Risk Older Adults. *J Aging Phys Act*. 2023;31(4):589-599. doi:10.1123/japa.2022-0176
14. Chang J, Zhu W, Zhang J, et al. The Effect of Chinese Square Dance Exercise on Cognitive Function in Older Women With Mild Cognitive Impairment: The Mediating Effect of Mood Status and Quality of Life. *Front Psychiatry*. 2021;12(July). doi:10.3389/fpsyt.2021.711079
15. Ho RTH, Fong TCT, Chan WC, et al. Psychophysiological Effects of Dance Movement Therapy and Physical Exercise on Older Adults with Mild Dementia: A Randomized Controlled Trial. *Journals of Gerontology - Series B Psychological Sciences and Social Sciences*. 2018;75(3):560-570. doi:10.1093/geronb/gby145
16. Van de Winckel A, Feys H, De Weerdt W, Dom R. Cognitive and behavioural effects of music-based exercises in patients with dementia. *Clin Rehabil*. 2004;18(3):253-260. doi:10.1191/0269215504cr750oa
17. Bracco L, Pinto-Carral A, Hillaert L, Mourey F. Tango-therapy vs physical exercise in older people with dementia; a randomized controlled trial. *BMC Geriatr*. 2023;23(1):1-13. doi:10.1186/s12877-023-04342-x
18. Liu CL, Cheng FY, Wei MJ, Liao YY. Effects of Exergaming-Based Tai Chi on Cognitive Function and Dual-Task Gait Performance in Older Adults With Mild Cognitive Impairment: A Randomized Control Trial. *Front Aging Neurosci*. 2022;14(March). doi:10.3389/fnagi.2022.761053
19. Uğur F, Sertel M. Wii Fit Exercise's Effects on Muscle Strength and Fear of Falling in Older Adults With Alzheimer Disease: A Randomized Controlled Trial. *J Aging Phys Act*. 2025;33(2):181-191. doi:10.1123/japa.2023-0428
20. Eggenberger P, Schumacher V, Angst M, Theill N, de Bruin ED. Does multicomponent physical exercise with simultaneous cognitive training boost cognitive performance in older adults? A 6-month randomized controlled trial with a 1-year follow-up. *Clin Interv Aging*. 2015;10:1335-1349. doi:10.2147/CIA.S87732
21. Hughes TF, Flatt JD, Fu B, Butters MA, Chang CCH, Ganguli M. Interactive video gaming compared with health education in older adults with mild cognitive impairment: A feasibility study. *Int J Geriatr Psychiatry*. 2014;29(9):890-898. doi:10.1002/gps.4075
22. Sabbagh et al. Sensor-based balance training with motion feedback in people with mild cognitive impairment. 2016;53(6):945-958. doi:10.1682/JRRD.2015.05.0089.Sensor-based
23. Padala KP, Padala PR, Malloy TR, et al. Wii-fit for improving gait and balance in an assisted living facility: A pilot study. *J Aging Res*. 2012;2012:6-11. doi:10.1155/2012/597573
24. Karssemeijer EGA, Aaronson JA, Bossers WJR, Donders R, Olde Rikkert MGM, Kessels RPC. The quest for synergy between physical exercise and cognitive stimulation via exergaming in people with dementia: A randomized controlled trial. *Alzheimers Res Ther*. 2019;11(1):1-13. doi:10.1186/s13195-018-0454-z
25. van Santen J, Dröes RM, Twisk JWR, Blanson Henkemans OA, van Straten A, Meiland FJM. Effects of Exergaming on Cognitive and Social Functioning of People with Dementia: A Randomized Controlled Trial. *J Am Med Dir Assoc*. 2020;21(12):1958-1967.e5. doi:10.1016/j.jamda.2020.04.018

26. Wu S, Ji H, Won J, Jo EA, Kim YS, Park JJ. The Effects of Exergaming on Executive and Physical Functions in Older Adults With Dementia: Randomized Controlled Trial. *J Med Internet Res*. 2023;25:1-17. doi:10.2196/39993
27. Liao YY, Chen IH, Hsu WC, Tseng HY, Wang RY. Effect of exergaming versus combined exercise on cognitive function and brain activation in frail older adults: A randomised controlled trial. *Ann Phys Rehabil Med*. 2021;64(5):101492. doi:10.1016/j.rehab.2021.101492
28. Swinnen N, Vandenbulcke M, de Bruin ED, et al. The efficacy of exergaming in people with major neurocognitive disorder residing in long-term care facilities: a pilot randomized controlled trial. *Alzheimers Res Ther*. 2021;13(1):1-13. doi:10.1186/s13195-021-00806-7
29. Zheng J, Yu P, Chen X. An Evaluation of the Effects of Active Game Play on Cognition, Quality of Life and Depression for Older People with Dementia. *Clin Gerontol*. 2022;45(4):1034-1043. doi:10.1080/07317115.2021.1980170
30. Kashyap M, Rai NK, Singh R, et al. Effect of Early Yoga Practice on Post Stroke Cognitive Impairment. 2022;22(4):2019. doi:10.4103/aian.AIAN
31. Khanthong P, Sriyakul K, Dechakhamphu A, Krajarng A, Kamalashiran C, Tungsukruthai P. Traditional Thai exercise (Ruesi Dadton) for improving motor and cognitive functions in mild cognitive impairment: a randomized controlled trial. *J Exerc Rehabil*. 2021;17(5):331-338. doi:10.12965/JER.2142542.271
32. Grzenda A, Siddarth P, Milillo MM, Aguilar-Faustino Y, Khalsa DS, Lavretsky H. Cognitive and immunological effects of yoga compared to memory training in older women at risk for alzheimer's disease. *Transl Psychiatry*. 2024;14(1):1-11. doi:10.1038/s41398-024-02807-0
33. Eyre HA, Siddarth P, Acevedo B, et al. A randomized controlled trial of Kundalini yoga in mild cognitive impairment. *Int Psychogeriatr*. 2017;29(4):557-567. doi:10.1017/S1041610216002155
34. Tremont G, Davis J, Ott BR, et al. Feasibility of a Yoga Intervention for Individuals with Mild Cognitive Impairment: A Randomized Controlled Trial. *Journal of Integrative and Complementary Medicine*. 2022;28(3):250-260. doi:10.1089/jicm.2021.0204
35. Li K, Yu H, Kortas JA, Lin X, Lipowski M. The effect of 12 weeks of Baduanjin exercise on cognitive function, lower limb balance and quality of life of the elderly with mild cognitive impairment: a randomized controlled trial. *Gazzetta Medica Italiana Archivio per le Scienze Mediche*. 2022;181(11):811-823. doi:10.23736/S0393-3660.22.04802-1
36. Su H, Wang H, Meng L, Bush E. The effects of Baduanjin exercise on the subjective memory complaint of older adults: A randomized controlled trial. *Medicine (United States)*. 2021;100(30):E25442. doi:10.1097/MD.0000000000025442
37. Zheng G, Zheng Y, Xiong Z, Ye B. Effect of Baduanjin exercise on cognitive function in patients with post-stroke cognitive impairment: a randomized controlled trial. *Clin Rehabil*. 2020;34(8):1028-1039. doi:10.1177/0269215520930256
38. Zheng G, Ye B, Xia R, et al. Traditional Chinese Mind-Body Exercise Baduanjin Modulate Gray Matter and Cognitive Function in Older Adults with Mild Cognitive Impairment: A Brain Imaging Study. *Brain Plasticity*. 2021;7(2):131-142. doi:10.3233/bpl-210121
39. Luo SS, Chen L, Wang GB, Wang YG, Su XY. Effects of long-term Wuqinxi exercise on working memory in older adults with mild cognitive impairment. *Eur Geriatr Med*. 2022;13(6):1327-1333. doi:10.1007/s41999-022-00709-2
40. Chang CL, Lin TK, Pan CY, et al. Distinct effects of long-term Tai Chi Chuan and aerobic exercise interventions on motor and neurocognitive performance in early-stage Parkinson's disease: a randomized controlled trial. *Eur J Phys Rehabil Med*. 2024;60(4):621-633. doi:10.23736/S1973-9087.24.08166-8
41. Sungkarat S, Boripuntakul S, Kumfu S, Lord SR, Chattipakorn N. Tai Chi Improves Cognition and Plasma BDNF in Older Adults With Mild Cognitive Impairment: A Randomized Controlled Trial. *Neurorehabil Neural Repair*. 2018;32(2):142-149. doi:10.1177/1545968317753682

42. Chen Y, Qin J, Tao L, et al. Effects of Tai Chi Chuan on Cognitive Function in Adults 60 Years or Older With Type 2 Diabetes and Mild Cognitive Impairment in China: A Randomized Clinical Trial. *JAMA Netw Open*. 2023;6(4):E237004. doi:10.1001/jamanetworkopen.2023.7004
43. Lin M, Liu W, Ma C, et al. Tai Chi-Induced Exosomal LRP1 is Associated With Memory Function and Hippocampus Plasticity in aMCI Patients. *American Journal of Geriatric Psychiatry*. 2024;32(10):1215-1230. doi:10.1016/j.jagp.2024.04.012
44. Yu AP, Chin EC, Yu DJ, et al. Tai Chi versus conventional exercise for improving cognitive function in older adults: a pilot randomized controlled trial. *Sci Rep*. 2022;12(1):1-15. doi:10.1038/s41598-022-12526-5
45. Li F, Harmer P, Fitzgerald K, Winters-Stone K. A cognitively enhanced online Tai Ji Quan training intervention for community-dwelling older adults with mild cognitive impairment: A feasibility trial. *BMC Geriatr*. 2022;22(1):1-13. doi:10.1186/s12877-021-02747-0
46. Jiayuan Z, Xiang-Zi J, Li-Na M, Jin-Wei Y, Xue Y. Effects of Mindfulness-Based Tai Chi Chuan on Physical Performance and Cognitive Function among Cognitive Frailty Older Adults: A Six-Month Follow-Up of a Randomized Controlled Trial. *Journal of Prevention of Alzheimer's Disease*. 2022;9(1):104-112. doi:10.14283/jpad.2021.40
47. Canan Okuyan ED. The effectiveness of Tai Chi Chuan on fear of movement, prevention of falls, physical activity, and cognitive status in older adults with mild cognitive impairment: A randomized controlled trial. *Perspect Psychiatr Care*. 2021;57(3):1273-1281. doi:10.1111/ppc.12684
48. Hsu CY, Yeh ML, Liu YCE. Three-month Chan-Chuang qigong program improves physical performance and quality of life of patients with cognitive impairment: A randomized controlled trial. *Res Nurs Health*. 2022;45(3):327-336. doi:10.1002/nur.22219
49. Li F, Harmer P, Voit J, Chou LS. Implementing an online virtual falls prevention intervention during a public health pandemic for older adults with mild cognitive impairment: A feasibility trial. *Clin Interv Aging*. 2021;16:973-983. doi:10.2147/CIA.S306431
50. Lam LCW, Chau RCM, Wong BML, et al. A 1-Year Randomized Controlled Trial Comparing Mind Body Exercise (Tai Chi) With Stretching and Toning Exercise on Cognitive Function in Older Chinese Adults at Risk of Cognitive Decline. *J Am Med Dir Assoc*. 2012;13(6):568.e15-568.e20. doi:10.1016/j.jamda.2012.03.008
51. Liu JYW, Kwan RYC, Lai CKY, Hill KD. A simplified 10-step Tai-chi programme to enable people with dementia to improve their motor performance: a feasibility study. *Clin Rehabil*. 2018;32(12):1609-1623. doi:10.1177/0269215518786530
52. Chan AWK, Yu DSF, Choi KC, Lee DTF, Sit JWH, Chan HYL. Tai chi qigong as a means to improve night-time sleep quality among older adults with cognitive impairment: A pilot randomized controlled trial. *Clin Interv Aging*. 2016;11:1277-1286. doi:10.2147/CIA.S111927
53. Nyman SR, Ingram W, Sanders J, et al. Randomised controlled trial of the effect of tai chi on postural balance of people with dementia. *Clin Interv Aging*. 2019;14:2017-2029. doi:10.2147/CIA.S228931
54. Gao R, Greiner C, Ryuno H, Zhang X. Effects of Tai Chi on physical performance, sleep, and quality of life in older adults with mild to moderate cognitive impairment. *BMC Complement Med Ther*. 2024;24(1). doi:10.1186/s12906-024-04705-w
55. Huang N, Li W, Rong X, et al. Effects of a Modified Tai Chi Program on Older People with Mild Dementia: A Randomized Controlled Trial. *Journal of Alzheimer's Disease*. 2019;72(3):947-956. doi:10.3233/JAD-190487
56. Cheng ST, Chow PK, Song YQ, et al. Mental and physical activities delay cognitive decline in older persons with dementia. *American Journal of Geriatric Psychiatry*. 2014;22(1):63-74. doi:10.1016/j.jagp.2013.01.060
57. Amjad I, Toor H, Niazi IK, et al. Therapeutic effects of aerobic exercise on EEG parameters and higher cognitive functions in mild cognitive impairment patients. *International Journal of Neuroscience*. 2019;129(6):551-562. doi:10.1080/00207454.2018.1551894

58. Tomoto T, Liu J, Tseng BY, et al. One-Year Aerobic Exercise Reduced Carotid Arterial Stiffness and Increased Cerebral Blood Flow in Amnestic Mild Cognitive Impairment. *Journal of Alzheimer's Disease*. 2021;80(2):841-853. doi:10.3233/JAD-201456
59. Karthikeyan T. Therapeutic effects of home-based exercise of geriatrics for the management of cognitive impairment. *ES J Public Health*. 2020;1(1):1003. www.escientificlibrary.com
60. Yu DJ, Yu AP, Bernal JDK, et al. Effects of exercise intensity and frequency on improving cognitive performance in middle-aged and older adults with mild cognitive impairment: A pilot randomized controlled trial on the minimum physical activity recommendation from WHO. *Front Physiol*. 2022;13(September):1-12. doi:10.3389/fphys.2022.1021428
61. Khattak HG, Ahmad Z, Arshad H, Anwar K. Effect of aerobic exercise on cognition in elderly persons with mild cognitive impairment. *Rawal Medical Journal*. 2022;47(3):698-701. doi:10.5455/rmj.20210713072242
62. Shimada H, Lee S, Akishita M, et al. Effects of golf training on cognition in older adults: A randomised controlled trial. *J Epidemiol Community Health (1978)*. 2018;72(10):944-950. doi:10.1136/jech-2017-210052
63. Baker LD, Frank LL, Foster-Schubert K, et al. Effects of aerobic exercise on mild cognitive impairment: A controlled trial. *Arch Neurol*. 2010;67(1):71-79. doi:10.1001/archneurol.2009.307
64. Tsai CL, Pai MC, Ukropec J, Ukropcová B. Distinctive Effects of Aerobic and Resistance Exercise Modes on Neurocognitive and Biochemical Changes in Individuals with Mild Cognitive Impairment. *Curr Alzheimer Res*. 2019;16(4):316-332. doi:10.2174/1567205016666190228125429
65. Rojasavastera R, Bovonsunthonchai S, Hiengkaew V, Senanarong V. Action observation combined with gait training to improve gait and cognition in elderly with mild cognitive impairment a randomized controlled trial. *Dementia e Neuropsychologia*. 2020;14(2):118-127. doi:10.1590/1980-57642020dn14-020004
66. Wei X hong, Ji L li. Effect of handball training on cognitive ability in elderly with mild cognitive impairment. *Neurosci Lett*. 2014;566:98-101. doi:10.1016/j.neulet.2014.02.035
67. Damirchi A, Hosseini F, Babaei P. Mental Training Enhances Cognitive Function and BDNF More Than Either Physical or Combined Training in Elderly Women With MCI: A Small-Scale Study. *Am J Alzheimers Dis Other Demen*. 2018;33(1):20-29. doi:10.1177/1533317517727068
68. Kohanpour MA, Peeri M, Azarbayjani MA. The effects of aerobic exercise with lavender essence use on cognitive state and serum brain-derived neurotrophic factor levels in elderly with mild cognitive impairment. *Journal of HerbMed Pharmacology*. 2017;6(2):80-84.
69. Krootnark K, Chaikeeree N, Saengsirisuwan V, Boonsinsukh R. Effects of low-intensity home-based exercise on cognition in older persons with mild cognitive impairment: a direct comparison of aerobic versus resistance exercises using a randomized controlled trial design. *Front Med (Lausanne)*. 2024;11(June):1-11. doi:10.3389/fmed.2024.1392429
70. Abd El-Kader SM, Al-Jiffri OH. Aerobic exercise improves quality of life, psychological well-being and systemic inflammation in subjects with alzheimer's disease. *Afr Health Sci*. 2016;16(4):1045-1055. doi:10.4314/ahs.v16i4.22
71. Ihle-Hansen H, Langhammer B, Lydersen S, Gunnes M, Indredavik B, Askim T. A physical activity intervention to prevent cognitive decline after stroke: Secondary results from the life after stroke study, an 18-month randomized controlled trial. *J Rehabil Med*. 2019;51(9):646-651. doi:10.2340/16501977-2588
72. Nagamatsu LS, Chan A, Davis JC, et al. Physical activity improves verbal and spatial memory in older adults with probable mild cognitive impairment: A 6-month randomized controlled trial. *J Aging Res*. 2013;2013(Mci). doi:10.1155/2013/861893
73. Hsu CL, Best JR, Davis JC, et al. Aerobic exercise promotes executive functions and impacts functional neural activity among older adults with vascular cognitive impairment. *Br J Sports Med*. 2018;52(3):184-191. doi:10.1136/bjsports-2016-096846

74. Stuckenschneider T, Sanders ML, Devenney KE, et al. NeuroExercise: The Effect of a 12-Month Exercise Intervention on Cognition in Mild Cognitive Impairment—A Multicenter Randomized Controlled Trial. *Front Aging Neurosci*. 2021;12(January):1-12. doi:10.3389/fnagi.2020.621947
75. Makino T, Umegaki H, Ando M, et al. Effects of Aerobic, Resistance, or Combined Exercise Training among Older Adults with Subjective Memory Complaints: A Randomized Controlled Trial. *Journal of Alzheimer's Disease*. 2021;82(2):701-717. doi:10.3233/JAD-210047
76. Brydges CR, Liu-Ambrose T, Bielak AAM. Using intraindividual variability as an indicator of cognitive improvement in a physical exercise intervention of older women with mild cognitive impairment. *Neuropsychology*. 2020;34(8):825-834. doi:10.1037/neu0000638
77. Morris JK, Vidoni ED, Johnson DK, et al. Aerobic exercise for Alzheimer's disease: A randomized controlled pilot trial. *PLoS One*. 2017;12(2):1-14. doi:10.1371/journal.pone.0170547
78. Yang SY, Shan CL, Qing H, et al. The Effects of Aerobic Exercise on Cognitive Function of Alzheimer's Disease Patients. *CNS Neurol Disord Drug Targets*. 2015;14(10):1292-1297. doi:10.2174/1871527315666151111123319
79. Choi W, Lee S. Ground kayak paddling exercise improves postural balance, muscle performance, and cognitive function in older adults with mild cognitive impairment: A randomized controlled trial. *Medical Science Monitor*. 2018;24:3909-3915. doi:10.12659/MSM.908248
80. Fischbacher M, Chocano-Bedoya PO, Meyer U, et al. Safety and feasibility of a Dalcroze eurhythmics and a simple home exercise program among older adults with mild cognitive impairment (MCI) or mild dementia: The MOVE for your MIND pilot trial. *Pilot Feasibility Stud*. 2020;6(1):1-8. doi:10.1186/s40814-020-00645-7
81. L.F. Law et al. Effects of functional task exercise on everyday problem-solving ability and functional status in older adults with mild cognitive impairment—a randomised controlled trial. *Age Ageing*. 2021;51(7):1-11. doi:10.1093/ageing/afac144
82. Baker LD, Pa JA, Katula JA, et al. Effects of exercise on cognition and Alzheimer's biomarkers in a randomized controlled trial of adults with mild cognitive impairment: The EXERT study. *Alzheimer's and Dementia*. 2025;21(4):1-17. doi:10.1002/alz.14586
83. Huang X, Zhang S, Zhao X, et al. Feasibility and effects of remotely supervised aerobic training and resistance training in older adults with mild cognitive impairment: A pilot three-arm randomised controlled trial. *Gen Psychiatr*. 2025;38(2). doi:10.1136/gpsych-2024-101858
84. Donnezan et al. Effects of simultaneous aerobic and cognitive training on executive functions, cardiovascular fitness and functional abilities in older adults with mild cognitive impairment. *Ment Health Phys Act*. 2018;15(April):78-87. doi:10.1016/j.mhpa.2018.06.001
85. Song D, Yu DSF. Effects of a moderate-intensity aerobic exercise programme on the cognitive function and quality of life of community-dwelling elderly people with mild cognitive impairment: A randomised controlled trial. *Int J Nurs Stud*. 2019;93:97-105. doi:10.1016/j.ijnurstu.2019.02.019
86. Varela S, Ayán C, Cancela JM, Martín V. Effects of two different intensities of aerobic exercise on elderly people with mild cognitive impairment: A randomized pilot study. *Clin Rehabil*. 2012;26(5):442-450. doi:10.1177/0269215511425835
87. Miu D, Edin F, Szeto S, Mak Y. A randomised controlled trial on the effect of exercise on physical, cognitive and affective function in dementia subjects. *Asian Journal of Gerontology & Geriatrics*. 2008;3(1):8-16.
88. Arcoverde C, Deslandes A, Moraes H, et al. Treadmill training as an augmentation treatment for Alzheimer's disease: A pilot randomized controlled study. *Arq Neuropsiquiatr*. 2014;72(3):190-196. doi:10.1590/0004-282X20130231
89. Angiolillo A, Leccese D, Ciccotelli S, et al. Effects of Nordic walking in Alzheimer's disease: A single-blind randomized controlled clinical trial. *Heliyon*. 2023;9(5):e15865. doi:10.1016/j.heliyon.2023.e15865

90. Enette L, Vogel T, Merle S, et al. Effect of 9 weeks continuous vs. interval aerobic training on plasma BDNF levels, aerobic fitness, cognitive capacity and quality of life among seniors with mild to moderate Alzheimer's disease: A randomized controlled trial. *European Review of Aging and Physical Activity*. 2020;17(1):1-16. doi:10.1186/s11556-019-0234-1
91. Phoemsapthawee et al. The Benefit of Arm Swing Exercise on Cognitive Performance in Older Women with Mild Cognitive Impairment. *Journal of Exercise Physiology*. 2016;8(1):11-25.
92. Lowery D, Cerga-Pashoja A, Iliffe S, et al. The effect of exercise on behavioural and psychological symptoms of dementia: The EVIDEM-E randomised controlled clinical trial. *Int J Geriatr Psychiatry*. 2014;29(8):819-827. doi:10.1002/gps.4062
93. Abbas RL, Saab IM, Al-Sharif HK, Naja N, El-Khatib A. Effect of Adding Motorized Cycle Ergometer Over Exercise Training on Balance in Older Adults with Dementia: A Randomized Controlled Trial. *Exp Aging Res*. 2023;49(2):100-111. doi:10.1080/0361073X.2022.2046947
94. Yu F, Salisbury D, Mathiason MA. Inter-individual differences in the responses to aerobic exercise in Alzheimer's disease: Findings from the FIT-AD trial. *J Sport Health Sci*. 2021;10(1):65-72. doi:10.1016/j.jshs.2020.05.007
95. Nakatsuka M, Nakamura K, Hamanosono R, et al. A Cluster Randomized Controlled Trial of Nonpharmacological Interventions for Old-Old Subjects with a Clinical Dementia Rating of 0.5: The Kurihara Project. *Dement Geriatr Cogn Dis Extra*. 2015;5(2):221-232. doi:10.1159/000380816
96. Eggermont LHP, Swaab DF, Hol EM, Scherder EJA. Walking the line: A randomised trial on the effects of a short term walking programme on cognition in dementia. *J Neurol Neurosurg Psychiatry*. 2009;80(7):802-804. doi:10.1136/jnnp.2008.158444
97. Guzel I, Can F. The effects of different exercise types on cognitive and physical functions in dementia patients: A randomized comparative study. *Arch Gerontol Geriatr*. 2024;119(18):105321. doi:10.1016/j.archger.2023.105321
98. Venturelli M, Scarsini R, Schena F. Six-month walking program changes cognitive and ADL performance in patients with Alzheimer. *Am J Alzheimers Dis Other Demen*. 2011;26(5):381-388. doi:10.1177/1533317511418956
99. Cancela JM, Ayán C, Varela S, Seijo M. Effects of a long-term aerobic exercise intervention on institutionalized patients with dementia. *J Sci Med Sport*. 2016;19(4):293-298. doi:10.1016/j.jsams.2015.05.007
100. Liu IT, Lee WJ, Lin SY, Chang ST, Kao CL, Cheng YY. Therapeutic Effects of Exercise Training on Elderly Patients With Dementia: A Randomized Controlled Trial. *Arch Phys Med Rehabil*. 2020;101(5):762-769. doi:10.1016/j.apmr.2020.01.012
101. Dillon K, Prapavessis H. REducing SEDENTary behavior among mild to moderate cognitively impaired assisted living residents: A pilot randomized controlled trial (RESEDENT study). *J Aging Phys Act*. 2021;29(1):27-35. doi:10.1123/JAPA.2019-0440
102. Scherder EJA, Van Paasschen J, Deijen JB, et al. Physical activity and executive functions in the elderly with mild cognitive impairment. *Aging Ment Health*. 2005;9(3):272-280. doi:10.1080/13607860500089930
103. Fernandez-Gonzalo R, Fernandez-Gonzalo S, Turon M, Prieto C, Tesch PA, García-Carreira MDC. Muscle, functional and cognitive adaptations after flywheel resistance training in stroke patients: A pilot randomized controlled trial. *J Neuroeng Rehabil*. 2016;13(1):1-11. doi:10.1186/s12984-016-0144-7
104. Singh et al. The Study of Mental and Resistance Training (SMART) Study-Resistance Training and/or Cognitive Training in Mild Cognitive Impairment: A Randomized, Double-Blind, Double-Sham Controlled Trial. *J Am Med Dir Assoc*. 2014;15(12):873-880. doi:10.1016/j.jamda.2014.09.010
105. Lv J, Liu Y. Effects of momentum-based dumbbell training on motor control in older adults with mild cognitive impairment. *Chinese Journal of Rehabilitation Medicine*. 2019;34(5):544-550. doi:10.3969/j.issn.1001-1242.2019.05.009

106. Wang L, Wu B, Tao H, et al. Effects and mediating mechanisms of a structured limbs-exercise program on general cognitive function in older adults with mild cognitive impairment: A randomized controlled trial. *Int J Nurs Stud*. 2020;110:103706. doi:10.1016/j.ijnurstu.2020.103706
107. Vints WAJ, Gökçe E, Šeikinaitė J, et al. Resistance training's impact on blood biomarkers and cognitive function in older adults with low and high risk of mild cognitive impairment: a randomized controlled trial. *European Review of Aging and Physical Activity*. 2024;21(1):1-15. doi:10.1186/s11556-024-00344-9
108. Kušleikienė S, Ziv G, Vints WAJ, et al. Cognitive gains and cortical thickness changes after 12 weeks of resistance training in older adults with low and high risk of mild cognitive impairment: Findings from a randomized controlled trial. *Brain Res Bull*. 2025;222(September 2024). doi:10.1016/j.brainresbull.2025.111249
109. Yoon DH, Lee JY, Song W. Effects of Resistance Exercise Training on Cognitive Function and Physical Performance in Cognitive Frailty: A Randomized Controlled Trial. *Journal of Nutrition, Health and Aging*. 2018;22(8):944-951. doi:10.1007/s12603-018-1090-9
110. Lee DW, Yoon DH, Lee JY, Panday SB, Park J, Song W. Effects of High-Speed Power Training on Neuromuscular and Gait Functions in Frail Elderly with Mild Cognitive Impairment Despite Blunted Executive Functions: A Randomized Controlled Trial. *Journal of Frailty and Aging*. 2020;9(3):179-184. doi:10.14283/jfa.2020.23
111. Holthoff VA, Marschner K, Scharf M, et al. Effects of physical activity training in patients with alzheimer's dementia: Results of a pilot RCT study. *PLoS One*. 2015;10(4):1-11. doi:10.1371/journal.pone.0121478
112. Yoon DH, Kang D, Kim HJ, Kim JS, Song HS, Song W. Effect of elastic band-based high-speed power training on cognitive function, physical performance and muscle strength in older women with mild cognitive impairment. *Geriatr Gerontol Int*. 2017;17(5):765-772. doi:10.1111/ggi.12784
113. Hong SG, Kim JH, Jun TW. Effects of 12-week resistance exercise on electroencephalogram patterns and cognitive function in the elderly with mild cognitive impairment: A randomized controlled trial. *Clinical Journal of Sport Medicine*. 2018;28(6):500-508. doi:10.1097/JSM.0000000000000476
114. Venturelli M, Lanza M, Muti E, Schena F. Positive effects of physical training in activity of daily living-dependent older adults. *Exp Aging Res*. 2010;36(2):190-205. doi:10.1080/03610731003613771
115. Baek JE, Hyeon SJ, Kim M, Cho HY, Hahm SC. Effects of dual-task resistance exercise on cognition, mood, depression, functional fitness, and activities of daily living in older adults with cognitive impairment: a single-blinded, randomized controlled trial. *BMC Geriatr*. 2024;24(1):1-12. doi:10.1186/s12877-024-04942-1
116. Kim J, Yim J. Effects of an exercise protocol for improving handgrip strength and walking speed on cognitive function in patients with chronic stroke. *Medical Science Monitor*. 2017;23:5402-5409. doi:10.12659/MSM.904723
117. Bo W, Lei M, Tao S, et al. Effects of combined intervention of physical exercise and cognitive training on cognitive function in stroke survivors with vascular cognitive impairment: a randomized controlled trial. *Clin Rehabil*. 2019;33(1):54-63. doi:10.1177/0269215518791007
118. Shokri G, Mohammadian F, Noroozian M, Amani-Shalamzari S, Suzuki K. Effects of remote combine exercise-music training on physical and cognitive performance in patients with Alzheimer's disease: a randomized controlled trial. *Front Aging Neurosci*. 2023;15(January):1-9. doi:10.3389/fnagi.2023.1283927
119. Greblo Jurakic Z, Krizanic V, Sarabon N, Markovic G. Effects of feedback-based balance and core resistance training vs. Pilates training on cognitive functions in older women with mild cognitive impairment: a pilot randomized controlled trial. *Aging Clin Exp Res*. 2017;29(6):1295-1298. doi:10.1007/s40520-017-0740-9

120. Sobol NA, Hoffmann K, Frederiksen KS, et al. Effect of aerobic exercise on physical performance in patients with Alzheimer's disease. *Alzheimer's and Dementia*. 2016;12(12):1207-1215. doi:10.1016/j.jalz.2016.05.004
121. Zhang Q, Zhu M, Huang L, et al. A Study on the Effect of Traditional Chinese Exercise Combined With Rhythm Training on the Intervention of Older Adults With Mild Cognitive Impairment. *Am J Alzheimers Dis Other Demen*. 2023;38(48):1-12. doi:10.1177/15333175231190626
122. Ghahfarrokhi MM, Shirvani H, Rahimi M, Bazgir B, Shamsadini A, Sobhani V. Feasibility and preliminary efficacy of different intensities of functional training in elderly type 2 diabetes patients with cognitive impairment: a pilot randomised controlled trial. *BMC Geriatr*. 2024;24(1):1-15. doi:10.1186/s12877-024-04698-8
123. Yan Y, Xu Y, Wang X, et al. The effect of multi-component exercise intervention in older people with Parkinson's disease and mild cognitive impairment: A randomized controlled study. *Geriatr Nurs (Minneap)*. 2024;60:137-145. doi:10.1016/j.gerinurse.2024.08.028
124. David S, Costa AS, Hohenfeld C, et al. Modulating effects of fitness and physical activity on Alzheimer's disease: Implications from a six-month randomized controlled sports intervention. *Journal of Alzheimer's Disease*. 2025;103(2):552-569. doi:10.1177/13872877241303764
125. Yang JG, Thapa N, Park HJ, et al. Virtual Reality and Exercise Training Enhance Brain, Cognitive, and Physical Health in Older Adults with Mild Cognitive Impairment. *Int J Environ Res Public Health*. 2022;19(20). doi:10.3390/ijerph192013300
126. Uemura K, Doi T, Shimada H, et al. Effects of Exercise Intervention on Vascular Risk Factors in Older Adults with Mild Cognitive Impairment: A Randomized Controlled Trial. *Dement Geriatr Cogn Dis Extra*. 2012;2(1):445-455. doi:10.1159/000343486
127. Li PWC, Yu DSF, Siu PM, Wong SCK, Chan BS. Peer-supported exercise intervention for persons with mild cognitive impairment: A waitlist randomised controlled trial (the BRAin Vitality Enhancement trial). *Age Ageing*. 2022;51(10):1-10. doi:10.1093/ageing/afac213
128. Avenali M, Picascia M, Tassorelli C, Sinforiani E, Bernini S. Evaluation of the efficacy of physical therapy on cognitive decline at 6-month follow-up in Parkinson disease patients with mild cognitive impairment: a randomized controlled trial. *Aging Clin Exp Res*. 2021;33(12):3275-3284. doi:10.1007/s40520-021-01865-4
129. Bademli K, Lok N, Canbaz M, Lok S. Effects of Physical Activity Program on cognitive function and sleep quality in elderly with mild cognitive impairment: A randomized controlled trial. *Perspect Psychiatr Care*. 2019;55(3):401-408. doi:10.1111/ppc.12324
130. Lok N, Tosun AS, Lok S, Temel V, Aydın Z. Effect of physical activity program applied to patients with Alzheimer's disease on cognitive functions and depression level: a randomised controlled study. *Psychogeriatrics*. 2023;23(5):856-863. doi:10.1111/psyg.13010
131. De Sá CA, Saretto CB, Cardoso AM, Remor A, Breda CO, da Silva Corralo V. Effects of a physical exercise or motor activity protocol on cognitive function, lipid profile, and BDNF levels in older adults with mild cognitive impairment. *Mol Cell Biochem*. 2024;479(3):499-509. doi:10.1007/s11010-023-04733-z
132. Padala KP, Padala PR, Lensing SY, et al. Home-Based Exercise Program Improves Balance and Fear of Falling in Community-Dwelling Older Adults with Mild Alzheimer's Disease: A Pilot Study. *Journal of Alzheimer's Disease*. 2017;59(2):565-574. doi:10.3233/JAD-170120
133. Langoni CDS, Resende TDL, Barcellos AB, et al. Effect of Exercise on Cognition, Conditioning, Muscle Endurance, and Balance in Older Adults with Mild Cognitive Impairment: A Randomized Controlled Trial. *Journal of Geriatric Physical Therapy*. 2019;42(2):E15-E22. doi:10.1519/JPT.0000000000000191
134. Vreugdenhil A, Cannell J, Davies A, Razay G. A community-based exercise programme to improve functional ability in people with Alzheimer's disease: A randomized controlled trial. *Scand J Caring Sci*. 2012;26(1):12-19. doi:10.1111/j.1471-6712.2011.00895.x

135. Dawson N, Judge KS, Gerhart H. Improved Functional Performance in Individuals with Dementia after a Moderate-Intensity Home-Based Exercise Program: A Randomized Controlled Trial. *Journal of Geriatric Physical Therapy*. 2019;42(1):18-27. doi:10.1519/JPT.0000000000000128
136. Santana-Sosa E, Barriopedro MI, López-Mojares LM, Pérez M, Lucia A. Exercise training is beneficial for Alzheimer's patients. *Int J Sports Med*. 2008;29(10):845-850. doi:10.1055/s-2008-1038432
137. Verdelho A, Correia M, Gonçalves-Pereira M, et al. Physical Activity in Mild Vascular Cognitive Impairment: Results of the AFIVASC Randomized Controlled Trial at 6 Months. *Journal of Alzheimer's Disease*. 2024;101(4):1379-1392. doi:10.3233/JAD-240246
138. Doi T, Makizako H, Shimada H, et al. Effects of multicomponent exercise on spatial-temporal gait parameters among the elderly with amnestic mild cognitive impairment (aMCI): Preliminary results from a randomized controlled trial (RCT). *Arch Gerontol Geriatr*. 2013;56(1):104-108. doi:10.1016/j.archger.2012.09.003
139. Shimada H, Suzuki T, Makizako H, et al. Effects of multicomponent exercise on cognitive function in older adults with amnestic mild cognitive impairment: a randomized controlled trial. *Alzheimer's & Dementia*. 2012;8(4S_Part_4). doi:10.1016/j.jalz.2012.05.386
140. Papatsimpas V, Vrouva S, Papathanasiou G, et al. Does Therapeutic Exercise Support Improvement in Cognitive Function and Instrumental Activities of Daily Living in Patients with Mild Alzheimer's Disease? A Randomized Controlled Trial. *Brain Sci*. 2023;13(7). doi:10.3390/brainsci13071112
141. Papamichail P, Sagredaki ML, Bouzineki C, Kanellopoulou S, Lyros E, Christakou A. The Effectiveness of an Exercise Program on Muscle Strength and Range of Motion on Upper Limbs, Functional Ability and Depression at Early Stage of Dementia. *J Clin Med*. 2024;13(14):1-10. doi:10.3390/jcm13144136
142. Rivas-Campo Y, Aibar-Almazán A, Afanador-Restrepo DF, et al. Effects of High-Intensity Functional Training (HIFT) on the Functional Capacity, Frailty, and Physical Condition of Older Adults with Mild Cognitive Impairment: A Blind Randomized Controlled Clinical Trial. *Life*. 2023;13(5):1-16. doi:10.3390/life13051224
143. Prick AE, De Lange J, Scherder E, Twisk J, Pot AM. The effects of a multicomponent dyadic intervention with physical exercise on the cognitive functioning of people with dementia: A randomized controlled trial. *J Aging Phys Act*. 2017;25(4):539-552. doi:10.1123/japa.2016-0038
144. Lamb SE, Sheehan B, Atherton N, et al. Dementia And Physical Activity (DAPA) trial of moderate to high intensity exercise training for people with dementia: Randomised controlled trial. *BMJ (Online)*. 2018;361. doi:10.1136/bmj.k1675
145. Kovas et al. Effects of a multimodal exercise program on balance, functional mobility and fall risk in older adults with cognitive impairment: a randomized controlled single-blind study. 2011;47(3):381-390.
146. de Oliveira Silva F, Ferreira JV, Plácido J, et al. Three months of multimodal training contributes to mobility and executive function in elderly individuals with mild cognitive impairment, but not in those with Alzheimer's disease: A randomized controlled trial. *Maturitas*. 2019;126(April):28-33. doi:10.1016/j.maturitas.2019.04.217
147. Fonte C, Smania N, Pedrinolla A, et al. Comparison between physical and cognitive treatment in patients with MCI and Alzheimer's disease. *Aging*. 2019;11(10):3138-3155. doi:10.18632/aging.101970
148. Akbuga Koc E, Yazici-Mutlu Ç, Cinar N, Sahiner T. Comparison of the effect of online physical exercise and computerized cognitive stimulation in patients with Alzheimer's disease during the Covid-19 pandemic. *Complement Ther Clin Pract*. 2024;57(May):10-20. doi:10.1016/j.ctcp.2024.101881

149. Shaw I, Cronje M, Shaw BS. Group-based exercise as a therapeutic strategy for the improvement of mental outcomes in mild to moderate alzheimer's patients in low resource care facilities. *Asian J Sports Med*. 2021;12(1):1-6. doi:10.5812/asjsm.106593
150. Cezar NO de C, Ansai JH, Oliveira MPB de, et al. Feasibility of improving strength and functioning and decreasing the risk of falls in older adults with Alzheimer's dementia: a randomized controlled home-based exercise trial. *Arch Gerontol Geriatr*. 2021;96(March). doi:10.1016/j.archger.2021.104476
151. Stevens J, Killeen M. A randomised controlled trial testing the impact of exercise on cognitive symptoms and disability of residents with dementia. *Contemporary nurse : a journal for the Australian nursing profession*. 2006;21(1):32-40. doi:10.5172/conu.2006.21.1.32
152. Ullrich P, Werner C, Schönstein A, et al. Effects of a Home-Based Physical Training and Activity Promotion Program in Community-Dwelling Older Persons with Cognitive Impairment after Discharge from Rehabilitation: A Randomized Controlled Trial. *Journals of Gerontology - Series A Biological Sciences and Medical Sciences*. 2022;77(12):2435-2444. doi:10.1093/gerona/glac005
153. Hauer K, Schwenk M, Zieschang T, Essig M, Becker C, Oster P. Physical training improves motor performance in people with dementia: A randomized controlled trial. *J Am Geriatr Soc*. 2012;60(1):8-15. doi:10.1111/j.1532-5415.2011.03778.x
154. Suttanon P, Hill KD, Said CM, et al. Feasibility, safety and preliminary evidence of the effectiveness of a home-based exercise programme for older people with Alzheimer's disease: A pilot randomized controlled trial. *Clin Rehabil*. 2013;27(5):427-438. doi:10.1177/0269215512460877
155. Sanders LMJ, Hortobágyi T, Karssemeijer EGA, Van Der Zee EA, Scherder EJA, Van Heuvelen MJG. Effects of low- And high-intensity physical exercise on physical and cognitive function in older persons with dementia: A randomized controlled trial. *Alzheimers Res Ther*. 2020;12(1):1-15. doi:10.1186/s13195-020-00597-3
156. Levinger P, Goh AMY, Dunn J, et al. Exercise interveNtion outdoor proJect in the cOmmunitY – results from the ENJOY program for independence in dementia: a feasibility pilot randomised controlled trial. *BMC Geriatr*. 2023;23(1):1-16. doi:10.1186/s12877-023-04132-5
157. Casas-Herrero Á, Sáez de Asteasu ML, Antón-Rodrigo I, et al. Effects of Vivifrail multicomponent intervention on functional capacity: a multicentre, randomized controlled trial. *J Cachexia Sarcopenia Muscle*. 2022;13(2):884-893. doi:10.1002/jcsm.12925
158. Mollinedo Cardalda I, López A, Cancela Carral JM. The effects of different types of physical exercise on physical and cognitive function in frail institutionalized older adults with mild to moderate cognitive impairment. A randomized controlled trial. *Arch Gerontol Geriatr*. 2019;83(May):223-230. doi:10.1016/j.archger.2019.05.003
159. Kim MJ, Han CW, Min KY, et al. Physical Exercise with Multicomponent Cognitive Intervention for Older Adults with Alzheimer's Disease: A 6-Month Randomized Controlled Trial. *Dement Geriatr Cogn Dis Extra*. 2016;6(2):222-232. doi:10.1159/000446508
160. Bossers WJR, Van Der Woude LHV, Boersma F, Hortobágyi T, Scherder EJA, Van Heuvelen MJG. A 9-Week Aerobic and Strength Training Program Improves Cognitive and Motor Function in Patients with Dementia: A Randomized, Controlled Trial. *American Journal of Geriatric Psychiatry*. 2015;23(11):1106-1116. doi:10.1016/j.jagp.2014.12.191
161. Toots A, Littbrand H, Boström G, et al. Effects of exercise on cognitive function in older people with dementia: A randomized controlled trial. *Journal of Alzheimer's Disease*. 2017;60(1):323-332. doi:10.3233/JAD-170014
162. Henskens M, Nauta IM, Van Eekeren MCA, Scherder EJA. Effects of Physical Activity in Nursing Home Residents with Dementia: A Randomized Controlled Trial. *Dement Geriatr Cogn Disord*. 2018;46(1-2):60-80. doi:10.1159/000491818

163. Kemoun G, Thibaud M, Roumagne N, et al. Effects of a physical training programme on cognitive function and walking efficiency in elderly persons with dementia. *Dement Geriatr Cogn Disord*. 2010;29(2):109-114. doi:10.1159/000272435
164. Rolland Y, Pillard F, Klapouszczak A, et al. Exercise program for nursing home residents with Alzheimer's disease: A 1-year randomized, controlled trial. *J Am Geriatr Soc*. 2007;55(2):158-165. doi:10.1111/j.1532-5415.2007.01035.x
165. Brett L, Stapley P, Meedya S, Traynor V. Effect of physical exercise on physical performance and fall incidents of individuals living with dementia in nursing homes: a randomized controlled trial. *Physiother Theory Pract*. 2021;37(1):38-51. doi:10.1080/09593985.2019.1594470
166. Almeida S, Paixão C, da Silva MG, Marques A. Lifestyle-integrated functional exercise for people with Dementia: A pilot study. *J Aging Phys Act*. 2021;29(5):771-780. doi:10.1123/JAPA.2020-0349
167. Mak A, Delbaere K, Refshauge K, et al. Sunbeam Program Reduces Rate of Falls in Long-Term Care Residents With Mild to Moderate Cognitive Impairment or Dementia: Subgroup Analysis of a Cluster Randomized Controlled Trial. *J Am Med Dir Assoc*. 2022;23(5):743-749.e1. doi:10.1016/j.jamda.2022.01.064
168. Gebhard D, Mess F. Feasibility and Effectiveness of a Biography-Based Physical Activity Intervention in Institutionalized People With Dementia: Quantitative and Qualitative Results From a Randomized Controlled Trial. *J Aging Phys Act*. 2022;30(2):237-251. doi:10.1123/japa.2020-0343
169. Telenius EW, Engedal K, Bergland A. Long-term effects of a 12 weeks high-intensity functional exercise program on physical function and mental health in nursing home residents with dementia: A single blinded randomized controlled trial Physical functioning, physical health and activity. *BMC Geriatr*. 2015;15(1):1-11. doi:10.1186/s12877-015-0151-8
170. de Souto Barreto P, Cesari M, Denormandie P, Armaingaud D, Vellas B, Rolland Y. Exercise or Social Intervention for Nursing Home Residents with Dementia: A Pilot Randomized, Controlled Trial. *J Am Geriatr Soc*. 2017;65(9):E123-E129. doi:10.1111/jgs.14947
171. Li L, Liu M, Zeng H, Pan L. Multi-component exercise training improves the physical and cognitive function of the elderly with mild cognitive impairment: A six-month randomized controlled trial. *Ann Palliat Med*. 2021;10(8):8919-8929. doi:10.21037/apm-21-1809
172. Roach KE, Tappen RM, Kirk-Sanchez N, Williams CL, Loewenstein D. A randomized controlled trial of an activity specific exercise program for individuals with alzheimer disease in long-term care settings. *Journal of Geriatric Physical Therapy*. 2011;34(2):50-56. doi:10.1519/JPT.0b013e31820aab9c
