# Supplementary material for: Dance versus other exercise modalities in mild cognitive impairment and dementia: comparative efficacy from a systematic review and bayesian network meta-analysis
Source: Front Physiol. 2026 Mar 25;17:1782774. doi: 10.3389/fphys.2026.1782774 (PMC13056856; doi:10.3389/fphys.2026.1782774)
Supplement: Supplementary file 9 [file Table8.pdf]

Supplementary Table 8. Risk of Bias Assessment Results

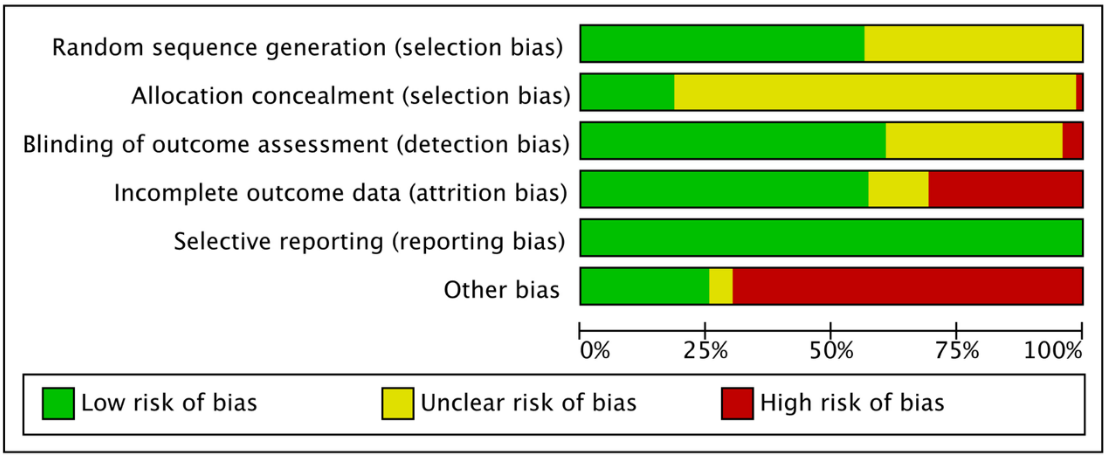

Risk of Bias Graph

	Random sequence generation (selection bias)	Allocation concealment (selection bias)	Blinding of outcome assessment (detection bias)	Incomplete outcome data (attrition bias)	Selective reporting (reporting bias)	Other bias
Abbas et al.2023	+	?	+	?	+	+
Abd El-Kader and Al-Jiffri.2016	?	?	?	+	+	−
Almeida et al.2021	+	?	+	+	+	−
Amjad et al.2018	?	?	?	+	+	+
Angiolillo et al.2023	+	?	+	−	+	−
Arcoverde et al.2013	?	?	+	+	+	+
Avenali et al.2021	+	?	+	+	+	−
Bademli et al.2018	+	?	−	+	+	+
Baek et al.2024	?	+	+	+	+	−
Baker et al.2010	?	?	+	−	+	+
Baker et al.2025	+	?	+	+	+	+
Barreto et al.2017	+	+	?	+	+	−
Bisbe et al.2020	+	+	+	−	+	+
Blumen et al.2023	+	?	+	−	+	+
Bo et al.2019	+	?	+	−	+	+
Bossers et al.2014	?	?	+	+	+	−
Bracco et al.2023	+	?	−	+	+	−
Brett et al.2021	+	?	?	−	+	−
Brydges et al.2020	?	?	?	?	+	−
Cancela et al.2016	+	?	−	+	+	−
Cardalda et al.2019	+	?	?	+	+	−
Casas–Herrero et al.2022	+	?	+	−	+	−

Cezar et al.2021	+	+	+	−	+	−
Chan et al.2016	+	?	+	−	+	−
Chang et al.2021	+	?	+	−	+	−
Chang et al.2024	?	?	+	−	+	+
Chen et al.2023	+	?	+	+	+	+
Cheng et al.2014	?	?	−	+	+	−
Choi et al.2018	+	?	?	+	+	−
Damirchi et al.2017	?	?	?	−	+	−
David et al.2025	+	?	?	+	+	−
Dawson et al.2019	?	?	?	+	+	+
De Sa et al.2024	?	+	+	−	+	−
Dillon and Prapavessis.2021	?	?	?	−	+	+
Doi et al.2013	?	?	?	+	+	+
Doi et al.2017	+	?	+	−	+	+
Donnezan et al.2018	?	?	?	−	+	?
Eggenberger et al.2015	+	?	−	−	+	−
Eggermont et al.2009	?	?	+	?	+	−
Enette et al.2020	?	?	?	−	+	−
Esmail et al.2020	+	−	+	−	+	−
Eyre et al.2017	+	?	+	−	+	−
Fernandez-Gonzalo et al.2016	?	?	?	+	+	−
Fischbacher et al.2020	+	?	+	−	+	−
Fonte et al.2019	?	?	+	+	+	−
Franco et al.2020	+	+	+	+	+	−
Gao et al.2024	+	+	+	+	+	−
Gebhard and Mess.2022	?	?	?	−	+	−
Ghahfarrokhi et al.2024	+	+	+	+	+	?
Grzenda et al.2024	?	?	?	?	+	−
Guzel et al.2024	+	?	+	−	+	−
Hauer et al.2012	?	?	+	+	+	−
Henskens et al.2018	+	?	?	−	+	−
Ho et al.2018	+	?	+	?	+	−

Holthoff et al.2015	?	?	?	+	+	+
Hong et al.2018	?	?	?	+	+	−
Hsu et al.2018	+	?	?	−	+	−
Hsu et al.2021	+	+	?	+	+	−
Huang et al.2019	+	+	?	+	+	−
Huang et al.2025	+	+	?	−	+	−
Hughes et al.2014	+	?	?	+	+	−
Ihle-Hansen et al.2019	+	?	+	+	+	−
Jiayuan et al.2022	+	?	+	+	+	+
Jurakic et al.2017	?	?	?	?	+	−
Karssemeijer et al.2019	?	?	+	+	+	+
Karthikeyan T.2020	?	?	?	?	+	+
Kashyap et al.2022	+	?	?	−	+	−
Kemoun et al.2010	?	?	?	+	+	−
Khanthong et al.2021	?	?	+	−	+	−
Khattak et al.2022	?	?	?	?	+	?
Kim and Yim.2017	?	?	?	+	+	−
Kim et al.2016	+	+	+	−	+	−
Koc et al.2024	?	?	+	+	+	−
Kohanpour et al.2017	?	?	?	?	+	−
Kovacs et al.2013	?	?	+	−	+	−
Krootnark et al.2024	+	+	+	+	+	−
Kropacova et al.2019	+	−	+	−	+	−
Kusleikiene et al.2025	+	?	?	−	+	+
Lamb et al.2018	+	?	+	+	+	−
Lam et al.2012	?	?	+	+	+	−
Langoni et al.2019	?	?	+	+	+	−
Law et al.2021	+	?	+	+	+	−
Lazarou et al.2017	+	?	+	−	+	−
Lee et al.2020	?	?	?	+	+	−
Levinger et al.2023	+	?	−	−	+	−
Li (Baduanjin) et al.2022	+	+	+	+	+	+

Li (CE) et al.2021	?	?	+	+	+	−
Li (CE) et al.2022	+	+	+	+	+	−
Li (Tai Chi) et al.2021	+	?	+	+	+	−
Li (Tai Chi) et al.2022	+	+	+	+	+	+
Liao et al.2021	+	?	+	−	+	−
Lin et al.2024	+	?	+	+	+	+
Liu et al.2018	+	+	+	+	+	−
Liu et al.2020	?	?	+	+	+	−
Liu et al.2022	+	?	+	+	+	−
Lok et al.2023	+	?	?	+	+	+
Lowery et al.2013	+	?	+	+	+	+
Lu et al.2016	+	?	+	+	+	−
Luo et al.2022	?	?	?	+	+	−
Mak et al.2022	?	?	+	−	+	−
Makino et al.2021	?	?	+	+	+	+
Miu et al.2008	?	?	+	+	+	+
Morris et al.2017	+	+	+	+	+	+
Nagamatsu et al.2013	+	?	+	−	+	−
Nakatsuka et al.2015	?	?	?	−	+	+
Nyman et al.2019	+	+	+	+	+	−
Okuyan and Deveci.2020	+	?	+	+	+	−
Padala et al.2012	+	?	?	+	+	−
Padala et al.2017	+	+	−	+	+	−
Papamichail et al.2024	+	?	+	?	+	−
Papatsimpas et al.2023	+	?	+	+	+	−
Phoemsapthawee et al.2016	?	?	?	?	+	−
Prick et al.2017	+	?	+	+	+	−
Qi et al.2018	?	?	?	+	+	−
Rivas-Campo et al.2023	+	?	+	+	+	−
Roach et al.2011	?	?	+	?	+	?
Rojasavastera et al.2020	?	?	+	+	+	−
Rolland et al.2007	+	?	+	+	+	−

Sanchez-Alcala et al.2025	+	+	+	+	+	−
Sanders et al.2020	?	?	+	+	+	−
Santana-Sosa et al.2008	?	?	?	?	+	−
Santen et al.2020	+	?	?	−	+	+
Scherder et al.2005	?	?	+	?	+	−
Schwenk et al.2016	+	?	?	+	+	+
Shaw et al.2021	+	?	?	?	+	?
Shimada et al.2018	+	?	+	+	+	+
Shokri et al.2024	?	?	+	−	+	?
Silva et al.2019	?	?	+	?	+	+
Singh et al.2014	+	+	+	−	+	−
Sobol et al.2016	?	?	+	+	+	−
Song et al.2019	+	+	+	+	+	−
Song et al.2024	+	+	+	−	+	−
Stevens and Killeen.2006	+	?	?	?	+	−
Stuckenschneider et al.2021	?	?	?	+	+	+
Su et al.2021	+	?	?	+	+	+
Sungkarat et al.2018	+	+	+	+	+	−
Suttanon et al.2012	+	+	+	+	+	−
Suzuki et al.2012	?	?	?	+	+	+
Swinnen et al.2021	?	?	?	−	+	−
Telenius et al.2015	?	+	+	+	+	−
Thiel et al.2024	?	?	?	+	+	+
Tomoto et al.2021	?	?	+	?	+	+
Toots et al.2017	+	?	+	+	+	−
Tremont et al.2022	+	?	+	+	+	−
Tsai et al.2019	?	?	?	+	+	−
Uemura et al.2012	?	?	+	+	+	+
Ugur and Sertel 2025	+	+	+	+	+	−
Ullrich et al.2022	?	?	+	+	+	−
Varela et al.2011	?	?	?	+	+	+
Venturelli et al.2010	?	?	?	−	+	?

Venturelli et al.2011	?	?	?	+	+	?
Verdelho et al.2024	+	?	+	+	+	+
Vints et al.2024	+	?	+	-	+	+
Vreugdenhil et a.2012	?	?	+	+	+	-
Wang et al.2020	+	+	+	+	+	-
Wei and Ji.2014	?	?	?	?	+	-
Winckel et al.2004	+	?	?	+	+	-
Wu et al.2023	+	?	+	-	+	-
Yan et al.2024	+	?	+	+	+	-
Yang et al.2015	?	?	?	?	+	-
Yang et al.2022	?	?	?	+	+	-
Yoon et al.2017	?	?	?	-	+	-
Yoon et al.2018	?	?	?	-	+	+
Yu (AE) et al. 2022	+	?	+	-	+	-
Yu (Tai Chi) et al.2022	+	?	+	-	+	-
Yu et al.2021	?	?	+	?	+	+
Zhang et al.2023	?	?	?	-	+	-
Zheng et al.2020	+	+	+	-	+	-
Zheng et al.2021	?	?	+	+	+	-
Zheng et al.2022	+	?	+	-	+	-
Zhu et al.2018	+	+	+	+	+	-
Zhu et al.2022	+	+	+	+	+	-

Risk of Bias Summary
