# Supplementary material for: Dance versus other exercise modalities in mild cognitive impairment and dementia: comparative efficacy from a systematic review and bayesian network meta-analysis
Source: Front Physiol. 2026 Mar 25;17:1782774. doi: 10.3389/fphys.2026.1782774 (PMC13056856; doi:10.3389/fphys.2026.1782774)
Supplement: Supplementary file 10 [file Table10.pdf]

**Supplementary Table. 10 Recommendation for Interventions**

**Recommendations for Exercise-based Interventions**

| Domain | Details |
|---|---|
| **Individualization** | a) Conduct health assessments to identify contraindications (e.g., cardiovascular disease, musculoskeletal disorders, and medications) that could affect training activities.<br>b) Tailored intervention to baseline physical function, activity levels, cognition, lifestyle, and preferences.<br>c) Emphasize participants' autonomy and choice.<br>d) Use simplified instructions, haptic feedback, and mirror techniques.<br>e) Foster supportive, enjoyable, and socially engaging environment. |
| **Optional Exercises** | a) **Dance**<br>b) **Aerobic exercise:** walking, jogging, swimming, treadmill, cycling, elliptical training, etc.<br>c) **Resistance Exercise:** resistance bands, dumbbells, or body weight (e.g., curls and squats).<br>d) **Multicomponent Exercise:** combines AE, RE, balance, coordination, and stretching training.<br>e) **Exergaming:** interactive exercise gaming with cognitive benefits (e.g., Wii and Kinect).<br>f) **Yoga: Kundalini, Hatha yoga with** breathing/meditation.<br>g) **Chinese Traditional Exercise:** Tai Chi, Baduanjin, and Wuqinxi. |
| **Training Plan** | a) Pre-session orientation: introduce benefits, procedures, and tools (e.g., HR monitor and RPE scale).<br>b) Adaptation period: 1–2 weeks.<br>c) Session structure (60 mins)<br>• Warm-up (10 mins): breathing exercises, muscle stretching, and joint mobilization.<br>• Main training (40 mins): split into two 20-min blocks, with a 5~10-min break.<br>• Cool-down (10 mins): deep breathing, relaxation, and stretching.<br>• Optional meditation (10~15 mins): integrated into yoga or Tai Chi activities.<br>• Social interaction (10 mins): feedback and social interaction between participants and instructors.<br>• Post-session support: provide recorded routines and materials for independent practice.<br>• Lifestyle maintenance: continue regular diet and medical routines. |
| **Training Intensity** | a) Progressive loading based on capacity (e.g., 57%–95% HRmax, 30%–89% HRR, or 50%–85% $VO_2$max).<br>a) Duration: 12 weeks of programs.<br>b) Frequency: three sessions/week.<br>b) Session length: 60 mins. |
| **Training Flexibility** | a) Reassess every three months to adjust training plans.<br>b) Offer sessions at various times of day.<br>c) Encourage breaks and hydration as needed. |

| | |
|---|---|
| **Individual vs. Group Exercise** | a) Group sessions (2 to 15 participants) to promote socialization.<br>b) Individual sessions may suit those with health limitations or personal preferences. |
| **Training Monitoring Methods** | a) Heart rate (HR)<br>b) Rating of perceived exertion (RPE)<br>c) Blood pressure (BP)<br>d) "Talk" test<br>e) Pedometer<br>f) Practice diary/log |
| **Instructor Qualification** | a) Suitable professionals: physiotherapists, occupational therapists, certified fitness trainers, nurses, kinesiologists, gerontologists, and trained nursing home staff.<br>b) Ensure consistency and standardization by assigning a single instructor per program.<br>c) Instructors should be specialized in cognitive impairment care and adapted communication techniques as well as motivational strategies. |
| **Motivational & Adherence Strategies** | a) Using co-instructors for support.<br>b) Incorporate multisensory cues (e.g., visual, verbal, and tactile).<br>c) Use props to increase engagement and enjoyment.<br>d) Implement a rewards system.<br>e) Offer remote check-ins (e.g., calls and text messages) to encourage adherence.<br>f) To train caregivers to supervise and maintain safety.<br>g) Display exercise schedules at home.<br>h) Schedule periodic follow-up visits. |
| **Music** | Use personalized playlists with meaningful or familiar songs to improve motivation and memory. |
| **Possible Equipment** | a) Training: treadmills, bikes, ellipticals, leg presses, dumbbells<br>b) Support: mirrors, chairs, projectors, and computers.<br>c) Monitoring: HR monitors, RPE scales, and pedometers.<br>d) Exergaming: Nintendo Wii™, Kinect, ExerHeart, DividatSenso, and StepMania<br>a) Software: E-Prime 2.0, LongGood, and Zoom platform |
| **Training Environment** | a) Venues may include studios, community centers, gyms, senior care facilities, parks, and recreation clubs.<br>b) For home-based training, ensure non-slip flooring, obstacle-free space, and adequate movement area. |

**Recommendations for Dance-Based Interventions**

| Domain | Recommendations |
|---|---|
| **Dance Types** | *Dance Movement Therapy (DMT):*<br>DMT should adhere to standards established by the American Dance Therapy Association (ADTA).<br>*General Dance Interventions:*<br>a) Balance complexity, repetition, and variety in dance routines to engage in memory, concentration, and dual task functioning.<br>b) Ensure participant acceptability by alternating fast/slow rhythms and combining solo/paired movements.<br>c) Emphasize strength, balance, trunk mobility, and flexibility.<br>d) Recommended dance styles included:<br>1) Choreographed dance: specifically designed to align with participants' physical and cognitive abilities.<br>2) Improvisational dance: suitable for participants with prior dance experience and skills.<br>3) Culturally relevant dances (e.g., Irish country dance, African dance, and Greek traditional dance).<br>4) International ballroom dances (e.g., Tango, Waltz, Viennese Waltz, Foxtrot, Rumba, and Cha-Cha-Cha).<br>5) Chinese square dancing: characterized by movements such as hand clapping, high-fiving, chest expansion, arm extension, and leg kicking.<br>6) Aerobic dance: rhythmic, full-body exercise set to music that engages large muscle groups, incorporating movements such as knee bending, heel lifts, boxing, shoulder movements, kicking, square stepping, sculling, and jumping, as well as daily activity-like gestures (e.g., mimicking hand washing).<br>7) Other styles (e.g., Salsa, Rock, Pop, East Coast Swing, Merengue, Disco-Hustle, Jitterbug, and Blues). |
| **Training Plan** | Suggested 60 mins dance session includes:<br>a) Warm-up (10 mins): light stepping, breathing exercises, head movements, and side bending and turning, accompanied by relaxing yet lively music to facilitate engagement.<br>b) Main dance (40 mins): two 20-min segments with a 5~10-min break.<br>c) Cool-down (10 mins): low intensity stepping, breathing exercises, shoulder movements, and gentle stretching, accompanied by soft music.<br>d) A 10~20-min daily home practice is recommended. |
| **Music** | a) Choose music based on cultural familiarity (preferably from childhood or young adulthood).<br>b) Match music to movement goals.<br>c) Use songs with lyrics to support memory and engagement. |
| **Dance Instructors** | a) For Dance Movement Therapy (DMT): registered therapist certified by ADTA or equivalent.<br>b) For general dance: instructors experienced with older adults (certified dance coaches, students majoring in sports, and physiotherapists).<br>c) To enhance accessibility, dance routines may also be delivered by trained nurses or assistants. |

| | |
|---|---|
| **Teaching Strategies** | a) Break routines into short, progressive segments, with new components gradually introduced to maintain participant engagement.<br>b) Repetitive is essential for learning; to avoid cognitive overload, at least one-third of each intervention session should be dedicated to instruction and practice, ensuring an enjoyable learning experience.<br>c) Use a stepwise instructional model:<br>1) Learning movements by imitating the instructor.<br>2) Performing movements with instructor guidance.<br>3) Practicing movements with both instructor support and music through repetition.<br>4) Performing movements with music only, without visual cues to reinforce memory and independence. |
